# Visible Light Photoredox-Catalyzed Decarboxylative Alkylation of 3-Aryl-Oxetanes and Azetidines via Benzylic Tertiary Radicals and Implications of Benzylic Radical Stability

**DOI:** 10.1021/acs.joc.3c00083

**Published:** 2023-03-03

**Authors:** Maryne A. J. Dubois, Juan J. Rojas, Alistair J. Sterling, Hannah C. Broderick, Milo A. Smith, Andrew J. P. White, Philip W. Miller, Chulho Choi, James J. Mousseau, Fernanda Duarte, James A. Bull

**Affiliations:** † Department of Chemistry, Imperial College London, Molecular Sciences Research Hub, White City Campus, Wood Lane, London W12 0BZ, U.K.; ‡ Department of Chemistry, Chemistry Research Laboratory, University of Oxford, Oxford OX1 3TA, U.K.; § Pfizer Global Research and Development, 445 Eastern Point Rd., Groton, Connecticut 06340, United States

## Abstract

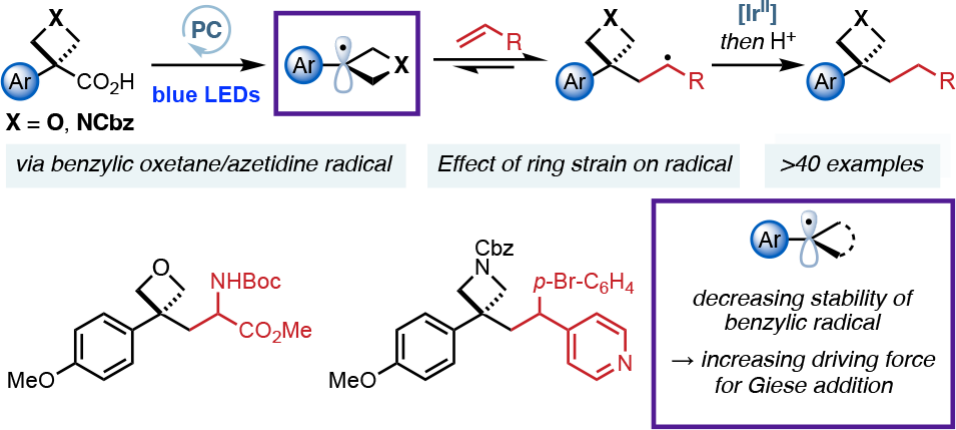

Four-membered heterocycles offer exciting potential as small polar motifs in medicinal chemistry but require further methods for incorporation. Photoredox catalysis is a powerful method for the mild generation of alkyl radicals for C–C bond formation. The effect of ring strain on radical reactivity is not well understood, with no studies that address this question systematically. Examples of reactions that involve benzylic radicals are rare, and their reactivity is challenging to harness. This work develops a radical functionalization of benzylic oxetanes and azetidines using visible light photoredox catalysis to prepare 3-aryl-3-alkyl substituted derivatives and assesses the influence of ring strain and heterosubstitution on the reactivity of small-ring radicals. 3-Aryl-3-carboxylic acid oxetanes and azetidines are suitable precursors to tertiary benzylic oxetane/azetidine radicals which undergo conjugate addition into activated alkenes. We compare the reactivity of oxetane radicals to other benzylic systems. Computational studies indicate that Giese additions of unstrained benzylic radicals into acrylates are reversible and result in low yields and radical dimerization. Benzylic radicals as part of a strained ring, however, are less stable and more π-delocalized, decreasing dimer and increasing Giese product formation. Oxetanes show high product yields due to ring strain and Bent’s rule rendering the Giese addition irreversible.

## Introduction

**Figure 1:**
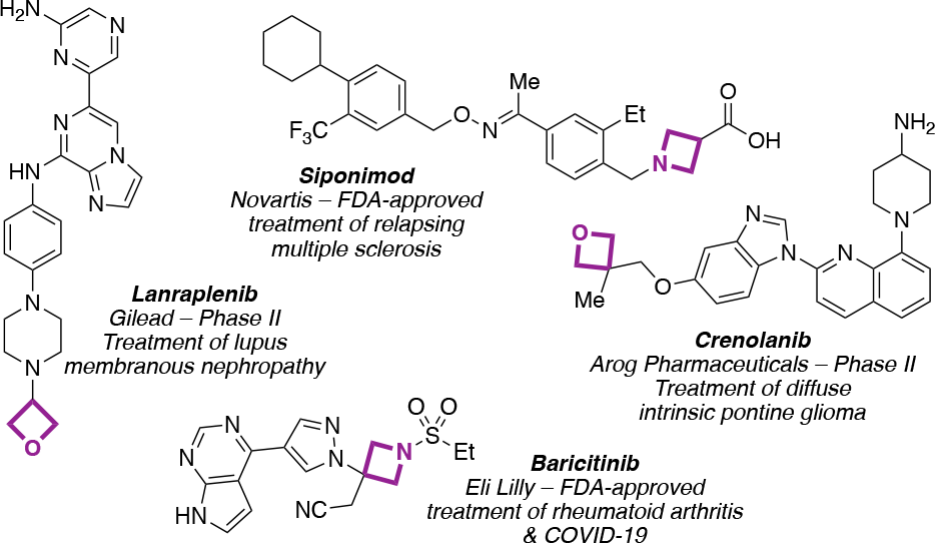
Oxetane- and azetidine-containing pharmaceuticals.

Oxetanes and azetidines continue to attract interest as valuable motifs in medicinal chemistry.^1^ These motifs have increasingly appeared in clinical candidates, including Lanraplenib,^2^ Crenolanib,^3^ and FDA-approved Siponimod^4^ and Baricitinib (Figure 1).^5^ The low molecular weight and high polarity of four-membered heterocycles can provide attractive molecular properties, as well as replacement groups for sensitive and metabolically exposed functionalities.^1,6^ 3,3-Disubstituted oxetanes, in particular, present interesting opportunities as bioisosteres, providing comparable features to carbonyl groups and advantages due to increased steric protection, which improves stability to nucleophiles and acidic conditions. The attractive features of four-membered rings have prompted the development of several new approaches for their synthesis and late-stage incorporation to overcome the challenges posed by their ring strain and potential instability.^7^

**Scheme 1:**
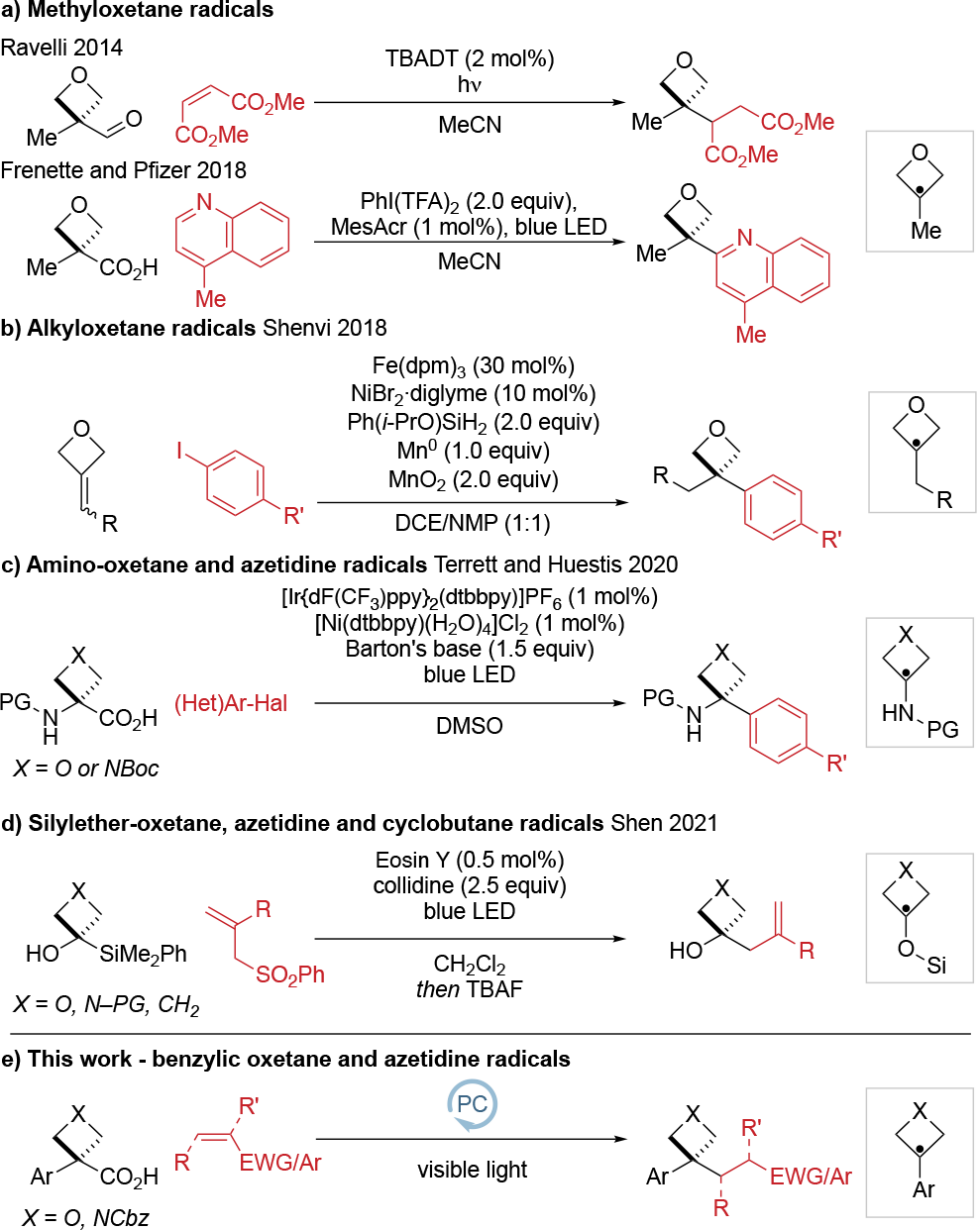
Strategies for the Formation of 3,3-Disubstituted Oxetanes and Azetidines by Radical Functionalization

Recent years have seen developments in the generation and reaction of oxetane and azetidine radicals as reactive intermediates. Radicals have been generated from oxetane itself and azetidine derivatives at the activated C2-position, where the radical is stabilized by the adjacent lone pair.^8^ Radical generation at the 3-position must compete with possible HAT processes at the 2-position, which would generate a heteroatom-stabilized radical, and hence requires a group that can act as a radical precursor.^9−17^ Recently, oxetane functionalization has been achieved using visible light mediated photoredox catalysis, which has emerged as a powerful and general tool to generate radical species under mild conditions.^18^ 3-Iodo-oxetane and -azetidine are increasingly employed in coupling reactions,^9−11^ while there are only limited examples from oxetane-3-carboxylic acid. To date there have been very few and isolated examples of tertiary oxetane radicals at the 3-position (Scheme 1).

In 2014, Ravelli reported the generation of 3-methyloxetane radicals by decarbonylation using UV light and TBADT ([(*n*-Bu)_4_N]_4_[W_10_O_32_]), and their reaction with Michael acceptors.^8a^ In 2018, Frenette and Pfizer developed visible light conditions for Minisci reactions.^12^ Also in 2018, Shenvi used HAT/Ni dual catalysis to hydroarylate oxetane alkylidenes with aryl halides via a 3-alkyloxetane radical.^14^ In 2020, Terrett and Huestis reported the synthesis of 3-aryl-3-amino oxetanes and azetidines through the photocatalytic generation and coupling of 3-amino radical intermediates with aryl halides.^15^ Very recently, 3-silyloxy azetidine and oxetane radicals were reported by Shen from 3-silyl azetidin-3-ols and oxetan-3-ols through a radical 1,2-silyl transfer which underwent C–C coupling with Michael acceptors.^16^

Following our interest in 3-aryloxetane and azetidine derivatives involving carbocation intermediates,^19^ we envisaged that benzylic oxetane radicals would broaden the range of options for oxetane incorporation and provide access to valuable, unexplored, and medicinally relevant chemical space under mild photoredox conditions. However, tertiary benzylic radicals remain underinvestigated in photoredox catalysis^20^ and might be expected to display low reactivity in their addition reactions due to their relatively stabilized nature.^21^ Further, benzylic radicals are prone to oxidation to form stabilized carbocations through radical-polar crossover pathways.^22^ To date, there have been no reports of the reaction of 3-aryloxetane or azetidine radicals, and the effect of the four-membered ring on the reactivity and radical structure was unclear at the outset of this work.

Here, we report our studies on the formation and reactions of oxetane and azetidine radicals using visible light mediated photoredox catalysis starting from carboxylic acid derivatives. We also report a systematic comparison of reaction outcomes for related substituted benzylic radicals and highlight important structural features for the reactivity of benzylic radicals. Most notably, we draw correlations between radical stability, hybridization, and the equilibrium of the reversible Giese addition, which determines product yields and the extent of radical dimerization.

## Results and Discussion

### Reaction Optimization and Scope

We first investigated several possible radical precursors, initially derived from oxetanols. Attempts to prepare 3-aryloxetane derivatives of typical radical precursors such as bromide and chloride were unsuccessful (Supporting Scheme S1), and similarly, borate or silicate derivatives are not readily available. Oxalates, as the acid or Cs-salt,^23^ were prone to hydrolysis under reaction conditions, and parallel screening of conditions did not provide a productive reaction (Supporting Scheme S2 and Table S1). We hence examined oxetane carboxylic acids. Carboxylic acids have been extensively used in the formation of tertiary radical centers under photocatalytic conditions.^18b,24−26^ Aryloxetane carboxylic acids were not available and prompted our recent report on their short two-step synthesis from 3-aryl-oxetan-3-ols involving catalytic Friedel–Crafts reaction followed by mild oxidative cleavage.^27,28^ Initial investigations with acid **1** were based on a report by MacMillan in 2014,^29^ which generated C(sp^3^) radicals under decarboxylative photoredox conditions and trapped these with electron-deficient alkenes.^30^ Electron-rich oxetane acid **1** was used as the model substrate, and ethyl acrylate, as radical acceptor. The major product of this reaction was 3,3-disubstituted oxetane **2a**, which was formed alongside dialkylated product **2a′**, dimer **3**, and reduced product **4**. After an extensive survey of discrete and continuous reaction variables (e.g., further bases, solvents, photocatalysts; Supplementary Tables S2–S5), desired 3,3-disubstituted oxetane **2a** was obtained in 61% yield (±5%, 95% confidence interval, *n* = 6, performed by three different chemists; Table 1, entry 1), with minimized formation of side products (**3**, **4**) and finally little deviation from the MacMillan conditions. The mass balance in the standard reaction between **1** and ethyl acrylate was variable depending on the purity of **1** used (*cf.*Table 1 entry 1 and Supporting Table S6). Nonetheless, complete conversion of starting material **1** is always observed. Deviations from an overall conserved mass balance of 100% are ascribed to intractable degradation products from the oxetane radical.

**Table 1:**
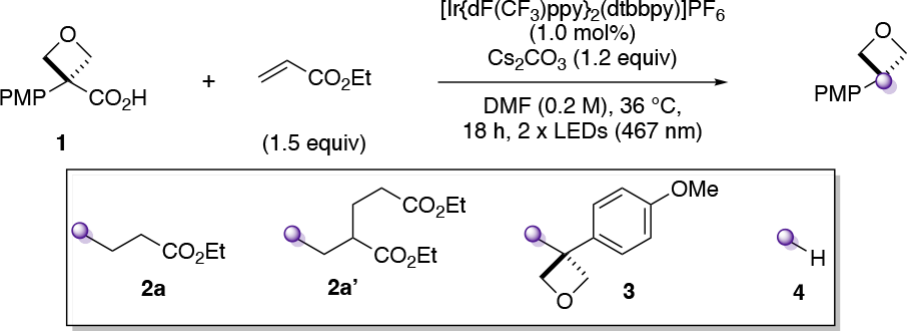
Selected Optimization Studies for the Reaction of Oxetane Acid **1** with Ethyl Acrylate

		yield (%)^a^			
entry	change from standard conditions	**2a**	**2a′**	**3**	**4**
1^b^	none	61 (58)	8 (8)	1	1
2	cat. = Ru(bpy)_3_(PF_6_)_2_	<5	0	2	0
3	cat. = Mes-Acr^+^	0	0	0	0
4	0.5 mol % Ir cat.	46	11	2	0
5	28 °C (fan cooling)	54	11	3	0
6	solvent = MeCN	41	7	2	1
7	solvent = 1,4-dioxane	28	3	3	1
8	[0.1 M]	59	15	2	3
9	2.0 equiv of acrylate	59	7	1	1
10	0.7 equiv of acrylate	59^c^	8^c^	2	3
11	DBU as base	39	4^c^	2	1
12	no photocatalyst	0	0	0	0
13	no light	0	0	0	0
14	no base	0	0	0	0

a Reactions run on a 0.2 mmol scale under argon. Yield calculated by analysis of the ^1^H NMR spectrum of the crude mixture of the reaction using 1,3,5-trimethoxybenzene as internal standard and a 30 s relaxation delay (d1).

b Reported yields are the mean average of 6 experiments, isolated yields of a single run are in parentheses.

c Yields vs ethyl acrylate.

**Scheme 2:**
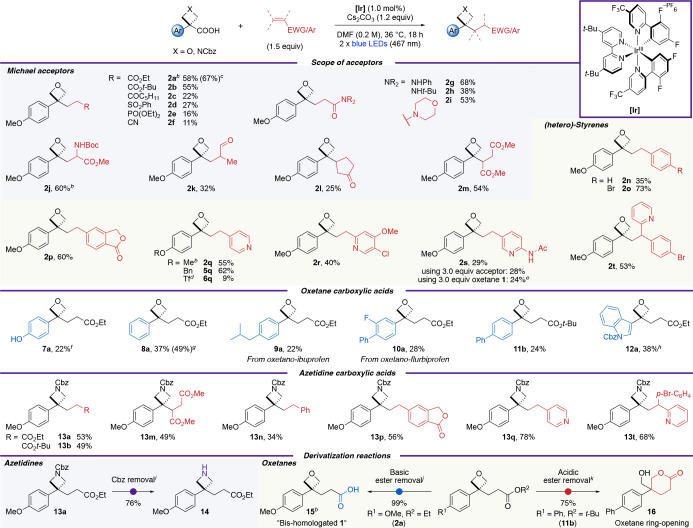
Reaction Scope of Oxetane and Azetidine Acids and Alkenes Reactions run on a 0.20 mmol scale unless otherwise stated. Isolated yields are reported. Characterized by X-ray crystallography. Using repurified **1** (Supporting Table S6). Isolated in 70% purity. Yield based on alkene (0.20 mmol scale). Using TIPS-protected oxetane acid. Using repurified Ph oxetane carboxylic acid, on a 0.10 mmol scale and run for 14 h (Supporting Table S14). 0.10 mmol scale. H_2_, Pd/C (10% *w/w*, 10 mol % Pd), EtOH, 25 °C, 22 h. LiOH (3.0 equiv), H_2_O:THF:MeOH (3:1:1), 25 °C, 24 h. Trifluoroacetic acid (10 equiv), CH_2_Cl_2_, 0–25 °C, 17 h.

The optimized conditions used two readily available 467 nm LED Kessil lamps, Cs_2_CO_3_ as base, and DMF as reaction solvent (see the Supporting Information page S13 for a detailed description of the reaction setup). Iridium photocatalyst [Ir{dF(CF_3_)ppy}_2_(dtbbpy)]PF_6_ (**[Ir]**; see Scheme 2) provided appropriate redox potentials to oxidize the carboxylate and reduce the Giese adduct. Other photocatalysts such as [Ru(bpy)_3_](PF_6_)_2_ or (Mes-Acr)(ClO_4_) formed little (<5%) or no product (entries 2–3). Only slight reductions in yield were observed when reducing photocatalyst loading to 0.5 mol % (entry 4), performing the reaction at lower temperatures (28 °C, fan controlled; entry 5), in different solvents (MeCN, 1,4-dioxane; entries 6–7), lower concentration (entry 8), with variable amounts of ethyl acrylate (entries 9–10) or with an organic base (DBU; entry 11). Control experiments demonstrated the requirement for photocatalyst, light, and base (entries 12–14).

**Figure 2:**
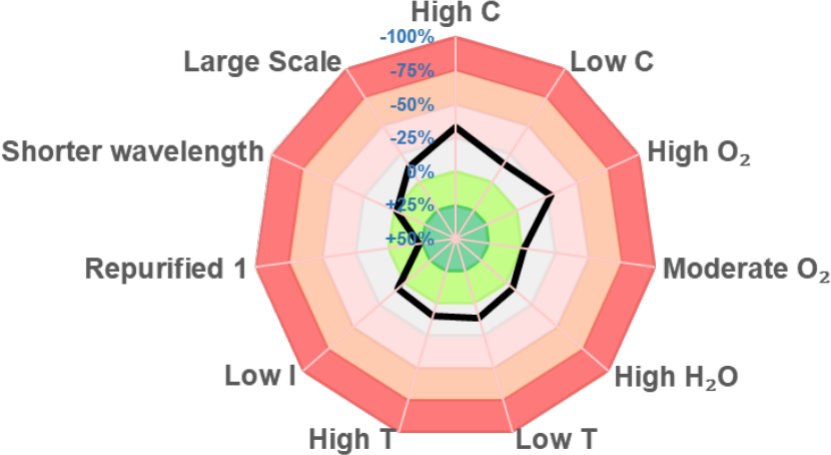
Assessment of sensitivity of the reaction of oxetane acid **1** with ethyl acrylate. Numbers indicate the deviation in percentage yield on alteration of selected reaction parameters.

We examined deviations in reaction conditions that are often encountered between laboratories by using a sensitivity screen as described by Glorius (Figure 2).^31^ The reaction was shown to be broadly insensitive to small changes in the optimized conditions, thus facilitating implementation of the decarboxylative oxetane-functionalization protocol (see Supplementary Table S6 for details). Only high concentrations (0.25 M instead of 0.2 M) and high levels of oxygen (i.e., under air) were identified as potential pitfalls for the reaction, factors that can be readily controlled. Notably, given the known sensitivity of photochemical reactions to increased scale,^32^ the input of **1** could be increased to 5 times the standard scale (0.2 to 1.0 mmol) by simply increasing the size of the reaction vial with no impact on yield (58% **2a**, 153 mg). Repurification of **1** (to 99.9% pure instead of 98.9% by quantitative ^1^H NMR) further increased the yield of **2a** (67%).

With optimized and reliable conditions in hand, the scope of the reaction was probed (Scheme 2). A diverse set of novel 3,3-disubstituted oxetane products was obtained by varying both the radical acceptor and the oxetane acid precursor. Simple acrylates were successful coupling partners and generated the products in good yields (**2a**, **2b**). Vinyl ketones, sulfones, phosphonates, and nitriles could also be employed, albeit in lower yields (**2c**–**2f**). Medicinally interesting amide derivatives were synthesized in moderate yields (**2g**–**2i**).

Similarly, a radical acceptor with two stabilizing groups was well tolerated using protected dehydroalanine to generate unnatural amino acid **2j**. Methacrolein was also successful, providing aldehyde **2k**. Substitution at the β-position was less well tolerated, though successful reactions were achieved with cyclopentenone and dimethyl maleate, affording **2l** and **2m** in 25% and 54% yield, respectively. Styrenes were also successful coupling partners (**2n**–**2t**). Unactivated, electron-neutral styrene yielded oxetane **2n** in 35% yield. Reactivity increased with more electron-poor acceptors whereby *p*-bromophenyl (73%) and isobenzofuranone (60%) functionalities were incorporated efficiently (**2o**, **2p**). The tolerance of an aryl bromide group is noteworthy, especially since it serves as a synthetic handle for downstream diversifications. Importantly, pyridines, the most prevalent aromatic N-heterocycles in bioactive compounds,^33^ could be readily introduced while tolerating functionalities such as a secondary amide (**2q**–**2t**). Increasing the equivalents of vinylpyridine acetamide to 3.0 equiv or using oxetane acid **1** in excess did not improve the yield of **2s**.

Next, variation in the oxetane acids was investigated (**5**–**12**). A benzyl protecting group, which can be labile to photoredox conditions,^34^ was well tolerated (**5q**; 62%). An electron-withdrawing triflate group could also be incorporated in low yields (**6q**; see the Supporting Information page S36 for further discussion). TIPS-protected phenol was deprotected under the reaction conditions to give free phenol **7a**. Importantly, and in contrast to our previous strategies generating oxetane carbocations,^19^ electron-neutral phenyl oxetane acid was a successful substrate and provided 3,3-disubstituted oxetane **8a** in 49% yield. A comparison of the reaction profiles for the formation of **2a** and **8a** indicated the rate of reaction was faster to form **2a**, which could be correlated with the lower oxidation potential of the carboxylate from **1** (see Supporting Tables S14–S15 and Figure S11). Electron-neutral oxetane analogs of ibuprofen and flurbiprofen gave oxetanes **9a** and **10a**, and unsubstituted biphenyl oxetane acid gave an oxetane-containing ester analog of fenbufen (**11b**). The medicinally important indole group was also incorporated (**12a**).

Pleasingly, 3-arylazetidine radicals could also be formed under the reaction conditions and a range of 3,3-disubstituted azetidines were synthesized in comparable yields to their oxetane analogs (**13a**, **b**, **m**, **n**, **p**, **q**, **t**). Azetidine pyridines **13q** and **13t** showed a boost in yield compared to the oxetanes. Further information on the examples in Scheme 2 that showed significant amounts of side products and other radical acceptors that did not yield the desired Giese product can be found in Supporting Schemes S5 and S6, respectively.

The Cbz group could be readily removed from **13a** by hydrogenolysis to generate a free N–H azetidine (**14**; 76% yield). Ester hydrolysis from the functionalized oxetane products under basic conditions using LiOH gave carboxylic acid **15** in 99% yield.^35^ This is the formal bis-homologation product of oxetane **1**, which was further characterized by X-ray crystallography (see Supporting Figure S33). Interestingly, deprotection of the *t*-Bu ester **11b** using trifluoroacetic acid prompted ring-opening of the oxetane ring to form tetrahydropyranone **16** in 75% yield due to the internal nucleophile.

**Figure 3:**
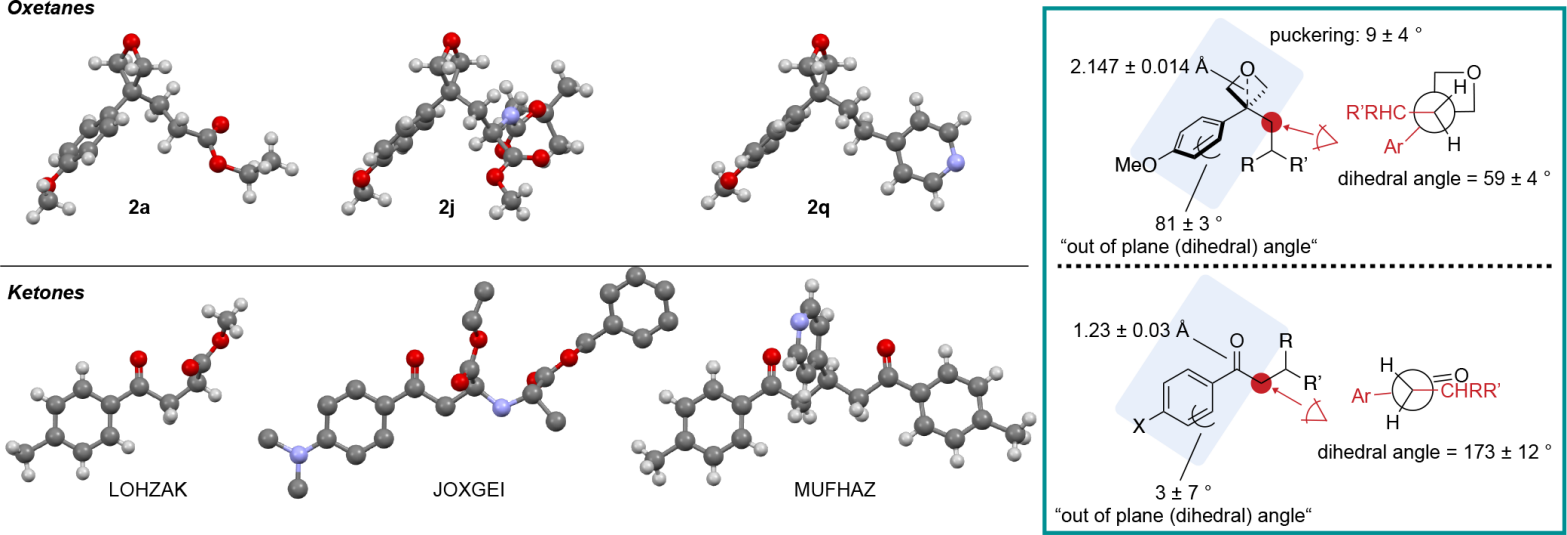
X-ray structures of 3-aryl-3-alkyl oxetanes. Interatomic distances and torsion (dihedral) angles are given as the mean average of the three X-ray structures displayed with the error corresponding to the 95% confidence interval (for the oxetanes and ketones respectively). For the atoms used in the measurement of dihedral angles, see Supporting Figures S27–S32. The ketone structures were accessed under the CCDC identifiers “LOHZAK”,^36^ “JOXGEI”,^37^ and “MUFHAZ”.^38^

3,3-Disubstitued oxetanes **2a**, **2j**, and **2q** were further characterized by X-ray crystallography and compared to known analogous phenone structures, which displayed interesting differences in conformation (Figure 3; also see Supporting Information pp S75–S81). The 3,3-disubstituted oxetane derivatives adopt a conformation with increased three-dimensional nature. Most notably the aromatic ring on the oxetane is almost orthogonal to the pseudocarbonyl plane (dihedral angle between O_oxetane_–Cq_oxetane_–Cq_aromatic_–CH_aromatic_), while, in the ketone, the aromatic ring is aligned with the plane as to maximize favorable π-conjugation. Furthermore, the increased steric bulk of the oxetane group together with the decreased steric requirement of the “twisted” aromatic ring induce a switch in the preferred conformation of the CH_2_CHRR′ chain, which now lies on the side of the aromatic instead of the carbonyl (oxetane). The conformational preferences in the X-ray structures of **2a**, **2j**, and **2q** did not seem to be influenced significantly by crystal packing effects, as revealed by analysis of intermolecular interactions in **2a**, **2j**, and **2q** (Supporting Information pages S75–S81).

### Mechanistic Studies: Structure and Reactivity of Benzylic Radicals

**Scheme 3:**
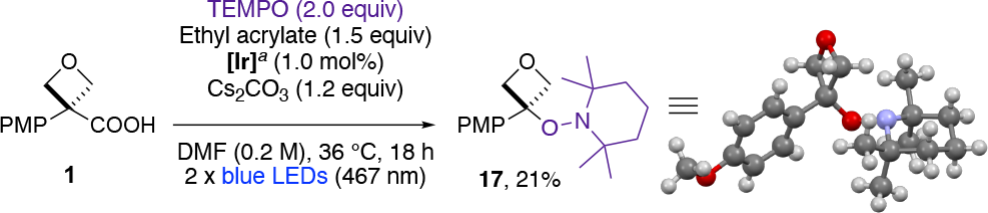
Isolation of Oxetane–TEMPO Adduct as Evidence for Oxetane Radical **[Ir]** = [Ir{dF(CF_3_)ppy}_2_(dtbbpy)]PF_6_ (see Scheme 2).

Based on the precedent of decarboxylative photoredox reactions,^26a−26d,29^ we propose an oxetane/azetidine radical intermediate formed by oxidation of the carboxylate anion with the excited form of the photocatalyst followed by decarboxylation (see Figure 5 for the full mechanistic picture). Reacting oxetane **1** with ethyl acrylate in the presence of TEMPO did not form the usual product **2a**. Oxetane–TEMPO adduct **17** was instead isolated in 21% yield (after chromatography) and its structure confirmed by X-ray crystallography, supporting the proposed oxetane radical (Scheme 3).

**Scheme 4:**
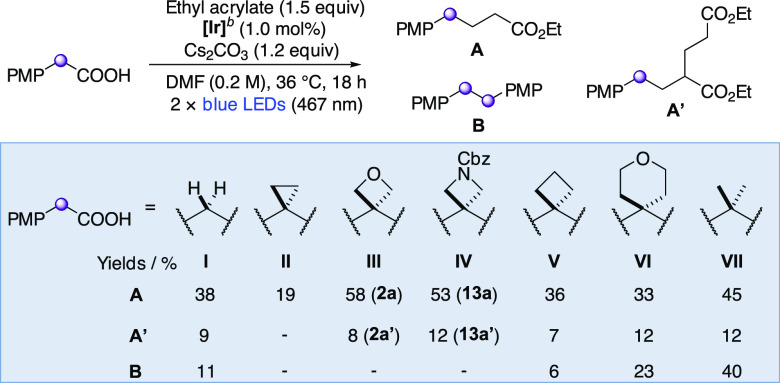
Reactivity of Tertiary Benzylic Radicals Isolated yields are reported. **[Ir]** = [Ir{dF(CF_3_)ppy}_2_(dtbbpy)]PF_6_ (see Scheme 2).

Intermolecular reactions that involve radicals at benzylic positions often lead to low yields of the desired product and increased side reactions such as homocoupling or reduction of the benzylic radical.^13,20c,21,39^ The reasons behind this, and the effect of additional substituents at the benzylic position on reactivity, are not well understood. To provide insight into the key radical-addition step, we compared the behavior of a series of aryl acetic acids, with different substitution at the benzylic center, in the reaction with ethyl acrylate (Scheme 4). Only oxetane and azetidine undergo the desired Giese reaction pathway efficiently (>50% **2a** = **III-A**, and **13a** = **IV-A**). Very little or no dimer formation was observed for the three- and four-membered rings, in contrast to methylene, *gem*-dimethyl, and tetrahydropyran linkers.^40^

### Computational Studies: Comparison of Benzylic Radicals

The significant differences in reactivity of aryl acetic acids with different benzylic linkers were further investigated computationally to provide insights into the underlying features that lead to these disparities. Since maximizing the yield of Giese product requires minimization of undesired dimerization, we sought to identify the origins of dimer formation. The relative quantities of dimer observed vary significantly depending on the identity of the benzylic substituents (Scheme 4). We isolated two factors that minimize dimer formation: (1) decreasing the stability of the benzylic radical, and (2) enhancing π-delocalization of the benzylic radical into the aromatic system.

**Figure 4:**
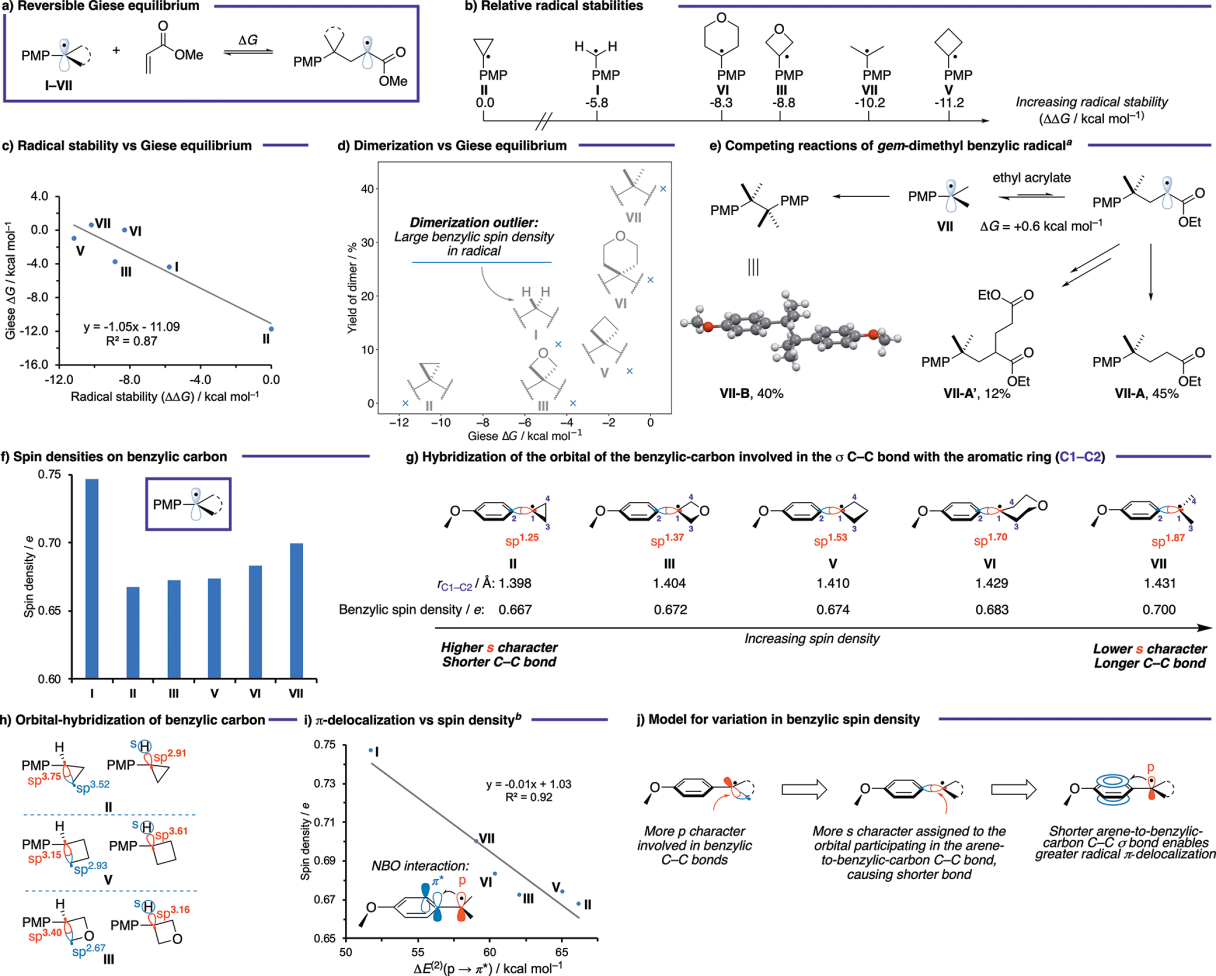
Analysis of the reactivity of benzylic radicals in a Giese-type reaction. All calculations at the CPCM^45^ (DMF)-ωB97X-D3^46^/def2-TZVP^147^//ωB97X-D3^46^/def2-SVP^147^ level. Free energies calculated at 298.15 K and 1 M standard state. Hybridizations calculated using Natural Bond Orbital (NBO) theory.^47^^*a*^Yields were experimentally determined and as depicted in Scheme 4. Δ*G* calculated using methyl acrylate (Supporting Figure S7). ^*b*^Δ*E*^(2)^(p → π*) calculated using NBO second order perturbation theory.

First, computational studies showed the Giese addition of benzylic radicals into methyl acrylate to range from moderately exergonic to slightly endergonic (Δ*G* = −11.7 to +0.6 kcal mol^–1^), indicating the possibility of reversible radical additions for some substrates (Figure 4a; see Figure 4c and Supporting Table S9 for the computed Δ*G* data of the Giese equilibrium). The relative stability of the benzylic radicals was then calculated (stabilities determined through H atom exchange equilibria with cyclopropyl radical **II**; Figure 4b and Supporting Figure S6) and found to directly influence the equilibrium position of the Giese addition, with a linear relationship (*R*^2^ = 0.87, Figure 4c). This Giese equilibrium determines the extent of dimerization: as the radical becomes less stable, the driving force for the Giese addition increases and dimerization is disfavored (Figure 4d). For example, the relative instability of cyclopropyl radical **II** causes an exergonic addition to the acrylate (Δ*G* = −11.7 kcal mol^–1^), resulting in rapid Giese quenching of this radical and therefore dimer suppression. The cyclopropane radical, however, is known to be very unstable and more prone to ring opening than its larger-ring counterparts, presumably leading to its increased degradation and consequently, a low yield of Giese product **II-A** (19%) and no observable dimer **II-B**.^41^ Conversely, the *gem*-dimethyl benzylic radical **VII** is stabilized to the extent that the addition to the acrylate becomes endergonic (Δ*G* = +0.6 kcal mol^–1^) and is therefore reversible, allowing a buildup of this radical in the reaction and increasing the likelihood of dimerization (Figure 4e). The energy barriers for the Giese addition were calculated to be relatively low, Δ*G*^‡^ = 15.3–19.5 kcal mol^–1^. For substrates with only a small, or nonexistent, driving force (e.g., cyclobutyl, THP, and *gem*-dimethyl), the reverse process will have similar forward and backward reaction barriers, leading to balanced concentrations of benzylic- and Giese adduct radicals. However, for systems with a larger driving force (e.g., oxetane, methylene and cyclopropyl), the Giese process will be irreversible resulting in low concentrations of benzylic radicals.

The unsubstituted benzylic radical (**I**) is an outlier in this stability/dimerization trend in that dimer is observed (11%), despite the equilibrium lying toward the Giese adduct (Δ*G* = −4.4 kcal mol^–1^). To explain this phenomenon, we noted that the spin density is much more localized at the benzylic position than that of the rest of the radicals under study (ρ_s_ = 0.75, Figure 4f). More localized benzylic radicals may incur a lower penalty to dimerization due to nonperfect synchronization,^42^ making unsubstituted radical **I** the most susceptible to dimerization of the series.^43^ On the other hand, the lower spin density of the cyclopropane radical **II** (ρ_s_ = 0.67) increases the barrier to dimerization relative to the Giese addition.

We propose that the extent of radical delocalization is controlled by the hybridization of the orbital of the benzylic carbon involved in the arene-to-benzylic-carbon σ bond (C1–C2, Figure 4g), which in turn is determined by the hybridization of the orbitals involved in the C–C σ bonds of the additional benzylic substituents (C1–C3 and C1–C4, Figure 4g and h). Substrates in which the orbitals making these benzylic C–C bonds *require* enhanced p-character due to the small internal angles of small rings, *e.g*. cyclopropane **II** (sp^3.75^; Figure 4h), must assign greater s-character to the orbital of C1 involved in the C–C σ bond with the arene (sp^1.25^; Figure 4g). This is expressed in a shorter C1–C2 bond, increased radical delocalization, and reduced benzylic spin density (Figure 4i; see Figure 4j for our proposed model for variation in spin density). We propose that delocalization is enhanced in oxetane **III** compared to cyclobutane **V** due to Bent’s rule:^44^ the electronegative oxetane oxygen atom withdraws electron density from the ring C–C bonds, forcing a greater p-contribution on the orbitals forming these bonds in **III** than **V** (sp^3.40^ vs sp^3.15^, respectively, Figure 4h). As a result, the orbital of the benzylic-carbon (C1) involved in the C–C bond with the arene in **III** (C1–C2) is richer in s-character (sp^1.37^) and the bond is shorter (1.404 Å) than in **V** (sp^1.53^ and 1.410 Å, respectively), thereby increasing π-delocalization and subsequently minimizing dimerization.

Overall, dimerization will be minimized for substrates with destabilized benzylic radicals that are able to efficiently delocalize into the arene π system. For example, the Giese addition for oxetane radical **III** is exergonic (Δ*G* = −3.7 kcal mol^–1^), and the radical is sufficiently π-delocalized (ρ_s_ = 0.67) due to the high p-character demanded within the oxetane ring (*vide supra*), such that no dimer is observed and the yield of Giese product is high in the reaction with acrylates.

**Figure 5:**
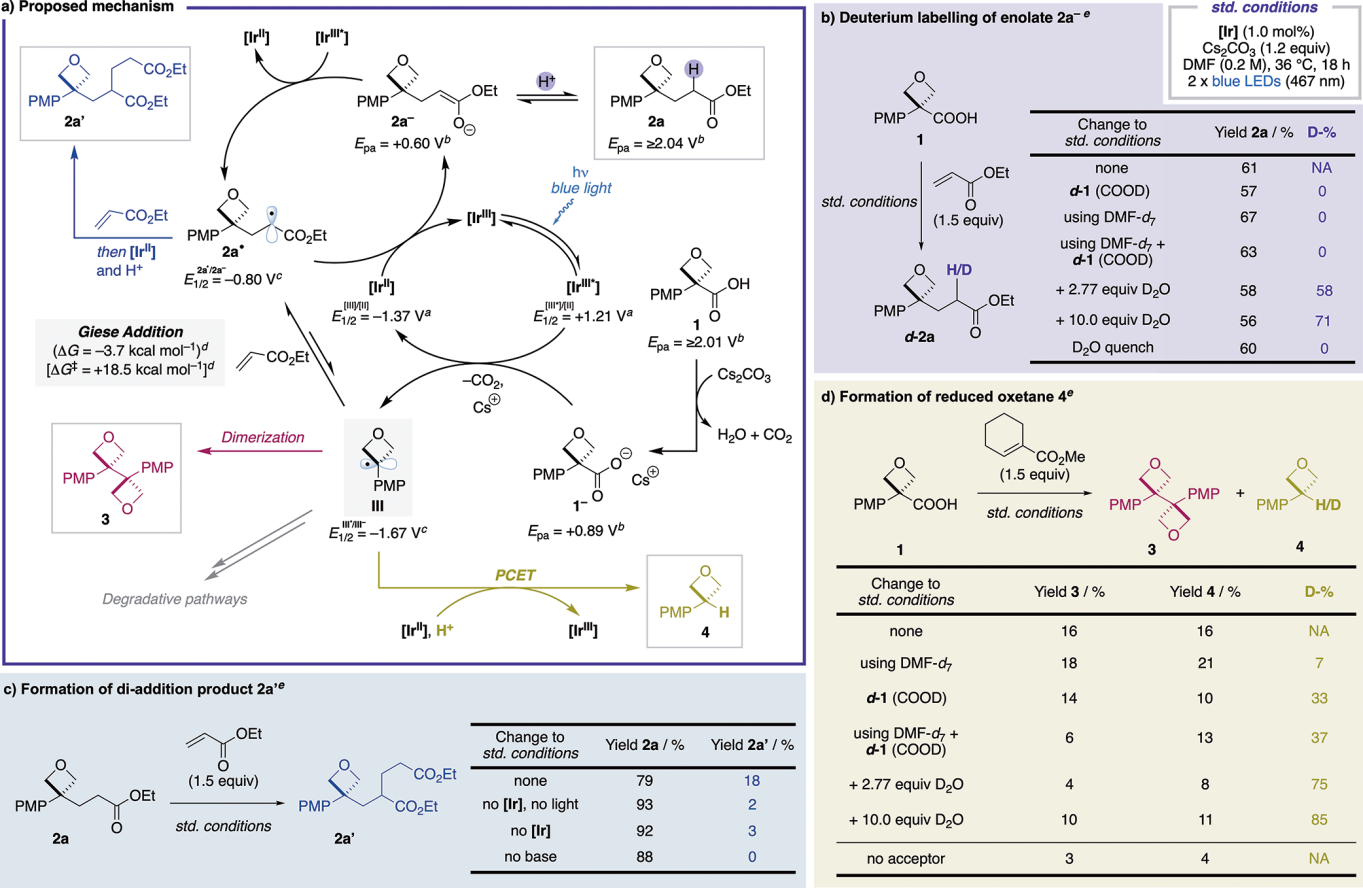
Mechanistic studies. ^*a*^Literature values vs SCE in MeCN.^49^^*b*^Oxidation potentials measured by cyclic voltammetry in a 0.1 M solution of NBu_4_ClO_4_ in MeCN at 25 °C with 100 mV s^–1^ scan rate and reported vs SCE. Anions generated *in situ* through the addition of 1 equiv NBu_4_OH (1 M in MeOH). Here, the anodic peak potentials (*E*_pa_) are reported.^50^ See the Supporting Information, page S35 for further details. ^*c*^Values calculated at the SMD^51^(DMF)-M06-2X^52^/ma-def2-TZVP^147^//ωB97X-D3^46^/def2-SVP^147^ level (298.15 K, 1 M). Value for **2a**^**•**^ calculated using the methyl ester. ^*d*^Values calculated using methyl acrylate at the CPCM^45^ (DMF)-ωB97X-D3^46^/def2-TZVP^147^//ωB97X-D3^46^/def2-SVP^147^ level (298.15 K, 1 M). ^*e*^Yields and percentage of deuteration (D-%) calculated by analysis of the ^1^H NMR spectrum of the crude mixture of the reaction using 1,3,5-trimethoxybenzene as internal standard and a 30 s relaxation delay (d1). **[Ir]** = [Ir{dF(CF_3_)ppy}_2_(dtbbpy)]PF_6_ (see Scheme 2).

Based on literature precedent^26a−26d,29^ and focused experimental (Figure 5) and computational data (Figure 4), we propose the mechanism depicted in Figure 5a. The **[Ir**^**III**^**]** catalyst is excited by visible light to **[Ir**^III*^]. Oxetane acid **1**, which cannot be oxidized directly by **[Ir**^III*^], is deprotonated by Cs_2_CO_3_ to **1**^**–**^, which is now oxidizable at +0.89 V vs SCE. **1**^**–**^ transfers an electron to **[Ir**^III*^] and is thereby oxidized to a carboxylate radical (not shown) that rapidly (*k* = 10^10^ s^–1^)^48^ decarboxylates to oxetane radical **III**.

Oxetane radical **III** then undergoes an essentially irreversible (forward reaction ca. 500 times faster than reverse reaction) and slightly exergonic Giese addition into ethyl acrylate to form adduct **2a**^**•**^, which is reduced by **[Ir**^**II**^**]** to enolate **2a**^**–**^, thereby regenerating the catalyst, **[Ir**^**III**^**]**. In a final step, **2a**^**–**^ is protonated to form the product **2a**. Deuteration studies (Figure 5b) support the protonation step, with high deuterium incorporation α to the ester with D_2_O as additive. The source of protons under the standard reaction conditions remains unclear, with no deuterium incorporated with either *d***-1** (COOD) or DMF-*d*_7_ and no significant effect of water content on the reaction outcome (Supporting Tables S11–S12).

Diaddition product **2a′** was shown to form predominantly via the polarity mismatched radical conjugate addition of **2a**^**•**^ into a second molecule of ethyl acrylate (followed by reduction by **[Ir**^**II**^**]** and protonation) and not by a more intuitive polar conjugate addition of **2a**^**–**^ into ethyl acrylate. **2a**^**•**^ can either directly add into ethyl acrylate after its formation or be reformed at a later stage by oxidation of **2a**^**–**^ by the excited photocatalyst **[Ir**^III*^], as suggested by the oxidation potentials (Figure 5a). Importantly, it was shown that the final product **2a** is converted into **2a′** by resubmission to the reaction conditions (18%), but not in the absence of **[Ir]**, light, or base (<3%; Figure 5c). Slow depletion of **2a** with increased amounts of **2a′** was observed at long reaction times (Supporting Table S14 and Figure S11).

Oxetane radical **III** can also embark on alternative reaction pathways such as dimerization to form **3** or reduction to generate **4**. The extent of these side reactions was intriguingly dictated by the choice of radical acceptor (see Supporting Schemes S5 and S6 for full overview). Particularly, less electrophilic and/or more sterically hindered alkenes led to increased amounts of **3** and **4**. This is presumably due to an increase in Δ*G* and Δ*G*^‡^ (both more positive) of the Giese addition, shifting the equilibrium toward oxetane radical **III**. A higher concentration of oxetane radical significantly increases the rate of dimerization (directly proportional to the square of radical concentration), and the formation of reduced oxetane **4**.

Reduction of oxetane radical **III** to generate **4** was intriguing, with no explicit reductant or H atom transfer (HAT) reagent present in the reaction mixture. We investigated the formation of **4** through deuteration studies using cyclohexene methyl carboxylate as the acceptor, which had shown elevated amounts of **4** (Figure 5d). High deuterium incorporation with D_2_O as an additive suggests protons to be the source of “H” in **4**. The low deuterium incorporation with DMF-*d*_7_ rules out HAT from DMF to the oxetane radical.^53^ Reduction of the oxetane radical by **[Ir**^**II**^**]** to an oxetane-3-anion followed by protonation is unlikely based on the calculated reduction potential of the oxetane radical (*E*_1/2_^III/III^–^^ = −1.67 V vs SCE) which lies outside the reduction range of **[Ir**^**II**^**]** (Figure 5a). We hence tentatively propose reduced oxetane **4** to be generated through a concerted multisite proton-coupled electron transfer (PCET)^54^ with **[Ir**^**II**^**]** as the source of electrons.

Interestingly, only minimal amounts of **3** and **4** were observed in the absence of acceptor (Figure 5d), hinting to its involvement in the oxidation of **[Ir**^**II**^**]** back to **[Ir**^**III**^**]**. However, direct oxidation of **[Ir**^**II**^**]** by the alkene is unfeasible based on the alkenes’ reduction potentials (e.g., for cyclohexene methyl carboxylate calculated to be *E*_1/2_^M/M^•–^^ = −2.60 V vs SCE; Supporting Table S7).^55^ Thus, speculatively, we propose that the high concentration of strained oxetane radical also increases the rate of irreversible oxetane degradation pathways to generate radicals that can more easily add into the acceptor and form unidentified Giese adducts capable of oxidizing **[Ir**^**II**^**]**.^56^

## Conclusions

In summary, we report the generation of unusual tertiary benzylic strained oxetane and azetidine radicals under photoredox catalysis. We have developed a protocol for the use of 3-aryl-3-carboxylic acid oxetanes and azetidines as radical precursors which react with alkenes to form medicinally relevant alkylated 3,3-disubstituted oxetanes and azetidines with previously inaccessible substitution patterns. The reaction is reproducible, easy to set up, and insensitive to common deviations from the conditions. The products could be further transformed to reveal free NH azetidine functionality, as well as a free “bis-homologated” oxetane carboxylic acid or a tetrahydropyranone heterocycle after oxetane ring-opening, depending on the conditions used for ester removal. An experimental comparison of the reactivity of different benzylic radicals revealed only oxetane and azetidine substrates to favor a productive Giese reaction pathway with the other benzylic linkers showing significantly lower yields and/or increased amounts of dimer side products. A computational investigation revealed the Giese addition of benzylic radicals into acrylate acceptors to be reversible in some cases, with less stable radicals shifting the equilibrium toward the coupled product. Furthermore, reduced spin density on the benzylic carbon was found to minimize formation of the dimer side product. Oxetane radicals, and by analogy azetidine radicals, are the only species that lie in the sweet spot of undergoing little degradation of the benzylic radical, while showing an exergonic Giese addition and minimal dimer formation.

Experimental and computational studies are presented to explain the differing reaction outcome with certain acceptors, and to suggest the mechanism for the formation of side products. We envisage this protocol will encourage the use of 3-aryl-3-carboxylic acid oxetanes and azetidines as convenient radical precursors in medicinal chemistry and expand the medicinal chemist’s toolbox for the incorporation of four-membered rings into drug-like compounds. The computational and experimental mechanistic investigations presented herein improve the general understanding of photoredox catalyzed reactions, particularly in Giese-type transformations and of strained ring and/or benzylic substrates. The insights from these fundamental studies will aid the development of further methodologies that involve such substrates and provide synthetic chemists with important factors to be considered during the optimization of such reactions.

## Data Availability

The data underlying this study are available in the published article and its Supporting Information. Raw and processed characterization data for all novel compounds, raw and processed data of CV measurements and Cartesian coordinates from computed structures can be found at the Imperial College London Research Data Repository: 10.14469/hpc/10668.

## Supporting Information Available

X-ray crystallographic data; detailed optimization and sensitivity tables; further discussion of mechanism and scope; cyclic voltammetry (CV) measurements; experimental procedures, detailed description of setup, characterization data and copies of ^1^H, ^13^C, ^19^F, and ^31^P NMR spectra (PDF)

## Notes

A version of this manuscript was deposited on the preprint repository ChemRxiv.^57^
