# Supplementary material for: Visible Light Photoredox-Catalyzed Decarboxylative Alkylation of 3-Aryl-Oxetanes and Azetidines via Benzylic Tertiary Radicals and Implications of Benzylic Radical Stability
Source: J Org Chem. 2023 Mar 3;88(10):6476–88. doi: 10.1021/acs.joc.3c00083 (PMC10204094; doi:10.1021/acs.joc.3c00083)
Supplement: Supplementary file 1 — jo3c00083_si_001.pdf [file jo3c00083_si_001.pdf]

## SUPPORTING INFORMATION

# Visible Light Photoredox-Catalyzed Decarboxylative Alkylation of 3-Aryl-Oxetanes and Azetidines via Benzylic Tertiary Radicals and Implications of Benzylic Radical Stability

**Maryne, A. J. Dubois,[a,†] Juan J. Rojas,[a,†] Alistair J. Sterling,[b,†] Hannah C. Broderick,[a] Milo, A. Smith,[a] Andrew, J. P. White,[a] Philip W. Miller,[a] Chulho Choi,[c] James, J. Mousseau,[c] Fernanda Duarte,*,[b] and James, A. Bull*,[a]**

[a] Department of Chemistry, Imperial College London, Molecular Sciences Research Hub, White City Campus, Wood Lane, London W12 0BZ, UK.
[b] Department of Chemistry, Chemistry Research Laboratory, University of Oxford, Oxford, UK.
[c] Pfizer Global Research and Development, 445 Eastern Point Rd., Groton, CT 06340, USA.
[†] These authors contributed equally.

*E-mail: j.bull@imperial.ac.uk
*E-mail: fernanda.duartegonzalez@chem.ox.ac.uk

General Experimental Conditions ..... S3
Structures of Additional Compounds in SI ..... S5
Other Oxetane Radical Precursors ..... S6
Decarboxylative Alkylation of 3-Aryl-Oxetanes ..... S7–S14
Further Optimization Reactions ..... S7
Reproducibility of Optimal Conditions ..... S10
General Procedure A and B ..... S11
Photoredox Apparatus and Set-up ..... S13
Sensitivity Screen ..... S14
Synthesis of Oxetane and Azetidine Carboxylic Acid Starting Materials ..... S15
Examples of Scope with Formation of More than One Product ..... S17
Low Yielding and Unsuccessful Radical Acceptors ..... S18
Mechanistic Investigations ..... S19–S45
Computational Details ..... S19–S26
Methods ..... S19
Calculation of Reduction Potential ..... S20
Radical Stability ..... S22
Giese Addition ..... S24
Benzylic Spin Density ..... S26
Deuteration Studies ..... S27–S32
Reaction of Oxetane **1** with Ethyl Acrylate ..... S28
Formation of Reduced Oxetane **4** ..... S31
Reaction Profiles for Formation of **2a** and **8a** ..... S33
Cyclic Voltammetry Experiments ..... S35
Experimental Details and Characterization Data ..... S46–S74
PMP Oxetane Dimer **3** and Reduced Oxetane **4** ..... S46
3-Aryl-3-Alkyl-Oxetanes (**2a–2t**, **7a–12a**, **S5**, **S6**) ..... S46
3-Aryl-3-Alkyl-Azetidines (**13a**, **13b**, **13m**, **13n**, **13p**, **13q**, **13t**, **S9**) ..... S59
Oxetane and Azetidine Derivatization Products (**14–16**) ..... S64
Oxetane–Tempo Adduct **17** ..... S66
PMP Acetic Acids with different benzylic substituents (**I–VII**) ..... S67–S73
Methylene Products (**I-A**, **I-A'**, **I-B**) ..... S67
Cyclopropane Products (**II-A**) ..... S68
Oxetane Products (**III-A**, **III-A'**) ..... S69
Azetidine Products (**IV-A**, **IV-A'**) ..... S70
Cyclobutane Products (**V-A**, **V-A'**, **V-B**) ..... S71
Tetrahydropyran Products (**VI-A**, **VI-A'**, **VI-B**) ..... S72
*gem*-Dimethyl Products (**VII-A**, **VII-A'**, **VII-B**) ..... S73
4CzIPN ..... S74
X-Ray Crystallography Details ..... S75–S83
3-Aryl-3-Alkyl-Oxetanes **2a**, **2j**, **2q**, **15** ..... S76
Oxetane–TEMPO Adduct **17** ..... S84
*gem*-Dimethyl Dimer **VII-B** ..... S85
$^{1}H$, $^{13}C$, $^{19}F$ and $^{31}P$ NMR Spectra of Selected Compounds ..... S86–S153
References ..... S154

**General Experimental Considerations**

All non-aqueous reactions were carried out under an inert atmosphere (argon) with flame-dried glassware, using standard techniques, unless specified. Anhydrous solvents were obtained by filtration through drying columns (DMF, EtOH, MeCN, $CH_2Cl_2$) or used as supplied (1,4-dioxane).

Reactions that required thermal activation were heated using a water bath (for temperatures up to 25 °C) or a silicon oil bath (for temperatures >25 °C).

Flash column chromatography was performed using 230–400 mesh silica, or 40–230 μm basic alumina grade IV with the indicated solvent system according to standard techniques. Analytical thin-layer chromatography (TLC) was performed on precoated glass-backed silica gel plates. Visualization of the developed chromatogram was performed by UV absorbance (254 nm) and stained with aqueous potassium permanganate solution, phosphomolybdic acid solution, *para*-anisaldehyde solution or ninhydrin solution in ethanol.

Infrared spectra ($\nu_{max}$, FTIR ATR) were obtained using an Agilent Technologies Cary 630 FTIR or a Perkin Elmer Spectrum 100 FTIR Spectrometer and recorded in reciprocal centimeters ($cm^{-1}$) (br = broad, w = weak, st = stretch, as = asymmetric, sy = symmetric). Only significantly strong and clearly assignable signals diagnostic for major functional groups are reported.

Nuclear magnetic resonance spectra were recorded on 400 or 500 MHz spectrometers. The frequency used to record the NMR spectra is given in each assignment and spectrum ($^{1}H$ NMR at 400 or 500 MHz; $^{13}C$ NMR at 101 MHz or 126 MHz; $^{19}F$ NMR at 377 MHz; $^{31}P$ NMR at 162 MHz). Chemical shifts for $^{1}H$ NMR spectra are recorded in parts per million (ppm) from tetramethylsilane with the residual protic solvent resonance as the internal standard ($CHCl_3$: $\delta$ = 7.27 ppm). Data are reported as follows: chemical shift (multiplicity [s = singlet, d = doublet, t = triplet, q = quartet, hept = heptet, m = multiplet, br s = broad singlet, br d = broad doublet, br t = broad triplet and br m = broad multiplet], coupling constant (in Hz), integration of equivalent nuclei and assignment). $^{13}C$ NMR spectra were recorded with complete proton decoupling, indicated as $^{13}C\{^{1}H\}$. Chemical shifts are reported in parts per million (ppm) from tetramethylsilane with the solvent resonance as the internal standard ($^{13}CDCl_3$: $\delta$ = 77.0 ppm). *J* values are reported in Hz. $^{19}F$ and $^{31}P$ NMR spectra were recorded with or without complete proton decoupling. Decoupling is indicated as $^{19}F\{^{1}H\}$ and $^{31}P\{^{1}H\}$ and where relevant this is stated in each assignment. $^{19}F$ and $^{31}P$ NMR spectra are indirectly referenced to $CFCl_3$ and $H_3PO_4$ automatically by direct measurement of the absolute frequency of the deuterium lock signal by the spectrometer hardware.

Assignments of $^{1}H$ and $^{13}C$ spectra were based upon the analysis of $\delta$ and *J* values, by analogy of previous examples, as well as DEPT, COSY, HSQC and HMBC experiments where appropriate. Selected 2D spectra are included in this document (pages S86–S153). All raw and processed 2D spectra can be found at https://doi.org/10.14469/hpc/10668.

For clarity NMR spectra are displayed as follows unless this would obscure signals: $^{1}H$ NMR spectra are displayed between 10.0 ppm and 0.0 ppm; $^{13}C$ NMR spectra are displayed between 210 ppm and 0 ppm; $^{19}F$ and $^{31}P$ NMR spectra are displayed for the full sweep width as acquired.

Melting points were recorded using an Optimelt MPA100 apparatus and are uncorrected.

High resolution mass spectrometry (HRMS) analyses were performed through the Imperial College mass spectrometry service or by NovaBioAssays, using an electrospray ion source (ESI), nanospray ionization (NSI), electron impact ionization (EI) or atmospheric pressure chemical ionization (APCI) using an atmospheric solids analysis probe (ASAP). ESI was performed using either: a Waters LCT Premier (ES-ToF) equipped with an ESI source operated in positive or negative ion mode; or a Waters UPLC with TUV and Thermo Q Exactive equipped with ESI source operated in positive mode. APCI was performed using a Thermo Scientific Q-Extractive/Dionex Ultimate 3000 using an ASAP to insert samples into the APCI source operated in positive or negative mode. The sample was introduced at ambient temperature and the temperature increased until the sample vaporized.

The software used was either MassLynx 4.1 or Bruker Daltonics DataAnalysis 4.0. Please note: The MassLynx 4.1 software, used at the Imperial College mass spectrometry service, does not account for the electron and all the calibrations/references are calculated accordingly, i.e. $[M+H]^+$ is detected and the mass is calibrated to output [M+H]. In the cases where this software is used, we report the HRMS as [M+H].

**Reagents**: Where the synthesis of a reagent is not stated, the reagent was commercially available. Commercial reagents were used as supplied, or purified by standard techniques where necessary. Catalysts were acquired as described below and stored in a desiccator.

- $[Ir\{dF(CF_3)ppy\}_2(dtbbpy)]PF_6$ (**[Ir]**) was purchased from Sigma-Aldrich (CAS: 870987-63-6, product code: 747793-100MG, purity not given) or Strem Chemicals (CAS: 870987-63-6, product code: 77-0425, purity: 99%). Used without further purification.
- $[Ru(bpy)_3](PF_6)_2$ was purchased from Sigma-Aldrich (CAS: 60804-74-2, product code: 754730-1G, purity: 97%). Used without further purification.
- (Mes-Acr)($ClO_4$) was purchased from Sigma-Aldrich (CAS: 674783-97-2, product code: 747610-100MG, purity: 95%). Used without further purification.
- $[Ru(bpz)_3](PF_6)_2$ was purchased from Sigma-Aldrich (CAS: 80907-56-8, product code: 747777-50MG, purity: 95%). Used without further purification.
- 4CzIPN was synthesized according to a literature procedure (see page S74).[1] Purified by recrystallization from hot $CHCl_3$ and hexane.
- Alkenes were commercially available or obtained from the Pfizer in-house library. Liquid alkenes were distilled in the dark under vacuum and stored in a round bottom flask sealed with a septum, under argon and in the dark at –20 °C.
- $NBu_4OH$ (1 M in MeOH) was purchased from Sigma-Aldrich (CAS: 2052-49-5, product code: 230189-100ML).
- $NBu_4ClO_4$ was purchased from TCI (CAS: 1923-70-2, product code: T0836-25G, purity ≥ 98%).
- Basic alumina grade IV for flash column chromatography was prepared from commercial basic alumina Brockmann I from Acros Organics (CAS: 1344-28-1, product code: 189990010) by adding 10% of water. For example: 10 mL of water were added to 100 g of basic alumina and shaken vigorously for 5 min until the mixture was powdery and free of lumps. Caution: basic alumina becomes warm upon mixture with water.

**Structures of Additional Compounds in SI**

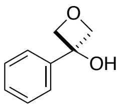

**S1**

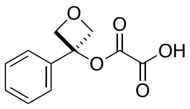

**S2**

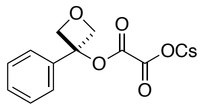

**S3**

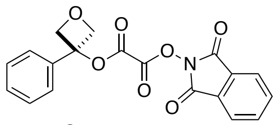

**S4**

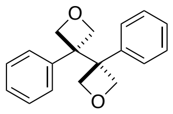

**S5**

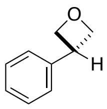

**S6**

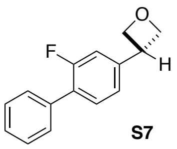

**S7**

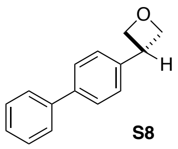

**S8**

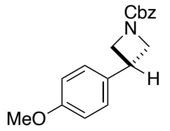

**S9**

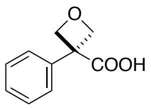

**S10**

### Other Oxetane Radical Precursors

Attempts to brominate or chlorinate phenyl oxetanol **S1** to generate 3-aryl-3-halide-oxetanes as radical precursors were unsuccessful. Complete degradation of the oxetane ring was observed both using $CBr_4$ in an Appel reaction or with Ghosez's reagent (Scheme S1). Oxetanes with potential leaving groups in the 3-position are known to be prone to elimination processes that lead to unstable oxetene species.

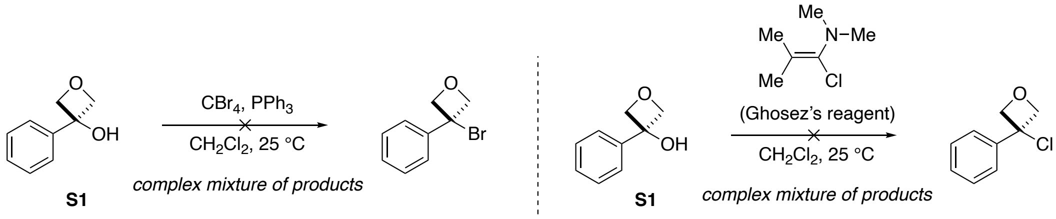


**Scheme S1** Unsuccessful attempts to synthesize 3-aryl-oxetane-3-halides.

Oxetane oxalates **S2–S4** were found to suffer from instability issues which either impeded their purification or led to hydrolysis under photoredox conditions[2] (Scheme S2).

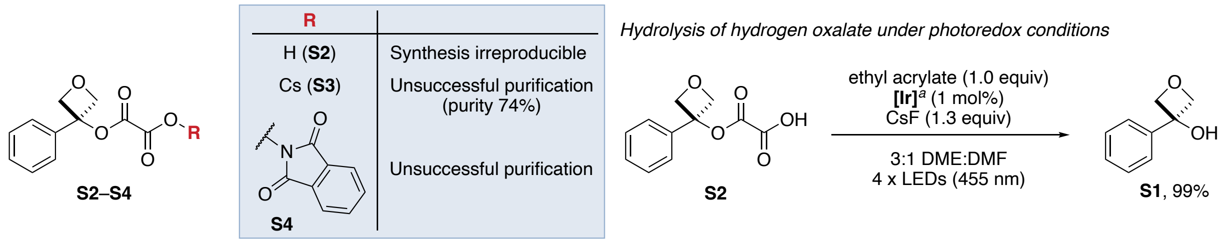


**Scheme S2** Instability of oxetane oxalates. [a] **[Ir]** = $[Ir\{dF(CF_3)ppy\}_2(dtbbpy)]PF_6$.

Cesium oxalate salts have been proposed to be more stable than the corresponding protonated- and *N*-phthalimidoyl oxalate species.[2] The 74%-pure oxetane Cs-oxalate **S3** was hence submitted to a screening of typical photoredox conditions for Giese couplings with ethyl acrylate (Table S1). Even though small amounts of coupled product **8a** were observed under some of the conditions (Entries 1–3), the Giese reaction using oxetane carboxylic acid **1** was far more efficient and robust (see Table 1 and Tables S2–S6). Instability of the Cs-oxalate under the reaction conditions and/or the presence of the oxalate impurity are potential reasons for the low yield observed.

**Table S1** Screen of conditions for the decarboxylative alkylation of oxetane Cs-oxalate **S3** with ethyl acrylate. Reactions were carried out on a 0.15 mmol scale (oxalate).

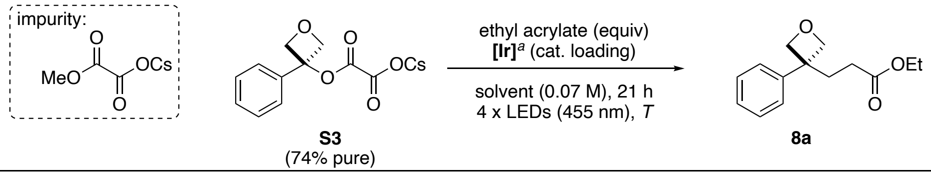


| Entry | cat. loading / mol% | equiv acrylate | solvent | *T* / °C | Yield 8a / %[b] |
|---|---|---|---|---|---|
| 1 | 1 | 1 | 3:1 DME/DMF[c] | 45–50 | 12 |
| 2 | 1 | 2 | 3:1 DME/DMF[c] | 45–50 | 19 |
| 3 | 2.5 | 1 | 3:1 DME/DMF[c] | 45–50 | 9 |
| 4 | 1 | 1 | 3:1 DME/DMF[c] | 25–30[d] | 1 |
| 5 | 1 | 1 | MeCN | 45–50 | 1 |
| 6 | 1 | 3 | 9:1 DMF/$H_2O$ | 45–50 | 0 |
| 7 | 1 | 3 | 9:1 acetone/MeOH | 45–50 | 0 |

[a] [Ir] = $[Ir\{dF(CF_3)ppy\}_2(dtbbpy)]PF_6$. [b] Yields calculated by analysis of the $^1$H NMR spectrum of the crude mixture of the reaction using 1,3,5-trimethoxybenzene as internal standard. [c] +10 equiv $H_2O$. [d] Using a fan.

**Decarboxylative Alkylation of 3-Aryl-Oxetanes**

Further Optimization Reactions

In addition to the optimization study depicted in Table 1, longer reaction times and different amounts of reagents were tested (Table S2). The yield remained unaffected at longer reaction times (Entry 2) and interestingly, sub-stochiometric amounts of base were still sufficient to promote the reaction (Entry 3; note there is no reaction in the absence of base, see Table 1). The equivalents of acrylate did not have a significant impact on the yield, thus allowing the use of radical acceptors as limiting reagent with an excess of oxetane acid (Entries 4–6; also see Table 1). Performing the reaction in a DMF:$H_2O$ mixture led to a sharp decrease in yield (Entry 7).

**Table S2** Further optimization reactions for the decarboxylative alkylation of oxetane acid **1** with ethyl acrylate. Reactions were carried out on a 0.2 mmol scale (oxetane acid) using **General Conditions A** with any change noted.

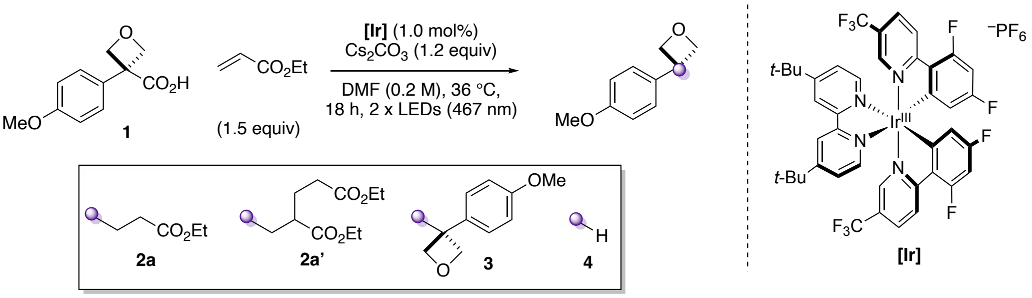


| Entry | Change from standard conditions | Yield / %[a] | | | |
|---|---|---|---|---|---|
| | | 2a | 2a' | 3 | 4 |
| 1[b] | none | 61 (58) | 8 (8) | 1 | 1 |
| 2 | 40 h reaction time | 60 | 8 | 1 | 1 |
| 3 | 0.5 equiv $Cs_2CO_3$ | 46 | 7 | 2 | 2 |
| 4 | 1.3 equiv acrylate | 61 | 7 | 1 | 1 |
| 5 | 1.0 equiv acrylate | 48 | 4 | 1 | 1 |
| 6 | 0.8 equiv acrylate | 58[c] | 6[c] | 5 | 1 |
| 7 | Using DMF:$H_2O$ 2:1 as solvent | 11 | 0 | 0 | 0 |

[a] Yields calculated by analysis of the $^1H$ NMR spectrum of the crude mixture of the reaction using 1,3,5-trimethoxybenzene as internal standard and a 30 s relaxation delay (d1).[3] Isolated yields in parentheses. [b] Reported yields are an average of 6 experiments, isolated yields of a single run are in parentheses. [c] Yields vs ethyl acrylate.

Further, other solvents and inorganic bases, including $K_2HPO_4$ as originally used by MacMillan,[4] led to significantly lower yields than under the standard conditions (Table S3).

**Table S3** Further optimization reactions for the decarboxylative alkylation of oxetane acid **1** with ethyl acrylate varying solvent and base. Reactions were carried out on a 0.2 mmol scale (oxetane acid) using **General Conditions A** with the noted changes.

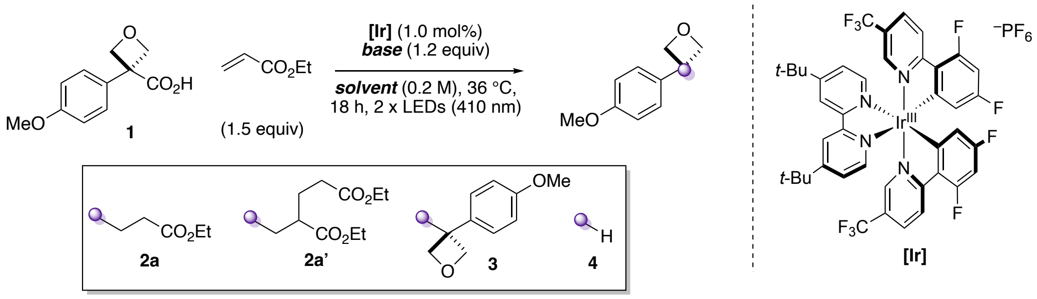


| Entry | Solvent | Base | Yield / %[a] | | | |
|---|---|---|---|---|---|---|
| | | | 2a | 2a' | 3 | 4 |
| 1 | MeOH | $Cs_2CO_3$ | 0 | ND | 0 | traces |
| 2 | Acetone | $Cs_2CO_3$ | 37 | ND | 15 | traces |
| 3 | EtOAc | $Cs_2CO_3$ | 12 | ND | 5 | 6 |
| 4 | DMF | $K_2CO_3$ | 48 | ND | 6 | 7 |
| 5 | DMF | $Na_2CO_3$ | 41 | ND | 8 | 7 |
| 6 | DMF | $K_2HPO_4$ | 43 | ND | 11 | 14 |

[a] Yields calculated by analysis of the $^1H$ NMR spectrum of the crude mixture of the reaction using 1,3,5-trimethoxybenzene as internal standard a standard 1 s relaxation delay (d1).

Ph-oxetane acid **S10** showed reduced yields of Giese product and also a higher oxidation potential (measured by cyclic voltammetry, see Table S15) compared to PMP-oxetane acid **1**. Photocatalysts more oxidizing than the standard **[Ir]** were hence tested for the synthesis of **8a** (and also **2a**) but no increase in yield was observed (Table S4). $[Ru(bpz)_3](PF_6)_2$ showed no reaction (Entry 2 and 6) and 4CzIPN formed **2a** and **8a** in 60% and 31% yield, respectively (Entry 3 and 8). Although $[Ru(bpz)_3](PF_6)_2$ is more oxidizing than **[Ir]**, its reduced form, $[Ru^I]$, does not seem to be reducing enough to generate the product (Table S4), highlighting the requirement of the reaction for a specific oxidation/reduction window (see Figure 5 for the full proposed mechanistic picture). Interestingly, using 4CzIPN and LEDs with a wavelength of 427 nm, an increased amount of dimer **3** and reduced oxetane **4** were observed compared to the reaction with **[Ir]** (Entry 4 vs 1).

**Table S4** Decarboxylative alkylation with ethyl acrylate using more oxidizing photocatalysts. Reactions were carried out on a 0.2 mmol scale (oxetane acid) using **General Conditions A** with any change noted.

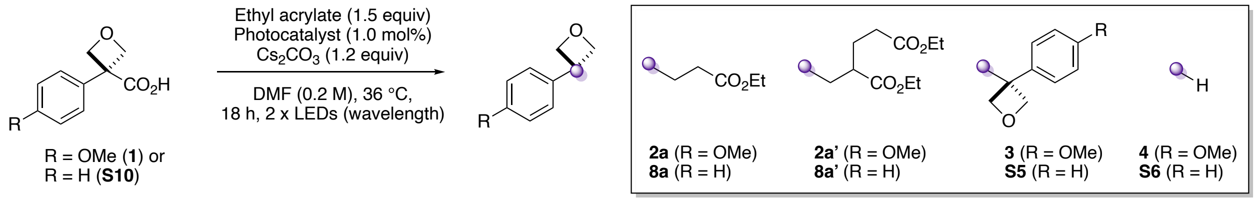


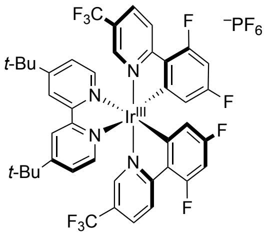


**[Ir]**
[Ir[III*]]→[Ir[II]] $E_{1/2}$ = +1.21 V vs SCE[a]
[Ir[III]]→[Ir[II]] $E_{1/2}$ = –1.37 V vs SCE[a]

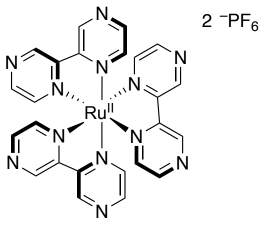


[Ru(bpz)$_3$](PF$_6$)$_2$
[Ru[II*]]→[Ru[I]] $E_{1/2}$ = +1.45 V vs SCE[b]
[Ru[II]]→[Ru[I]] $E_{1/2}$ = –0.80 V vs SCE[b]

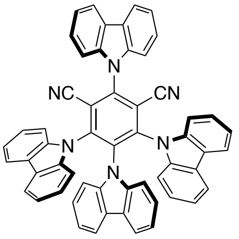


4CzIPN
[PC*]→[PC•−] $E_{1/2}$ = +1.35 V vs SCE[c]
[PC]→[PC•−] $E_{1/2}$ = –1.21 V vs SCE[c]

| Entry | R = | Photocatalyst | Wavelength / nm | Yield / %[d] | | | |
|---|---|---|---|---|---|---|---|
| | | | | 2a/8a | 2a'/8a' | 3/S5[e] | 4/S6 |
| 1[f] | OMe | **[Ir]** | 467 | 61 (58) | 8 (8) | 1 | 1 |
| 2 | OMe | [Ru(bpz)$_3$](PF$_6$)$_2$ | 467 | 0 | 0 | 0 | 0 |
| 3 | OMe | 4CzIPN | 467 | 60 | 6 | 11 | 0 |
| 4 | OMe | 4CzIPN | 427 | 57 | 4 | 21 | 7 |
| 5 | H | **[Ir]** | 467 | (37) | (8) | 0 | 0 |
| 6 | H | [Ru(bpz)$_3$](PF$_6$)$_2$ | 467 | 0 | 0 | 0 | 0 |
| 7 | H | 4CzIPN | 467 | 29 | 5 | 5 | 0[g] |
| 8 | H | 4CzIPN | 427 | 31 | 5 | 5 | 1[g] |

[a] Data from ref. 5. [b] Data from ref. 6. [c] Data from ref. 7. [d] Yields calculated by analysis of the $^1$H NMR spectrum of the crude mixture of the reaction using 1,3,5-trimethoxybenzene as internal standard and a 30 s relaxation delay (d1). Isolated yields in parentheses. [e] **S5** not isolated. Peaks with diagnostic chemical shifts comparable to oxetane dimer **3** in the $^1$H NMR spectrum of the crude mixture of the reaction were used to determine the yield. [f] Reported yields are an average of 6 experiments, isolated yields of a single run are in parentheses. [g] **S6** is expected to be very volatile.

Reproducibility of Optimal Conditions

The optimal conditions of the oxetane alkylation were repeated by three different chemists and using two different batches of oxetane carboxylic acid to ensure reproducibility (Table S5).

**Table S5** Reproducibility of the oxetane alkylation. Reactions were carried out on a 0.2 mmol scale using **General Conditions A**.

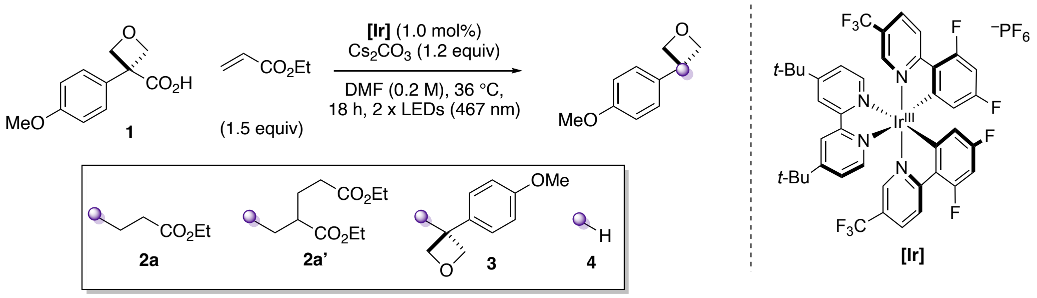


| Entry | Chemist | Batch of oxetane acid | Yield / %[a] | | | |
|---|---|---|---|---|---|---|
| | | | **2a** | **2a'** | **3** | **4** |
| 1 | 1 | 1 | 58 (57) | ND | ND | ND |
| 2 | 2 | 1 | 67 | 8 | 1 | 1 |
| 3 | 2 | 1 | 67 (58) | 11 (8) | 2 | 1 |
| 4 | 2 | 1 | 56 | 9 | 1 | 1 |
| 5 | 2 | 2 | 60 | 6 | 2 | 1 |
| 6 | 3 | 1 | 58 (53) | 7 (10) | 0 | 1 |
| | Mean average $\bar{x}$ | | 61 | 8 | 1 | 1 |

[a] Yields calculated by analysis of the $^{1}$H NMR spectrum of the crude mixture of the reaction using 1,3,5-trimethoxybenzene as internal standard and a 30 s relaxation delay (d1). Isolated yields in parentheses. ND = not determined.

The sample standard deviation *s* was then calculated for the yield of **2a** with *n* = number of reactions:

$$s = \sqrt{\frac{1}{n-1}\sum_{i=1}^{n}(x_i - \bar{x})^2} = 4.81663$$

Given the small sample size ($n \leq 100$), a 95% confidence interval (CI) for the yield of **2a** was calculated to give a better idea of the error associated with the reported yield:

$$\mathrm{CI} = t_s \frac{s}{\sqrt{n}} = 5,$$

whereby $t_s$ corresponds to the 95% fractile with $\nu$ degrees of freedom ($\nu = n - 1$). This leads to an average yield of **2a** of 61 ± 5%.

**General Procedure A**

*For liquid alkenes*

An oven-dried 4 mL vial was charged with carboxylic acid (0.20 mmol, 1.0 equiv), oven-dried $Cs_2CO_3$ (78.2 mg, 0.24 mmol, 1.2 equiv) and [Ir{dF($CF_3$)ppy}$_2$(dtbbpy)]$PF_6$ (2.2 mg, 0.002 mmol, 1.0 mol%). The vial was sealed with a screwcap equipped with a PTFE/silicon septum and anhydrous DMF (1.0 mL, 0.2 M) was added by syringe. Argon was bubbled through the mixture for 5 min and liquid alkene (0.30 mmol, 1.5 equiv) was added by syringe under argon. After sealing the cap with parafilm on top, the reaction mixture was stirred at 1000 rpm using the set-up shown in Figure S2 or S3 and irradiated with two 467 nm Kessel lamps at 36 °C (heat generated by the lamps). After 18 h the lights were switched off and the reaction mixture was transferred into a separating funnel. Distilled water (10 mL) and $Et_2O$ or EtOAc (10 mL) were added, the layers were separated and the aqueous portion was extracted with $Et_2O$ or EtOAc (2 × 10 mL). The organic extracts were combined, dried over $Na_2SO_4$, filtered and concentrated *in vacuo* using a rotatory evaporator. The crude reaction mixture was purified by flash column chromatography.

**General Procedure B**

*For solid alkenes*

An oven-dried 4 mL vial was charged with carboxylic acid (0.20 mmol, 1.0 equiv), oven-dried $Cs_2CO_3$ (78.2 mg, 0.24 mmol, 1.2 equiv), [Ir{dF($CF_3$)ppy}$_2$(dtbbpy)]$PF_6$ (2.2 mg, 0.002 mmol, 1.0 mol%) and solid alkene (0.30 mmol, 1.5 equiv). The vial was sealed with a screwcap equipped with a PTFE/silicon septum and anhydrous DMF (1.0 mL, 0.2 M) was added by syringe. Argon was then bubbled through the mixture for 5 min. After sealing the cap with parafilm on top, the reaction mixture was stirred at 1000 rpm using the set-up shown in Figure S2 or S3 and irradiated with two 467 nm Kessel lamps at 36 °C (heat generated by the lamps). After 18 h the lights were switched off and the reaction mixture was transferred into a separating funnel. Distilled water (10 mL) and $Et_2O$ or EtOAc (10 mL) were added, the layers were separated and the aqueous portion was extracted with $Et_2O$ or EtOAc (2 × 10 mL). The organic extracts were combined, dried over $Na_2SO_4$, filtered and concentrated *in vacuo* using a rotatory evaporator. The crude reaction mixture was purified by flash column chromatography.

Further remarks:

Unless otherwise stated, all purifications by flash chromatography were performed on a basic alumina (IV) stationary phase (see page S4 for preparation). Silica can also be used as stationary phase, but basic alumina (IV) was found to provide a time advantage and higher yields. Additionally, degradation on silica (but not on basic alumina (IV)) was observed for products that contained a pyridine ring.

Radical scavengers used as stabilizers in many commercial alkenes must be removed before using the alkene. This was typically done by distillation (see page S4).

If a toxic and volatile alkene is used as radical acceptor (e.g. ethyl acrylate), use a rotatory evaporator in a ventilated space during workup. Quench excess acceptor in the syringe with a dilute bleach bath (bleach:$H_2O$ = 1:1) and leave the syringe to stand in the bleach bath for 24 h before disposal.

LED lamps emit potentially high-intensity blue/visible-light which is not generally considered harmful unless the eye is exposed for a long period of time. Care should be taken not to look directly at the light. Wear transparent-orange covered safety glasses and use transparent-orange covered safety screen in front of the set up.

In productive reactions, the color of the reaction mixture typically turns from light yellow to dark brown (Figure S1)

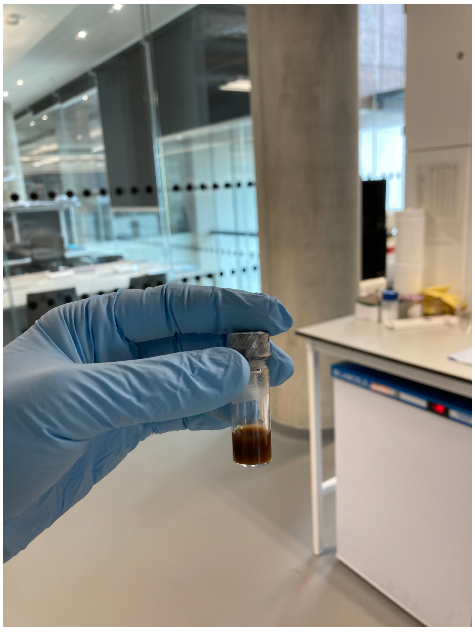

**Figure S1** Typical end color of the reaction mixture in productive reactions.

Photoredox apparatus

Photoredox reactions were performed in oven-dried 4 mL vials (product code: 854190, Sigma-Aldrich) with a thickness of 1 mm which were sealed with screwcaps with PTFE/silicon septa (product code: SU860078, Sigma-Aldrich). Reactions were run in batches of one (Figure S2) or two vials (Figure S3). The vials were attached together using two elastic bands positioned over the screw-caps. Irradiation was provided by two 40 W Kessil PR160 LED lamps with intensity dials set to maximum and placed 4 cm away from the center of the vials. When a fan (Honeywell HT900E) was used, setting III was employed, and the fan was placed 14 cm away on the front of the vials. The reactions were stirred at 1000 rpm. Without a fan, the temperature of DMF in the vial was determined to be 36 °C with an internal thermometer (2 lamps). With a fan, the temperature was 28 °C.

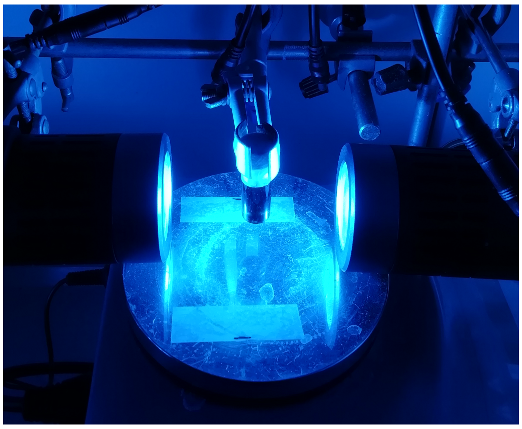

**Figure S2**: Picture of a photoredox reaction run as a single reaction.

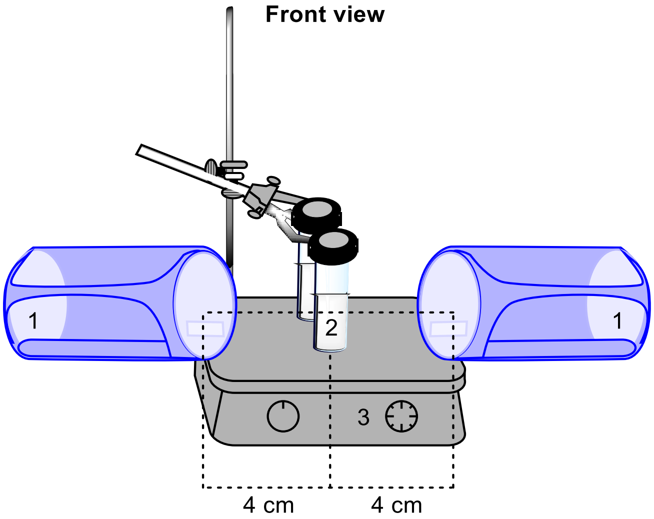


**Figure S3**: Photoredox set up with two vials. 1 = Kessil lamps. 2 = vial. 3 = hot plate. The vials are attached together using two elastic bands positioned over the screw-caps.

Sensitivity Screen

Deviations in reaction conditions often encountered between laboratories were examined using a sensitivity screen as described by Glorius (Table S6).[8] The reaction was found to be tolerant to most deviations and only high concentrations (Entry 2) and high levels of oxygen (i.e. under air; Entry 4) led to a significant drop in yield. Using repurified oxetane acid **1** increased the yield of **2a** by 14% (+23% yield deviation).

**Table S6** Sensitivity screen of the alkylation of 3-aryl-oxetanes. Reactions were carried out on a 0.2 mmol scale using **General Conditions A** with the noted deviations.

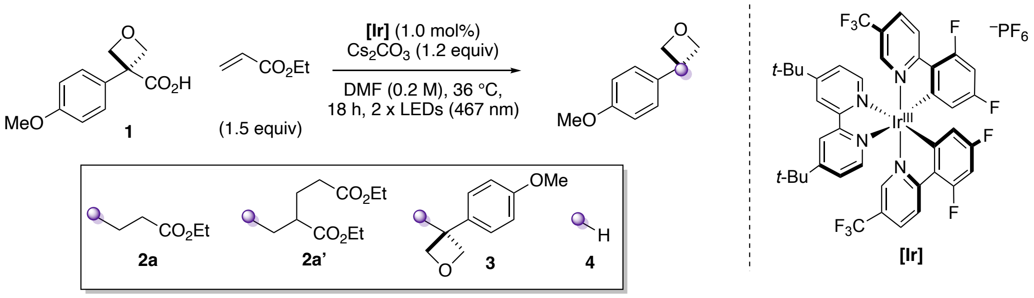


| Entry | Deviation from standard conditions | Yield / %[a] 2a | 2a' | 3 | 4 | Yield deviation / % |
|---|---|---|---|---|---|---|
| 1[b] | None | 61 | 8 | 1 | 1 | 0 |
| 2 | High concentration (0.25 M) | 41 | 7 | 3 | 1 | –33 |
| 3 | Low concentration (0.15 M) | 51 | 9 | 3 | 1 | –16 |
| 4 | High $O_2$ (sealed under air) | 44 | 8 | 11 | 1 | –28 |
| 5 | Moderate $O_2$ (solvent not sparged) | 60 | 7 | 3 | 1 | –2 |
| 6 | Added water (+10 μL $H_2O$ {1% *v/v*}) | 57 | 6 | 2 | 1 | –7 |
| 7[c] | High temperature (60 °C) | 55 | 8 | 1 | 1 | –10 |
| 8[d] | Low temperature (28 °C) | 54 | 11 | 3 | 0 | –11 |
| 9 | Low light intensity (dial set to 75%) | 57 | 8 | 1 | 1 | –7 |
| 10 | 2 × 440 nm Kessil lamps | 62 | 9 | 3 | 1 | +2 |
| 11 | Increased scale (0.5 mmol) | 53 | 6 | 2 | 2 | –13 |
| 12[e] | Repurified **1** (99.9% purity) | 75 (67) | 15 | 4 | 3 | +23 |

[a] Yields calculated by analysis of the $^1H$ NMR spectrum of the crude mixture of the reaction using 1,3,5-trimethoxybenzene as internal standard and a 30 s relaxation delay (d1). Isolated yield in parenthesis. [b] Reported yields are an average of 6 experiments. [c] Reaction set-up with vial covered in aluminum foil. Temperature measured in DMF prior to reaction. [d] Fan placed 14 cm away from vial. Temperature measured in DMF prior to reaction. [e] **1** (previously 98.9% pure) repurified by trituration from a $CH_2Cl_2$/pentane mixture. See page S15 for details.

**Synthesis of Oxetane and Azetidine Carboxylic Acid Starting Materials**

All carboxylic acids used as starting materials were either commercially available or synthesized from the corresponding alcohol according to our previously reported procedure.[9] See Scheme S3 as an example of the synthesis of oxetane carboxylic acid **1** in a >4 g scale.

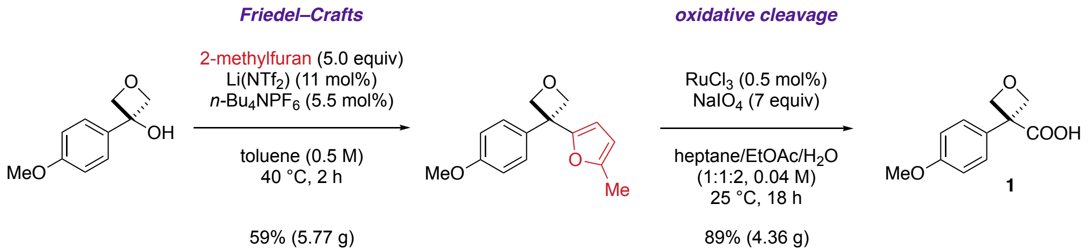


**Scheme S3** 4 g synthesis of oxetane carboxylic acid **1** as an example of the general route towards oxetane, azetidine and cyclobutane carboxylic acid starting materials.

<u>Further details:</u>

Oxetane carboxylic acid **1** is a solid with color ranging from off-white to brown and varying from batch to batch, presumably caused by traces of an impurity from the oxidative cleavage step. The brown color can be reduced (yet not completely removed on most occasions) by drying under high vacuum (the impurity was found to be slightly volatile). A batch of 'brown' carboxylic acid (Figure S4) was determined to be 98.9% pure by $^1$H NMR using 1,3,5-trimethoxybenzene as internal standard and which gave consistent yields with other synthesized batches (Table S5; batch 1 = 'white' acid and batch 2 = 'brown' acid).

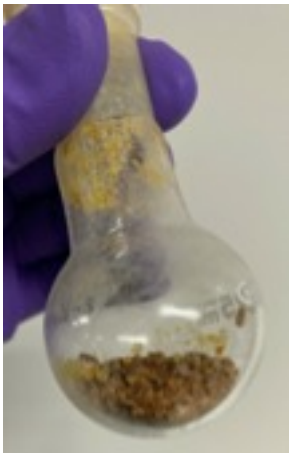

**Figure S4** 'Brown' oxetane carboxylic acid **1** (purity = 98.9%).

A sample of **1** was further purified to 99.9% purity by trituration from $CH_2Cl_2$/pentane by the following procedure (also see Scheme S4): oxetane acid **1** (1.5 g; 81.0% purity) was dissolved in $CH_2Cl_2$ (30 mL) and pentane was added (30 mL) until a solid precipitated. The solid was filtered and washed with pentane (10 mL) to afford **1** in 99.9% purity as a crystalline beige solid (784 mg, Figure S5). The filtrate was filtered and washed with pentane (10 mL) a second time to afford further **1** in 96.2% purity as a beige solid (291 mg). The filtrate was filtered and washed with pentane (10 mL) a third time to afford further **1** in 93.2% purity as a white solid (134 mg). The remaining filtrate was concentrated *in vacuo* using a rotatory evaporator to afford **1** as a dark brown solid in 52.6% purity (328 mg).

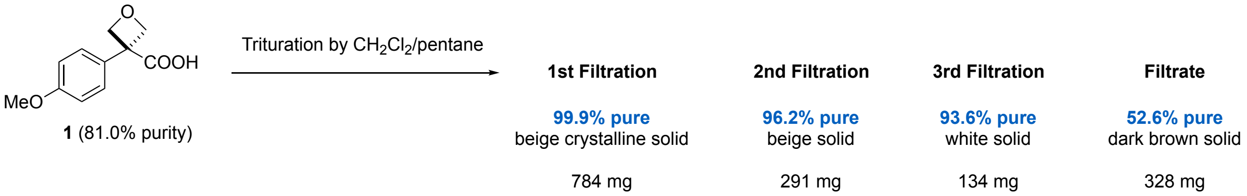

**Scheme S4** Repurification of **1** by trituration with $CH_2Cl_2$/pentane.

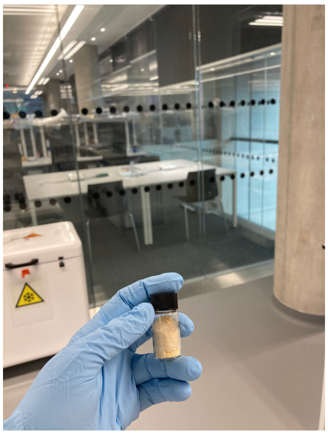

**Figure S5** Repurified oxetane carboxylic acid **1** (purity = 99.9%).

Using repurified **1** in the photoredox protocol increased the yield of **2a** by 14% (from 61 to 75% yield; see Table S6) and the overall mass balance of the reaction by 26% (from 71 to 97% total mass accounted).

**Examples of Scope with Formation of More than One Product**

In some of the examples depicted in Scheme 2, significant amounts of di-addition products, oxetane dimer **3** and reduced oxetane **4** were isolated. See Scheme S5 for further information on the side products isolated alongside the mentioned examples.

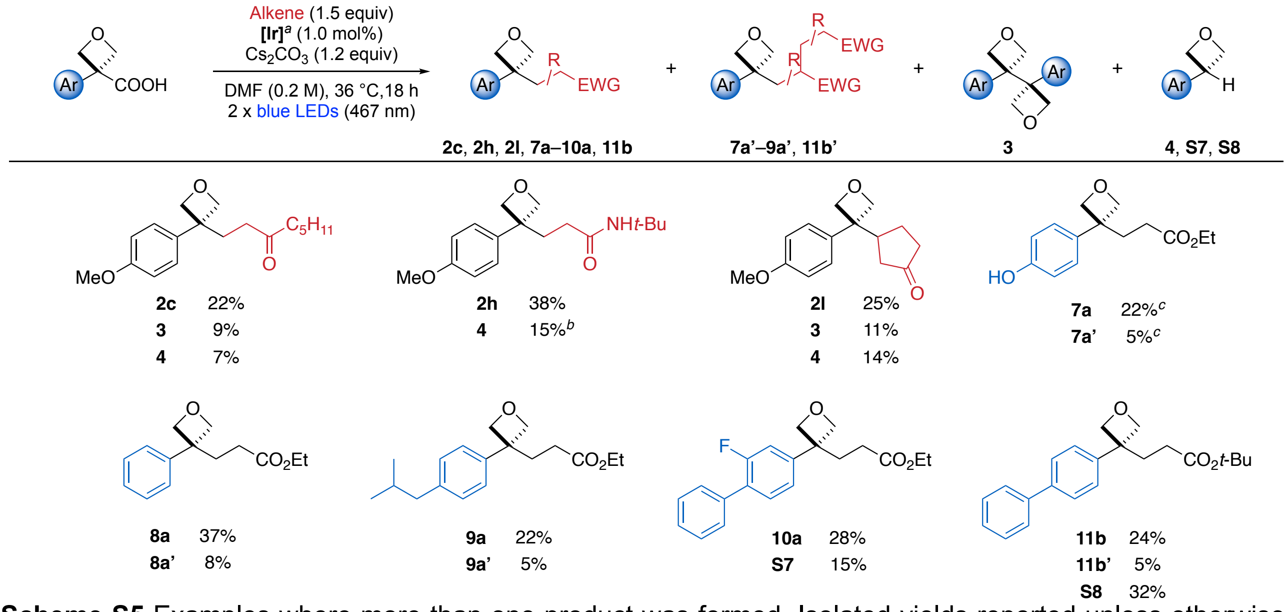


**Scheme S5** Examples where more than one product was formed. Isolated yields reported unless otherwise stated. [a] **[Ir]** = [Ir{dF($CF_3$)ppy}$_2$(dtbbpy)]$PF_6$. [b] Yield calculated by analysis of the $^1$H NMR spectrum of the crude mixture of the reaction using 1,3,5-trimethoxybenzene as internal standard. [c] Using TIPS-protected oxetane acid.

## Low Yielding and Unsuccessful Radical Acceptors

Scheme S6 shows additional oxetane products that were obtained in low yields (<5%; not isolated) and examples where the reaction did not yield the desired product. Di-acceptors seem to generate a too stable anion which is propense to undergo multiple Michael additions and lead to oligomerization products. β-Substituted alkenes, especially cyclic ones, presumably cause a slower rate of addition of the oxetane radical into the acceptor due to an increased steric barrier and thus, lead to high amounts of oxetane dimer **3** and reduced oxetane **4**.

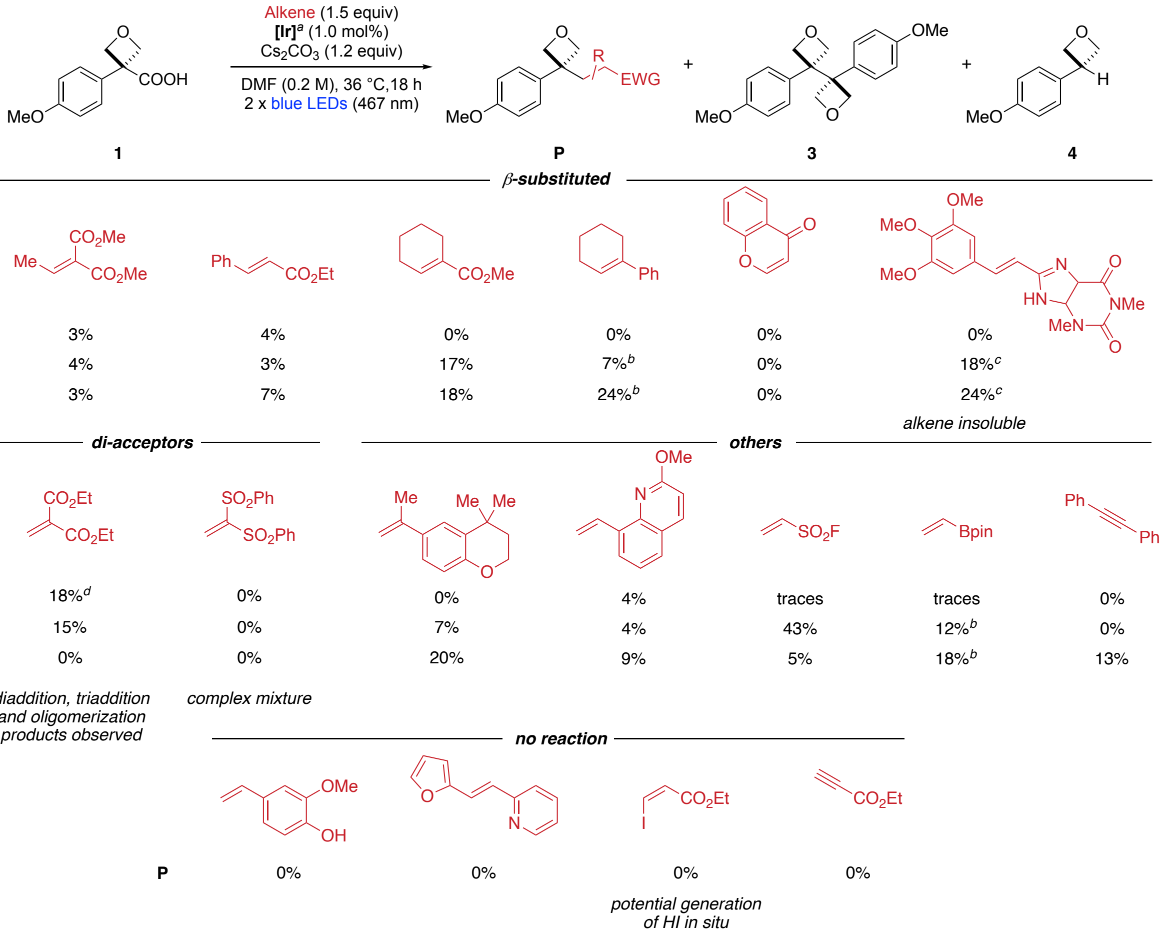


**Scheme S6** Low yielding and unsuccessful radical acceptors. Yields calculated by analysis of the $^1$H NMR spectrum of the crude mixture of the reaction using 1,3,5-trimethoxybenzene as internal standard. [a] **[Ir]** = [Ir{dF(CF$_3$)ppy}$_2$(dtbbpy)]PF$_6$. [b] Estimated yields based on the mass of the crude reaction mixture and the ratio of **3** and **4** in the $^1$H NMR spectrum of the crude mixture. [c] Isolated yields. [d] Product could not be isolated in sufficient purity and was obtained as a mixture with dimer **3** and oligomers of varying lengths.

**Mechanistic Investigations**

**Computational Details**

Methods

Quantum mechanical calculations were carried out using ORCA (v. 5.0.1).[10] Conformationally-sampled stationary points were initially identified using autodE (v. 1.2.0),[11] using the default settings: Low energy conformers were generated using the ETKDGv2 algorithm[12] implemented in RDKit (v. 2018.09.1) and optimized sequentially with xTB (v. 6.3.3) at the GFN2-xTB level,[13] and ORCA at the PBE0-D3BJ/def2-TZVP//PBE0-D3BJ/def2-SVP level.[14] Stationary points for the radical stability and Giese equilibria were subsequently refined at the CPCM(DMF)-ωB97X-D3/def2-TZVP//ωB97X-D3/def2-SVP level, using 'tight' optimization and SCF criteria ($10^{-6}$ and $10^{-8}$ Ha tolerance, respectively) and the RIJCOSX resolution-of-identity approximation.[15] Stationary points for the redox potential calculations were refined at the SMD(DMF)-M06-2X/ma-def2-TZVP//ωB97X-D3/def2-SVP level.[16] All open-shell systems were calculated using an unrestricted formalism. Thermodynamic corrections were applied from the optimization level of theory using the otherm code.[17] Entropies were calculated using the quasi-RRHO approximation ($\omega_0$ = 45 $cm^{-1}$, $\alpha$ = 4, $T$ = 298.15 K, 1 M standard state).[18] Atomic charges and spins were calculated both using the Hirshfeld[19] and the NPA method,[20] implemented in NBO v. 7.0, using densities obtained at the single-point level.

Cartesian coordinates of all optimized structures can be found at the Imperial College London Research Data Repository: https://doi.org/10.14469/hpc/10668.

Calculation of Reduction Potential

The standard reduction potential of the oxetane radical **III** was calculated using the equation

$$E^{\circ} = \frac{-\Delta G^{\circ}}{nF}$$

where $\Delta G^{o}$ is the standard free energy change (in this case at 1 M concentration) associated with the reduction of the oxetane radical, *n* is the number of electrons transferred (in this case 1), and *F* is the Faraday constant (96485 C $mol^{-1}$ ≡ 23.061 kcal volt-gram-$equivalent^{-1}$). Using this equation, and taking the free energy of the electron as –0.04 eV based on the 'ion convention' formalism and Fermi-Dirac statistics for comparison with experimental data,[21] the value of $\Delta G^{o}$ for the reduction of the oxetane radical **III** is –65.8 kcal $mol^{-1}$, and its reduction potential is 2.85 V. Using the convention of referencing this latter value to the standard calomel electrode (SCE, 0.244 V), which itself is referenced to the standard hydrogen electrode (and therefore requires a further correction of 4.28 V),[21] we arrive at a value of **$E^{o}$ = –1.67 V vs SCE** for the reduction of oxetane radical **III**. The potential for the reduction of Ir(III) to Ir(II) for [Ir{dF($CF_3$)ppy}$_2$(dtbbpy)]$PF_6$ is known experimentally to be –1.34 V vs SCE.[5] Subsequently, the reduction of the oxetane radical by Ir(II) corresponds to a cell potential of approximately –1.67 – (–1.34) = –0.33 V ≡ 7.6 kcal $mol^{-1}$, so is therefore thermodynamically disfavored and unlikely to play a role in the catalytic cycle.

In contrast, reduction of the radical derived from the Giese addition to **III** is much more facile; following the same procedure as above, we arrive at a value of **$E^{o}$ = –0.80 V vs SCE**. As a result, reduction of this radical by Ir(II) corresponds to a cell potential of approximately –0.80 – (–1.34) = +0.54 V ≡ –12.4 kcal $mol^{-1}$, providing evidence for the involvement of enolate formation in the catalytic cycle.

The likelihood of the reduction of some alkene radical acceptors were also investigated, and each was found to be thermodynamically unfavorable.

**Table S7.** Differences in electronic energy (Δ*E*), zero-point energy (ΔZPE), enthalpy (Δ*H*), entropy (*T*Δ*S*) and Gibbs free energy (Δ*G*) at the optimization level of theory, and Δ*E*, Δ*H* and Δ*G* at the single-point level of theory obtained using thermal corrections from the optimization level, for the redox processes described above. All energies in kcal mol$^{-1}$, and potentials in V. Gibbs free energies calculated at 298.15 K and 1 M.

| | ωB97X-D3/def2-SVP | | | | | SMD(DMF)-M06-2X/ma-def2-TZVP | | | | |
|---|---|---|---|---|---|---|---|---|---|---|
| **Process** | **Δ*E*** | **ΔZPE** | **Δ*H*** | ***T*Δ*S*** | **Δ*G* (1 atm)** | **Δ*E*** | **Δ*H*** | **Δ*G* (1 M)** | ***E*º** | ***E*º vs SCE** |
| **III**$^{\bullet}$ + e$^{-}$ --> **III**$^{-}$ | -11.0 | -1.7 | -12.6 | 0.6 | -12.3 | -61.0 | -62.6 | -65.8 | 2.85 | -1.67 |
| **III**-adduct$^{\bullet}$ + e$^{-}$ --> **III**-adduct$^{-}$ | -33.7 | -1.0 | -34.8 | -0.6 | -33.4 | -83.1 | -84.2 | -85.8 | 3.72 | -0.80 |
| cyclohexenyl acrylate + e$^{-}$ --> radical anion | 15.09 | -3.24 | 12.09 | 0.61 | 12.34 | -36.72 | -39.72 | -44.36 | 1.92 | -2.60 |
| 1-phenyl cyclohexene + e$^{-}$ --> radical anion | 17.88 | -3.60 | 14.62 | 0.61 | 14.87 | -32.95 | -36.21 | -41.11 | 1.78 | -2.74 |
| Methyl acrylate + e$^{-}$ --> radical anion | - | - | - | - | - | - | - | - | - | -2.10,[a] -2.60[b] |
| [Ir(III)] + e$^{-}$ --> [Ir(II)] | - | - | - | - | - | - | - | - | - | -1.34[c] |

[a] Experimental value taken from Ref.29. [b] Experimental value taken from Ref. 30. [c] Experimental value taken from Ref. 5. [Ir] = [Ir{dF($CF_3$)ppy}$_2$(dtbbpy)]$PF_6$.

Radical Stability

***Radical stability equilibria:***

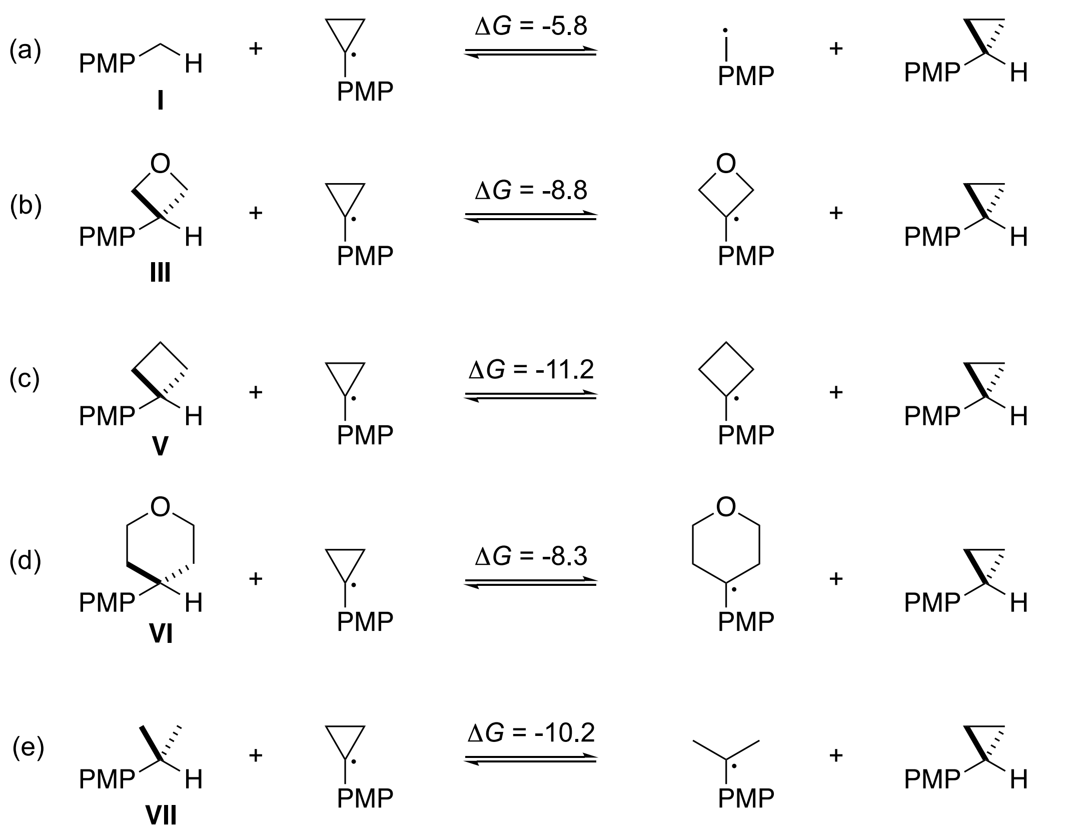


**Figure S6.** H-atom transfer equilibria relative to benzylcyclopropyl radical **II** in kcal mol$^{-1}$, calculated at the CPCM(DMF)-ωB97X-D3/def2-TZVP//ωB97X-D3/def2-SVP level. Free energies calculated at 298.15 K and 1 M.

**Table S8.** Differences in electronic energy ($\Delta E$), zero-point energy ($\Delta$ZPE), enthalpy ($\Delta H$), entropy ($T\Delta S$) and Gibbs free energy ($\Delta G$) at the optimization level of theory, and $\Delta E$, $\Delta H$ and $\Delta G$ at the single-point level of theory obtained using thermal corrections from the optimization level, for the reactions shown in Figure S6. All energies in kcal mol$^{-1}$, and Gibbs free energies calculated at 298.15 K and 1 M.

| | ωB97X-D3/def2-SVP | | | | | CPCM(DMF)- ωB97X-D3/def2-TZVP | | |
|---|---|---|---|---|---|---|---|---|
| **Reaction** | **Δ*E*** | **ΔZPE** | **Δ*H*** | ***T*Δ*S*** | **Δ*G*** | **Δ*E*** | **Δ*H*** | **Δ*G*** |
| a | -5.7 | 0.7 | -5.5 | -1.2 | -4.4 | -7.1 | -7.0 | -5.8 |
| b | -7.8 | 0.1 | -7.9 | -0.4 | -7.5 | -9.2 | -9.2 | -8.8 |
| c | -10.9 | 0.0 | -10.9 | -0.1 | -10.8 | -11.3 | -11.3 | -11.2 |
| d | -8.6 | 0.6 | -8.2 | -0.5 | -7.7 | -9.2 | -8.8 | -8.3 |
| e | -9.9 | 0.2 | -9.8 | -0.1 | -9.6 | -10.5 | -10.3 | -10.2 |

Giese Addition

***Giese equilibria:***

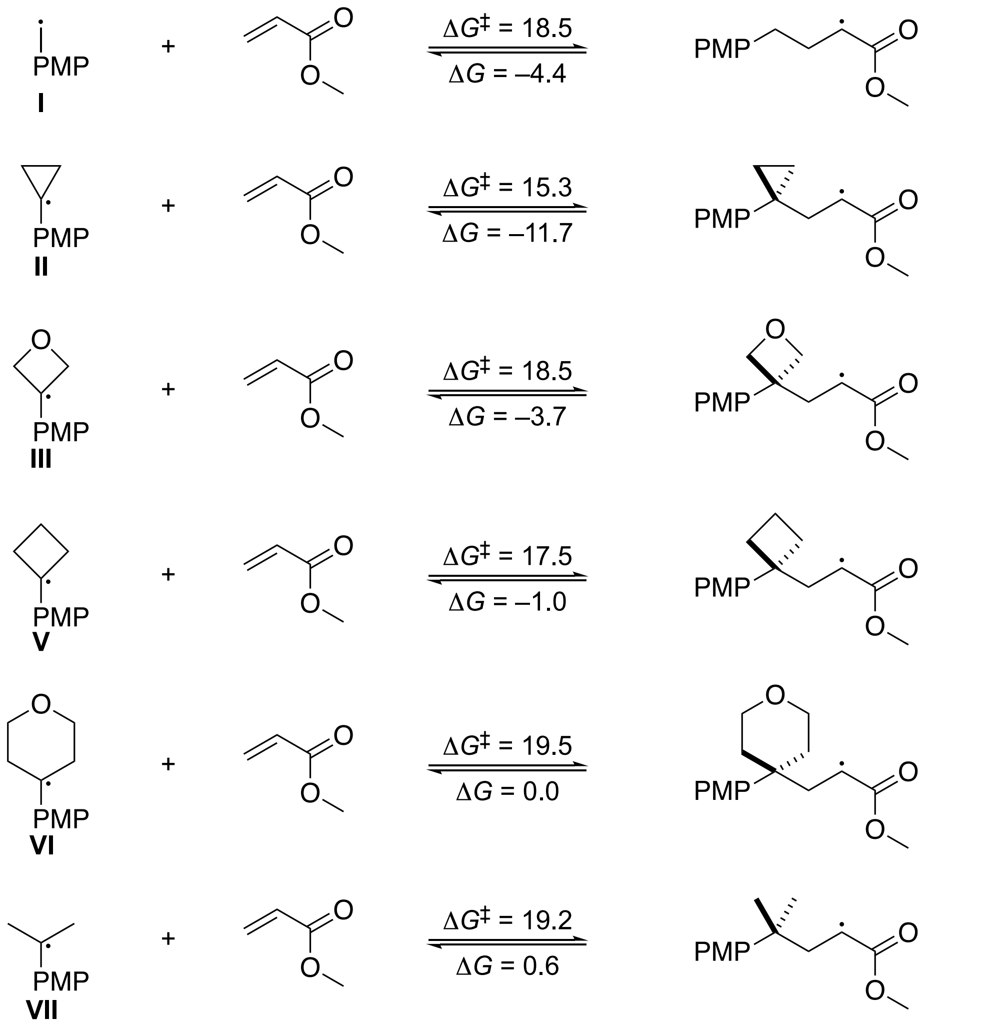


**Figure S7.** Giese addition barriers and equilibria in kcal mol$^{-1}$, calculated at the CPCM(DMF)-ωB97X-D3/def2-TZVP//ωB97X-D3/def2-SVP level. Free energies calculated at 298.15 K and 1 M.

**Table S9.** Differences in electronic energy (Δ*E*), zero-point energy (ΔZPE), enthalpy (Δ*H*), entropy (*T*Δ*S*) and Gibbs free energy (Δ*G*) at the optimization level of theory, and Δ*E*, Δ*H* and Δ*G* at the single-point level of theory obtained using thermal corrections from the optimization level, for the reactions shown in Figure S7. All energies in kcal $mol^{-1}$, and Gibbs free energies calculated at 298.15 K and either 1 atm or 1 M, as specified.

| | | ωB97X-D3/def2-SVP | | | | | CPCM(DMF)-ωB97X-D3/def2-TZVP | | | |
|---|---|---|---|---|---|---|---|---|---|---|
| **System** | **Giese addition** | **Δ*E*** | **ΔZPE** | **Δ*H*** | ***T*Δ*S*** | **Δ*G* (1 atm)** | **Δ*E*** | **Δ*H*** | **Δ*G* (1 atm)** | **Δ*G* (1 M)** |
| **I** | TS | 1.9 | 1.2 | 2.8 | -13.6 | 16.4 | 5.0 | 5.9 | 20.4 | 18.5 |
| | adduct | -26.4 | 3.5 | -23.2 | -12.8 | -10.4 | -21.7 | -18.5 | -2.5 | -4.4 |
| **II** | TS | -0.6 | 1.1 | 0.2 | -13.1 | 13.4 | 2.4 | 3.2 | 17.2 | 15.3 |
| | adduct | -35.1 | 3.4 | -32.2 | -13.8 | -18.5 | -29.4 | -26.5 | -9.8 | -11.7 |
| **III** | TS | 2.3 | 1.1 | 3.2 | -13.2 | 16.4 | 5.5 | 6.3 | 20.4 | 18.5 |
| | adduct | -25.7 | 3.1 | -22.9 | -13.1 | -9.9 | -20.4 | -17.6 | -1.8 | -3.7 |
| **V** | TS | 1.8 | 1.2 | 2.7 | -13.7 | 16.4 | 4.0 | 4.8 | 19.4 | 17.5 |
| | adduct | -24.0 | 3.5 | -21.3 | -14.7 | -6.7 | -19.1 | -16.4 | 0.9 | -1.0 |
| **VI** | TS | 2.2 | 1.2 | 3.1 | -13.8 | 16.9 | 5.9 | 6.8 | 21.4 | 19.5 |
| | adduct | -22.9 | 3.3 | -20.3 | -14.5 | -5.9 | -17.7 | -15.1 | 1.9 | 0.0 |
| **VII** | TS | 2.9 | 1.5 | 3.8 | -14.0 | 17.9 | 5.1 | 6.1 | 21.1 | 19.2 |
| | adduct | -21.8 | 3.6 | -19.1 | -14.7 | -4.4 | -17.6 | -14.9 | 2.5 | 0.6 |

## Benzylic Spin Density

**Table S10.** Benzylic C–C bond length (Å), spin density (*e*) at the benzylic position, and NBO second order perturbation energy, $\Delta E^{(2)}$ (kcal mol$^{-1}$), for the benzylic radicals **I**-**III** and **V**-**VII**.

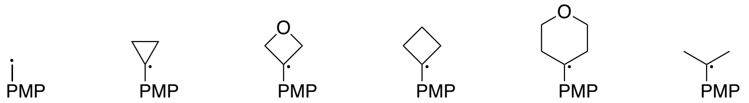


**I** **II** **III** **V** **VI** **VII**

| | | Benzylic spin density / *e* | | $\Delta E^{(2)}$ / kcal mol$^{-1}$ |
|---|---|---|---|---|
| **Molecule** | **Benzylic C–C / Å** | **Hirshfeld** | **NPA** | **p→π*** |
| **I** | 1.407 | 0.604 | 0.747 | 51.8 |
| **II** | 1.398 | 0.470 | 0.667 | 66.2 |
| **III** | 1.404 | 0.478 | 0.672 | 62.1 |
| **V** | 1.410 | 0.483 | 0.674 | 65.1 |
| **VI** | 1.429 | 0.488 | 0.683 | 60.4 |
| **VII** | 1.431 | 0.507 | 0.700 | 59.1 |

**Deuteration Studies**

Oxetane carboxylic acid **1** was deuterated by dissolving and stirring in $CD_3OD$ (ca. 100 mg in 1 mL). The solvent was then removed *in vacuo* using a rotatory evaporator and complete removal was achieved by drying under high vacuum (~4 h at ca. 1 × $10^{-3}$ mbar).

Full deuteration of the acid was confirmed by IR which shows a shift in the O–H (O–D) stretching frequency from 3444 $cm^{-1}$ (ref. 9) to 2228 $cm^{-1}$ due to a smaller bond stretching frequency of the O–D bond caused by an almost two-fold increase of the reduced mass μ (Figure S8).

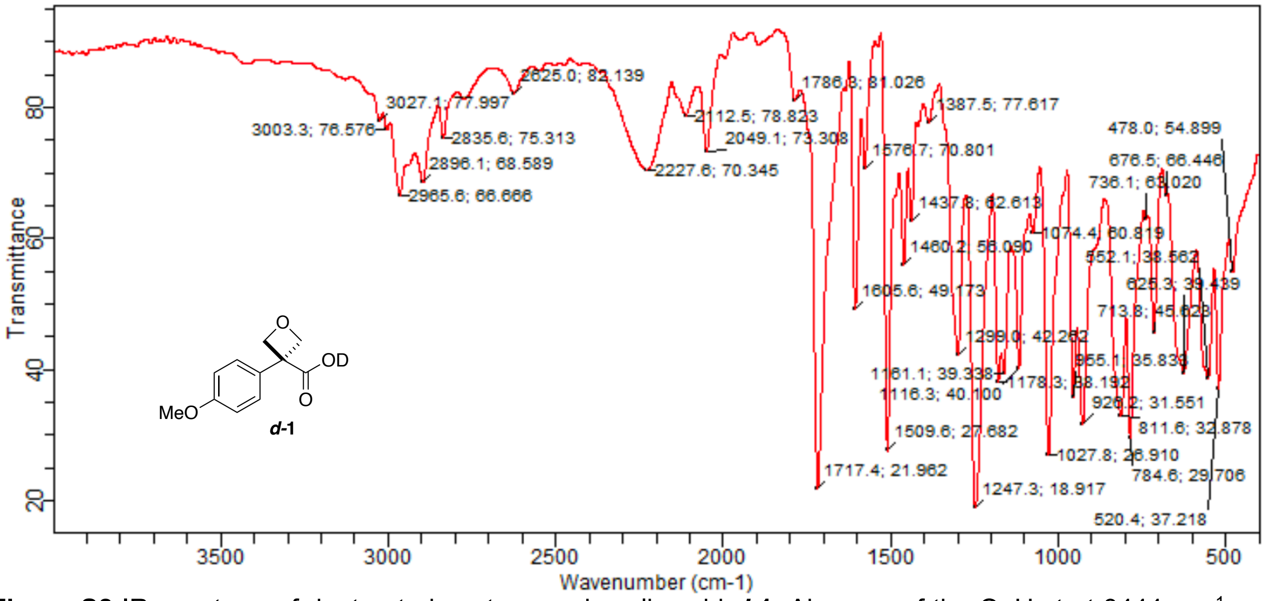


**Figure S8** IR spectrum of deuterated oxetane carboxylic acid ***d*-1**. Absence of the O–H st at 3444 $cm^{-1}$ and appearance of a new broad signal at 2228 $cm^{-1}$ (O–D) confirmed the deuteration. Based on the differences in reduced mass, a 0.728 times smaller stretching frequency of the O–D bond would be expected (theoretical: 2507 $cm^{-1}$) which is in good agreement with the observed value (2228 $cm^{-1}$). The O–H st at 3444 $cm^{-1}$ in ref. 9 is also very broad and stretches between ca. 3500 and 2500 $cm^{-1}$.

Reaction of Oxetane **1** with Ethyl Acrylate

Decarboxylative alkylation of oxetane **1** with ethyl acrylate is believed to involve the protonation of an enolate intermediate as the final step. Deuteration experiments were conducted to support this and showed high deuterium incorporation into **2a** only with $D_2O$ as additive. (Table S11). The enolate was shown to be protonated during the course- and not upon quenching with water at the end of the reaction (Entry 7). Further, the amount of **2a'** formed was unaffected by the water content of the reaction (also see Table S12).

**Table S11** Deuteration experiments in the reaction of oxetane acid **1** with ethyl acrylate. Reactions were carried out on a 0.2 mmol scale using **General Conditions A** with noted deviations.

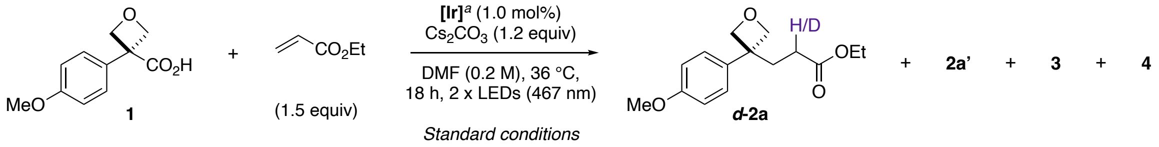


| Entry | Deviation from standard conditions | Yields / %[b] | | | |
|---|---|---|---|---|---|
| | | **2a / D-incorporation** | **2a'** | **3** | **4** |
| 1[c] | none | 61 / NA | 8 | 1 | 1 |
| 2 | ***d*-1** (COOD) | 57 / 0 | 13 | 2 | 2 |
| 3 | Using DMF-$d_7$[d] | 67 / 0 | 8 | 2 | 0 |
| 4 | Using DMF-$d_7$ + ***d*-1** (COOD)[d] | 63 / 0 | 11 | 3 | 0 |
| 5 | + 2.77 equiv $D_2O$[e] | 64 / 58[f] | 8 | 2 | 0 |
| 6 | + 10.0 equiv $D_2O$ | 56 / 71[f] (78)[g] | 10 | 1 | 0 |
| 7 | $D_2O$ quench[h] | 60 / 0 | 8 | 3 | 0 |

[a] **[Ir]** = [Ir{dF($CF_3$)ppy}$_2$(dtbbpy)]$PF_6$. [b] Yields and percentage of deuteration (D-incorporation) calculated by analysis of the $^1$H NMR spectrum of the crude mixture of the reaction using 1,3,5-trimethoxybenzene as internal standard and a 30 s relaxation delay (d1). [c] Reported yields are an average of 6 experiments. [d] 0.14 mmol scale. [e] 2.77 equiv $D_2O$ equate to 10 μL (1% *v/v*), which were shown to leave the reaction unaffected (Table S5). [f] Relevant signals in the $^1$H NMR spectrum of the crude reaction mixture obscured by other species. Approximate value given. [g] Degree of deuteration measured after isolation by column chromatography. [h] 1 mL $D_2O$ was added to the reaction mixture and stirred for 5 min; then the standard workup procedure was performed. NA = Not applicable.

Figure S9 shows the spectra for the entries in Table S11 with the different degrees of deuteration.

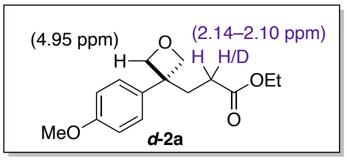

(4.95 ppm)
(2.14–2.10 ppm)
H
H H/D
O
OEt
O
MeO
*d*-**2a**


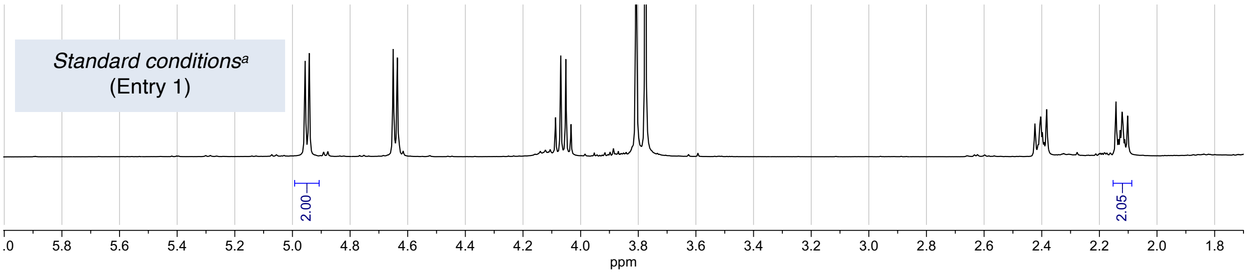

*Standard conditions*[a]
(Entry 1)
2.00
2.05
ppm


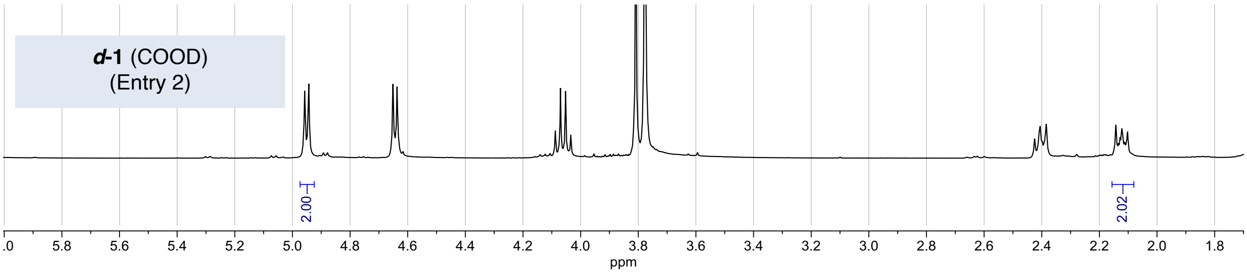

***d*-1** (COOD)
(Entry 2)
2.00
2.02
ppm


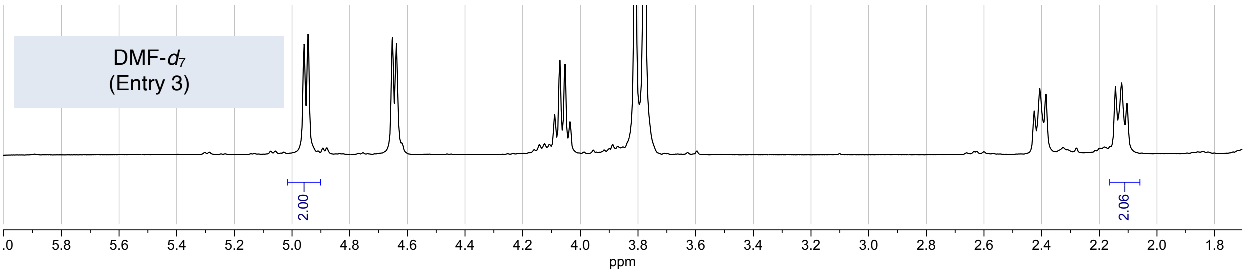

DMF-$d_7$
(Entry 3)
2.00
2.06
ppm


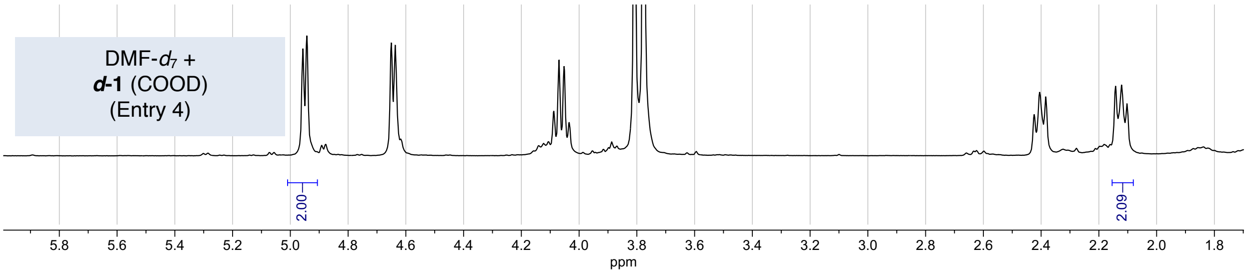

DMF-$d_7$ +
***d*-1** (COOD)
(Entry 4)
2.00
2.09
ppm


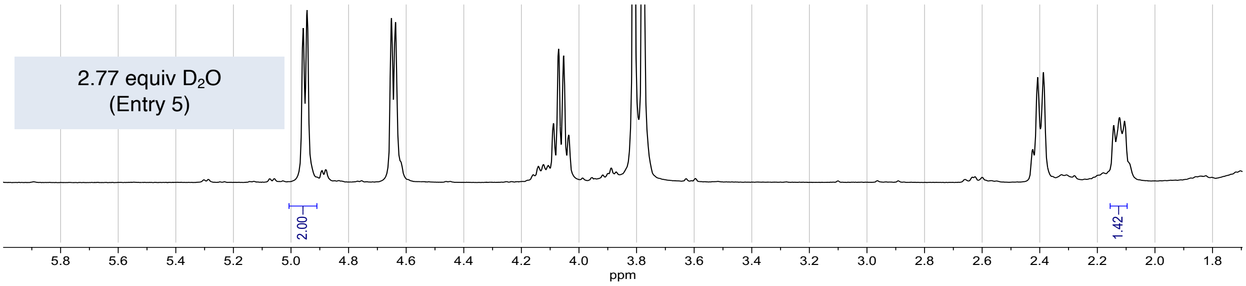

2.77 equiv $D_2O$
(Entry 5)
2.00
1.42
ppm


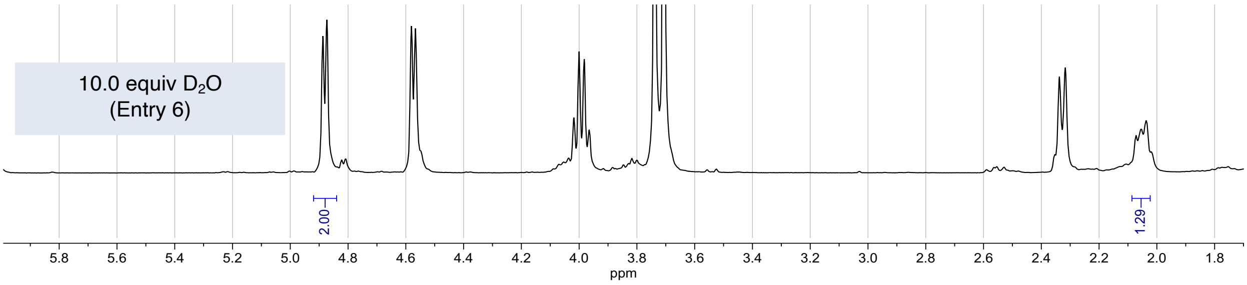

10.0 equiv $D_2O$
(Entry 6)
2.00
1.29
ppm

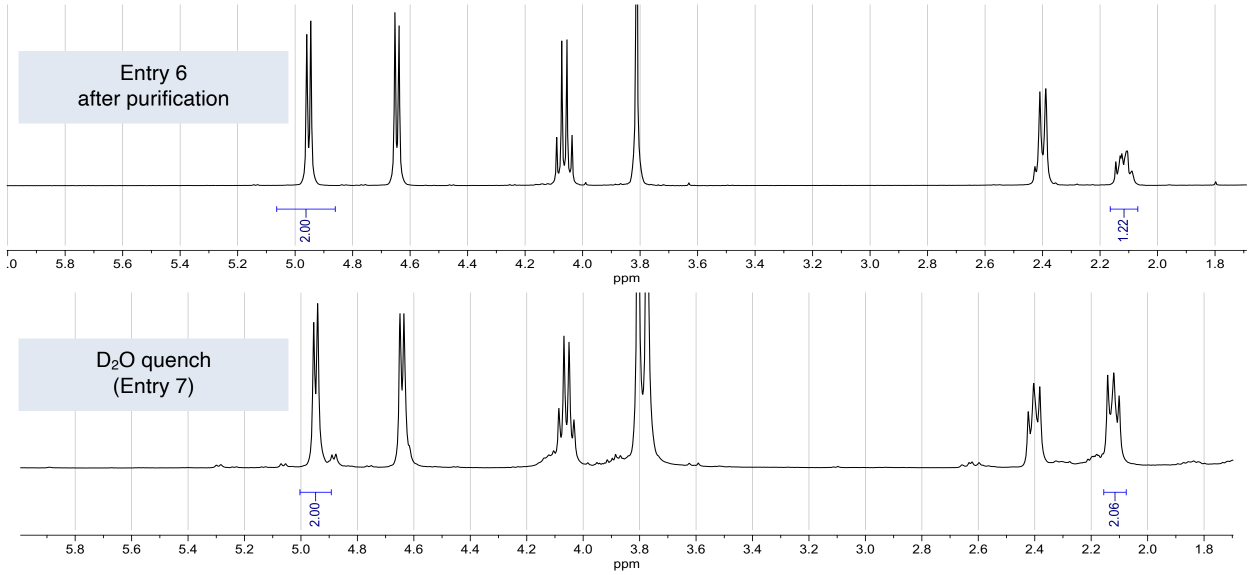


**Figure S9** Spectra of the deuteration studies in Table S11. The chemical shift of the protons alpha to the ester is at ca. 2.14–2.10 ppm. Unambiguous assignment of this signal was performed by HMBC (see page S88). The integrations were normalized to one of the sets of chemically equivalent methylene groups on oxetane (2 H, d, 4.95 ppm). [a] Representative spectrum of the crude reaction mixture of one of the six examples.

The high deuterium incorporation observed with $D_2O$ as additive (Table S11, entries 5–7) prompted us to investigate the Giese reaction under rigorously anhydrous conditions (Table S12). The yield stayed unaffected compared to the standard set-up (entry 2; see **General Conditions A**, page S11) and there was no deuterium incorporation with neither ***d*-1** (COOD) nor a $D_2O$ quench (entries 3–4). From the deuteration studies in Tables S11 and S12 we hence conclude that **2a** is formed through the protonation of an enolate intermediate, but the source of protons remains unclear.

**Table S12** Evaluation of extra dry conditions in the reaction of oxetane acid **1** with ethyl acrylate. Reactions were carried out on a 0.16 mmol scale using **General Conditions A** with noted deviations.

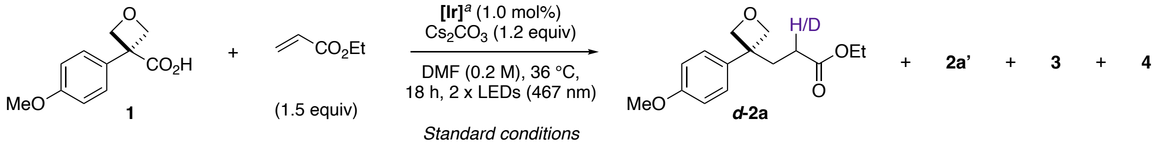


| Entry | Deviation from standard conditions | Yields / %[b] | | | |
|---|---|---|---|---|---|
| | | 2a / D-incorporation | 2a' | 3 | 4 |
| 1 | none | 63 / NA | 12 | 1 | 0 |
| 2 | Extra dry DMF[c] | 68 / NA | 9 | 1 | 0 |
| 3 | Extra dry DMF[c] + ***d*-1** (COOD) | 72 / 0 | 9 | 1 | 0 |
| 4 | Extra dry DMF[c] + $D_2O$ quench[d] | 60 / 0 | 5 | 1 | 0 |

[a] **[Ir]** = [Ir{dF($CF_3$)ppy}$_2$(dtbbpy)]$PF_6$. [b] Yields and percentage of deuteration (D-incorporation) calculated by analysis of the $^1$H NMR spectrum of the crude mixture of the reaction using 1,3,5-trimethoxybenzene as internal standard and a 30 s relaxation delay (d1). [c] $Cs_2CO_3$ dried overnight at 200 °C under high vacuum, **1** and **[Ir]** dried overnight at 25 °C under high vacuum, DMF dried by filtration through a drying column, then over activated molecular sieves for 48 h (water-content measured to be 21 ppm by Karl-Fischer Titration [≙ 0.5 mol% $H_2O$]), solids of reaction mixture evacuated and refilled with Ar (3x) before addition of DMF. [d] 1 mL $D_2O$ was added to the reaction mixture and stirred for 5 min; then the standard workup procedure was performed. NA = Not applicable.

Formation of Reduced Oxetane **4**

Deuterium studies were performed using cyclohexene methyl carboxylate as radical acceptor (Table S13), which had shown increased amounts of reduced oxetane **4** as side product (Scheme S6). See the main text and Figure 5 for further discussion.

**Table S13** Deuteration experiments in the reaction of oxetane acid **1** with cyclohexene methyl carboxylate. Reactions were carried out on a 0.14 mmol scale using **General Conditions A** with the noted deviations.

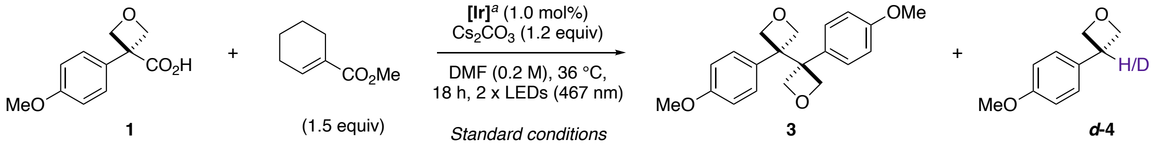


| Entry | Deviation from standard conditions | Yield / %[b] 3 | Yield / %[b] 4 | D-incorporation / %[b] |
|---|---|---|---|---|
| 1 | none | 16 | 16 | NA |
| 2 | Using DMF-*d*7 | 18 | 21 | 7 |
| 3 | ***d*-1** (COOD) | 14 | 10 | 33 |
| 4 | Using DMF-*d*7 + ***d*-1** (COOD) | 6 | 13 | 37 |
| 5[c] | + 2.77 equiv $D_2O$ | 4 | 8 | 75 |
| 6 | + 10.0 equiv $D_2O$ | 10 | 11 | 85 |

[a] **[Ir]** = [Ir{dF($CF_3$)ppy}$_2$(dtbbpy)]$PF_6$. [b] Yields and percentage of deuteration (D-incorporation) calculated by analysis of the $^1$H NMR spectrum of the crude mixture of the reaction using 1,3,5-trimethoxybenzene as internal standard and a 30 s relaxation delay (d1). [c] 2.77 equiv $D_2O$ equate to 7 μL (1% *v/v*), which were shown to leave the reaction unaffected (Table S6).

Figure S10 shows the spectra for the entries in Table S13 with the different degrees of deuteration.

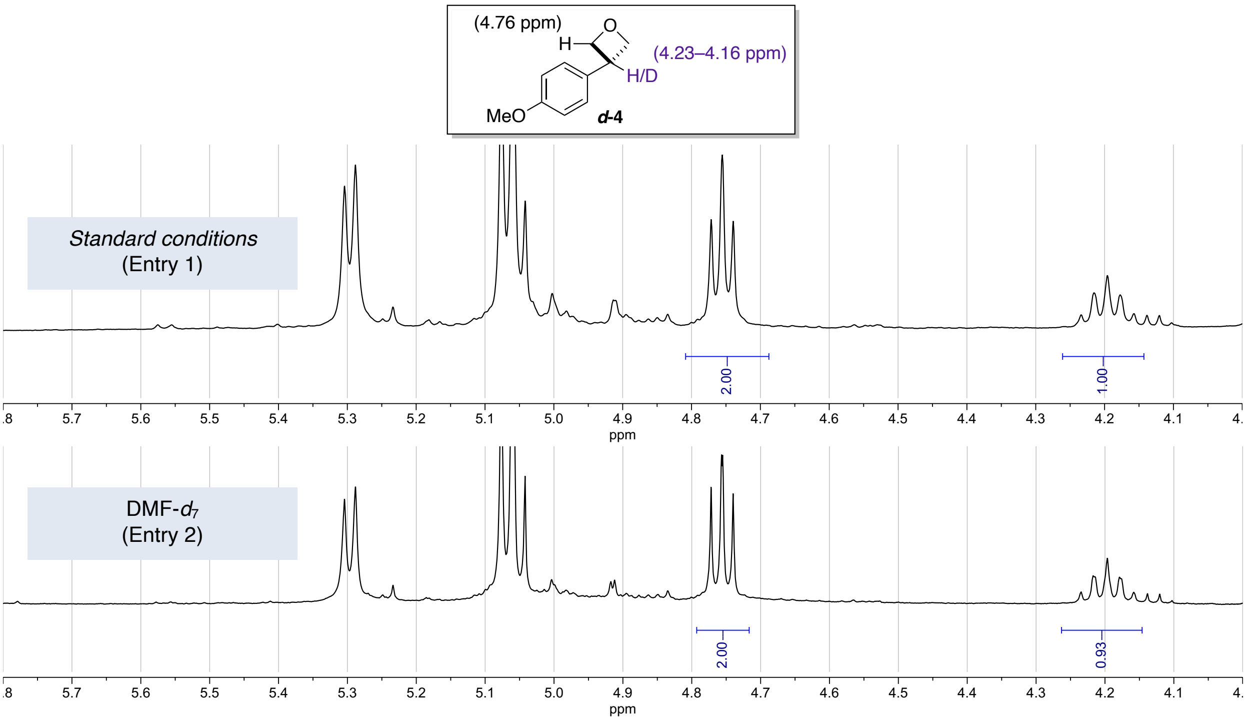

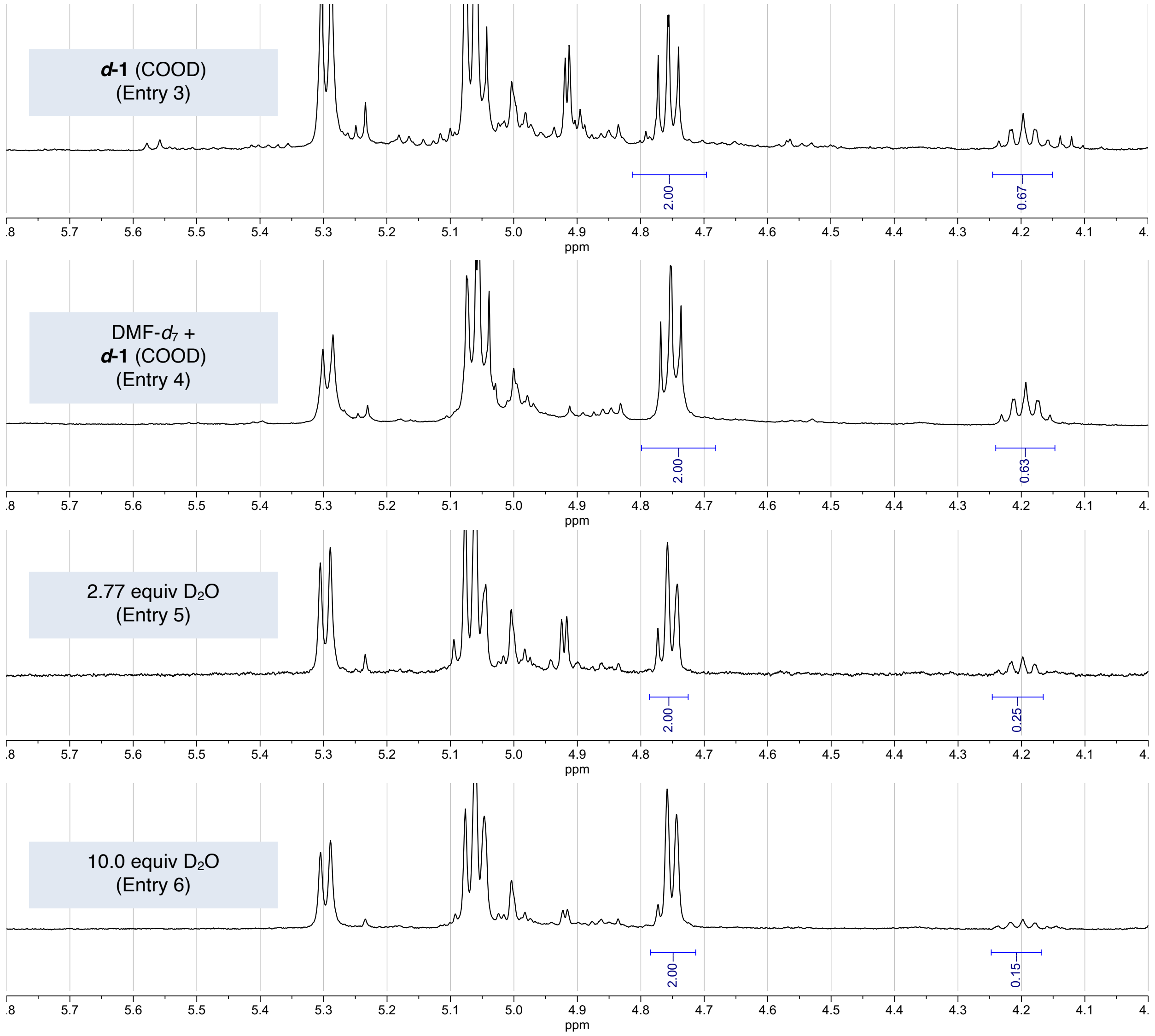


**Figure S10** Spectra of the deuteration studies in Table S13. The chemical shift of the proton in the 3-position of oxetane is at ca. 4.23–4.16 ppm. The integrations were normalized to one of the sets of chemically equivalent methylene groups on oxetane (2 H, dd, 4.76 ppm).

**Reaction profiles for Formation of 2a and 8a**

The reaction profiles for the formation of **2a** and **8a** were constructed to better understand the differences in yield between these two substrates. Reactions were conducted with PMP-oxetane acid **1** and Ph-oxetane acid **S10** using the **Procedure** below for different durations. Each time point represents an independent reaction. Recovery of starting material was assessed by acidifying the basic aq. phase to pH 1 and re-extracting with EtOAc (see **Procedure**). 96% starting material **1** was recovered when performing the workup at $t$ = 0 min. The reaction profiles were conducted with repurified oxetane carboxylic acids. For repurification of **1**, see page S15. **S10** was repurified by column chromatography (30% EtOAc/pentane + 0.5% AcOH).

**Procedure**
An oven-dried 4 mL vial was charged with carboxylic acid (0.10 mmol, 1.0 equiv), oven-dried $Cs_2CO_3$ (39.1 mg, 0.12 mmol, 1.2 equiv) and [Ir{dF($CF_3$)ppy}$_2$(dtbbpy)]$PF_6$ (1.1 mg, 0.001 mmol, 1.0 mol%). The vial was sealed with a screwcap equipped with a PTFE/silicon septum and anhydrous DMF (0.5 mL, 0.2 M) was added by syringe. Argon was bubbled through the mixture for 5 min and ethyl acrylate (18 μL, 0.15 mmol, 1.5 equiv) was added by syringe under argon. After sealing the cap with parafilm on top, the reaction mixture was stirred at 1000 rpm using the set-up shown in Figure S2 or S3 and irradiated with two 467 nm Kessel lamps at 36 °C (heat generated by the lamps). After $t$ the lights were switched off and the reaction mixture was transferred into a separating funnel. Distilled water (10 mL) and $Et_2O$ (10 mL) were added, the layers were separated and the aqueous portion was extracted with $Et_2O$ (2 × 10 mL). The aq. phase was acidified to pH 1 and extracted with EtOAc (3 × 10 mL). The $Et_2O$ and EtOAc extracts were combined separately, dried over $Na_2SO_4$, filtered and concentrated *in vacuo* using a rotatory evaporator. Yields were calculated by analysis of the $^1$H NMR spectrum of the crude mixtures of the reaction ($Et_2O$ and EtOAc extracts separately) using 1,3,5-trimethoxybenzene as internal standard and a 30 s relaxation delay (d1).

The yields of all species at the different time points are given in Table S14, a graphical representation of the reaction profiles for the formation of the Giese products **2a** and **8a** in Figure S11. The following observations are noteworthy:

1. The yield of **2a** is higher than that of **8a** at every time point.
2. The rate of formation of **2a** is higher than that of **8a**.
3. The overall mass balance is higher with PMP-oxetane acid **1** than with Ph-oxetane acid **S10**.
4. The yield of Giese product (both **2a** and **8a**) starts to diminish at very long reaction times with increased formation of di-addition products **2a’** and **8a’**.

**Table S14** Data for reaction profiles for the formation of **2a** and **8a**. Reactions were carried out on a 0.1 mmol scale (oxetane acid) using the **Procedure** described above.

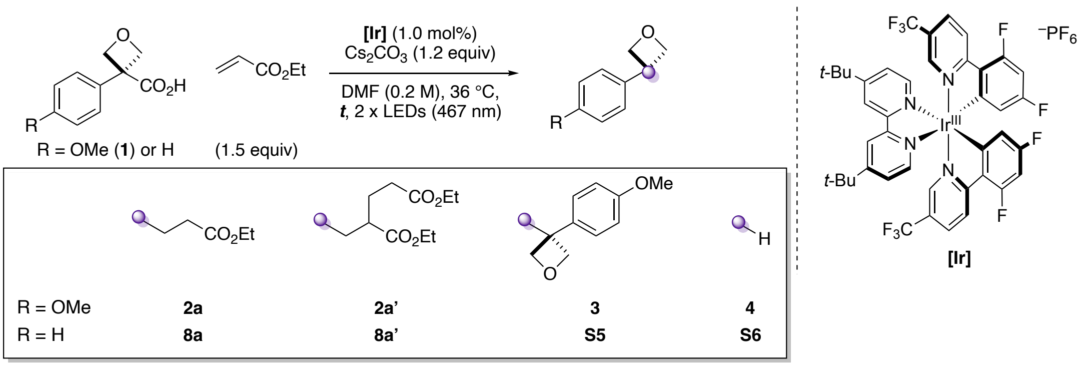


| Entry | R | $t$ / h | Yield / %[a] | | | | | |
|---|---|---|---|---|---|---|---|---|
| | | | 2a/8a | 2a'/8a' | 3/S5 | 4/S6 | RSM | Total |
| 1 | OMe (**1**) | 2 | 36 | 3 | 1 | 1 | 50 | 91 |
| 2 | | 6 | 81 | 10 | 6 | 3 | 0 | 100 |
| 3 | | 14 | 78 | 14 | 4 | 1 | 0 | 97 |
| 4 | | 24 | 77 | 6 | 3 | 1 | 0 | 87 |
| 5 | H (**S10**) | 2 | 15 | 2 | 0 | 0 | 54 | 71 |
| 6 | | 6 | 36 | 3 | 1 | 0 | 22 | 62 |
| 7 | | 14 | 55 | 11 | 2 | 0 | 4 | 72 |
| 8 | | 24 | 51 | 15 | 0 | 0 | 1 | 67 |

[a] Yields calculated by analysis of the $^{1}H$ NMR spectrum of the crude mixture of the reaction using 1,3,5-trimethoxybenzene as internal standard and a 30 s relaxation delay (d1).

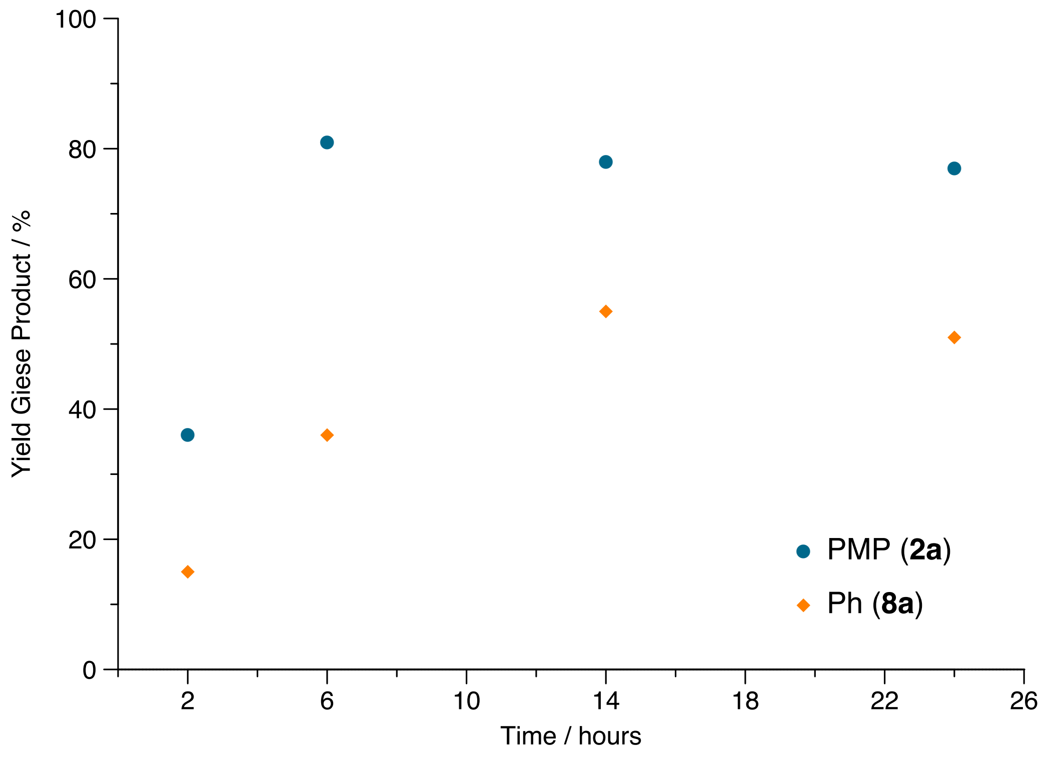


**Figure S11** Comparison of reaction profiles for the formation of **2a** and **8a**. Values from Table S14.

**Cyclic Voltammetry Experiments**

Cyclic voltammetry (CV) measurements were performed on an Autolab PGSTAT204 potentiostat from Metrohm using Nova 2.1 as software. A glassy carbon disc from Metrohm was used as working electrode (3 mm surface area and circular disk geometry), a Pt-plated copper block from IKA (part number: 0040002852) as counter electrode and a Pt-wire as quasi-reference electrode.

Cyclic voltammograms were measured at 25 °C, with a 100 mV $s^{-1}$ scan rate, between –2.2 and +2.2 V, starting at 0 V in the positive scan direction, in 10 mV steps and with 1 mA current range. A quasi-Faraday cage was achieved by wrapping the electrochemical cell in Al-foil. The glassy carbon electrode was polished mechanically in a figure-of-eight motion using an aqueous suspension of nanometric alumina on a polishing pad, between each set of experiments (1 set = CV of analyte (3 scans) + CV of analyte with ferrocene (3 scans)).

Cyclic voltammograms were obtained using a 0.1 M solution of $NBu_4ClO_4$ in MeCN as supporting electrolyte. Oxygen-free conditions were achieved by purging the electrochemical cell with a balloon filled with argon for 5 minutes before each measurement. Three scans were performed per measurement and numerical values were extracted from the third scan. The analyte was measured in 2 mM concentration. Ferrocene (Fc) was added as an internal standard after each measurement (in 1 mM concentration) and the voltammogram was recorded again.[22] Anodic peak potentials ($E_{pa}$) are reported vs the $Fc^+$/Fc couple and converted to vs SCE by adding 380 mV (see Table S15).[23] Carboxylates and enolates were generated *in situ* through the addition of 1.0 equiv $NBu_4OH$ (1 M in MeOH) to the carboxylic acid/ester substrates.

All compounds evaluated showed chemically irreversible oxidation waves, as is common for organic compounds. Here, the oxidation potentials are reported as the anodic peak potentials ($E_{pa}$) instead of the potentials at half the peak ($E_{p/2}$) for better reproducibility, as suggested by Lam.[24] $E_{p/2}$ could nevertheless be obtained by recording the potential at half the value of the maximum current.[25] Raw recorded values are available from the Imperial College London Data Repository at: https://doi.org/10.14469/hpc/10668.

Values were exported from the Nova software as Excel files. Data were analyzed and plotted with the open-source statistical program 'R', version 3.2.2 (April 08, 2015 – "Fire Safety").[26] For clarity, only positive values (0 to +2.2 V) of the cyclic voltammograms are plotted (with the exception of cyclohexene methyl carboxylate where the full voltammogram is plotted).

Cyclic voltammograms of a diverse set of arylacetic carboxylic acids and carboxylates with varying aromatic and benzylic substituents were measured (Table S15, Entries 1–10). The CV of phenyl triflate (Entry 11), cyclohexene methyl carboxylate (Entry 12), and aryl-alkyl oxetane **2a** (both as ester and enolate; entries 13 and 14) were also measured. Two general trends were observed:

1. As previously observed by Ravelli,[27] arylacetic carboxylates with electron-rich aromatic rings show a lower oxidation potential than electron-neutral examples. Specifically, $E^{ox}$(PMP) < $E^{ox}$(Ph) < (Table S15, Entries 2–3). It is noteworthy that the trends in oxidation potential correlate well with the Hammett $\sigma_p$ constants of the different substituents (*p*-OMe: –0.27; *p*-H: 0),[28] supporting the measured oxidation potentials. Note that the oxidation potential of *p*-OTf-phenyl-oxetane carboxylate could not be accurately determined due to several oxidation peaks at low potentials, which might suggest degradation of this compound through oxidative degradation pathways (Figure S16). These low-potential oxidations seem to be a result of the combination of the triflate group with the oxetane carboxylate motif, since they are absent in both other aryl-oxetane carboxylates and unsubstituted phenyl triflate (see Table S15, Entry 11 and Figure S23).
2. Arylacetic carboxylates with benzylic substituents that increase the electron-density at the benzylic position show a lower oxidation potential than others. Specifically, $E^{ox}$(cyclobutane) ~ $E^{ox}$(*gem*-dimethyl) < $E^{ox}$(oxetane) ~ $E^{ox}$(methylene) (Table S15, Entries 2–10). This trend was observed both for PMP and phenyl aromatic rings.

The two trends above suggest that the higher the electron-density at the benzylic position of arylacetic carboxylates is, the lower the oxidation potential of the carboxylate. This general trend can be correlated to the p$K_a$ of the corresponding carboxylic acids: higher electron-density at the benzylic position → higher p$K_a$ (*i.e.* negative charge in the carboxylate less stabilized) → carboxylate easier to oxidize (lower oxidation potential). The nucleophilicity of the corresponding benzylic radicals correlate to the degree of stabilization of a positive charge in the benzylic position (*i.e.* the better stabilized the positive charge, the higher the nucleophilicity of the radical). The nucleophilicity of the radicals should hence also correlate with the CV data (*i.e.* more nucleophilic radical ≙ higher electron-density at benzylic position → lower oxidation potential of carboxylate). The proposed correlation between radical philicity and oxidation potential seems to hold true for our set of arylacetic acids (Figure S12) and could in principle, be generally applicable to estimate relative radical philicities through CV measurements.

Reduction of cyclohexene methyl carboxylate by the reduced form of the photocatalyst (**[Ir$^{II}$]**; $E_{1/2}^{III/II}$ = –1.37 V vs SCE[5]) is unfeasible based on the measured CV (no significant reduction waves observed up to –3.0 V) and calculated reduction potential (–2.60 V vs SCE; see Table S7; also see Figure 5 for the full mechanistic picture). Further, the reduction potential of related methyl acrylate has been reported at –2.10[29] and –2.60 V vs SCE.[30]

Interestingly, the enolate of the standard product (**2a$^-$**) showed an oxidation potential in the range of **[Ir$^{III*}$]** ($E_{1/2}^{III*/II}$ = +1.21 V vs SCE[5])). This was not the case with the ester form of **2a** (Table S15, entries 13–14). These values suggest that enolate **2a$^-$**, but not **2a**, can be oxidized back to the α-ester radical **2a$^•$** by the excited photocatalyst **[Ir$^{III*}$]**. See the main text and Figure 5 for further discussion. The measured oxidation potential of **2a$^-$** is in the range of related enolate compounds.[31]

**Table S15** CV values of arylacetic carboxylates (Entries 1–9), phenyl triflate (Entry 10) and cyclohexene methyl carboxylate (Entry 11). See page S35 for experimental details.

| Entry | Substrate[a] | *E* / V | | | |
|---|---|---|---|---|---|
| | | $E_{pa}$ (Obs) | $E_{1/2}$ ($Fc^+$/Fc) | $E_{pa}$ (vs $Fc^+$/Fc) | $E_{pa}$ (vs SCE) |
| 1 | 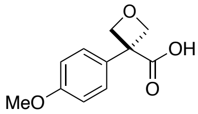 | ≥2.08[b] | +0.45 | ≥1.63[b] | ≥2.01[b] |
| 2 | 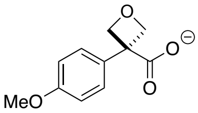 | +1.31 | +0.80 | +0.51 | +0.89 |
| 3 | 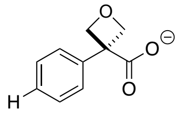 | +1.79 | +1.09 | +0.70 | +1.08 |
| 4 | 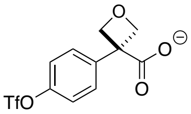 | ND[c] | +0.54 | ND[c] | ND[c] |
| 5 | 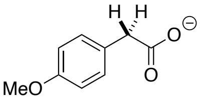 | +1.51 | +1.07 | +0.44 | +0.82 (+0.99)[d] |
| 6 | 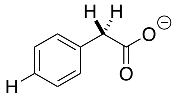 | +1.81 | +1.13 | +0.68 | +1.06 (+1.27)[c] |
| 7 | 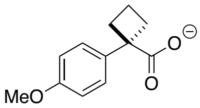 | +1.48 | +1.14 | +0.34 | +0.72 |
| 8 | 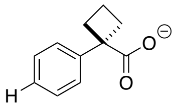 | +1.61 | +1.11 | +0.50 | +0.88 |
| 9 | 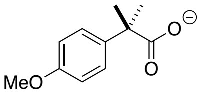 | +1.50 | +1.15 | +0.35 | +0.73 |
| 10 | 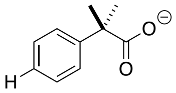 | +1.63 | +1.09 | +0.54 | +0.92 |
| 11 | 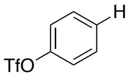 | +1.66 | +0.24 | +1.43 | +1.81 |
| 12 | 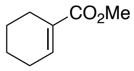 | +1.91 (–1.84)[e] | +0.17 | +1.74 (–2.01)[e] | +2.12 (–1.63)[e] |
| 13 | 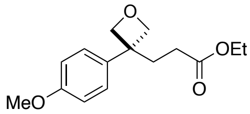 | ≥2.14[b] | +0.47[b] | ≥1.66[b] | ≥2.04[b] |
| 14[f] | 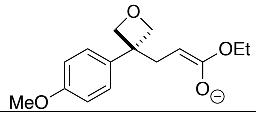 | +1.21 | +0.99 | +0.22 | +0.60 |

[a] Carboxylates measured with $^+NBu_4$ as countercation. [b] Estimated based on shoulder peak. No oxidation peaks between 0 and +2 V. [c] Value could not be determined due to the presence of many oxidation peaks at low potentials. [d] Literature value.[27] [e] Value in parenthesis refers to the cathodic peak potential ($E_{pc}$) value from a small reduction wave with 9 μA; see Figure S24. [f] Enolate generated by adding 1 equiv $NBu_4OH$.

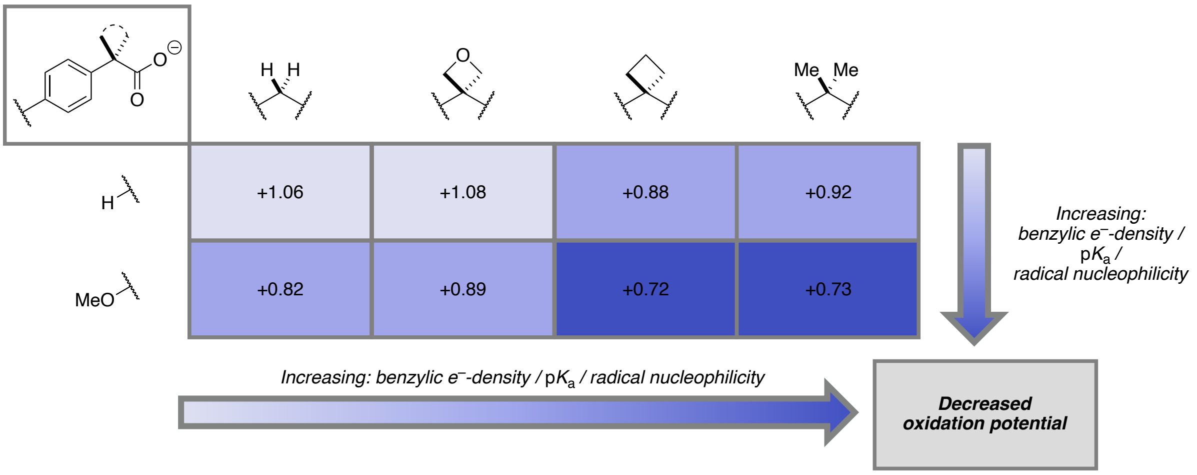


**Figure S12** Comparison of oxidation potentials ($E_{pa}$ / V vs SCE) of different arylacetic acids and correlation to electron-density at benzylic position. $E_{pa}$ values from Table S15.

Figures S13–S26 show the raw measured cyclic voltammograms for the entries in Table S15. For values vs SCE, see Table S15.

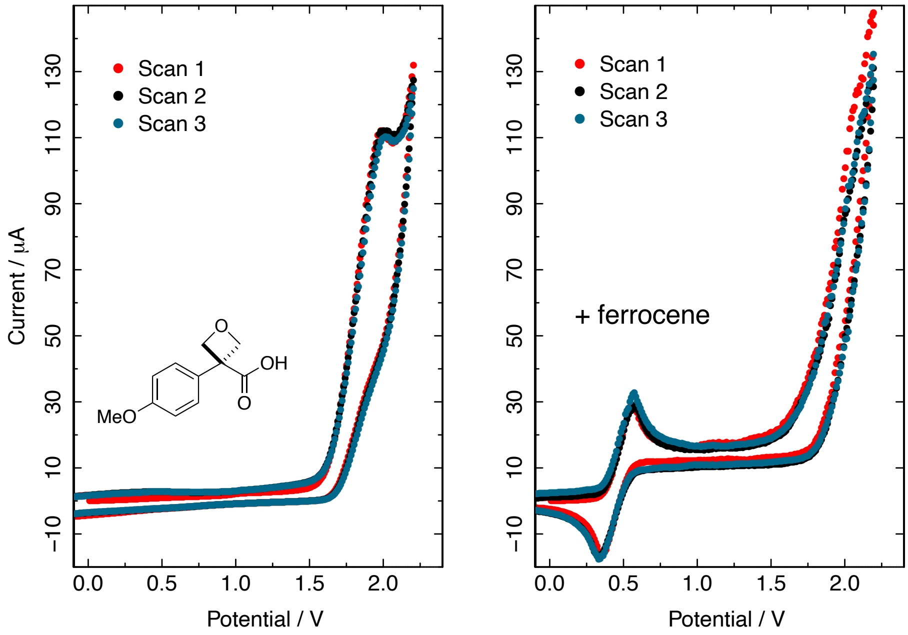


**Figure S13** Measured cyclic voltammogram of PMP-oxetane carboxylic acid **1** without (*left*) and with (*right*) ferrocene. Table S15 Entry 1.

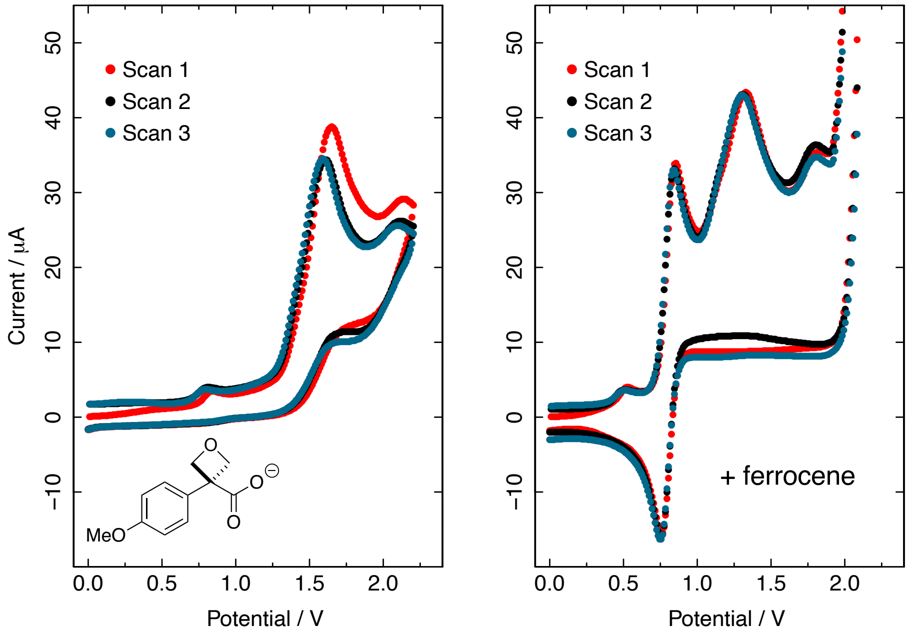


**Figure S14** Measured cyclic voltammogram of PMP-oxetane carboxylate **1**⁻ without (*left*) and with (*right*) ferrocene. Table S15 Entry 2.

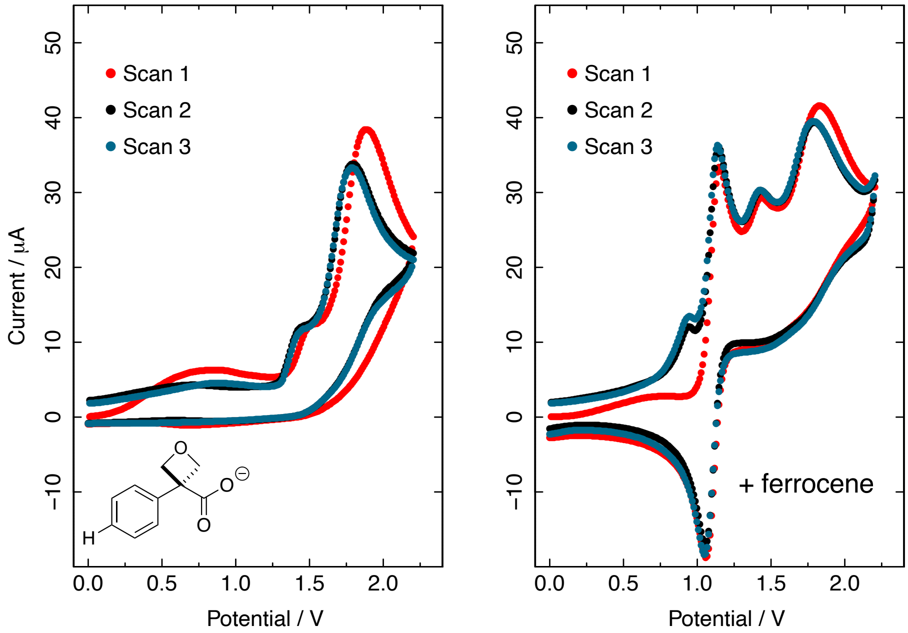


**Figure S15** Measured cyclic voltammogram of Ph-oxetane carboxylate **S10**⁻ without (*left*) and with (*right*) ferrocene. Table S15 Entry 3.

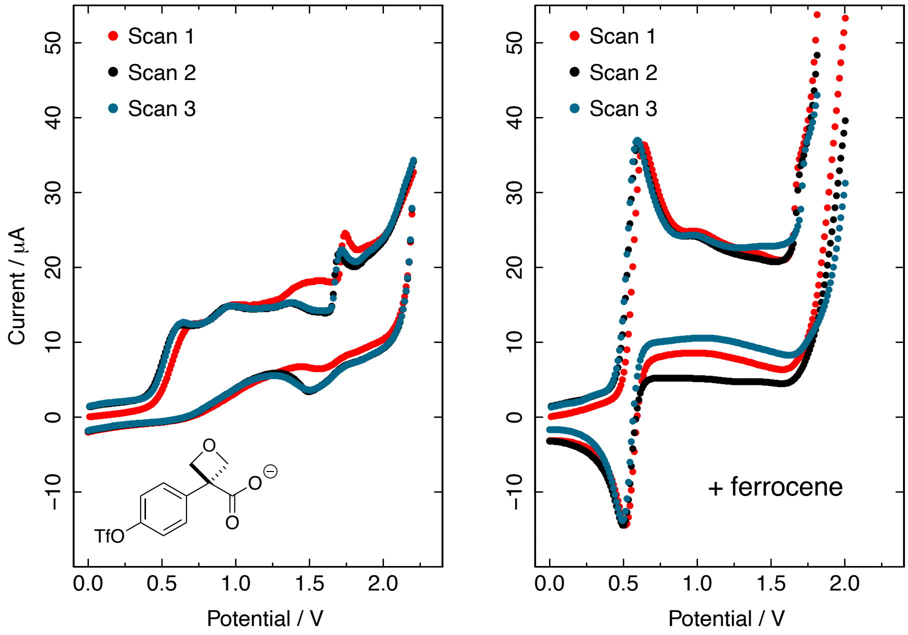


**Figure S16** Measured cyclic voltammogram of *p*-OTf-Ph-oxetane carboxylate without (*left*) and with (*right*) ferrocene. Table S15 Entry 4.

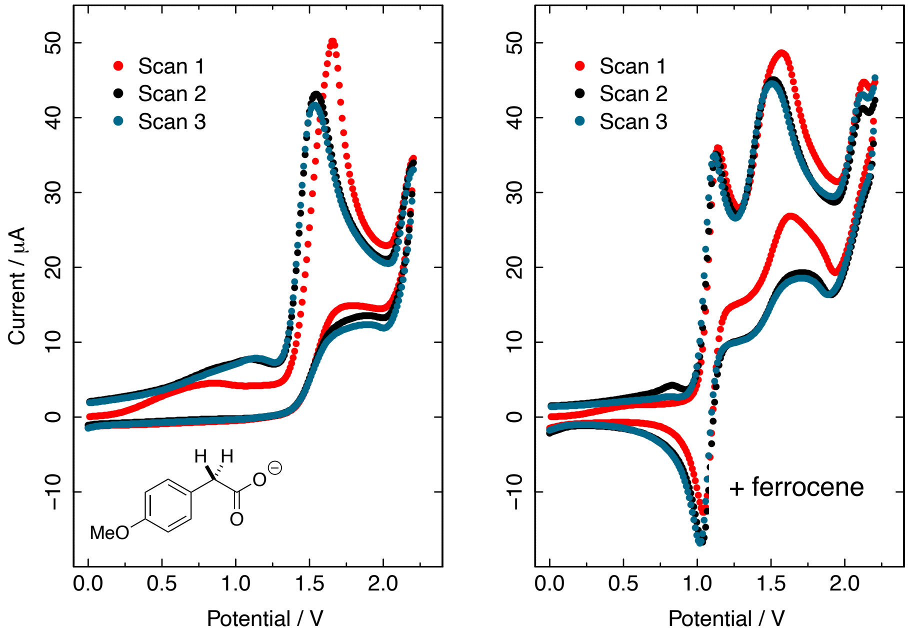


**Figure S17** Measured cyclic voltammogram of PMP-methylene carboxylate without (*left*) and with (*right*) ferrocene. Table S15 Entry 5.

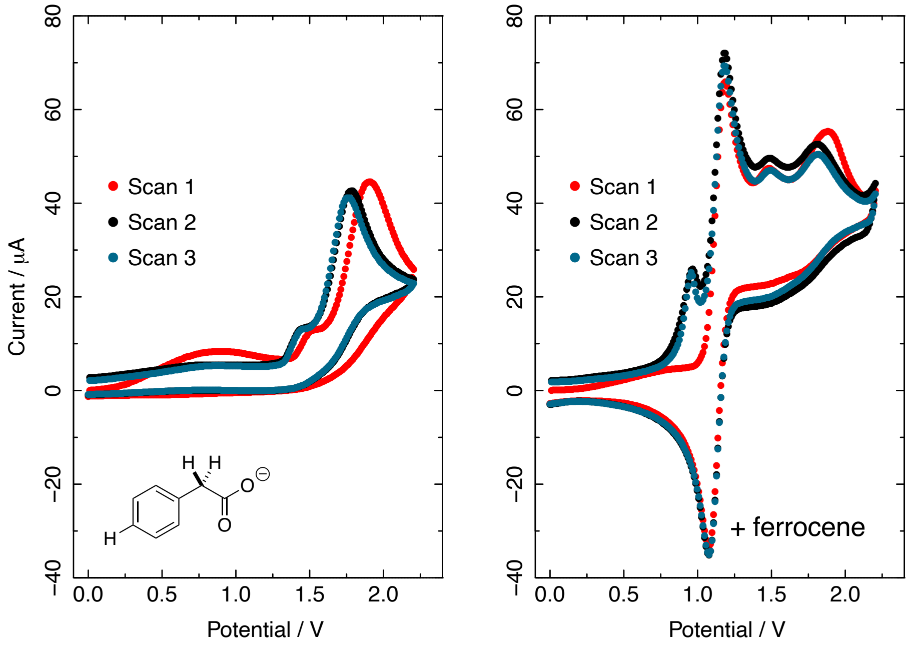


**Figure S18** Measured cyclic voltammogram of Ph-methylene carboxylate without (*left*) and with (*right*) ferrocene. Table S15 Entry 6.

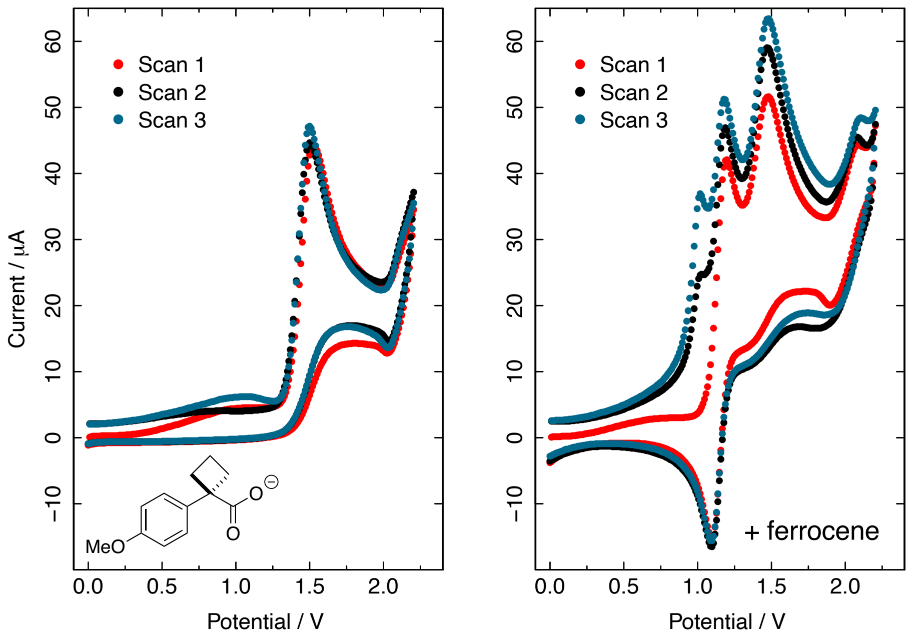


**Figure S19** Measured cyclic voltammogram of PMP-cyclobutane carboxylate without (*left*) and with (*right*) ferrocene. Table S15 Entry 7.

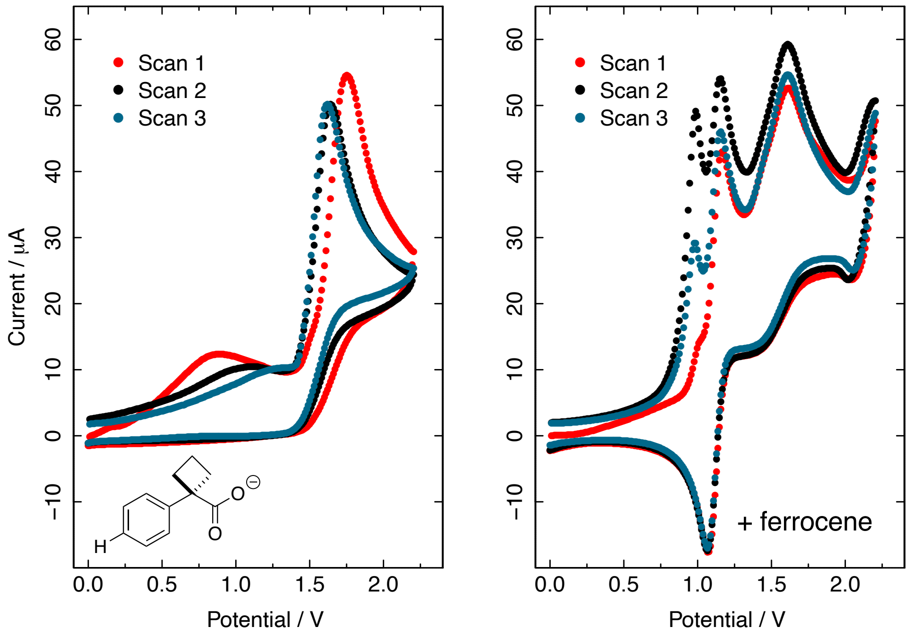


**Figure S20** Measured cyclic voltammogram of Ph-cyclobutane carboxylate without (*left*) and with (*right*) ferrocene. Table S15 Entry 8.

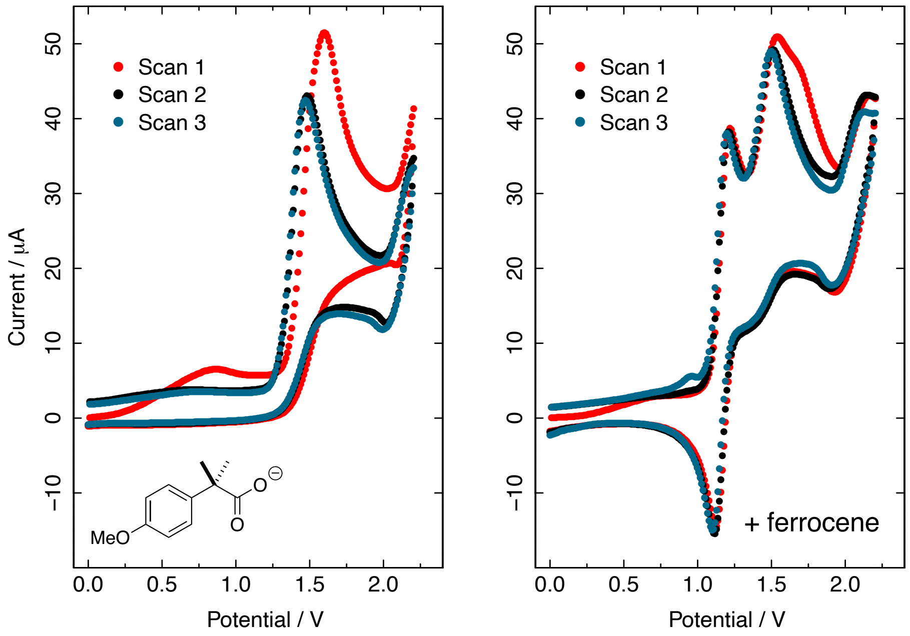


**Figure S21** Measured cyclic voltammogram of PMP-*gem*-dimethyl carboxylate without (*left*) and with (*right*) ferrocene. Table S15 Entry 9.

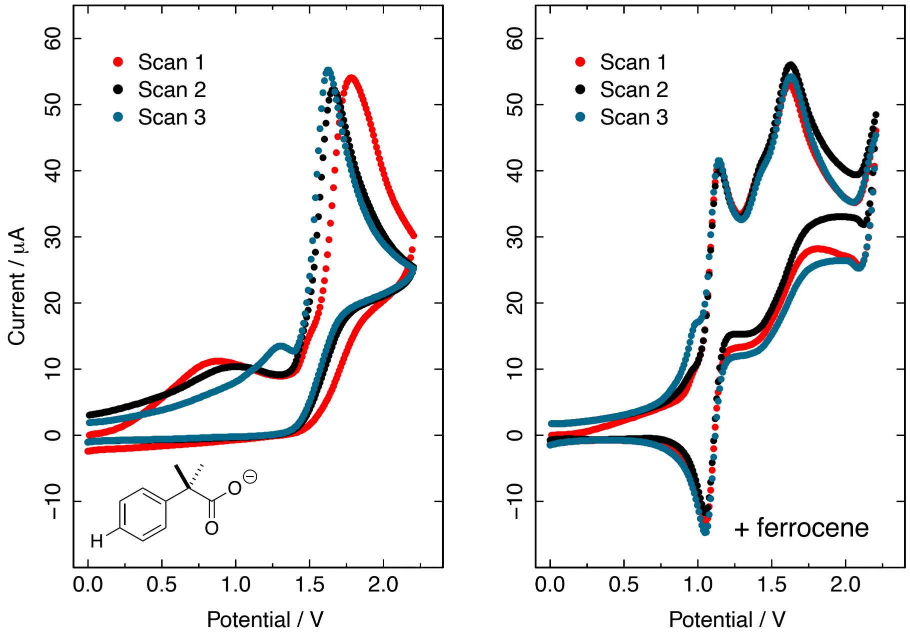


**Figure S22** Measured cyclic voltammogram of Ph-*gem*-dimethyl carboxylate without (*left*) and with (*right*) ferrocene. Table S15 Entry 10.

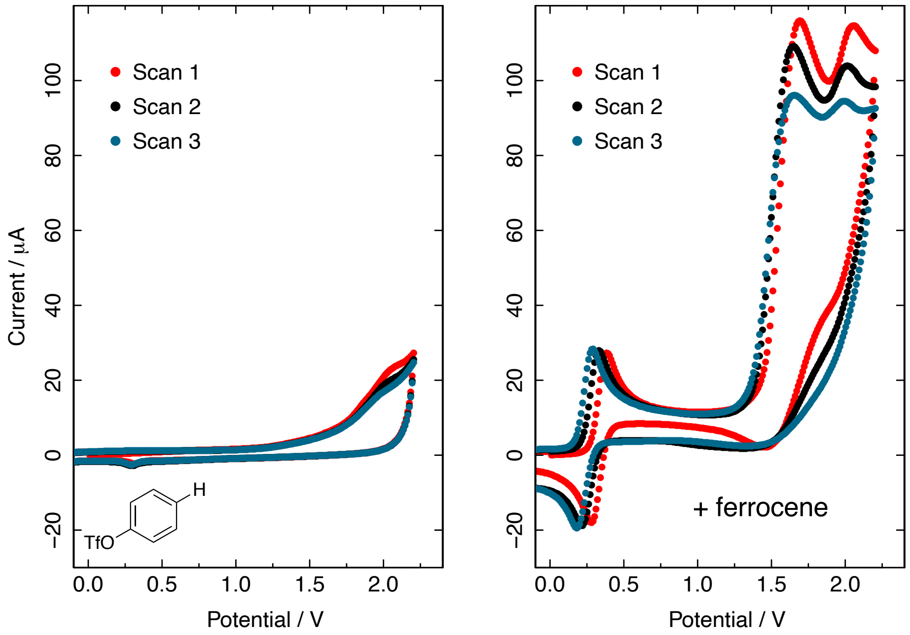


**Figure S23** Measured cyclic voltammogram of phenyl triflate without (*left*) and with (*right*) ferrocene. Table S15 Entry 11.

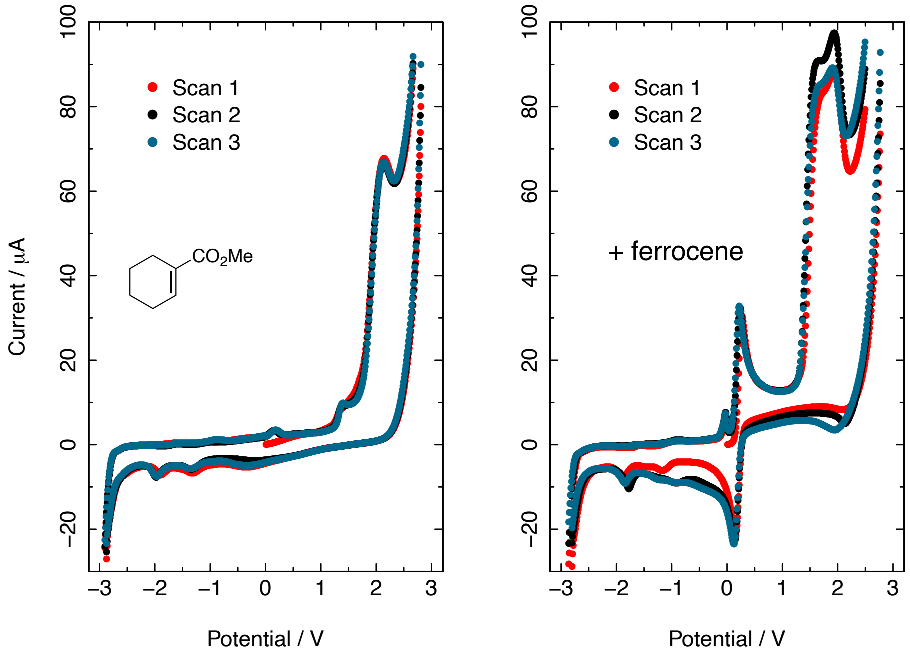


**Figure S24** Measured cyclic voltammogram of cyclohexene methyl carboxylate without (*left*) and with (*right*) ferrocene. Table S15 Entry 12.

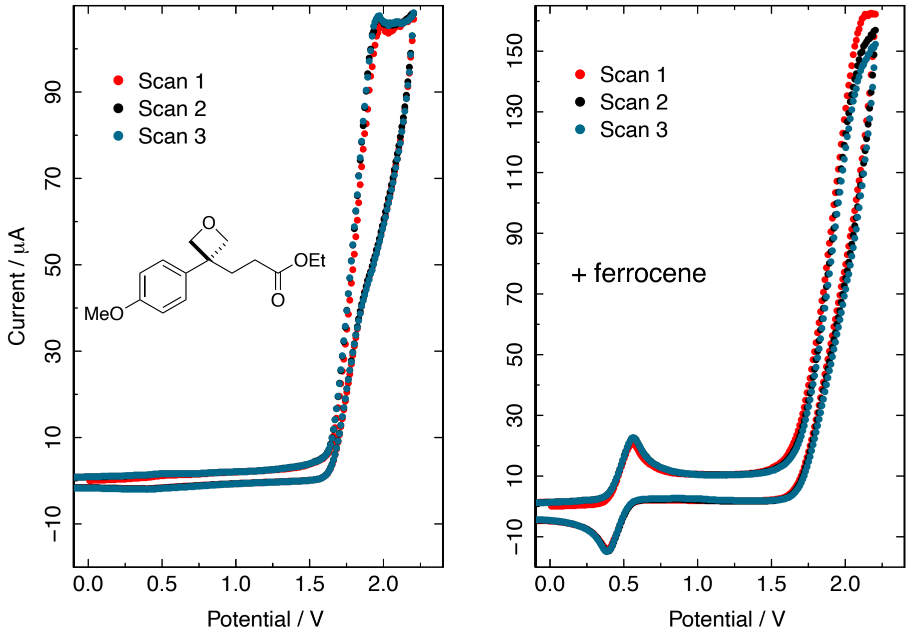


**Figure S25** Measured cyclic voltammogram of aryl-alkyl-oxetane **2a** without (*left*) and with (*right*) ferrocene. Table S15 Entry 13.

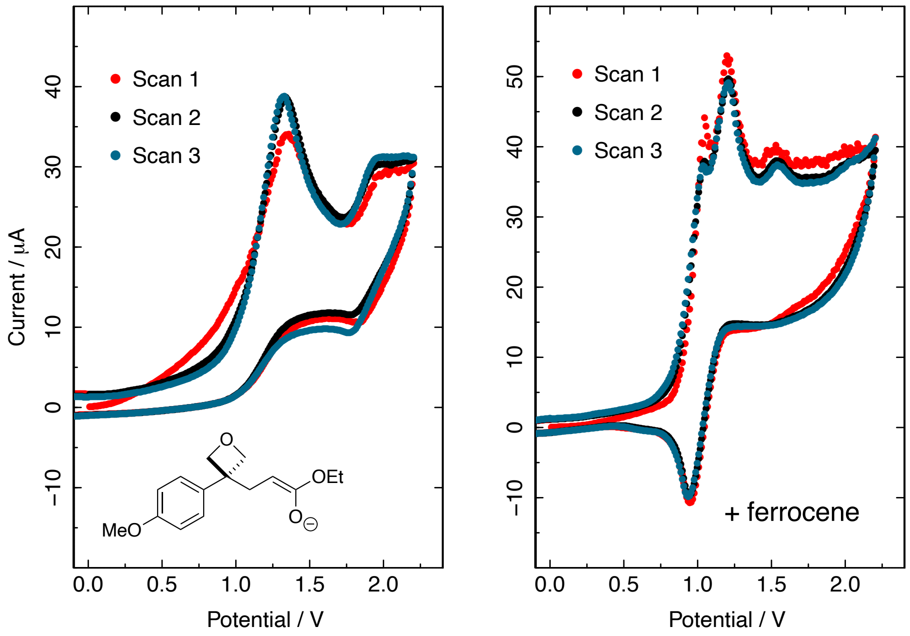


**Figure S26** Measured cyclic voltammogram of aryl-alkyl-oxetane enolate **2a**$^-$ without (*left*) and with (*right*) ferrocene. Table S15 Entry 14.

## Experimental Details and Characterization Data

PMP Oxetane Dimer **3** and Reduced Oxetane **4**

Dimer **3** and reduced oxetane **4** were isolated as side products on several occasions and characterized in the reaction with cyclopentenone (see page S50).

3-Aryl-3-Alkyl-Oxetanes (**2a–2t**, **7a–12a**, **S5**, **S6**)

**Ethyl 3-(3-(4-methoxyphenyl)oxetan-3-yl)propanoate (2a, III-A) and diethyl 2-((3-(4-methoxyphenyl)oxetan-3-yl)methyl)pentanedioate (2a', III-A')**

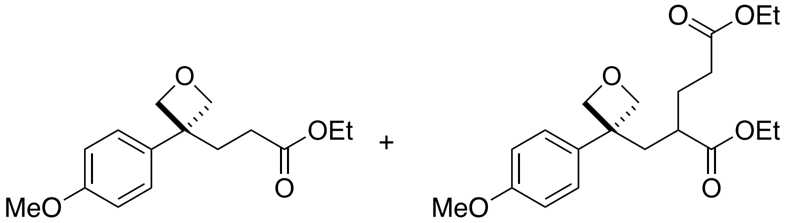


**2a**, **III-A** **2a'**, **III-A'**

Following **General Procedure A**, oxetane carboxylic acid **1** (41.6 mg) and ethyl acrylate (33 μL) were used. Purification by basic alumina (IV) column chromatography (20% $Et_2O$/pentane) afforded oxetane **2a** as a colorless oil (30.7 mg, 58%), followed by di addition product **2a'** as a colorless gum (5.8 mg, 8%; contains 5% of dimer **3**).

**2a** (**III-A**): $R_f$ = 0.33 (30% $Et_2O$/pentane); IR (film)/cm$^{-1}$ 2935, 1728 (C=O st), 1514, 1245, 1179, 1031, 981, 830; $^1$H NMR (400 MHz, $CDCl_3$) δ 6.97–6.93 (m, 2 H, 2 × Ar-CH), 6.90–6.87 (m, 2 H, 2 × Ar-CH), 4.95 (d, *J* = 5.7 Hz, 2 H, C*H*HOC*H*H), 4.64 (d, *J* = 5.7 Hz, 2 H, CH*H*OCH*H*), 4.06 (q, *J* = 7.1 Hz, 2 H, C*H*$_2$CH$_3$), 3.81 (s, 3 H, OCH$_3$), 2.43–2.38 (m, 2 H, C*H*$_2$CH$_2$), 2.14–2.10 (m, 2 H, CH$_2$C*H*$_2$), 1.22 (t, *J* = 7.1 Hz, 3 H, CH$_2$C*H*$_3$); $^{13}$C{$^1$H} NMR (101 MHz, $CDCl_3$) δ 173.1 ($C_q$=O), 158.1 (Ar-$C_q$OMe), 135.9 (Ar-*C*$_q$C$_q$), 126.8 (2 × Ar-CH), 114.0 (2 × Ar-CH), 81.5 (CH$_2$OCH$_2$), 60.4 (*C*H$_2$CH$_3$), 55.3 (OCH$_3$), 46.3 (C$_q$), 35.9 (*C*H$_2$CH$_2$), 29.9 (CH$_2$*C*H$_2$), 14.1 (CH$_2$*C*H$_3$); HRMS (ESI) *m/z*: [M+H]$^+$ Calcd for $C_{15}H_{21}O_4^+$ 265.1434; Found 265.1434.

**2a'** (**III-A'**): $R_f$ = 0.15 (50% $Et_2O$/pentane); IR (film)/cm$^{-1}$ 2937, 1730 (C=O st), 1513, 1249, 1182, 731; $^1$H NMR (400 MHz, $CDCl_3$) δ 7.00–6.96 (m, 2 H, 2 × Ar-CH), 6.90–6.86 (m, 2 H, 2 × Ar-CH), 4.95 (d, *J* = 5.7 Hz, 1 H, C*H*HO), 4.89 (d, *J* = 5.7 Hz, 1 H, C*H*HO), 4.64 (d, *J* = 5.7 Hz, 1 H, CH*H*O), 4.63 (d, *J* = 5.7 Hz, 1 H, CH*H*O), 4.08 (q, *J* = 7.1 Hz, 2 H, C*H*$_2$CH$_3$), 3.96–3.83 (m, 2 H, C*H*$_2$CH$_3$), 3.81 (s, 3 H, OCH$_3$), 2.66–2.60 (m, 1 H, C$_q$C*H*H), 2.21–2.16 (m, 2 H, CHCH$_2$C*H*$_2$), 2.15–2.11 (m, 2 H, CH + C$_q$CH*H*), 1.90–1.80 (m, 1 H, CHC*H*HCH$_2$), 1.74–1.65 (m, 1 H, CHCH*H*CH$_2$), 1.22 (t, *J* = 7.1 Hz, 3 H, CH$_2$C*H*$_3$), 1.18 (t, *J* = 7.1 Hz, 3 H, CH$_2$C*H*$_3$); $^{13}$C{$^1$H} NMR (101 MHz, $CDCl_3$) δ 175.1 (C$_q$=O), 172.6 (C$_q$=O), 158.1 (Ar-C$_q$OMe), 135.8 (Ar-*C*$_q$C$_q$), 127.1 (2 × Ar-CH), 113.8 (2 × Ar-CH), 82.2 (*C*H$_2$OCH$_2$), 81.8 (CH$_2$O*C*H$_2$), 60.5 (*C*H$_2$CH$_3$), 60.4 (*C*H$_2$CH$_3$), 55.3 (OCH$_3$), 46.5 (C$_q$), 43.3 (C$_q$*C*H$_2$), 41.3 (CH), 31.7 (CHCH$_2$*C*H$_2$), 28.6 (CH*C*H$_2$CH$_2$), 14.1 (CH$_2$*C*H$_3$), 14.0 (CH$_2$*C*H$_3$); HRMS (ESI) *m/z*: [M+H]$^+$ Calcd for $C_{20}H_{29}O_6$ 365.1964; Found 365.1967.

Notes:

***2a** was obtained in 67% yield (35.2 mg) when using repurified oxetane acid **1** (99.9% instead of 98.9% purity; see page S15 for details).*

*On some occasions, **2a** crystallized to form colorless crystals (mp = 57 °C) and was further characterized by X-ray crystallography (Figures S27–S28).*

***2a** was also obtained in 58% yield on a 1.0 mmol scale by using a 12 mL vial (purchased from Agilent, product code 5183-4332):*

An oven-dried 12 mL vial was charged with oxetane carboxylic acid **1** (208.3 mg, 1.0 mmol, 1.0 equiv), oven-dried $Cs_2CO_3$ (391 mg, 1.2 mmol, 1.2 equiv) and [Ir{dF(CF$_3$)ppy}$_2$(dtbbpy)]PF$_6$

(11.0 mg, 0.01 mmol, 1.0 mol%). The vial was sealed with a screwcap equipped with a PTFE/silicon septum and anhydrous DMF (5.0 mL, 0.2 M) was added by syringe. Argon was bubbled through the mixture for 5 min and ethyl acrylate (164 μL, 1.5 mmol, 1.5 equiv) was added by syringe under argon. After sealing the cap with parafilm on top, the reaction mixture was stirred at 1000 rpm using the set-up shown in Figure S2 and irradiated with two 467 nm Kessel lamps at 36 °C (heat generated by the lamps). After 40 h the lights were switched off and the reaction mixture was transferred into a separating funnel. Distilled water (30 mL) and $Et_2O$ (30 mL) were added, the layers were separated and the aqueous portion was extracted with $Et_2O$ (2 × 30 mL). The organic extracts were combined, dried over $Na_2SO_4$, filtered and concentrated *in vacuo* using a rotatory evaporator. Purification by basic alumina (IV) column chromatography (20–40% $Et_2O$/pentane) afforded oxetane **2a** as a colorless oil (153 mg, 58%) followed by oxetane **2a'** as a colorless oil (20.6 mg, 6%; containing 15% of dimer **3**).

**tert-Butyl 3-(3-(4-methoxyphenyl)oxetan-3-yl)propanoate (2b)**

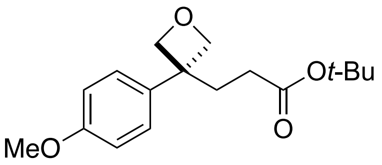


Following **General Procedure A**, oxetane carboxylic acid **1** (41.6 mg) and *t*-Bu-acrylate (45 μL) were used. Purification by basic alumina (IV) column chromatography (20% $Et_2O$/pentane) afforded oxetane **2b** as a white solid (31.9 mg, 55%). $R_f$ = 0.35 (30% $Et_2O$/pentane); mp = 90–91 °C; IR (film)/$cm^{-1}$ 2933, 1722 (C=O st), 1513, 1245, 1148, 980, 842; $^1H$ NMR (400 MHz, $CDCl_3$) δ 6.97–6.94 (m, 2 H, 2 × Ar-CH), 6.91–6.87 (m, 2 H, 2 × Ar-CH), 4.94 (d, *J* = 5.7 Hz, 2 H, C*H*HOC*H*H), 4.65 (d, *J* = 5.7 Hz, 2 H, CH*H*OCH*H*), 3.81 (s, 3 H, $OCH_3$), 2.37–2.33 (m, 2 H, $CH_2CH_2$), 2.06–2.02 (m, 2 H, $CH_2CH_2$), 1.41 (s, 9 H, 3 × $CH_3$); $^{13}C\{^1H\}$ NMR (101 MHz, $CDCl_3$) δ 172.4 ($C_q$=O), 158.1 (Ar-$C_q$OMe), 136.1 (Ar-$C_qC_q$), 126.8 (2 × Ar-CH), 113.9 (2 × Ar-CH), 81.6 ($CH_2OCH_2$), 80.4 ($C_q(CH_3)_3$), 55.3 ($OCH_3$), 46.2 ($C_q$), 36.0 ($CH_2CH_2$), 31.0 ($CH_2CH_2$), 28.0 ($C_q(CH_3)_3$); HRMS (APCI) *m/z*: $[M+Na]^+$ Calcd for $C_{17}H_{24}NaO_4^+$ 315.1567; Found 315.1553.

**1-(3-(4-Methoxyphenyl)oxetan-3-yl)octan-3-one (2c)**

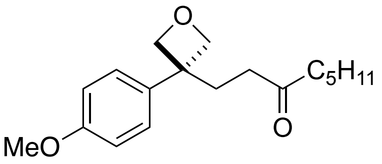


Following **General Procedure A**, oxetane carboxylic acid **1** (41.7 mg) and 1-octen-3-one (45 μL) were used. Purification by silica column chromatography (10–20% EtOAc/hexane) afforded oxetane **2c** as a colorless oil (13.0 mg, 22%), followed by oxetane dimer **3** as a colorless oil (3.0 mg, 9%) and reduced oxetane **4** as a colorless oil (2.0 mg, 7%). $R_f$ = 0.33 (20% EtOAc/hexane); IR (film)/$cm^{-1}$ 2930, 1715 (C=O st), 1513, 1245, 828; $^1H$ NMR (400 MHz, $CDCl_3$) δ 6.97–6.94 (m, 2 H, 2 × Ar-CH), 6.91–6.88 (m, 2 H, 2 × Ar-CH), 4.94 (d, *J* = 5.7 Hz, 2 H, C*H*HOC*H*H), 4.63 (d, *J* = 5.7 Hz, 2 H, CH*H*OCH*H*), 3.81 (s, 3 H, $OCH_3$), 2.35–2.29 (m, 4 H, $CH_2C{=}OCH_2$), 2.26–2.21 (m, 2 H, $C_qCH_2CH_2$), 1.50 (p, *J* = 7.4 Hz, 2 H, $C{=}OCH_2CH_2$), 1.31–1.16 (m, 4 H, $CH_2CH_2CH_3$), 0.87 (t, *J* = 7.1 Hz, 3 H, $CH_3$). HRMS (ES) *m/z*: $[M+H]^+$ Calcd for $C_{18}H_{27}O_3$ 291.1963; Found 291.1960.

Notes:

*The compound degraded in $CDCl_3$ before $^{13}C$ NMR data could be collected.*

**3-(4-Methoxyphenyl)-3-(2-(phenylsulfonyl)ethyl)oxetane (2d)**

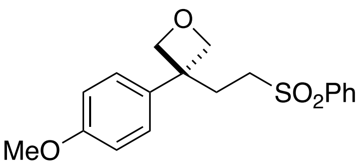


Following **General Procedure B**, oxetane carboxylic acid **1** (41.6 mg) and phenyl vinyl sulfone (50.5 mg) were used. Purification by basic alumina (IV) column chromatography (2% $Et_2O/CH_2Cl_2$) afforded oxetane **2d** as a colorless oil (17.9 mg, 27%). $R_f$ = 0.31 (4% $Et_2O/CH_2Cl_2$); IR (film)/$cm^{-1}$ 2937, 1513, 1305 and 1248 ($SO_2$ st), 1144; $^1H$ NMR (400 MHz, $CDCl_3$) δ 7.88–7.85 (m, 2 H, 2 × Ar-CH), 7.70–7.64 (m, 1 H, Ar-CH), 7.59–7.55 (m, 2 H, 2 × Ar-CH), 6.87 (s, 4 H, 4 × Ar-CH), 4.92 (d, *J* = 5.9 Hz, 2 H, C*H*HOC*H*H), 4.54 (d, *J* = 5.9 Hz, 2 H, CH*H*OCH*H*), 3.81 (s, 3 H, $OCH_3$), 2.92–2.88 (m, 2 H, $CH_2CH_2$), 2.48–2.44 (m, 2 H, $CH_2CH_2$); $^{13}C\{^1H\}$ NMR (101 MHz, $CDCl_3$) δ 158.4 (Ar-$C_q$OMe), 134.5 (Ar-$C_qC_q$), 133.8 (Ar-CH),

129.4 (2 × Ar-CH), 128.4 (Ar-$C_qSO_2$), 128.0 (2 × Ar-CH), 126.6 (2 × Ar-CH), 114.3 (2 × Ar-CH), 81.0 ($CH_2OCH_2$), 55.3 ($OCH_3$), 52.2 ($CH_2CH_2SO_2$), 45.7 ($C_q$), 33.3 ($CH_2CH_2SO_2$); HRMS (ESI) *m/z*: $[M+H]^+$ Calcd for $C_{18}H_{21}O_4S$ 333.1161; Found 333.1162.

**Diethyl (2-(3-(4-methoxyphenyl)oxetan-3-yl)ethyl)phosphonate (2e)**

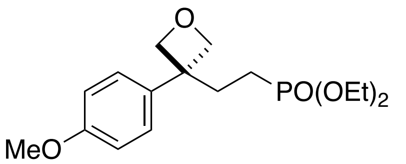


Following **General Procedure A**, oxetane carboxylic acid **1** (41.6 mg) and diethyl vinylphosphonate (46 μL) were used. Purification by basic alumina (IV) column chromatography (100% EtOAc) afforded oxetane **2e** as a colorless oil (10.8 mg, 16%). $R_f$ = 0.12 (100% EtOAc); IR (film)/cm$^{-1}$ 2933, 1513, 1245 (P=O st), 1029, 965; $^1$H NMR (400 MHz, $CDCl_3$) δ 6.95–6.91 (m, 2 H, 2 × Ar-CH), 6.90–6.86 (m, 2 H, 2 × Ar-CH), 4.94 (d, *J* = 5.7 Hz, 2 H, C*H*HOC*H*H), 4.61 (d, *J* = 5.7 Hz, 2 H, CH*H*OCH*H*), 4.10–4.00 (m, 4 H, 2 × C*H*$_2$CH$_3$), 3.81 (s, 3 H, $OCH_3$), 2.36–2.30 (m, 2 H, C*H*$_2$CH$_2$), 1.57–1.48 (m, 2 H, CH$_2$C*H*$_2$), 1.29 (t, *J* = 7.1 Hz, 6 H, 2 × CH$_2$C*H*$_3$); $^{13}C\{^1H\}$ NMR (101 MHz, $CDCl_3$) δ 158.2 (Ar-$C_q$OMe), 135.5 (Ar-*C*$_q$C$_q$), 126.8 (2 × Ar-CH), 114.0 (2 × Ar-CH), 81.2 ($CH_2OCH_2$), 61.6 (d, $^2J_{C-P}$ = 6.4 Hz, 2 × *C*H$_2$CH$_3$), 55.3 ($OCH_3$), 46.7 (d, $^3J_{C-P}$ = 18.4 Hz, $C_q$), 33.6 (d, $^2J_{C-P}$ = 4.0 Hz, *C*H$_2$CH$_2$P), 21.1 (d, $^1J_{C-P}$ = 142.6 Hz, CH$_2$*C*H$_2$P), 16.4 (d, $^3J_{C-P}$ = 5.7 Hz, 2 × CH$_2$*C*H$_3$); $^{31}P\{^1H\}$ NMR (162 MHz, $CDCl_3$) δ 31.7; HRMS (ESI) *m/z*: $[M+H]^+$ Calcd for $C_{16}H_{26}O_5P$ 329.1518; Found 329.1523.

**3-(3-(4-Methoxyphenyl)oxetan-3-yl)propanenitrile (2f)**

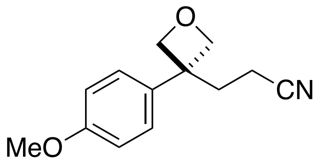


Following **General Procedure A**, oxetane carboxylic acid **1** (41.6 mg) and acrylonitrile (20 μL) were used. Purification by basic alumina (IV) column chromatography (30% $Et_2O$/pentane) afforded oxetane **2f** as a colorless oil (4.8 mg, containing 6% BHT, 11%). $R_f$ = 0.17 (50% $Et_2O$/pentane); IR (film)/cm$^{-1}$ 2937, 2233 (C≡N st), 1610, 1513, 1248, 1181, 1028, 984, 834; $^1$H NMR (400 MHz, $CDCl_3$) δ 6.98–6.94 (m, 2 H, 2 × Ar-CH), 6.93–6.89 (m, 2 H, 2 × Ar-CH), 5.00 (d, *J* = 5.9 Hz, 2 H, C*H*HOC*H*H), 4.72 (d, *J* = 5.9 Hz, 2 H, CH*H*OCH*H*), 3.83 (s, 3 H, $OCH_3$), 2.46–2.42 (m, 2 H, C*H*$_2$CH$_2$), 2.12–2.08 (m, 2 H, CH$_2$C*H*$_2$); $^{13}C\{^1H\}$ NMR (101 MHz, $CDCl_3$) δ 158.6 (Ar-$C_q$OMe), 134.2 (Ar-*C*$_q$C$_q$), 126.7 (2 × Ar-CH), 119.1 (CN), 114.4 (2 × Ar-CH), 81.1 ($CH_2OCH_2$), 55.3 ($OCH_3$), 46.4 ($C_q$), 36.2 (*C*H$_2$CH$_2$), 13.0 (CH$_2$*C*H$_2$); HRMS (EI) *m/z*: $[M]^{\bullet+}$ Calcd for $C_{13}H_{15}NO_2^{\bullet+}$ 217.1097; Found 217.1106.

**3-(3-(4-Methoxyphenyl)oxetan-3-yl)-*N*-phenylpropanamide (2g)**

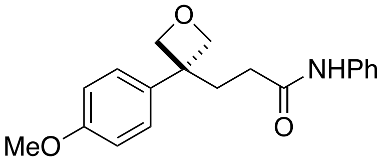


Following **General Procedure B**, oxetane carboxylic acid **1** (41.6 mg) and *N*-phenylacrylamide (44.2 mg) were used. Purification by basic alumina (IV) column chromatography (50–80% $Et_2O$/pentane) afforded oxetane **2g** as a colorless gum (42.5 mg, 68%). $R_f$ = 0.11 (20% $Et_2O$/pentane); IR (film)/cm$^{-1}$ 3306 (NH st, br), 2939, 1665 (C=O st), 1605, 1545, 1510, 1443, 1244, 1184, 980, 757; $^1$H NMR (400 MHz, $CDCl_3$) δ 7.45–7.42 (m, 2 H, 2 × Ar-CH), 7.32–7.28 (m, 2 H, 2 × Ar-CH), 7.17 (br s, 1 H, NH), 7.11–7.08 (m, 1 H, Ph-CH), 7.00–6.97 (m, 2 H, 2 × Ar-CH), 6.91–6.89 (m, 2 H, 2 × Ar-CH), 4.96 (d, *J* = 5.7 Hz, 2 H, C*H*HOC*H*H), 4.68 (d, *J* = 5.7 Hz, 2 H, CH*H*OCH*H*), 3.80 (s, 3 H, $OCH_3$), 2.52–2.48 (m, 2 H, C*H*$_2$CH$_2$), 2.19–2.15 (m, 2 H, CH$_2$C*H*$_2$); $^{13}C\{^1H\}$ NMR (101 MHz, $CDCl_3$) δ 170.4 ($C_q$=O), 158.2 (Ar-$C_q$OMe), 137.7 (Ar-$C_q$), 136.0 (Ar-$C_q$), 129.0 (2 × Ar-CH), 126.8 (2 × Ar-CH), 124.2 (Ph-CH), 119.7 (2 × Ar-CH), 114.0 (2 × Ar-CH), 81.5 ($CH_2OCH_2$), 55.3 ($OCH_3$), 46.3 ($C_q$), 36.3 (*C*H$_2$CH$_2$), 33.0 (CH$_2$*C*H$_2$); HRMS (EI) *m/z*: $[M+H]^+$ Calcd for $C_{19}H_{22}NO_3^+$ 312.1594; Found 312.1595.

**N-(*tert*-Butyl)-3-(3-(4-methoxyphenyl)oxetan-3-yl)propanamide (2h)**

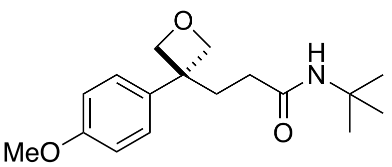

Following **General Procedure B**, oxetane carboxylic acid **1** (41.6 mg) and *N*-(*tert*-butyl)acrylamide (38.2 mg) were used. Purification by basic alumina (IV) column chromatography (70–80% $Et_2O$/pentane) afforded oxetane **2h** as a white solid (21.9 mg, 38%). $R_f$ = 0.17 (80% $Et_2O$/pentane); mp = 69–70 °C; IR (film)/cm$^{-1}$ 3313 (NH st, br), 2958, 1646 (C=O st), 1512, 1246, 1031, 980; $^1$H NMR (400 MHz, $CDCl_3$) δ 6.98–6.95 (m, 2 H, 2 × Ar-CH), 6.90–6.87 (m, 2 H, 2 × Ar-CH), 5.15 (br s, 1 H, NH), 4.93 (d, *J* = 5.7 Hz, 2 H, C*H*HOC*H*H), 4.65 (d, *J* = 5.7 Hz, 2 H, CH*H*OCH*H*), 3.81 (s, 3 H, $OCH_3$), 2.39–2.35 (m, 2 H, $CH_2CH_2$), 1.92–1.88 (m, 2 H, $CH_2CH_2$), 1.30 (s, 9 H, 3 × $CH_3$); $^{13}C\{^1H\}$ NMR (101 MHz, $CDCl_3$) δ 171.4 ($C_q$=O), 158.1 (Ar-$C_q$OMe), 136.3 (Ar-$C_qC_q$), 126.8 (2 × Ar-CH), 113.9 (2 × Ar-CH), 81.6 ($CH_2OCH_2$), 55.3 ($OCH_3$), 46.3 ($C_q$), 36.5 ($CH_2CH_2$), 32.8 ($CH_2CH_2$), 30.3 ($C_q(CH_3)_3$), 28.7 ($C_q(CH_3)_3$); HRMS (ESI) *m/z*: $[M+H]^+$ Calcd for $C_{17}H_{26}NO_3$ 292.1913; Found 292.1908.

**3-(3-(4-Methoxyphenyl)oxetan-3-yl)-1-morpholinopropan-1-one (2i)**

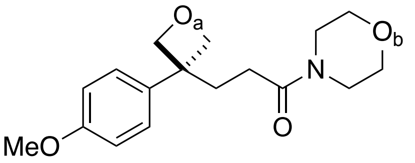

Following **General Procedure A**, oxetane carboxylic acid **1** (41.6 mg) and 1-morpholinoprop-2-en-1-one (38 μL) were used. Purification by silica column chromatography (30–50% acetone/pentane) afforded oxetane **2i** as a colorless gum (32.6 mg, 53%). $R_f$ = 0.10 (30% acetone/pentane); IR (film)/cm$^{-1}$ 2858, 1636 (C=O st), 1512, 1432, 1245, 1113, 1029, 979, 832; $^1$H NMR (400 MHz, $CDCl_3$) δ 7.00–6.96 (m, 2 H, 2 × Ar-CH), 6.90–6.87 (m, 2 H, 2 × Ar-CH), 4.94 (d, *J* = 5.7 Hz, 2 H, C*H*H$O_a$C*H*H), 4.66 (d, *J* = 5.7 Hz, 2 H, CH*H*$O_a$CH*H*), 3.81 (s, 3 H, $OCH_3$), 3.64–3.61 (m, 2 H, C*H*H$O_b$C*H*H), 3.59–3.55 (m, 4 H, CH*H*$O_b$CH*H* + C*H*HNC*H*H), 3.26 (br t, *J* = 4.9 Hz, 2 H, CH*H*NCH*H*), 2.43–2.38 (m, 2 H, $CH_2CH_2$), 2.12–2.08 (m, 2 H, $CH_2CH_2$); $^{13}C\{^1H\}$ NMR (101 MHz, $CDCl_3$) δ 170.9 ($C_q$=O), 158.1 (Ar-$C_q$OMe), 136.1 (Ar-$C_qC_q$), 126.7 (2 × Ar-CH), 114.0 (2 × Ar-CH), 81.5 ($CH_2O_aCH_2$), 66.8 ($CH_2O_bCH_2$), 66.4 ($CH_2O_bCH_2$), 55.2 ($OCH_3$), 46.2 ($C_q$), 45.7 ($CH_2NCH_2$), 41.8 ($CH_2NCH_2$), 36.0 ($CH_2CH_2$), 28.2 ($CH_2CH_2$); HRMS (ESI) *m/z*: $[M+H]^+$ Calcd for $C_{17}H_{24}NO_4$ 306.1705; Found 306.1691.

Notes:

*This compound slowly degraded on basic alumina (IV), but was still isolated in 51% yield (31.2 mg) when purified by basic alumina (IV) column chromatography.*

**Methyl 2-((*tert*-butoxycarbonyl)amino)-3-(3-(4-methoxyphenyl)oxetan-3-yl)propanoate (2j)**

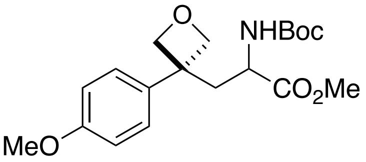

Following **General Procedure A**, oxetane carboxylic acid **1** (41.6 mg) and methyl 2-((*tert*-butoxycarbonyl)amino)acrylate (60.4 mg) were used. Purification by basic alumina (IV) column chromatography (10–60% $Et_2O$/pentane) afforded oxetane **2j** as a colorless gum which was crystallized by slow evaporation using acetone to form colorless crystals (44.2 mg, 60%). $R_f$ = 0.31 (80% $Et_2O$/pentane); mp = 103–106 °C; IR (film)/cm$^{-1}$ 3355 (NH st, br), 2956, 1720 (C=O st), 1715 (C=O st), 1513, 1252, 1163; $^1$H NMR (400 MHz, $CDCl_3$) δ 7.08–7.04 (m, 2 H, 2 × Ar-CH), 6.92–6.89 (m, 2 H, 2 × Ar-CH), 4.96–4.93 (m 2 H, C*H*HOC*H*H), 4.75–4.68 (br s, 1 H, NH), 4.75 (d, *J* = 5.7 Hz, 1 H, CH*H*$OCH_2$), 4.68 (d, *J* = 5.7 Hz, 1 H, $CH_2$OCH*H*), 4.05–4.04 (br m, 1 H, CH), 3.81 (s, 3 H, Ar-$C_qOCH_3$), 3.60 (s, 3 H, $CO_2CH_3$), 2.60 (br dd, $^2J$ = 14.1 Hz, $^3J$ = 4.0 Hz, 1 H, C*H*HCH), 2.47 (dd, $^2J$ = 14.1 Hz, $^3J$ = 8.1 Hz, 1 H, CH*H*CH), 1.40 (s, 9 H, $C_q(CH_3)_3$); $^{13}C\{^1H\}$ NMR (101 MHz, $CDCl_3$) δ 172.8 ($C_q=O_{ester}$), 158.2 (Ar-$C_q$OMe), 154.8 ($C_q=O_{carbamate}$), 135.2 (Ar-$C_qC_q$), 127.0 (2 × Ar-CH), 114.0 (2 × Ar-CH), 82.1 ($CH_2OCH_2$), 81.9 ($CH_2OCH_2$), 79.9 ($C_q(CH_3)_3$), 55.2 (Ar-$C_qOCH_3$), 52.3 ($CO_2CH_3$), 51.1 (CH), 45.8 ($C_q$), 42.8 ($CH_2$CH), 28.2 ($C_q(CH_3)_3$); HRMS (APCI) *m/z*: $[M+H]^+$ Calcd for $C_{19}H_{28}NO_6^+$ 366.1911; Found 366.1912.

Notes:

***2j** was further characterized by X-ray crystallography (see Figures S29–S30).*

*The NH, CH**H**OCH2 and CH2OCH**H** signals in the [1]H NMR spectrum were integrated together to 3 H.*

**3-(3-(4-Methoxyphenyl)oxetan-3-yl)-2-methylpropanal (2k)**

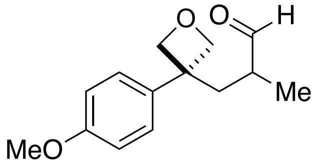

Following **General Procedure A**, oxetane carboxylic acid **1** (41.6 mg) and methacrolein (25 μL) were used. Purification by basic alumina (IV) column chromatography (30–70% $Et_2O$/pentane) afforded oxetane **2k** as a colorless oil (15.1 mg, 32%). $R_f$ = 0.15 (30% $Et_2O$/pentane); IR (film)/cm$^{-1}$ 2933, 1722 (C=O st), 1513, 1249, 1181, 835; $^1$H NMR (400 MHz, $CDCl_3$) δ 9.30 (d, *J* = 2.0 Hz, 1 H, HC=O), 7.00–6.96 (m, 2 H, 2 × Ar-CH), 6.91–6.88 (m, 2 H, 2 × Ar-CH), 5.01 (dd, *J* = 5.8, 0.8 Hz, 1 H, C*H*HOCH2), 4.93 (d, *J* = 5.8 Hz, 1 H, CH2OC*H*H), 4.68 (d, *J* = 5.8 Hz, 2 H, CH*H*OCH*H*), 3.81 (s, 3 H, OCH3), 2.62–2.56 (m, 1 H, CH3C*H*), 2.16–2.08 (m, 2 H, C*H*2CH), 1.00 (d, *J* = 6.9 Hz, 3 H, C*H*3CH); $^{13}$C{$^1$H} NMR (101 MHz, $CDCl_3$) δ 203.7 (C=O), 158.3 (Ar-*C*qOMe), 135.8 (Ar-*C*qCq), 126.9 (2 × Ar-CH), 114.1 (2 × Ar-CH), 82.6 (*C*H2OCH2), 81.7 (CH2O*C*H2), 55.2 (OCH3), 46.4 (Cq), 43.5 (CH), 42.0 (*C*H2CH), 14.8 (CH3); HRMS (ESI) *m/z*: [M+H]$^+$ Calcd for $C_{14}H_{19}O_3$ 235.1334; Found 235.1327.

**3-(3-(4-Methoxyphenyl)oxetan-3-yl)cyclopentan-1-one (2l), 3,3'-bis(4-methoxyphenyl)-3,3'-bioxetane (3)** and **3-(4-methoxyphenyl)oxetane (4)**

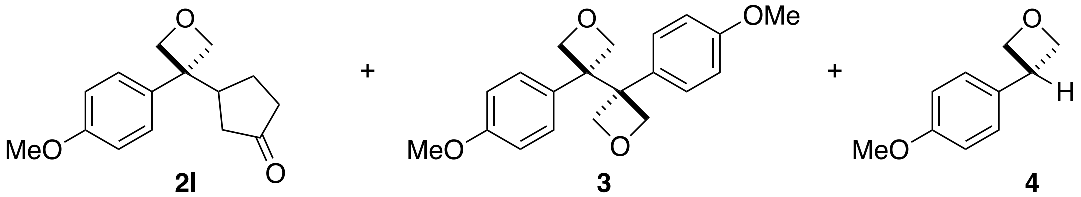

**2l** **3** **4**

Following **General Procedure A**, oxetane carboxylic acid **1** (41.6 mg) and 2-cyclopenten-1-one (25 μL) were used. Purification by basic alumina (IV) column chromatography (40–60% $Et_2O$/pentane) afforded oxetane **4** as a colorless oil (2.5 mg, 7%), followed by dimer **3** as a white film (3.7 mg, 11%, isolated with 7% BHT) and oxetane **2l** as a white solid (12.4 mg, 25%).

Oxetane **4**: $R_f$ = 0.53 (40% $Et_2O$/pentane); IR (film)/cm$^{-1}$ 2933, 1610, 1513, 1461, 1248, 1181, 1028, 834; $^1$H NMR (400 MHz, $CDCl_3$) δ 7.36–7.32 (m, 2 H, 2 × Ar-CH), 6.94–6.90 (m, 2 H, 2 × Ar-CH), 5.06 (dd, *J* = 8.4, 5.9 Hz, 2 H, C*H*HOC*H*H), 4.76 (dd, *J* = 6.9, 5.9 Hz, 2 H, CH*H*OCH*H*), 4.24–4.16 (m, 1 H, CH), 3.82 (s, 3 H, OCH3); $^{13}$C{$^1$H} NMR (101 MHz, $CDCl_3$) δ 158.6 (Ar-CqOMe), 133.6 (Ar-*C*qCq), 127.8 (2 × Ar-CH), 114.1 (2 × Ar-CH), 79.3 (CH2OCH2), 55.3 (OCH3), 39.7 (CH) The observed characterization data ($R_f$, IR, $^1$H, $^{13}$C) were consistent with that previously reported.[32]

Notes:

***4** is slightly volatile.*

Dimer **3:** $R_f$ = 0.47 (60% $Et_2O$/pentane); IR (film)/cm$^{-1}$ 2948, 1513, 1249, 992; $^1$H NMR (400 MHz, $CDCl_3$) δ 6.68 (d, *J* = 8.0 Hz, 4 H, 4 × Ar-CH), 6.38 (d, *J* = 8.0 Hz, 4 H, 4 × Ar-CH), 5.30 (d, *J* = 6.3 Hz, 4 H, 2 × C*H*HOC*H*H), 5.07 (d, *J* = 6.3 Hz, 4 H, 2 × CH*H*OCH*H*), 3.78 (s, 6 H, 2 × OCH3); $^{13}$C{$^1$H} NMR (101 MHz, $CDCl_3$) δ 158.1 (2 × Ar-CqOMe), 134.9 (2 × Ar-*C*qCq), 128.3 (4 × Ar-CH), 112.9 (4 × Ar-CH), 79.5 (2 × CH2OCH2), 55.2 (2 × OCH3), 50.9 (2 × Cq); HRMS (APCI) *m/z*: [M+H]$^+$ Calcd for $C_{20}H_{23}O_4^+$ 327.1591; Found 327.1603.

Oxetane **2l**: $R_f$ = 0.20 (60% $Et_2O$/pentane); IR (film)/cm$^{-1}$ 2956, 1740 (C=O st), 1513, 1249, 985; $^1$H NMR (400 MHz, $CDCl_3$) δ 6.91–6.85 (br m, 4 H, 4 × Ar-CH), 5.03–4.99 (br m, 2 H, C*H*HOC*H*H), 4.74 (d, *J* = 5.7 Hz, 1 H, CH*H*OCH2), 4.71 (d, *J* = 5.7 Hz, 1 H, CH2OCH*H*), 3.82 (s, 3 H, OCH3), 2.91 (dddd, *J* = 11.3, 11.3, 6.3, 6.3 Hz, 1 H, CH), 2.37 (dd, *J* = 18.2, 7.4 Hz, 1 H, CHC*H*HCO), 2.24–2.00 (m, 4 H, C*H*HCH2CO, CH2C*H*2CO, CHCH*H*CO), 1.67–1.55 (m, 1 H, CH*H*CH2CO); $^{13}$C{$^1$H} NMR

(101 MHz, $CDCl_3$) δ 217.7 ($C_q$=O), 158.3 (Ar-$C_q$OMe), 134.8 (Ar-$C_qC_q$), 127.5 (2 × Ar-CH), 113.8 (2 × Ar-CH), 80.4 ($CH_2OCH_2$), 80.0 ($CH_2OCH_2$), 55.3 ($OCH_3$), 48.6 ($C_q$), 45.4 (CH), 40.4 ($CHCH_2CO$), 38.4 ($CH_2CH_2CO$), 24.2 ($CH_2CH_2CO$); HRMS (APCI) *m/z*: $[M+H]^+$ Calcd for $C_{15}H_{19}O_3^+$ 247.1329; Found 247.1329.

**Dimethyl 2-(3-(4-methoxyphenyl)oxetan-3-yl)succinate (2m)**

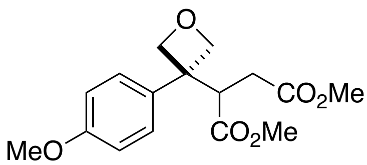

Following **General Procedure B**, oxetane carboxylic acid **1** (41.6 mg) and dimethyl fumarate (43.2 mg) were used. Purification by basic alumina (IV) column chromatography (30% $Et_2O$/pentane) afforded oxetane **2m** as a colorless oil (33.6 mg, 54%). $R_f$ = 0.10 (30% $Et_2O$/pentane); IR (film)/$cm^{-1}$ 2952, 1733 (C=O st), 1513, 1248, 1167, 1028, 987; $^1H$ NMR (400 MHz, $CDCl_3$) δ 6.90–6.85 (m, 4 H, 4 × Ar-CH), 5.12 (d, *J* = 6.2 Hz, 1 H, C*H*HOCH₂), 4.98 (d, *J* = 6.2 Hz, 1 H, CH*H*OCH₂), 4.94 (d, *J* = 6.1 Hz, 1 H, CH₂OC*H*H), 4.84 (d, *J* = 6.1 Hz, 1 H, CH₂OCH*H*), 3.80 (s, 3 H, Ar-$C_qOCH_3$), 3.66 (s, 3 H, $CO_2CH_3$), 3.65 (s, 3 H, $CO_2CH_3$), 3.63 (dd, *J* = 11.4, 3.3 Hz, 1 H, CH), 2.64 (dd, *J* = 16.9, 11.4 Hz, 1 H, CHC*H*H), 2.35 (dd, *J* = 16.9, 3.3 Hz, 1 H, CHCH*H*); $^{13}C\{^1H\}$ NMR (101 MHz, $CDCl_3$) δ 172.5 ($C_q$=O), 172.2 ($C_q$=O), 158.5 (Ar-$C_q$OMe), 133.7 (Ar-$C_qC_q$), 127.4 (2 × Ar-CH), 113.9 (2 × Ar-CH), 80.4 ($CH_2OCH_2$), 80.3 ($CH_2OCH_2$), 55.2 (Ar-$C_qOCH_3$), 51.9 (2 × $CO_2CH_3$), 49.0 (CH), 48.6 ($C_q$), 31.9 ($CH_2CH$); HRMS (ESI) *m/z*: $[M+H]^+$ Calcd for $C_{16}H_{21}O_6$ 309.1338; Found 309.1310.

**3-(4-Methoxyphenyl)-3-phenethyloxetane (2n)**

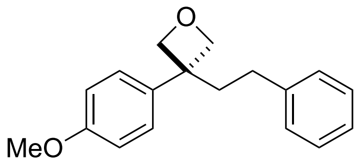

Following **General Procedure A**, oxetane carboxylic acid **1** (41.6 mg) and styrene (35 μL) were used. Purification by basic alumina (IV) column chromatography (10–70% $Et_2O$/pentane) afforded oxetane **2n** as a colorless oil (18.6 mg, 35%, containing 9% of **4**). $R_f$ = 0.21 (10% $Et_2O$/pentane); IR (film)/$cm^{-1}$ 2931, 1513, 1245, 1178, 1031, 980, 829, 700; $^1H$ NMR (400 MHz, $CDCl_3$) δ 7.29–7.25 (m, 2 H, 2 × Ar-CH), 7.20–7.16 (m, 1 H, Ph-CH), 7.14–7.12 (m, 2 H, 2 × Ar-CH), 7.06–7.02 (m, 2 H, 2 × Ar-CH), 6.95–6.91 (m, 2 H, 2 × Ar-CH), 4.98 (d, *J* = 5.5 Hz, 2 H, C*H*HOC*H*H), 4.68 (d, *J* = 5.5 Hz, 2 H, CH*H*OCH*H*), 3.84 (s, 3 H, $OCH_3$), 2.46–2.36 (m, 4 H, $CH_2CH_2$); $^{13}C\{^1H\}$ NMR (101 MHz, $CDCl_3$) δ 158.0 (Ar-$C_q$OMe), 141.7 (Ar-$C_qCH_2$), 136.7 (Ar-$C_qC_q$), 128.4 (2 × Ar-CH), 128.3 (2 × Ar-CH), 126.8 (2 × Ar-CH), 125.9 (Ph-CH), 113.9 (2 × Ar-CH), 81.9 ($CH_2OCH_2$), 55.3 ($OCH_3$), 46.9 ($C_q$), 43.1 ($CH_2CH_2$), 30.9 ($CH_2CH_2$); HRMS (APCI) *m/z*: $[M–OCH_3+H]^{•+}$ Calcd for $C_{17}H_{18}O^{•+}$ 238.1352; Found 238.1358.

**3-(4-Bromophenethyl)-3-(4-methoxyphenyl)oxetane (2o)**

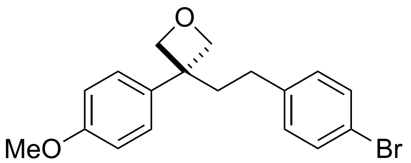

Following **General Procedure A**, oxetane carboxylic acid **1** (41.6 mg) and 1-bromo-4-vinylbenzene (39 μL) were used. Purification by basic alumina (IV) column chromatography (0–20% $Et_2O$/pentane) afforded oxetane **2o** as a colorless oil (20.1 mg). The aqueous phase from the reaction workup was left to evaporate, EtOAc was added (10 mL) and then filtered sequentially through celite and basic alumina (IV) to yield further 30.7 mg of oxetane **2o** (total of 50.8 mg, 73%). $R_f$ = 0.11 (20% $Et_2O$/pentane); IR (film)/$cm^{-1}$ 2933, 1515, 1244, 829; $^1H$ NMR (400 MHz, $CDCl_3$) δ 7.39–7.36 (m, 2 H, 2 × Ar-CH), 7.03–6.97 (m, 4 H, 2 × Ar-CH), 6.94–6.91 (m, 2 H, 2 × Ar-CH), 4.97 (d, *J* = 5.7 Hz, 2 H, C*H*HOC*H*H), 4.66 (d, *J* = 5.7 Hz, 2 H, CH*H*OCH*H*), 3.84 (s, 3 H, $OCH_3$), 2.35 (s, 4 H, $CH_2CH_2$); $^{13}C\{^1H\}$ NMR (101 MHz, $CDCl_3$) δ 158.1 (Ar-$C_q$OMe), 140.7 (Ar-$C_qCH_2$), 136.4 (Ar-$C_qC_q$), 131.4 (2 × Ar-CH), 130.0 (2 × Ar-CH), 126.8 (2 × Ar-CH), 119.6 (Ar-$C_q$Br), 114.0 (2 × Ar-CH), 81.8 ($CH_2OCH_2$), 55.3 ($OCH_3$), 46.8 ($C_q$), 43.0 ($CH_2CH_2$), 30.4 ($CH_2CH_2$); mass-ion not found by HRMS (APCI nor EI).

**5-(2-(3-(4-Methoxyphenyl)oxetan-3-yl)ethyl)isobenzofuran-1(3*H*)-one (2p)**

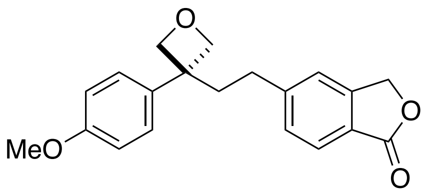

Following **General Procedure B**, oxetane carboxylic acid **1** (41.6 mg) and 5-vinylisobenzofuran-1(3*H*)-one (48.1 mg) were used. Purification by basic alumina (IV) column chromatography (20–70% $Et_2O$/pentane) afforded oxetane **2p** as a white solid (39.1 mg, 60%). $R_f$ = 0.14 (70% $Et_2O$/pentane); mp = 148–149 °C; IR (film)/cm$^{-1}$ 2922, 1751 (C=O st), 1513, 1245, 1036; $^1$H NMR (400 MHz, $CDCl_3$) δ 7.81 (dd, *J* = 7.9, 0.7 Hz, 1 H, Ar-CH), 7.29–7.22 (m, 1 H, Ar-CH), 7.22 (br s, 1 H, Ar-CH), 7.03–6.99 (m, 2 H, 2 × Ar-CH), 6.95–6.91 (m, 2 H, 2 × Ar-CH), 5.26 (s, 2 H, $CH_2OCO$), 5.00 (d, *J* = 5.7 Hz, 2 H, C*H*HOC*H*H), 4.67 (d, *J* = 5.7 Hz, 2 H, CH*H*OCH*H*), 3.84 (s, 3 H, $OCH_3$), 2.57–2.53 (m, 2 H, C*H*$_2$CH$_2$), 2.43–2.39 (m, 2 H, CH$_2$C*H*$_2$); $^{13}$C{$^1$H} NMR (101 MHz, $CDCl_3$) δ 171.0 ($C_q$=O), 158.2 (Ar-$C_q$OMe), 149.1 (Ar-*C*$_q$CO), 147.1 (Ar-*C*$_q$CH$_2$O), 136.1 (Ar-*C*$_q$C$_q$), 129.5 (Ar-CH), 126.7 (2 × Ar-CH), 125.7 (Ar-CH), 123.7 (Ar-*C*$_q$CH$_2$), 121.7 (Ar-CH), 114.0 (2 × Ar-CH), 81.6 ($CH_2OCH_2$), 69.4 (Ar-C$_q$*C*H$_2$O), 55.3 ($OCH_3$), 46.9 ($C_q$), 43.0 (*C*H$_2$CH$_2$), 31.4 (CH$_2$*C*H$_2$); HRMS (ESI) *m/z*: [M+MeCN+H]$^+$ Calcd for $C_{22}H_{24}NO_4$ 366.1705; Found 366.1710.

**4-(2-(3-(4-Methoxyphenyl)oxetan-3-yl)ethyl)pyridine (2q)**

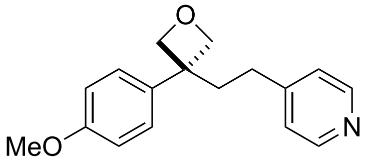

Following **General Procedure A**, oxetane carboxylic acid **1** (41.6 mg) and 4-vinylpyridine (35 μL) were used. Purification by basic alumina (IV) column chromatography (60% $Et_2O$/pentane) afforded oxetane **2q** as a yellow oil which was crystallized by slow evaporation using acetone to form colorless crystals (29.5 mg, 55%). $R_f$ = 0.03 (60% $Et_2O$/pentane); mp = 69–70 °C; IR (film)/cm$^{-1}$ 2869, 1513, 1246, 1179, 1029, 979, 829; $^1$H NMR (400 MHz, $CDCl_3$) δ 8.50–8.42 (m, 2 H, 2 × Ar$_{(pyridine)}$-CH), 7.06–7.04 (m, 2 H, 2 × Ar$_{(pyridine)}$-CH), 7.03–6.99 (m, 2 H, 2 × Ar-CH), 6.95–6.91 (m, 2 H, 2 × Ar-CH), 5.00 (d, *J* = 5.7 Hz, 2 H, C*H*HOC*H*H), 4.67 (d, *J* = 5.7 Hz, 2 H, CH*H*OCH*H*), 3.84 (s, 3 H, $OCH_3$), 2.44–2.35 (m, 4 H, $CH_2CH_2$); $^{13}$C{$^1$H} NMR (101 MHz, $CDCl_3$) δ 158.2 (Ar-$C_q$OMe), 150.8 (Ar$_{(pyridine)}$-*C*$_q$CH$_2$), 149.6 (2 × Ar$_{(pyridine)}$-CH), 136.1 (Ar-*C*$_q$C$_q$), 126.7 (2 × Ar-CH), 123.7 (2 × Ar$_{(pyridine)}$-CH), 114.0 (2 × Ar-CH), 81.6 ($CH_2OCH_2$), 55.3 ($OCH_3$), 46.8 ($C_q$), 41.8 (*C*H$_2$CH$_2$), 30.3 (CH$_2$*C*H$_2$); HRMS (ESI) *m/z*: [M+H]$^+$ Calcd for $C_{17}H_{20}NO_2^+$ 270.1489; Found 270.1486.

Notes:

***2q** was further characterized by X-ray crystallography (see Figures S31–S32).*

**4-(2-(3-(4-(Benzyloxy)phenyl)oxetan-3-yl)ethyl)pyridine (5q)**

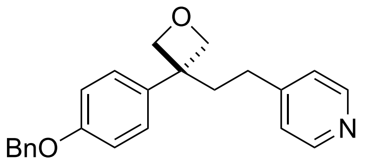

Following **General Procedure A**, 3-(4-(benzyloxy)phenyl)oxetane-3-carboxylic acid (56.9 mg) and 4-vinylpyridine (35 μL) were used. Purification by basic alumina (IV) column chromatography (50% $Et_2O$/pentane) afforded oxetane **5q** as a yellow solid (42.5 mg, 62%). $R_f$ = 0.10 (70% $Et_2O$/pentane); mp = 106–108 °C; IR (film)/cm$^{-1}$ 2859, 1595, 1513, 1245, 1185, 1118, 1021, 980, 797, 741; $^1$H NMR (400 MHz, $CDCl_3$) δ 8.48–8.46 (m, 2 H, 2 × Ar$_{(pyridine)}$-CH), 7.47–7.33 (m, 5 H, 5 × Ph-CH), 7.06–7.04 (m, 2 H, 2 × Ar$_{(pyridine)}$-CH), 7.01 (s, 4 H, 4 × Ar-CH), 5.09 (s, 2 H, $CH_2Ph$), 4.99 (d, *J* = 5.7 Hz, 2 H, C*H*HOC*H*H), 4.67 (d, *J* = 5.7 Hz, 2 H, CH*H*OCH*H*), 2.45–2.35 (m, 4 H, $CH_2CH_2$); $^{13}$C{$^1$H} NMR (101 MHz, $CDCl_3$) δ 157.4 (Ar-$C_q$OBn), 150.6 (Ar$_{(pyridine)}$-*C*$_q$CH$_2$), 149.8 (2 × Ar$_{(pyridine)}$-CH), 136.9 (Ar-*C*$_q$CH$_2$), 136.4 (Ar-*C*$_q$C$_q$), 128.6 (2 × Ph-CH), 128.0 (Ph-CH), 127.5 (2 × Ph-CH), 126.8 (2 × Ar-CH), 123.7 (2 × Ar$_{(pyridine)}$-CH), 115.0 (2 × Ar-CH), 81.6 ($CH_2OCH_2$), 70.1 ($CH_2Ph$), 46.9 ($C_q$), 41.8 (*C*H$_2$CH$_2$), 30.3 (CH$_2$*C*H$_2$); HRMS (ESI) *m/z*: [M+H]$^+$ Calcd for $C_{23}H_{24}NO_2$ 346.1807; Found 346.1813.

**4-(3-(2-(Pyridin-4-yl)ethyl)oxetan-3-yl)phenyl trifluoromethanesulfonate (6q)**

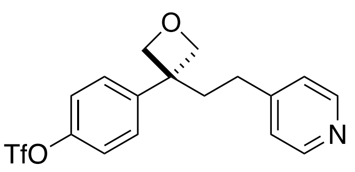


Following **General Procedure A**, 3-(4-(((trifluoromethyl)sulfonyl)oxy)phenyl)oxetane-3-carboxylic acid (65.2 mg) and 4-vinylpyridine (35 μL) were used. Purification by basic alumina (IV) column chromatography (50% $Et_2O$/pentane) afforded oxetane **6q** as a colourless oil (6.6 mg, 70% pure, 9%). $R_f$ = 0.08 ($Et_2O$); IR (film)/$cm^{-1}$ 2945 1424, 1215, 1141, 891; $^1H$ NMR (400 MHz, $CDCl_3$) δ 8.50–8.48 (m, 2 H, 2 × $Ar_{(pyridine)}$-CH), 7.34–7.30 (m, 2 H, 2 × Ar-CH), 7.19–7.16 (m, 2 H, 2 × Ar-CH), 7.06–7.04 (m, 2 H, 2 × $Ar_{(pyridine)}$-CH), 4.99 (d, *J* = 5.9 Hz, 2 H, C*H*HOC*H*H), 4.72 (d, *J* = 5.9 Hz, 2 H, CH*H*OCH*H*), 2.42 (s, 4 H, $CH_2CH_2$); $^{13}C\{^1H\}$ NMR (101 MHz, $CDCl_3$) δ 149.94 ($Ar_{(pyridine)}$-$C_qCH_2$), 149.86 (2 × $Ar_{(pyridine)}$-CH), 148.1 (Ar-$C_q$OTf), 144.7 (Ar-$C_qC_q$), 127.6 (2 × Ar-CH), 123.6 (2 × $Ar_{(pyridine)}$-CH), 121.7 (2 × Ar-CH), 81.1 ($CH_2OCH_2$), 47.3 ($C_q$), 41.5 ($CH_2CH_2$), 30.2 ($CH_2CH_2$); $^{19}F\{^1H\}$ NMR (377 MHz, $CDCl_3$) δ –72.8; HRMS (APCI) *m/z*: $[M+H]^+$ Calcd for $C_{17}H_{17}NO_4SF_3^+$ 388.0825; Found 388.0818.

Notes:

*The $CF_3$ signal was not observed in the $^{13}C$ NMR spectrum.*

**5-Chloro-4-methoxy-2-(2-(3-(4-methoxyphenyl)oxetan-3-yl)ethyl)pyridine (2r)**

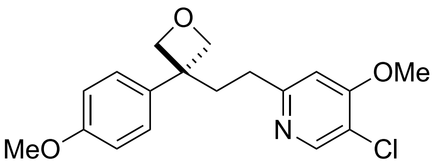


Following **General Procedure B**, oxetane carboxylic acid **1** (41.6 mg) and 5-chloro-4-methoxy-2-vinylpyridine (50.9 mg) were used. Purification by basic alumina (IV) column chromatography (80–100% $Et_2O$/pentane) afforded oxetane **2r** as a colorless gum (28.9 mg, 40%). $R_f$ = 0.14 ($Et_2O$); IR (film)/$cm^{-1}$ 2931, 2224, 1589, 1513, 1245, 1024, 979, 831; $^1H$ NMR (400 MHz, $CDCl_3$) δ 8.55 (s, 1 H, $Ar_{(pyridine)}$-CH), 7.01–6.98 (m, 2 H, 2 × Ar-CH), 6.91–6.88 (m, 2 H, 2 × Ar-CH), 6.62 (s, 1 H, $Ar_{(pyridine)}$-CH), 4.99 (d, *J* = 5.7 Hz, 2 H, C*H*HOC*H*H), 4.69 (d, *J* = 5.7 Hz, 2 H, CH*H*OCH*H*), 3.94 (s, 3 H, $OCH_3$), 3.81 (s, 3 H, $OCH_3$), 2.65–2.61 (m, 2 H, $CH_2CH_2$), 2.52–2.48 (m, 2 H, $CH_2CH_2$); $^{13}C\{^1H\}$ NMR (101 MHz, $CDCl_3$) δ 168.1 ($Ar_{(pyridine)}$-$C_qCH_2$), 166.7 ($Ar_{(pyridine)}$-$C_q$OMe), 158.1 ($Ar_{(PMP)}$-$C_q$OMe), 153.6 ($Ar_{(pyridine)}$-CH), 136.1 (Ar-$C_qC_q$), 126.8 (2 × Ar-CH), 114.0 (2 × Ar-CH), 105.4 ($Ar_{(pyridine)}$-CH), 97.7 ($Ar_{(pyridine)}$-$C_q$Cl), 81.6 ($CH_2OCH_2$), 56.1 ($OCH_3$), 55.3 ($OCH_3$), 46.7 ($C_q$), 40.3 ($CH_2CH_2$), 34.4 ($CH_2CH_2$); mass-ion not found by HRMS (APCI nor EI).

Notes:

*There is an additional signal in the $^{13}C$ NMR spectrum at 114.5 ppm which shows clear HMBC correlation signals with the pyridine protons (page S112). Presumably this is due to chemical exchange between two different pyridine species in equilibrium (for example pyridine–pyridinium).*

*Presumably strong intramolecular fragmentation, known for 2-alkylpyridines,[33] led to intractable ion fragments in the mass spectrum.*

***N*-(5-(2-(3-(4-Methoxyphenyl)oxetan-3-yl)ethyl)pyridin-2-yl)acetamide (2s)**

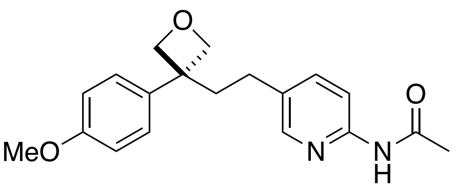


Following **General Procedure B**, oxetane carboxylic acid **1** (41.6 mg) and *N*-(5-vinylpyridin-2-yl)acetamide (49.0 mg) were used. Purification by basic alumina (IV) column chromatography (100% $CH_2Cl_2$) afforded oxetane **2s** as a colorless oil (19.0 mg, 29%). $R_f$ = 0.13 (50% $Et_2O/CH_2Cl_2$); IR (film)/$cm^{-1}$ 2937, 1692 (C=O st), 1521, 1305, 1252, 835; $^1H$ NMR (400 MHz, $CDCl_3$) δ 8.39 (s, 1 H, NH), 8.09 (d, *J* = 8.5 Hz, 1 H, $Ar_{(pyridine)}$-CH), 8.00 (d, *J* = 2.4 Hz, 1 H, $Ar_{(pyridine)}$-CH), 7.46 (dd, *J* = 8.5, 2.4 Hz, 1 H, $Ar_{(pyridine)}$-CH), 7.03–6.99 (m, 2 H, 2 × Ar-CH), 6.94–6.91 (m, 2 H, 2 × Ar-CH), 4.98 (d, *J* = 5.7 Hz, 2 H, C*H*HOC*H*H), 4.67 (d, *J* = 5.7 Hz, 2 H, CH*H*OCH*H*), 3.83 (s, 3 H, $OCH_3$), 2.36 (s, 4 H, $CH_2CH_2$), 2.18 (s, 3 H, $C(O)CH_3$), $^{13}C\{^1H\}$ NMR

(101 MHz, $CDCl_3$) δ 168.5 ($C_q$=O), 158.1 (Ar-$C_q$OMe), 149.7 ($Ar_{(pyridine)}$-$C_q$NH), 147.1 ($Ar_{(pyridine)}$-CH), 138.2 ($Ar_{(pyridine)}$-CH), 136.2 (Ar-$C_q$$C_q$), 132.9 ($Ar_{(pyridine)}$-$C_q$$CH_2$), 126.8 (2 × Ar-CH), 114.0 (2 × Ar-CH), 113.7 ($Ar_{(pyridine)}$-CH), 81.7 ($CH_2OCH_2$), 55.3 ($OCH_3$), 46.8 ($C_q$), 42.8 ($CH_2CH_2$), 27.5 ($CH_2CH_2$), 24.6 (C(O)$CH_3$); HRMS (ESI) *m/z*: $[M+H]^+$ Calcd for $C_{19}H_{23}N_2O_3$ 327.1709; Found 327.1699.

Notes:

*Attempts to increase the yield of **2s** by using a higher excess of vinyl pyridine (3.0 equiv; 28% yield) or by using oxetane acid **1** in excess (3.0 equiv; 1.0 equiv vinyl pyridine; 24% yield) were unsuccessful.*

**2-(1-(4-Bromophenyl)-2-(3-(4-methoxyphenyl)oxetan-3-yl)ethyl)pyridine (2t)**

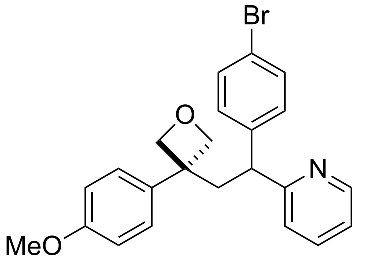


Following **General Procedure B**, oxetane carboxylic acid **1** (41.6 mg) and 2-(1-(4-bromophenyl)vinyl)pyridine (78.0 mg) were used. Purification by basic alumina (IV) column chromatography (60% $Et_2O$/pentane) afforded oxetane **2t** as a colorless paste (44.3 mg, 53%). $R_f$ = 0.03 (70% $Et_2O$/pentane); IR (film)/$cm^{-1}$ 2933, 1513, 1249, 1181, 831; $^1H$ NMR (400 MHz, $CDCl_3$) δ 8.42 (dd, *J* = 4.8, 1.6 Hz, 1 H, $Ar_{(pyridine)}$-CH), 8.36 (br d, *J* = 2.2 Hz, 1 H, $Ar_{(pyridine)}$-CH), 7.45 (br dt, *J* = 7.9, 1.9 Hz, 1 H, $Ar_{(pyridine)}$-CH), 7.41–7.37 (m, 2 H, 2 × $Ar_{(p\text{-}Br)}$-CH), 7.17 (ddd, *J* = 7.9, 4.8, 0.8 Hz, 1 H, $Ar_{(pyridine)}$-CH), 7.05–7.01 (m, 2 H, 2 × $Ar_{(p\text{-}Br)}$-CH), 6.99–6.95 (m, 2 H, 2 × Ar-CH), 6.93–6.89 (m, 2 H, 2 × Ar-CH), 4.72 (d, *J* = 5.9 Hz, 1 H, C*H*HOCH₂), 4.71 (d, *J* = 5.9 Hz, 1 H, $CH_2$OC*H*H), 4.39 (d, *J* = 5.9 Hz, 1 H, CH*H*OCH₂), 4.38 (d, *J* = 5.9 Hz, 1 H, $CH_2$OCH*H*), 3.84 (s, 3 H, $OCH_3$), 3.60 (dd, *J* = 7.5, 7.0 Hz, 1 H, CH), 2.95 (dd, *J* = 13.9, 7.5 Hz, 1 H, C*H*H), 2.86 (dd, *J* = 13.9, 7.0 Hz, 1 H, CH*H*); $^{13}C\{^1H\}$ NMR (101 MHz, $CDCl_3$) δ 158.2 (Ar-$C_q$OMe), 149.4 ($Ar_{(pyridine)}$-CH), 148.0 ($Ar_{(pyridine)}$-CH), 142.3 ($Ar_{(pyridine)}$-$C_q$CH), 139.3 ($Ar_{(p\text{-}Br)}$-$C_q$CH), 135.7 (Ar-$C_q$$C_q$), 134.9 ($Ar_{(pyridine)}$-CH), 131.8 (2 × $Ar_{(p\text{-}Br)}$-CH), 129.5 (2 × $Ar_{(p\text{-}Br)}$-CH), 127.0 (2 × Ar-CH), 123.4 ($Ar_{(pyridine)}$-CH), 120.6 (Ar-$C_q$Br), 114.2 (2 × Ar-CH), 81.9 ($CH_2OCH_2$), 81.5 ($CH_2OCH_2$), 55.3 ($OCH_3$), 47.0 ($C_q$), 45.8 ($CH_2$), 44.9 (CH); HRMS (ESI) *m/z*: $[M+H]^+$ Calcd for $C_{23}H_{23}NO_2{}^{79}Br$ 424.0912; Found 424.0904.

Notes:

*The doublets at 4.72 and 4.71 ppm and the doublets at 4.39 and 4.38 ppm in the $^1H$ NMR spectrum were integrated together to 2 H and 2 H, respectively.*

**Ethyl 3-(3-(4-hydroxyphenyl)oxetan-3-yl)propanoate (7a)** and **diethyl 2-((3-(4-hydroxyphenyl)oxetan-3-yl)methyl)pentanedioate (7a')**

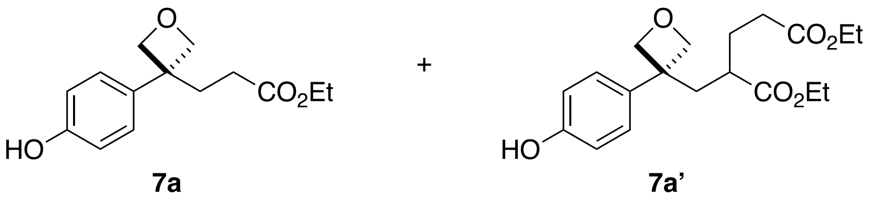


**7a** **7a'**

Following **General Procedure A**, 3-(4-((triisopropylsilyl)oxy)phenyl)oxetane-3-carboxylic acid (70.1 mg) and ethyl acrylate (33 μL) were used. Purification by basic alumina (IV) column chromatography (30–50% $Et_2O$/pentane) afforded oxetane **7a** as a colorless oil (11.1 mg, 22%), followed by oxetane **7a'** as a colorless oil (3.6 mg, 5%).

Oxetane **7a**: $R_f$ = 0.29 (50% $Et_2O$/pentane); IR (film)/$cm^{-1}$ 3306 (OH st, br), 2959, 1729 (C=O st), 1517, 1267, 1226, 1177, 834; $^1H$ NMR (400 MHz, $CDCl_3$) δ 6.90–6.86 (m, 2 H, 2 × Ar-CH), 6.81–6.77 (m, 2 H, 2 × Ar-CH), 5.75 (s, 1 H, OH), 4.96 (d, *J* = 5.7 Hz, 2 H, C*H*HOC*H*H), 4.66 (d, *J* = 5.7 Hz, 2 H, CH*H*OCH*H*), 4.07 (q, *J* = 7.2 Hz, 2 H, $CH_2CH_3$), 2.42–2.38 (m, 2 H, $CH_2CH_2$), 2.15–2.11 (m, 2 H, $CH_2CH_2$), 1.22 (t, *J* = 7.2 Hz, 3 H, $CH_2CH_3$); $^{13}C\{^1H\}$ NMR (101 MHz, $CDCl_3$) δ 173.3 ($C_q$=O),

154.4 (Ar-C$_q$OH), 135.5 (Ar-*C$_q$*C$_q$), 127.0 (2 × Ar-CH), 115.4 (2 × Ar-CH), 81.7 (CH$_2$OCH$_2$), 60.6 (*C*H$_2$CH$_3$), 46.2 (C$_q$), 35.9 (*C*H$_2$CH$_2$), 29.9 (CH$_2$*C*H$_2$), 14.1 (CH$_2$*C*H$_3$); HRMS (APCI) *m/z*: $[M+H]^+$ Calcd for $C_{14}H_{19}O_4^+$ 251.1278; Found 251.1281.

Notes:

***7a** was also obtained from unprotected 3-(4-hydroxyphenyl)oxetane-3-carboxylic acid in 17% yield.*

Oxetane **7a'**: R$_f$ = 0.18 (50% Et$_2$O/pentane); IR (film)/cm$^{-1}$ 3377 (OH st, br), 2960, 1729 (C=O st), 1521, 1267, 1222, 1177; $^1$H NMR (400 MHz, CDCl$_3$) δ 6.95–6.91 (m, 2 H, 2 × Ar-CH), 6.81–6.78 (m, 2 H, 2 × Ar-CH), 5.10 (s, 1 H, OH), 4.95 (d, *J* = 5.7 Hz, 1 H, C*H*HOCH$_2$), 4.88 (d, *J* = 5.7 Hz, 1 H, CH$_2$OC*H*H), 4.64 (d, *J* = 5.7 Hz, 1 H, CH*H*OCH$_2$), 4.62 (d, *J* = 5.7 Hz, 1 H, CH$_2$OCH*H*), 4.09 (q, *J* = 7.2 Hz, 2 H, C*H$_2$*CH$_3$), 3.97–3.83 (m, 2 H, C*H$_2$*CH$_3$), 2.62 (dd, *J* = 14.2, 10.3 Hz, 1 H, C$_q$C*H*HCH), 2.22–2.11 (m, 4 H, C$_q$CH*H*CH, CH, CHC*H$_2$*CH$_2$), 1.90–1.80 (m, 1 H, CH$_2$C*H*HCO$_2$), 1.74–1.66 (m, 1 H, CH$_2$CH*H*CO$_2$), 1.22 (t, *J* = 7.2 Hz, 3 H, CH$_2$C*H$_3$*), 1.19 (t, *J* = 7.2 Hz, 3 H, CH$_2$C*H$_3$*); $^{13}$C{$^1$H} NMR (101 MHz, CDCl$_3$) δ 175.0 (C$_q$=O), 172.7 (C$_q$=O), 154.2 (Ar-C$_q$OH), 135.8 (Ar-*C$_q$*C$_q$), 127.3 (2 × Ar-CH), 115.3 (2 × Ar-CH), 82.1 (*C*H$_2$OCH$_2$), 81.8 (CH$_2$O*C*H$_2$), 60.54 (*C*H$_2$CH$_3$), 60.48 (*C*H$_2$CH$_3$), 46.5 (C$_q$), 43.3 (C$_q$*C*H$_2$CH), 41.4 (CH), 31.7 (CH*C*H$_2$CH$_2$), 28.7 (CH$_2$*C*H$_2$CO$_2$), 14.1 (CH$_2$*C*H$_3$), 14.0 (CH$_2$*C*H$_3$); HRMS (APCI) *m/z*: $[M+H]^+$ Calcd for $C_{19}H_{27}O_6^+$ 351.1802; Found 351.1802.

Notes:

*The doublets at 4.64 and 4.62 ppm in the $^1$H NMR spectrum were integrated together to 2 H.*

**Ethyl 3-(3-phenyloxetan-3-yl)propanoate (8a)** and **diethyl 2-((3-phenyloxetan-3-yl)methyl)pentanedioate (8a')**

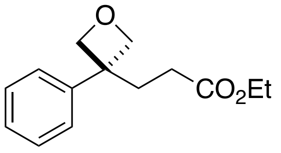


**8a**

\+

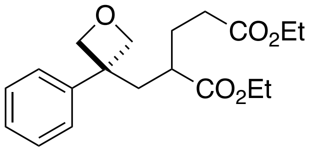


**8a'**

Following **General Procedure A**, 3-phenyloxetane-3-carboxylic acid (35.6 mg) and ethyl acrylate (33 μL) were used. Purification by basic alumina (IV) column chromatography (20% Et$_2$O/pentane) afforded oxetane **8a** as a colorless oil (17.2 mg, 37%), followed by oxetane **8a'** as a colorless oil (5.3 mg, 8%).

Oxetane **8a**: R$_f$ = 0.38 (30% Et$_2$O/pentane); IR (film)/cm$^{-1}$ 2937, 1730 (C=O st), 1179, 980, 701; $^1$H NMR (400 MHz, CDCl$_3$) δ 7.38–7.34 (m, 2 H, 2 × Ar-CH), 7.28–7.23 (m, 1 H, Ar-CH), 7.03–7.00 (m, 2 H, 2 × Ar-CH), 5.00 (d, *J* = 5.7 Hz, 2 H, C*H*HOC*H*H), 4.67 (d, *J* = 5.7 Hz, 2 H, CH*H*OCH*H*), 4.06 (q, *J* = 7.1 Hz, 2 H, C*H$_2$*CH$_3$), 2.46–2.42 (m, 2 H, C*H$_2$*CH$_2$), 2.18–2.12 (m, 2 H, CH$_2$C*H$_2$*), 1.22 (t, *J* = 7.1 Hz, 3 H, CH$_2$C*H$_3$*); $^{13}$C{$^1$H} NMR (101 MHz, CDCl$_3$) δ 173.0 (C$_q$=O), 143.8 (Ar-*C$_q$*C$_q$), 128.6 (2 × Ar-CH), 126.6 (Ar-CH), 125.7 (2 × Ar-CH), 81.4 (CH$_2$OCH$_2$), 60.5 (*C*H$_2$CH$_3$), 46.8 (C$_q$), 35.9 (*C*H$_2$CH$_2$), 29.8 (CH$_2$*C*H$_2$), 14.1 (CH$_2$*C*H$_3$); HRMS (ESI) *m/z*: $[M+H]^+$ Calcd for $C_{14}H_{19}O_3$ 235.1334; Found 235.1340.

Oxetane **8a'**: R$_f$ = 0.24 (30% Et$_2$O/pentane); IR (film)/cm$^{-1}$ 2937, 1729 (C=O st), 1159, 705; $^1$H NMR (400 MHz, CDCl$_3$) δ 7.37–7.32 (m, 2 H, 2 × Ar-CH), 7.26–7.22 (m, 1 H, Ar-CH), 7.07–7.04 (m, 2 H, 2 × Ar-CH), 5.00 (d, *J* = 5.5 Hz, 1 H, C*H*HOCH$_2$), 4.93 (d, *J* = 5.7 Hz, 1 H, CH$_2$OC*H*H), 4.66 (d, *J* = 5.5 Hz, 1 H, CH*H*OCH$_2$), 4.65 (d, *J* = 5.7 Hz, 1 H, CH$_2$OCH*H*), 4.08 (q, *J* = 7.2 Hz, 2 H, C*H$_2$*CH$_3$), 3.93–3.80 (m, 2 H, C*H$_2$*CH$_3$), 2.66 (dd, *J* = 14.6, 10.3 Hz, 1 H, C$_q$C*H*HCH), 2.21–2.12 (m, 4 H, C$_q$CH*H*CH + CH + CHC*H$_2$*CH$_2$), 1.89–1.80 (m, 1 H, CH$_2$C*H*HCO$_2$), 1.74–1.65 (m, 1 H, CH$_2$CH*H*CO$_2$), 1.21 (t, *J* = 7.2 Hz, 3 H, CH$_2$C*H$_3$*), 1.17 (t, *J* = 7.2 Hz, 3 H, CH$_2$C*H$_3$*); $^{13}$C{$^1$H} NMR (101 MHz, CDCl$_3$) δ 175.0 (C$_q$=O), 172.6 (C$_q$=O), 143.7 (Ar-*C$_q$*C$_q$), 128.4 (2 × Ar-CH), 126.6 (Ar-CH), 126.0 (2 × Ar-CH), 82.0 (*C*H$_2$OCH$_2$), 81.6 (CH$_2$O*C*H$_2$), 60.5 (*C*H$_2$CH$_3$), 60.4 (*C*H$_2$CH$_3$), 47.1 (C$_q$), 43.2 (C$_q$*C*H$_2$CH), 41.3 (CH), 31.6 (CH*C*H$_2$CH$_2$), 28.7 (CH$_2$*C*H$_2$CO$_2$), 14.2 (CH$_2$*C*H$_3$), 14.0 (CH$_2$*C*H$_3$); HRMS (APCI) *m/z*: $[M+H]^+$ Calcd for $C_{19}H_{27}O_5^+$ 335.1853; Found 335.1846.

Notes:

***8a** was obtained in 49% yield (11.4 mg) when using repurified Ph-oxetane carboxylic acid (See pages S33–34) on a 0.1 mmol scale for 14 h.*

*The doublets at 4.66 and 4.65 ppm in the $^1$H NMR spectrum were integrated together to 2 H.*

**Ethyl 3-(3-(4-isobutylphenyl)oxetan-3-yl)propanoate (9a) and diethyl 2-((3-(4-isobutylphenyl)oxetan-3-yl)methyl)pentanedioate (9a')**

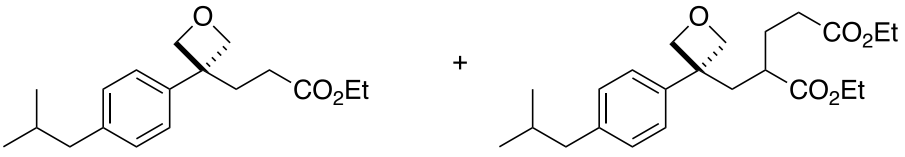

**9a** **9a'**

Following **General Procedure A**, 3-(4-isobutylphenyl)oxetane-3-carboxylic acid (47.2 mg) and ethyl acrylate (33 μL) were used. Purification by basic alumina (IV) column chromatography (10–50% $Et_2O$/pentane) afforded oxetane **9a** as a colorless oil (13.0 mg, 22% yield; containing 4% of oxetane dimer) followed by oxetane **9a'** as a colorless oil (4.0 mg, 5%).

Oxetane **9a**: $R_f$ = 0.19 (20% $Et_2O$/pentane); IR (film)/cm$^{-1}$ 2950, 1733 (C=O st), 1179, 983; $^1$H NMR (400 MHz, $CDCl_3$) δ 7.14–7.11 (m, 2 H, 2 × Ar-H), 6.94–6.91 (m, 2 H, 2 × Ar-CH), 4.98 (d, *J* = 5.8 Hz, 2 H, C*H*HOC*H*H), 4.66 (d, *J* = 5.8 Hz, 2 H, CH*H*OCH*H*), 4.06 (q, *J* = 7.1 Hz, 2 H, C*$H_2$*$CH_3$), 2.47–2.40 (m, 4 H, C*$H_2$*CH + C*$H_2$*$CH_2$), 2.15–2.11 (m, 2 H, $CH_2$C*$H_2$*), 1.86 (hept, *J* = 6.7 Hz, 1 H, CH), 1.22 (t, *J* = 7.1 Hz, 3 H, $CH_2$C*$H_3$*), 0.91 (d, *J* = 6.7 Hz, 6 H, 2 × CHC*$H_3$*); $^{13}$C{$^1$H} NMR (101 MHz, $CDCl_3$) δ 173.1 ($C_q$=O), 141.0 (Ar-*$C_q$*$C_q$), 140.0 (Ar-*$C_q$*$CH_2$), 129.2 (2 × Ar-CH), 125.4 (2 × Ar-CH), 81.5 ($CH_2OCH_2$), 60.4 (*C*$H_2CH_3$), 46.6 ($C_q$), 45.0 (*C*$H_2$CH), 35.9 (*C*$H_2CH_2$), 30.2 (CH), 29.9 ($CH_2$*C*$H_2$), 22.4 (2 × $CH_3$), 14.1 ($CH_2CH_3$); HRMS (ESI) *m/z*: [M+H]$^+$ Calcd for $C_{18}H_{27}O_3$ 291.1960; Found 291.1956.

Oxetane **9a'**: $R_f$ = 0.16 (50% $Et_2O$/pentane); IR (film)/cm$^{-1}$ 2952, 1731 (C=O st), 1159; $^1$H NMR (400 MHz, $CDCl_3$) δ 7.12–7.10 (m, 2 H, 2 × Ar-CH), 6.96–6.94 (m, 2 H, 2 × Ar-CH), 4.98 (d, *J* = 5.8 Hz, 1 H, C*H*HOC$H_2$), 4.92 (d, *J* = 5.8 Hz, 1 H, $CH_2$OC*H*H), 4.65 (d, *J* = 5.8 Hz, 1 H, CH*H*OC$H_2$), 4.64 (d, *J* = 5.8 Hz, 1 H, $CH_2$OCH*H*), 4.08 (q, *J* = 7.1 Hz, 2 H, C*$H_2$*$CH_3$), 3.93–3.83 (m, 2 H, C*$H_2$*$CH_3$), 2.67–2.62 (m, 1 H, CH$CO_2$Et), 2.45 (d, *J* = 7.2 Hz, 2 H, C*$H_2$*$CO_2$Et), 2.22–2.11 (m, 4 H, C*$H_2$*CH$(CH_3)_2$ + $C_q$C*$H_2$*CH), 1.89–1.81 (m, 2 H, C*$H_2$*$CH_2CO_2$Et), 1.71–1.67 (m, 1 H, C*H*$(CH_3)_2$), 1.23–1.16 (m, 6 H, 2 × $CH_2$C*$H_3$*), 0.91 (d, *J* = 6.6 Hz, 6 H, 2 × CHC*$H_3$*); $^{13}$C{$^1$H} NMR (101 MHz, $CDCl_3$) δ 175.1 ($C_q$=O), 172.5 ($C_q$=O), 141.0 (Ar-*$C_q$*$C_q$), 139.9 (Ar-*$C_q$*$CH_2$), 129.1 (2 × Ar-CH), 125.8 (2 × Ar-CH), 82.2 (*C*$H_2OCH_2$), 81.7 ($CH_2$O*C*$H_2$), 60.4 (2 × *C*$H_2CH_3$), 46.8 ($C_q$), 45.0 (*C*$H_2CO_2$Et), 43.3 (*C*H$CO_2$Et), 41.3 ($C_q$*C*$H_2$CH), 31.7 (*C*$H_2CH_2CO_2$Et), 30.2 (*C*$H_2$CH$(CH_3)_2$), 28.7 (*C*H$(CH_3)_2$), 22.3 (2 × CH*C*$H_3$), 14.1 ($CH_2$*C*$H_3$), 14.0 ($CH_2$*C*$H_3$); HRMS (ESI) *m/z*: [M+H]$^+$ Calcd for $C_{23}H_{35}O_5$ 391.2484; Found 391.2496.

Notes:

*The doublets at 4.65 and 4.64 ppm in the $^1$H NMR spectrum were integrated together to 2 H.*

**Ethyl 3-(3-(2-fluoro-[1,1'-biphenyl]-4-yl)oxetan-3-yl)propanoate (10a) and 3-(2-fluoro-[1,1'-biphenyl]-4-yl)oxetane (S7)**

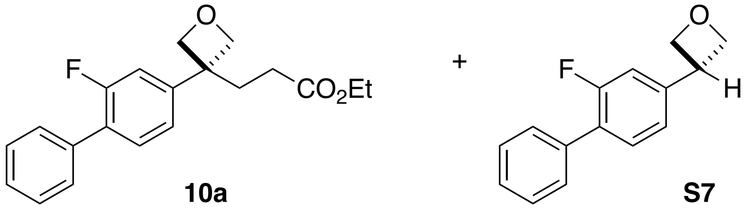

**10a** **S7**

Following **General Procedure A**, 3-(2-fluoro-[1,1'-biphenyl]-4-yl)oxetane-3-carboxylic acid (54.5 mg) and ethyl acrylate (35 μL). Purification by basic alumina (IV) column chromatography (0–50% pentane/hexane) afforded oxetane **10a** as a yellow oil (18.4 mg, 28%) followed by oxetane **S7** as a yellow oil (6.9 mg, 15%).

Oxetane **10a:** $R_f$ = 0.11 (100% hexane); IR (film)/cm$^{-1}$ 2931, 1729 (C=O st), 1182; $^1$H NMR (400 MHz, $CDCl_3$) δ 7.56–7.54 (m, 2 H, 2 × Ar-CH), 7.48–7.44 (m, 3 H, 3 × Ar-CH), 7.42–7.39 (m, 1 H, Ar-CH), 6.91 (dd, *J* = 7.9, 1.9 Hz, 1 H, Ar-CH), 6.86 (dd, $J_{C\text{-}F}$ = 11.5 Hz, $J_{C\text{-}H}$ = 1.9 Hz, 1 H, Ar-CH), 4.99 (d, *J* = 5.8 Hz, 2 H, C*H*HOC*H*H), 4.69 (d, *J* = 5.8 Hz, 2 H, CH*H*OCH*H*), 4.09 (q, *J* = 7.2 Hz, 2 H, C$H_2$$CH_3$), 2.49–2.45 (m, 2 H, C$H_2$$CH_2$), 2.22–2.18 (m, 2 H, $CH_2$C$H_2$), 1.24 (t, *J* = 7.2 Hz, 3 H, $CH_2$C$H_3$); $^{13}$C{$^1$H} NMR (101 MHz, $CDCl_3$) δ 172.8 ($C_q$=O), 159.8 (d, $^1J_{C\text{-}F}$ = 249.1 Hz, Ar-$C_q$F), 145.4 (d, $^3J_{C\text{-}F}$ = 6.9 Hz, Ar-$C_q$$C_q$), 135.3 (Ar-$C_q$Ar-$C_q$), 131.0 (d, $^3J_{C\text{-}F}$ = 4.0 Hz, Ar-CH), 128.9 (d, $^4J_{C\text{-}F}$ = 2.7 Hz, 2 × Ar-CH), 128.5 (2 × Ar-CH + Ar-$C_q$Ar-$C_q$), 127.7 (Ar-CH), 121.8 (d, $^4J_{C\text{-}F}$ = 3.4 Hz, Ar-CH), 113.7 (d, $^2J_{C\text{-}F}$ = 23.5 Hz, Ar-CH), 81.2 ($CH_2OCH_2$), 60.6 (C$H_2$$CH_3$), 46.7 ($C_q$), 35.6 (C$H_2$$CH_2$), 29.8 ($CH_2$C$H_2$), 14.1 ($CH_3$); $^{19}$F{$^1$H} NMR (377 Hz, $CDCl_3$) δ –117.1; HRMS (ESI) *m/z*: $[M+H]^+$ Calcd for $C_{20}H_{22}O_3F$ 329.1553; Found 329.1559.

Oxetane **S7**: $R_f$ = 0.11 (10% EtOAc/hexane); IR (film)/cm$^{-1}$: 2957, 2870, 1483, 1418, 981, 766, 698; $^1$H NMR (400 MHz, $CDCl_3$) δ 7.58–7.55 (m, 2 H, 2 × Ar-CH), 7.48–7.43 (m, 3 H, 3 × Ar-CH), 7.41–7.36 (m, 1 H, Ar-CH), 7.27–7.21 (m, 2 H, 2 × Ar-CH), 5.12 (dd, *J* = 8.3, 6.2 Hz, 2 H, C*H*HOC*H*H), 4.80 (dd, *J* = 6.2, 6.2 Hz, 2 H, CH*H*OCH*H*), 4.30–4.23 (m, 1 H, CH); $^{13}$C{$^1$H} NMR (101 MHz, $CDCl_3$) δ 159.8 (d, $^1J_{C\text{-}F}$ = 247.7 Hz, Ar-$C_q$F) 143.1 (d, $^3J_{C\text{-}F}$ = 7.3 Hz, Ar-$C_q$$C_q$), 135.4 (Ar-$C_q$Ar-$C_q$), 131.0 (d, $^3J_{C\text{-}F}$ = 4.2 Hz, Ar-CH), 128.9 (d, $^4J_{C\text{-}F}$ = 2.8 Hz, 2 × Ar-CH), 128.5 (2 × Ar-CH + Ar-$C_q$Ar-$C_q$), 127.7 (Ar-CH), 122.8 (d, $^3J_{C\text{-}F}$ = 3.4 Hz, Ar-CH), 114.5 (d, $^2J_{C\text{-}F}$ = 23.5 Hz, Ar-CH), 78.6 ($CH_2OCH_2$), 39.9 (CH); $^{19}$F{$^1$H} NMR (377 Hz, $CDCl_3$) δ –117.6; HRMS (ESI) *m/z*: [M–H] Calcd for $C_{15}H_{12}OF$ 227.0872; Found 227.0862.

***tert*-Butyl 3-(3-([1,1'-biphenyl]-4-yl)oxetan-3-yl)propanoate (11b), 3-([1,1'-biphenyl]-4-yl)oxetane (S8)** and **di-*tert*-butyl 2-((3-([1,1'-biphenyl]-4-yl)oxetan-3-yl)methyl)pentanedioate (11b')**

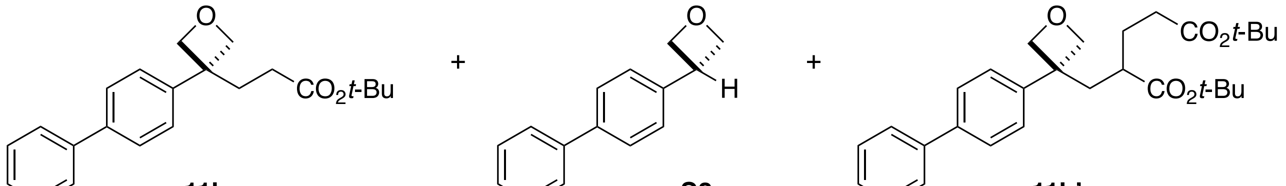


Following **General Procedure A**, 3-([1,1'-biphenyl]-4-yl)oxetane-3-carboxylic acid (50.9 mg) and *t*-Bu-acrylate (45 μL) were used. Purification by basic alumina (IV) column chromatography (10–20% $Et_2O$/pentane) afforded oxetane **S8** as a white solid (13.5 mg, 32%), followed by oxetane **11b** as a white solid (16.5 mg, 24%) and oxetane **11b'** as a colorless oil (4.2 mg, 5%).

Oxetane **S8**: $R_f$ = 0.41 (20% $Et_2O$/pentane); mp = 65–67 °C; IR (film)/cm$^{-1}$ 2952, 2870, 1487, 977, 768; $^1$H NMR (400 MHz, $CDCl_3$) δ 7.64–7.60 (m, 4 H, 4 × Ar-CH), 7.51–7.44 (m, 4 H, 4 × Ar-CH), 7.39–7.35 (m, 1 H, Ph-CH), 5.13 (dd, *J* = 8.5, 6.0 Hz, 2 H, C*H*HOC*H*H), 4.84 (dd, *J* = 6.8, 6.0 Hz, 2 H, CH*H*OCH*H*), 4.30 (tt, *J* = 8.5, 6.8, 1 H, CH); $^{13}$C{$^1$H} NMR (101 MHz, $CDCl_3$) δ 140.7 (Ar-$C_q$), 140.5 (Ar-$C_q$), 140.0 (Ar-$C_q$), 128.8 (2 × Ar-CH), 127.4 (2 × Ar-CH), 127.3 (Ph-CH), 127.2 (2 × Ar-CH), 127.0 (2 × Ar-CH), 78.9 ($CH_2OCH_2$), 40.0 (CH). The observed characterization data ($^1$H, $^{13}$C NMR) were consistent with that previously reported.[34]

Oxetane **11b**: $R_f$ = 0.28 (20% $Et_2O$/pentane); mp = 97–98 °C; IR (film)/cm$^{-1}$ 2933, 1725 (C=O st), 1151, 984; $^1$H NMR (400 MHz, $CDCl_3$) δ 7.61–7.58 (m, 4 H, 4 × Ar-CH), 7.48–7.44 (m, 2 H, 2 × Ar-CH), 7.38–7.34 (m, 1 H, Ph-CH), 7.13–7.10 (m, 2 H, 2 × Ar-CH), 5.02 (d, *J* = 5.7 Hz, 2 H, C*H*HOC*H*H), 4.71 (d, *J* = 5.7 Hz, 2 H, CH*H*OCH*H*), 2.45–2.41 (m, 2 H, C$H_2$$CH_2$), 2.13–2.09 (m, 2 H, $CH_2$C$H_2$), 1.41 (s, 9 H, 3 × $CH_3$); $^{13}$C{$^1$H} NMR (101 MHz, $CDCl_3$) δ 172.4 ($C_q$=O), 143.0 (Ar-$C_q$), 140.6 (Ar-$C_q$), 139.5 (Ar-$C_q$), 128.8 (2 × Ar-CH), 127.31 (Ph-CH), 127.28 (2 × Ar-CH), 127.0 (2 × Ar-CH), 126.2 (2 × Ar-CH), 81.5 ($CH_2OCH_2$), 80.5 ($C_q(CH_3)_3$), 46.7 ($C_q$), 35.9 (C$H_2$$CH_2$), 31.0 ($CH_2$C$H_2$),

28.0 ($C_q(CH_3)_3$); HRMS (ESI) *m/z*: $[M+MeCN+Na]^+$ Calcd for $C_{24}H_{29}NO_3Na$ 402.2045; Found 402.2024.

Oxetane **11b'**: $R_f$ = 0.19 (20% $Et_2O$/pentane); IR (film)/cm$^{-1}$ 2974, 1725 (C=O st), 1148; $^1H$ NMR (400 MHz, $CDCl_3$) δ 7.59–7.56 (m, 4 H, 4 × Ar-CH), 7.47–7.43 (m, 2 H, 2 × Ar-CH), 7.38–7.34 (m, 1 H, Ph-CH), 7.18–7.15 (m, 2 H, 2 × Ar-CH), 4.991 (d, *J* = 5.7 Hz, 1 H, C*H*HOCH$_2$), 4.986 (d, *J* = 5.7 Hz, 1 H, CH$_2$OC*H*H), 4.75 (d, *J* = 5.7 Hz, 1 H, CH*H*OCH$_2$), 4.71 (d, *J* = 5.7 Hz, 1 H, CH$_2$OCH*H*), 2.70 (dd, *J* = 14.5, 10.1 Hz, 1 H, C$_q$C*H*HCH), 2.21–2.03 (m, 4 H, C$_q$CH*H*CH, CH, CHC*H$_2$*CH$_2$), 1.86–1.76 (m, 1 H, CH$_2$C*H*HCO$_2$), 1.68–1.62 (m, 1 H, CH$_2$CH*H*CO$_2$), 1.38 (s, 9 H, 3 × CH$_3$), 1.36 (s, 9 H, 3 × CH$_3$); $^{13}C\{^1H\}$ NMR (101 MHz, $CDCl_3$) δ 174.6 (C$_q$=O), 172.0 (C$_q$=O), 143.2 (Ar-C$_q$), 140.7 (Ar-C$_q$), 139.5 (Ar-C$_q$), 128.8 (2 × Ar-CH), 127.3 (4 × Ar-CH), 127.0 (Ar-CH), 126.5 (2 × Ar-CH), 82.6 (*C*H$_2$OCH$_2$), 81.4 (CH$_2$O*C*H$_2$), 80.7 (*C$_q$*(CH$_3$)$_3$), 80.3 (*C$_q$*(CH$_3$)$_3$), 47.0 (C$_q$), 43.2 (C$_q$*C*H$_2$CH), 42.1 (CH), 32.9 (CH*C*H$_2$CH$_2$), 29.4 (CH$_2$*C*H$_2$CO$_2$), 28.0 (C$_q$(*C*H$_3$)$_3$), 27.9 (C$_q$(*C*H$_3$)$_3$); HRMS (ESI) *m/z*: $[M+Na]^+$ Calcd for $C_{29}H_{38}O_5Na$ 489.2617 Found 489.2618.

Notes:

*The doublets at 4.991 and 4.986 ppm in the $^1H$ NMR spectrum were integrated together to 2 H.*

**Benzyl 3-(3-(3-ethoxy-3-oxopropyl)oxetan-3-yl)-1*H*-indole-1-carboxylate (12a)**

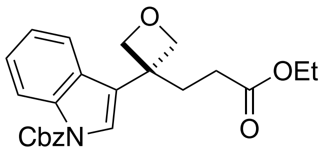


An oven-dried 4 mL vial was charged with 3-(1-((benzyloxy)carbonyl)-1*H*-indol-3-yl)oxetane-3-carboxylic acid (35.1 mg, 0.10 mmol, 1.0 equiv), oven-dried $Cs_2CO_3$ (39.1 mg, 0.12 mmol, 1.2 equiv) and [Ir{dF(CF$_3$)ppy}$_2$(dtbbpy)]PF$_6$ (1.1 mg, 0.001 mmol, 1.0 mol%). The vial was sealed with a screwcap equipped with a PTFE/silicon septum and anhydrous DMF (0.5 mL, 0.2 M) was added by syringe. Argon was bubbled through the mixture for 5 min and ethyl acrylate (18 μL, 0.15 mmol, 1.5 equiv) was added by syringe under argon. After sealing the cap with parafilm on top, the reaction mixture was stirred at 1000 rpm using the set-up shown in Figure S2 and irradiated with two 467 nm Kessel lamps at 36 °C (heat generated by the lamps). After 18 h the lights were switched off and the reaction mixture was transferred into a separating funnel. Distilled water (10 mL) and EtOAc (10 mL) were added, the layers were separated and the aqueous portion was extracted with EtOAc (2 × 10 mL). The organic extracts were combined, dried over $Na_2SO_4$, filtered and concentrated *in vacuo* using a rotatory evaporator. Purification by basic alumina (IV) column chromatography (30–50% $Et_2O$/pentane) afforded oxetane **12a** as a colorless gum (15.4 mg, 38%). $R_f$ = 0.28 (50% $Et_2O$/pentane); IR (film)/cm$^{-1}$ 2951, 1730 (C=O st), 1453, 1396, 1237; $^1H$ NMR (400 MHz, $CDCl_3$) δ 8.22 (br s, 1 H, Ar$_{(indole)}$–CH), 7.52–7.50 (m, 2 H, 2 × Ar-CH), 7.47–7.31 (m, 6 H, 6 × Ar-CH), 7.27–7.23 (m, 1 H, Ar$_{(indole)}$-CH), 5.47 (s, 2 H, CH$_2$Ph), 5.00 (d, *J* = 5.8 Hz, 2 H, C*H*HOC*H*H), 4.78 (d, *J* = 5.8 Hz, 2 H, CH*H*OCH*H*), 4.02 (q, *J* = 7.2 Hz, 2 H, C*H$_2$*CH$_3$), 2.58–2.54 (m, 2 H, C*H$_2$*CH$_2$), 2.19–2.15 (m, 2 H, CH$_2$C*H$_2$*), 1.17 (t, *J* = 7.2 Hz, 3 H, CH$_2$C*H$_3$*); $^{13}C\{^1H\}$ NMR (101 MHz, $CDCl_3$) δ 172.9 (C$_q$=O$_{(ester)}$), 150.7 (C$_q$=O$_{(carbamate)}$), 136.0 (Ar-C$_q$), 134.9 (Ph-C$_q$), 128.82 (Ar-CH), 128.80 (2 × Ar-CH), 128.6 (2 × Ar-CH), 128.2 (Ar-C$_q$), 125.0 (Ar-CH), 123.5 (Ar-*C$_q$*C$_q$), 123.0 (Ar-CH), 122.1 (Ar-CH), 119.5 (Ar-CH), 115.7 (Ar$_{(indole)}$-CH), 81.2 (CH$_2$OCH$_2$), 68.9 (CH$_2$Ph), 60.5 (*C*H$_2$CH$_3$), 41.9 (C$_q$), 32.9 (*C*H$_2$CH$_2$), 30.1 (CH$_2$*C*H$_2$), 14.1 (CH$_2$*C*H$_3$); HRMS (ESI) *m/z*: $[M+H]^+$ Calcd for $C_{24}H_{26}NO_5$ 408.1811; Found 408.1818.

Notes:

*The signals at 150.7 and 136.0 ppm in the $^{13}C$ NMR spectrum are very weak due to chemical exchange between carbamate rotamers. They were assigned based on HMBC cross correlation peaks (page S128).*

3-Aryl-3-Alkyl-Azetidines (**S9**, **13a**, **13b**, **13m**, **13n**, **13p**, **13q**, **13t**)

General notes:

*Hindered rotation around the C–N bond of the azetidine carbamate (Cbz) led, in some examples, to a very broad peak (sometimes 2 peaks) of the azetidine $CH_2$ signals in the $^{13}C$ NMR spectrum. In such cases, an HMBC correlation spectrum provided evidence for the chemical shift of the azetidine $CH_2$ signals.*

**Benzyl 3-(4-methoxyphenyl)azetidine-1-carboxylate (S9)**

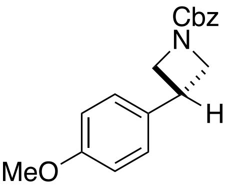

**S9** was isolated in the reaction of the 1-((benzyloxy)carbonyl)-3-(4-methoxyphenyl)azetidine-3-carboxylic acid with cyclopentene-1-one. No alkylated azetidine product was observed.

Following **General Procedure A**, 1-((benzyloxy)carbonyl)-3-(4-methoxyphenyl)azetidine-3-carboxylic acid (68.3 mg) and 2-cyclopenten-1-one (25 μL) were used. Purification by silica flash column chromatography (40–70% $Et_2O$/pentane) afforded reduced azetidine **S9** as a yellow oil (5.3 mg, 9%). $R_f$ = 0.36 (40% $Et_2O$/pentane); IR (film)/cm$^{-1}$ 2960, 1707 (C=O st), 1513, 1413, 1249, 1126; $^1$H NMR (400 MHz, $CDCl_3$) δ 7.40–7.39 (m, 5 H, 5 × Ar-CH), 7.26–7.22 (m, 2 H, 2 × Ar-CH), 6.91–6.88 (m, 2 H, 2 × Ar-CH), 5.14 (s, 2 H, $CH_2$Ph), 4.40 (dd, *J* = 8.7, 8.7 Hz, 2 H, C*H*HNC*H*H), 4.03 (dd, *J* = 8.7, 6.1 Hz, 2 H, CH*H*NCH*H*), 3.81 (s, 3 H, $OCH_3$), 3.75 (tt, *J* = 8.7, 6.1 Hz, 1 H, CH); $^{13}$C{$^1$H} NMR (101 MHz, $CDCl_3$) δ 158.6 (Ar-$C_q$OMe), 156.4 ($C_q$=O), 136.7 (Ar-$C_q$$CH_2$), 133.9 (Ar-$C_qC_q$), 128.5 (2 × Ar-CH), 128.02 (Ph-CH), 127.98 (2 × Ar-CH), 127.8 (2 × Ar-CH), 114.1 (2 × Ar-CH), 66.6 ($CH_2$Ph), 56.9 (br, $CH_2NCH_2$), 55.3 ($OCH_3$), 33.3 (CH); HRMS (ESI) *m/z*: $[M+H]^+$ Calcd for $C_{18}H_{20}NO_3$ 298.1443; Found 298.1449.

Notes:

*In another occasion,* ***S9*** *crystallized in the freezer to white crystals (mp = 76–77 °C).*

**Benzyl 3-(3-ethoxy-3-oxopropyl)-3-(4-methoxyphenyl)azetidine-1-carboxylate (13a, IV-A)** and **diethyl 2-((1-((benzyloxy)carbonyl)-3-(4-methoxyphenyl)azetidin-3-yl)methyl)pentanedioate (13a', IV-A')**

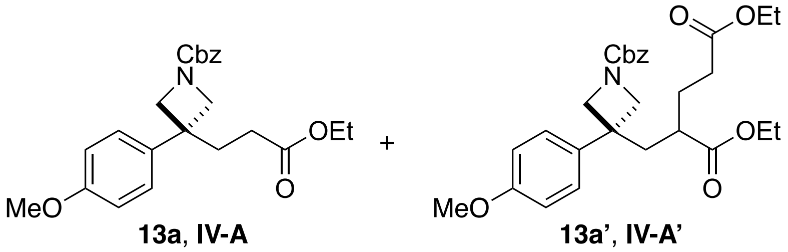

**13a**, **IV-A** + **13a'**, **IV-A'**

Following **General Procedure A**, 1-((benzyloxy)carbonyl)-3-(4-methoxyphenyl)azetidine-3-carboxylic acid (68.3 mg) and ethyl acrylate (33 μL) were used. Purification by silica flash column chromatography (40% $Et_2O$/pentane) afforded azetidine **13a** as a colorless oil (42.5 mg, 53%), followed by azetidine **13a'** as a colorless oil (11.7 mg, 12%).

Azetidine **13a** (**IV-A**): $R_f$ = 0.50 (70% $Et_2O$/pentane); IR (film)/cm$^{-1}$ 2954, 1705 (C=O st), 1515, 1414, 1354, 1246, 1179, 1115, 1029; $^1$H NMR (400 MHz, $CDCl_3$) δ 7.30–7.22 (m, 5 H, 5 × Ar-CH), 6.94–6.90 (m, 2 H, 2 × Ar-CH), 6.82–6.78z (m, 2 H, 2 × Ar-CH), 5.02 (s, 2 H, $CH_2$Ph), 4.17 (d, *J* = 8.3 Hz, 2 H, C*H*HNC*H*H), 3.97 (q, *J* = 7.2 Hz, 2 H, C*$H_2$*$CH_3$), 3.95 (d, *J* = 8.3 Hz, 2 H, CH*H*NCH*H*), 3.72 (s, 3 H, $OCH_3$), 2.20–2.15 (m, 2 H, C*$H_2$*$CH_2$), 2.03–1.99 (m, 2 H, $CH_2$C*$H_2$*), 1.13 (t, *J* = 7.2 Hz, 3 H, $CH_2$C*$H_3$*); $^{13}$C{$^1$H} NMR (101 MHz, $CDCl_3$) δ 172.8 ($C_q$=$O_{(ester)}$), 158.3 (Ar-$C_q$OMe), 156.5 ($C_q$=$O_{(carbamate)}$), 136.6 (Ar-$C_q$$CH_2$), 135.5 (Ar-$C_qC_q$), 128.4 (2 × Ar-CH), 128.0 (Ph-CH), 127.9 (2 × Ar-CH), 127.0 (2 × Ar-CH), 114.0 (2 × Ar-CH), 66.7 ($CH_2$Ph), 60.5 (*C*$H_2CH_3$), 59.7 (br, $CH_2NCH_2$), 55.3 ($OCH_3$), 41.3 ($C_q$), 36.9 (*C*$H_2CH_2$), 29.8 ($CH_2$*C*$H_2$), 14.1 ($CH_2CH_3$); HRMS (ESI) *m/z*: $[M+H]^+$ Calcd for $C_{23}H_{28}NO_5^+$ 398.1962; Found 398.1963.

Notes:

*The quartet at 3.97 and doublet at 3.95 ppm in the $^1H$ NMR spectrum were integrated together to 4 H*

Azetidine **13a'** (**IV-A'**): $R_f$ = 0.43 (70% $Et_2O$/pentane); IR (film)/$cm^{-1}$ 2956, 1707 (C=O st), 1416, 1249, 1178, 1118; $^1H$ NMR (400 MHz, $CDCl_3$) δ 7.38–7.30 (m, 5 H, 5 × Ar-CH), 7.04–7.00 (m, 2 H, 2 × Ar-CH), 6.98–6.84 (m, 2 H, 2 × Ar-CH), 5.08 (s, 2 H, $CH_2Ph$), 4.28 (d, *J* = 8.3 Hz, 1 H, C*H*HNC$H_2$), 4.20 (d, *J* = 8.3 Hz, 1 H, $CH_2$NC*H*H), 4.08 (q, *J* = 7.1 Hz, 2 H, C*H*$_2$CH$_3$), 4.01 (d, *J* = 8.3 Hz, 2 H, CH*H*NCH*H*), 3.95–3.82 (m, 2 H, C*H*$_2$CH$_3$), 3.80 (s, 3 H, $OCH_3$), 2.50 (dd, *J* = 14.0, 9.6 Hz, 1 H, $C_q$C*H*HCH), 2.19–2.14 (m, 3 H, CH + CHC*H*$_2$CH$_2$), 1.97 (dd, *J* = 14.0, 3.1 Hz, 1 H, $C_q$CH*H*CH), 1.85–1.76 (m, 1 H, $CH_2$C*H*HCO$_2$), 1.71–1.66 (m, 1 H, $CH_2$CH*H*CO$_2$), 1.21 (t, *J* = 7.2 Hz, 3 H, $CH_2$C*H*$_3$), 1.17 (t, *J* = 7.1 Hz, 3 H, $CH_2$C*H*$_3$); $^{13}C\{^1H\}$ NMR (101 MHz, $CDCl_3$) δ 174.9 ($C_q$=$O_{(ester)}$), 172.5 ($C_q$=$O_{(ester)}$), 158.2 (Ar-$C_q$OMe), 156.4 ($C_q$=$O_{(carbamate)}$), 136.5 (Ar-*C*$_q$CH$_2$), 135.4 (Ar-*C*$_q$C$_q$), 128.4 (2 × Ar-CH), 128.01 (Ph-CH), 127.95 (2 × Ar-CH), 127.4 (2 × Ar-CH), 113.8 (2 × Ar-CH), 66.7 ($CH_2Ph$), 60.5 (*C*H$_2$CH$_3$), 60.4 (*C*H$_2$CH$_3$), 59.5 (br, $CH_2NCH_2$), 55.3 ($OCH_3$), 44.2 ($C_q$), 41.5 ($C_q$*C*H$_2$CH), 41.3 (CH), 31.6 (CH*C*H$_2$CH$_2$), 28.8 ($CH_2$*C*H$_2$CO$_2$), 14.1 ($CH_2$*C*H$_3$), 14.0 ($CH_2$*C*H$_3$); HRMS (ESI) *m/z*: $[M+Na]^+$ Calcd for $C_{28}H_{35}NO_7Na$ 520.2311; Found 520.2324.

**Benzyl 3-(3-(tert-butoxy)-3-oxopropyl)-3-(4-methoxyphenyl)azetidine-1-carboxylate (13b)** and **di-*tert*-butyl 2-((1-((benzyloxy)carbonyl)-3-(4-methoxyphenyl)azetidin-3-yl)methyl)pentanedioate (13b')**

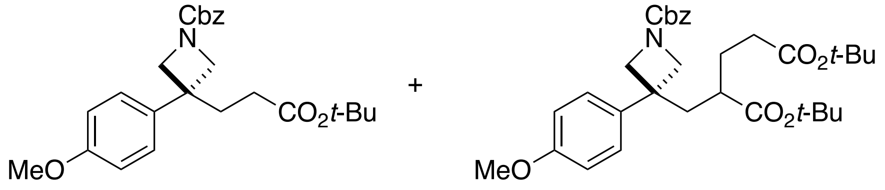


**13b** **13b'**

Following **General Procedure A**, 1-((benzyloxy)carbonyl)-3-(4-methoxyphenyl)azetidine-3-carboxylic acid (68.3 mg) and *t*-Bu-acrylate (45 μL) were used. Purification by silica column chromatography (3–4% $Et_2O$/$CH_2Cl_2$) afforded azetidine **13b** as a colorless oil (41.3 mg, 49%), followed by azetidine **13b'** as a pink oil (15.3 mg, 14%).

Azetidine **13b**: $R_f$ = 0.39 (4% $Et_2O$/$CH_2Cl_2$); IR (film)/$cm^{-1}$ 2956, 1707 (C=O st), 1416, 1249, 1148; $^1H$ NMR (400 MHz, $CDCl_3$) δ 7.28–7.22 (m, 5 H, 5 × Ar-CH), 6.93–6.90 (m, 2 H, 2 × Ar-CH), 6.82–6.78 (m, 2 H, 2 × Ar-CH), 5.02 (s, 2 H, $CH_2Ph$), 4.16 (d, *J* = 8.2 Hz, 2 H, C*H*HNC*H*H), 3.96 (d, *J* = 8.2 Hz, 2 H, CH*H*NCH*H*), 3.72 (s, 3 H, $OCH_3$), 2.14–2.10 (m, 2 H, C*H*$_2$CH$_2$), 1.95–1.91 (m, 2 H, $CH_2$C*H*$_2$), 1.31 (s, 9 H, 3 × $CH_3$); $^{13}C\{^1H\}$ NMR (101 MHz, $CDCl_3$) δ 172.3 ($C_q$=$O_{(ester)}$), 158.2 (Ar-$C_q$OMe), 156.5 ($C_q$=$O_{(carbamate)}$), 136.5 (Ph-*C*$_q$CH$_2$), 135.7 (Ar-*C*$_q$C$_q$), 128.4 (2 × Ar-CH), 128.0 (Ph-CH), 127.9 (2 × Ar-CH), 127.0 (2 × Ar-CH), 113.9 (2 × Ar-CH), 80.5 (*C*$_q$(CH$_3$)$_3$), 66.7 ($CH_2Ph$), 60.2 and 59.3 (br, $CH_2NCH_2$), 55.3 ($OCH_3$), 41.3 ($C_q$), 36.9 (*C*H$_2$CH$_2$), 30.9 ($CH_2$*C*H$_2$), 28.0 ($C_q$(*C*H$_3$)$_3$); HRMS (ESI) *m/z*: $[M+H]^+$ Calcd for $C_{25}H_{32}NO_5$ 426.2280; Found 426.2284.

Azetidine **13b'**: $R_f$ = 0.23 (4% $Et_2O$/$CH_2Cl_2$); IR (film)/$cm^{-1}$ 2974, 1715 (C=O st), 1249, 1144; $^1H$ NMR (400 MHz, $CDCl_3$) δ 7.35–7.30 (m, 5 H, 5 × Ar-CH), 7.05–7.02 (m, 2 H, 2 × Ar-CH), 6.89–6.85 (m, 2 H, 2 × Ar-CH), 5.08 (s, 2 H, $CH_2Ph$), 4.24 (d, *J* = 8.3 Hz, 1 H, C*H*HNC$H_2$), 4.22 (d, *J* = 8.2 Hz, 1 H, $CH_2$NC*H*H), 4.07 (d, *J* = 8.3 Hz, 1 H, CH*H*NC$H_2$), 4.02 (d, *J* = 8.2 Hz, 1 H, $CH_2$NCH*H*), 3.80 (s, 3 H, $OCH_3$), 2.47 (dd, *J* = 14.0, 9.4 Hz, 1 H, $C_q$C*H*HCH), 2.17–2.05 (m, 2 H, CHC*H*$_2$CH$_2$), 2.03–1.96 (m, 1 H, CH), 1.84 (dd, *J* = 14.0, 3.0 Hz, 1 H, $C_q$CH*H*CH), 1.78–1.69 (m, 1 H, $CH_2$C*H*HCO$_2$), 1.61–1.52 (m, 1 H, $CH_2$CH*H*CO$_2$), 1.38 (s, 9 H, 3 × $CH_3$), 1.37 (s, 9 H, 3 × $CH_3$); $^{13}C\{^1H\}$ NMR (101 MHz, $CDCl_3$) δ 174.5 ($C_q$=$O_{(ester)}$), 171.9 ($C_q$=$O_{(ester)}$), 158.2 (Ar-$C_q$OMe), 156.4 ($C_q$=$O_{(carbamate)}$), 136.6 (Ar-*C*$_q$CH$_2$), 135.9 (Ar-*C*$_q$C$_q$), 128.4 (2 × Ar-CH), 128.0 (Ph-CH), 127.9 (2 × Ar-CH), 127.3 (2 × Ar-CH), 113.9 (2 × Ar-CH), 80.7 (*C*$_q$(CH$_3$)$_3$), 80.3 (*C*$_q$(CH$_3$)$_3$), 66.6 ($CH_2Ph$), 61.4 and 59.7 (br, $CH_2NCH_2$), 55.3 ($OCH_3$), 44.2 ($C_q$*C*H$_2$CH), 42.2 (CH), 41.6 ($C_q$), 32.9 (CH*C*H$_2$CH$_2$), 29.3 ($CH_2$*C*H$_2$CO$_2$), 28.0 ($C_q$(*C*H$_3$)$_3$), 27.9 ($C_q$(*C*H$_3$)$_3$); HRMS (ESI) *m/z*: $[M+Na]^+$ Calcd for $C_{32}H_{43}NO_7Na$ 576.2937; Found 576.2935.

Notes:

*The singlets at 1.38 and 1.37 ppm in the $^1H$ NMR spectrum were integrated together to 18 H.*

*The doublets at 4.24 and 4.22 ppm in the $^1H$ NMR spectrum were integrated together to 2 H.*

**Dimethyl 2-(1-((benzyloxy)carbonyl)-3-(4-methoxyphenyl)azetidin-3-yl)succinate (13m)**

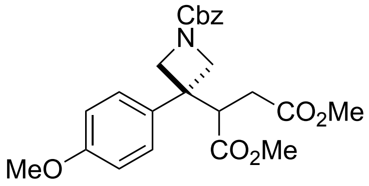


Following **General Procedure B**, 1-((benzyloxy)carbonyl)-3-(4-methoxyphenyl)azetidine-3-carboxylic acid (68.3 mg) and dimethyl fumarate (43.2 mg) were used. Purification by silica flash column chromatography (50% $Et_2O$/pentane) afforded azetidine **13m** as a colorless oil (43.2 mg, 49%). $R_f$ = 0.14 (50% $Et_2O$/pentane); IR (film)/$cm^{-1}$ 2952, 1733 (C=O st), 1710 (C=O st), 1513, 1416, 1357, 1252, 1166; $^1H$ NMR (400 MHz, $CDCl_3$) δ 7.39–7.29 (m, 5 H, 5 × Ar-CH), 6.97–6.93 (m, 2 H, 2 × Ar-CH), 6.88–6.84 (m, 2 H, 2 × Ar-CH), 5.09 (s, 2 H, $CH_2Ph$), 4.58 (d, *J* = 8.8 Hz, 1 H, C*H*HNCH$_2$), 4.31 (d, *J* = 8.8 Hz, 1 H, $CH_2$NC*H*H), 4.27 (s, 2 H, CH*H*NCH*H*), 3.80 (s, 3 H, $OCH_3$), 3.64 (s, 3 H, $CO_2CH_3$), 3.63 (s, 3 H, $CO_2CH_3$), 3.41 (dd, *J* = 11.5, 3.1 Hz, 1 H, CH), 2.61 (dd, *J* = 16.9, 11.5 Hz, 1 H, CHC*H*H), 2.32 (dd, *J* = 16.9, 3.1 Hz, 1 H, CHCH*H*); $^{13}C\{^1H\}$ NMR (101 MHz, $CDCl_3$) δ 172.2 ($C_q$=$O_{(ester)}$), 172.0 ($C_q$=$O_{(ester)}$), 158.6 (Ar-$C_q$OMe), 156.2 ($C_q$=$O_{(carbamate)}$), 136.4 (Ar-$C_q$CH$_2$), 133.4 (Ar-$C_qC_q$), 128.5 (2 × Ar-CH), 128.1 (Ph-CH), 128.0 (2 × Ar-CH), 127.7 (2 × Ar-CH), 113.9 (2 × Ar-CH), 66.8 ($CH_2Ph$), 58.8 (br, $CH_2NCH_2$), 55.3 ($OCH_3$), 52.0 ($CO_2CH_3$), 51.9 ($CO_2CH_3$), 49.6 (CH), 43.3 ($C_q$), 32.0 (CH*C*H$_2$); HRMS (ESI) *m/z*: $[M+H]^+$ Calcd for $C_{24}H_{28}NO_7$ 442.1866; Found 442.1854.

Notes:

*The singlets at 3.64 and 3.63 ppm in the $^1H$ NMR spectrum were integrated together to 6 H.*

**Benzyl 3-(4-methoxyphenyl)-3-phenethylazetidine-1-carboxylate (13n)** and **benzyl 3-(4-methoxyphenyl)azetidine-1-carboxylate (S9)**

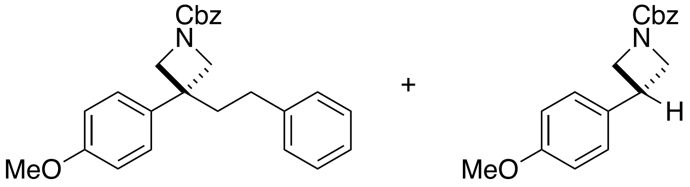


**13n** **S9**

Following **General Procedure A**, 1-((benzyloxy)carbonyl)-3-(4-methoxyphenyl)azetidine-3-carboxylic acid (68.3 mg) and styrene (35 μL) were used. Purification by silica flash column chromatography (2% $Et_2O/CH_2Cl_2$) afforded azetidine **13n** as a yellow oil (27.2 mg, 34%), followed by azetidine **S9** as a colorless oil (2.1 mg, 4%).

Azetidine **13n**: $R_f$ = 0.50 (2% $Et_2O/CH_2Cl_2$); IR (film)/$cm^{-1}$ 2952, 1707 (C=O st), 1513, 1416, 1357, 1249, 1118, 1033; $^1H$ NMR (400 MHz, $CDCl_3$) δ 7.37–7.30 (m, 5 H, 5 × Ar-CH), 7.25–7.23 (m, 2 H, 2 × Ar-CH), 7.19–7.15 (m, 1 H, Ph-CH), 7.09–7.07 (m, 4 H, 4 × Ar-CH), 6.94–6.90 (m, 2 H, 2 × Ar-CH), 5.11 (s, 2 H, $CH_2Ph$), 4.28 (d, *J* = 8.2 Hz, 2 H, C*H*HNC*H*H), 4.08 (d, *J* = 8.2 Hz, 2 H, CH*H*NCH*H*), 3.84 (s, 3 H, $OCH_3$), 2.40–2.36 (m, 2 H, C*H$_2$*CH$_2$), 2.24–2.20 (m, 2 H, CH$_2$C*H$_2$*); $^{13}C\{^1H\}$ NMR (101 MHz, $CDCl_3$) δ 158.1 (Ar-$C_q$OMe), 156.5 ($C_q$=O), 141.5 (Ar-*C$_q$*CH$_2$CH$_2$), 136.6 (Ar-*C$_q$*CH$_2$O), 136.3 (Ar-*C$_q$*C$_q$), 128.5 (2 × Ar-CH), 128.4 (2 × Ar-CH), 128.2 (2 × Ar-CH), 128.02 (Ph-CH), 127.96 (2 × Ar-CH), 127.0 (2 × Ar-CH), 125.9 (Ph-CH), 113.9 (2 × Ar-CH), 66.7 ($CH_2Ph$), 59.6 (br, $CH_2NCH_2$), 55.3 ($OCH_3$), 44.2 (*C*H$_2$CH$_2$), 41.9 ($C_q$), 30.9 (CH$_2$*C*H$_2$); HRMS (APCI) *m/z*: $[M+H]^+$ Calcd for $C_{26}H_{28}NO_3^+$ 402.2064; Found 402.2053.

**Benzyl 3-(4-methoxyphenyl)-3-(2-(1-oxo-1,3-dihydroisobenzofuran-5-yl)ethyl)azetidine-1-carboxylate (13p)**

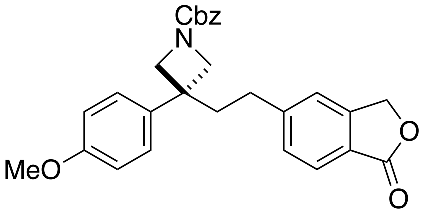


Following **General Procedure B**, 1-((benzyloxy)carbonyl)-3-(4-methoxyphenyl)azetidine-3-carboxylic acid (68.3 mg) and 5-vinylisobenzofuran-1(3*H*)-one (48.1 mg) were used. Purification by silica flash column chromatography (80% $Et_2O$/pentane) afforded azetidine **13p** as a white solid (51.3 mg, 56%). $R_f$ = 0.19 (80% $Et_2O$/pentane); mp = 45–49 °C; IR (film)/cm$^{-1}$ 2952, 1763 (C=O st), 1707 (C=O st), 1513, 1416, 1357, 1249, 1118, 1044; $^1$H NMR (400 MHz, $CDCl_3$) δ 7.79 (d, *J* = 7.8 Hz, 1 H, Ar-CH), 7.37–7.30 (m, 5 H, 5 × Ar-CH), 7.23 (dd, *J* = 7.8, 1.3 Hz, 1 H, Ar-CH), 7.18 (br s, 1 H, Ar-CH), 7.08–7.05 (m, 2 H, 2 × Ar-CH), 6.94–6.90 (m, 2 H, 2 × Ar-CH), 5.24 (s, 2 H, $CH_2OCO$), 5.11 (s, 2 H, $CH_2Ph$), 4.31 (d, *J* = 8.3 Hz, 2 H, C*H*HNC*H*H), 4.07 (d, *J* = 8.3 Hz, 2 H, CH*H*NCH*H*), 3.83 (s, 3 H, $OCH_3$), 2.51 (dd, *J* = 11.1, 5.8 Hz, 2 H, $CH_2CH_2$), 2.24 (dd, *J* = 11.1, 6.2 Hz, 2 H, $CH_2CH_2$); $^{13}$C{$^1$H} NMR (101 MHz, $CDCl_3$) δ 170.9 ($C_q$=$O_{(lactone)}$), 158.3 (Ar-$C_q$OMe), 156.6 ($C_q$=$O_{(carbamate)}$), 148.7 (Ar-$C_q$CO), 147.1 (Ar-$C_q$$CH_2O$), 136.5 (Ar-$C_qCH_{2(Cbz)}$), 135.8 (Ar-$C_qC_q$), 129.5 (Ar-CH), 128.5 (2 × Ar-CH), 128.1 (Ar-CH), 128.0 (2 × Ar-CH), 127.0 (2 × Ar-CH), 125.8 (Ar-CH), 123.7 (Ar-$C_qCH_2CH_2$), 121.6 (Ar-CH), 114.1 (2 × Ar-CH), 69.3 ($CH_2OCO_{(lactone)}$), 66.8 ($CH_2Ph$), 60.3 (br, $CH_2NCH_2$), 55.3 ($OCH_3$), 44.0 ($CH_2CH_2$), 41.9 ($C_q$), 31.5 ($CH_2CH_2$); HRMS (APCI) *m/z*: $[M+H]^+$ Calcd for $C_{28}H_{28}NO_5^+$ 458.1962; Found 458.1959.

**Benzyl 3-(4-methoxyphenyl)-3-(2-(pyridin-4-yl)ethyl)azetidine-1-carboxylate (13q)**

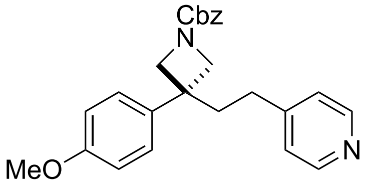


Following **General Procedure A**, 1-((benzyloxy)carbonyl)-3-(4-methoxyphenyl)azetidine-3-carboxylic acid (68.3 mg) and 4-vinylpyridine (35 μL) were used. Purification by silica flash column chromatography (100% $Et_2O$) afforded azetidine **13q** as a colorless oil (62.5 mg, 78%). $R_f$ = 0.13 (100% $Et_2O$); IR (film)/cm$^{-1}$ 2952, 1707 (C=O st), 1603, 1513, 1416, 1357, 1249; $^1$H NMR (400 MHz, $CDCl_3$) δ 8.46–8.45 (m, 2 H, 2 × $Ar_{(pyridine)}$-CH), 7.37–7.30 (m, 5 H, 5 × Ar-CH), 7.08–7.04 (m, 2 H, 2 × Ar-CH), 7.01–7.00 (m, 2 H, 2 × $Ar_{(pyridine)}$-CH), 6.94–6.90 (m, 2 H, 2 × Ar-CH), 5.11 (s, 2 H, $CH_2Ph$), 4.30 (d, *J* = 8.2 Hz, 2 H, C*H*HNC*H*H), 4.07 (d, *J* = 8.2 Hz, 2 H, CH*H*NCH*H*), 3.83 (s, 3 H, $OCH_3$), 2.39–2.35 (m, 2 H, $CH_2CH_2$), 2.23–2.19 (m, 2 H, $CH_2CH_2$); $^{13}$C{$^1$H} NMR (101 MHz, $CDCl_3$) δ 158.3 (Ar-$C_q$OMe), 156.5 ($C_q$=O), 150.3 ($Ar_{(pyridine)}$-$C_q$), 149.7 (2 × $Ar_{(pyridine)}$-CH), 136.5 (Ar-$C_qCH_2$), 135.8 (Ar-$C_qC_q$), 128.5 (2 × Ar-CH), 128.1 (Ph-CH), 128.0 (2 × Ar-CH), 127.0 (2 × Ar-CH), 123.6 (2 × $Ar_{(pyridine)}$-CH), 114.0 (2 × Ar-CH), 66.7 ($CH_2Ph$), 59.4 (br, $CH_2NCH_2$), 55.3 ($OCH_3$), 42.9 ($CH_2CH_2$), 41.9 ($C_q$), 30.3 ($CH_2CH_2$); HRMS (ESI) *m/z*: $[M+H]^+$ Calcd for $C_{25}H_{27}N_2O_3$ 403.2022; Found 403.2028.

**Benzyl 3-(2-(4-bromophenyl)-2-(pyridin-2-yl)ethyl)-3-(4-methoxyphenyl)azetidine-1-carboxylate (13t)**

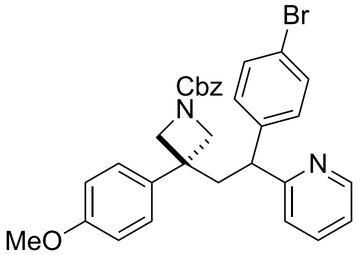


Following **General Procedure B**, 1-((benzyloxy)carbonyl)-3-(4-methoxyphenyl)azetidine-3-carboxylic acid (68.3 mg) and 2-(1-(4-bromophenyl)vinyl)pyridine (78.0 mg) were used. Purification by silica flash column chromatography ($Et_2O$) afforded azetidine **13t** as a bright yellow solid (76.3 mg, 68%). $R_f$ = 0.18 ($Et_2O$); mp = 51–55 °C; IR (film)/cm$^{-1}$ 2956, 1707 (C=O st), 1513, 1420, 1249; $^1$H NMR (400 MHz, $CDCl_3$) δ 8.41 (dd, *J* = 4.8, 1.6 Hz, 1 H, $Ar_{(pyridine)}$-CH), 8.32 (d, *J* = 2.3 Hz, 1 H, $Ar_{(pyridine)}$-CH), 7.41–7.30 (m, 8 H, 5 × Ph-CH + 2 × $Ar_{(p\text{-}Br)}$-CH + $Ar_{(pyridine)}$-CH), 7.15 (dd, *J* = 8.0, 4.8 Hz, 1 H, $Ar_{(pyridine)}$-CH), 7.00–6.97 (m, 4 H, 2 × $Ar_{(p\text{-}Br)}$-CH + 2 × $Ar_{(PMP)}$-CH), 6.90–6.86 (m, 2 H, 2 × $Ar_{(PMP)}$-CH), 5.05 (s, 2 H, $CH_2Ph$), 4.09 (d, *J* = 8.5 Hz, 1 H, C*H*HNC$H_2$), 4.07 (d, *J* = 8.5 Hz, 1 H, $CH_2$NC*H*H), 3.83 (s, 3 H, $OCH_3$), 3.81 (d, *J* = 8.5 Hz, 1 H, CH*H*NC$H_2$), 3.79 (d, *J* = 8.5 Hz, 1 H, $CH_2$NCH*H*), 3.59 (dd, *J* = 7.6, 6.8 Hz, 1 H, CH), 2.80 (dd, *J* = 13.6, 7.6 Hz, 1 H, C*H*H), 2.72 (dd, *J* = 13.6, 6.8 Hz, 1 H, CH*H*); $^{13}$C{$^1$H} NMR (101 MHz, $CDCl_3$) δ 158.2 (Ar-$C_q$OMe), 156.4 ($C_q$=O), 149.3 ($Ar_{(pyridine)}$-CH), 148.0 ($Ar_{(pyridine)}$-CH), 142.2 ($Ar_{(p\text{-}Br)}$-$C_q$CH), 139.3 ($Ar_{(pyridine)}$-$C_q$CH), 136.5 (Ph-$C_q$$CH_2$), 135.4 (Ar-$C_q$$C_q$), 134.8 ($Ar_{(pyridine)}$-CH), 131.9 (2 × $Ar_{(p\text{-}Br)}$-CH), 129.4 (2 × $Ar_{(p\text{-}Br)}$-CH), 128.5 (2 × Ph-CH), 128.1 (Ph-CH), 128.0 (2 × Ph-CH), 127.3 (2 × Ar-CH), 123.5 ($Ar_{(pyridine)}$-CH), 120.7 (Ar-$C_q$Br), 114.1 (2 × Ar-CH), 66.7 ($CH_2Ph$), 60.8 (br, $CH_2NCH_2$), 55.3 ($OCH_3$), 46.8 (*C*$H_2$CH), 45.0 (CH), 41.8 ($C_q$); HRMS (ESI) *m/z*: $[M+H]^+$ Calcd for $C_{31}H_{30}N_2O_3{}^{79}Br$ 557.1440; Found 557.1448.

*The doublets at 4.09 and 4.07 ppm and the doublets at 3.81 and 3.79 ppm in the $^1$H NMR spectrum were integrated together to 2 H and 2 H, respectively.*

Oxetane and Azetidine Derivatization Products (**14–16**)

**Ethyl 3-(3-(4-methoxyphenyl)azetidin-3-yl)propanoate (14)**

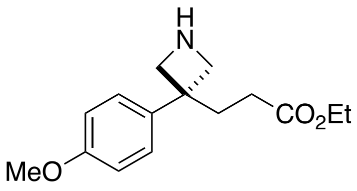


Palladium on carbon (10% *w/w*, 2.0 mg, 10 mol% Pd) was added to a solution of *N*-Cbz azetidine **13a** (73.0 mg, 0.184 mmol, 1.0 equiv) in EtOH (0.9 mL, 0.2 M) under $N_2$. The reaction mixture was sparged with $H_2$ for 5 min and then left to stir under an atmosphere of $H_2$ for 22 h. The reaction mixture was filtered through Celite, eluting with further EtOH (10 mL) and then concentrated *in vacuo* using a rotatory evaporator to afford free azetidine **14** as a colorless oil (36.7 mg, 76%). $R_f$ = 0.19 (3% MeOH/$CH_2Cl_2$ + 1% $NEt_3$); IR (film)/cm$^{-1}$ 3450 (NH st, br), 2928, 2634, 2088, 1726 (C=O st), 1515, 1246 1180, 1029; $^1$H NMR (400 MHz, $CDCl_3$) δ 6.99–6.95 (m, 2 H, 2 × Ar-CH), 6.88–6.84 (m, 2 H, 2 × Ar-CH), 4.04 (q, *J* = 7.2 Hz, 2 H, C*H*$_2$CH$_3$), 3.96 (d, *J* = 7.6 Hz, 2 H, C*H*HNC*H*H), 3.80 (s, 3 H, $OCH_3$), 3.60 (d, *J* = 7.6 Hz, 2 H, CH*H*NCH*H*), 2.37–2.29 (m, 2 H, C*H*$_2$CH$_2$), 2.12–2.08 (m, 2 H, CH$_2$C*H*$_2$), 1.98 (br s, 1 H, NH), 1.20 (t, *J* = 7.2 Hz, 3 H, CH$_2$C*H*$_3$); $^{13}$C{$^1$H} NMR (101 MHz, $CDCl_3$) δ 173.4 ($C_q$=O), 157.8 (Ar-$C_q$OMe), 138.0 (Ar-$C_qC_q$), 126.7 (2 × Ar-CH), 113.7 (2 × Ar-CH), 60.3 (*C*H$_2$CH$_3$), 57.3 ($CH_2NCH_2$), 55.2 ($OCH_3$), 45.8 ($C_q$), 36.4 (*C*H$_2$CH$_2$), 30.0 (CH$_2$*C*H$_2$), 14.1 (CH$_2$*C*H$_3$); HRMS (ESI) *m/z*: $[M+H]^+$ Calcd for $C_{15}H_{22}NO_3$ 264.1600; Found 264.1603.

**3-(3-(4-Methoxyphenyl)oxetan-3-yl)propanoic acid (15)**

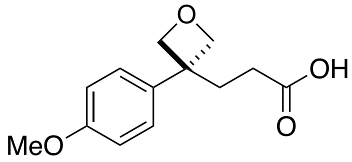


LiOH (7.2 mg, 0.3 mmol, 3.0 equiv) was added to a solution of PMP oxetane ethyl ester **2a** (26.4 mg, 0.1 mmol, 1.0 equiv) in a THF (0.2 mL) / MeOH (0.2 mL) / $H_2O$ (0.6 mL) mixture (THF:MeOH:$H_2O$ = 1:1:3, 0.1 M) at 25 °C under air. After stirring the reaction mixture at 25 °C for 24 h, distilled water (15 mL) was added. The aqueous solution was extracted with $Et_2O$ (2 × 10 mL), acidified to pH 1 with 1 M aq. HCl and re-extracted with EtOAc (3 × 15 mL). The EtOAc layers were combined, dried over $Na_2SO_4$, filtered and concentrated *in vacuo* using a rotatory evaporator to afford oxetane acid **15** as colorless crystals (23.4 mg, 99%). $R_f$ = 0.09 (50% EtOAc/pentane); mp = 104 °C; IR (film)/cm$^{-1}$ 3029 (COOH st, H-bonded, br), 2935, 1729 (C=O st, H-bonded), 1708, 1514, 1248, 831; $^1$H NMR (400 MHz, $CDCl_3$) δ 6.95 (d, *J* = 8.2 Hz, 2 H, 2 × Ar-CH), 6.88 (d, *J* = 8.2 Hz, 2 H, 2 × Ar-CH), 4.97 (d, *J* = 5.7 Hz, 2 H, C*H*HOC*H*H), 4.65 (d, *J* = 5.7 Hz, 2 H, CH*H*OCH*H*), 3.80 (s, 3 H, $OCH_3$), 2.41 (t, *J* = 8.0 Hz, 2 H, C*H*$_2$CH$_2$), 2.17 (t, *J* = 8.0 Hz, 2 H, CH$_2$C*H*$_2$); $^{13}$C{$^1$H} NMR (101 MHz, $CDCl_3$) δ 178.7 ($C_q$=O), 158.2 (Ar-$C_q$OMe), 135.5 (Ar-$C_qC_q$), 126.8 (2 × Ar-CH), 114.0 (2 × Ar-CH), 81.5 ($CH_2OCH_2$), 55.3 ($OCH_3$), 46.1 ($C_q$), 35.6 (*C*H$_2$CH$_2$), 29.5 (CH$_2$CH$_2$); HRMS (ESI) *m/z*: $[M+MeCN+H]^+$ Calcd for $C_{15}H_{20}NO_4$ 278.1392; Found 278.1400.

Notes:

***15** was further characterized by X-ray crystallography (see Figures S33–S34).*

**5-([1,1'-Biphenyl]-4-yl)-5-(hydroxymethyl)tetrahydro-2H-pyran-2-one (16)**

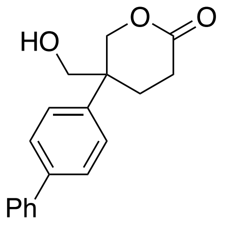

Trifluoroacetic acid (36 µL, 0.47 mmol, 10 equiv) was added dropwise to a solution of bi-phenyl oxetane ethyl ester **11b** (16.0 mg, 0.047 mmol, 1.0 equiv) in anhydrous $CH_2Cl_2$ (1.0 mL, 0.05 M) at 0 °C. After stirring for 5 min at 0 °C, the reaction mixture was warmed to 25 °C and stirred for further 17 h. The reaction mixture was concentrated *in vacuo* using a rotatory evaporator and purification by silica flash column chromatography (50% $Et_2O$/pentane, then 70% $Et_2O$/pentane + 0.5% AcOH to 100% $Et_2O$ + 0.5% AcOH) afforded impure **16** (12.9 mg). Repurification by silica flash column chromatography (50–60% EtOAc/pentane) afforded tetrahydropyranone **16** as a white solid (10.0 mg, 75%). $R_f$ = 0.15 (60% EtOAc/pentane); mp = 153–155 °C; IR (film)/cm$^{-1}$ 3427 (OH st, br), 2924, 1728 (C=O st), 1484, 1187, 1104, 1081, 1056, 1034, 766, 698; $^1$H NMR (400 MHz, $CDCl_3$) δ 7.68–7.61 (m, 2 H, 2 × Ar-CH), 7.63–7.56 (m, 2 H, 2 × Ar-CH), 7.52–7.42 (4 × Ar-CH), 7.42–7.34 (m, 1 H, Ph-CH), 4.88 (dd, $^2J$ = 11.9 Hz, $^4J_{eq,eq}$ = 2.1 Hz, 1H, (C=O)OC*H*H), 4.60 (d, *J* = 11.9 Hz, 1H, (C=O)OCH*H*)), 3.86 (dd, $^2J$ = 11.3 Hz, $^3J$ = 4.5 Hz, 1H, HOC*H*H), 3.78 (dd, $^2J$ = 11.3 Hz, $^3J$ = 7.0 Hz, 1H, HOCH*H*), 2.65 (ddd, $^2J$ = 17.8 Hz, $^3J_{eq,ax}$ = 6.6 Hz, $^3J_{eq,eq}$ = 4.2 Hz, 1H, C*H*HCH$_2$), 2.44 (ddd, $^2J$ = 17.8 Hz, $^3J_{ax,ax}$ = 10.4 Hz, $^3J_{ax,eq}$ = 7.2 Hz, 1H, CH*H*CH$_2$), 2.34–2.25 (m, 1H, CH$_2$C*H*H), 2.20 (ddd, $^2J$ = 13.7 Hz, $^3J_{ax,ax}$ = 10.4 Hz, $^3J_{ax,eq}$ = 6.6 Hz, 1 H, CH$_2$CH*H*), 1.53–1.44 (m, 1H, OH); $^{13}$C{$^1$H} NMR (101 MHz, $CDCl_3$) δ 170.8 ($C_q$=O), 140.5 (Ar-$C_q$), 140.2 (Ar-$C_q$), 138.3 (Ar-$C_qC_q$), 128.9 (2 × Ar-CH), 127.9 (2 × Ar-CH), 127.6 (Ph-CH), 127.1 (2 × Ar-CH), 127.0 (2 × Ar-CH), 73.0 (*C*H$_2$O(C=O)), 68.7 ($CH_2OH$), 42.6 ($C_q$), 28.0 (*C*H$_2$CH$_2$), 27.2 (CH$_2$*C*H$_2$); HRMS (ESI) *m/z*: [M+MeCN+H]$^+$ Calcd for $C_{20}H_{22}NO_3$ 324.1600; Found 324.1606.

Oxetane–TEMPO Adduct **17**

**1-((3-(4-Methoxyphenyl)oxetan-3-yl)oxy)-2,2,6,6-tetramethylpiperidine (17)**

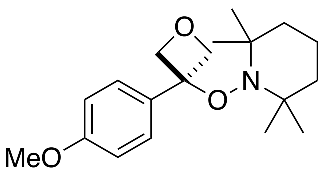


An oven-dried 4 mL vial was charged with oxetane carboxylic acid **1** (41.6 mg, 0.20 mmol, 1.0 equiv), oven-dried $Cs_2CO_3$ (78.2 mg, 0.24 mmol, 1.2 equiv), (2,2,6,6-tetramethylpiperidin-1-yl)oxyl (TEMPO, 62.5 mg, 0.40 mmol, 2.0 equiv) and [Ir{dF($CF_3$)ppy}$_2$(dtbbpy)]$PF_6$ (2.2 mg, 0.002 mmol, 1.0 mol%). The vial was sealed with a screwcap equipped with a PTFE/silicon septum and anhydrous DMF (1.0 mL, 0.2 M) was added by syringe. Argon was bubbled through the mixture for 5 min and ethyl acrylate (33 μL, 0.30 mmol, 1.5 equiv) was added by syringe under argon. After sealing the cap with parafilm on top, the reaction mixture was stirred at 1000 rpm using the set-up shown in Figure S2 and irradiated with two 467 nm Kessel lamps at 36 °C (heat generated by the lamps). After 18 h the lights were switched off and the reaction mixture was transferred into a separating funnel. Distilled water (10 mL) and $Et_2O$ (10 mL) were added, the layers were separated and the aqueous portion was extracted with $Et_2O$ (2 × 10 mL). The organic extracts were combined, dried over $Na_2SO_4$, filtered and concentrated *in vacuo* using a rotatory evaporator. Purification by basic alumina (IV) column chromatography (10–100% $Et_2O$/pentane) afforded impure oxetane **17** as a colorless gum (27.5 mg). Repurification by basic alumina (IV) column chromatography (80% $CH_2Cl_2$/pentane) afforded oxetane **17** as colorless crystals (13.6 mg, 21%). $R_f$ = 0.22 (80% $CH_2Cl_2$/pentane); mp = 207 °C; IR (film)/cm$^{-1}$ 2927, 1511, 1245, 1178; $^1$H NMR (400 MHz, $CDCl_3$) δ 7.66–7.63 (m, 2 H, 2 × Ar-CH), 6.96–6.92 (m, 2 H, 2 × Ar-CH), 5.57 (d, *J* = 7.0 Hz, 2 H, C*H*HOC*H*H), 4.52 (d, *J* = 7.0 Hz, 2 H, CH*H*OCH*H*), 3.84 (s, 3 H, $OCH_3$), 1.66–1.49 (m, 5 H, C*H*$_2$C*H*HC*H*$_2$), 1.38–1.33 (m, 1 H, $CH_2$CH*H*$CH_2$), 1.18 (s, 6 H, 2 × $CH_3$), 0.99 (s, 6 H, 2 × $CH_3$); $^{13}$C{$^1$H} NMR (101 MHz, $CDCl_3$) δ 158.5 (Ar-$C_q$OMe), 136.9 (Ar-*C*$_q$C$_q$), 125.8 (2 × Ar-CH), 113.6 (2 × Ar-CH), 82.1 ($C_q$), 81.3 ($CH_2OCH_2$), 59.5 ($C_qNC_q$), 55.2 ($OCH_3$), 40.4 (*C*$H_2CH_2$*C*$H_2$), 33.3 (2 × $CH_3$), 21.0 (2 × $CH_3$), 17.1 ($CH_2$*C*$H_2CH_2$); HRMS (ESI) *m/z*: $[M+H]^+$ Calcd for $C_{19}H_{30}NO_3$ 320.2226; Found 320.2223.

Notes:

***17** was further characterized by X-ray crystallography (see Figures S35–S36).*

PMP Acetic Acids with different benzylic substituents (**I**–**VII**)

**Methylene Products (I-A**, **I-A'**, **I-B)**

**Ethyl 4-(4-methoxyphenyl)butanoate (I-A)**, **diethyl 2-(4-methoxyphenethyl)pentanedioate (I-A')** and **1,2-bis(4-methoxyphenyl)ethane (I-B)**

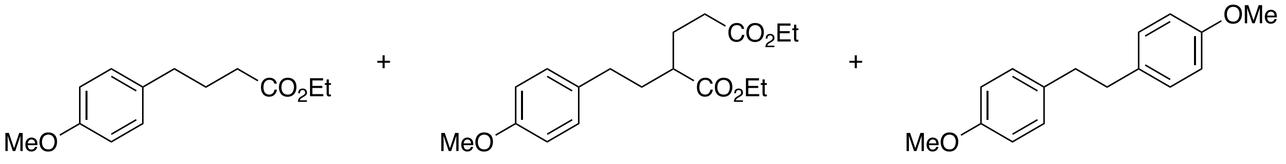


**I-A** **I-A'** **I-B**

Following **General Procedure A**, 2-(4-methoxyphenyl)acetic acid (33.2 mg) and ethyl acrylate (33 μL) were used. Purification by silica flash column chromatography (4–20% $Et_2O$/pentane) afforded dimer **I-B** as a white film (2.6 mg, 11%), followed by alkane **I-A** as a colorless oil (16.7 mg, 38%) and alkane **I-A'** as a colorless oil (5.9 mg, 9%).

Dimer **I-B**: $R_f$ = 0.79 (20% $Et_2O$/pentane); IR (film)/cm$^{-1}$ 2915, 2363, 1510, 1245, 1029; $^1$H NMR (400 MHz, $CDCl_3$) δ 7.11–7.07 (m, 4 H, 4 × Ar-CH), 6.85–6.81 (m, 4 H, 4 × Ar-CH), 3.80 (s, 6 H, 2 × $OCH_3$), 2.83 (s, 4 H, 2 × $CH_2$); $^{13}$C{$^1$H} NMR (101 MHz, $CDCl_3$) δ 158.0 (2 × Ar-$C_q$OMe), 134.0 (2 × Ar-$C_q$$CH_2$), 129.4 (4 × Ar-CH), 113.7 (4 × Ar-CH), 55.2 (2 × $OCH_3$), 37.3 (2 × $CH_2$). The observed characterization data ($^1$H and $^{13}$C NMR) were consistent with that previously reported.[35]

Alkane **I-A**: $R_f$ = 0.61 (20% $Et_2O$/pentane); IR (film)/cm$^{-1}$ 2937, 1730 (C=O st), 1513, 1245, 1178, 1036; $^1$H NMR (400 MHz, $CDCl_3$) δ 7.12–7.09 (m, 2 H, 2 × Ar-CH), 6.86–6.82 (m, 2 H, 2 × Ar-CH), 4.13 (q, *J* = 7.1 Hz, 2 H, $CH_2CH_3$), 3.80 (s, 3 H, $OCH_3$), 2.60 (t, *J* = 7.6 Hz, 2 H, $CH_2CH_2CH_2$), 2.31 (t, *J* = 7.5 Hz, 2 H, $CH_2CH_2CH_2$), 1.97–1.89 (m, 2 H, $CH_2CH_2CH_2$), 1.21 (t, *J* = 7.1 Hz, 3 H, $CH_2CH_3$); $^{13}$C{$^1$H} NMR (101 MHz, $CDCl_3$) δ 173.6 ($C_q$=O), 157.8 (Ar-$C_q$OMe), 133.5 (Ar-$C_q$$CH_2$), 129.4 (2 × Ar-CH), 113.7 (2 × Ar-CH), 60.2 ($CH_2CH_3$), 55.2 ($OCH_3$), 34.2 ($CH_2$), 33.6 ($CH_2$), 26.8 ($CH_2$), 14.2 ($CH_2CH_3$). The observed characterization data ($^1$H NMR) were consistent with that previously reported.[36]

Alkane **I-A'**: $R_f$ = 0.30 (20% $Et_2O$/pentane); IR (film)/cm$^{-1}$ 2937, 1730 (C=O st), 1513, 1245, 1178, 1036; $^1$H NMR (400 MHz, $CDCl_3$) δ 7.10–7.08 (m, 2 H, 2 × Ar-CH), 6.84–6.82 (m, 2 H, 2 × Ar-CH), 4.19–4.10 (m, 4 H, 2 × $CH_2CH_3$), 3.79 (s, 3 H, $OCH_3$), 2.62–2.49 (m, 2 H, 2 × CH), 2.45–2.38 (m, 1 H, CH), 2.34–2.28 (m, 2 H, 2 × CH), 2.00–1.82 (m, 3 H, 3 × CH), 1.78–1.69 (m, 1 H, CH), 1.29 (t, *J* = 7.1 Hz, 3 H, $CH_2CH_3$), 1.25 (t, *J* = 7.2 Hz, 3 H, $CH_2CH_3$); $^{13}$C{$^1$H} NMR (101 MHz, $CDCl_3$) δ 175.4 ($C_q$=O), 173.0 ($C_q$=O), 157.8 (Ar-$C_q$OMe), 133.5 (Ar-$C_q$$CH_2$), 129.3 (2 × Ar-CH), 113.8 (2 × Ar-CH), 60.41 ($CH_2CH_3$), 60.36 ($CH_2CH_3$), 55.2 ($OCH_3$), 44.3 (CH), 34.2 ($CH_2$), 32.6 ($CH_2$), 32.0 ($CH_2$), 27.2 ($CH_2$), 14.3 ($CH_2CH_3$), 14.2 ($CH_2CH_3$); HRMS (APCI) *m/z*: $[M+H]^+$ Calcd for $C_{18}H_{27}O_5^+$ 323.1853; Found 323.1851.

**Cyclopropane Products (II-A)**

**Ethyl 3-(1-(4-methoxyphenyl)cyclopropyl)propanoate (II-A)**

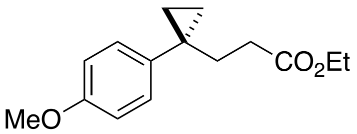

Following **General Procedure A**, 1-(4-methoxyphenyl)cyclopropane-1-carboxylic acid (38.4 mg) and ethyl acrylate (33 μL) were used. Purification by basic alumina (IV) column chromatography (0–5% $Et_2O$/pentane) afforded cyclopropane **II-A** as a colorless gum (9.2 mg, 19%). $R_f$ = 0.31 (10% $Et_2O$/pentane); IR (film)/cm$^{-1}$ 2982, 1730 (C=O st), 1513, 1245, 1178, 1036; $^1$H NMR (400 MHz, $CDCl_3$) δ 7.24–7.20 (m, 2 H, 2 × Ar-CH), 6.84–6.81 (m, 2 H, 2 × Ar-CH), 4.06 (q, *J* = 7.1 Hz, 2 H, $CH_2CH_3$), 3.79 (s, 3 H, $OCH_3$), 2.29–2.25 (m, 2 H, $CH_2CH_2$), 1.87–1.83 (m, 2 H, $CH_2CH_2$), 1.22 (t, *J* = 7.1 Hz, 3 H, $CH_2CH_3$), 0.78–0.75 (m, 2 H, $CH_{2(cyclopropane)}$), 0.69–0.66 (m, 2 H, $CH_{2(cyclopropane)}$); $^{13}C\{^1H\}$ NMR (101 MHz, $CDCl_3$) δ 173.8 ($C_q$=O), 158.0 (Ar-$C_q$OMe), 136.2 (Ar-$C_qC_q$), 130.3 (2 × Ar-CH), 113.5 (2 × Ar-CH), 60.2 ($CH_2CH_3$), 55.3 and 55.2 ($OCH_3$), 35.9 ($CH_2CH_2$), 32.4 ($CH_2CH_2$), 24.6 ($C_q$), 14.2 ($CH_2CH_3$), 12.8 (2 × $CH_{2(cyclopropane)}$); HRMS (ESI) *m/z*: $[M+H]^+$ Calcd for $C_{15}H_{21}O_3$ 249.1491; Found 249.1500.

**Oxetane Products (III-A, III-A')**

**Ethyl 3-(3-(4-methoxyphenyl)oxetan-3-yl)propanoate (III-A, 2a) and diethyl 2-((3-(4-methoxyphenyl)oxetan-3-yl)methyl)pentanedioate (III-A', 2a')**

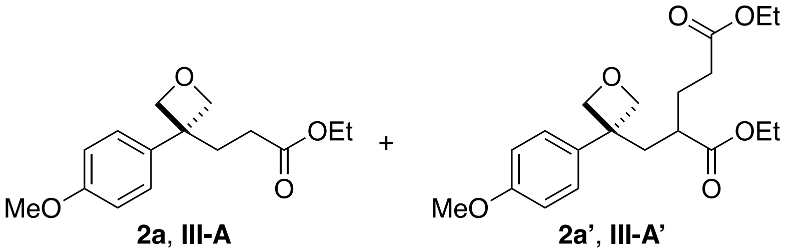


**2a**, **III-A** + **2a'**, **III-A'**

For procedure and characterization data, see **2a** and **2a'**, page S46.

**Azetidine Products (IV-A, IV-A')**

**Benzyl 3-(3-ethoxy-3-oxopropyl)-3-(4-methoxyphenyl)azetidine-1-carboxylate (IV-A, 13a)** and **diethyl 2-((1-((benzyloxy)carbonyl)-3-(4-methoxyphenyl)azetidin-3-yl)methyl)pentanedioate (IV-A', 13a')**

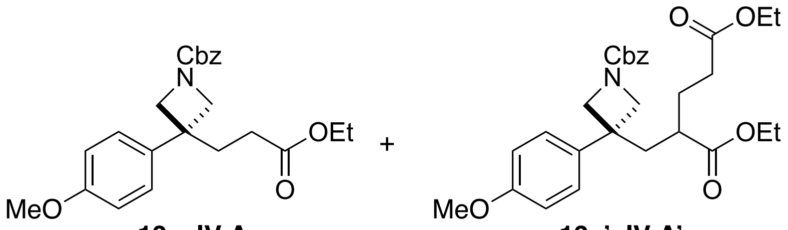


**13a**, **IV-A** **13a'**, **IV-A'**

For procedure and characterization data, see **13a** and **13a'**, page S59–S60.

**Cyclobutane Products (V-A, V-A’, V-B)**

**Ethyl 3-(1-(4-methoxyphenyl)cyclobutyl)propanoate (V-A), diethyl 2-((1-(4-methoxyphenyl)cyclobutyl)methyl)pentanedioate (V-A’)** and **1,1'-bis(4-methoxyphenyl)-1,1'-bi(cyclobutane) (V-B)**

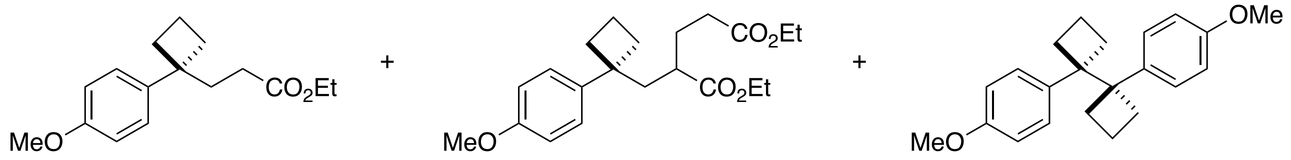


**V-A** **V-A’** **V-B**

Following **General Procedure A**, 1-(4-methoxyphenyl)cyclobutane-1-carboxylic acid (41.3 mg) and ethyl acrylate (33 μL) were used. Purification by silica flash column chromatography (4–20% $Et_2O$/pentane) afforded dimer **V-B** as a colorless oil (1.9 mg, 6%), followed by cyclobutane **V-A** as a colorless oil (19.0 mg, 36%) and cyclobutane **V-A’** as a colorless oil (5.3 mg, 7%).

Dimer **V-B:** $R_f$ = 0.32 (4% $Et_2O$/pentane); IR (film)/$cm^{-1}$ 2944, 1513, 1245, 1178, 828; $^1H$ NMR (400 MHz, $CDCl_3$) δ 6.64 (s, 8 H, 8 × Ar-CH), 3.77 (s, 6 H, 2 × $OCH_3$), 2.66–2.59 (m, 4 H, 2 × $CH_2$), 2.23–2.16 (m, 4 H, 2 × $CH_2$), 1.79–1.67 (m, 4 H, 2 × $CH_2$); $^{13}C\{^1H\}$ NMR (101 MHz, $CDCl_3$) δ 157.1 (2 × Ar-$C_q$OMe), 138.5 (2 × Ar-$\mathit{C_q}C_q$), 128.9 (4 × Ar-CH), 112.0 (4 × Ar-CH), 55.2 (2 × $OCH_3$), 52.6 (2 × $C_q$), 29.9 (2 × $\mathit{C}H_2CH_2\mathit{C}H_2$), 15.1 (2 × $CH_2\mathit{C}H_2CH_2$); HRMS (APCI) *m/z*: $[M+NH_4]^+$ Calcd for $C_{22}H_{30}NO_2^+$ 340.2271; Found 340.2275.

Cyclobutane **V-A**: $R_f$ = 0.14 (4% $Et_2O$/pentane); IR (film)/$cm^{-1}$ 2933, 1733 (C=O st), 1513, 1248, 1178, 1036; $^1H$ NMR (400 MHz, $CDCl_3$) δ 7.04–7.01 (m, 2 H, 2 × Ar-CH), 6.86–6.83 (m, 2 H, 2 × Ar-CH), 4.04 (q, *J* = 7.2 Hz, 2 H, $C\mathit{H_2}CH_3$), 3.80 (s, 3 H, $OCH_3$), 2.37–2.27 (m, 2 H, $C\mathit{H_2}CH_2$), 2.14–2.05 (m, 5 H, $CH_2C\mathit{H_2}$ + $CH_{2(cyclobutane)}$ + $C\mathit{H}H_{(cyclobutane)}$), 2.04–1.99 (m, 2 H, $CH_{2(cyclobutane)}$), 1.87–1.79 (m, 1 H, $CH\mathit{H}_{(cyclobutane)}$), 1.21 (t, *J* = 7.2 Hz, 3 H, $CH_2C\mathit{H_3}$); $^{13}C\{^1H\}$ NMR (101 MHz, $CDCl_3$) δ 174.0 ($C_q$=O), 157.4 (Ar-$C_q$OMe), 141.1 (Ar-$\mathit{C_q}C_q$), 126.7 (2 × Ar-CH), 113.4 (2 × Ar-CH), 60.2 ($\mathit{C}H_2CH_3$), 55.2 ($OCH_3$), 45.3 ($C_q$), 37.3 ($\mathit{C}H_2CH_2$), 32.5 ($\mathit{C}H_2CH_2\mathit{C}H_2$), 30.1 ($CH_2\mathit{C}H_2$), 15.8 ($CH_2\mathit{C}H_2CH_2$), 14.2 ($CH_2\mathit{C}H_3$); HRMS (APCI) *m/z*: $[M+Na]^+$ Calcd for $C_{16}H_{22}O_3Na^+$ 285.1461; Found 285.1465.

Cyclobutane **V-A’**: $R_f$ = 0.29 (20% $Et_2O$/pentane); IR (film)/$cm^{-1}$ 2978, 1733 (C=O st), 1513, 1248, 1178, 1036; $^1H$ NMR (400 MHz, $CDCl_3$) δ 7.09–7.05 (m, 2 H, 2 × Ar-CH), 6.85–6.82 (m, 2 H, 2 × Ar-CH), 4.07 (q, *J* = 7.1 Hz, 2 H, $C\mathit{H_2}CH_3$), 3.92–3.82 (m, 2 H, $C\mathit{H_2}CH_3$), 3.80 (s, 3 H, $OCH_3$), 2.39–2.23 (m, 3 H, $C_qC\mathit{H}HCH$ + 2 × $CH_{(cyclobutane)}$), 2.16–1.97 (m, 6 H, 3 × $CH_{(cyclobutane)}$ + CH + $CHC\mathit{H_2}CH_2$), 1.83–1.72 (m, 3 H, $CH_2C\mathit{H}HCO_2$ + $C_qCH\mathit{H}CH$ + $CH_{(cyclobutane)}$), 1.68–1.62 (m, 1 H, $CH_2CH\mathit{H}CO_2$), 1.21 (t, *J* = 7.2 Hz, 3 H, $CH_2C\mathit{H_3}$), 1.17 (t, *J* = 7.2 Hz, 3 H, $CH_2C\mathit{H_3}$); $^{13}C\{^1H\}$ NMR (101 MHz, $CDCl_3$) δ 175.8 ($C_q$=O), 172.8 ($C_q$=O), 157.4 (Ar-$C_q$OMe), 140.6 (Ar-$\mathit{C_q}C_q$), 127.1 (2 × Ar-CH), 113.3 (2 × Ar-CH), 60.3 ($\mathit{C}H_2CH_3$), 60.2 ($\mathit{C}H_2CH_3$), 55.2 ($OCH_3$), 45.7 ($C_q$), 44.9 ($C_q\mathit{C}H_2CH$), 41.5 (CH), 33.6 ($CH_{2(cyclobutane)}$), 33.0 ($CH_{2(cyclobutane)}$), 31.8 ($CH\mathit{C}H_2CH_2$), 29.9 ($CH_2\mathit{C}H_2CO_2$), 15.9 ($CH_{2(cyclobutane)}$), 14.1 ($CH_2\mathit{C}H_3$), 14.0 ($CH_2\mathit{C}H_3$); HRMS (ESI) *m/z*: $[M+H]^+$ Calcd for $C_{21}H_{31}O_5$ 363.2171; Found 363.2173.

Tetrahydropyran Products (**VI-A**, **VI-A’**, **VI-B**)

**Ethyl 3-(4-(4-methoxyphenyl)tetrahydro-2H-pyran-4-yl)propanoate (VI-A)**, **diethyl 2-((4-(4-methoxyphenyl)tetrahydro-2H-pyran-4-yl)methyl)pentanedioate (VI-A’)**, and **4,4'-bis(4-methoxyphenyl)octahydro-2H,2'H-4,4'-bipyran (VI-B)**

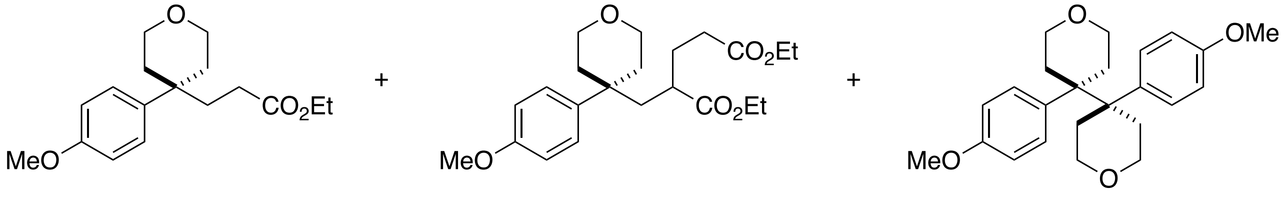


**VI-A** **VI-A’** **VI-B**

Following **General Procedure A**, 4-(4-methoxyphenyl)tetrahydro-2H-pyran-4-carboxylic acid (47.3 mg) and ethyl acrylate (33 μL) were used. Purification by silica flash column chromatography (8–60% $Et_2O$/pentane) afforded THP **VI-A** as a colorless oil (19.3 mg, 33%), followed by THP **VI-A’** as a colorless oil (9.5 mg, 12%) and dimer **VI-B** as a white solid (8.7 mg, 23%).

THP **VI-A**: $R_f$ = 0.31 (40% $Et_2O$/pentane); IR (film)/$cm^{-1}$ 2937, 2855, 1730 (C=O st), 1513, 1249, 1185, 1115, 1033, 828; $^1$H NMR (400 MHz, $CDCl_3$) δ 7.19–7.15 (m, 2 H, 2 × Ar-CH), 6.91–6.87 (m, 2 H, 2 × Ar-CH), 4.02 (q, *J* = 7.1 Hz, 2 H, $CH_2CH_3$), 3.81 (s, 3 H, $OCH_3$), 3.80–3.76 (m, 2 H, $CH_{eq}HOCH_{eq}H$), 3.55 (ddd, $^2J$ = 11.8 Hz, $^3J_{ax,ax}$ = 9.3 Hz, $^3J_{ax,eq}$ = 2.7 Hz, 2 H, $CHH_{ax}OCHH_{ax}$), 2.14–2.09 (m, 2 H, $CH_{eq}HC_qCH_{eq}H$), 1.98–1.89 (m, 4 H, $CH_2CH_2CO_2$), 1.81 (ddd, $^2J$ = 13.6 Hz, $^3J_{ax,ax}$ = 9.3 Hz, $^3J_{ax,eq}$ = 3.7 Hz, 2 H, $CHH_{ax}C_qCHH_{ax}$), 1.19 (t, *J* = 7.1 Hz, 3 H, $CH_2CH_3$); $^{13}C\{^1H\}$ NMR (101 MHz, $CDCl_3$) δ 173.7 ($C_q$=O), 157.7 (Ar-$C_q$OMe), 135.9 (Ar-$C_qC_q$), 127.8 (2 × Ar-CH), 113.9 (2 × Ar-CH), 64.2 ($CH_2OCH_2$), 60.3 ($CH_2CH_3$), 55.2 ($OCH_3$), 38.22 ($C_q$), 38.16 ($CH_2$), 36.2 ($CH_2C_qCH_2$), 28.8 ($CH_2$), 14.1 ($CH_2CH_3$); HRMS (ESI) *m/z*: $[M+H]^+$ Calcd for $C_{17}H_{25}O_4$ 293.1753; Found 293.1748.

THP **VI-A’**: $R_f$ = 0.24 (40% $Et_2O$/pentane); IR (film)/$cm^{-1}$ 2937, 2855, 1730 (C=O st), 1513, 1252, 1185, 1032; $^1$H NMR (400 MHz, $CDCl_3$) δ 7.18–7.14 (m, 2 H, 2 × Ar-CH), 6.89–6.85 (m, 2 H, 2 × Ar-CH), 4.07 (q, *J* = 7.1 Hz, 2 H, $CH_2CH_3$), 3.91–3.81 (m, 2 H, $CH_2CH_3$), 3.81 (s, 3 H, $OCH_3$), 3.79–3.71 (m, 2 H, CHHOCHH), 3.57–3.46 (m, 2 H, CHHOCHH), 2.27 (dd, *J* = 14.0, 9.5 Hz, 1 H, $C_qCHHCH$), 2.15–2.03 (m, 4 H, CH + $CHHCH_2CO_2$ + $CHHC_qCHH$), 2.04–1.93 (m, 1 H, $CHHCH_2CO_2$), 1.84–1.67 (m, 3 H, $CHHC_qCHH$ + $CH_2CHHCO_2$), 1.61–1.53 (m, 1 H, $CH_2CHHCO_2$), 1.55 (dd, *J* = 14.0, 2.0 Hz, 1 H, $C_qCHHCH$), 1.21 (t, *J* = 7.1 Hz, 3 H, $CH_2CH_3$), 1.16 (t, *J* = 7.2 Hz, 3 H, $CH_2CH_3$); $^{13}C\{^1H\}$ NMR (101 MHz, $CDCl_3$) δ 175.8 ($C_q$=O), 172.7 ($C_q$=O), 157.7 (Ar-$C_q$OMe), 135.6 (Ar-$C_qC_q$), 128.2 (2 × Ar-CH), 113.7 (2 × Ar-CH), 64.2 ($CH_2OCH_2$), 64.1 ($CH_2OCH_2$), 60.4 ($CH_2CH_3$), 60.3 ($CH_2CH_3$), 55.2 ($OCH_3$), 45.9 ($C_qCH_2CH$), 40.1 (CH), 38.7 ($C_q$), 36.8 ($CH_2C_qCH_2$), 36.0 ($CH_2C_qCH_2$), 31.6 ($CHCH_2CH_2$), 29.5 ($CH_2CH_2CO_2$), 14.1 ($CH_2CH_3$), 14.0 ($CH_2CH_3$); HRMS (ESI) *m/z*: $[M+H]^+$ Calcd for $C_{22}H_{33}O_6$ 393.2277; Found 393.2273.

Notes:

*The multiplet at 3.91–3.81 ppm and the singlet at 3.81 ppm were integrated together to 5 H.*

*The multiplet at 1.61–1.53 ppm and the double doublet at 1.55 ppm were integrated together to 2 H.*

Dimer **VI-B:** $R_f$ = 0.06 (40% $Et_2O$/pentane); mp = 180–185 °C; IR (film)/$cm^{-1}$ 2956, 2863, 1733, 1513, 1252, 1185, 1107, 1033; $^1$H NMR (400 MHz, $CDCl_3$) δ 6.78–6.72 (m, 8 H, 8 × Ar-CH), 3.82 (s, 6 H, 2 × $OCH_3$), 3.72 (ddd, $^2J$ = 11.7 Hz, $^3J_{eq,ax}$ = 4.1 Hz, $^3J_{eq,eq}$ = 2.1 Hz, 4 H, 2 × $CH_{eq}HOCH_{eq}H$), 3.26 (ddd, $^2J$ = 11.7 Hz, $^3J_{ax,ax}$ = 11.7 Hz, $^3J_{ax,eq}$ = 1.9 Hz, 4 H, 2 × $CHH_{ax}OCHH_{ax}$), 2.08–2.05 (m, 4 H, 2 × $CH_{eq}HC_qCH_{eq}H$), 1.99 (ddd, $^2J$ = 13.6 Hz, $^3J_{ax,ax}$ = 11.7 Hz, $^3J_{ax,eq}$ = 4.1 Hz, 4 H, 2 × $CHH_{ax}C_qCHH_{ax}$); $^{13}C\{^1H\}$ NMR (101 MHz, $CDCl_3$) δ 157.7 (2 × Ar-$C_q$OMe), 131.1 (4 × Ar-CH), 130.7 (2 × Ar-$C_qC_q$), 112.6 (4 × Ar-CH), 64.5 (2 × $CH_2OCH_2$), 55.1 (2 × $OCH_3$), 46.9 (2 × $C_q$), 30.5 (2 × $CH_2C_qCH_2$); HRMS (ESI) *m/z*: $[M+MeCN+H]^+$ Calcd for $C_{26}H_{34}NO_4$ 424.2488; Found 424.2500.

*gem*-Dimethyl Products (**VII-A**, **VII-A’**, **VII-B**)

**Ethyl 4-(4-methoxyphenyl)-4-methylpentanoate (VII-A), diethyl 2-(2-(4-methoxyphenyl)-2-methylpropyl)pentanedioate (VII-A’) and 4,4'-(2,3-dimethylbutane-2,3-diyl)bis(methoxybenzene) (VII-B)**

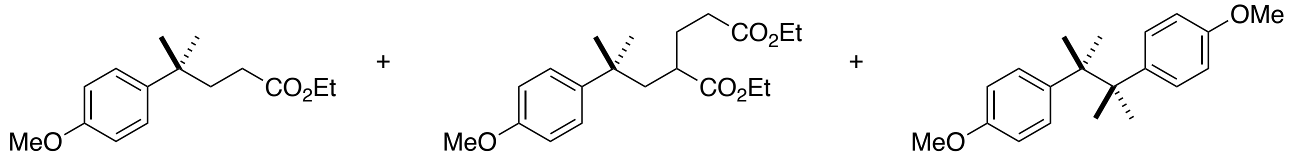


**VII-A** **VII-A’** **VII-B**

Following **General Procedure A**, 2-(4-methoxyphenyl)-2-methylpropanoic acid (38.8 mg) and ethyl acrylate (33 μL) were used. Purification by silica flash column chromatography (2–15% $Et_2O$/pentane) afforded dimer **VII-B** (11.8 mg, 40%) as a white solid, followed by alkane **VII-A** (22.5 mg, 45%) as a colorless oil and alkane **VII-A’** (8.1 mg, 12%) as a colorless oil.

Dimer **VII-B:** $R_f$ = 0.54 (4% $Et_2O$/pentane); mp = 185–188 °C [lit. 184 °C][37]; IR (film)/$cm^{-1}$ 2985, 1513, 1244, 1185, 1036; $^1H$ NMR (400 MHz, $CDCl_3$) δ 6.98–6.95 (m, 4 H, 4 × Ar-CH), 6.75–6.71 (m, 4 H, 4 × Ar-CH), 3.80 (s, 6 H, 2 × $OCH_3$), 1.28 (s, 12 H, 4 × $CH_3$); $^{13}C\{^1H\}$ NMR (101 MHz, $CDCl_3$) δ 157.3 (2 × Ar-$C_q$OMe), 139.1 (2 × Ar-$C_qC_q$), 129.6 (4 × Ar-CH), 111.8 (4 × Ar-CH), 55.1 (2 × $OCH_3$), 43.1 (2 × $C_q$), 25.3 (4 × $CH_3$). The observed characterization data ($^1H$ and $^{13}C$ NMR) were consistent with that previously reported.[38]

Notes:

**VII-B** *was further characterized by X-ray crystallography (see Figure S37).*

*gem*-Dimethyl **VII-A**: $R_f$ = 0.23 (4% $Et_2O$/pentane); IR (film)/$cm^{-1}$ 2960, 1730 (C=O st), 1513, 1249, 1182, 1036, 831; $^1H$ NMR (400 MHz, $CDCl_3$) δ 7.27–7.23 (m, 2 H, 2 × Ar-CH), 6.87–6.84 (m, 2 H, 2 × Ar-CH), 4.06 (q, *J* = 7.1 Hz, 2 H, $CH_2CH_3$), 3.80 (s, 3 H, $OCH_3$), 2.08–2.04 (m, 2 H, $CH_2CH_2$), 1.96–1.92 (m, 2 H, $CH_2CH_2$), 1.31 (s, 6 H, 2 × $CH_3$), 1.21 (t, *J* = 7.1 Hz, 3 H, $CH_2CH_3$); $^{13}C\{^1H\}$ NMR (101 MHz, $CDCl_3$) δ 174.1 ($C_q$=O), 157.5 (Ar-$C_q$OMe), 140.1 (Ar-$C_qC_q$), 126.8 (2 × Ar-CH), 113.5 (2 × Ar-CH), 60.2 ($CH_2CH_3$), 55.2 ($OCH_3$), 39.0 ($CH_2CH_2$), 36.7 ($C_q$), 30.3 ($CH_2CH_2$), 28.9 (2 × $CH_3$), 14.2 ($CH_2CH_3$); HRMS (ESI) *m/z*: $[M+MeCN+H]^+$ Calcd for $C_{17}H_{26}NO_3$ 292.1913; Found 292.1919.

*gem*-Dimethyl **VII-A’**: $R_f$ = 0.34 (20% $Et_2O$/pentane); IR (film)/$cm^{-1}$ 2960, 1730 (C=O st), 1513, 1252, 1185, 1036; $^1H$ NMR (400 MHz, $CDCl_3$) δ 7.25–7.21 (m, 2 H, 2 × Ar-CH), 6.85–6.81 (m, 2 H, 2 × Ar-CH), 4.09 (q, *J* = 7.1 Hz, 2 H, $CH_2CH_3$), 3.93–3.82 (m, 2 H, $CH_2CH_3$), 3.79 (s, 3 H, $OCH_3$), 2.28–2.15 (m, 4 H, $C_qCH$HCH + CH + $CHCH_2CH_2$), 1.82–1.62 (m, 3 H, $C_qCHH$CH + $CH_2CH_2CO_2$), 1.29 (s, 3 H, $CH_3$), 1.26 (s, 3 H, $CH_3$), 1.22 (t, *J* = 7.1 Hz, 3 H, $CH_2CH_3$), 1.16 (t, *J* = 7.2 Hz, 3 H, $CH_2CH_3$); $^{13}C\{^1H\}$ NMR (101 MHz, $CDCl_3$) δ 176.1 ($C_q$=O), 172.8 ($C_q$=O), 157.5 (Ar-$C_q$OMe), 140.1 (Ar-$C_qC_q$), 127.0 (2 × Ar-CH), 113.3 (2 × Ar-CH), 60.4 ($CH_2CH_3$), 60.2 ($CH_2CH_3$), 55.2 ($OCH_3$), 46.6 ($C_qCH_2CH$), 41.5 (CH), 37.2 ($C_q$), 31.8 ($CHCH_2CH_2$), 29.6 ($CH_2CH_2CO_2$), 29.2 ($CH_3$), 28.7 ($CH_3$), 14.2 ($CH_2CH_3$), 14.0 ($CH_2CH_3$); HRMS (ESI) *m/z*: $[M+H]^+$ Calcd for $C_{20}H_{31}O_5$ 351.2171; Found 351.2167.

4CzIPN

**2,4,5,6-Tetra(9*H*-carbazol-9-yl)isophthalonitrile (4CzIPN)**[39]

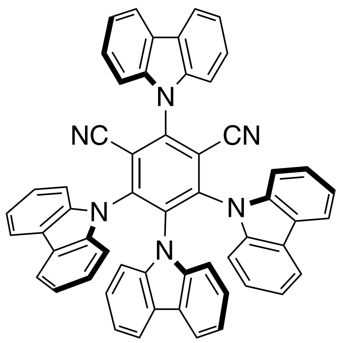

NaH (60% in mineral oil, 600 mg, 15 mmol, 7.5 equiv) was added portionwise to a solution of 9*H*-carbazole (1.67 g, 10 mmol, 5.0 equiv) in anhydrous THF (40 mL, 0.05 M) at 25 °C. After stirring for 30 min at 25 °C, 2,4,5,6-tetrafluoroisophthalonitrile (400 mg, 2.0 mmol, 1.0 equiv) was added and the reaction mixture was stirred at 25 °C for 15 h. The reaction was quenched with distilled water (2 mL) and the mixture was then concentrated *in vacuo* using a rotatory evaporator to yield the crude mixture as a yellow solid. The solid was washed sequentially with distilled water (50 mL), EtOH (50 mL) and $Et_2O$ (50 mL). Purification by recrystallization from hot $CHCl_3$/hexane (5:1; 24 mL) afforded 4CzIPN as a bright yellow solid (1.28 g, 81%). $R_f$ = 0.32 (50% $CH_2Cl_2$/hexane); mp = no melting above 270 °C; IR (film)/cm$^{-1}$ 3052, 1449, 1310, 1221, 907, 742, 722; $^1$H NMR (400 MHz, $CDCl_3$) $\delta$ 8.24 (d, *J* = 7.6 Hz, 2 H, 2 × Ar–CH), 7.75–7.69 (m, 8 H, 8 × Ar–CH), 7.50 (ddd, *J* = 8.0, 6.4, 1.7 Hz, 2 H, 2 × Ar–CH), 7.34 (d, *J* = 7.6 Hz, 2 H, 2 × Ar–CH), 7.27–7.21 (m, 4 H, 4 × Ar–CH), 7.13–7.06 (m, 8 H, 8 × Ar–CH), 6.86–6.82 (m, 4 H, 4 × Ar–CH), 6.65 (td, *J* = 7.2, 1.2 Hz, 2 H, 2 × Ar–CH); $^{13}$C{$^1$H} NMR (101 MHz, $CDCl_3$) $\delta$ 145.2, 144.6, 139.9, 138.1, 136.9, 134.7, 126.9, 125.8, 124.9, 124.7, 124.5, 123.8, 122.4, 121.9, 121.4, 121.0, 120.4, 119.6, 116.3, 111.6, 109.9, 109.5, 109.4. The observed characterization data ($R_f$, mp, IR, $^1$H, $^{13}$C) were consistent with that previously reported.[40]

**X-Ray Crystallography Details**

**3-Aryl-3-Alkyl-Oxetanes**

Structural Analysis

X-Ray crystallography provides a means to assess the structural changes induced by replacing a phenone group with an aryloxetane. See Figures S28, S30, S32, S34 and S36 for a summary of the pertinent bond lengths and angles in **2a**, **2j**, **2q**, **15** and **17**. The following properties are worthy of note:

1. The $C_q$–O through-space distance in oxetanes (2.147 ± 0.014 Å)[41] is significantly longer than the typical distance of the phenone C=O double bond (1.23 ± 0.03 Å).41
2. The steric requirement of the oxetane motif is significantly higher than that of the carbonyl due to the two additional methylene groups. This results in a change in the preferred conformation of the alkyl chain attached to the oxetane. The large substituent on the $sp^3$ carbon on oxetane now lies on the side of the aromatic group (which is now also twisted out of plane) instead of the side of the oxetane (carbonyl) as observed in phenones.
3. Importantly, the aromatic ring is positioned almost orthogonal to the O–$C_q$–$C_q$ plane to reduce steric repulsions. This contrasts with what is typically observed in phenones, where the aromatic group is on the same plane of the C=O bond as to increase $\pi$-conjugation. Consequently, 3-aryl-3-alkyl-oxetanes are overall less planar than their phenone analogues.

**X-Ray crystal structure of ethyl 3-(3-(4-methoxyphenyl)oxetan-3-yl)propanoate (2a)**

Crystals suitable for X-ray analysis were grown by slow evaporation from $CDCl_3$ at 25 °C.

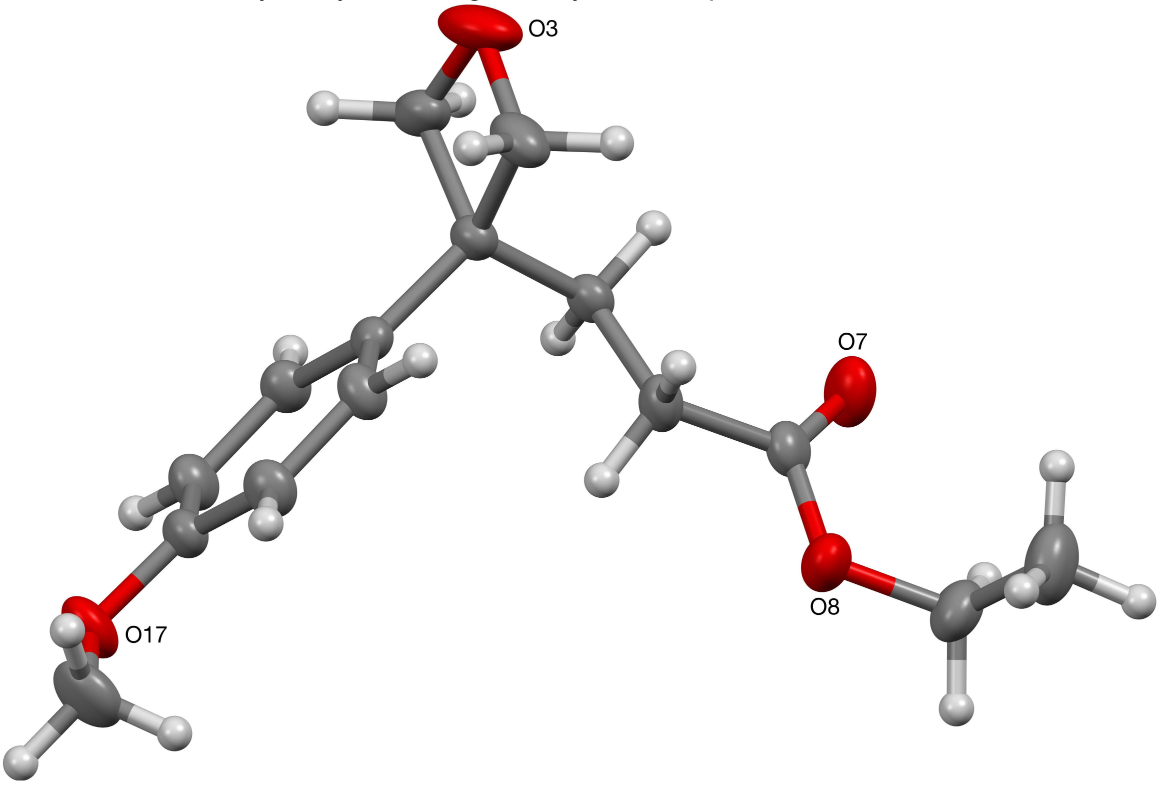


**Figure S27** X-Ray crystal structure of **2a** (50% probability ellipsoids).

*Crystal data for* **2a**: $C_{15}H_{20}O_4$, $M$ = 264.31, monoclinic, $P2_1/n$ (no. 14), $a$ = 8.9682(5), $b$ = 22.7014(11), $c$ = 6.9047(4) Å, β = 91.319(5)°, $V$ = 1405.35(13) Å$^3$, $Z$ = 4, $D_c$ = 1.249 g cm$^{-3}$, μ(Mo-Kα) = 0.090 mm$^{-1}$, $T$ = 173 K, colorless blocks, Agilent Xcalibur 3 E diffractometer; 2998 independent measured reflections ($R_{int}$ = 0.0247), $F^2$ refinement,[X1,X2] $R_1$(obs) = 0.0484, $wR_2$(all) = 0.1199 ($R_1 = \Sigma||F_o| - |F_c||/\Sigma|F_o|$; $wR_2 = \{\Sigma[w(F_o^2 - F_c^2)^2] / \Sigma[w(F_o^2)^2]\}^{1/2}$; $w^{-1} = \sigma^2(F_o^2) + (aP)^2 + bP$), 2247 independent observed absorption-corrected reflections [$|F_o| > 4\sigma(|F_o|)$, completeness to $\theta_{full}$(25.2°) = 99.8% {completeness to 0.84 Å resolution}], 175 parameters. CCDC 2184087.

An inspection of the packing of the molecules in the structure of **2a** does not reveal any notable intermolecular interactions, the only potential contact of any note being an edge-to-face C–H···π approach between the C15–H hydrogen atom on the aromatic ring of the *p*-methoxy phenyl group in one molecule and the centroid of the same ring in a glide-related counterpart [H··· π distance 3.16 Å, C–H··· π angle 166°, H··· π vector inclined by *ca*. 71° to the mean plane of the ring], but this is far too long to be considered a significant interaction.

*Most relevant bond lengths and angles:*

| ***Bond lengths*** | ***Bond angles*** | ***Torsion angles*** |
| --- | --- | --- |

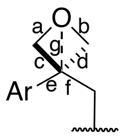


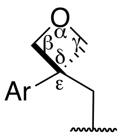


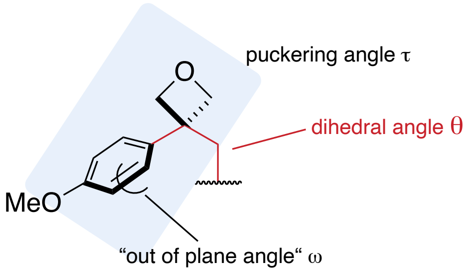


a = C(2)–O(3) = 1.44 Å
b = C(4)–O(3) = 1.44 Å
c = C(1)–C(2) = 1.54 Å
d = C(1)–C(4) = 1.54 Å
e = C(1)–C(11) = 1.51 Å
f = C(1)–C(5) = 1.53 Å
g[a] = C(1)–O(3) = 2.14 Å

α = C(2)–O(3)–C(4) = 91.5 °
β = O(3)–C(2)–C(1) = 91.9 °
γ = O(3)–C(4)–C(1) = 91.8 °
δ = C(2)–C(1)–C(4) = 84.2 °
ε = C(11)–C(1)–C(5) = 112.2 °

τ = O(3)–C(2)–C(4)–C(1) = 8.1 °
ω = O(3)–C(1)–C(11)–C(16) = 81.1 °
θ = C(6)–C(5)–C(1)–C(11) = 60.0 °

**Figure S28** Most relevant bond lengths and angles in the crystal structure of **2a**. [a]g is the through-space distance between C(1) and O(3), not a bond length. This distance can be directly compared to the C=O bond length of phenones.

**X-Ray crystal structure of methyl 2-((*tert*-butoxycarbonyl)amino)-3-(3-(4-methoxyphenyl)oxetan-3-yl)propanoate (2j)**

Crystals suitable for X-ray analysis were grown by slow evaporation from acetone at 25 °C.

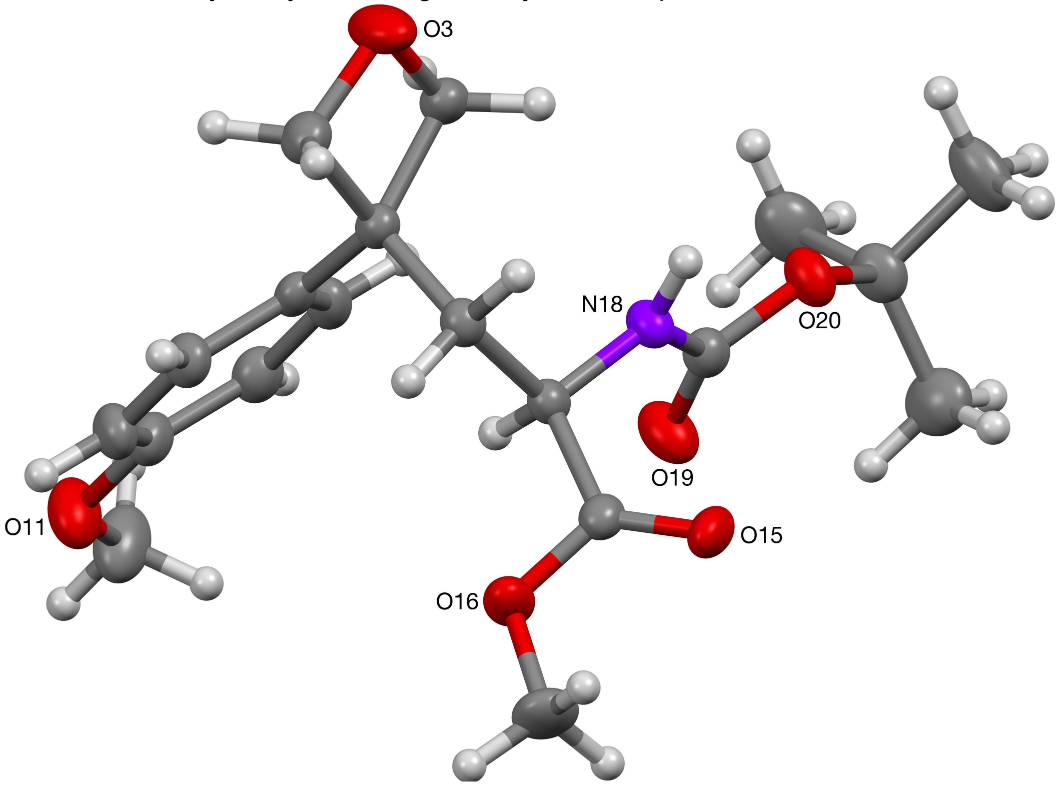


**Figure S29** X-Ray crystal structure of **2j** (50% probability ellipsoids).

*Crystal data for* **2j**: $C_{19}H_{27}NO_6$, $M$ = 365.41, monoclinic, $P2_1/n$ (no. 14), $a$ = 10.3444(4), $b$ = 13.5829(5), $c$ = 14.1806(6) Å, β = 100.532(4)°, $V$ = 1958.90(13) Å$^3$, $Z$ = 4, $D_c$ = 1.239 g cm$^{-3}$, μ(Mo-Kα) = 0.092 mm$^{-1}$, $T$ = 173 K, colorless blocks, Agilent Xcalibur 3 E diffractometer; 4138 independent measured reflections ($R_{int}$ = 0.0278), $F^2$ refinement,[X1,X2] $R_1$(obs) = 0.0447, $wR_2$(all) = 0.1115 ($R_1 = \Sigma||F_o| - |F_c||/\Sigma|F_o|$; $wR_2 = \{\Sigma[w(F_o^2 - F_c^2)^2] / \Sigma[w(F_o^2)^2]\}^{1/2}$; $w^{-1} = \sigma^2(F_o^2) + (aP)^2 + bP$), 3061 independent observed absorption-corrected reflections [$|F_o| > 4\sigma(|F_o|)$, completeness to $\theta_{full}$(25.2°) = 99.9% {completeness to 0.84 Å resolution}], 244 parameters. CCDC 2184088.

The N18–H hydrogen atom was located from Δ*F* maps and refined freely subject to N–H distance constraints of 0.90 Å.

The only intermolecular interaction of note in the structure of **2j** is an N–H···O hydrogen bond between the N18 amino nitrogen in one molecule and the O15 oxygen atom in a centrosymmetrically related molecule with N···O and H···O separations of 2.9638(17) and 2.076(2) Å, and an N–H···O angle of 168.5(8)° [N–H distance constrained to 0.90 Å].

*Most relevant bond lengths and angles:*

***Bond lengths*** ***Bond angles*** ***Torsion angles***

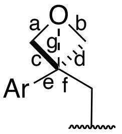


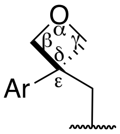


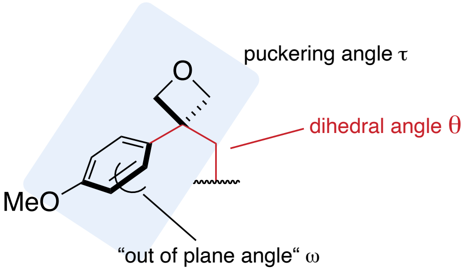


a = C(2)–O(3) = 1.44 Å
b = C(4)–O(3) = 1.44 Å
c = C(1)–C(2) = 1.55 Å
d = C(1)–C(4) = 1.55 Å
e = C(1)–C(5) = 1.51 Å
f = C(1)–C(13) = 1.53 Å
g[a] = C(1)–O(3) = 2.15 Å

$\alpha$ = C(2)–O(3)–C(4) = 91.6 °
$\beta$ = O(3)–C(2)–C(1) = 92.1 °
$\gamma$ = O(3)–C(4)–C(1) = 91.9 °
$\delta$ = C(2)–C(1)–C(4) = 83.8 °
$\varepsilon$ = C(5)–C(1)–C(13) = 110.1 °

$\tau$ = O(3)–C(2)–C(4)–C(1) = 8.0 °
$\omega$ = O(3)–C(1)–C(5)–C(10) = 82.5 °
$\theta$ = C(14)–C(13)–C(1)–C(5) = 57.0 °

**Figure S30** Most relevant bond lengths and angles in the crystal structure of **2j**. [a]g is the through-space distance between C(1) and O(3), not a bond length. This distance can be directly compared to the C=O bond length of phenones.

**X-Ray crystal structure of 4-(2-(3-(4-methoxyphenyl)oxetan-3-yl)ethyl)pyridine (2q)**

Crystals suitable for X-ray analysis were grown by slow evaporation from acetone at 25 °C.

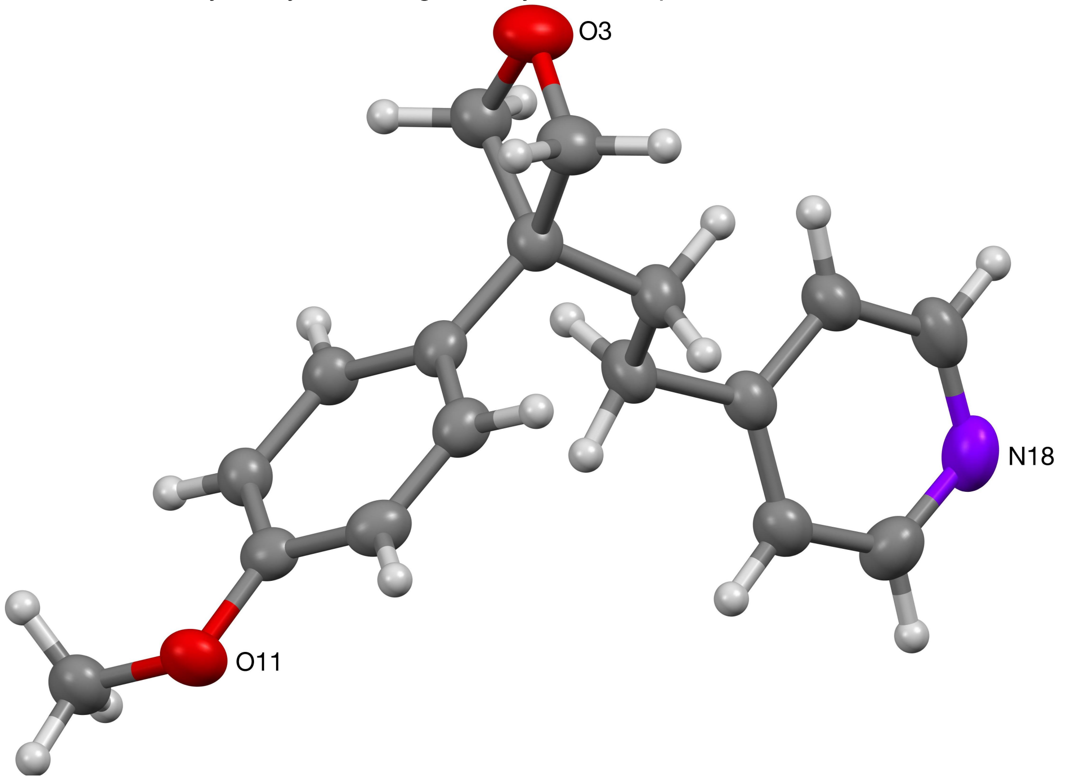


**Figure S31** X-Ray crystal structure of **2q** (50% probability ellipsoids).

*Crystal data for* **2q**: $C_{17}H_{19}NO_2$, *M* = 269.33, monoclinic, $P2_1/n$ (no. 14), *a* = 15.0216(4), *b* = 7.9557(2), *c* = 12.2428(4) Å, β = 99.885(3)°, *V* = 1441.38(7) Å$^3$, *Z* = 4, $D_c$ = 1.241 g cm$^{-3}$, μ(Cu-Kα) = 0.644 mm$^{-1}$, *T* = 173 K, colorless tablets, Agilent Xcalibur PX Ultra A diffractometer; 2832 independent measured reflections ($R_{int}$ = 0.0332), $F^2$ refinement,[X1,X2] $R_1$(obs) = 0.0397, $wR_2$(all) = 0.1084 ($R_1 = \Sigma||F_o| - |F_c||/\Sigma|F_o|$; $wR_2 = \{\Sigma[w(F_o^2 - F_c^2)^2] / \Sigma[w(F_o^2)^2]\}^{1/2}$; $w^{-1} = \sigma^2(F_o^2) + (aP)^2 + bP$), 2154 independent observed absorption-corrected reflections [$|F_o| > 4\sigma(|F_o|)$, completeness to $\theta_{full}$(25.2°) = 99.5% {completeness to 0.84 Å resolution}], 183 parameters. CCDC 2184089.

The aromatic ring of the *p*-methoxy phenyl group in the structure of **2q** is approached on both sides by C–H hydrogen atoms — on one side from one of the methylene hydrogen atoms on C4 of a glide-related molecule and on the other side by one of the C12-methoxy hydrogen atoms of a screw-related molecule. The H···π separations are 2.93 and 2.80 Å respectively, with associated C–H··· π angles of 139 and 148°. The H··· π vectors are inclined by *ca*. 72 and 79° respectively to the mean plane of the ring, and subtended an angle of *ca*. 153° at the ring centroid.

The N18-based pyridyl ring is approached on one side by a C–H hydrogen atom from one of the methylene protons on C2 of a lattice translated molecule with an H··· π separation of 2.70 Å and a C–H··· π angle of 128°, the H··· π vector being inclined by *ca*. 79° to the mean plane of the ring. On the other side the pyridyl ring is involved in a π - π stacking interaction with a centrosymmetrically-related counterpart of itself with centroid···centroid and mean interplanar separations of 3.80 and 3.53 Å respectively, the rings being inclined by 0° (a consequence of the inversion centre).

*Most relevant bond lengths and angles:*

***Bond lengths***

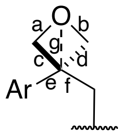


a = C(2)–O(3) = 1.44 Å
b = C(4)–O(3) = 1.45 Å
c = C(1)–C(2) = 1.55 Å
d = C(1)–C(4) = 1.55 Å
e = C(1)–C(5) = 1.50 Å
f = C(1)–C(13) = 1.54 Å
g[a] = C(1)–O(3) = 2.15 Å

***Bond angles***

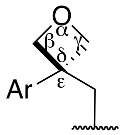


α = C(2)–O(3)–C(4) = 91.4 °
β = O(3)–C(2)–C(1) = 91.9 °
γ = O(3)–C(4)–C(1) = 91.8 °
δ = C(2)–C(1)–C(4) = 83.8 °
ε = C(5)–C(1)–C(13) = 111.8 °

***Torsion angles***

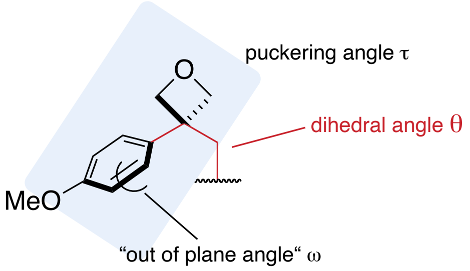


τ = O(3)–C(2)–C(4)–C(1) = 10.5 °
ω = O(3)–C(1)–C(5)–C(10) = 80.3 °
θ = C(14)–C(13)–C(1)–C(5) = 58.9 °

**Figure S32** Most relevant bond lengths and angles in the crystal structure of **2q**. [a]g is the through-space distance between C(1) and O(3), not a bond length. This distance can be directly compared to the C=O bond length of phenones.

**X-Ray crystal structure of 3-(3-(4-methoxyphenyl)oxetan-3-yl)propanoic acid (15)**

Crystals suitable for X-ray analysis were grown by slow evaporation from $CDCl_3$ at 25 °C.

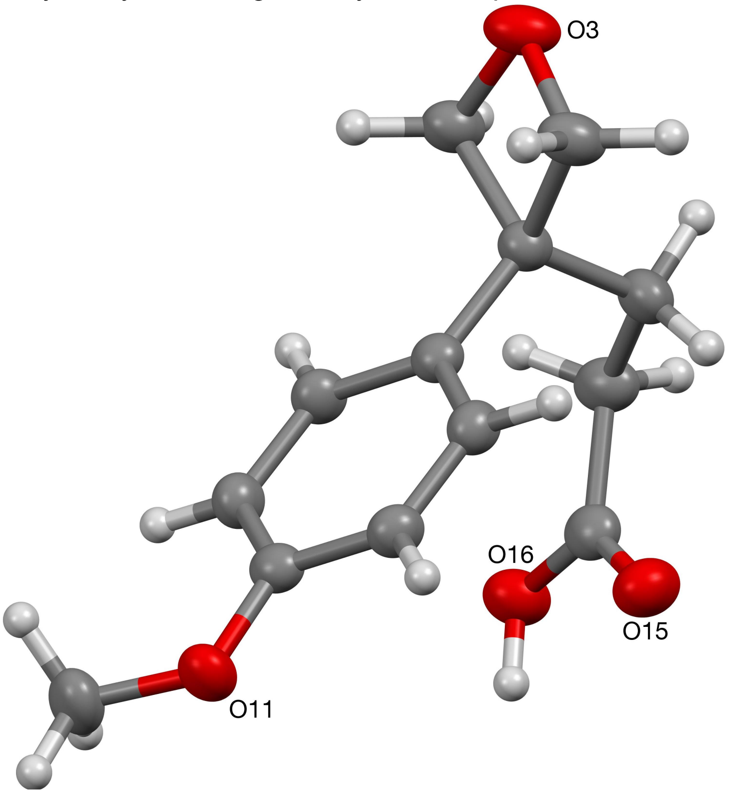


**Figure S33** X-Ray crystal structure of **15** (50% probability ellipsoids).

*Crystal data for* **15**: $C_{13}H_{16}O_4$, $M$ = 236.26, monoclinic, $P2_1/n$ (no. 14), $a$ = 12.1846(5), $b$ = 7.9034(2), $c$ = 12.7761(4) Å, β = 106.398(4)°, $V$ = 1180.29(7) Å$^3$, $Z$ = 4, $D_c$ = 1.330 g cm$^{-3}$, μ(Cu-Kα) = 0.812 mm$^{-1}$, $T$ = 173 K, colorless tablets, Agilent Xcalibur PX Ultra A diffractometer; 2316 independent measured reflections ($R_{int}$ = 0.0305), $F^2$ refinement,[X1,X2] $R_1$(obs) = 0.0397, $wR_2$(all) = 0.1072 ($R_1 = \Sigma||F_o| - |F_c||/\Sigma|F_o|$; $wR_2 = \{\Sigma[w(F_o^2 - F_c^2)^2] / \Sigma[w(F_o^2)^2]\}^{1/2}$; $w^{-1} = \sigma^2(F_o^2) + (aP)^2 + bP$), 1897 independent observed absorption-corrected reflections [$|F_o| > 4\sigma(|F_o|)$, completeness to $\theta_{full}$(25.2°) = 99.5% {completeness to 0.84 Å resolution}], 160 parameters. CCDC 2184090.

The O16–H hydrogen atom was located from Δ$F$ maps and refined freely subject to O–H distance constraints of 0.90 Å.

*Most relevant bond lengths and angles:*

| ***Bond lengths*** | ***Bond angles*** | ***Torsion angles*** |
|---|---|---|
| 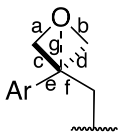 | 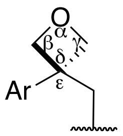 | 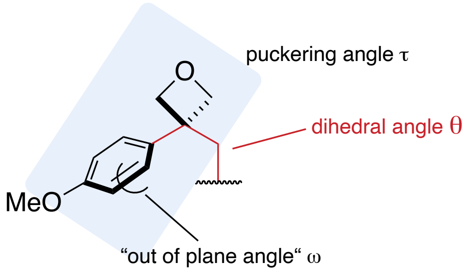 |







a = C(2)–O(3) = 1.46 Å
b = C(4)–O(3) = 1.46 Å
c = C(1)–C(2) = 1.54 Å
d = C(1)–C(4) = 1.54 Å
e = C(1)–C(5) = 1.51 Å
f = C(1)–C(13) = 1.53 Å
g[a] = C(1)–O(3) = 2.15 Å

α = C(2)–O(3)–C(4) = 90.8 °
β = O(3)–C(2)–C(1) = 91.3 °
γ = O(3)–C(4)–C(1) = 91.4 °
δ = C(2)–C(1)–C(4) = 84.6 °
ε = C(5)–C(1)–C(13) = 113.4 °

τ = O(3)–C(2)–C(4)–C(1) = 14.4 °
ω = O(3)–C(1)–C(5)–C(10) = 82.9 °
θ = C(14)–C(13)–C(1)–C(5) = 56.7 °

**Figure S34** Most relevant bond lengths and angles in the crystal structure of **15**. [a]g is the through-space distance between C(1) and O(3), not a bond length. This distance can be directly compared to the C=O bond length of phenones.

**Oxetane–TEMPO adduct**

**X-Ray crystal structure of 1-((3-(4-methoxyphenyl)oxetan-3-yl)oxy)-2,2,6,6-tetramethylpiperidine (17)**

Crystals suitable for X-ray analysis were grown by slow evaporation from $CDCl_3$ at 25 °C.

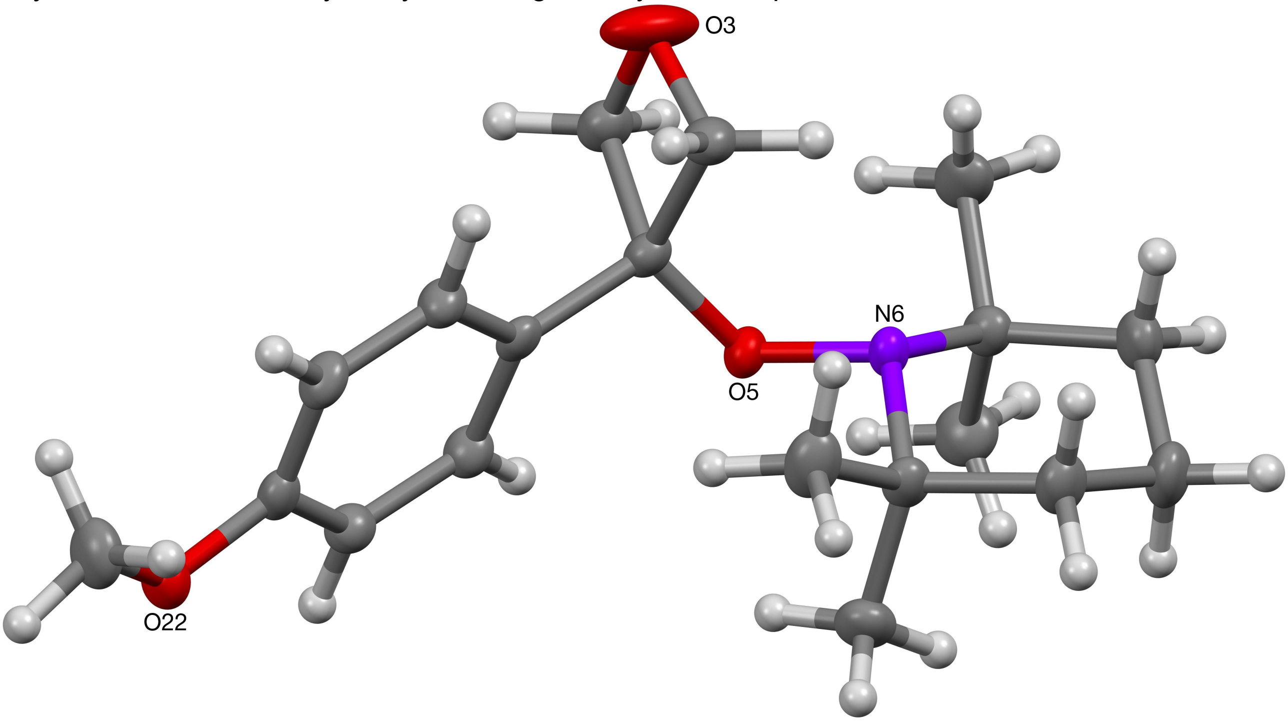


**Figure S35** X-Ray crystal structure of **17** (50% probability ellipsoids).

*Crystal data for* **17**: $C_{19}H_{29}NO_3$, $M$ = 319.43, monoclinic, $P2_1/n$ (no. 14), $a$ = 12.7040(6), $b$ = 7.4293(3), $c$ = 18.8877(8) Å, $\beta$ = 101.415(4)°, $V$ = 1747.40(13) Å$^3$, $Z$ = 4, $D_c$ = 1.214 g cm$^{-3}$, $\mu$(Mo-K$\alpha$) = 0.081 mm$^{-1}$, $T$ = 173 K, colorless needles, Agilent Xcalibur 3 E diffractometer; 3693 independent measured reflections ($R_{int}$ = 0.0326), $F^2$ refinement,[X1,X2] $R_1$(obs) = 0.0467, $wR_2$(all) = 0.1075 ($R_1 = \Sigma||F_o| - |F_c||/\Sigma|F_o|$; $wR_2 = \{\Sigma[w(F_o^2 - F_c^2)^2] / \Sigma[w(F_o^2)^2]\}^{1/2}$; $w^{-1} = \sigma^2(F_o^2) + (aP)^2 + bP$), 2701 independent observed absorption-corrected reflections [$|F_o| > 4\sigma(|F_o|)$, completeness to $\theta_{full}$(25.2°) = 99.9% {completeness to 0.84 Å resolution}], 214 parameters. CCDC 2184091.

*Most relevant bond lengths and angles:*

***Bond lengths***

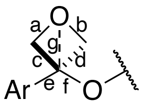


a = C(2)–O(3) = 1.44 Å
b = C(4)–O(3) = 1.44 Å
c = C(1)–C(2) = 1.55 Å
d = C(1)–C(4) = 1.53 Å
e = C(1)–C(16) = 1.52 Å
f = C(1)–O(5) = 1.45 Å
g[a] = C(1)–O(3) = 2.13 Å

***Bond angles***

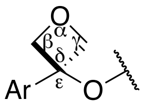


$\alpha$ = C(2)–O(3)–C(4) = 91.8 °
$\beta$ = O(3)–C(2)–C(1) = 91.2 °
$\gamma$ = O(3)–C(4)–C(1) = 91.7 °
$\delta$ = C(2)–C(1)–C(4) = 84.3 °
$\varepsilon$ = C(16)–C(1)–O(5) = 108.1 °

***Torsion angles***

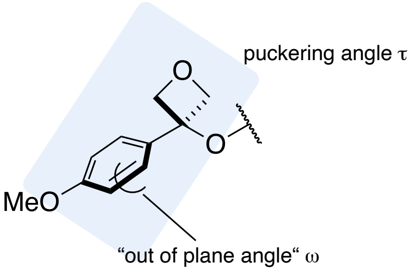


$\tau$ = O(3)–C(2)–C(4)–C(1) = 10.2 °
$\omega$ = O(3)–C(1)–C(5)–C(10) = 38.8 °

**Figure S36** Most relevant bond lengths and angles in the crystal structure of **17**. [a]g is the through-space distance between C(1) and O(3), not a bond length. This distance can be directly compared to the C=O bond length of phenones.

***gem*-Dimethyl Dimer**

**X-Ray crystal structure of 4,4'-(2,3-dimethylbutane-2,3-diyl)bis(methoxybenzene) (VII-B)**

Crystals suitable for X-ray analysis were grown by slow evaporation from $CDCl_3$ at 25 °C.

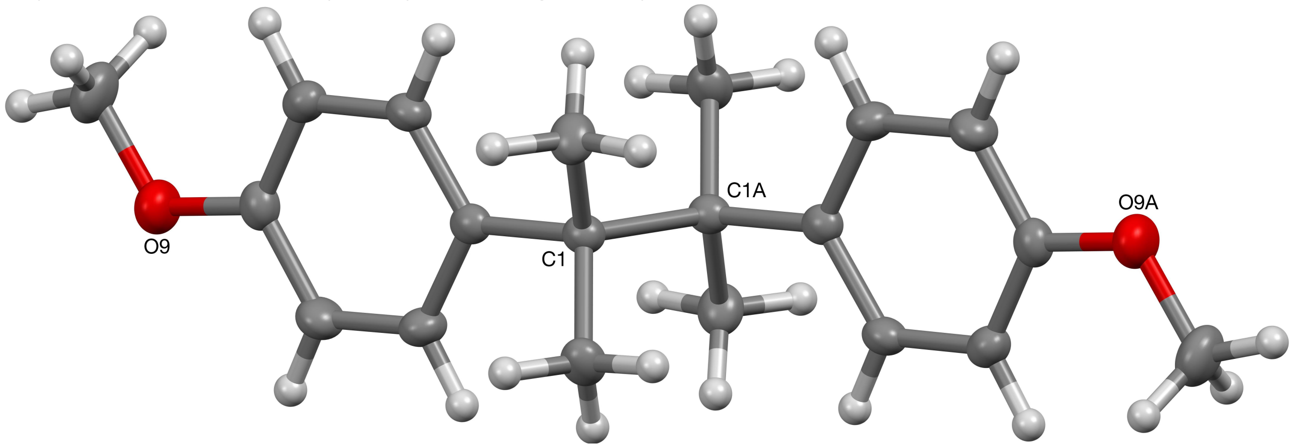


**Figure S37** X-Ray crystal structure of **VII-B** (50% probability ellipsoids).

*Crystal data for* **VII-B**: $C_{20}H_{26}O_2$, $M$ = 298.41, monoclinic, $P2_1/n$ (no. 14), $a$ = 6.7658(2), $b$ = 7.5974(3), $c$ = 15.7294(5) Å, β = 91.310(3)°, $V$ = 808.32(5) Å$^3$, $Z$ = 2 {the molecule has crystallographic $C_i$ symmetry}, $D_c$ = 1.226 g cm$^{-3}$, μ(Mo-Kα) = 0.077 mm$^{-1}$, $T$ = 173 K, colorless blocks, Agilent Xcalibur 3 E diffractometer; 1832 independent measured reflections ($R_{int}$ = 0.0282), $F^2$ refinement,[X1,X2] $R_1$(obs) = 0.0411, $wR_2$(all) = 0.1019 ($R_1 = \Sigma||F_o| - |F_c||/\Sigma|F_o|$; $wR_2 = \{\Sigma[w(F_o^2 - F_c^2)^2] / \Sigma[w(F_o^2)^2]\}^{1/2}$; $w^{-1} = \sigma^2(F_o^2) + (aP)^2 + bP$), 1491 independent observed absorption-corrected reflections [$|F_o| > 4\sigma(|F_o|)$, completeness to $\theta_{full}$(25.2°) = 100.0% {completeness to 0.84 Å resolution}], 104 parameters. CCDC 2184092.

The structure of **VII-B** sits across a centre of symmetry at the middle of the C1–C1A bond.

## $^{1}H$, $^{13}C$, $^{19}F$ and $^{31}P$ NMR Spectra of Selected Compounds

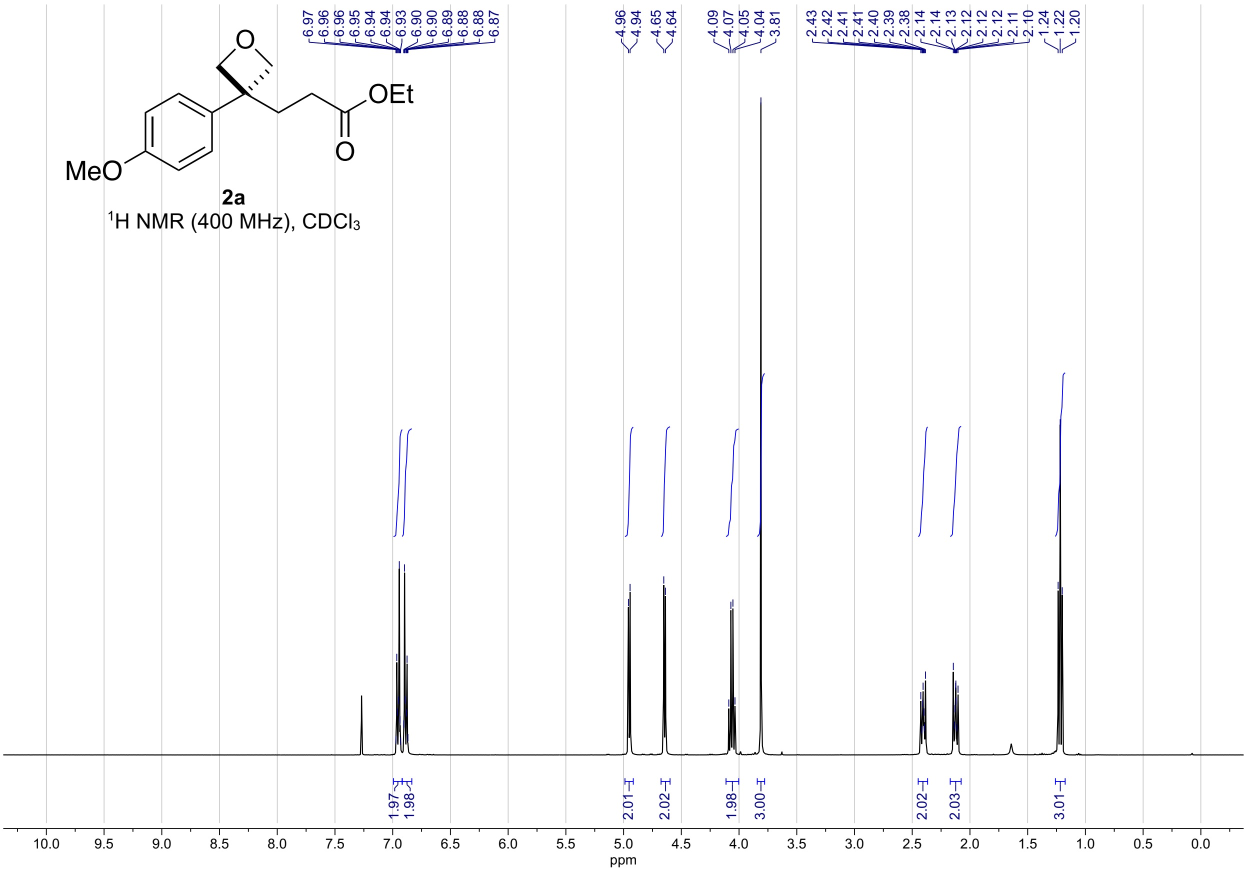
OEt
O
MeO
O
2a
$^{1}$H NMR (400 MHz), CDCl$_3$
6.97 6.96 6.96 6.95 6.94 6.94 6.93 6.90 6.90 6.89 6.88 6.88 6.87
4.96 4.94 4.65 4.64
4.09 4.07 4.05 4.04 3.81
2.43 2.42 2.41 2.41 2.40 2.39 2.38 2.14 2.14 2.13 2.12 2.12 2.12 2.11 2.10 1.24 1.22 1.20
1.97 1.98 2.01 2.02 1.98 3.00 2.02 2.03 3.01
10.0 9.5 9.0 8.5 8.0 7.5 7.0 6.5 6.0 5.5 5.0 4.5 4.0 3.5 3.0 2.5 2.0 1.5 1.0 0.5 0.0
ppm

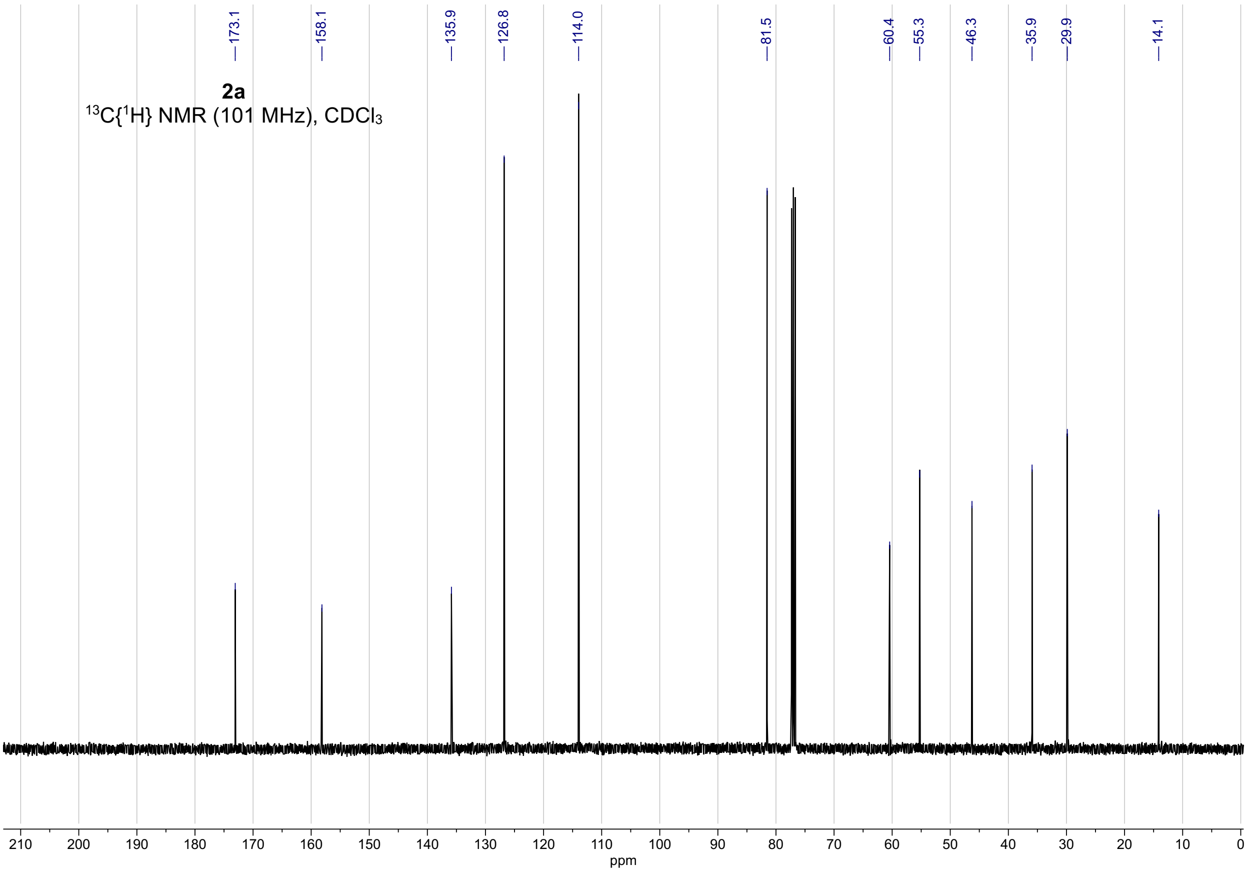
173.1
158.1
135.9
126.8
114.0
81.5
60.4
55.3
46.3
35.9
29.9
14.1
2a
$^{13}$C{$^{1}$H} NMR (101 MHz), CDCl$_3$
210 200 190 180 170 160 150 140 130 120 110 100 90 80 70 60 50 40 30 20 10 0
ppm

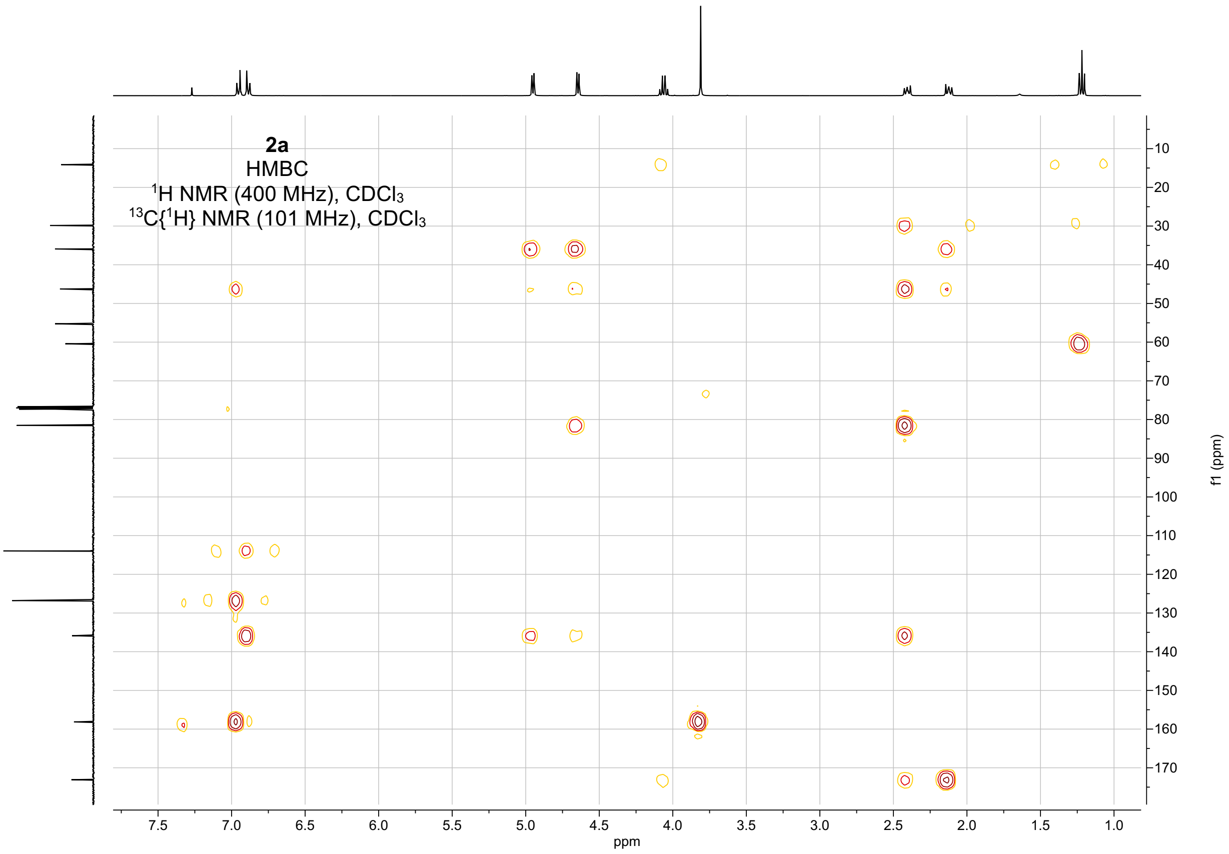
2a
HMBC
1H NMR (400 MHz), CDCl3
13C{1H} NMR (101 MHz), CDCl3
ppm
f1 (ppm)

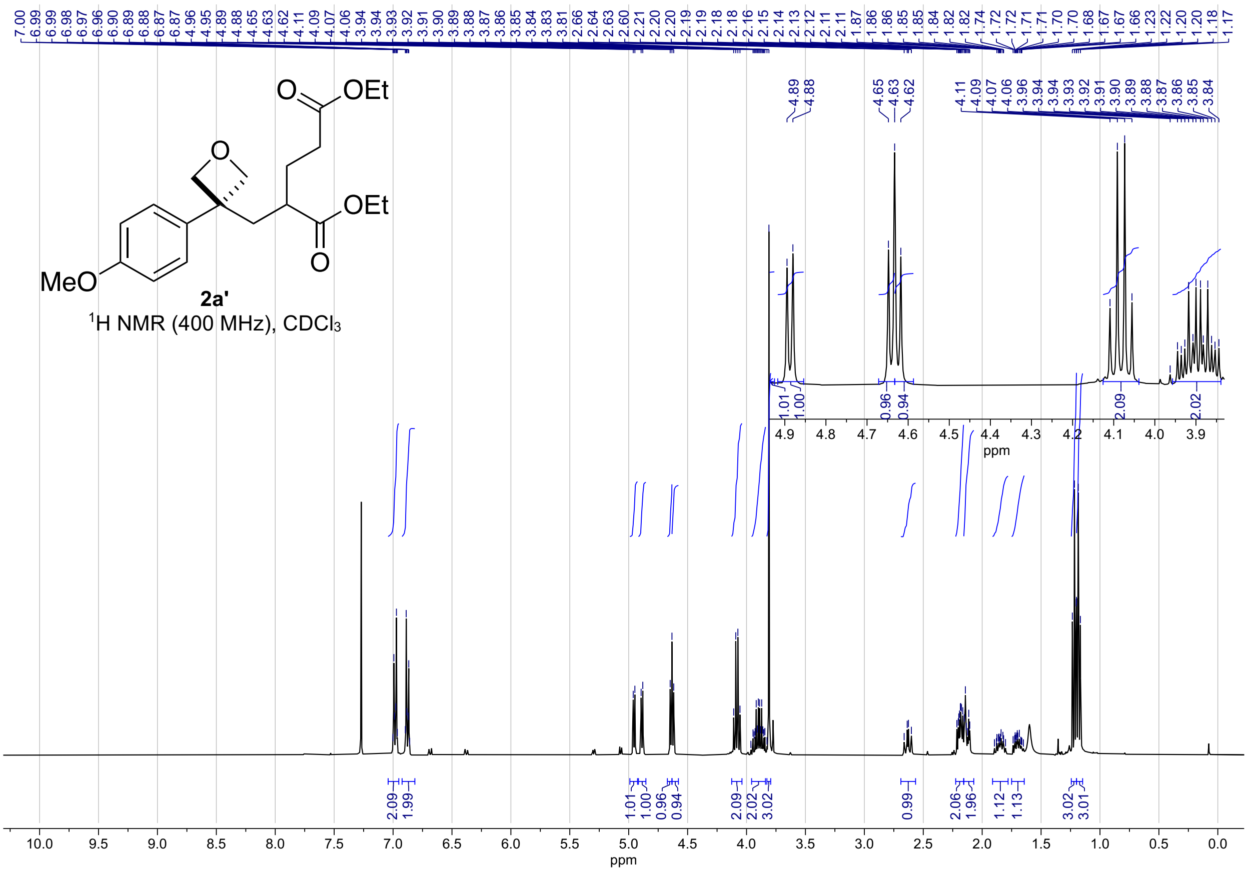

OEt
O
OEt
O
O
MeO
2a'
¹H NMR (400 MHz), CDCl₃
ppm


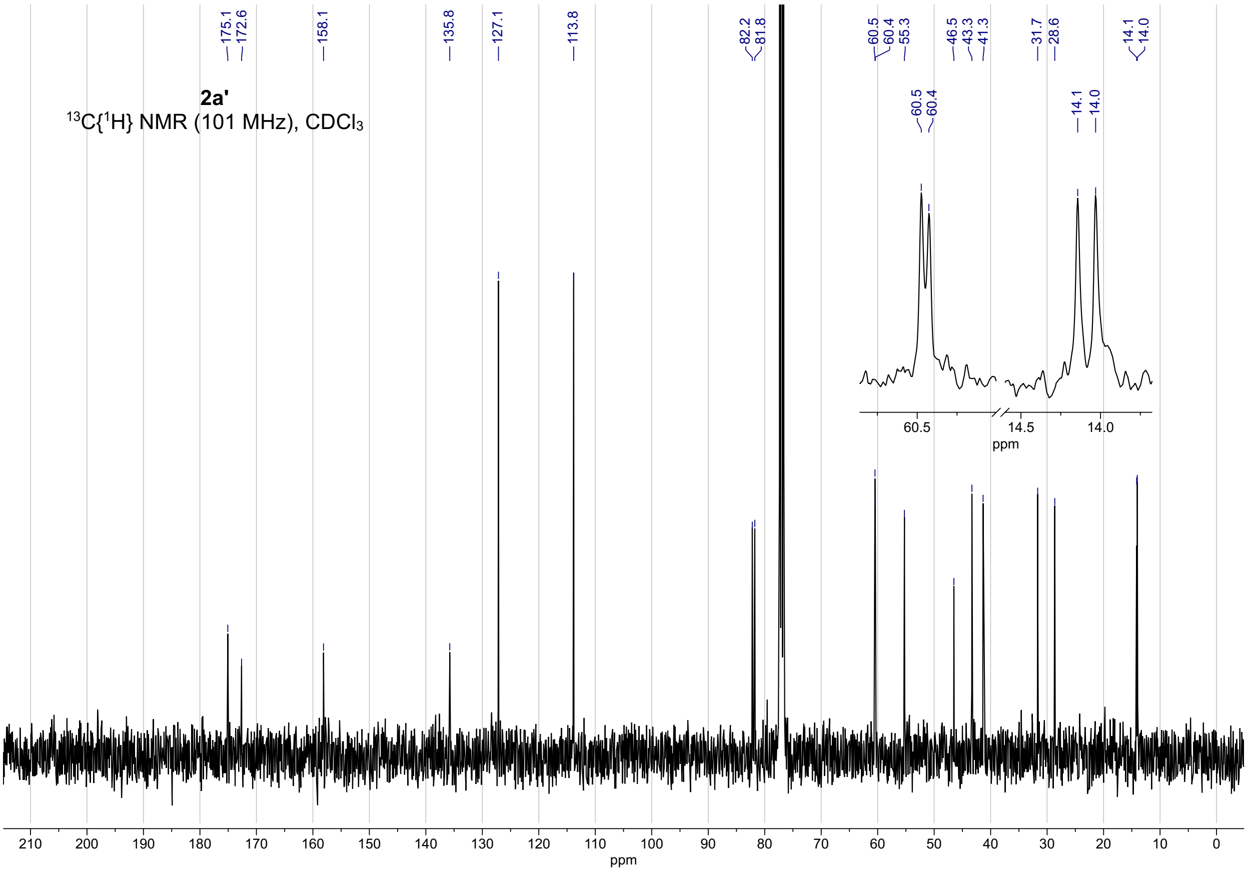

2a'
¹³C{¹H} NMR (101 MHz), CDCl₃
175.1
172.6
158.1
135.8
127.1
113.8
82.2
81.8
60.5
60.4
55.3
46.5
43.3
41.3
31.7
28.6
14.1
14.0
ppm

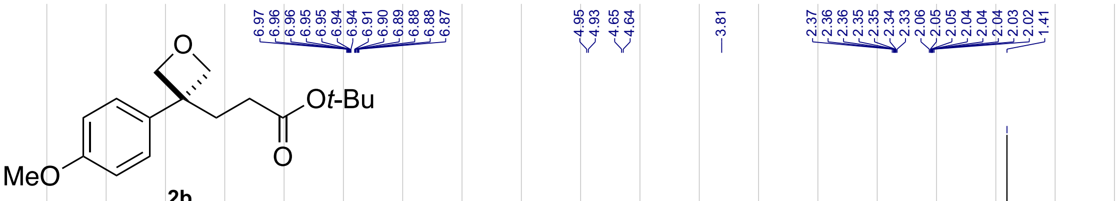

**2b**
$^1$H NMR (400 MHz), $CDCl_3$

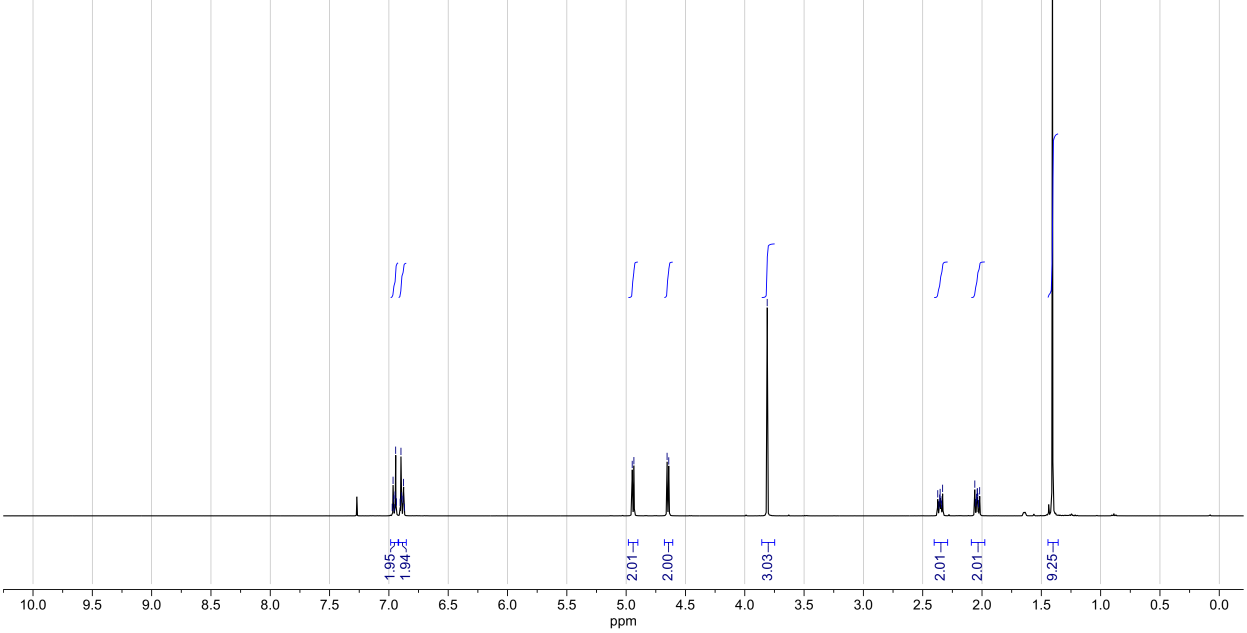


**2b**
$^{13}$C{$^1$H} NMR (101 MHz), $CDCl_3$

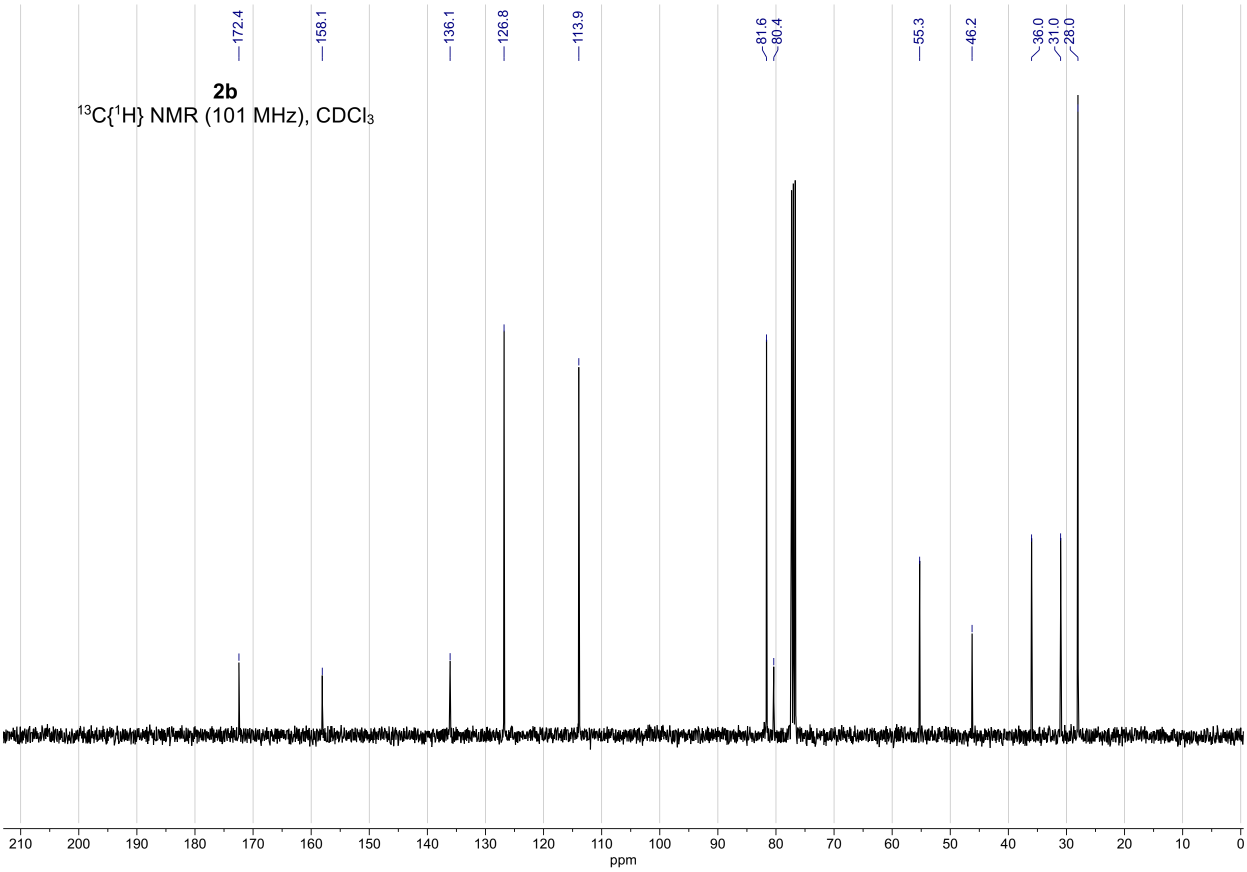

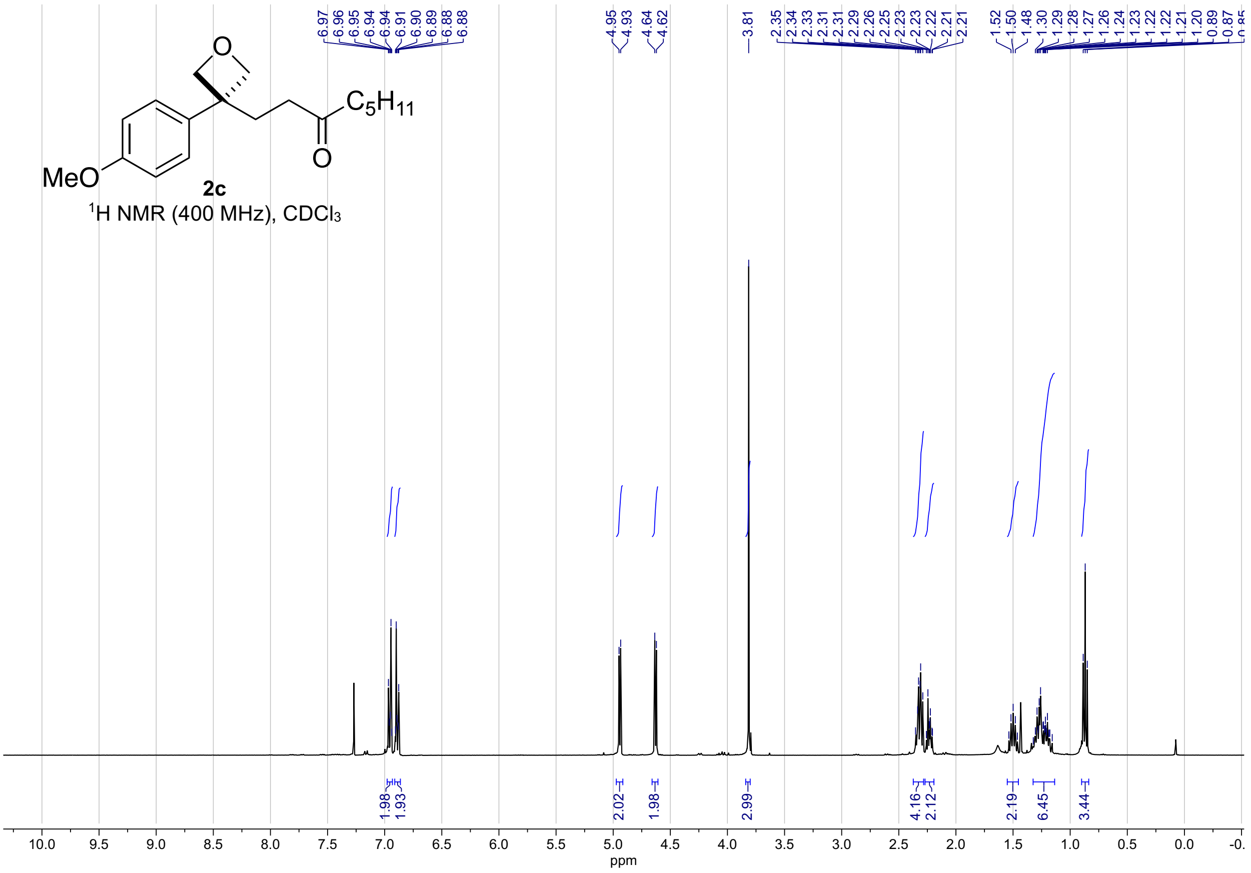
MeO
O
C5H11
O
2c
1H NMR (400 MHz), CDCl3
6.97 6.96 6.95 6.94 6.94 6.91 6.90 6.89 6.88 6.88
4.95 4.93 4.64 4.62
3.81
2.35 2.34 2.33 2.31 2.31 2.29 2.26 2.25 2.23 2.23 2.22 2.21 2.21
1.52 1.50 1.48 1.30 1.29 1.28 1.27 1.26 1.24 1.23 1.22 1.22 1.21 1.20 0.89 0.87
1.98 1.93 2.02 1.98 2.99 4.16 2.12 2.19 6.45 3.44
10.0 9.5 9.0 8.5 8.0 7.5 7.0 6.5 6.0 5.5 5.0 4.5 4.0 3.5 3.0 2.5 2.0 1.5 1.0 0.5 0.0
ppm

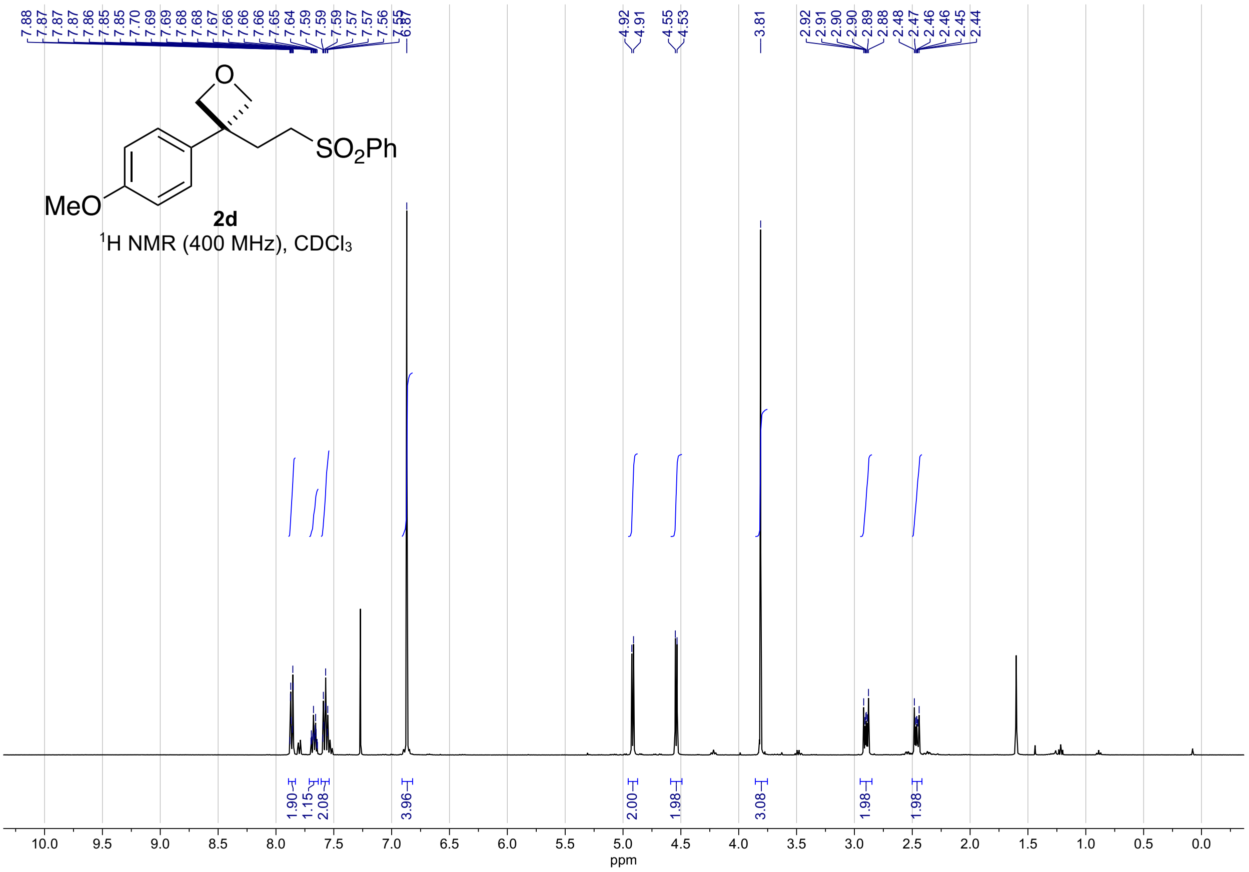
7.88 7.87 7.87 7.86 7.85 7.85 7.70 7.69 7.69 7.68 7.68 7.67 7.66 7.66 7.66 7.65 7.64 7.59 7.59 7.59 7.57 7.57 7.56 7.55 6.87
4.92 4.91 4.55 4.53
3.81
2.92 2.91 2.90 2.90 2.89 2.88 2.48 2.47 2.46 2.46 2.45 2.44
O
SO2Ph
MeO
2d
1H NMR (400 MHz), CDCl3
1.90 1.15 2.08 3.96 2.00 1.98 3.08 1.98 1.98
ppm

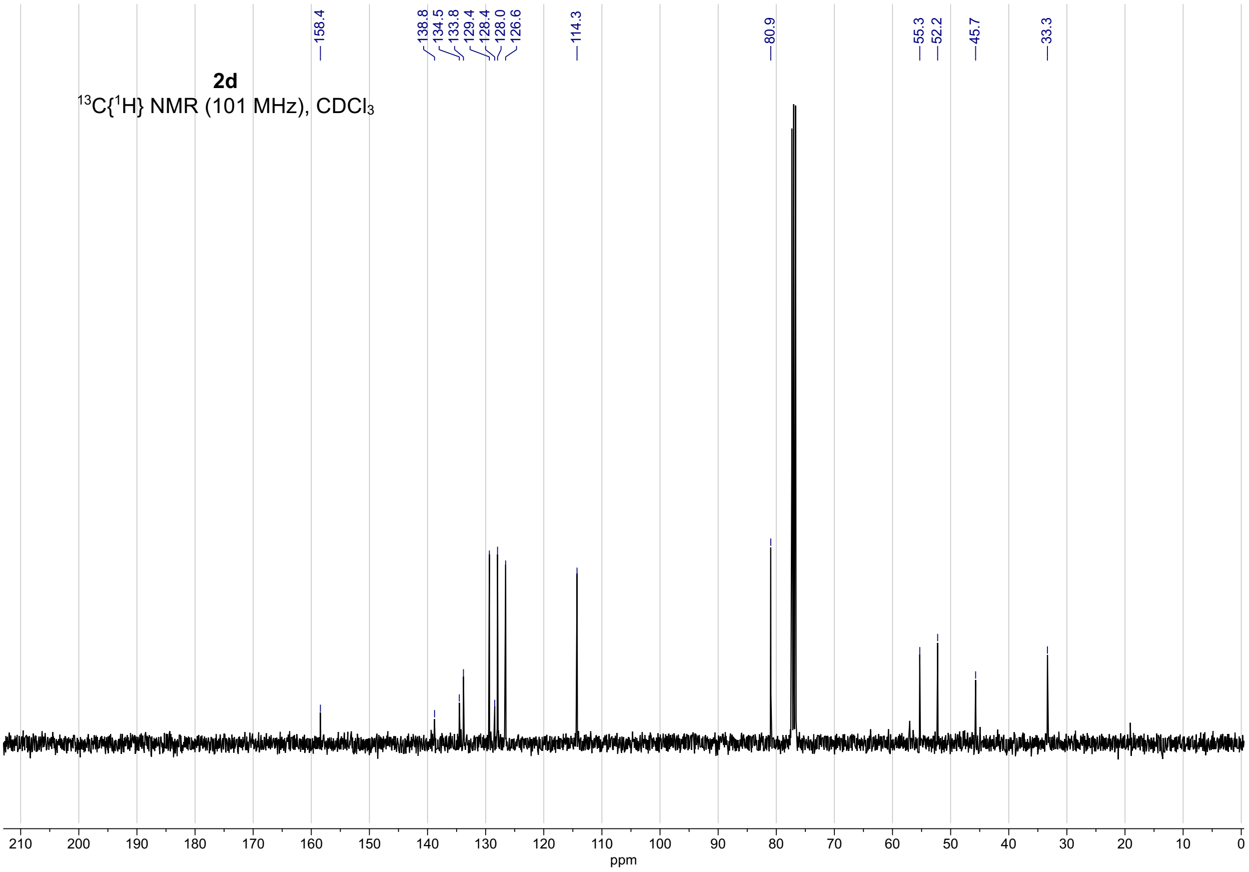
158.4
138.8 134.5 133.8 129.4 128.4 128.0 126.6
114.3
80.9
55.3 52.2 45.7 33.3
2d
13C{1H} NMR (101 MHz), CDCl3
ppm

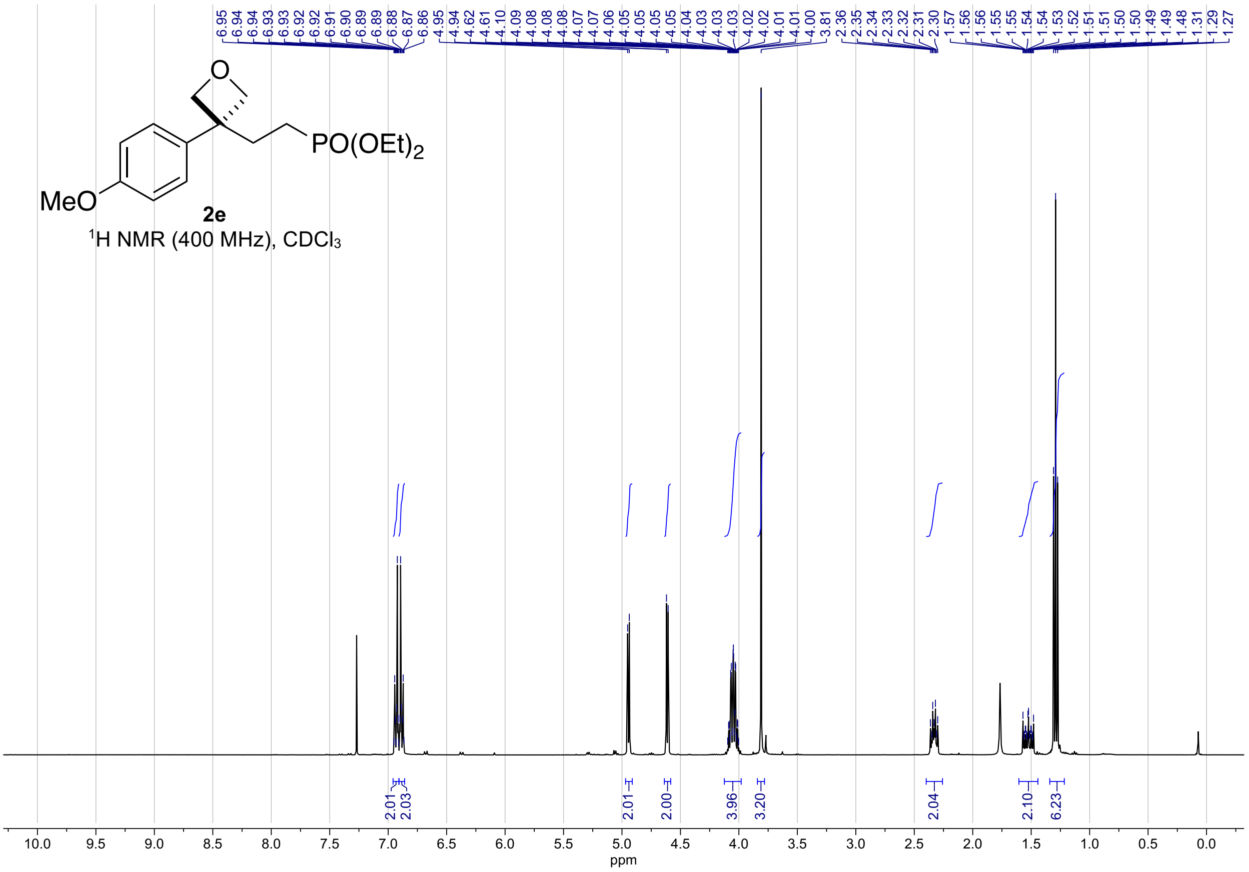
MeO
PO(OEt)2
2e
1H NMR (400 MHz), CDCl3
ppm

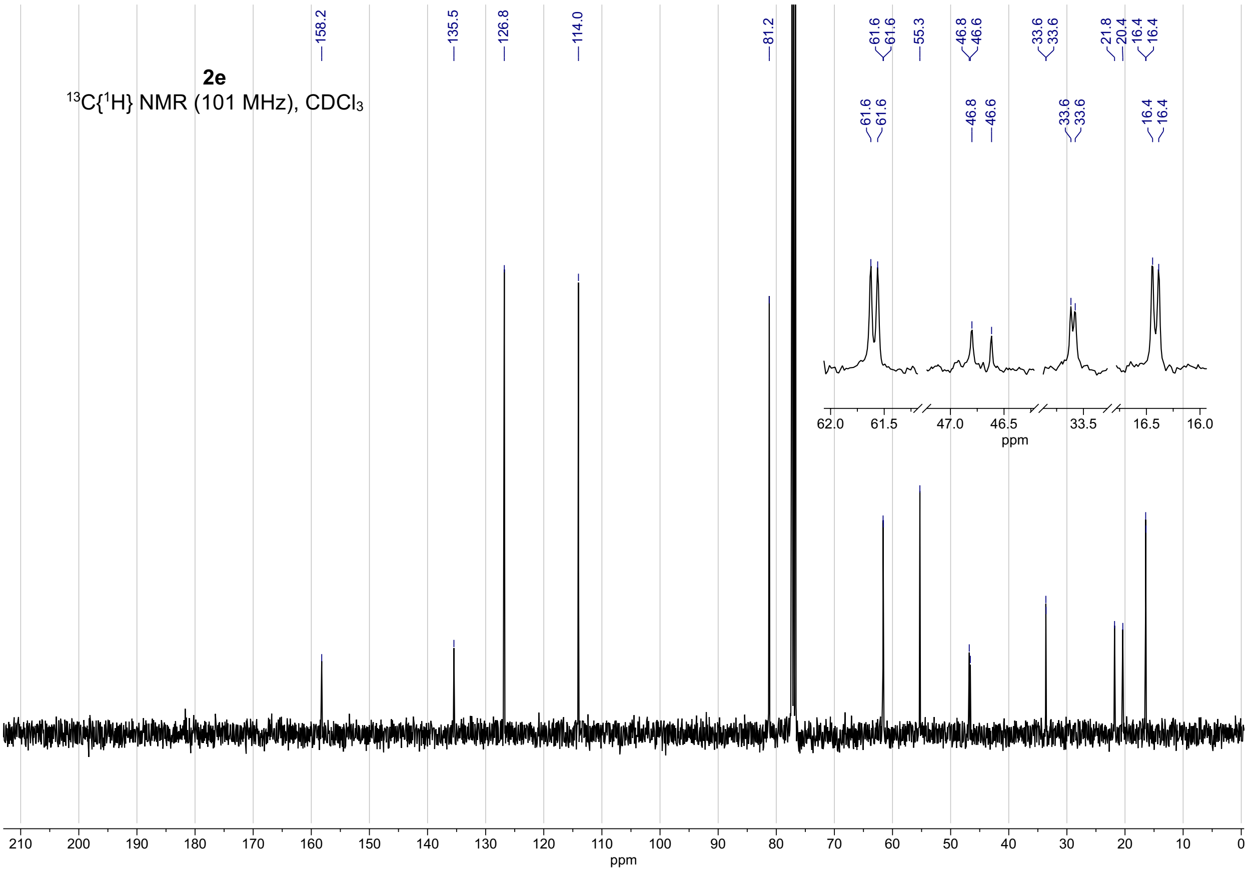
2e
13C{1H} NMR (101 MHz), CDCl3
ppm

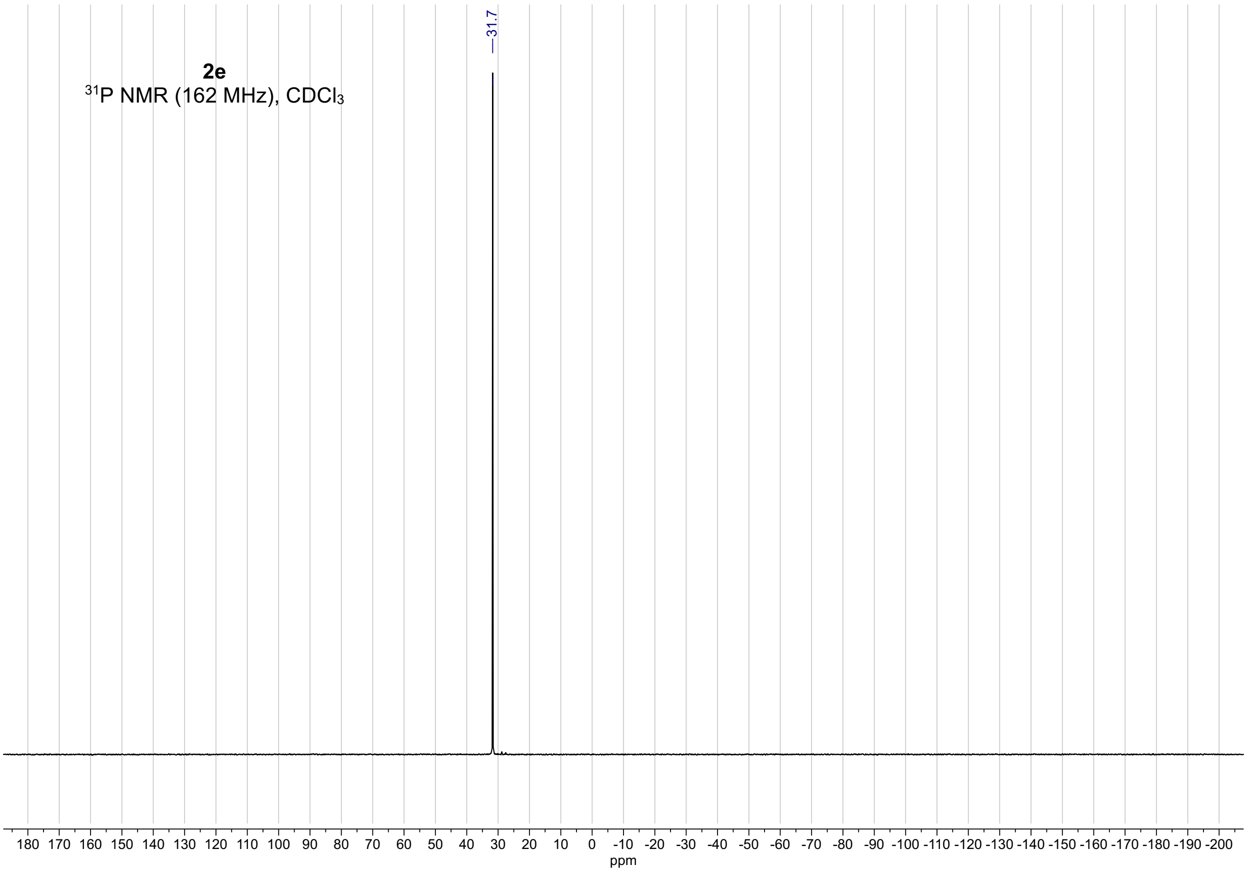

31.7
2e
31P NMR (162 MHz), CDCl3
180 170 160 150 140 130 120 110 100 90 80 70 60 50 40 30 20 10 0 -10 -20 -30 -40 -50 -60 -70 -80 -90 -100 -110 -120 -130 -140 -150 -160 -170 -180 -190 -200
ppm

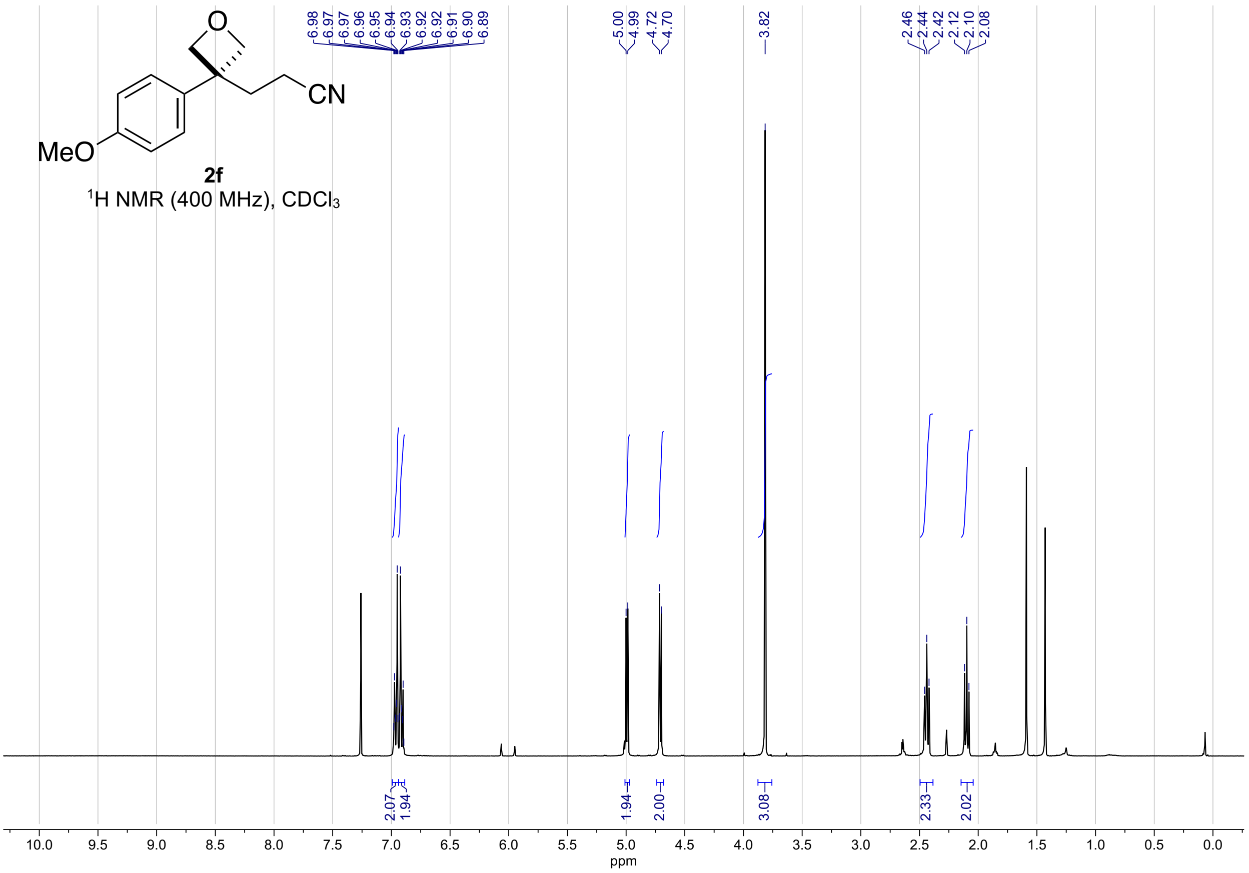
MeO
O
CN
2f
¹H NMR (400 MHz), CDCl₃
6.98 6.97 6.97 6.96 6.95 6.94 6.93 6.92 6.92 6.91 6.90 6.89
5.00 4.99 4.72 4.70
3.82
2.46 2.44 2.42 2.12 2.10 2.08
2.07 1.94 1.94 2.00 3.08 2.33 2.02
ppm

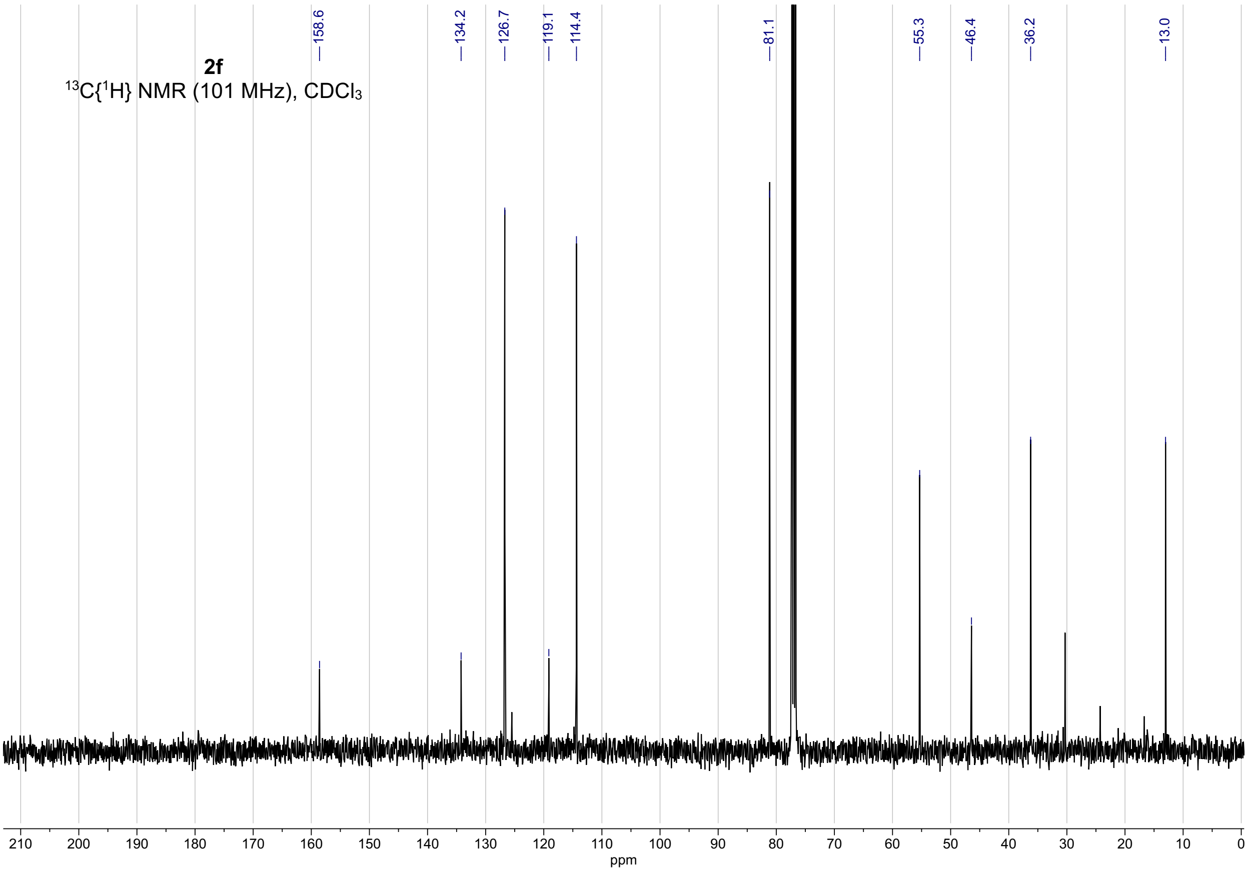
2f
¹³C{¹H} NMR (101 MHz), CDCl₃
158.6 134.2 126.7 119.1 114.4 81.1 55.3 46.4 36.2 13.0
ppm

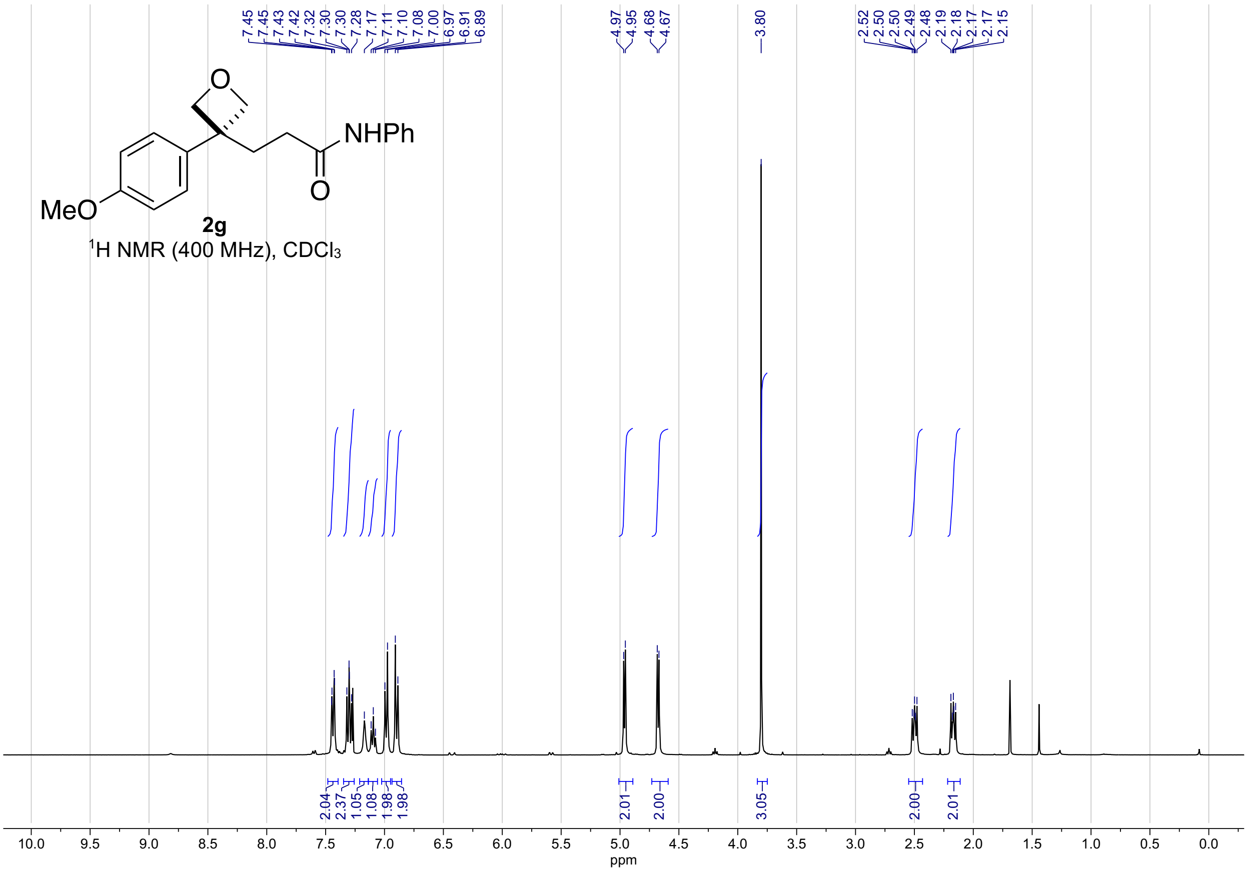


**2g**
$^{1}$H NMR (400 MHz), $CDCl_3$

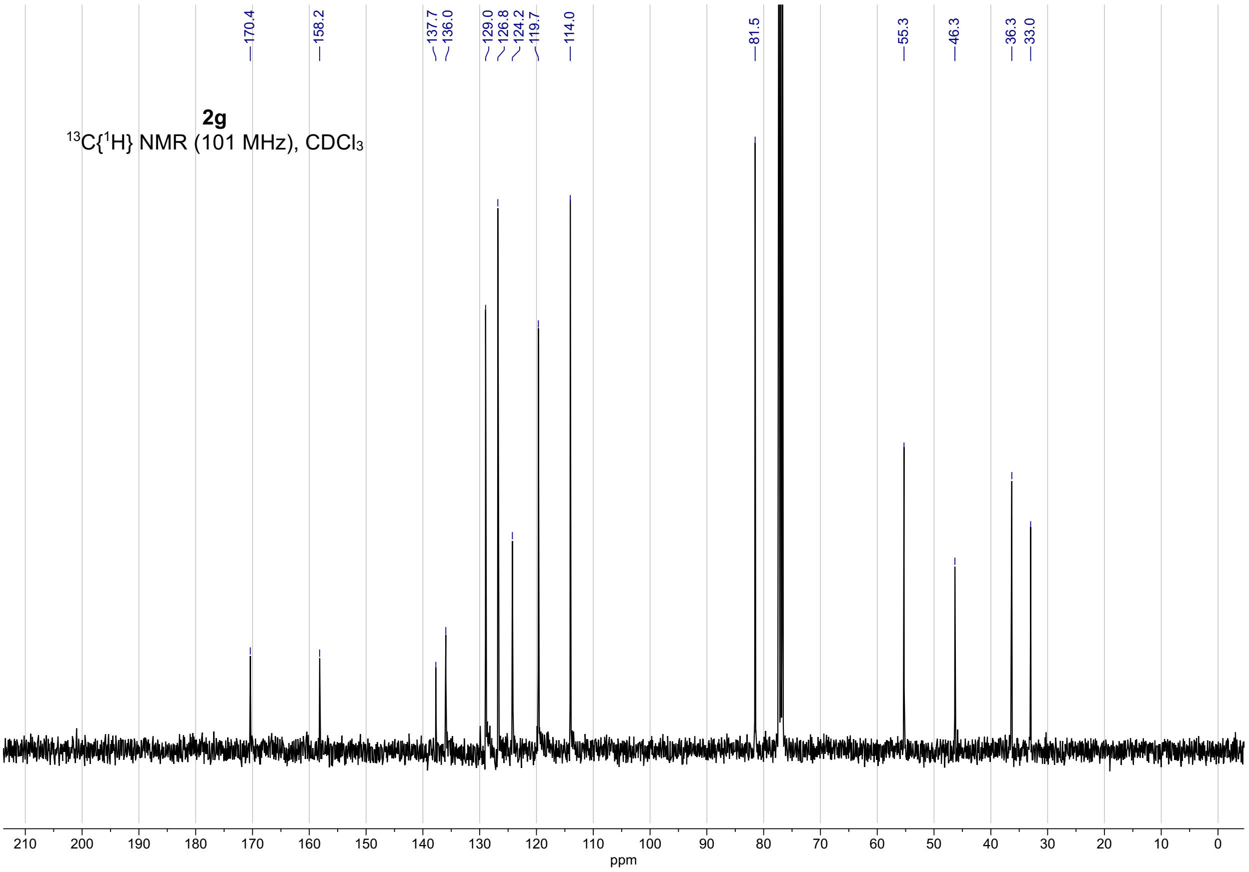


**2g**
$^{13}C\{^{1}H\}$ NMR (101 MHz), $CDCl_3$

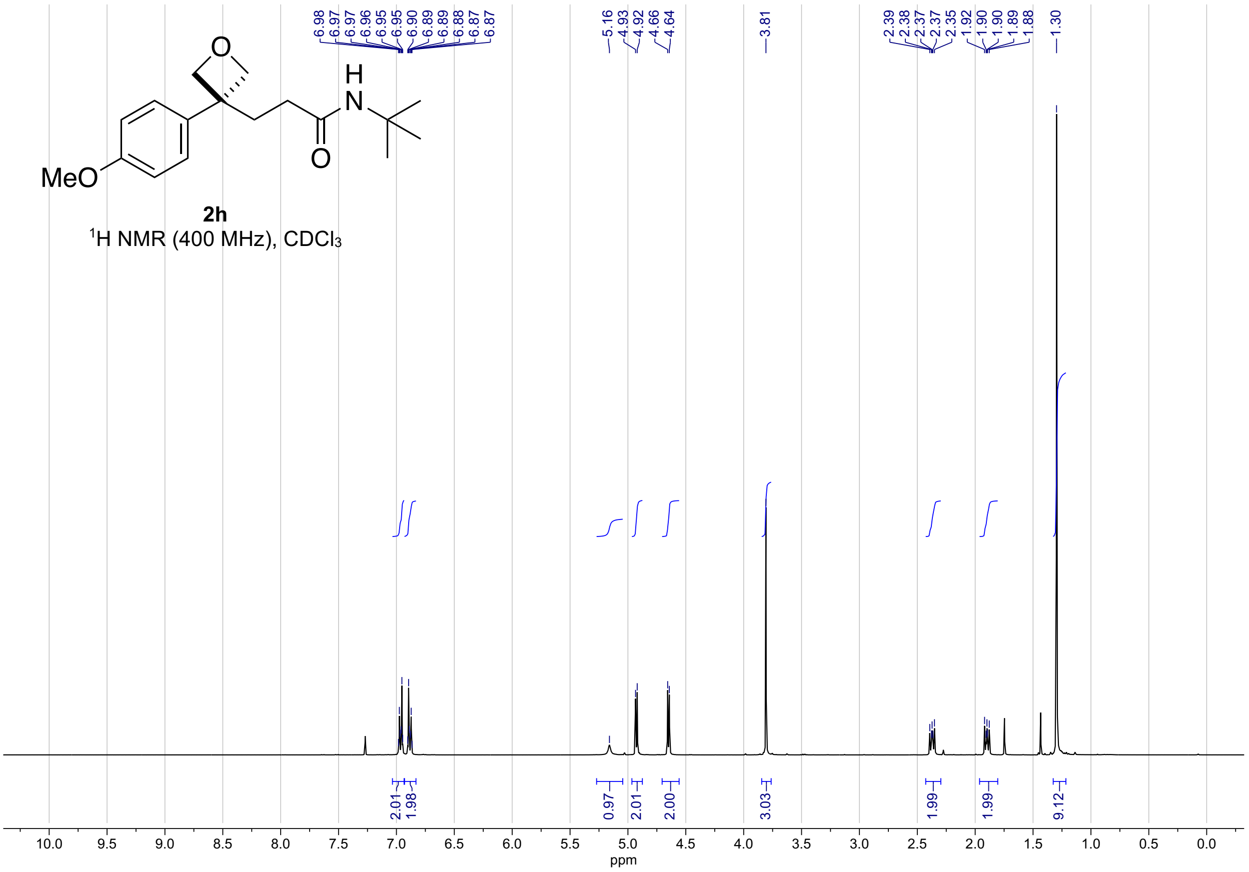


**2h**

$^{1}$H NMR (400 MHz), $CDCl_3$

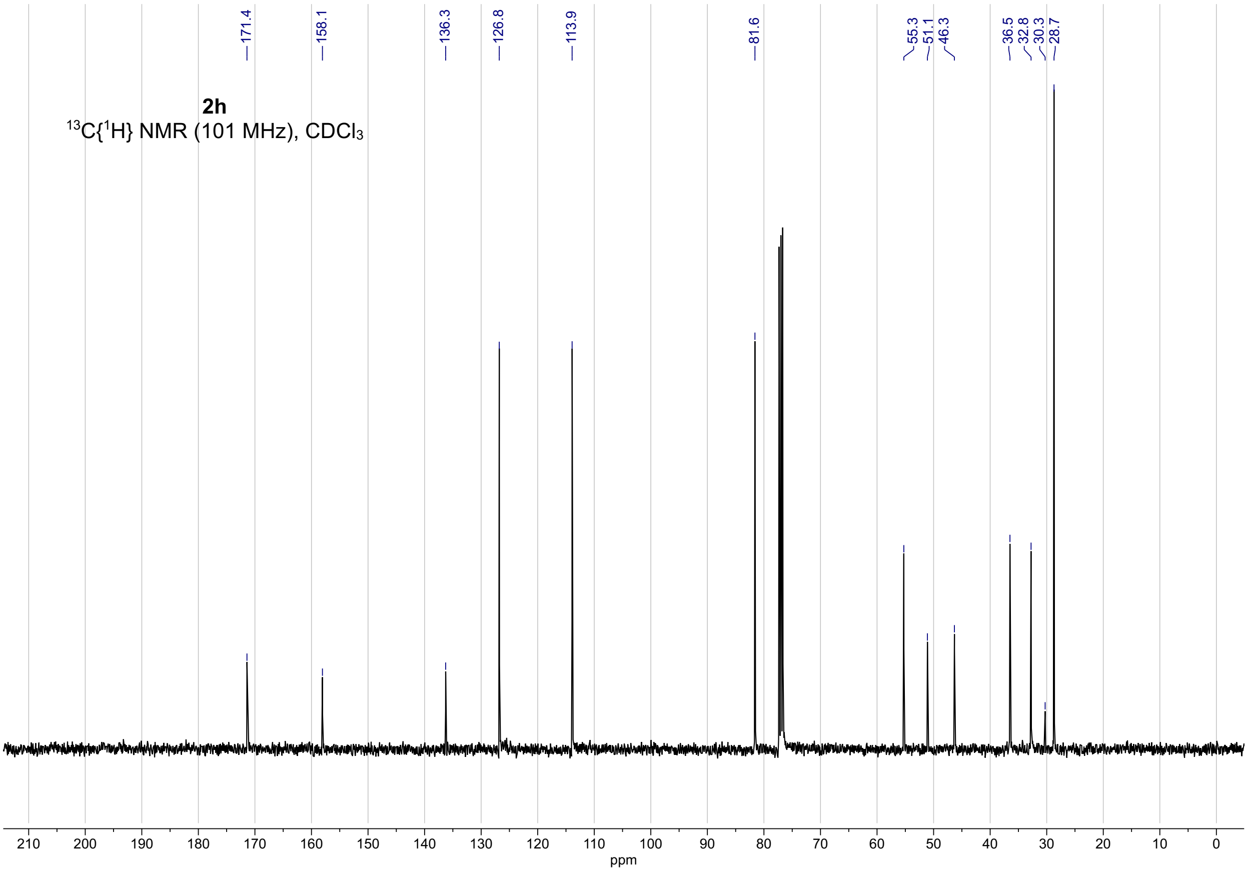


**2h**

$^{13}C\{^{1}H\}$ NMR (101 MHz), $CDCl_3$

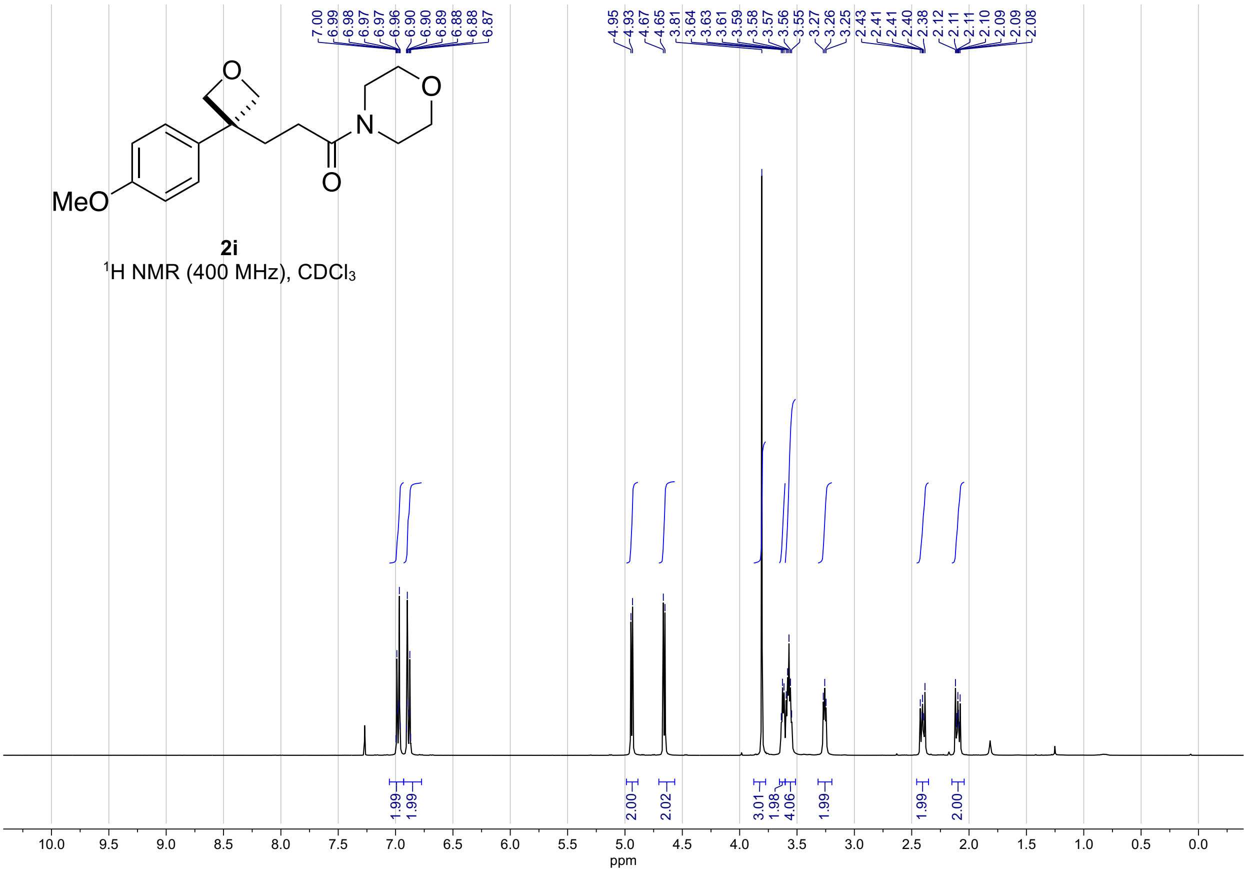

**2i**
$^{1}H$ NMR (400 MHz), $CDCl_3$

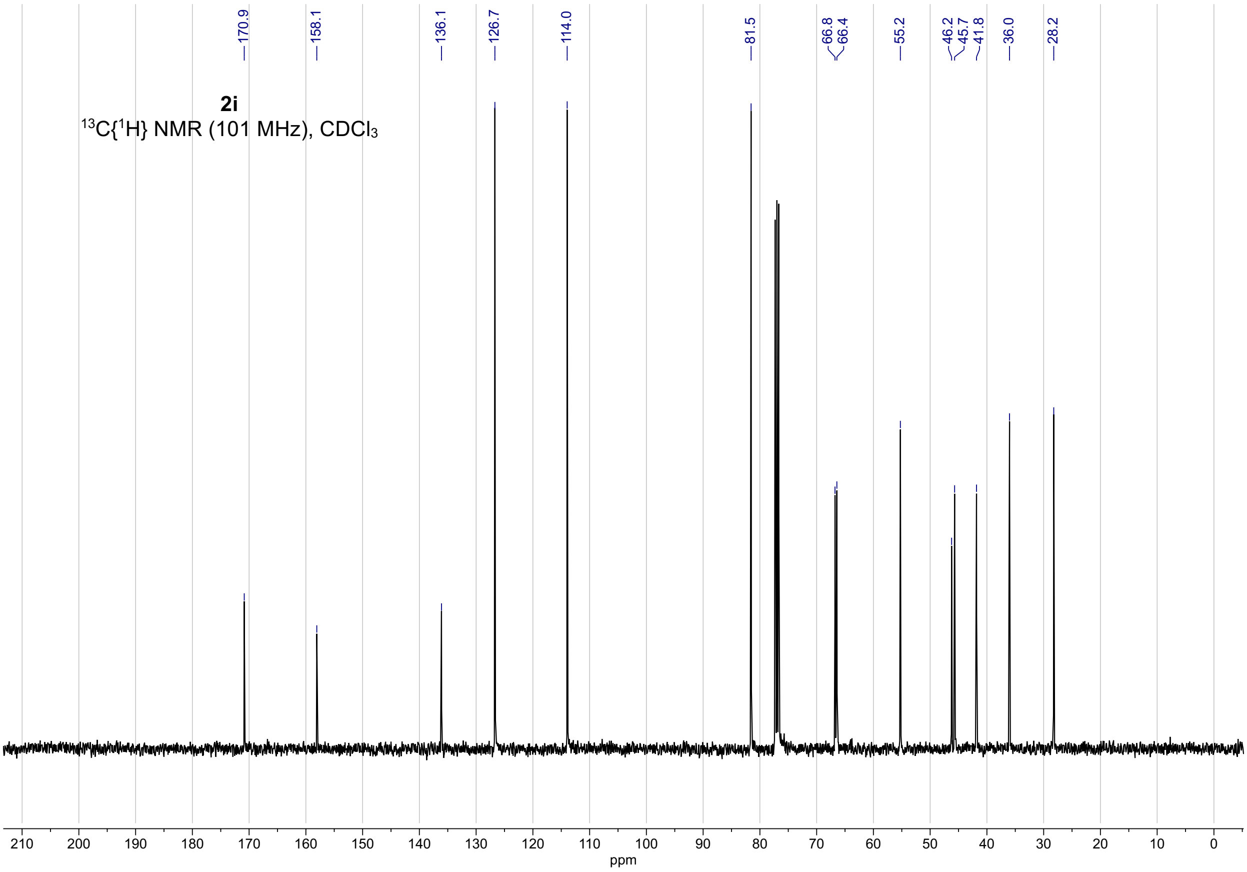

**2i**
$^{13}C\{^{1}H\}$ NMR (101 MHz), $CDCl_3$

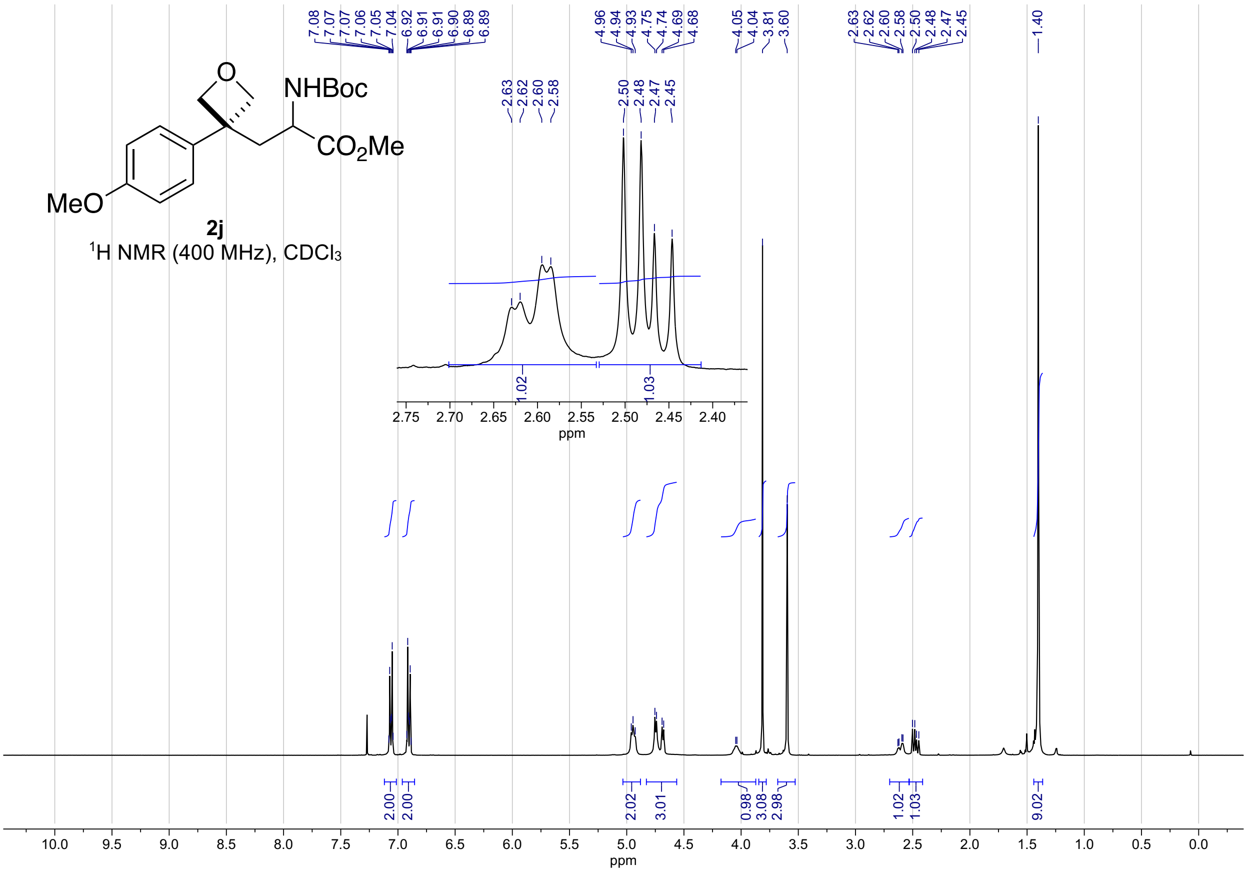
NHBoc
CO2Me
MeO
2j
1H NMR (400 MHz), CDCl3
7.08 7.07 7.07 7.06 7.05 7.04 6.92 6.91 6.91 6.90 6.89 6.89
4.96 4.94 4.93 4.75 4.74 4.69 4.68
4.05 4.04 3.81 3.60
2.63 2.62 2.60 2.58 2.50 2.48 2.47 2.45
1.40
2.00 2.00 2.02 3.01 0.98 3.08 2.98 1.02 1.03 9.02
ppm

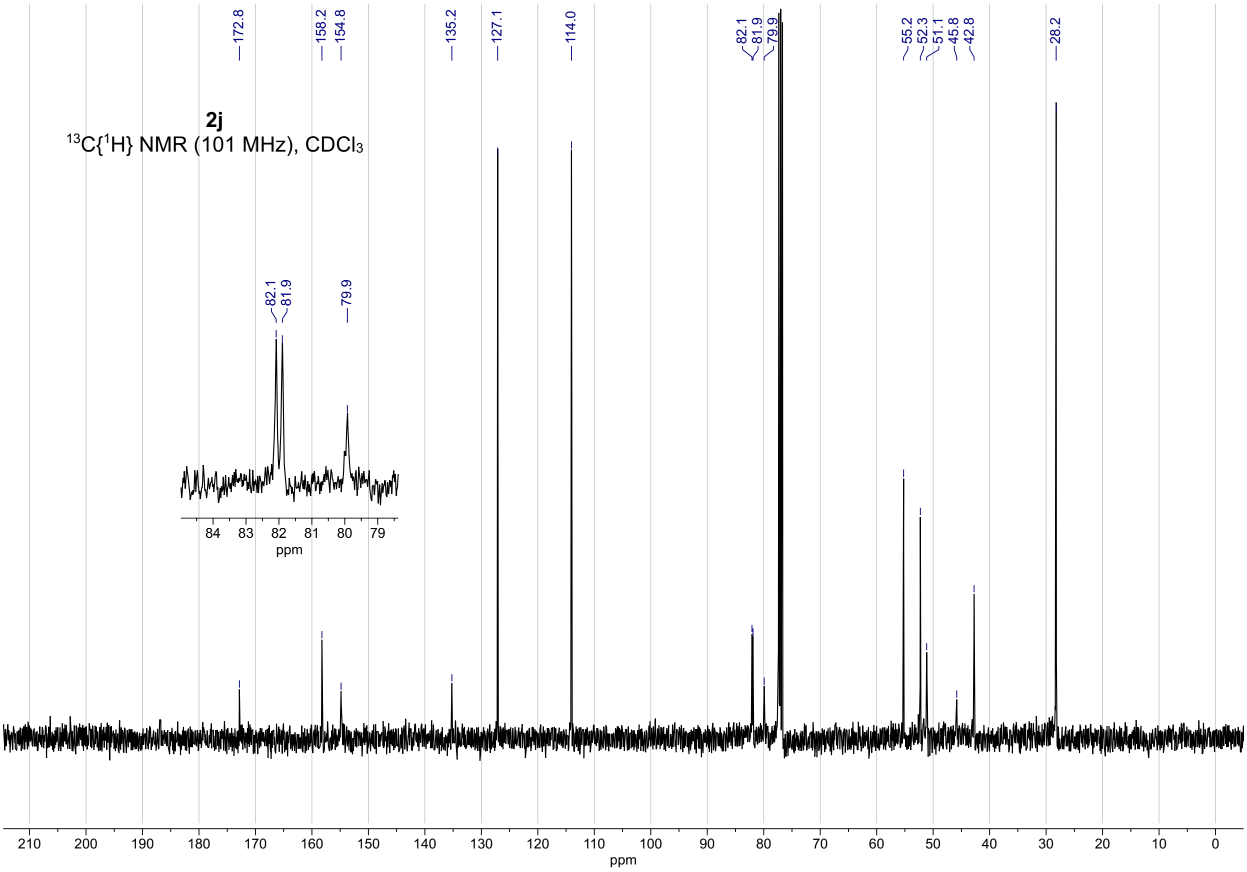
2j
13C{1H} NMR (101 MHz), CDCl3
172.8 158.2 154.8 135.2 127.1 114.0 82.1 81.9 79.9 55.2 52.3 51.1 45.8 42.8 28.2
ppm

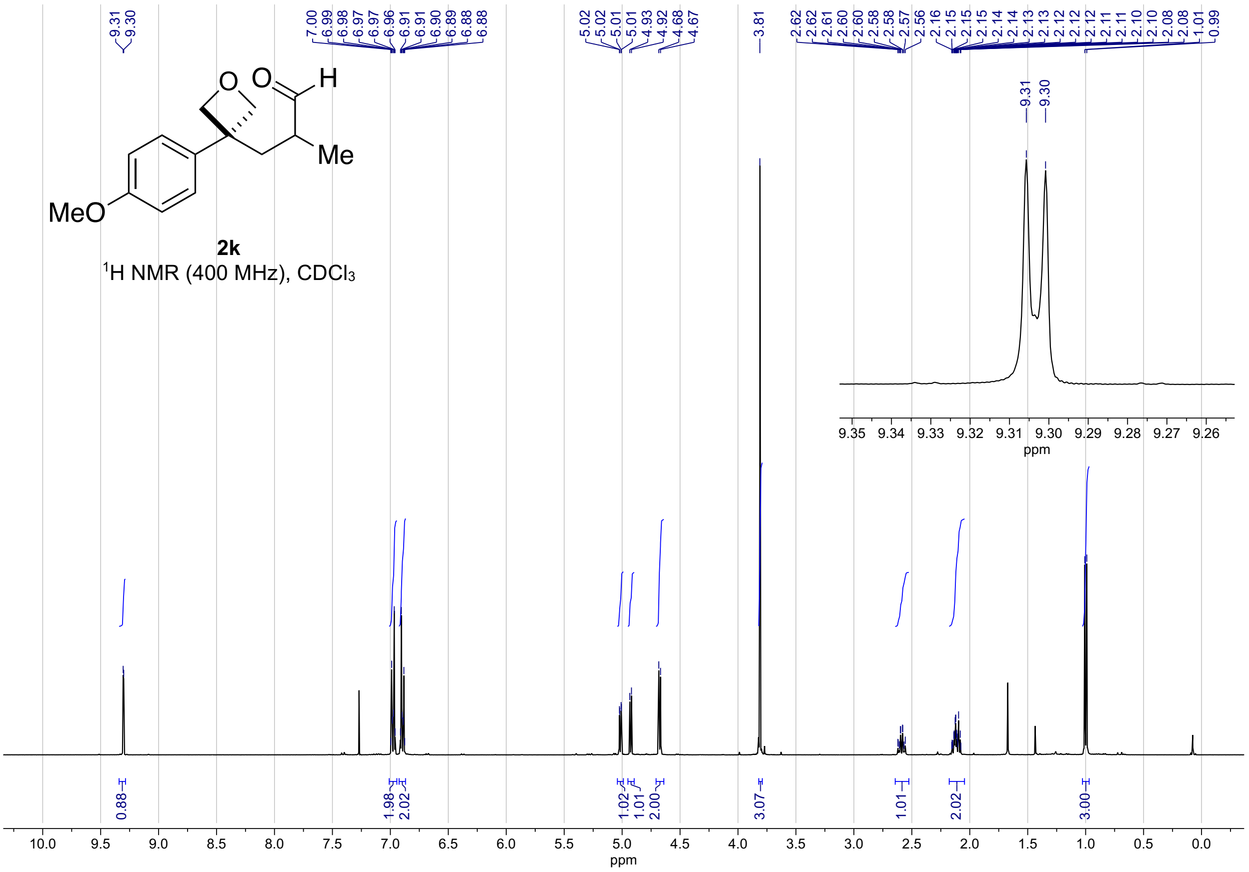
MeO
O
O
H
Me
2k
1H NMR (400 MHz), CDCl3

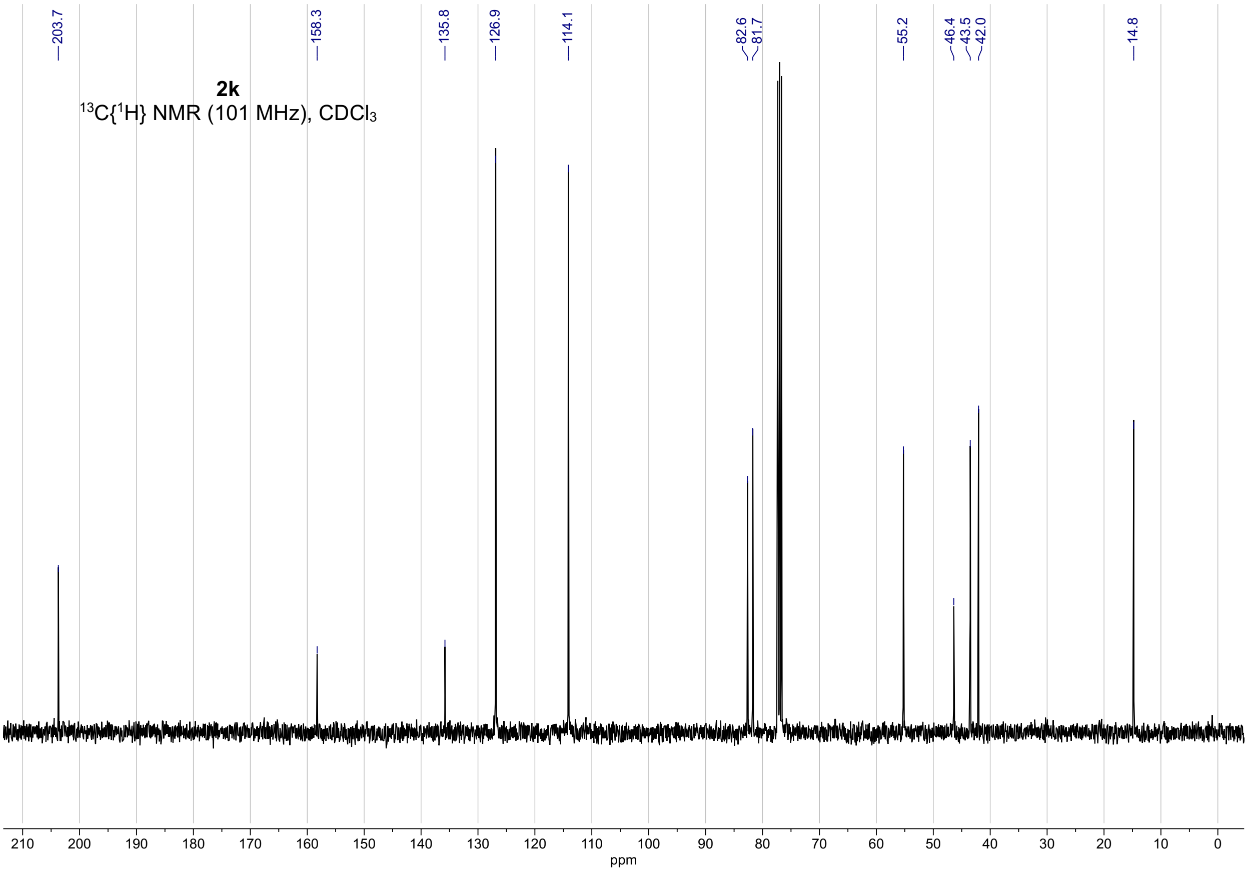
2k
13C{1H} NMR (101 MHz), CDCl3

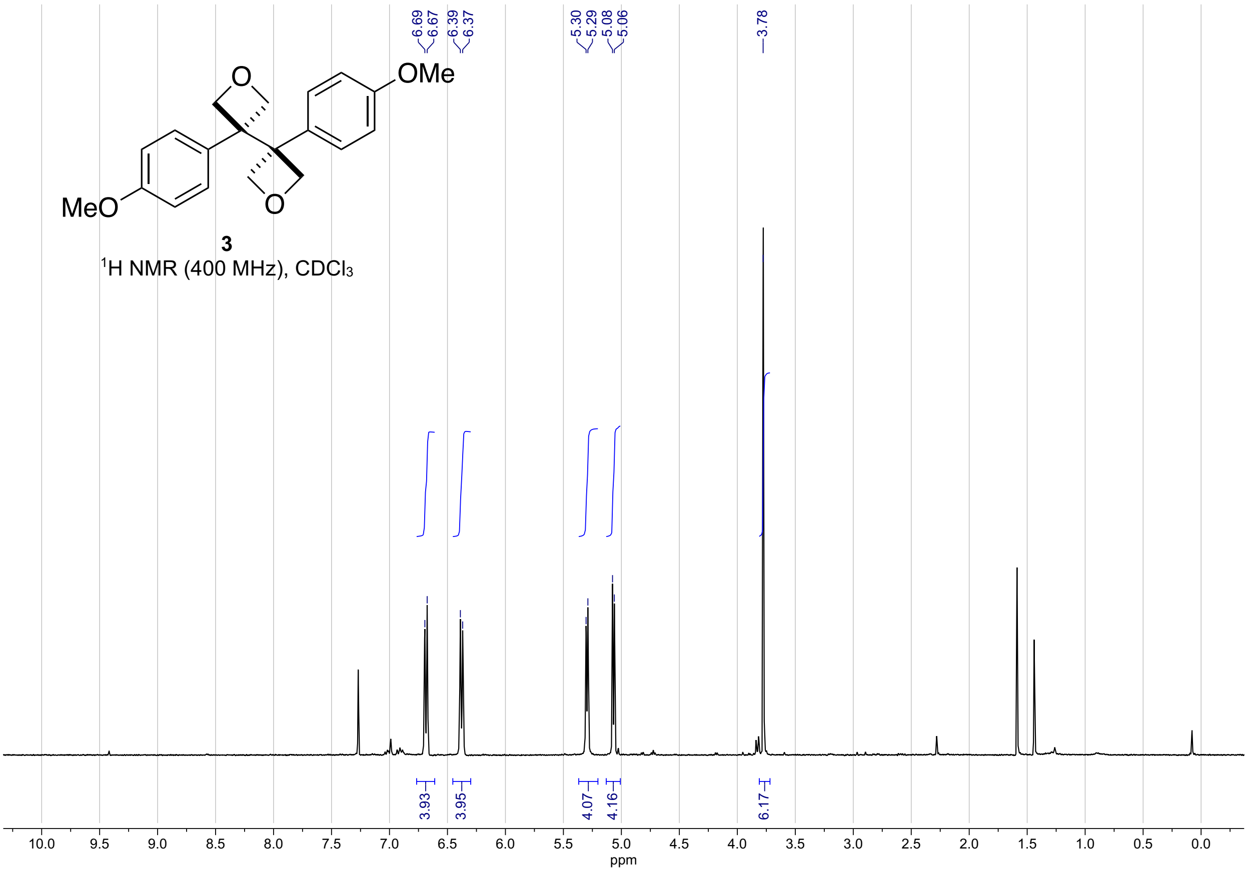
6.69
6.67
6.39
6.37
5.30
5.29
5.08
5.06
3.78
OMe
MeO
O
O
3
1H NMR (400 MHz), CDCl3
3.93
3.95
4.07
4.16
6.17
10.0 9.5 9.0 8.5 8.0 7.5 7.0 6.5 6.0 5.5 5.0 4.5 4.0 3.5 3.0 2.5 2.0 1.5 1.0 0.5 0.0
ppm

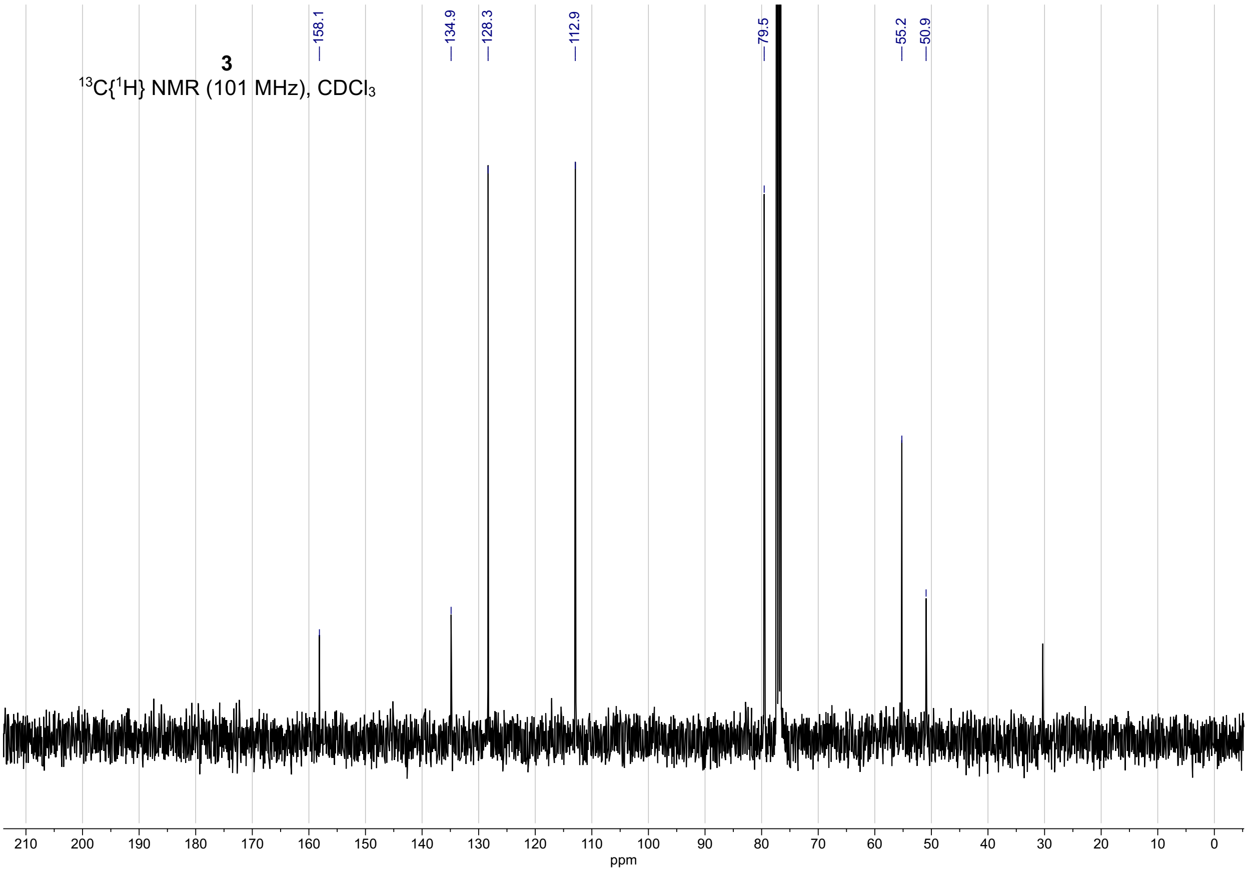
158.1
134.9
128.3
112.9
79.5
55.2
50.9
3
13C{1H} NMR (101 MHz), CDCl3
210 200 190 180 170 160 150 140 130 120 110 100 90 80 70 60 50 40 30 20 10 0
ppm

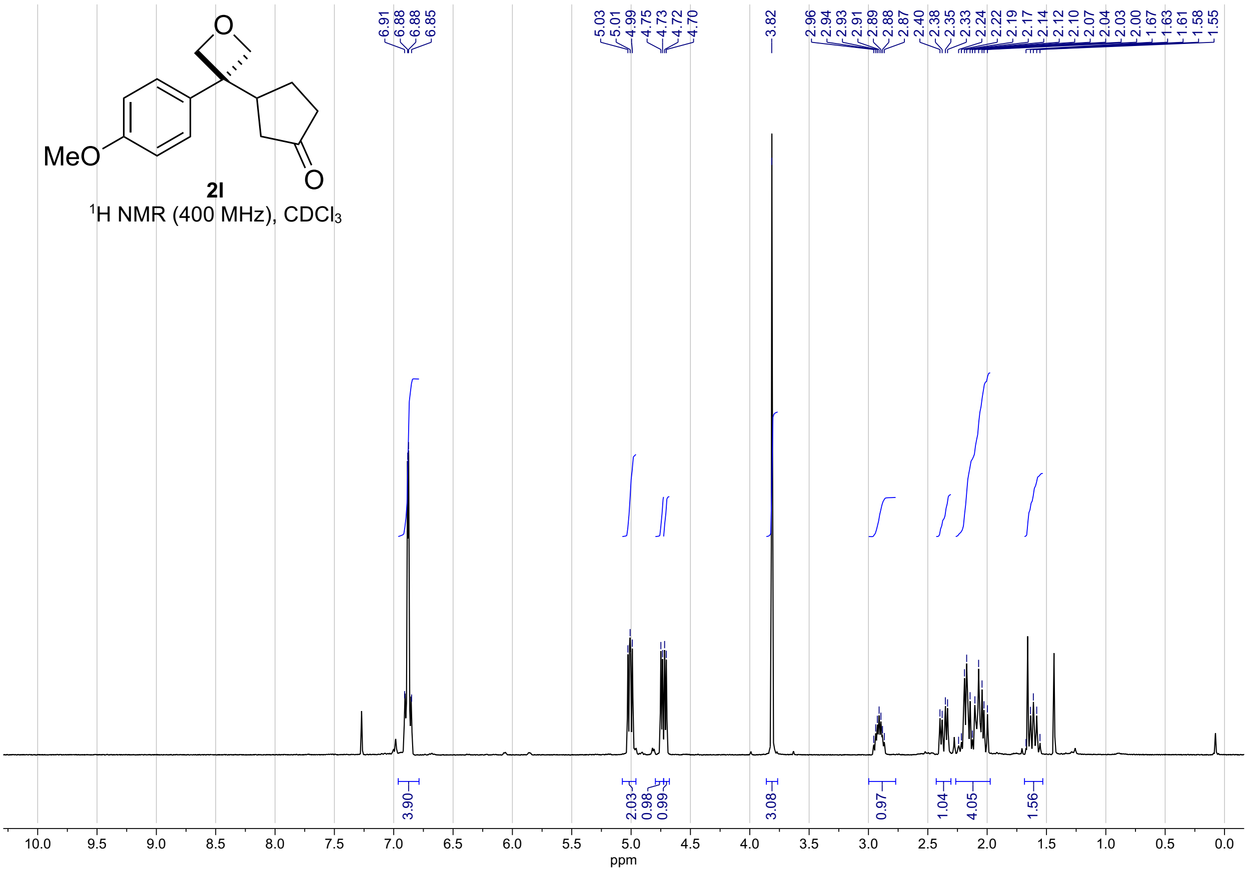
MeO
O
O
2l
$^1$H NMR (400 MHz), CDCl$_3$
ppm

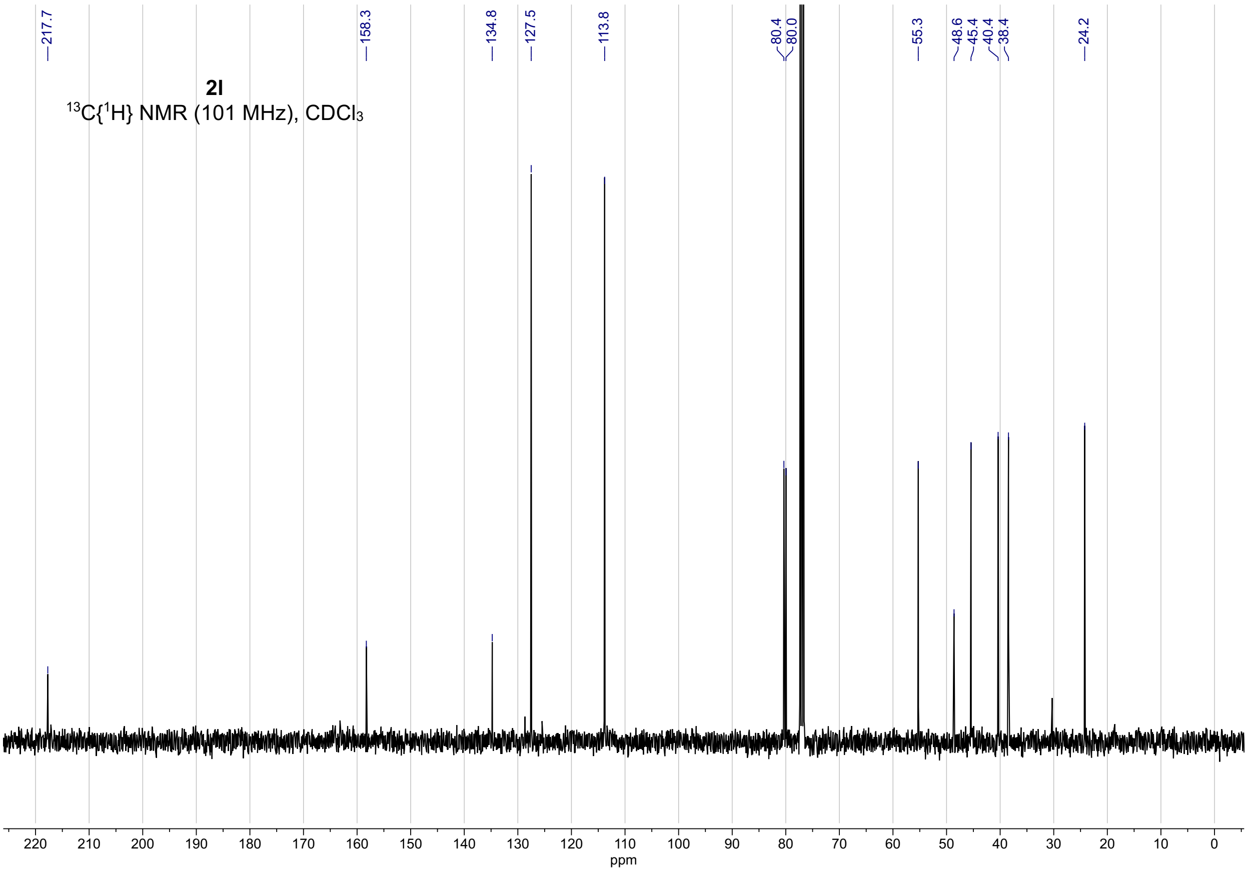
217.7
158.3
134.8
127.5
113.8
80.4
80.0
55.3
48.6
45.4
40.4
38.4
24.2
2l
$^{13}$C{$^1$H} NMR (101 MHz), CDCl$_3$
ppm

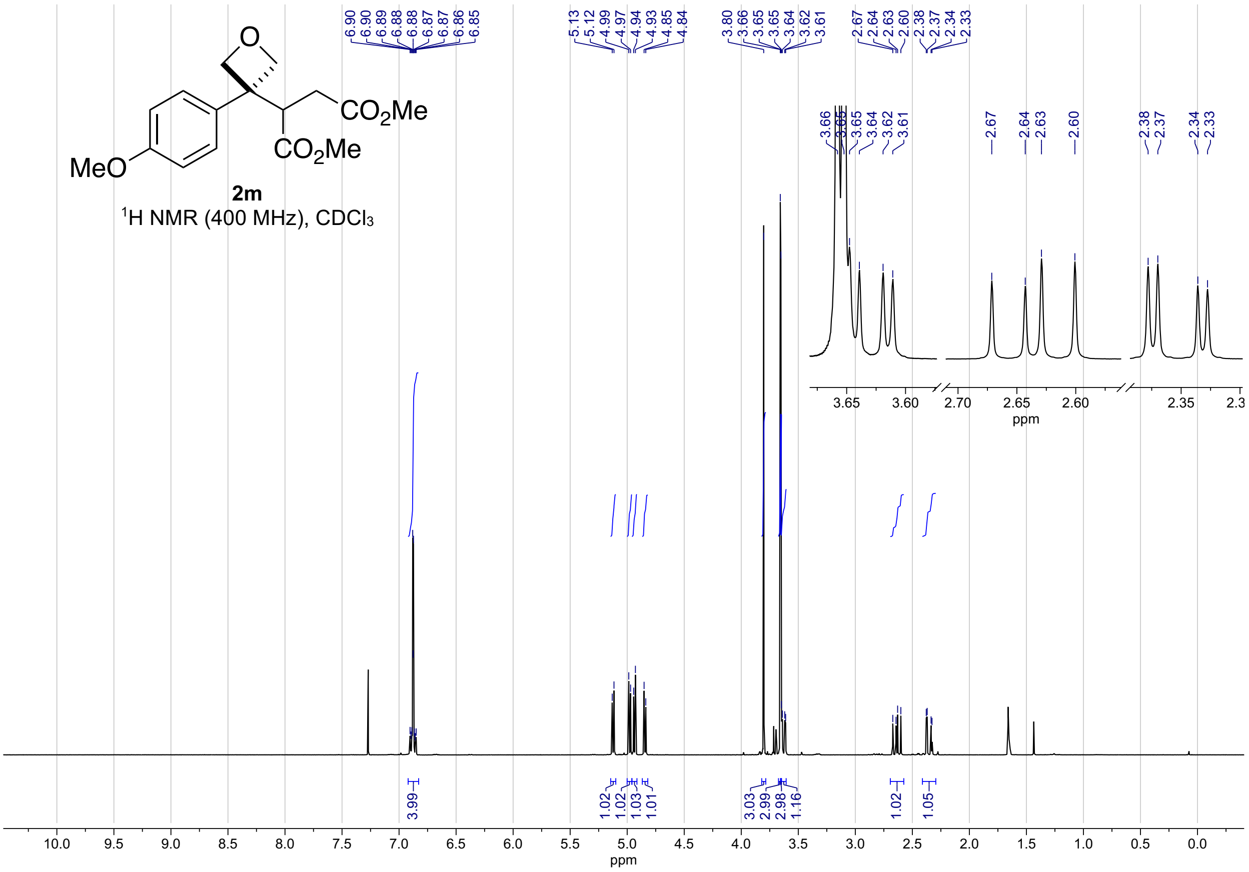
2m
$^1$H NMR (400 MHz), CDCl$_3$
ppm

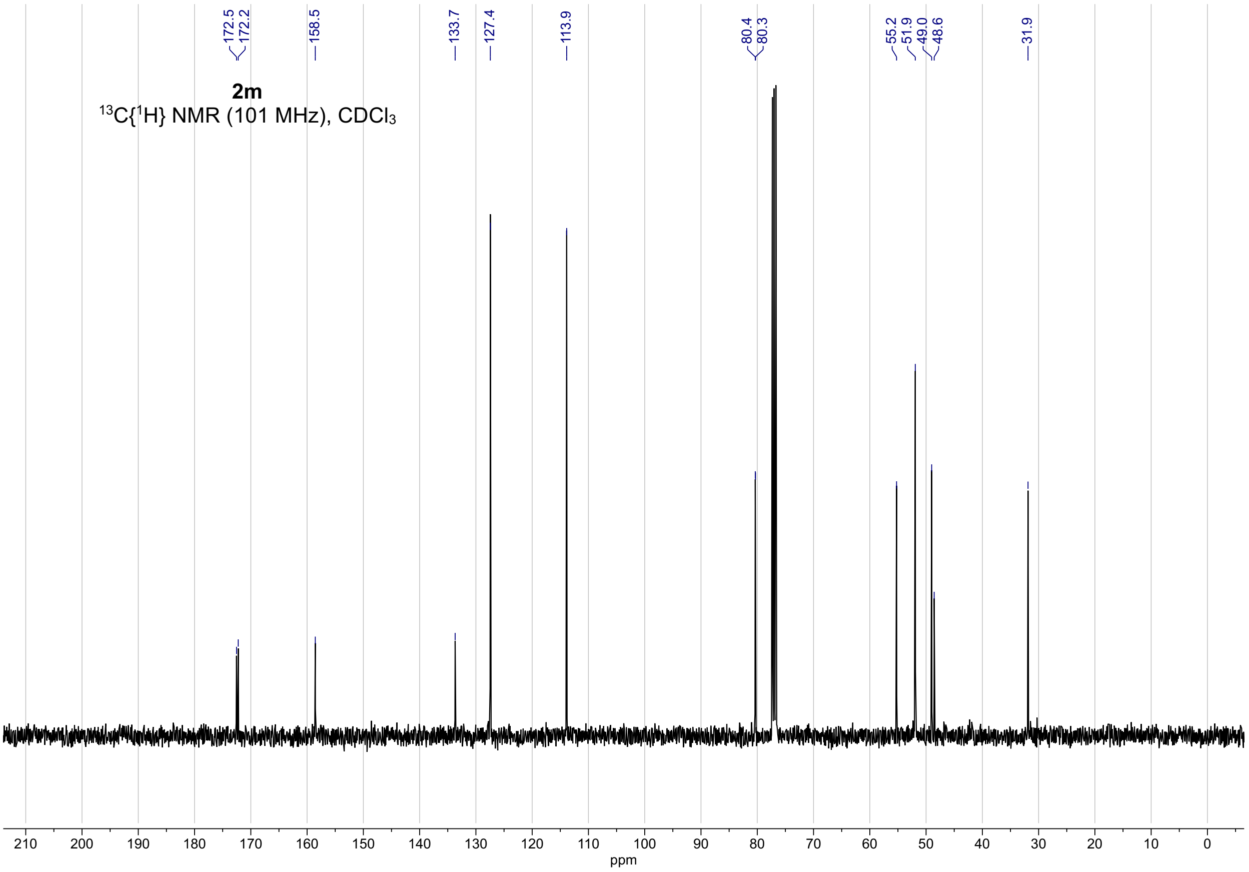
2m
$^{13}$C{$^1$H} NMR (101 MHz), CDCl$_3$
172.5
172.2
158.5
133.7
127.4
113.9
80.4
80.3
55.2
51.9
49.0
48.6
31.9
ppm

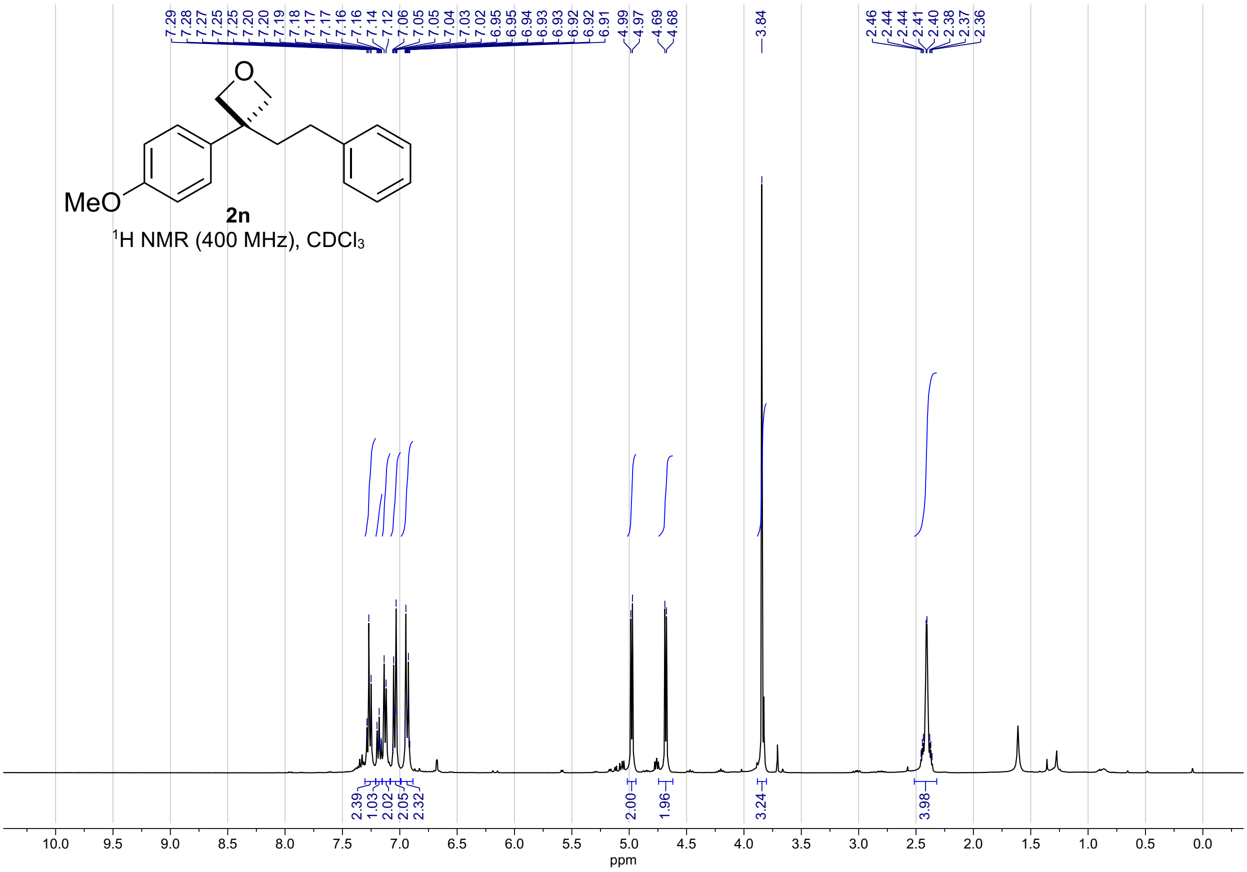

MeO
2n
[1]H NMR (400 MHz), CDCl3
ppm


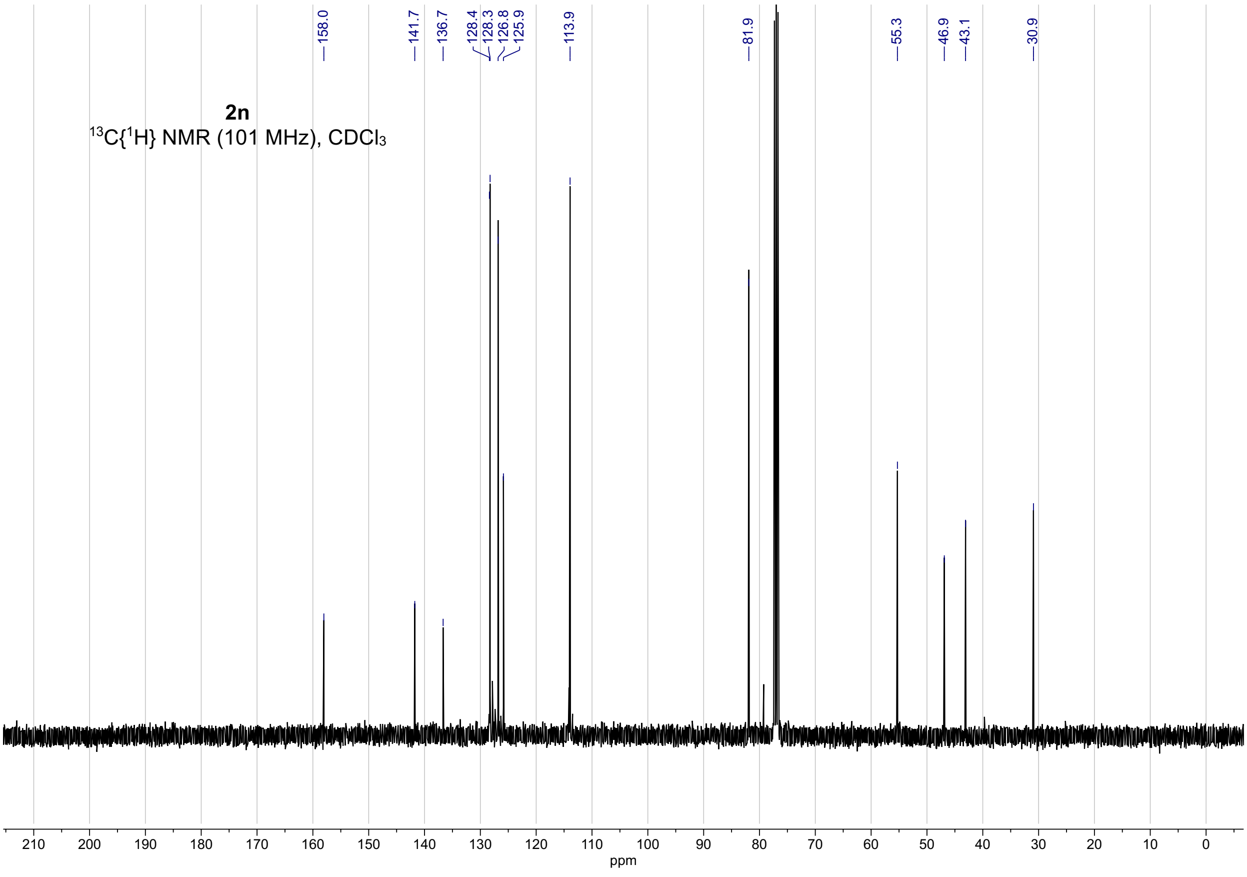

2n
[13]C{[1]H} NMR (101 MHz), CDCl3
158.0
141.7
136.7
128.4
128.3
126.8
125.9
113.9
81.9
55.3
46.9
43.1
30.9
ppm

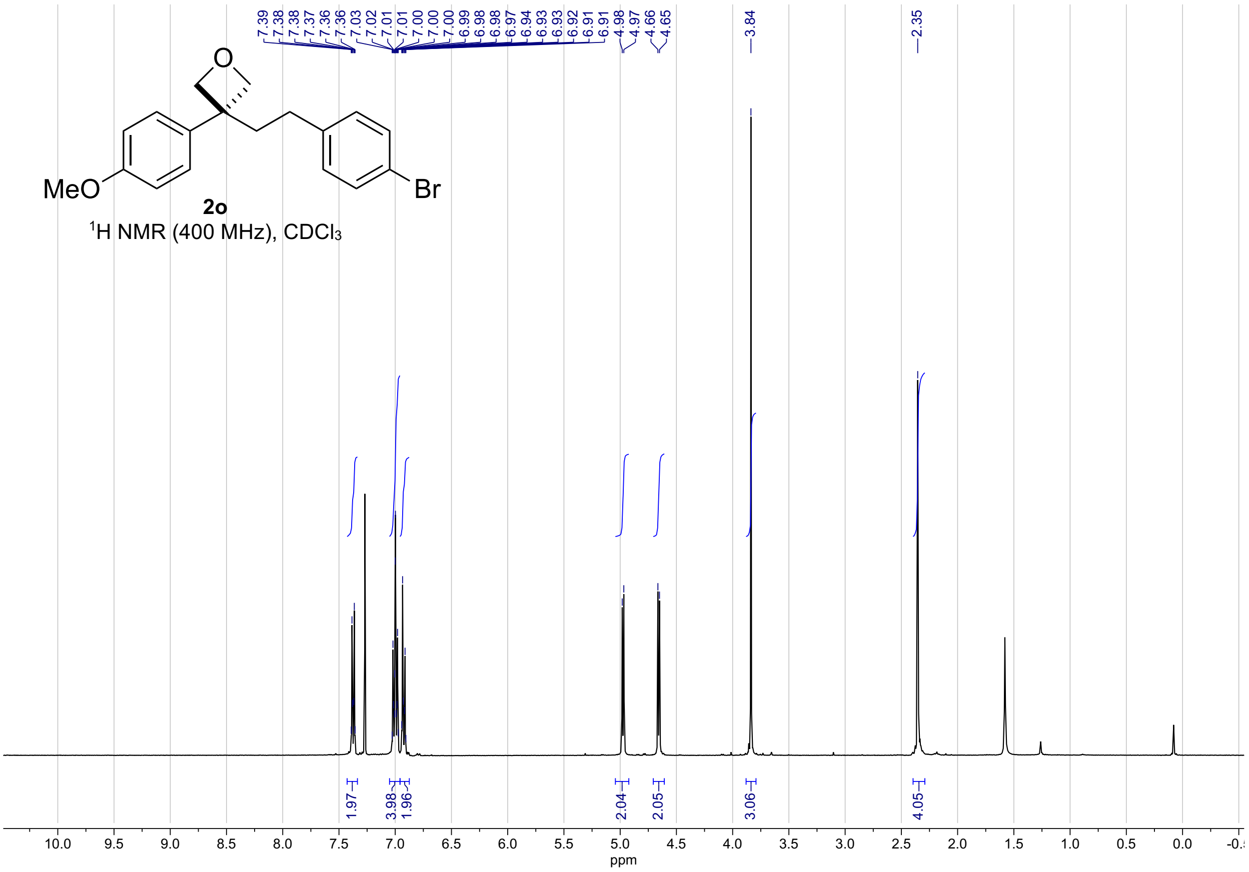

MeO
Br
2o
1H NMR (400 MHz), CDCl3


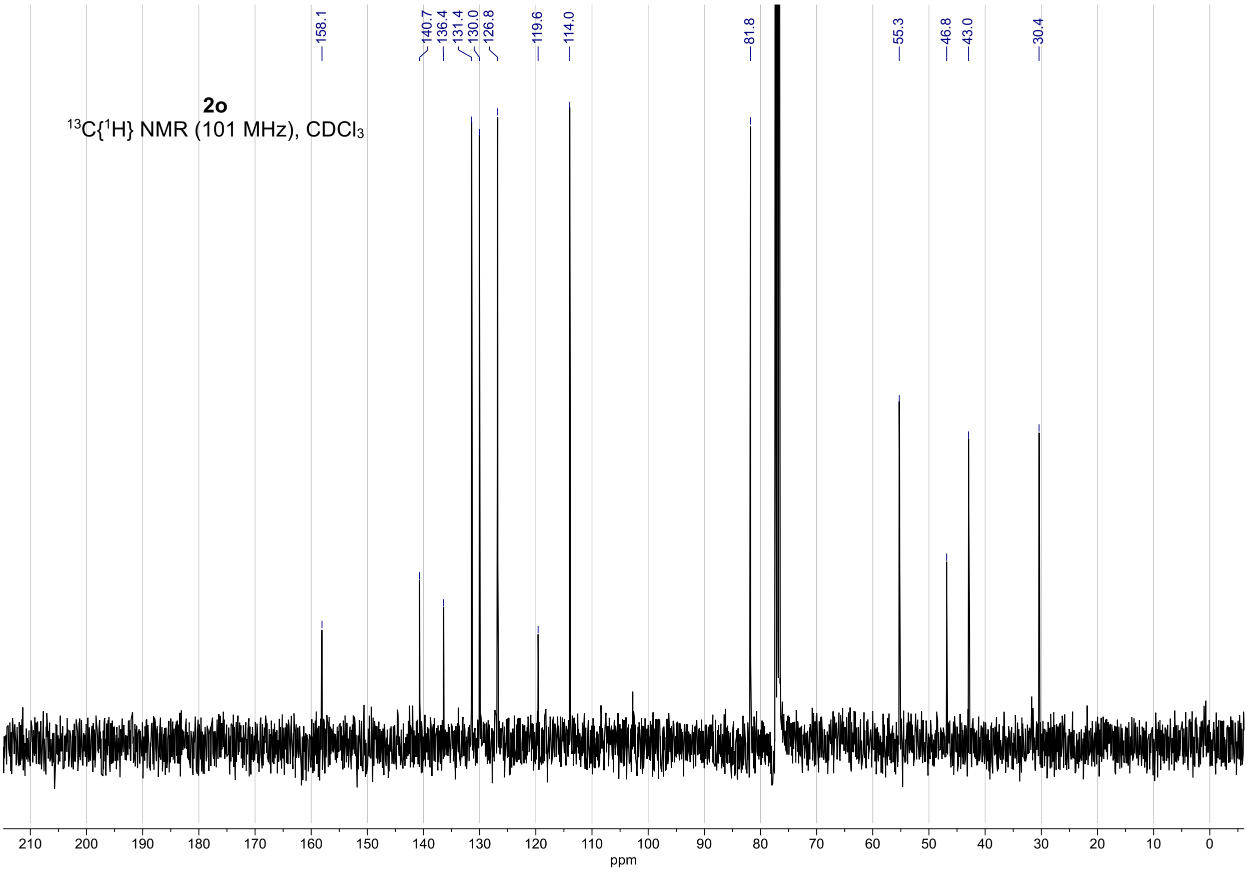

2o
13C{1H} NMR (101 MHz), CDCl3

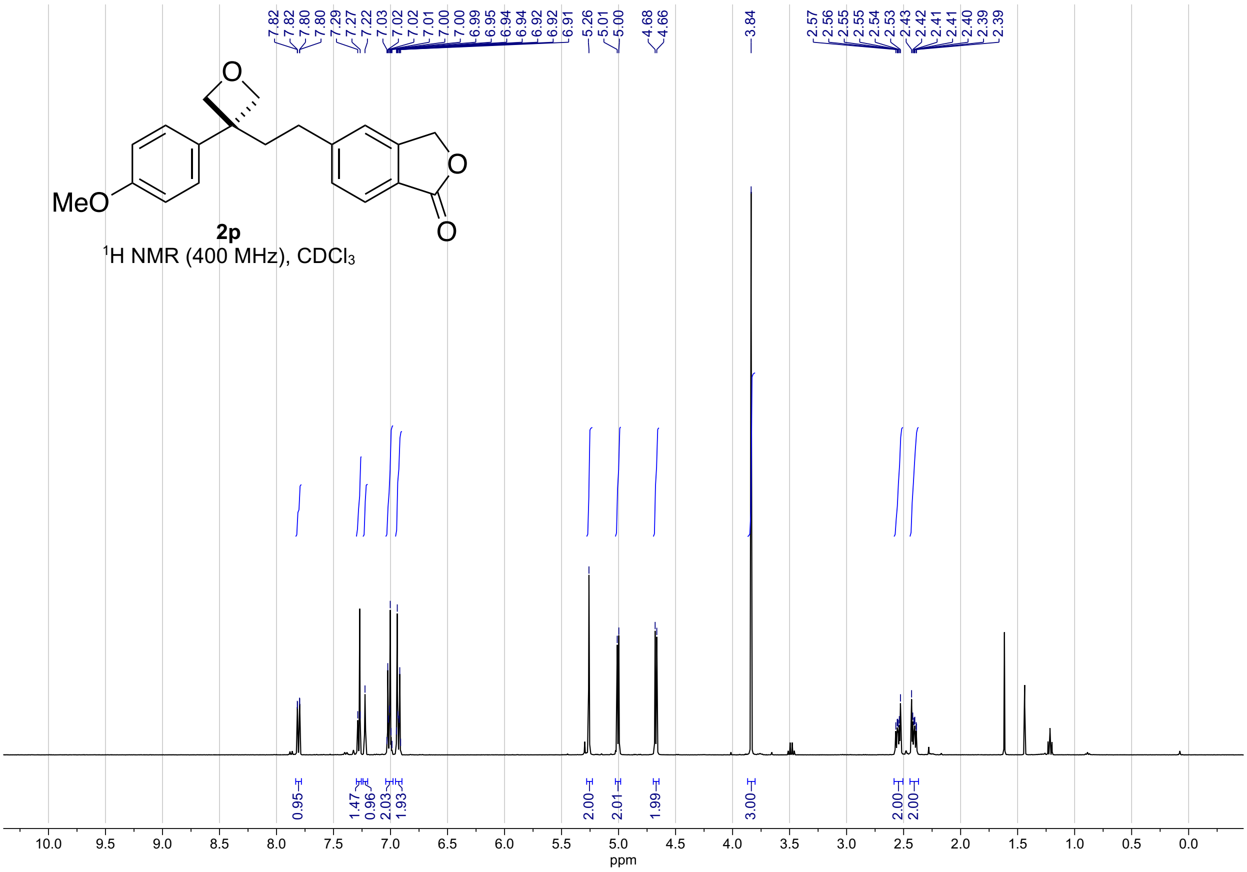
MeO
O
O
O
2p
[1]H NMR (400 MHz), CDCl3
7.82 7.82 7.80 7.80 7.29 7.27 7.22 7.03 7.02 7.02 7.01 7.00 7.00 6.99 6.95 6.94 6.94 6.92 6.92 6.91
5.26 5.01 5.00 4.68 4.66
3.84
2.57 2.56 2.55 2.55 2.54 2.53 2.43 2.42 2.41 2.41 2.40 2.39 2.39
0.95 1.47 0.96 2.03 1.93 2.00 2.01 1.99 3.00 2.00 2.00
ppm

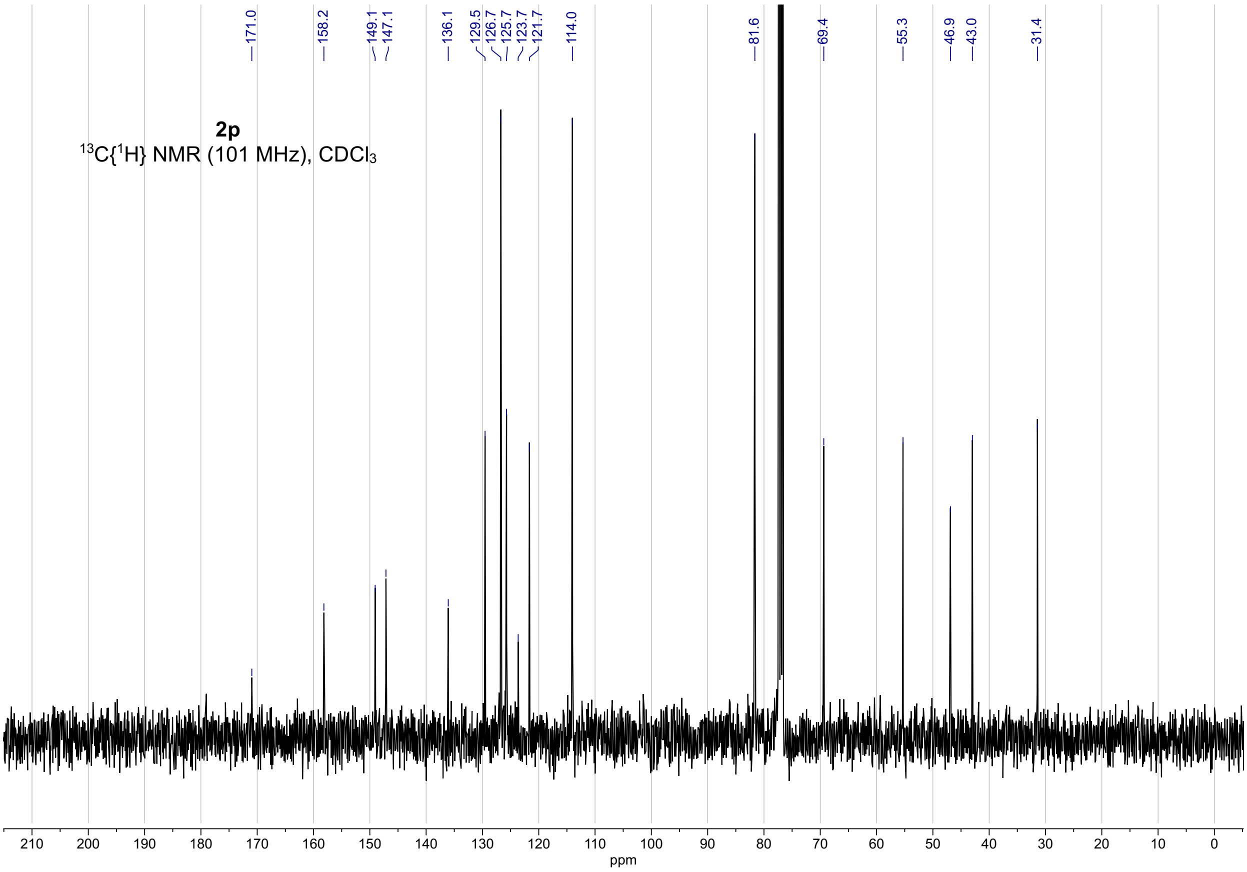
2p
[13]C{[1]H} NMR (101 MHz), CDCl3
171.0 158.2 149.1 147.1 136.1 129.5 126.7 125.7 123.7 121.7 114.0 81.6 69.4 55.3 46.9 43.0 31.4
ppm

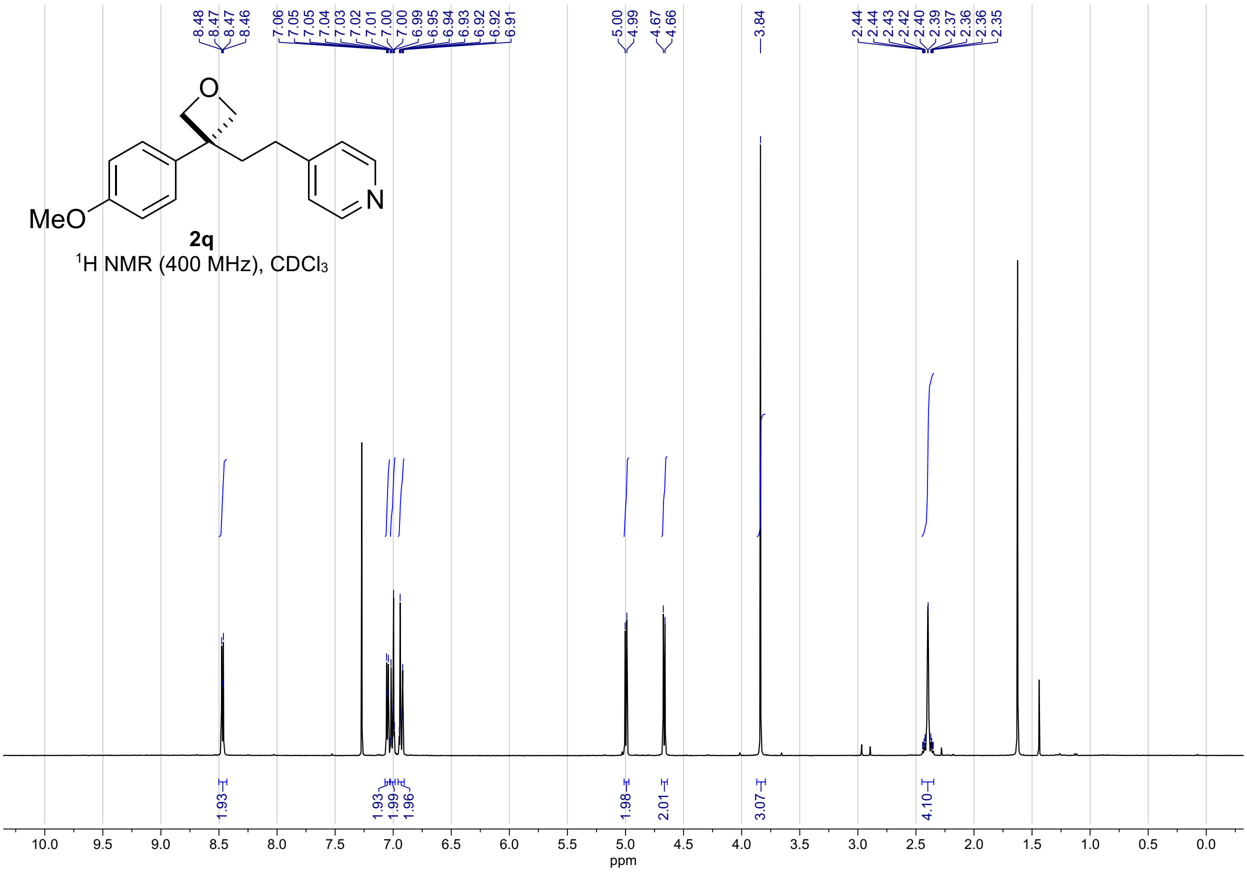
8.48
8.47
8.47
8.46
7.06
7.05
7.05
7.04
7.03
7.02
7.01
7.00
7.00
6.99
6.95
6.94
6.93
6.92
6.92
6.91
5.00
4.99
4.67
4.66
3.84
2.44
2.44
2.43
2.42
2.40
2.39
2.37
2.36
2.36
2.35
MeO
O
N
2q
¹H NMR (400 MHz), CDCl₃
1.93
1.93
1.99
1.96
1.98
2.01
3.07
4.10
10.0 9.5 9.0 8.5 8.0 7.5 7.0 6.5 6.0 5.5 5.0 4.5 4.0 3.5 3.0 2.5 2.0 1.5 1.0 0.5 0.0
ppm

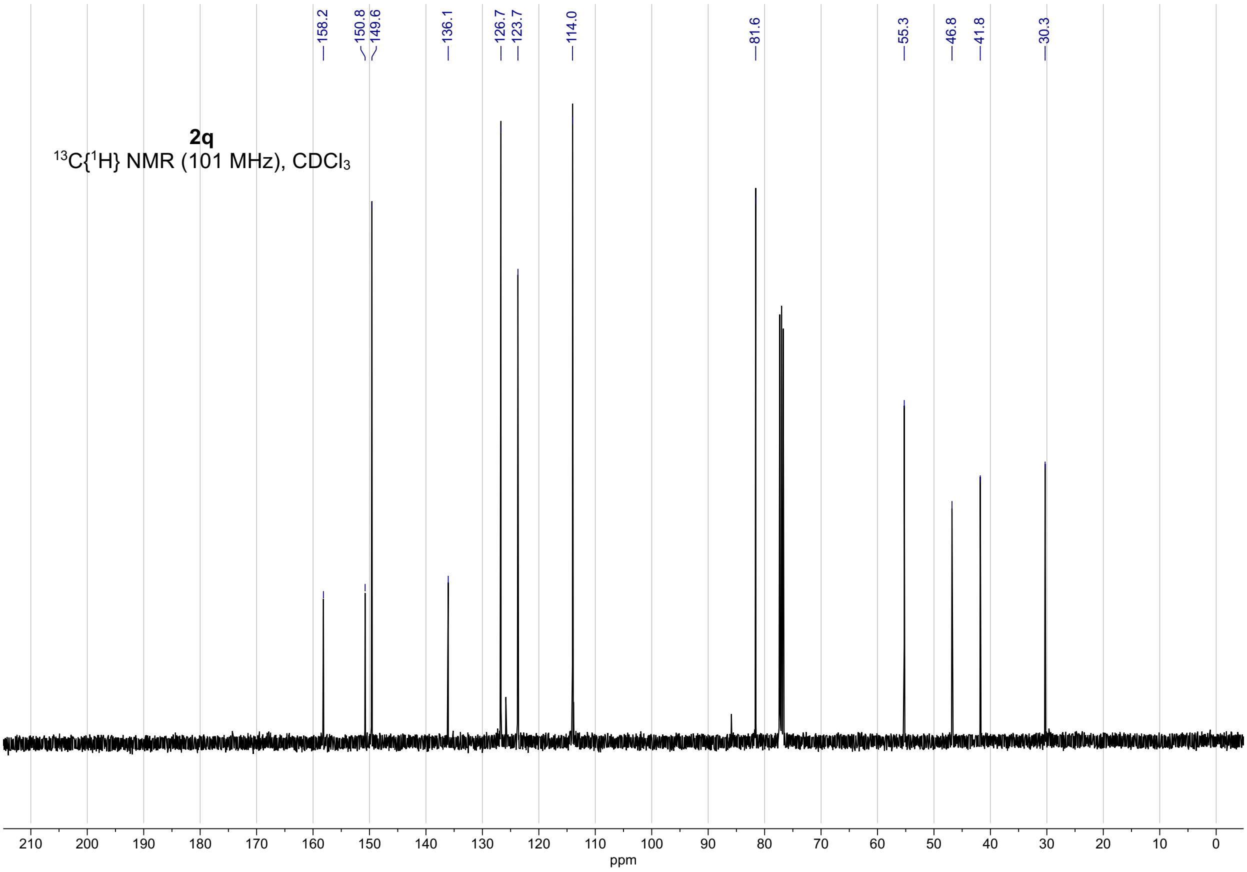
158.2
150.8
149.6
136.1
126.7
123.7
114.0
81.6
55.3
46.8
41.8
30.3
2q
¹³C{¹H} NMR (101 MHz), CDCl₃
210 200 190 180 170 160 150 140 130 120 110 100 90 80 70 60 50 40 30 20 10 0
ppm

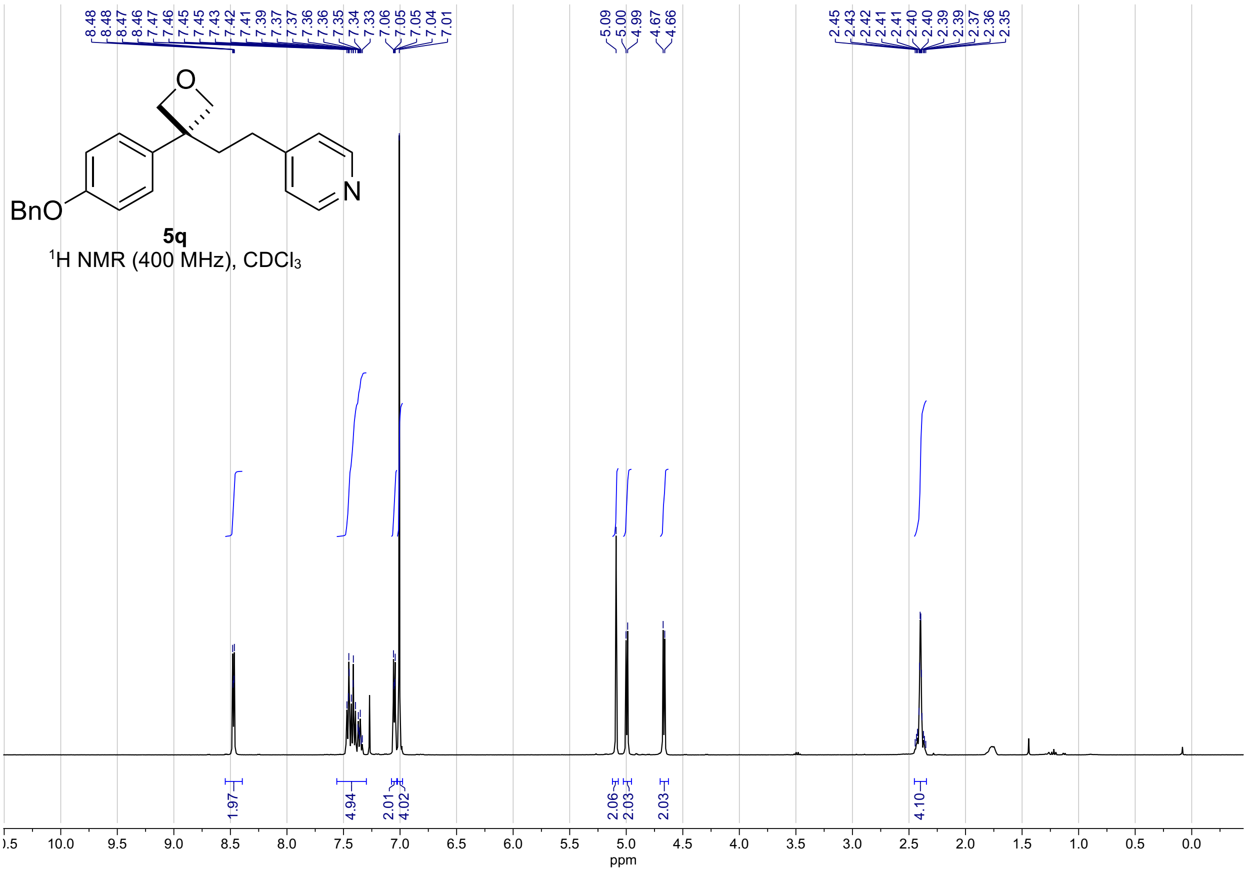
8.48
8.48
8.47
8.46
7.47
7.46
7.45
7.45
7.43
7.42
7.41
7.39
7.37
7.37
7.36
7.36
7.35
7.34
7.33
7.06
7.05
7.05
7.04
7.01
5.09
5.00
4.99
4.67
4.66
2.45
2.43
2.42
2.41
2.41
2.40
2.40
2.39
2.39
2.37
2.36
2.35
BnO
O
N
5q
¹H NMR (400 MHz), CDCl3
1.97
4.94
2.01
4.02
2.06
2.03
2.03
4.10
ppm

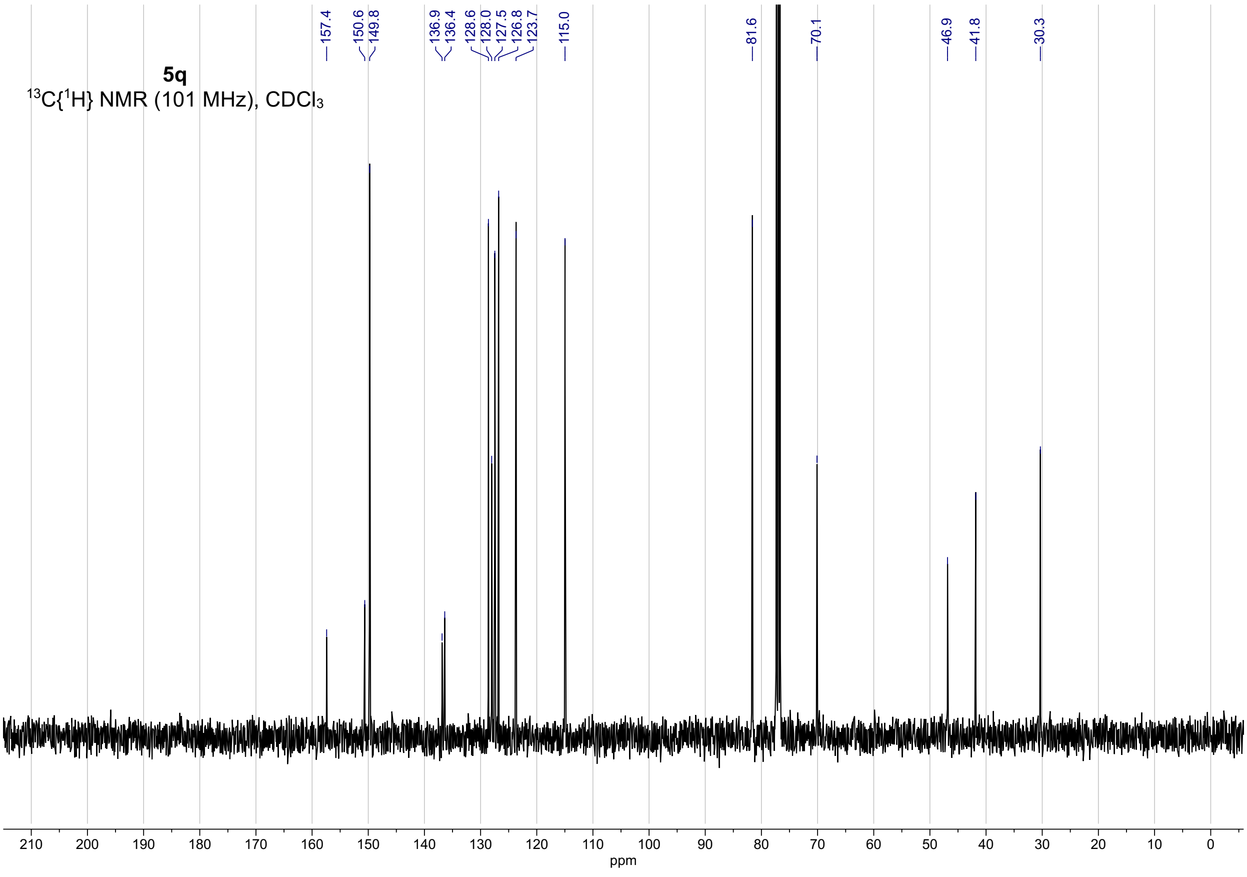
157.4
150.6
149.8
136.9
136.4
128.6
128.0
127.5
126.8
123.7
115.0
81.6
70.1
46.9
41.8
30.3
5q
¹³C{¹H} NMR (101 MHz), CDCl3
ppm

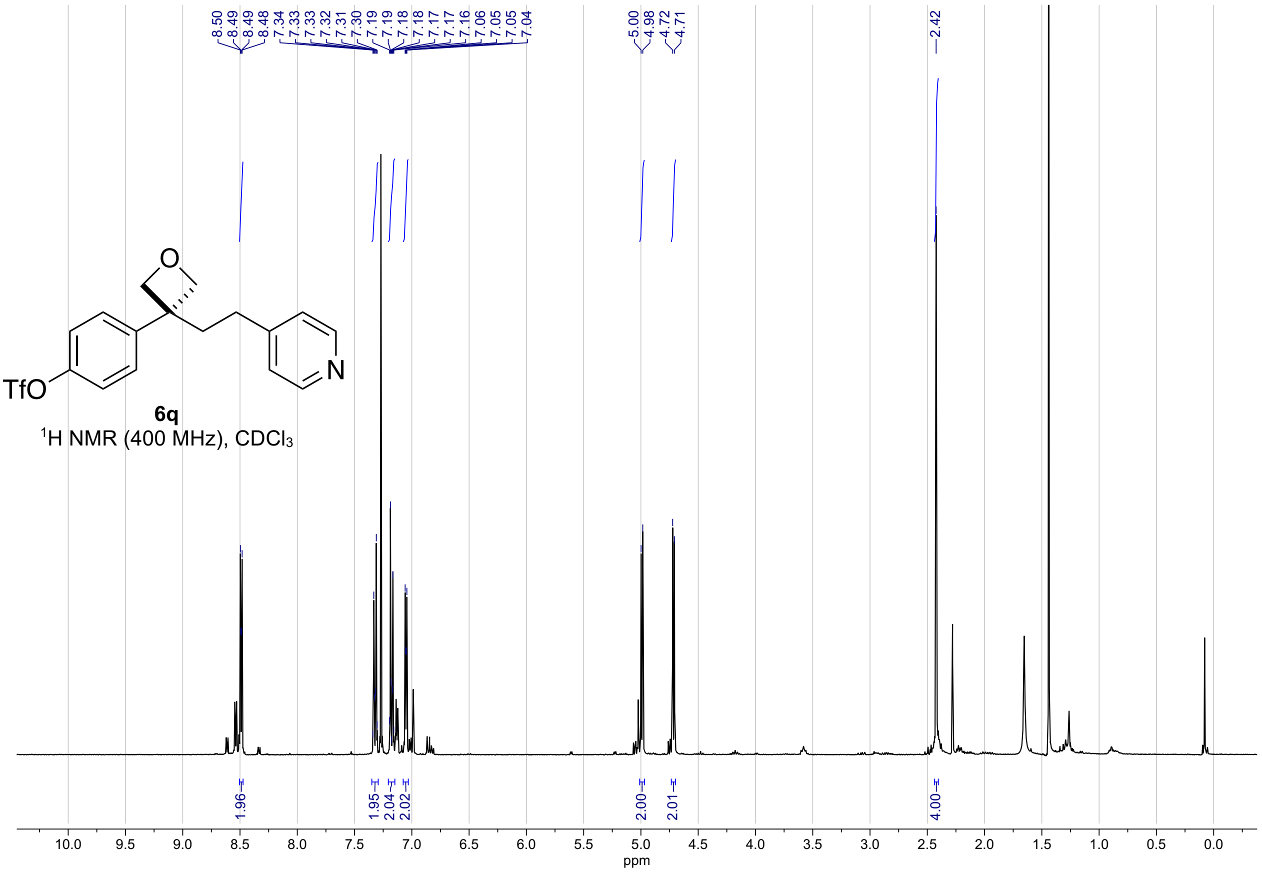
TfO
N
O
6q
1H NMR (400 MHz), CDCl3
8.50 8.49 8.49 8.48 7.34 7.33 7.33 7.32 7.31 7.30 7.19 7.19 7.18 7.18 7.17 7.17 7.16 7.06 7.05 7.05 7.04
5.00 4.98 4.72 4.71
2.42
1.96 1.95 2.04 2.02 2.00 2.01 4.00
ppm

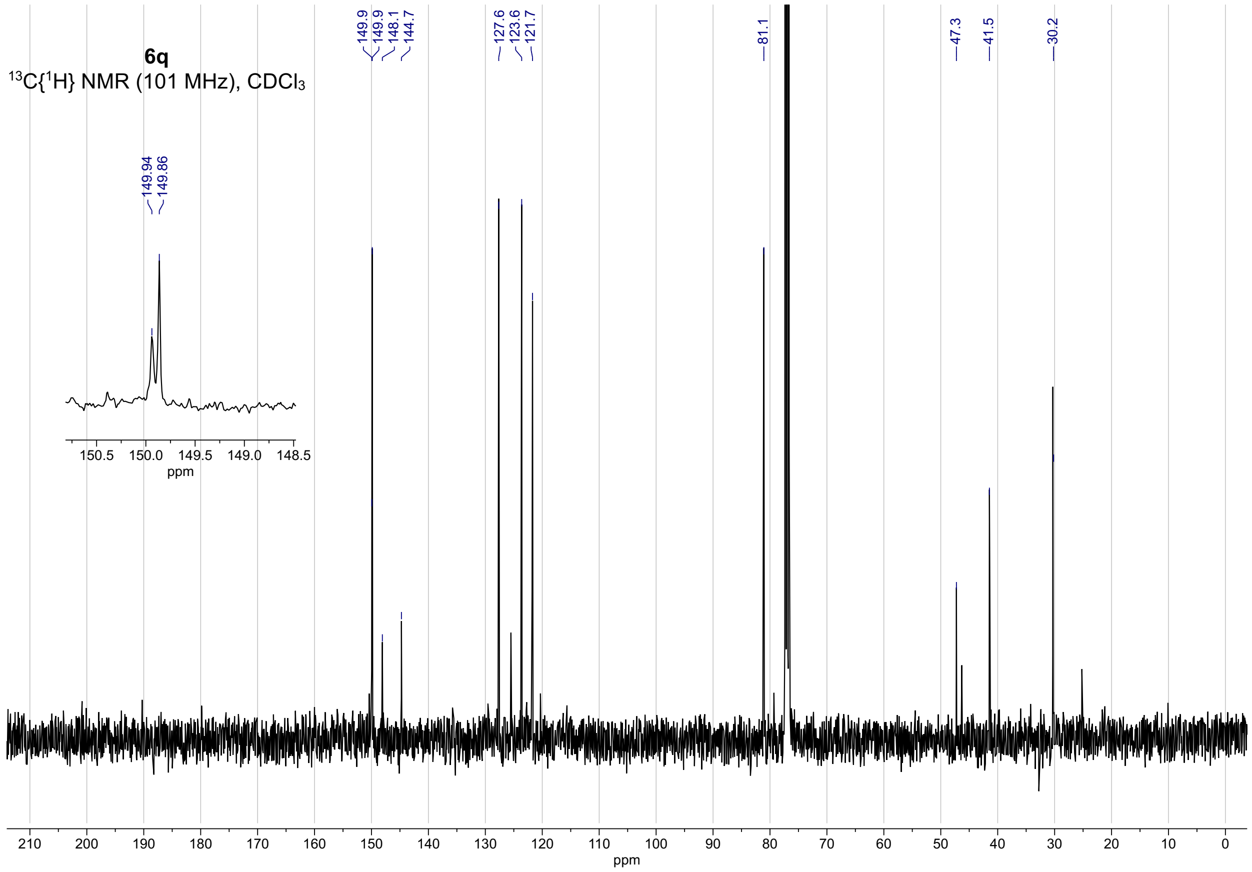
6q
13C{1H} NMR (101 MHz), CDCl3
149.94 149.86
149.9 149.9 148.1 144.7 127.6 123.6 121.7 81.1 47.3 41.5 30.2
ppm

**6q**

$^{19}F$ NMR (377 MHz), $CDCl_3$

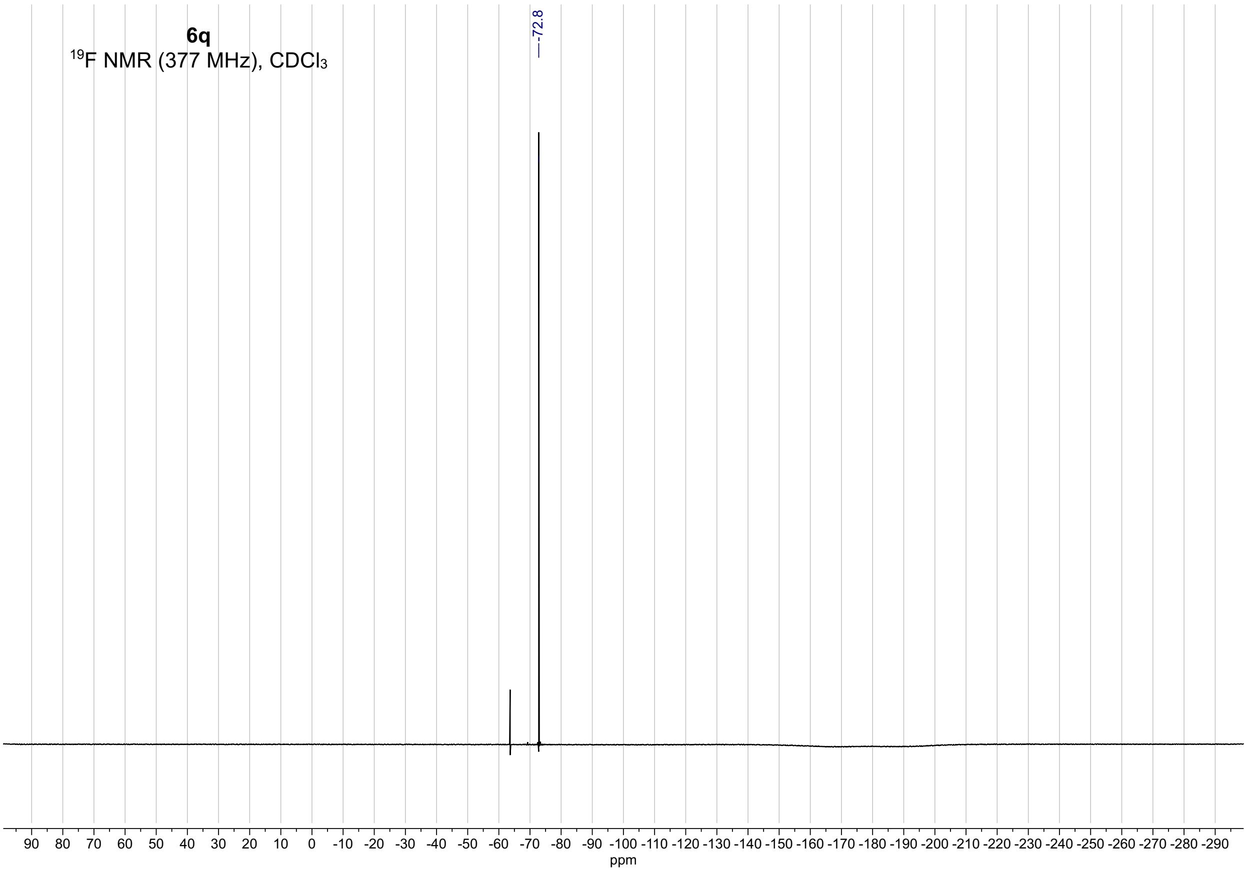

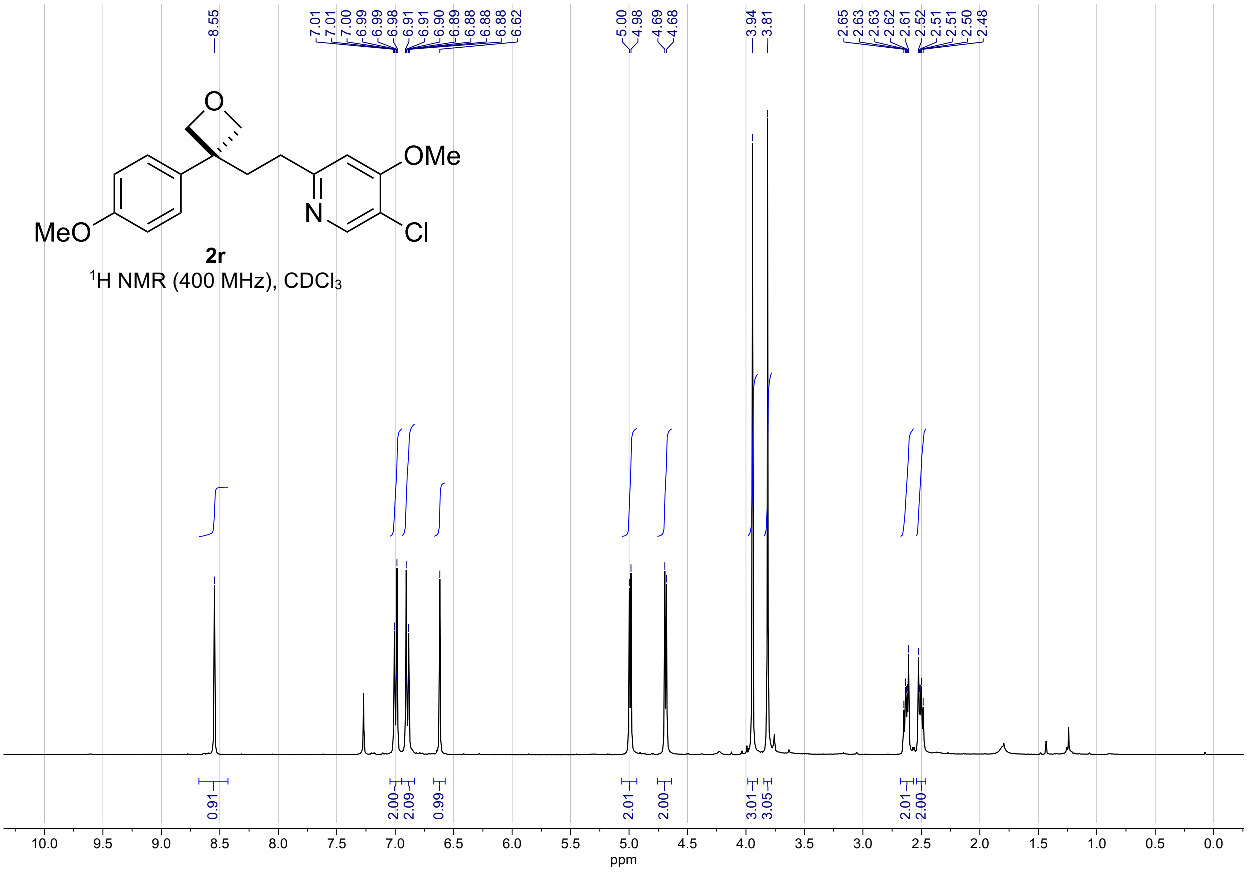
8.55
7.01 7.01 7.00 6.99 6.99 6.98 6.91 6.91 6.90 6.89 6.88 6.88 6.88 6.62
5.00 4.98 4.69 4.68
3.94 3.81
2.65 2.63 2.63 2.62 2.61 2.52 2.51 2.51 2.50 2.48
O
OMe
N
Cl
MeO
2r
¹H NMR (400 MHz), CDCl₃
0.91 2.00 2.09 0.99 2.01 2.00 3.01 3.05 2.01 2.00
10.0 9.5 9.0 8.5 8.0 7.5 7.0 6.5 6.0 5.5 5.0 4.5 4.0 3.5 3.0 2.5 2.0 1.5 1.0 0.5 0.0
ppm

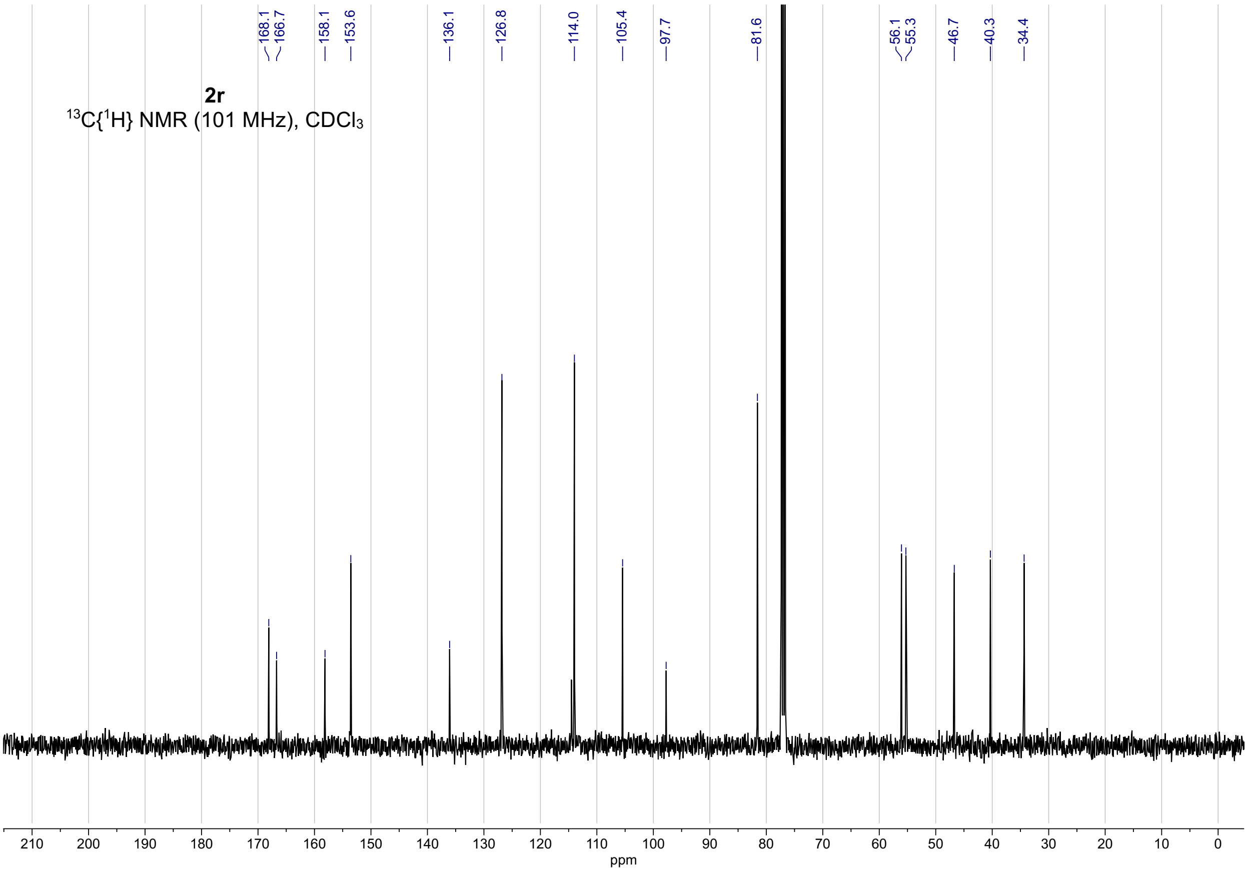
168.1 166.7 158.1 153.6 136.1 126.8 114.0 105.4 97.7 81.6 56.1 55.3 46.7 40.3 34.4
2r
¹³C{¹H} NMR (101 MHz), CDCl₃
210 200 190 180 170 160 150 140 130 120 110 100 90 80 70 60 50 40 30 20 10 0
ppm

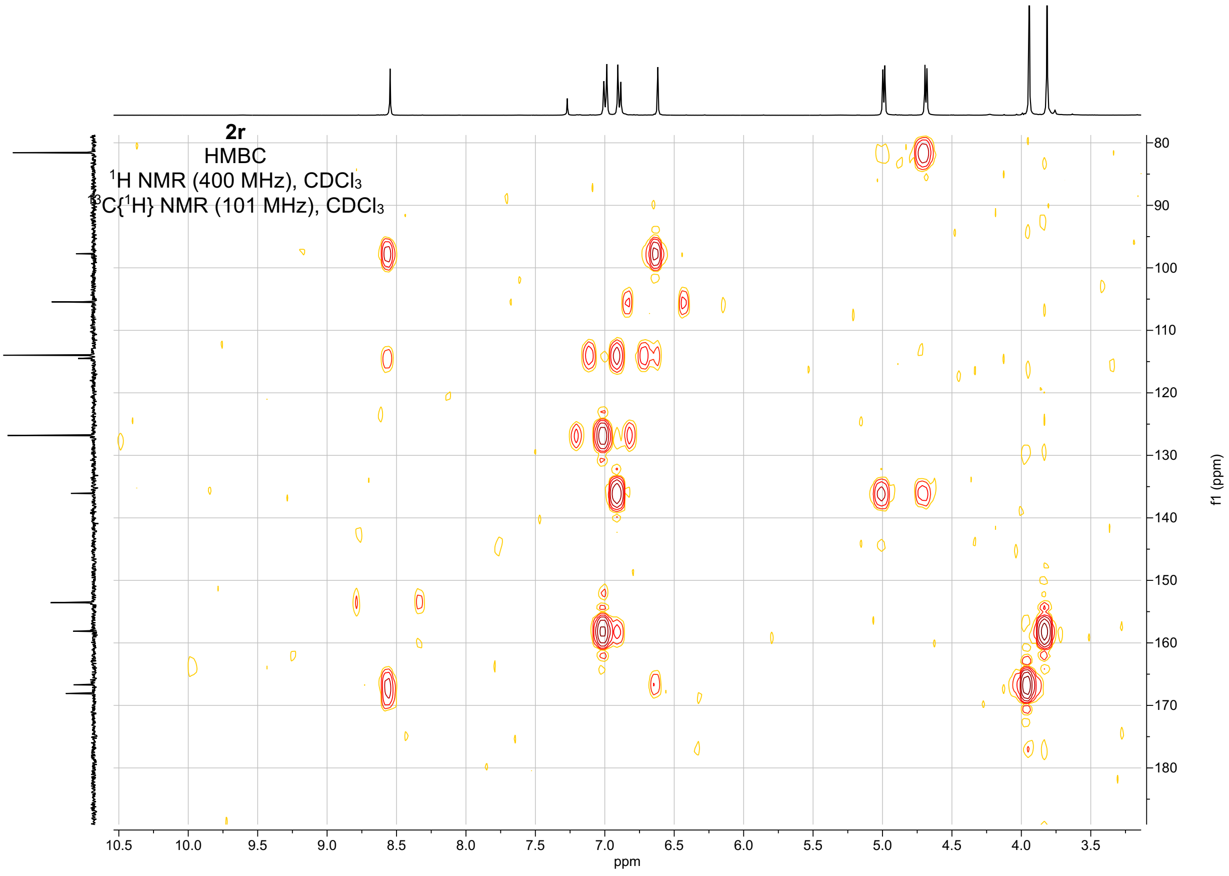

**2r**
HMBC
1H NMR (400 MHz), CDCl3
13C{1H} NMR (101 MHz), CDCl3
ppm
f1 (ppm)

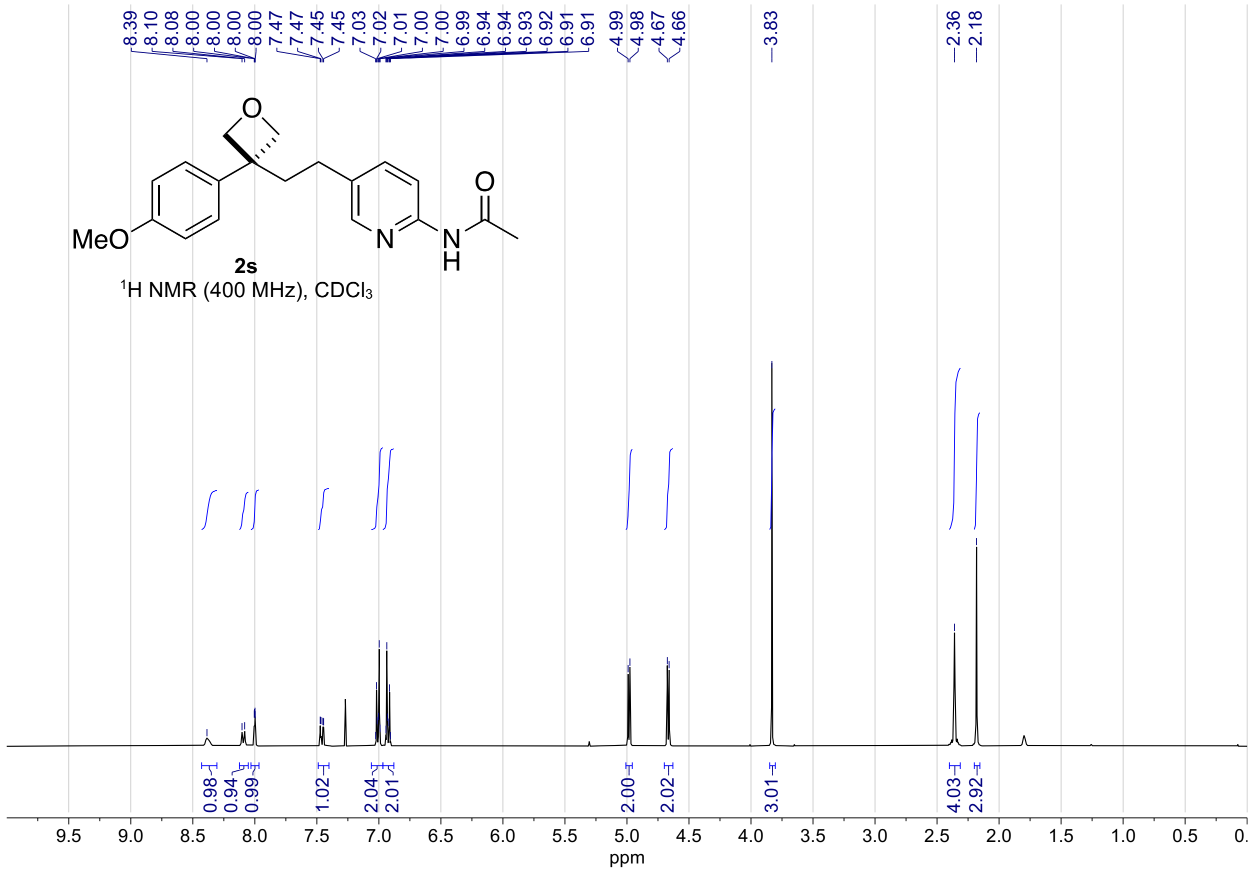
8.39 8.10 8.08 8.00 8.00 8.00 8.00 7.47 7.47 7.45 7.45 7.03 7.02 7.01 7.00 7.00 6.99 6.94 6.94 6.93 6.92 6.91 6.91
4.99 4.98 4.67 4.66
3.83
2.36 2.18
O
MeO
N
N
H
O
2s
¹H NMR (400 MHz), CDCl₃
0.98 0.94 0.99 1.02 2.04 2.01 2.00 2.02 3.01 4.03 2.92
9.5 9.0 8.5 8.0 7.5 7.0 6.5 6.0 5.5 5.0 4.5 4.0 3.5 3.0 2.5 2.0 1.5 1.0 0.5 0.
ppm

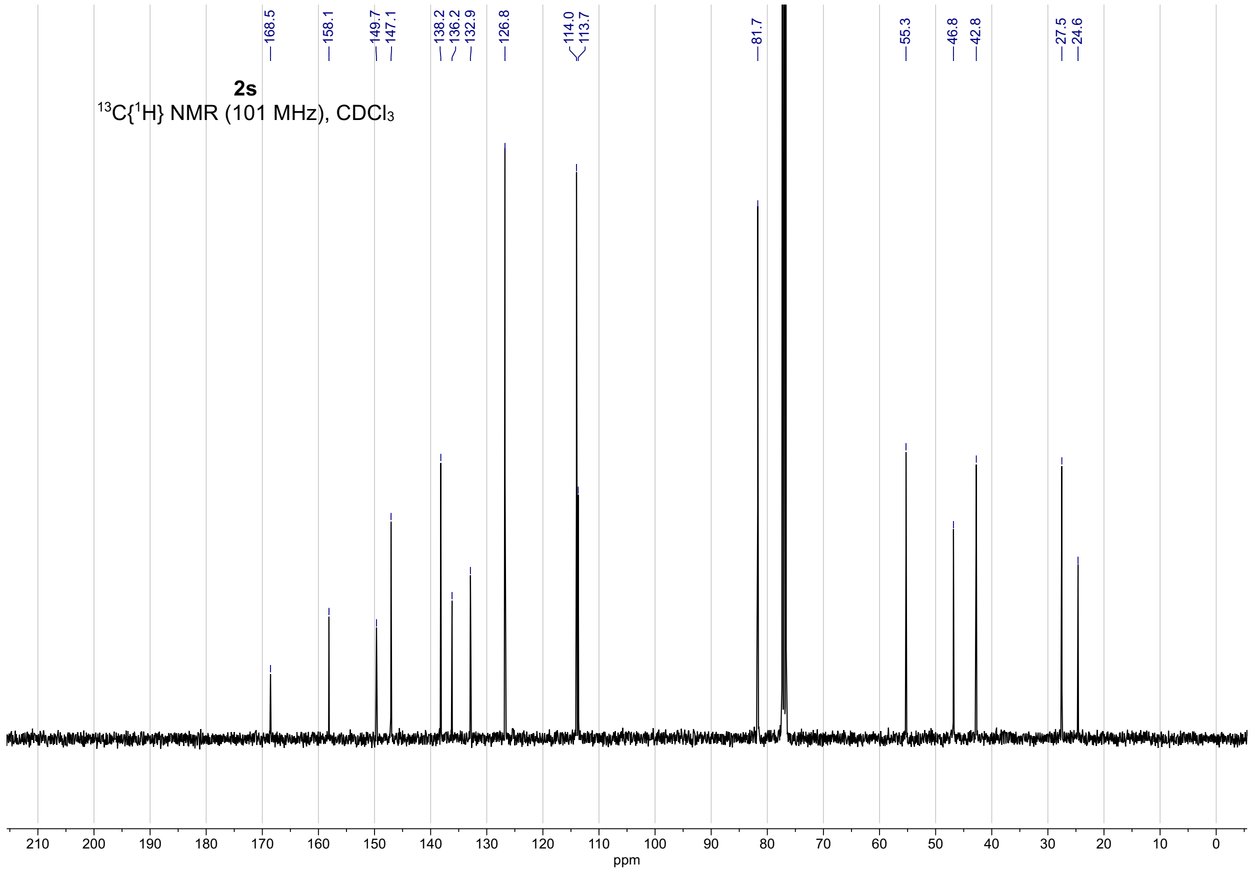
168.5 158.1 149.7 147.1 138.2 136.2 132.9 126.8 114.0 113.7 81.7 55.3 46.8 42.8 27.5 24.6
2s
¹³C{¹H} NMR (101 MHz), CDCl₃
210 200 190 180 170 160 150 140 130 120 110 100 90 80 70 60 50 40 30 20 10 0
ppm

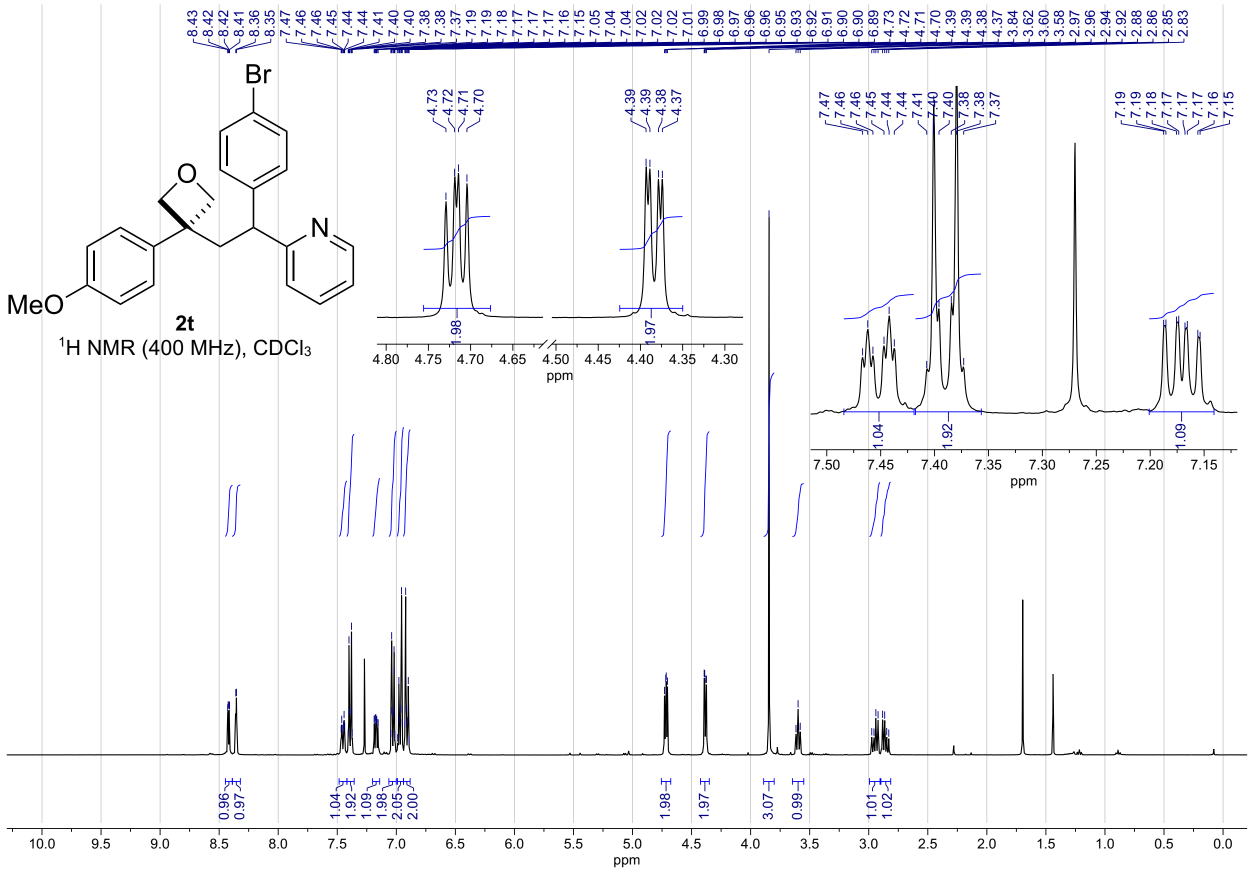

2t
[1]H NMR (400 MHz), CDCl3
MeO
Br
O
N
ppm


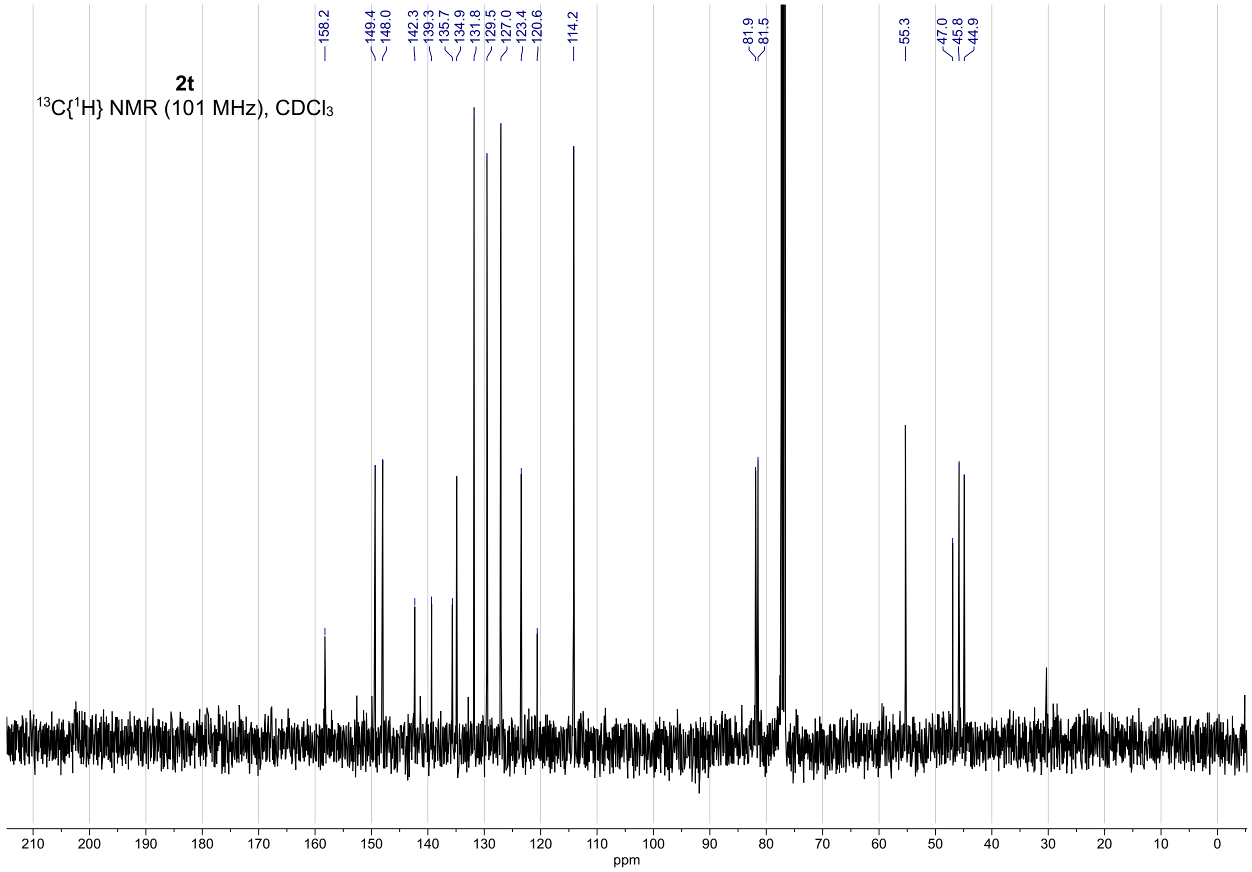

2t
[13]C{[1]H} NMR (101 MHz), CDCl3
158.2
149.4
148.0
142.3
139.3
135.7
134.9
131.8
129.5
127.0
123.4
120.6
114.2
81.9
81.5
55.3
47.0
45.8
44.9
ppm

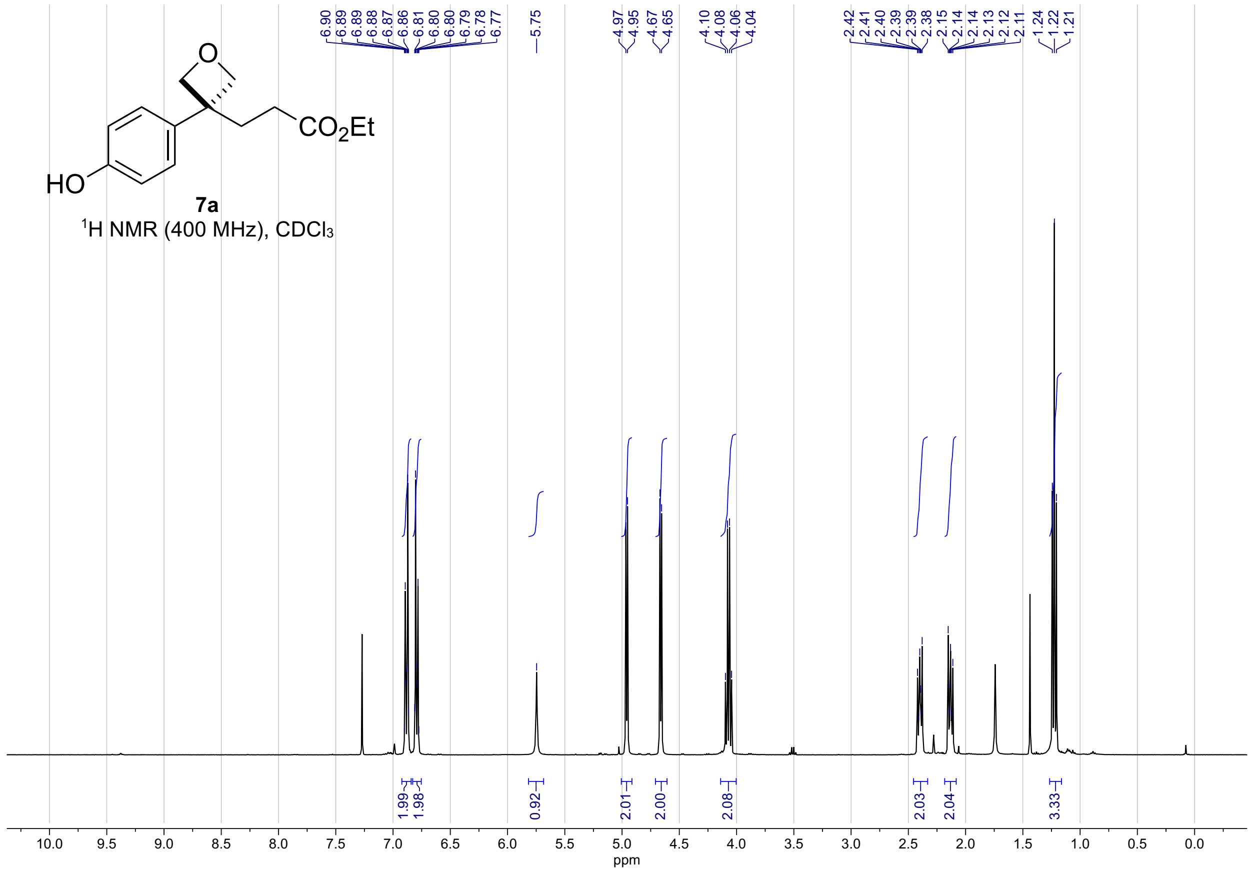
6.90 6.89 6.89 6.88 6.87 6.86 6.81 6.80 6.80 6.79 6.78 6.77
5.75
4.97 4.95 4.67 4.65
4.10 4.08 4.06 4.04
2.42 2.41 2.40 2.39 2.39 2.38 2.15 2.14 2.14 2.13 2.12 2.11 1.24 1.22 1.21
O
CO2Et
HO
7a
1H NMR (400 MHz), CDCl3
1.99 1.98 0.92 2.01 2.00 2.08 2.03 2.04 3.33
ppm

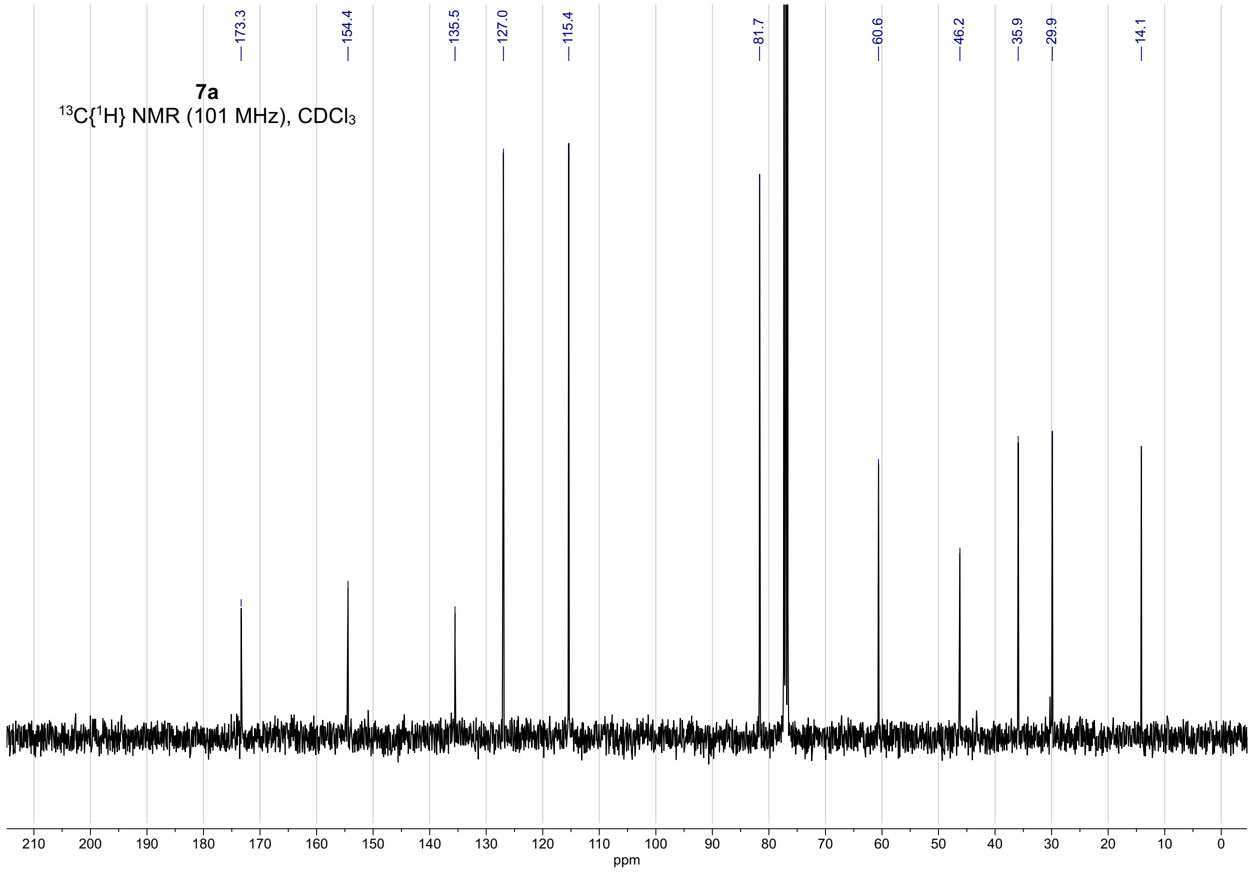
173.3
154.4
135.5
127.0
115.4
81.7
60.6
46.2
35.9
29.9
14.1
7a
13C{1H} NMR (101 MHz), CDCl3
ppm

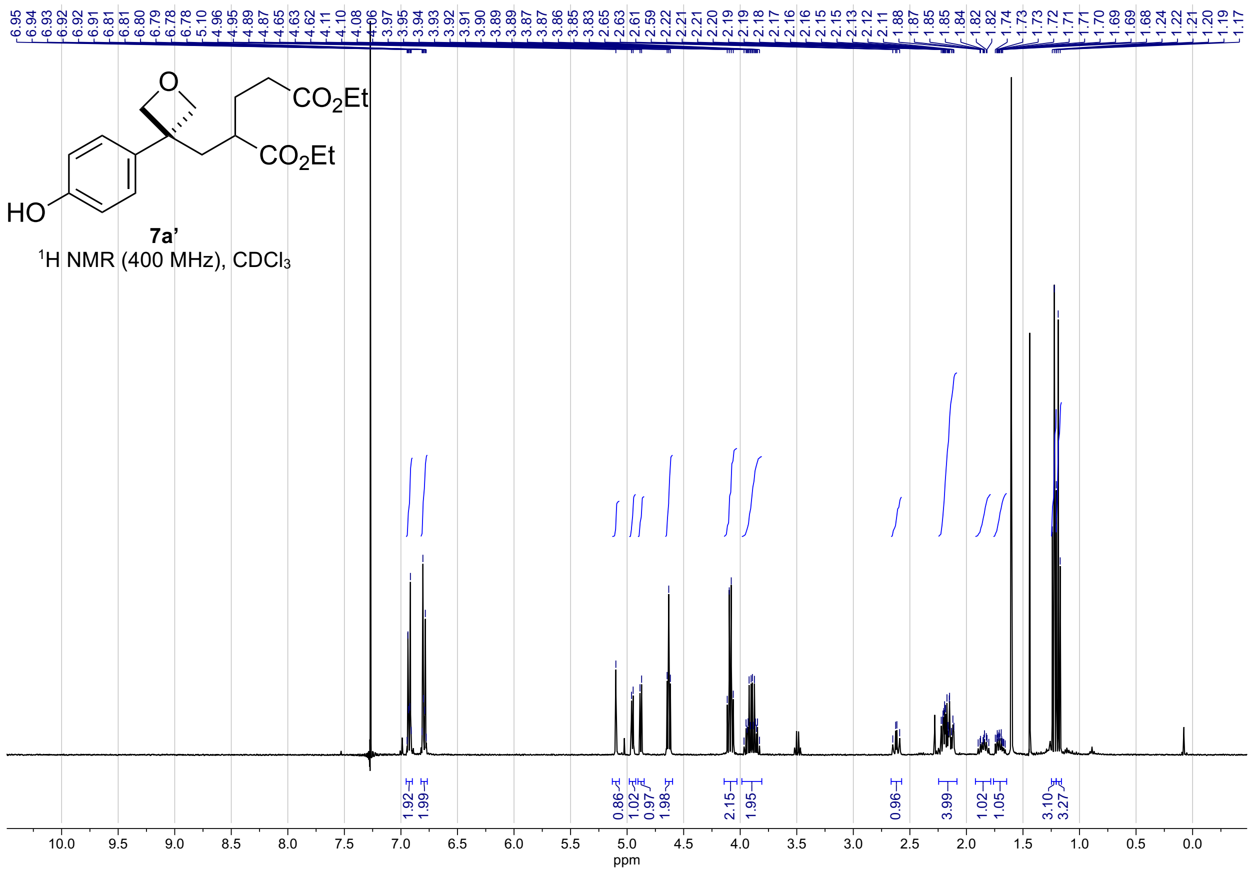

6.95 6.94 6.93 6.92 6.92 6.91 6.81 6.81 6.80 6.79 6.78 6.78 5.10 4.96 4.95 4.89 4.87 4.65 4.63 4.62 4.11 4.10 4.08 4.06 3.97 3.95 3.94 3.93 3.92 3.91 3.90 3.89 3.89 3.87 3.87 3.86 3.85 3.83 2.65 2.63 2.61 2.59 2.22 2.22 2.21 2.21 2.20 2.19 2.19 2.18 2.17 2.16 2.16 2.15 2.15 2.13 2.12 2.11 1.88 1.87 1.85 1.85 1.84 1.82 1.82 1.74 1.73 1.73 1.72 1.72 1.71 1.71 1.70 1.69 1.69 1.68 1.24 1.22 1.21 1.20 1.19 1.17
CO2Et
CO2Et
HO
7a'
1H NMR (400 MHz), CDCl3
1.92 1.99 0.86 1.02 0.97 1.98 2.15 1.95 0.96 3.99 1.02 1.05 3.10 3.27
10.0 9.5 9.0 8.5 8.0 7.5 7.0 6.5 6.0 5.5 5.0 4.5 4.0 3.5 3.0 2.5 2.0 1.5 1.0 0.5 0.0
ppm


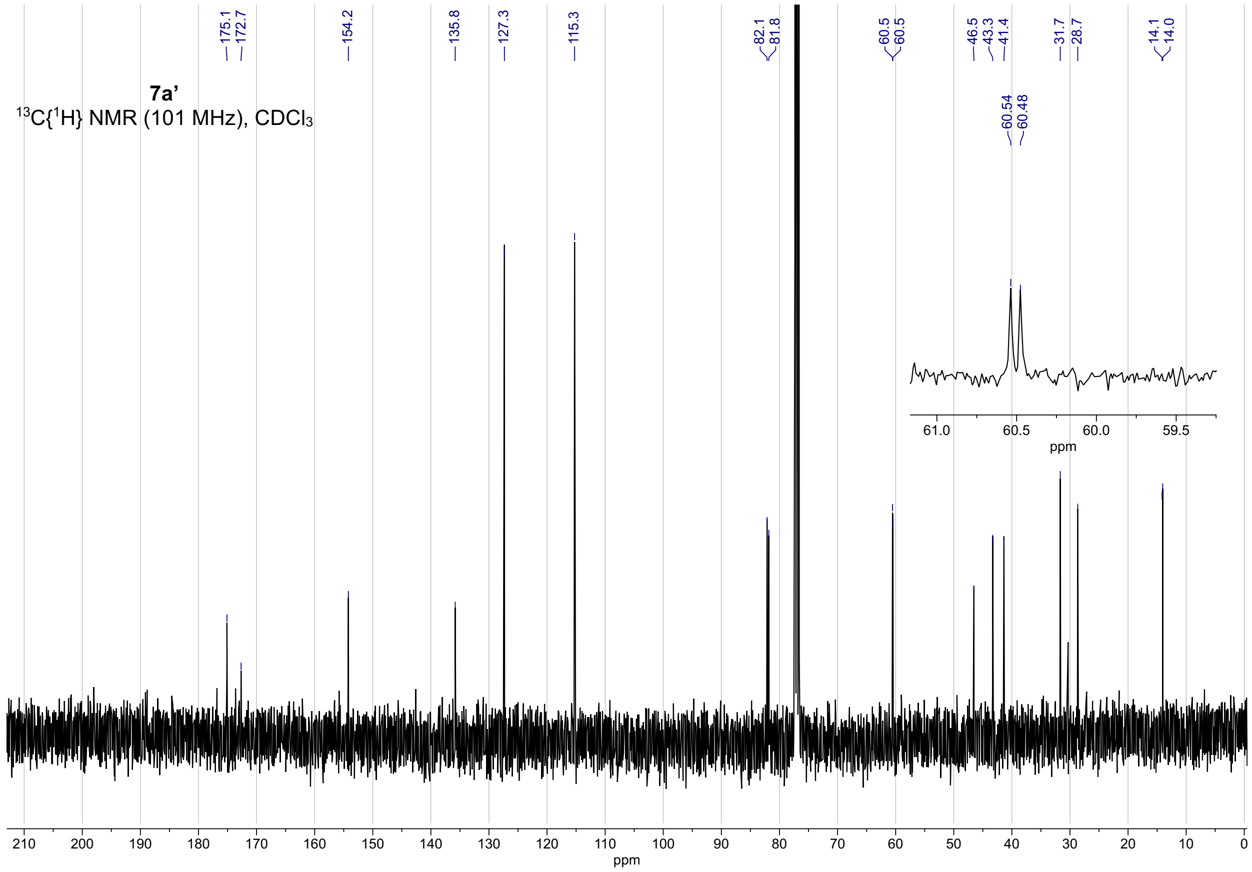

175.1 172.7 154.2 135.8 127.3 115.3 82.1 81.8 60.5 60.5 46.5 43.3 41.4 31.7 28.7 14.1 14.0
7a'
13C{1H} NMR (101 MHz), CDCl3
60.54 60.48
61.0 60.5 60.0 59.5
ppm
210 200 190 180 170 160 150 140 130 120 110 100 90 80 70 60 50 40 30 20 10 0
ppm

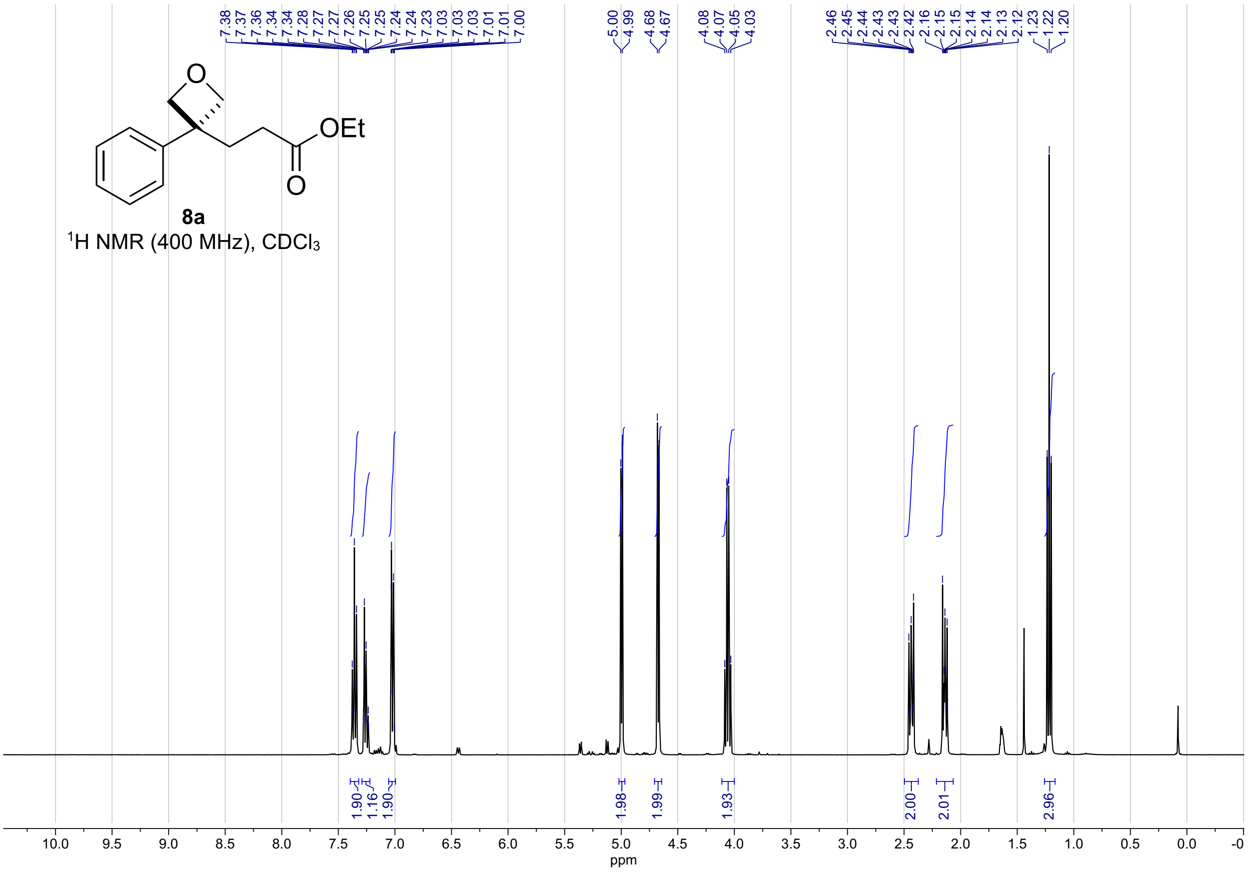
8a
$^1$H NMR (400 MHz), CDCl$_3$
ppm

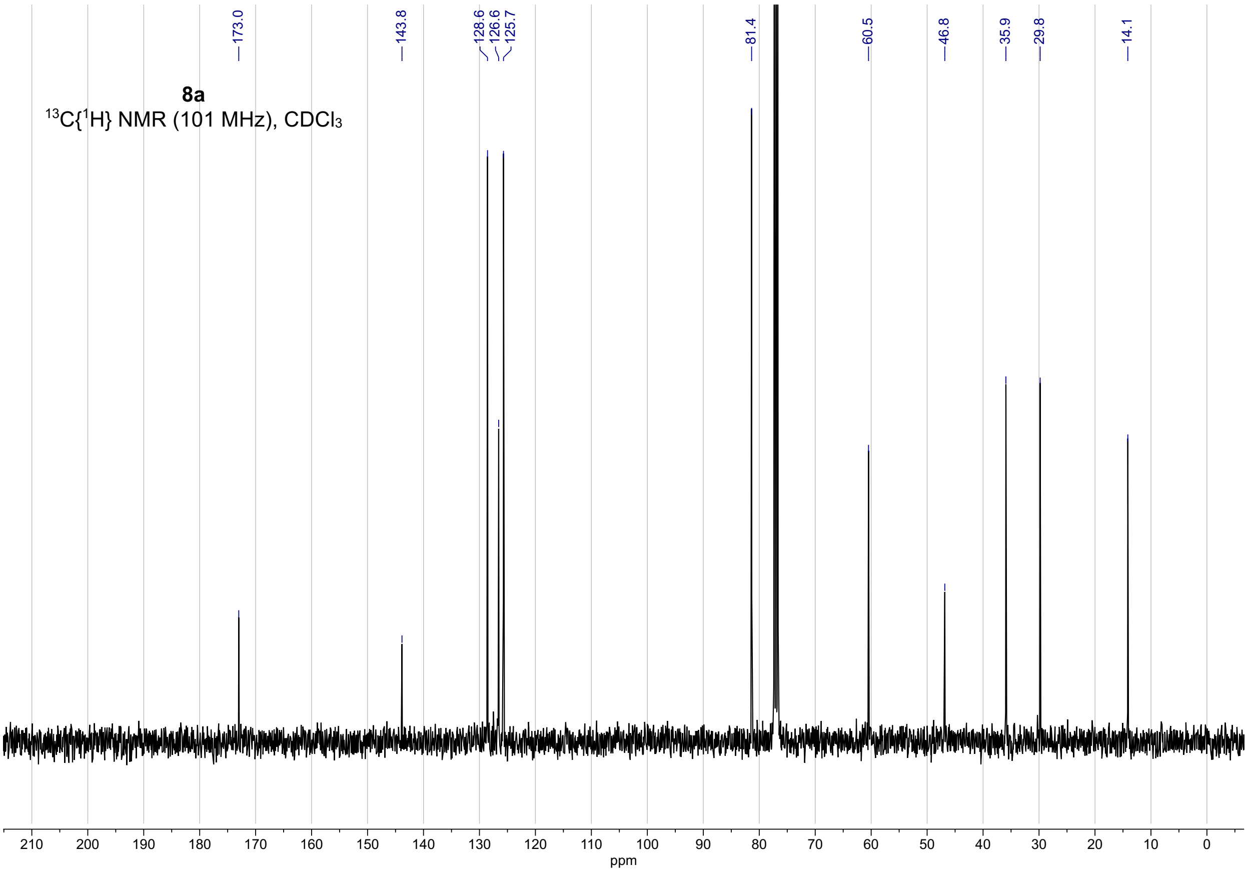
8a
$^{13}$C{$^1$H} NMR (101 MHz), CDCl$_3$
173.0
143.8
128.6
126.6
125.7
81.4
60.5
46.8
35.9
29.8
14.1
ppm

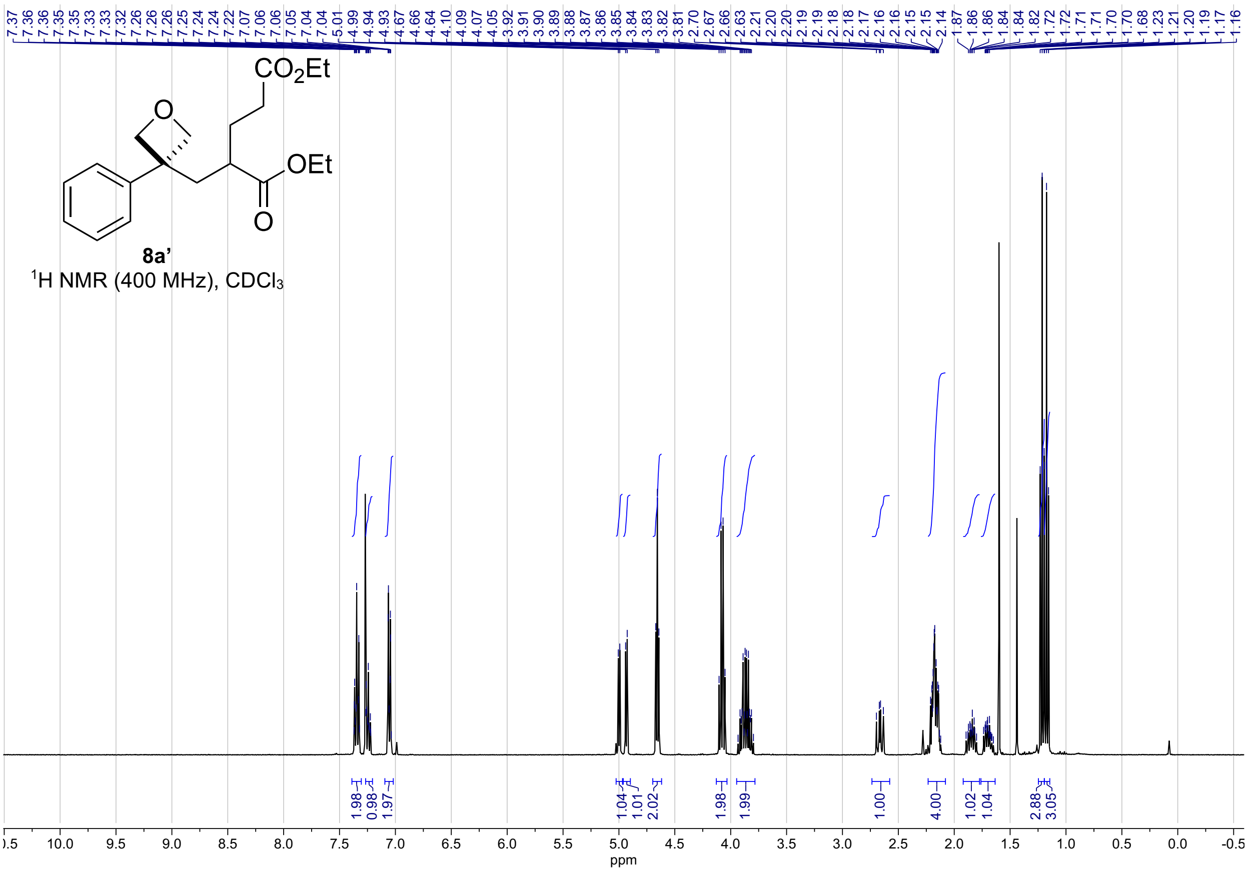
CO2Et
O
OEt
O
8a'
1H NMR (400 MHz), CDCl3
ppm

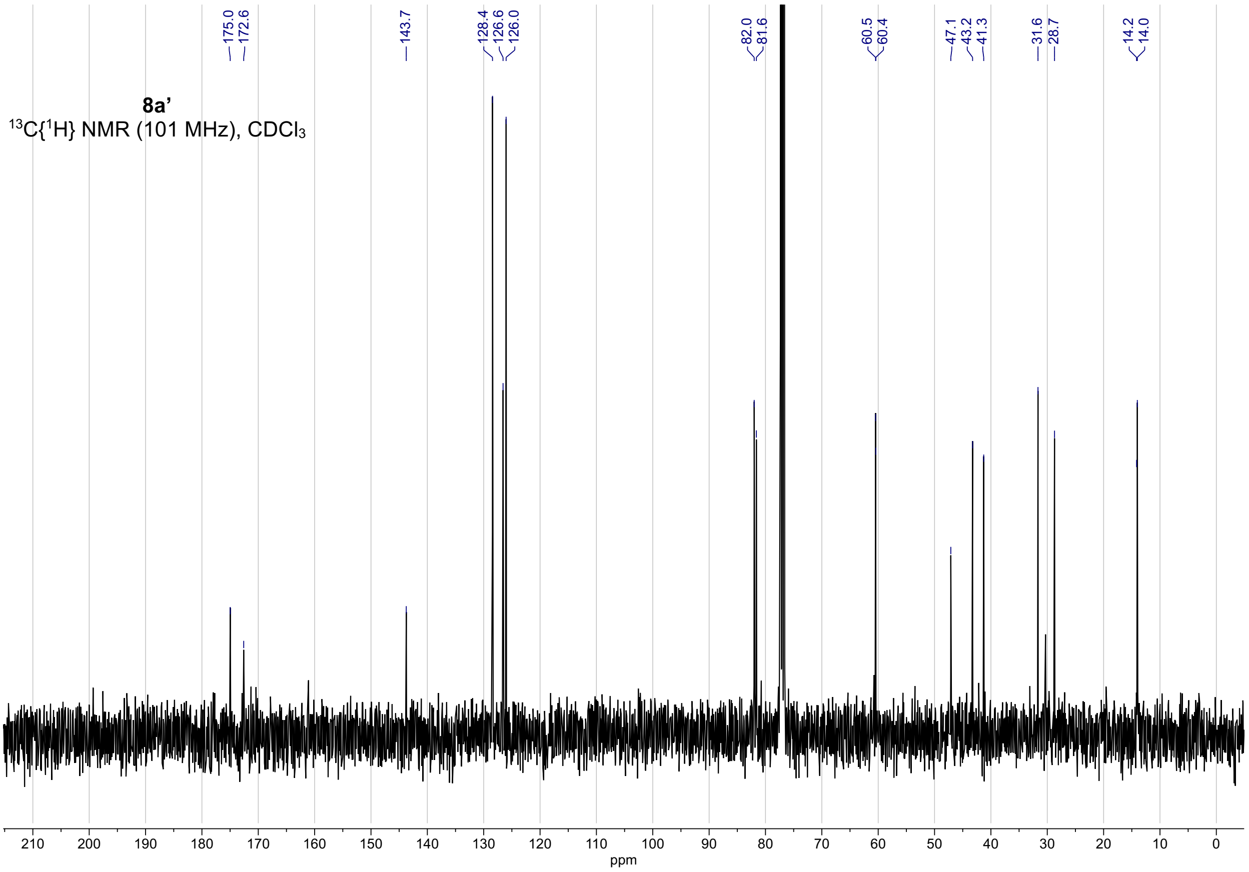
8a'
13C{1H} NMR (101 MHz), CDCl3
175.0
172.6
143.7
128.4
126.6
126.0
82.0
81.6
60.5
60.4
47.1
43.2
41.3
31.6
28.7
14.2
14.0
ppm

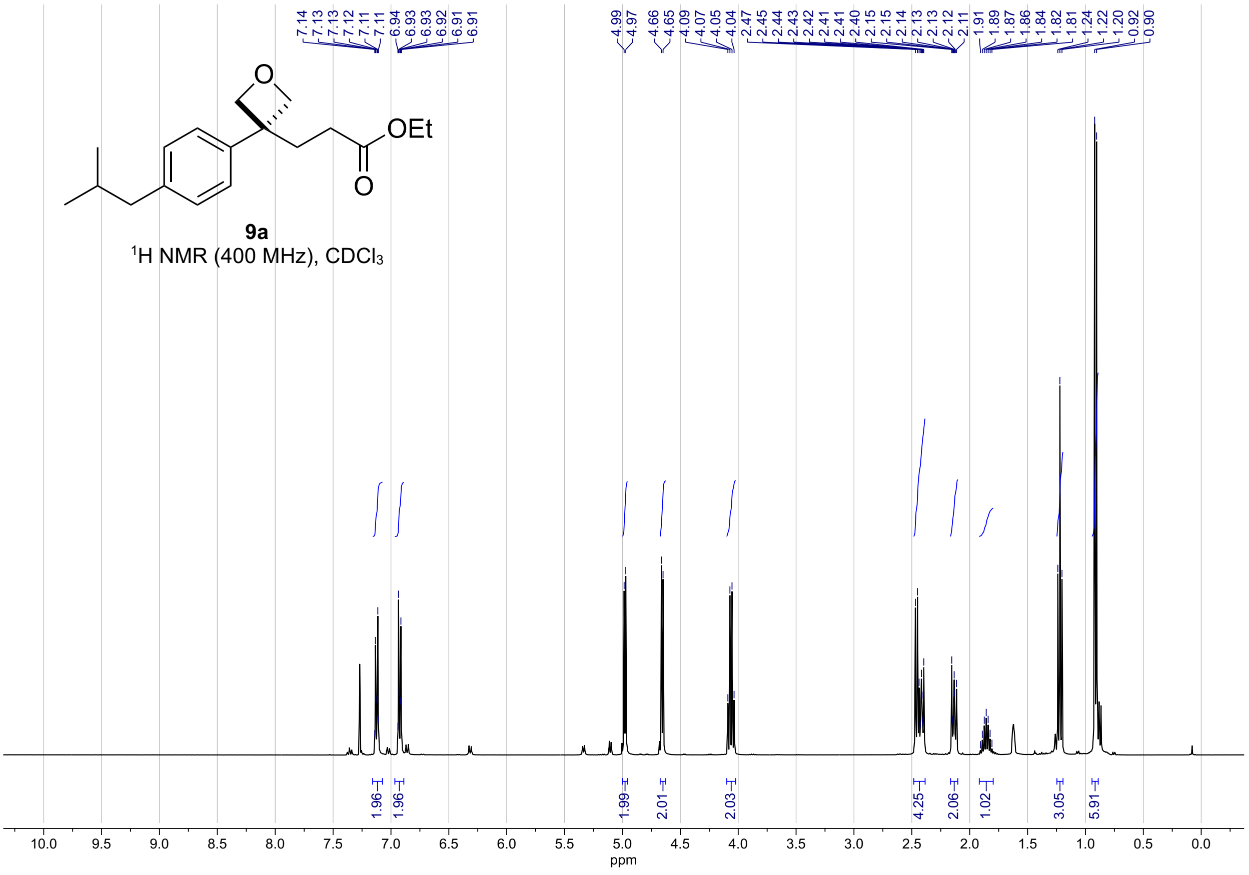
7.14 7.13 7.13 7.12 7.11 7.11 6.94 6.93 6.93 6.92 6.91 6.91
4.99 4.97 4.66 4.65 4.09 4.07 4.05 4.04 2.47 2.45 2.44 2.43 2.42 2.41 2.41 2.40 2.15 2.15 2.14 2.13 2.13 2.12 2.11 1.91 1.89 1.87 1.86 1.84 1.82 1.81 1.24 1.22 1.20 0.92 0.90
O
OEt
O
9a
1H NMR (400 MHz), CDCl3
1.96 1.96 1.99 2.01 2.03 4.25 2.06 1.02 3.05 5.91
10.0 9.5 9.0 8.5 8.0 7.5 7.0 6.5 6.0 5.5 5.0 4.5 4.0 3.5 3.0 2.5 2.0 1.5 1.0 0.5 0.0
ppm

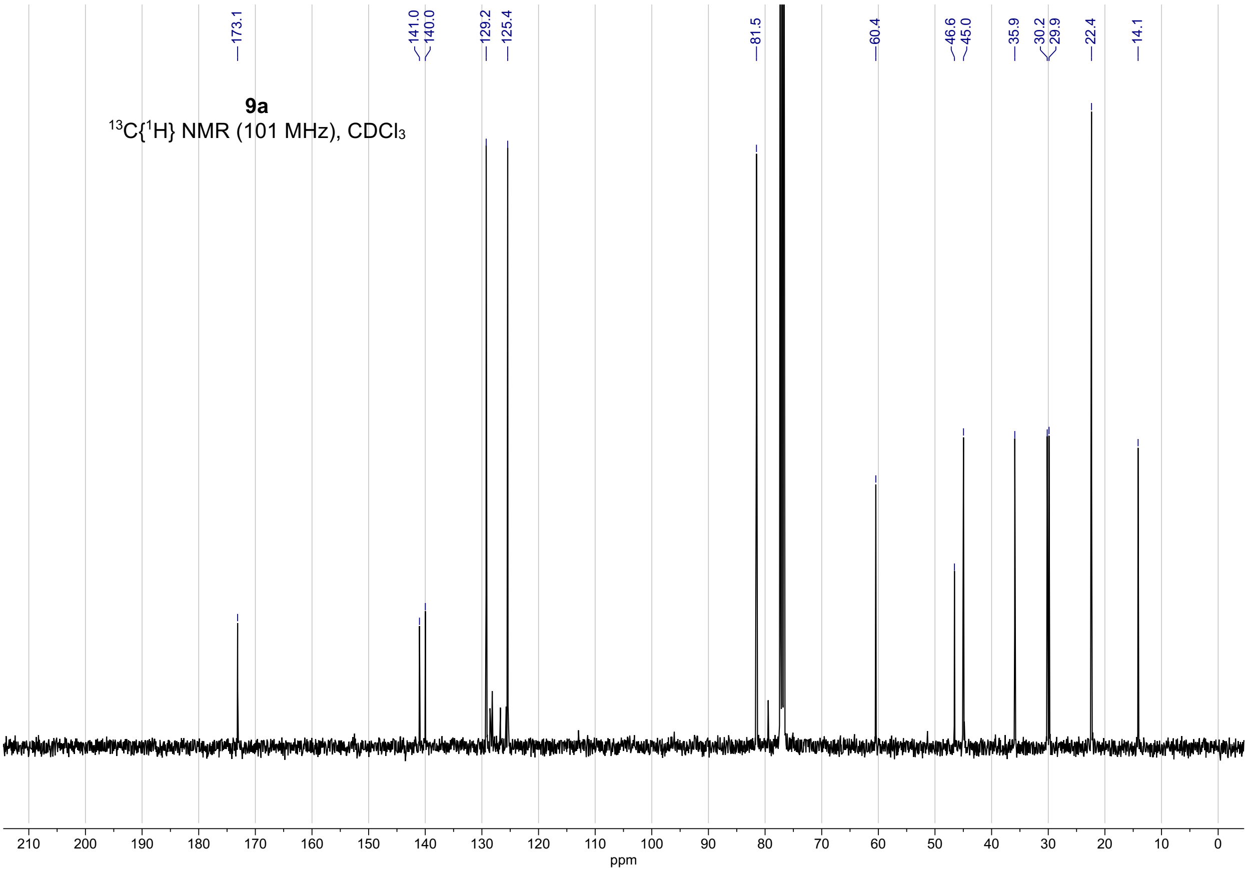
173.1 141.0 140.0 129.2 125.4 81.5 60.4 46.6 45.0 35.9 30.2 29.9 22.4 14.1
9a
13C{1H} NMR (101 MHz), CDCl3
210 200 190 180 170 160 150 140 130 120 110 100 90 80 70 60 50 40 30 20 10 0
ppm

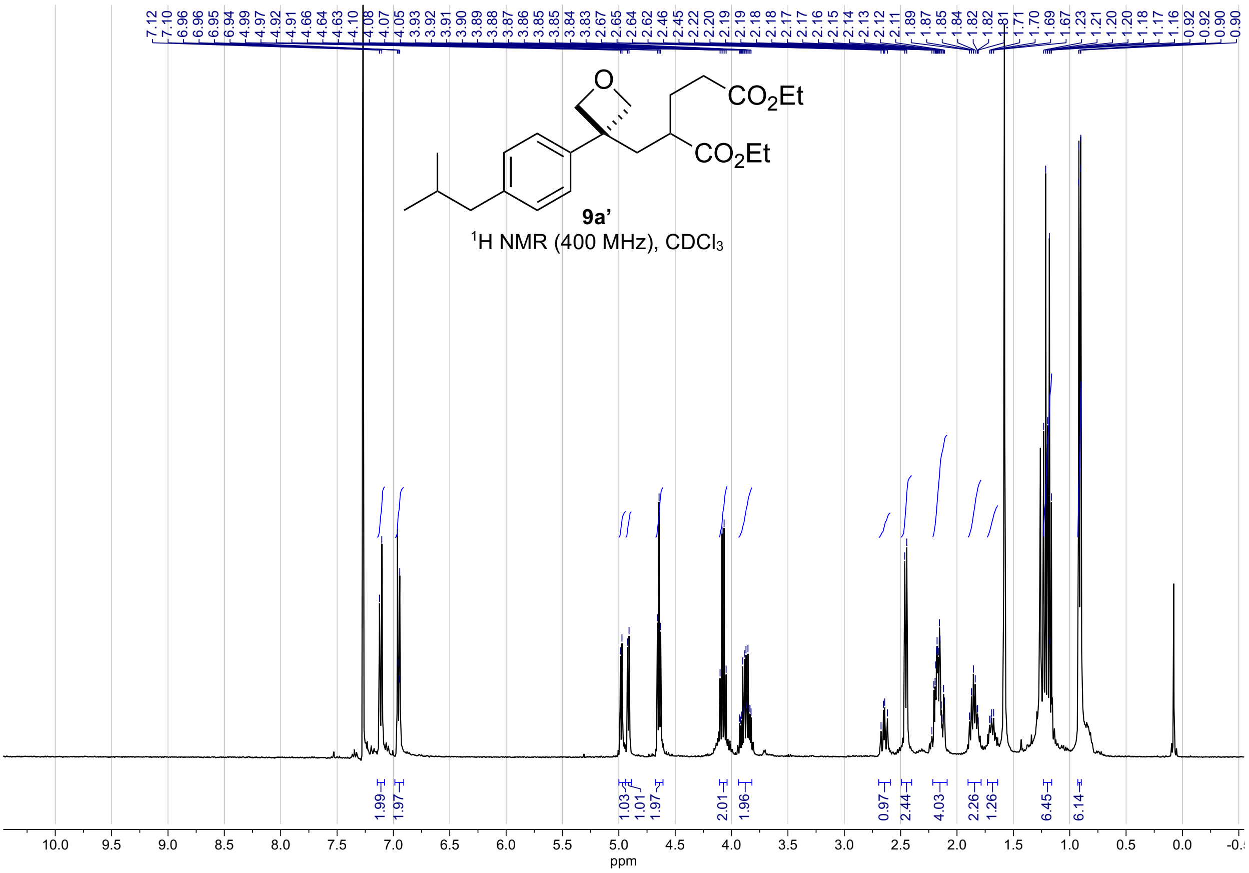
CO2Et
CO2Et
O
9a’
1H NMR (400 MHz), CDCl3
ppm

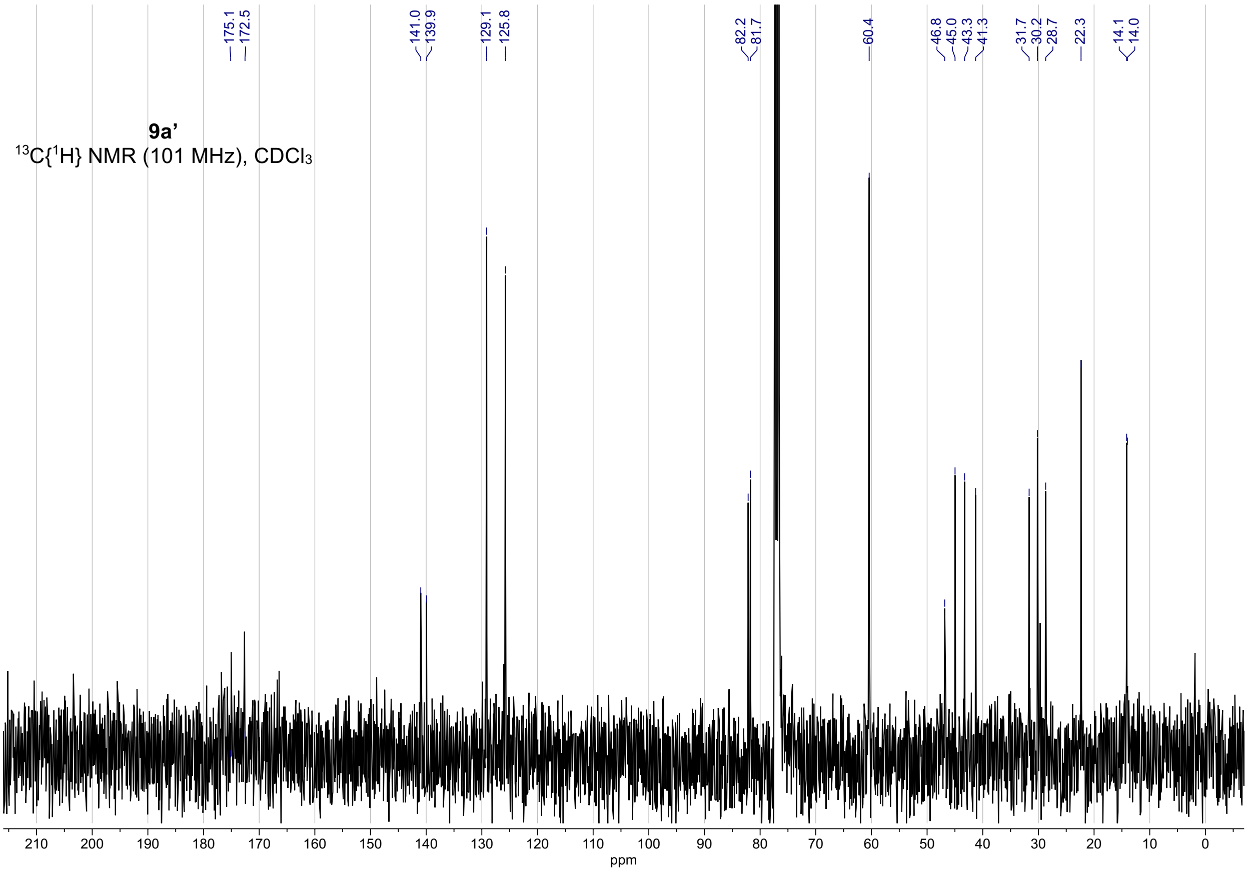
9a’
13C{1H} NMR (101 MHz), CDCl3
ppm

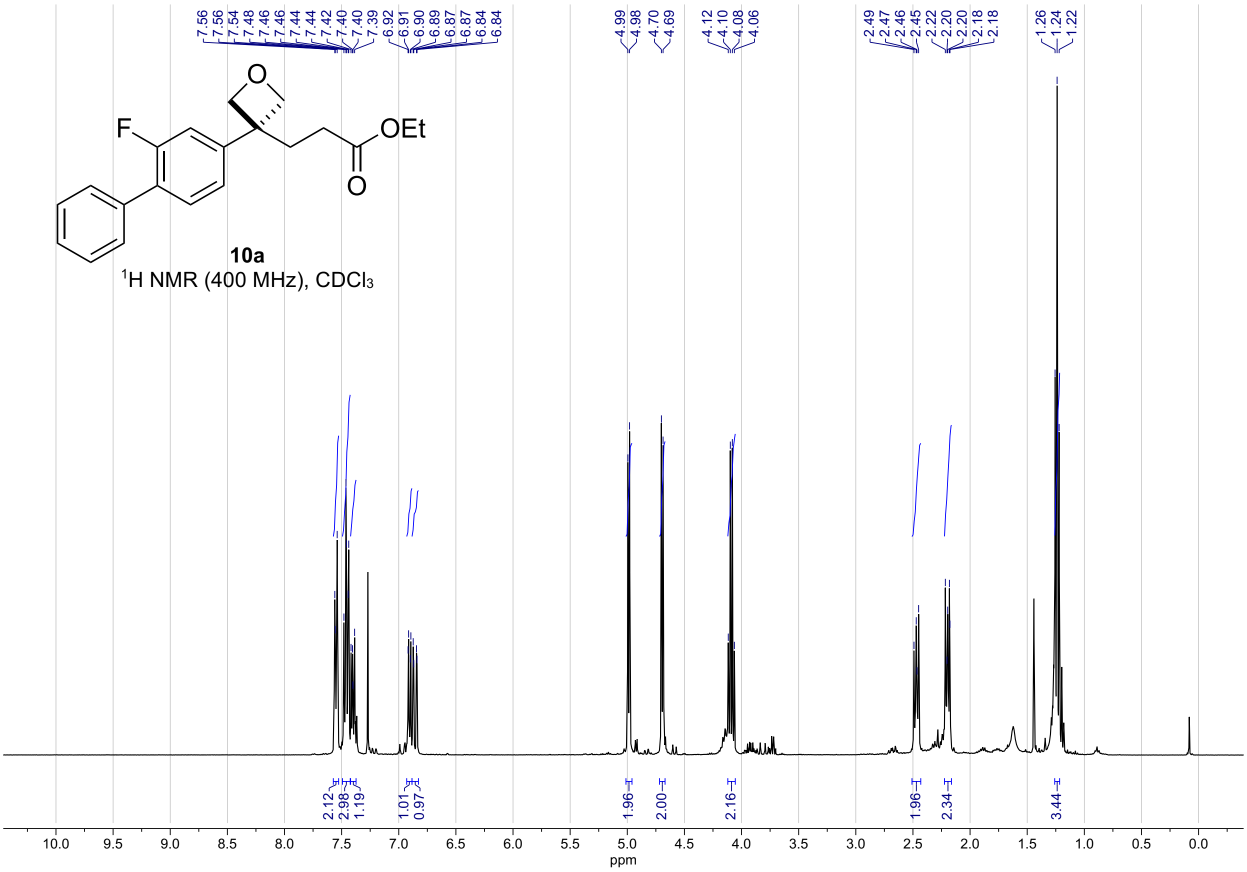
7.56
7.56
7.54
7.48
7.46
7.46
7.44
7.44
7.42
7.40
7.40
7.39
6.92
6.91
6.90
6.89
6.87
6.87
6.84
6.84
4.99
4.98
4.70
4.69
4.12
4.10
4.08
4.06
2.49
2.47
2.46
2.45
2.22
2.20
2.20
2.18
2.18
1.26
1.24
1.22
F
O
OEt
O
10a
¹H NMR (400 MHz), CDCl3
2.12
2.98
1.19
1.01
0.97
1.96
2.00
2.16
1.96
2.34
3.44
ppm

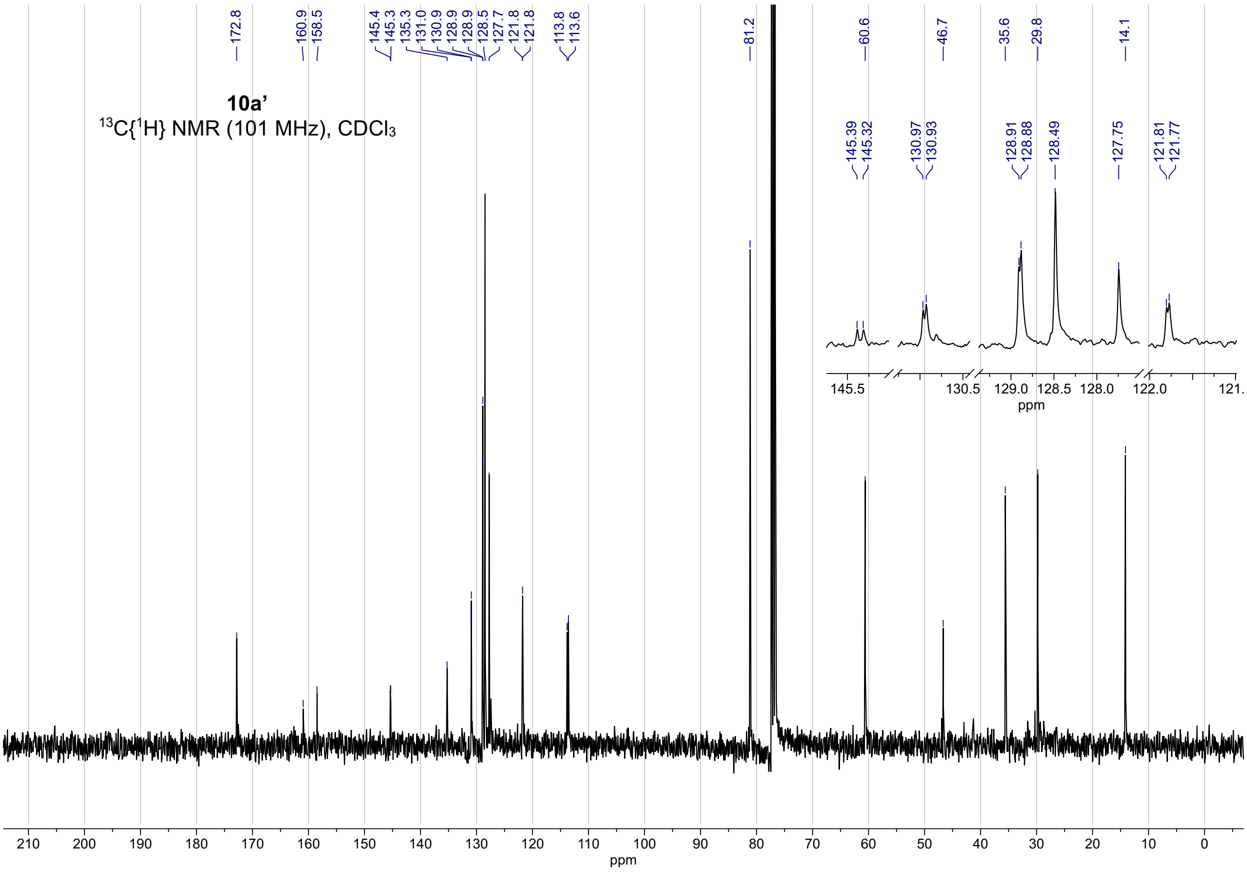
172.8
160.9
158.5
145.4
145.3
135.3
131.0
130.9
128.9
128.9
128.5
127.7
121.8
121.8
113.8
113.6
81.2
60.6
46.7
35.6
29.8
14.1
10a'
¹³C{¹H} NMR (101 MHz), CDCl3
145.39
145.32
130.97
130.93
128.91
128.88
128.49
127.75
121.81
121.77
ppm
ppm

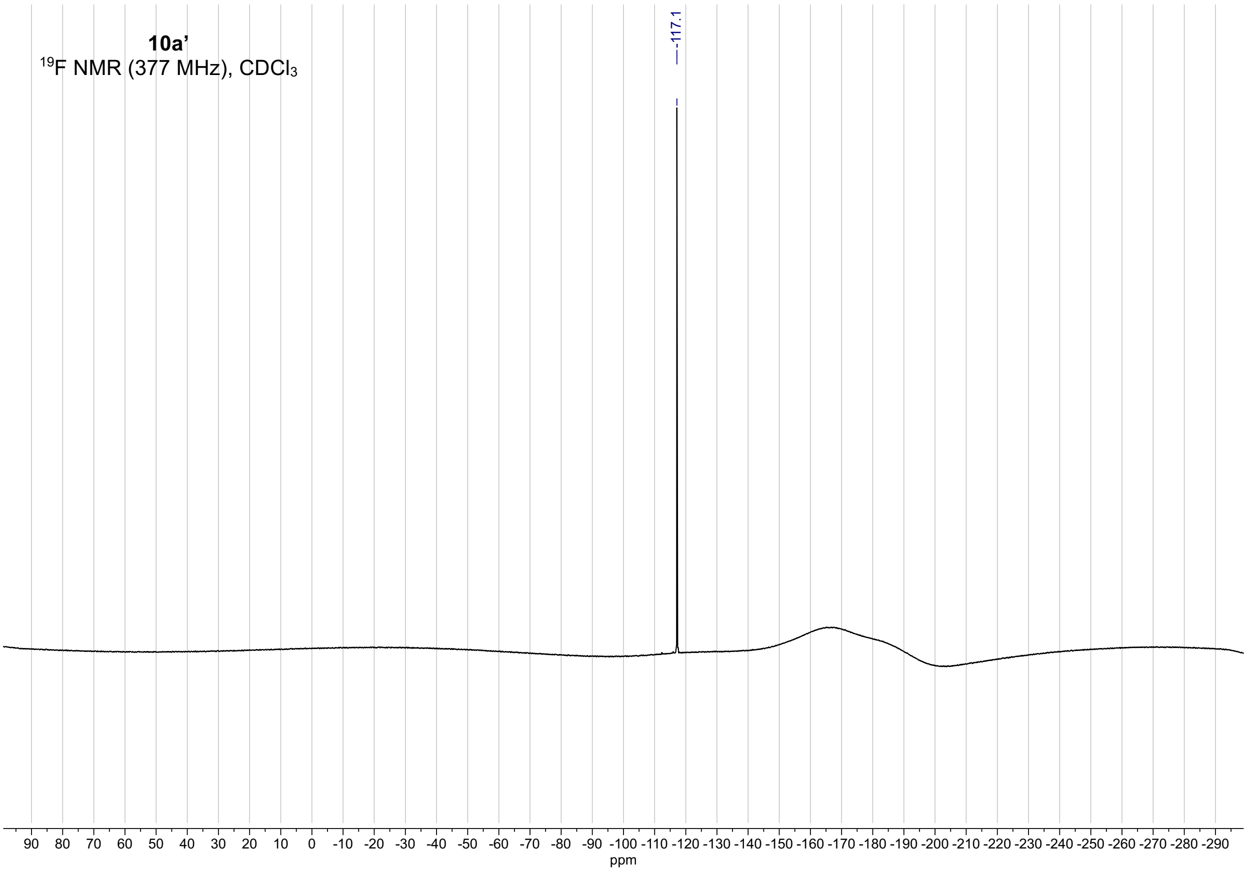

**10a'**
$^{19}$F NMR (377 MHz), CDCl$_3$
-117.1
90 80 70 60 50 40 30 20 10 0 -10 -20 -30 -40 -50 -60 -70 -80 -90 -100 -110 -120 -130 -140 -150 -160 -170 -180 -190 -200 -210 -220 -230 -240 -250 -260 -270 -280 -290
ppm

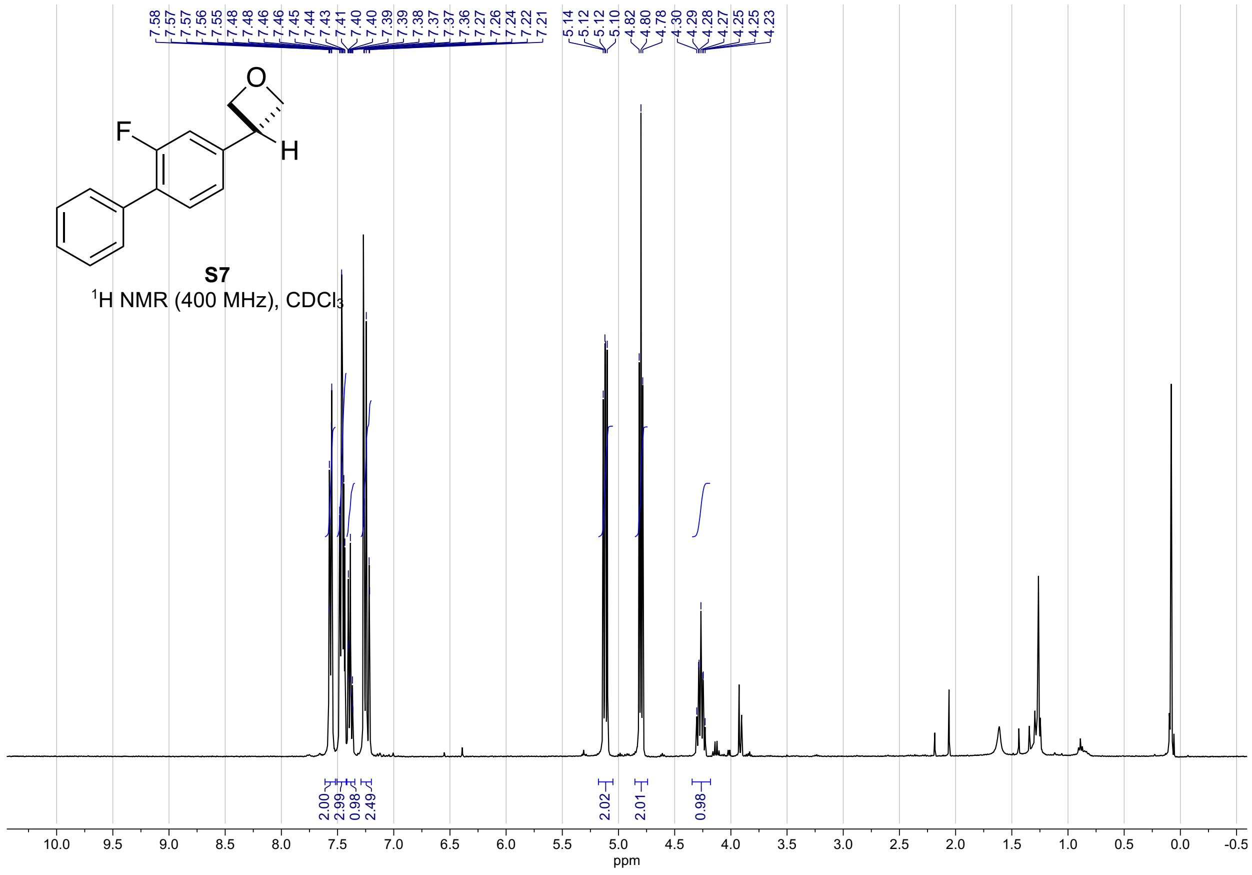
S7
$^{1}$H NMR (400 MHz), $CDCl_3$

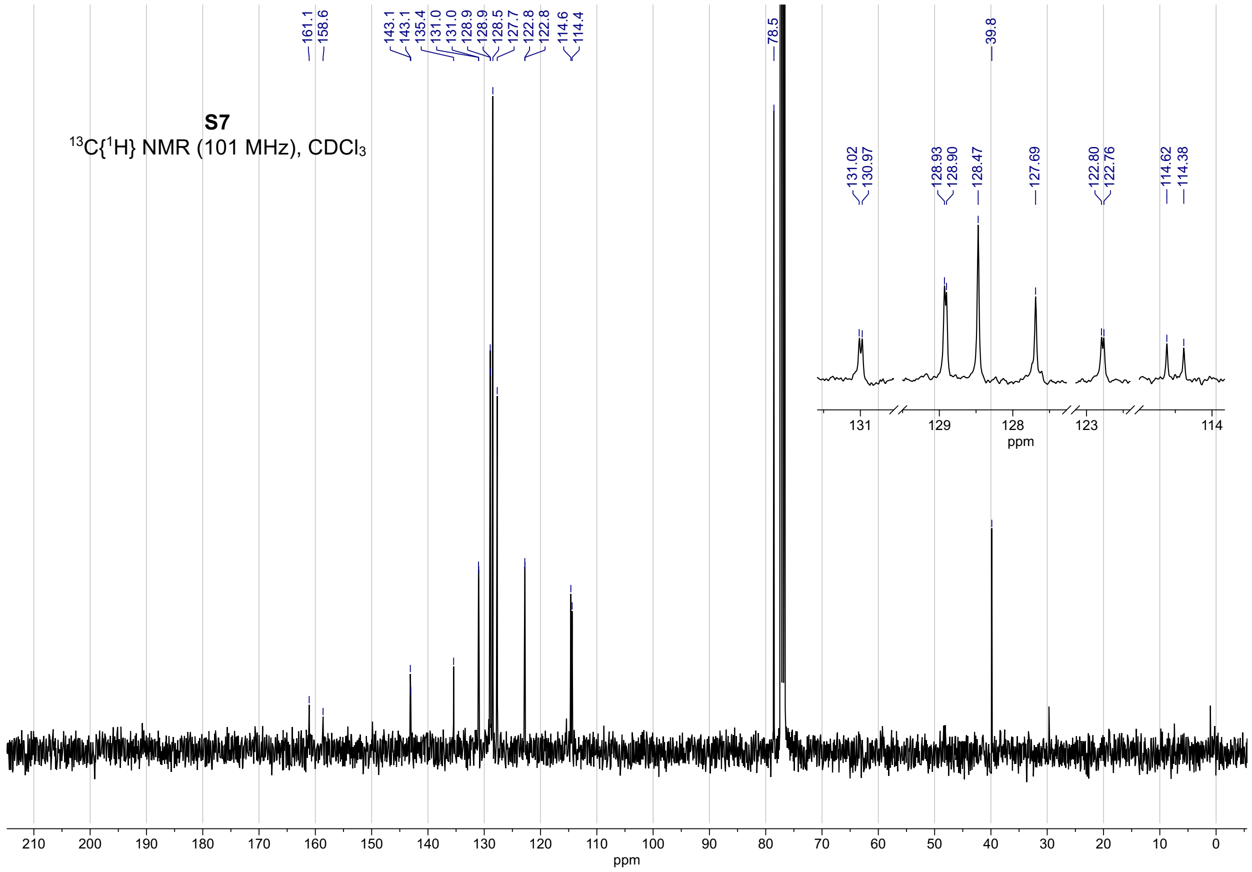
S7
$^{13}C\{^{1}H\}$ NMR (101 MHz), $CDCl_3$

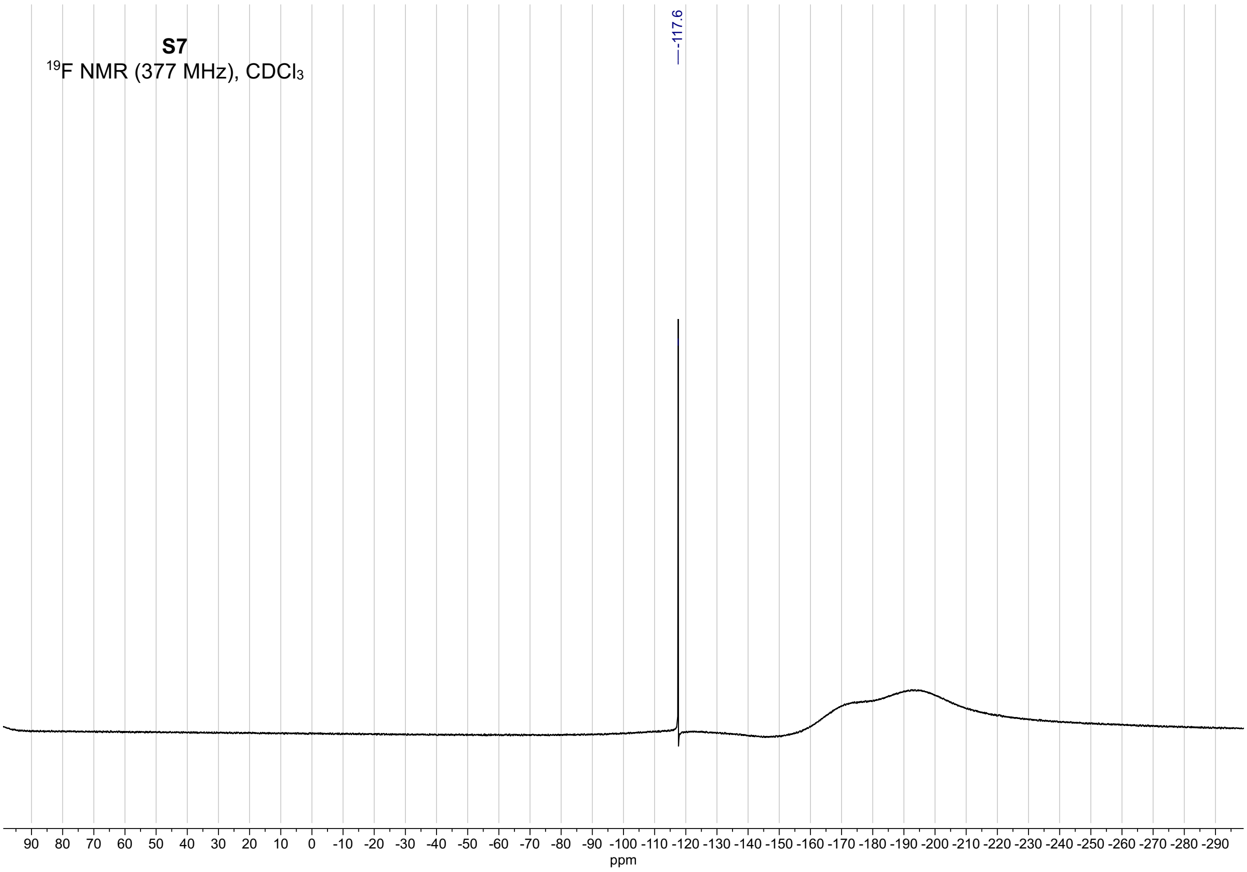
S7
$^{19}$F NMR (377 MHz), $CDCl_3$
-117.6
90 80 70 60 50 40 30 20 10 0 -10 -20 -30 -40 -50 -60 -70 -80 -90 -100 -110 -120 -130 -140 -150 -160 -170 -180 -190 -200 -210 -220 -230 -240 -250 -260 -270 -280 -290
ppm

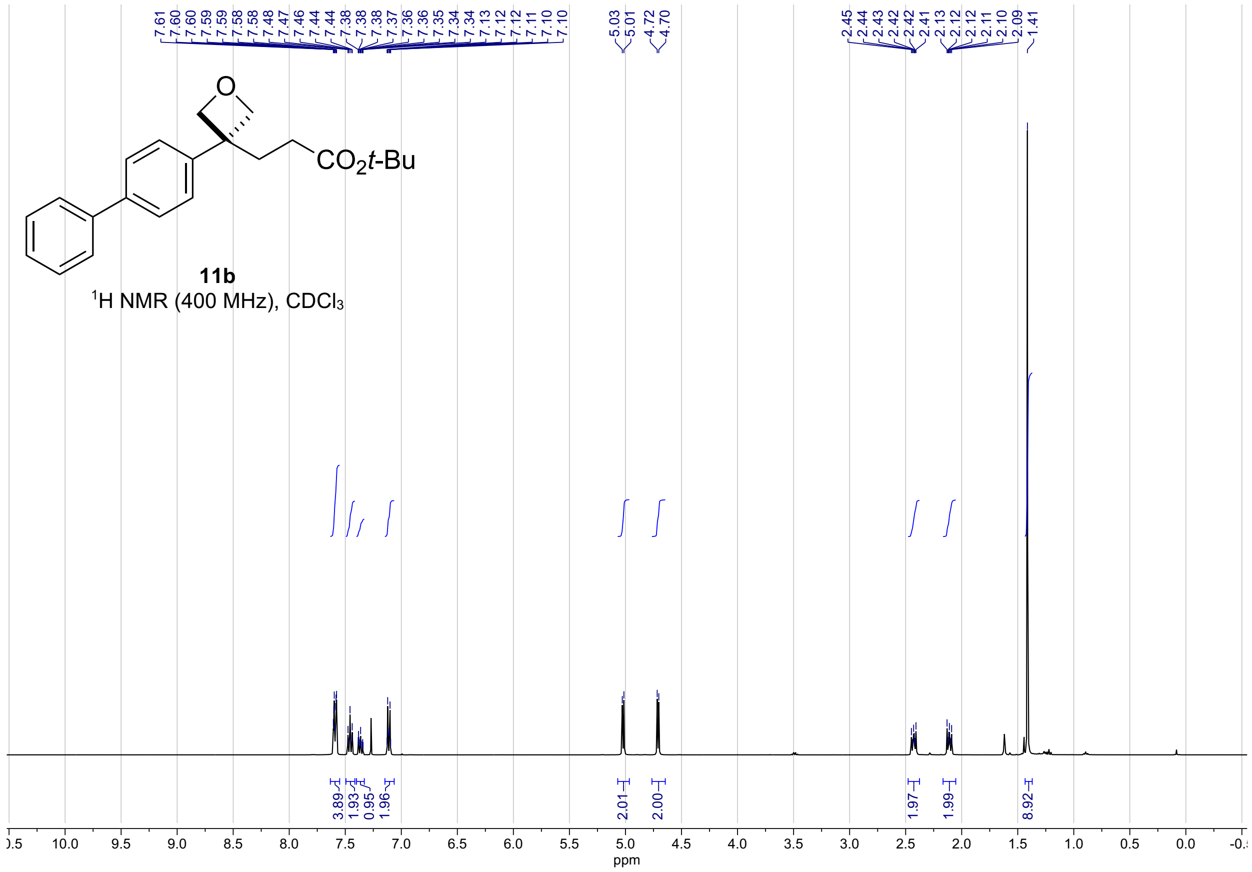
7.61 7.60 7.60 7.59 7.59 7.58 7.58 7.48 7.47 7.46 7.44 7.44 7.38 7.38 7.38 7.37 7.36 7.36 7.35 7.34 7.34 7.13 7.12 7.12 7.12 7.11 7.10 7.10
5.03 5.01 4.72 4.70
2.45 2.44 2.43 2.42 2.42 2.41 2.13 2.12 2.12 2.11 2.10 2.09 1.41
CO2t-Bu
**11b**
1H NMR (400 MHz), CDCl3
3.89 1.93 0.95 1.96 2.01 2.00 1.97 1.99 8.92
ppm

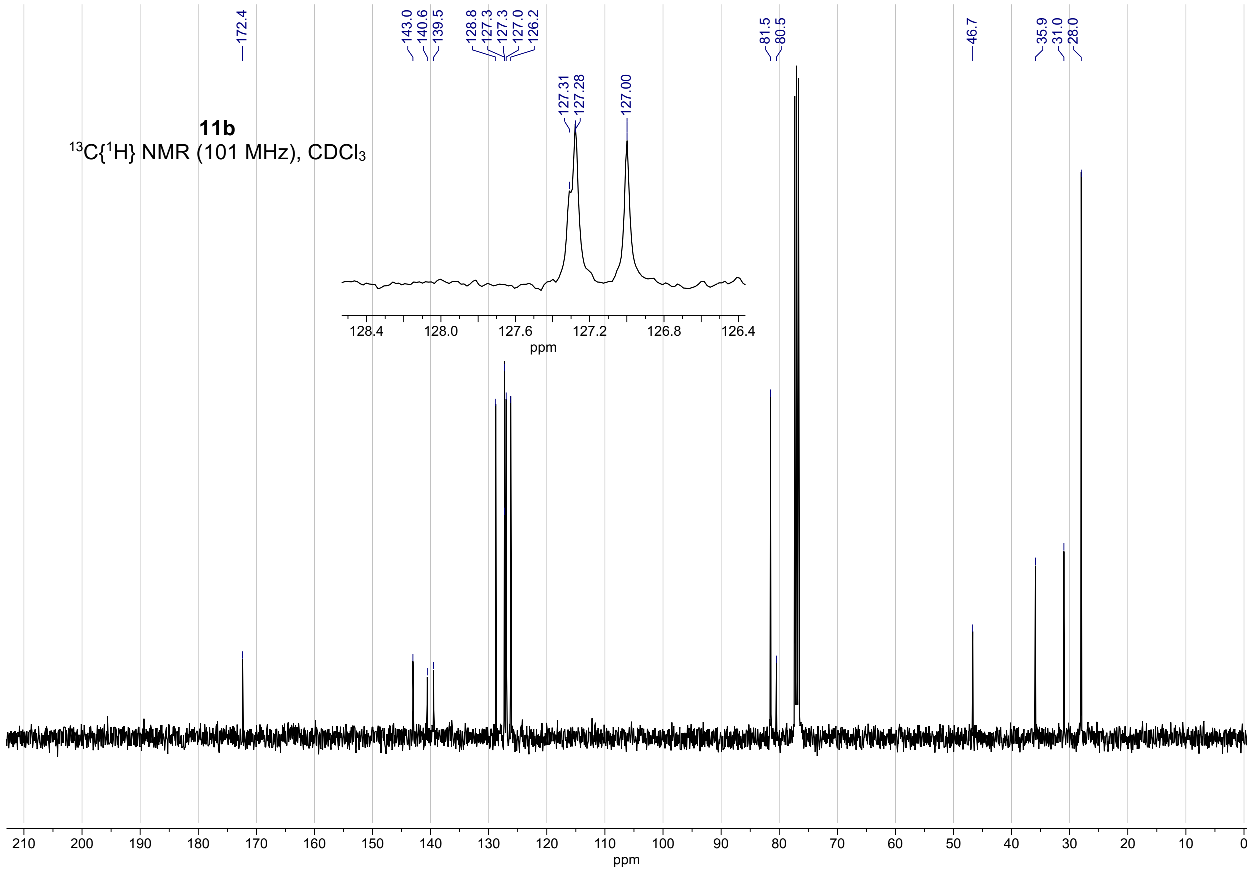
172.4
143.0 140.6 139.5
128.8 127.3 127.3 127.0 126.2
81.5 80.5
46.7
35.9 31.0 28.0
**11b**
13C{1H} NMR (101 MHz), CDCl3
127.31 127.28 127.00
ppm

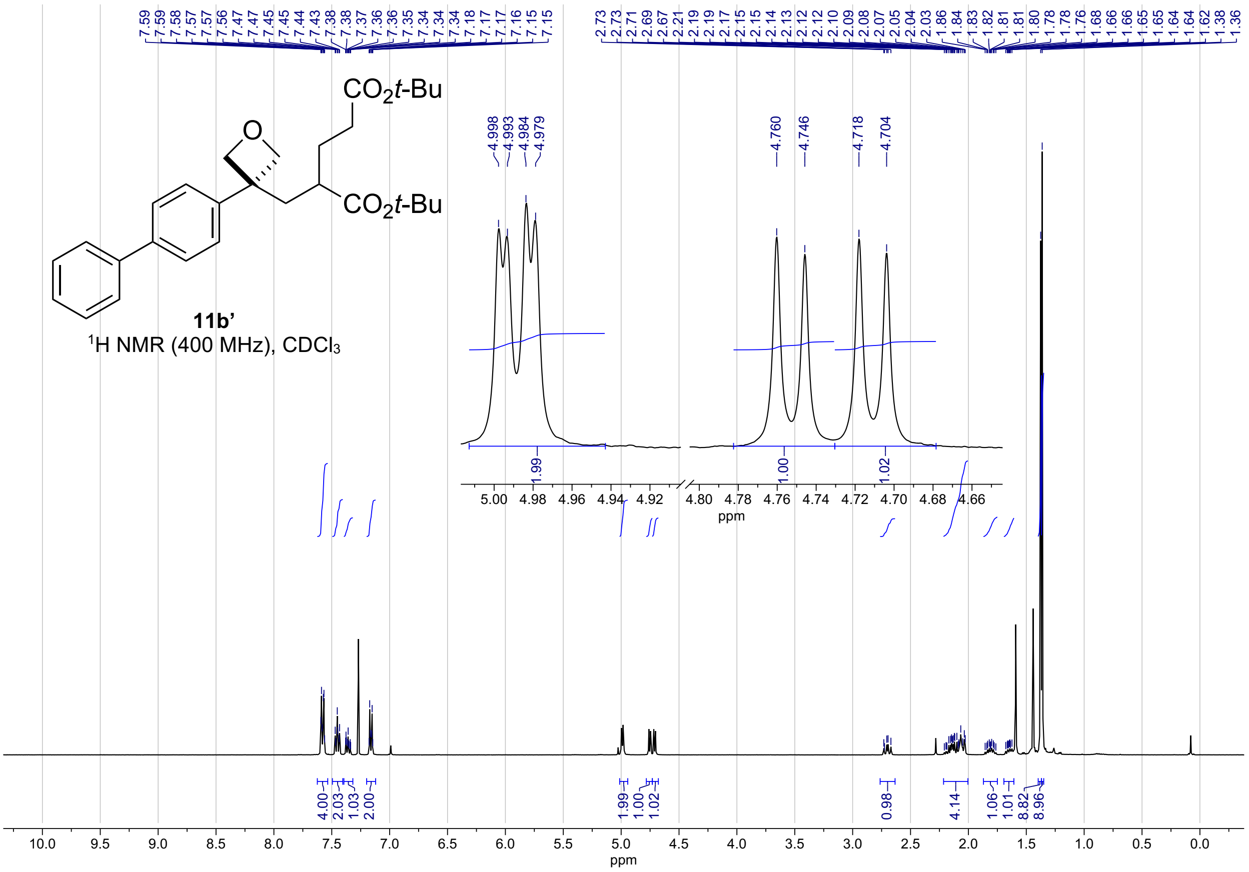
CO2t-Bu
CO2t-Bu
O
11b'
1H NMR (400 MHz), CDCl3
ppm

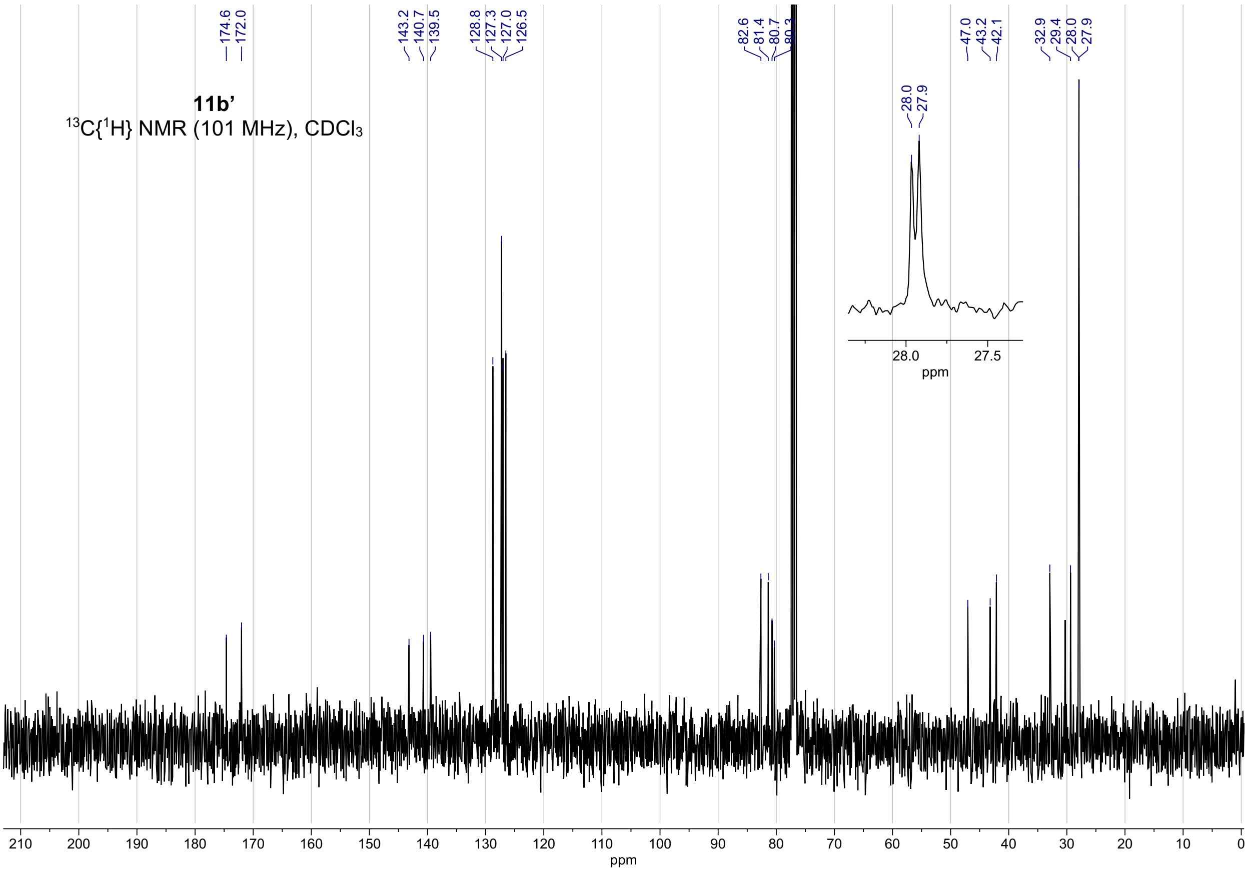
11b'
13C{1H} NMR (101 MHz), CDCl3
ppm

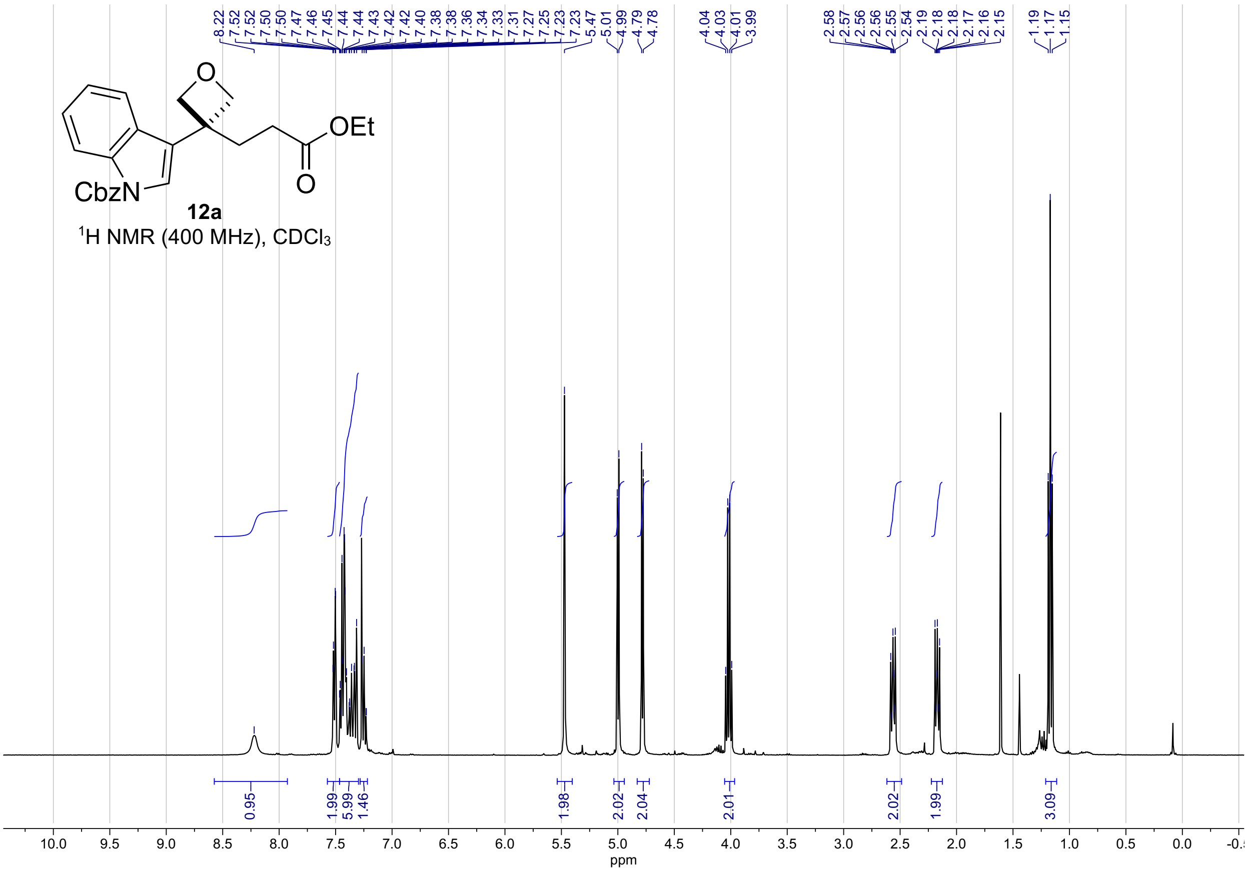
OEt
O
CbzN
12a
[1]H NMR (400 MHz), CDCl3
0.95
1.99
5.99
1.46
1.98
2.02
2.04
2.01
2.02
1.99
3.09
ppm

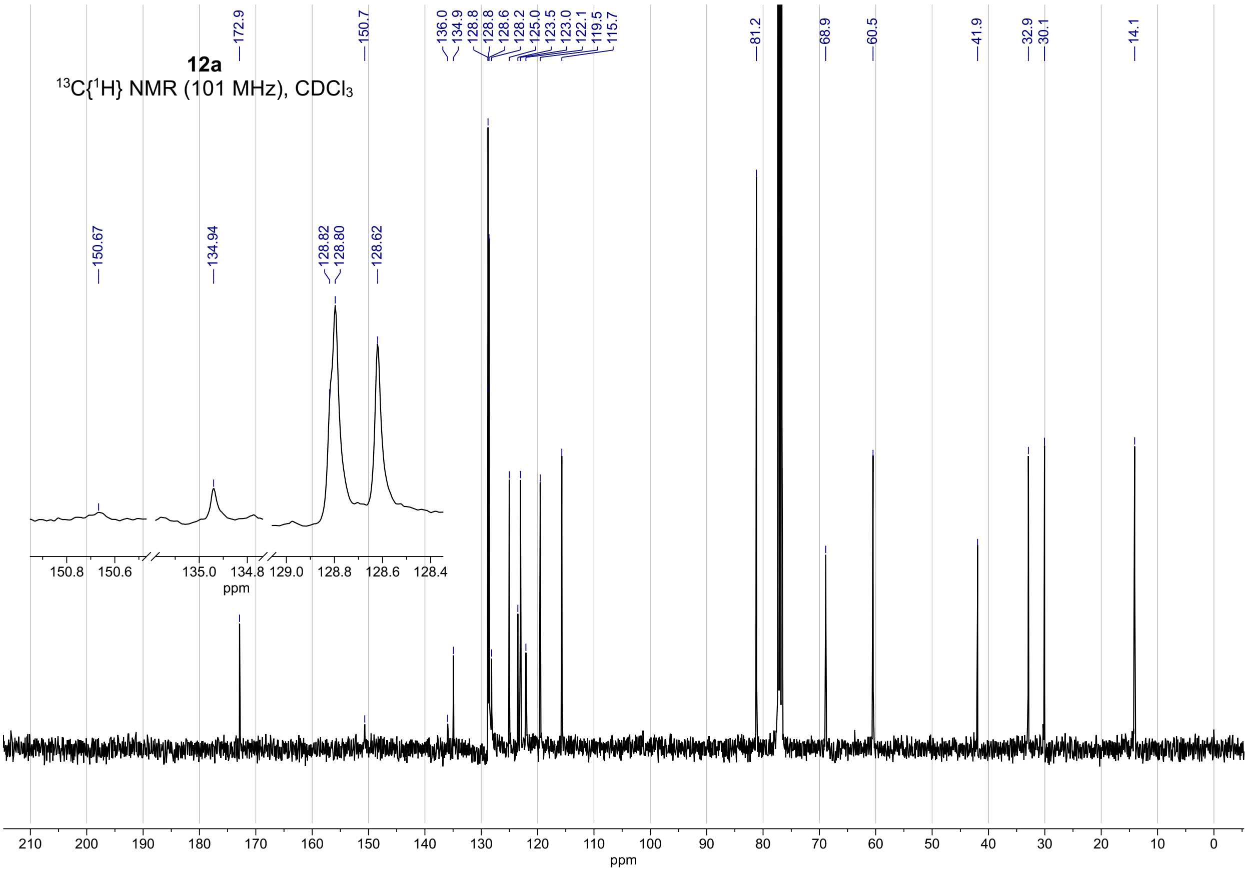
12a
[13]C{[1]H} NMR (101 MHz), CDCl3
172.9
150.7
136.0
134.9
128.8
128.8
128.6
128.2
125.0
123.5
123.0
122.1
119.5
115.7
81.2
68.9
60.5
41.9
32.9
30.1
14.1
150.67
134.94
128.82
128.80
128.62
ppm

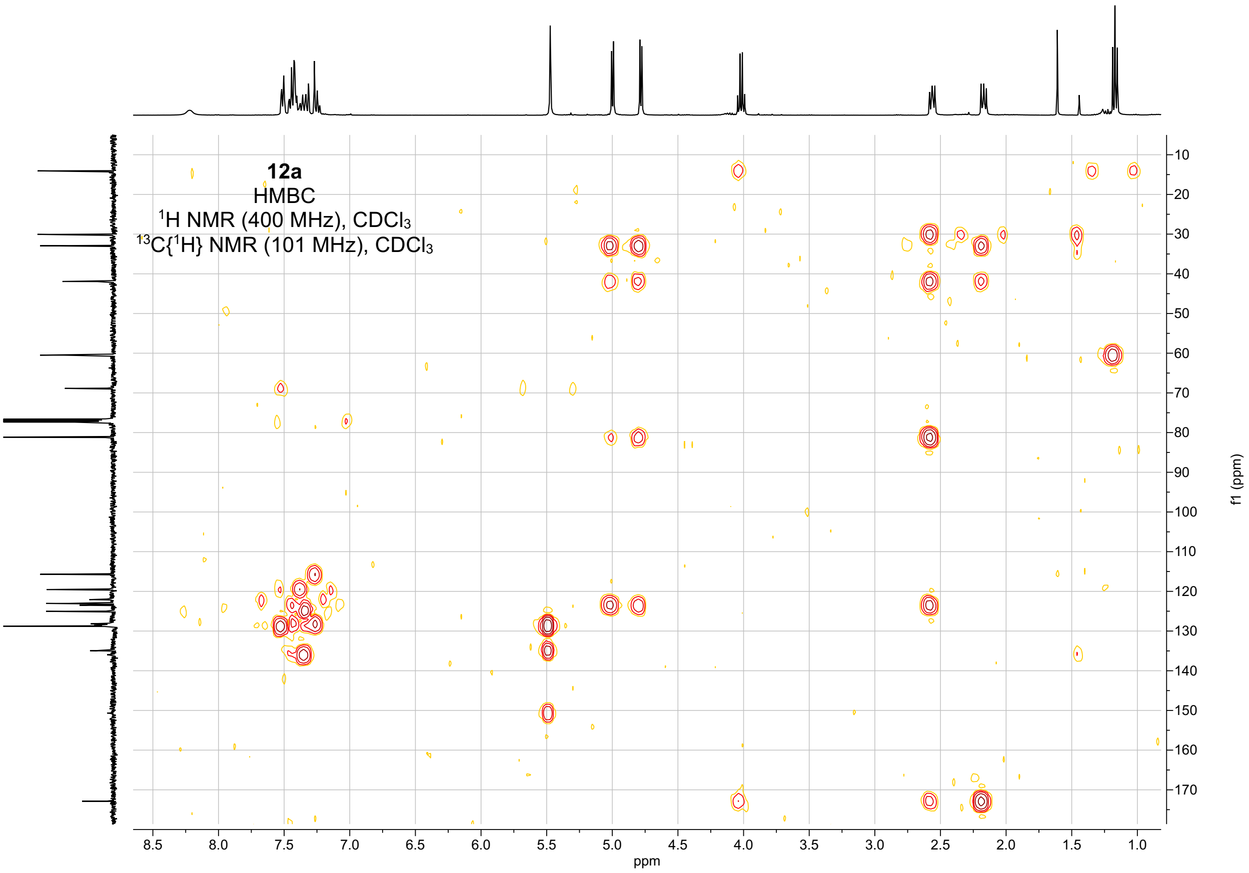

**12a**
HMBC
$^1$H NMR (400 MHz), CDCl$_3$
$^{13}$C{$^1$H} NMR (101 MHz), CDCl$_3$
8.5 8.0 7.5 7.0 6.5 6.0 5.5 5.0 4.5 4.0 3.5 3.0 2.5 2.0 1.5 1.0
ppm
10 20 30 40 50 60 70 80 90 100 110 120 130 140 150 160 170
f1 (ppm)

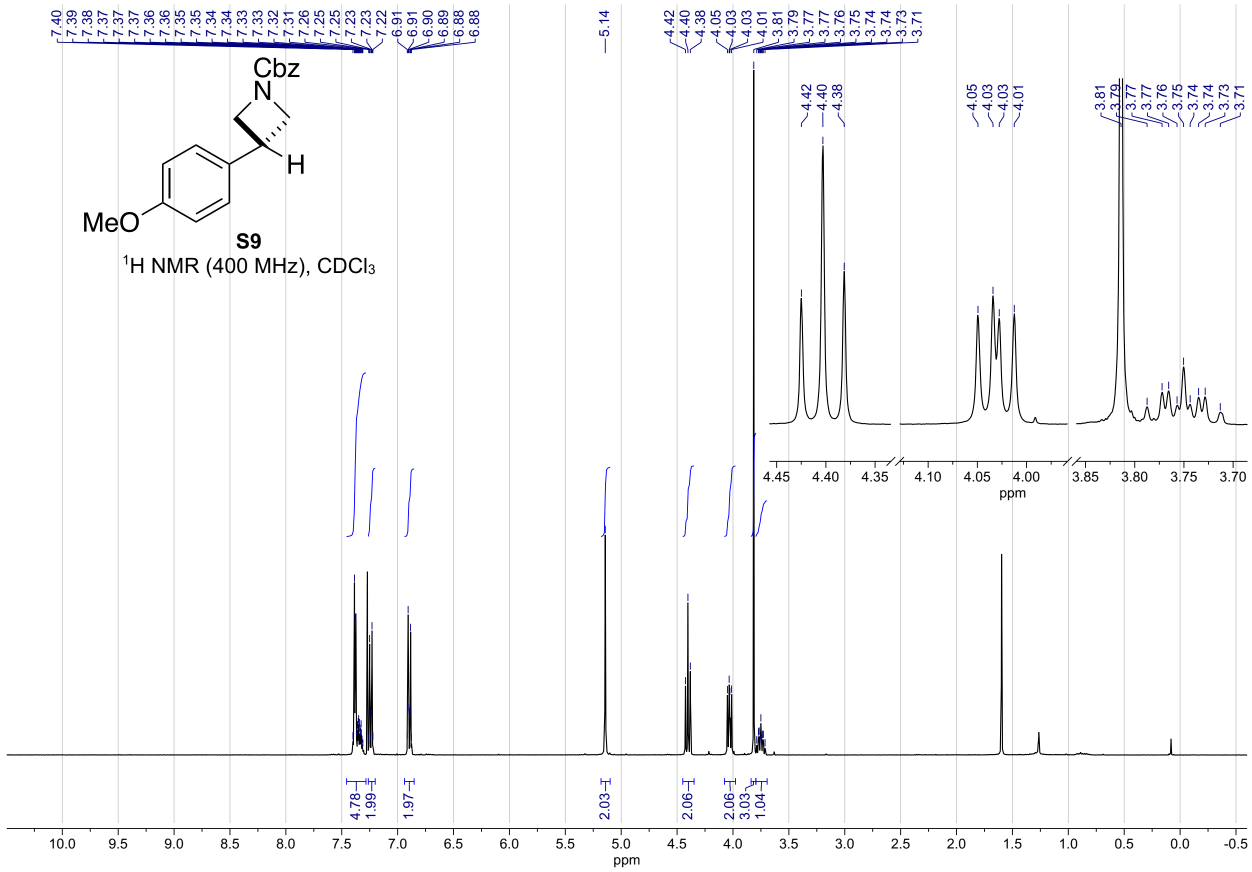
Cbz
N
H
MeO
S9
$^1$H NMR (400 MHz), CDCl$_3$
7.40 7.39 7.38 7.37 7.37 7.36 7.36 7.35 7.35 7.34 7.34 7.33 7.33 7.32 7.31 7.26 7.25 7.25 7.25 7.23 7.23 7.22 6.91 6.91 6.90 6.89 6.88 6.88
5.14
4.42 4.40 4.38 4.05 4.03 4.03 4.01 3.81 3.79 3.77 3.76 3.75 3.74 3.74 3.73 3.71
4.78 1.99 1.97 2.03 2.06 2.06 3.03 1.04
ppm

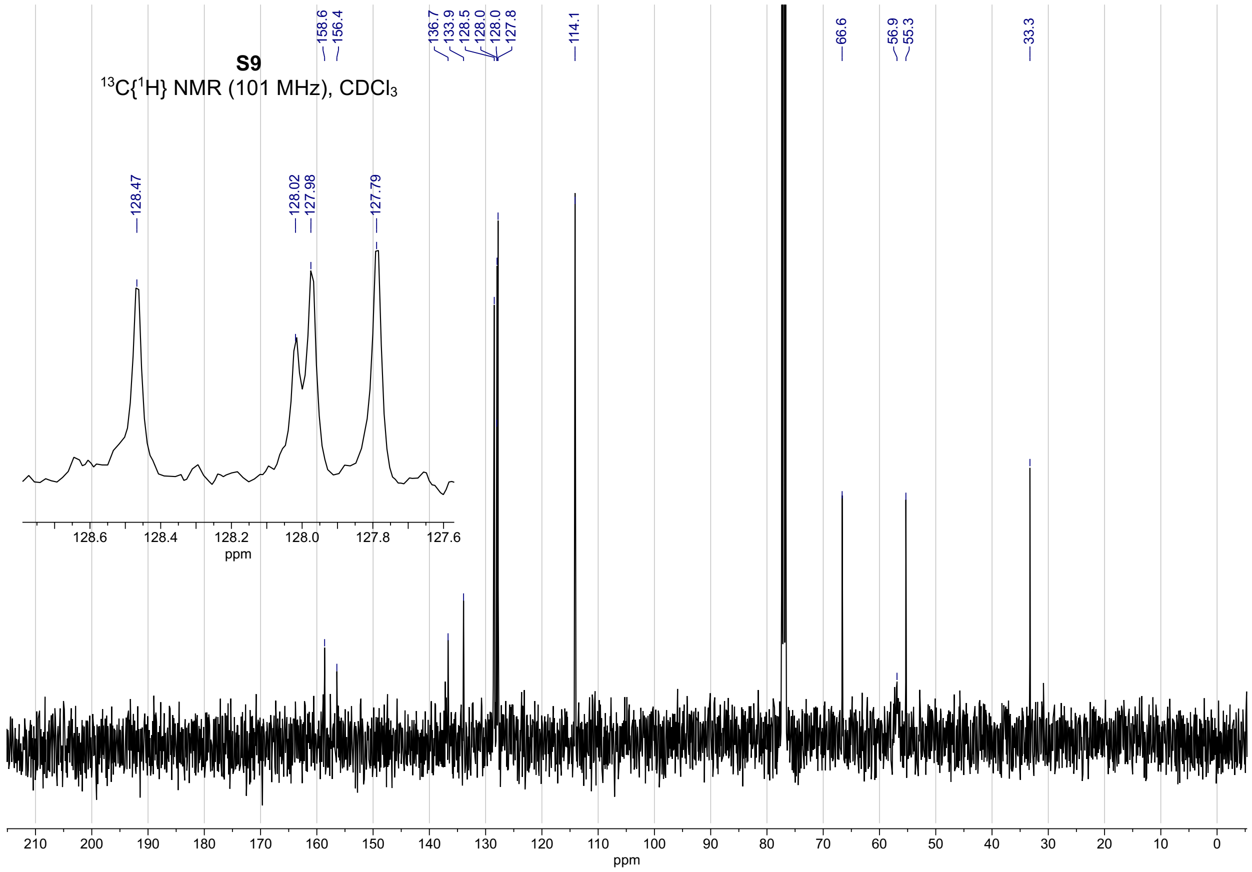
S9
$^{13}$C{$^1$H} NMR (101 MHz), CDCl$_3$
158.6 156.4 136.7 133.9 128.5 128.0 128.0 127.8 114.1 66.6 56.9 55.3 33.3
128.47 128.02 127.98 127.79
ppm

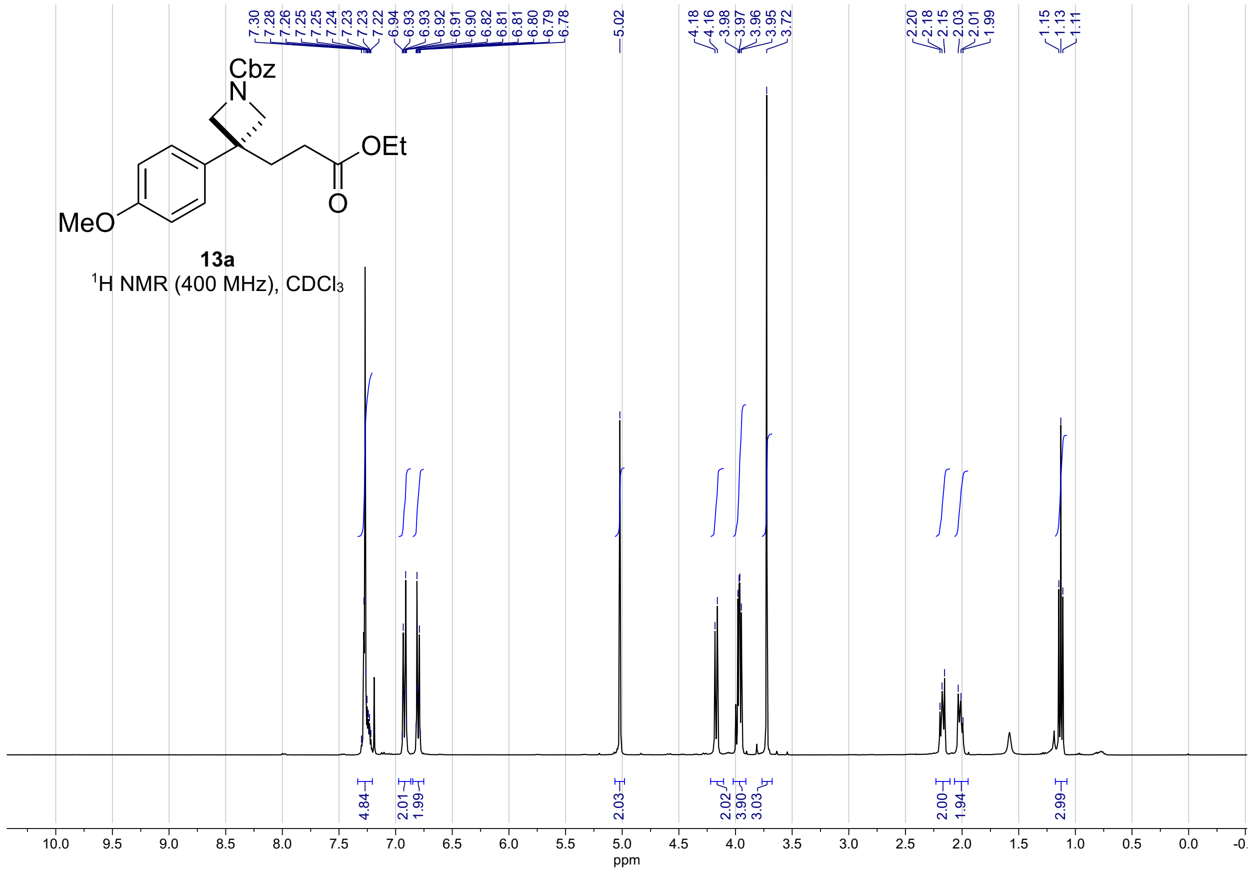
Cbz
N
OEt
O
MeO
13a
1H NMR (400 MHz), CDCl3
7.30 7.28 7.26 7.25 7.25 7.24 7.23 7.23 7.22 6.94 6.93 6.93 6.92 6.91 6.90 6.82 6.81 6.81 6.80 6.79 6.78
5.02
4.18 4.16 3.98 3.97 3.96 3.95 3.72
2.20 2.18 2.15 2.03 2.01 1.99
1.15 1.13 1.11
4.84 2.01 1.99 2.03 2.02 3.90 3.03 2.00 1.94 2.99
ppm

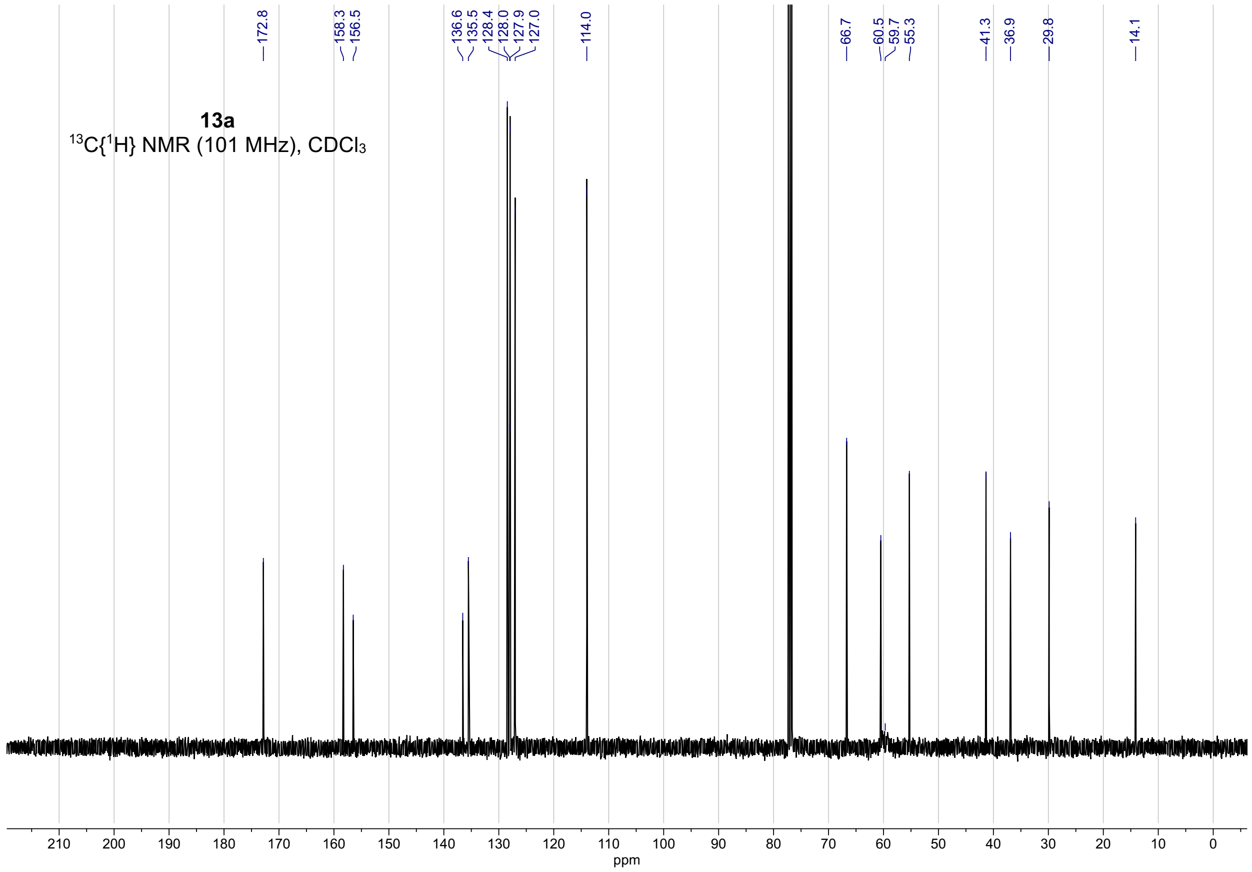
172.8
158.3 156.5
136.6 135.5 128.4 128.0 127.9 127.0
114.0
66.7 60.5 59.7 55.3
41.3 36.9 29.8 14.1
13a
13C{1H} NMR (101 MHz), CDCl3
ppm

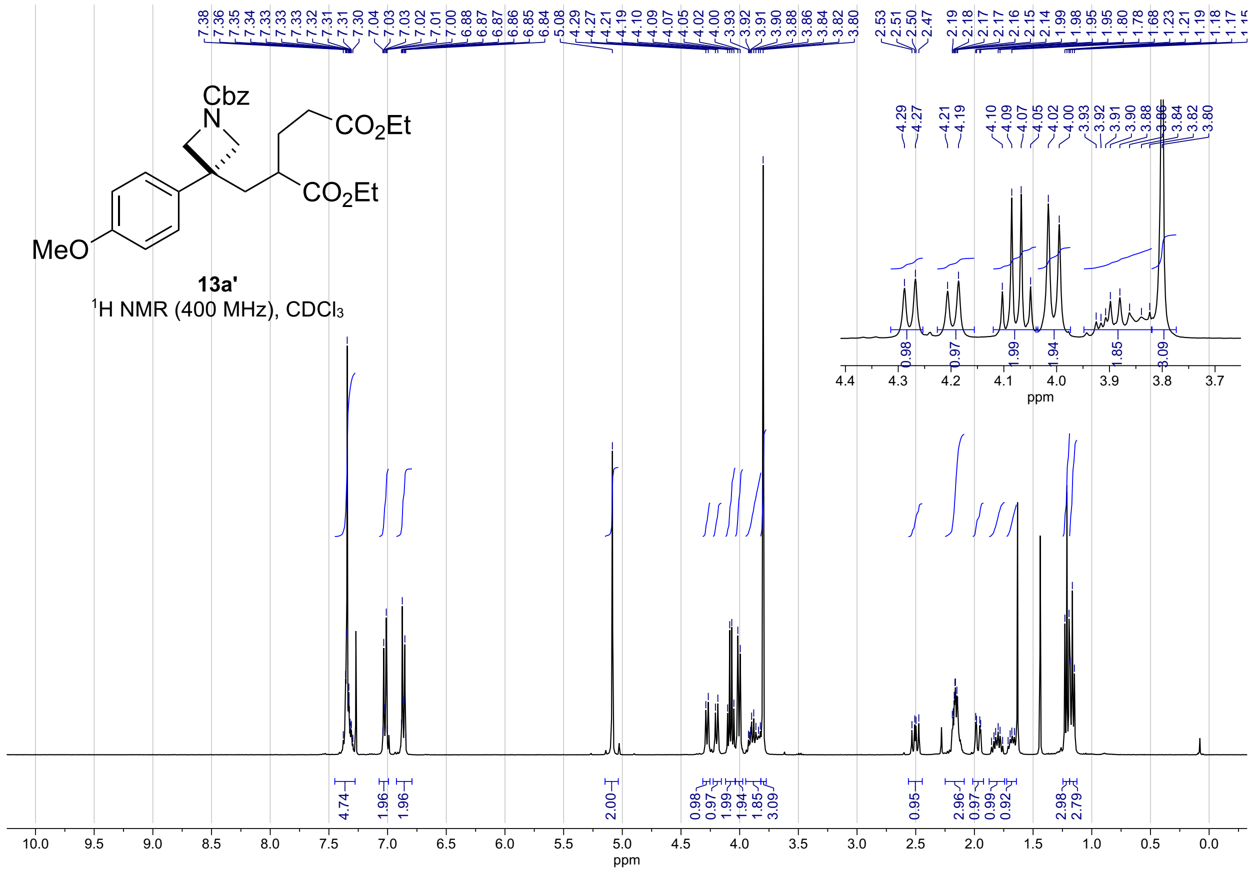
13a'
$^1$H NMR (400 MHz), $CDCl_3$

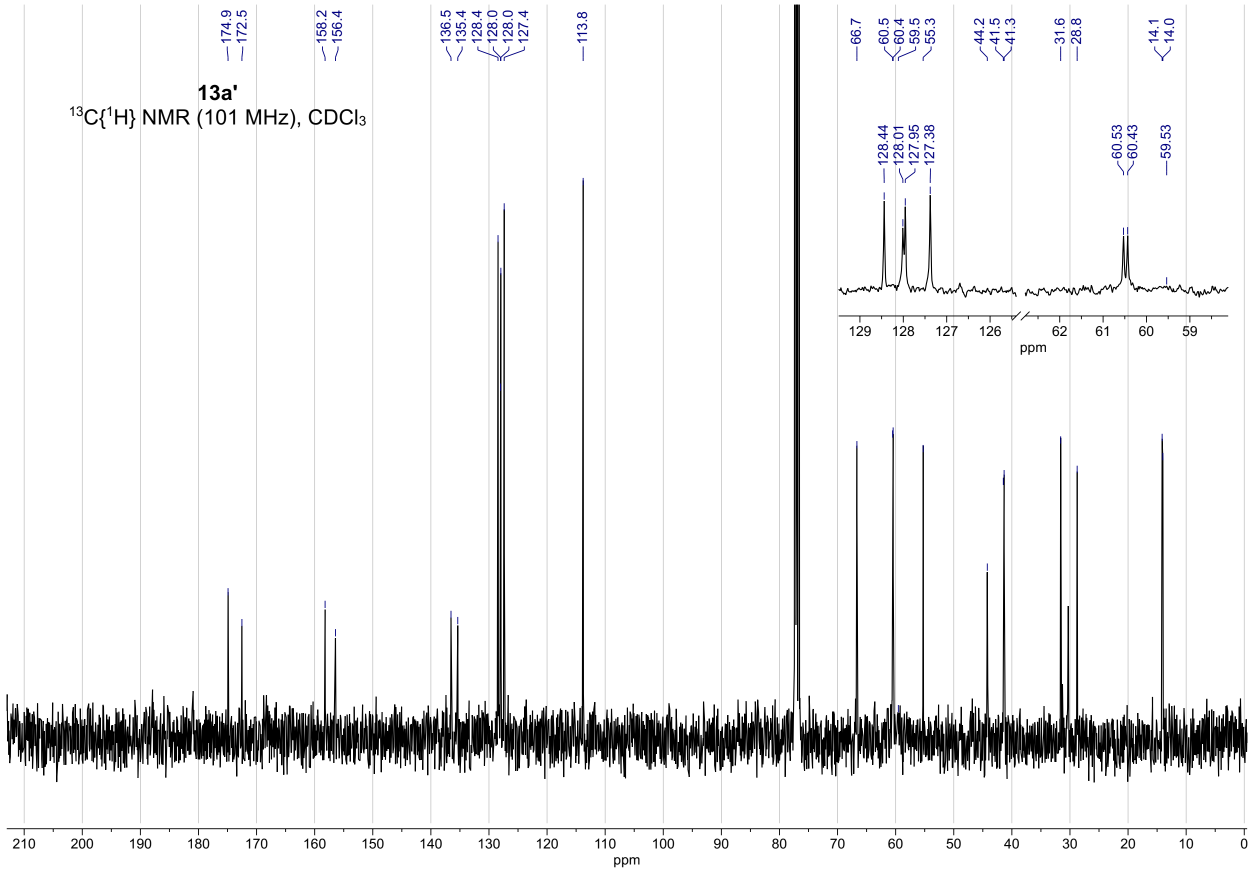
13a'
$^{13}C\{^1H\}$ NMR (101 MHz), $CDCl_3$

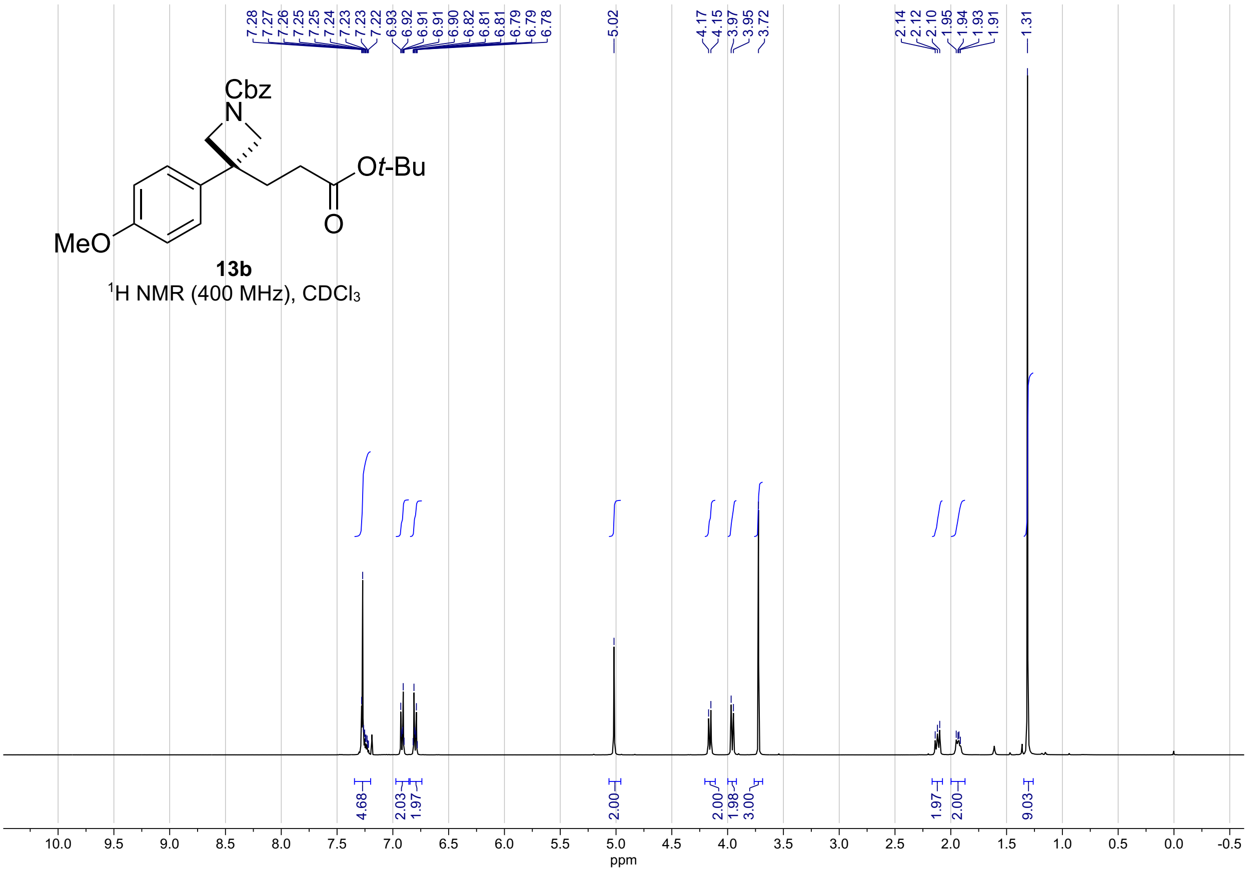
Cbz
N
O*t*-Bu
O
MeO
**13b**
$^1$H NMR (400 MHz), CDCl$_3$
ppm

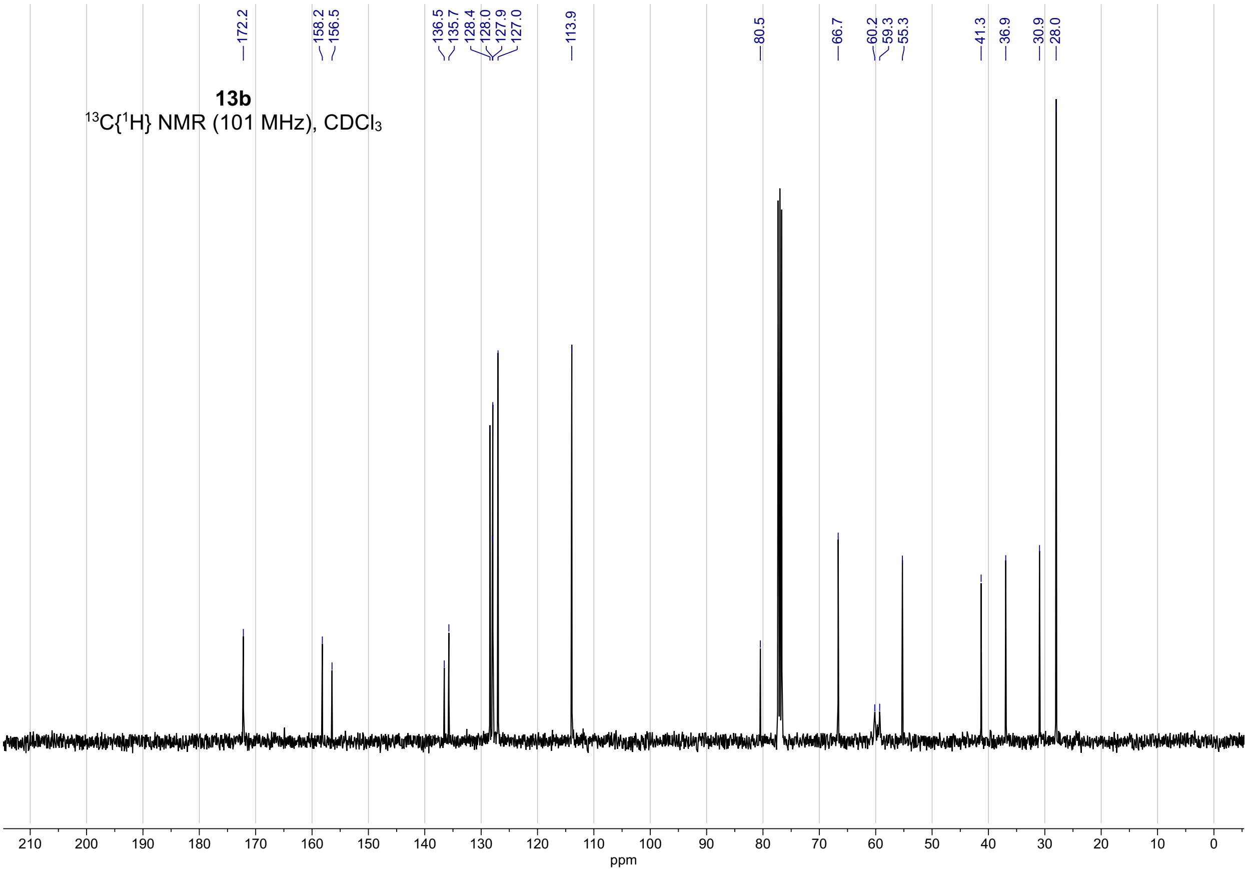
**13b**
$^{13}$C{$^1$H} NMR (101 MHz), CDCl$_3$
ppm

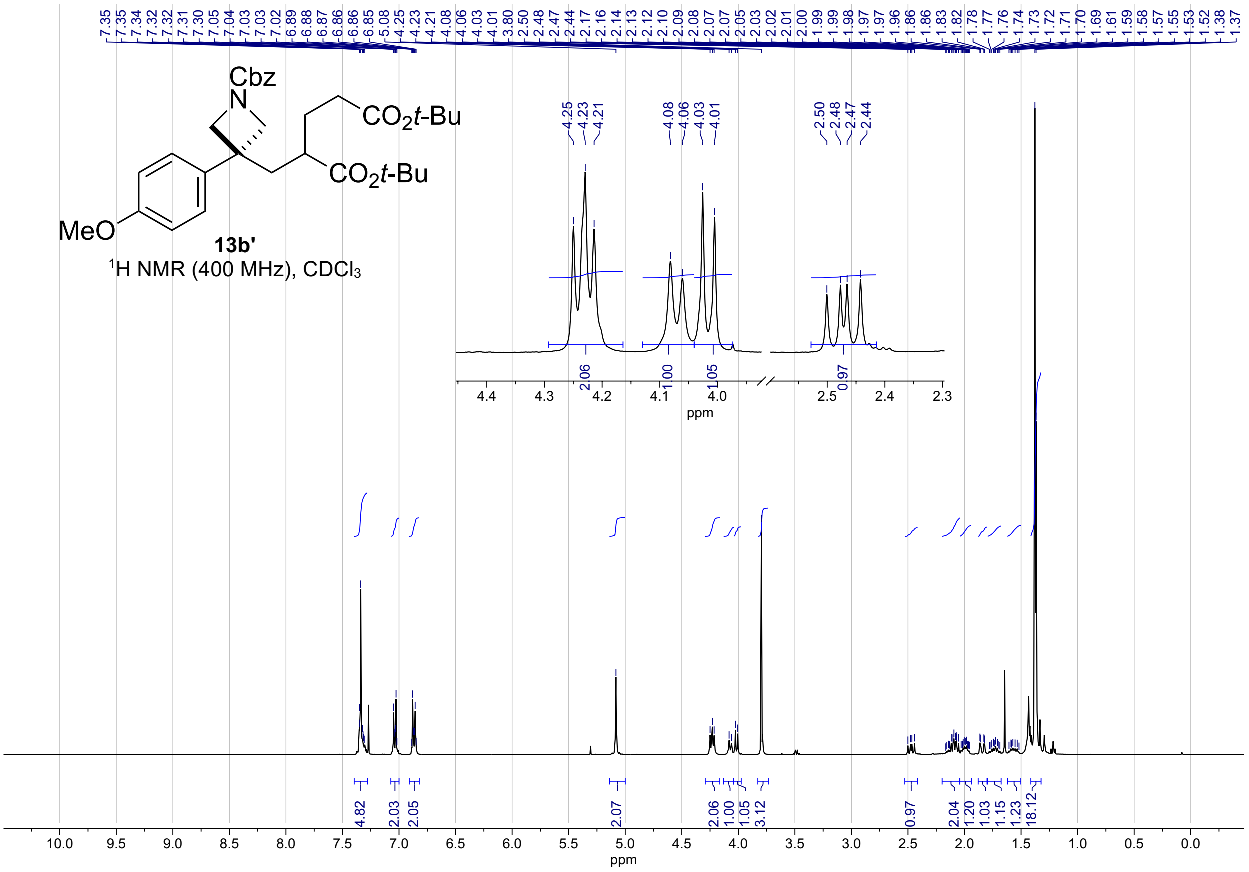
Cbz
N
$CO_2t$-Bu
$CO_2t$-Bu
MeO
13b'
$^1$H NMR (400 MHz), $CDCl_3$
ppm

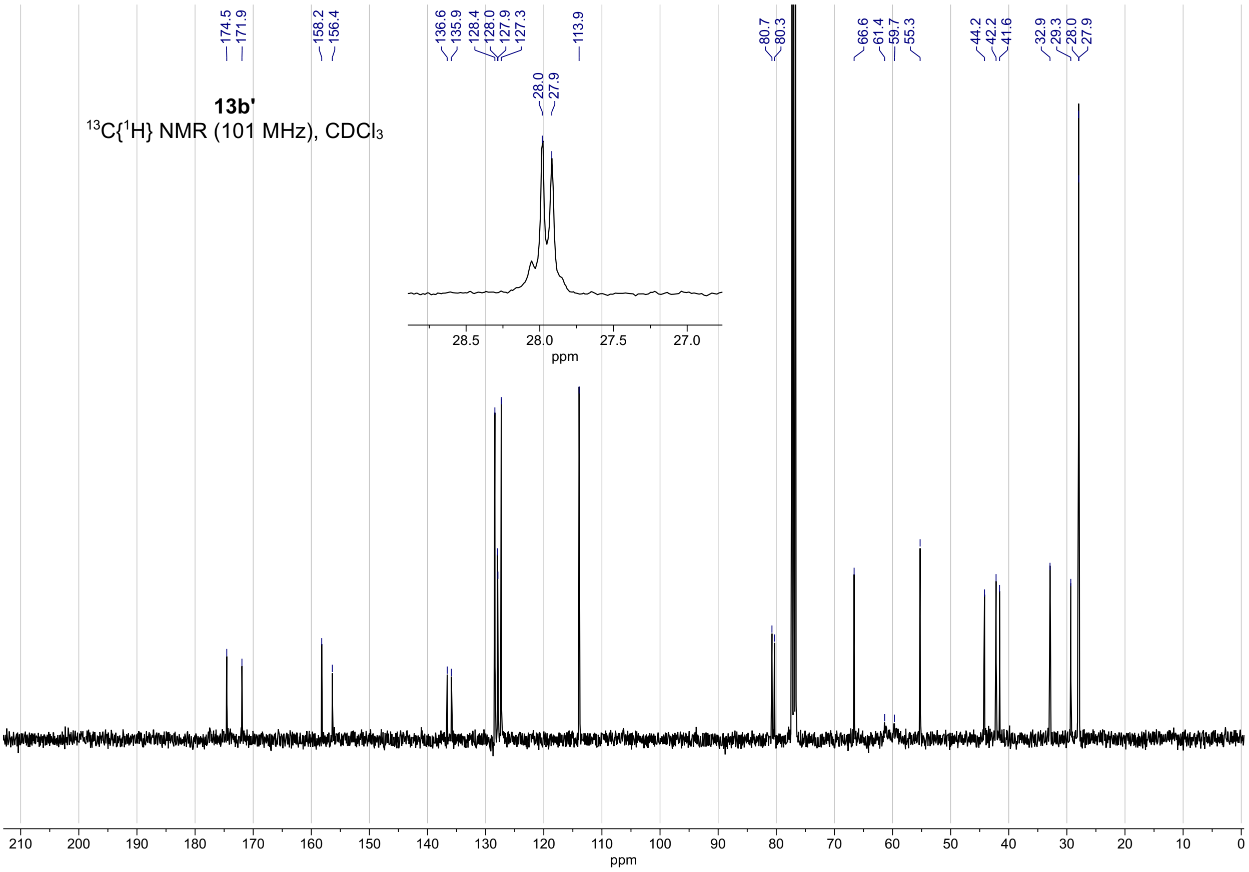
13b'
$^{13}C\{^1H\}$ NMR (101 MHz), $CDCl_3$
ppm

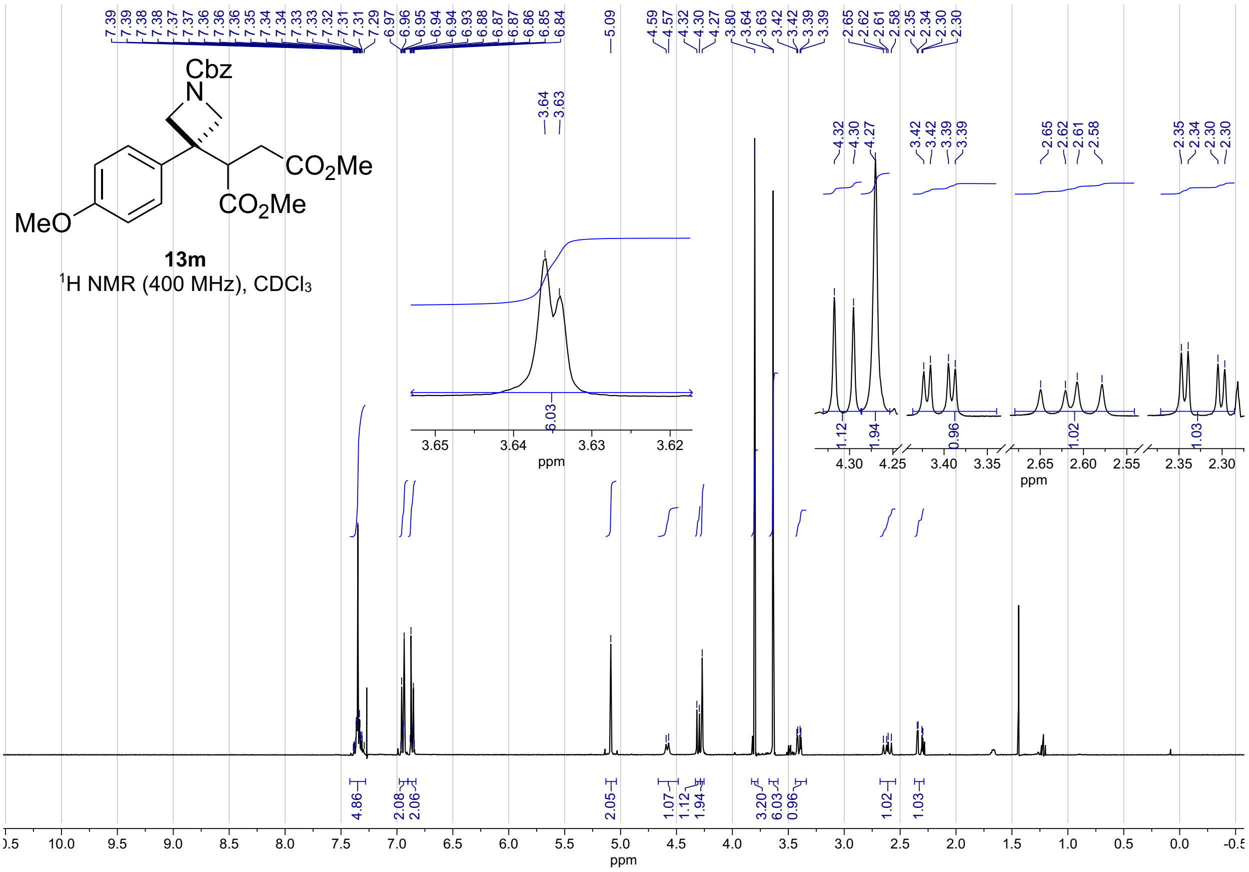
Cbz
N
CO2Me
CO2Me
MeO
13m
1H NMR (400 MHz), CDCl3
ppm

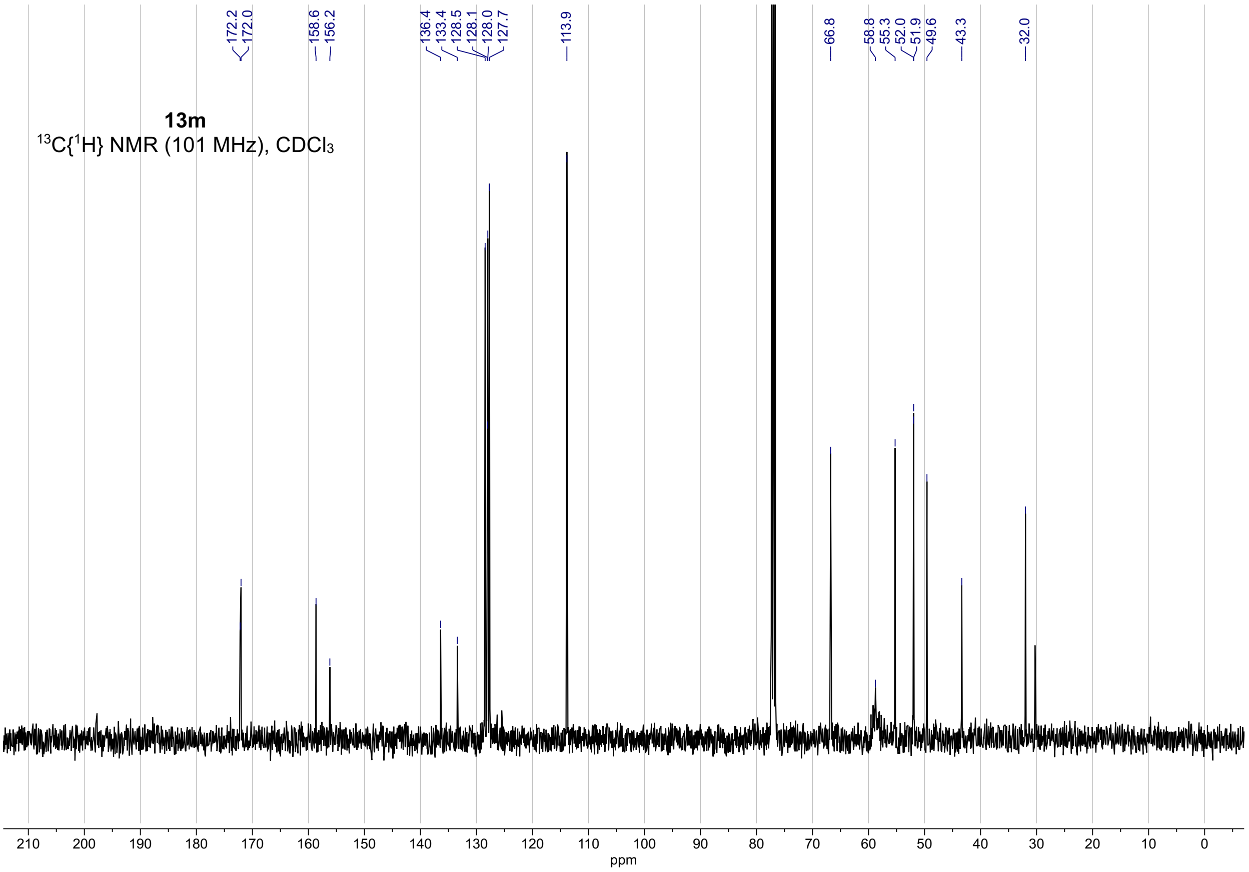
172.2
172.0
158.6
156.2
136.4
133.4
128.5
128.1
128.0
127.7
113.9
66.8
58.8
55.3
52.0
51.9
49.6
43.3
32.0
13m
13C{1H} NMR (101 MHz), CDCl3
ppm

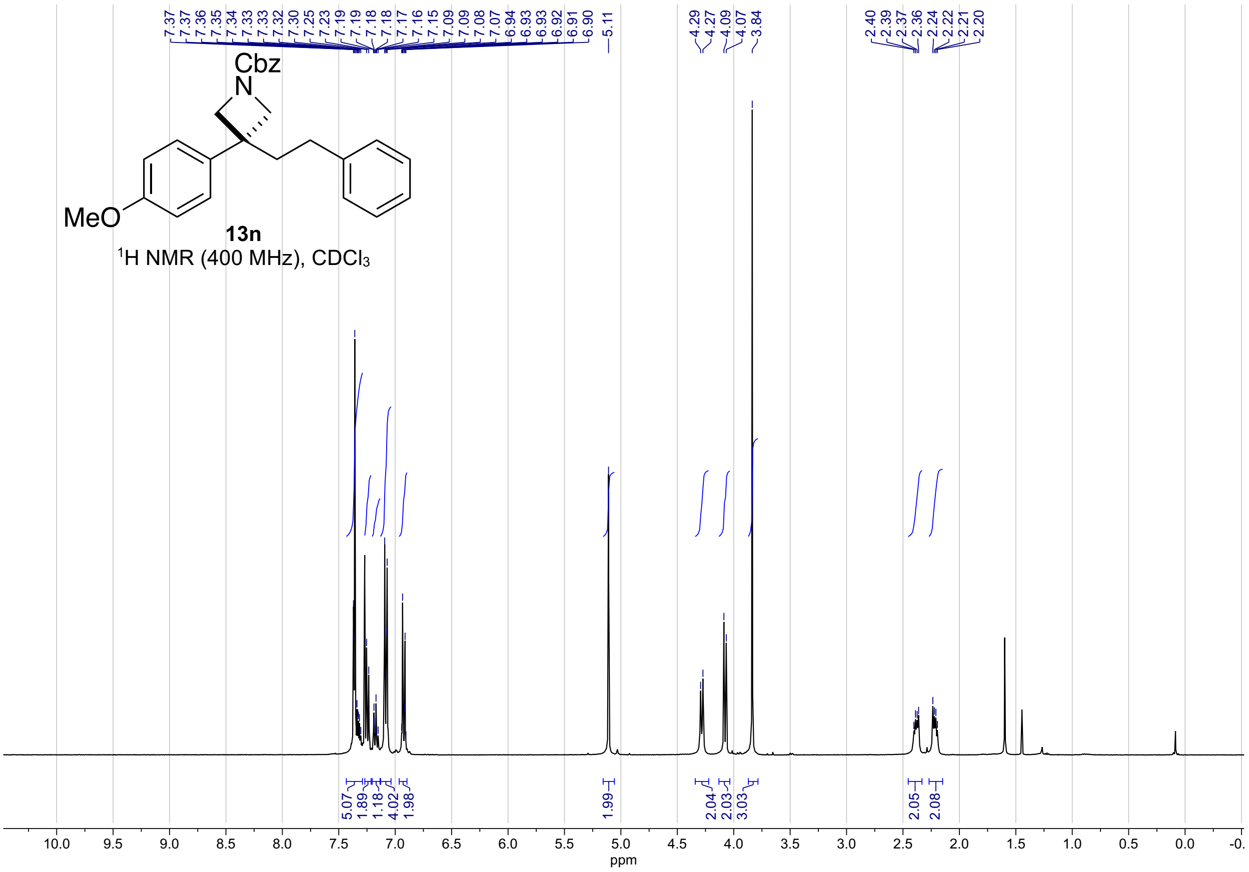

Cbz
N
MeO
13n
[1]H NMR (400 MHz), CDCl3
ppm


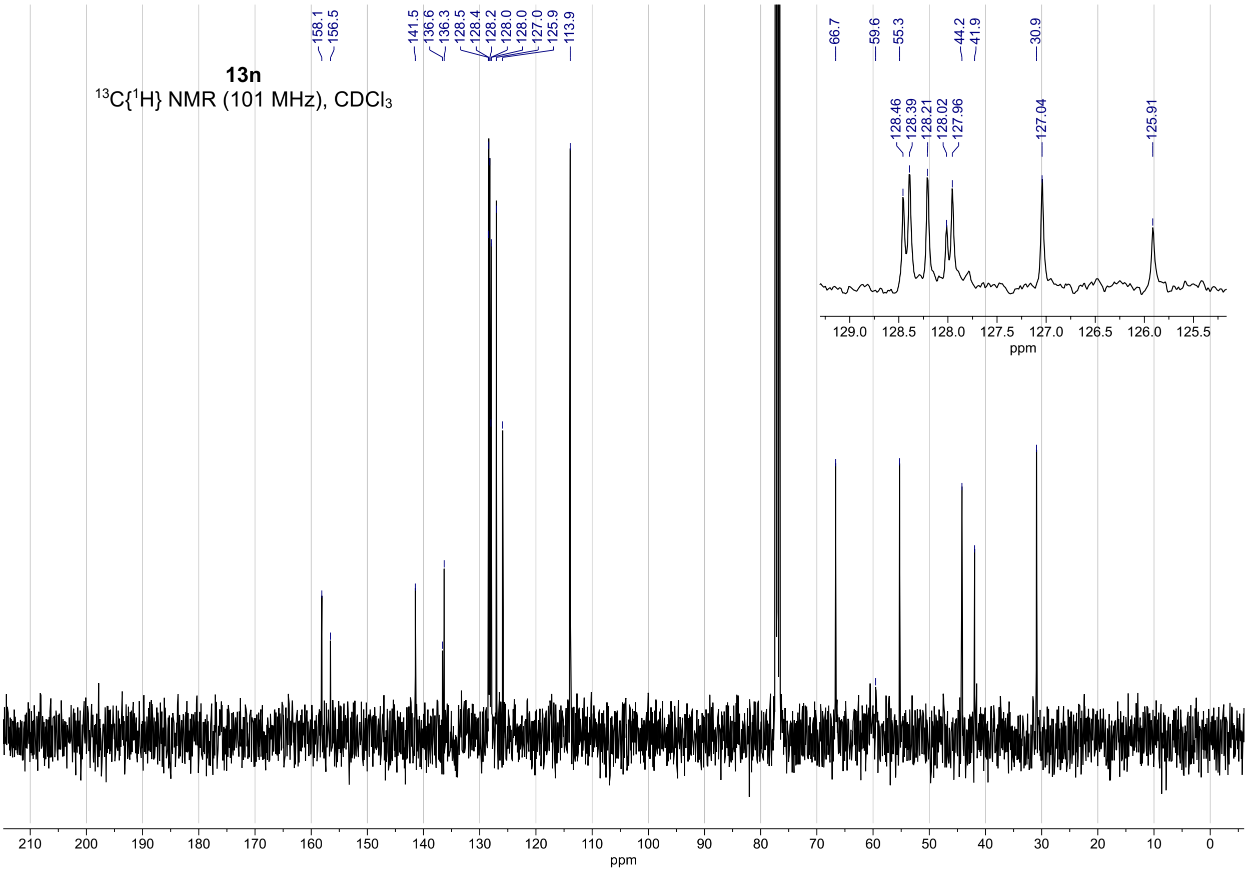

13n
[13]C{[1]H} NMR (101 MHz), CDCl3
ppm

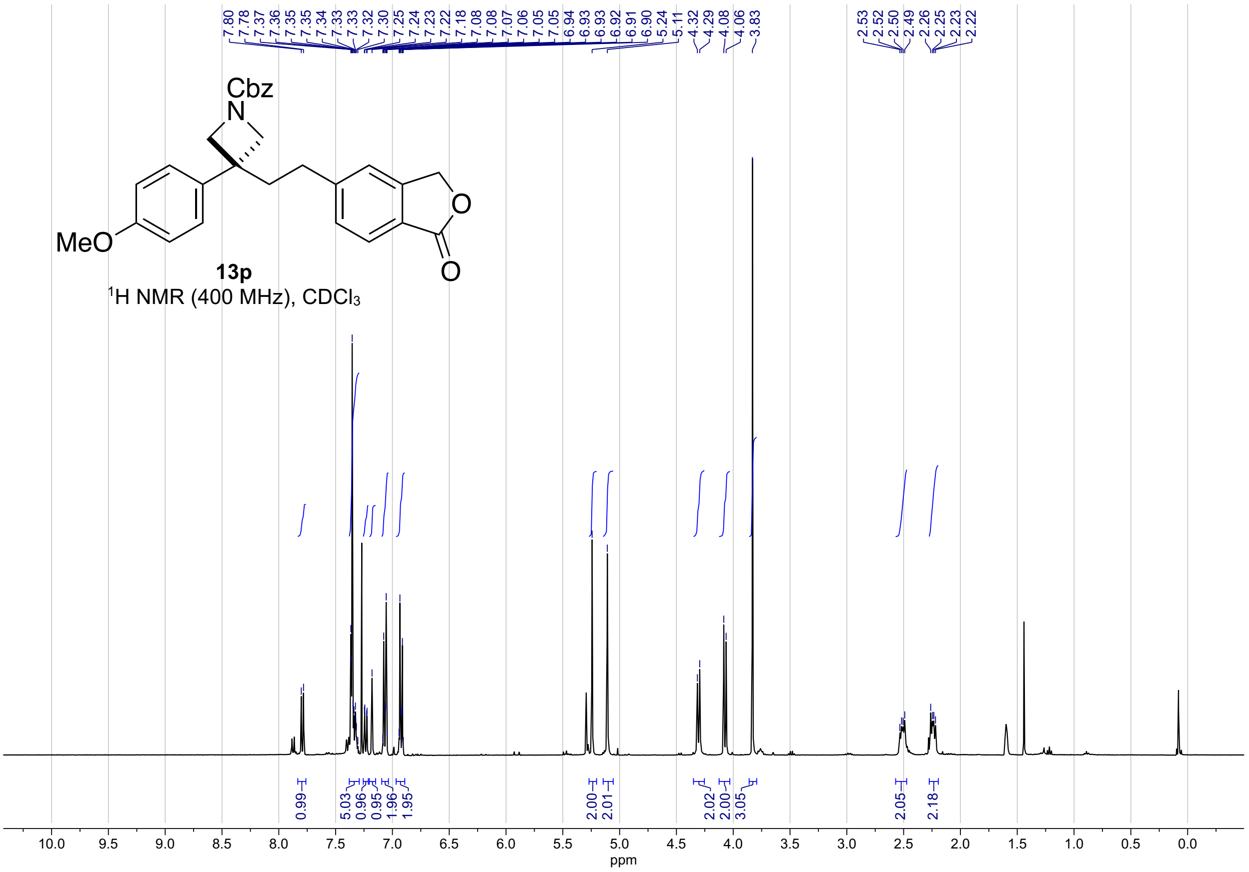
Cbz
N
O
O
MeO
13p
[1]H NMR (400 MHz), CDCl3
ppm

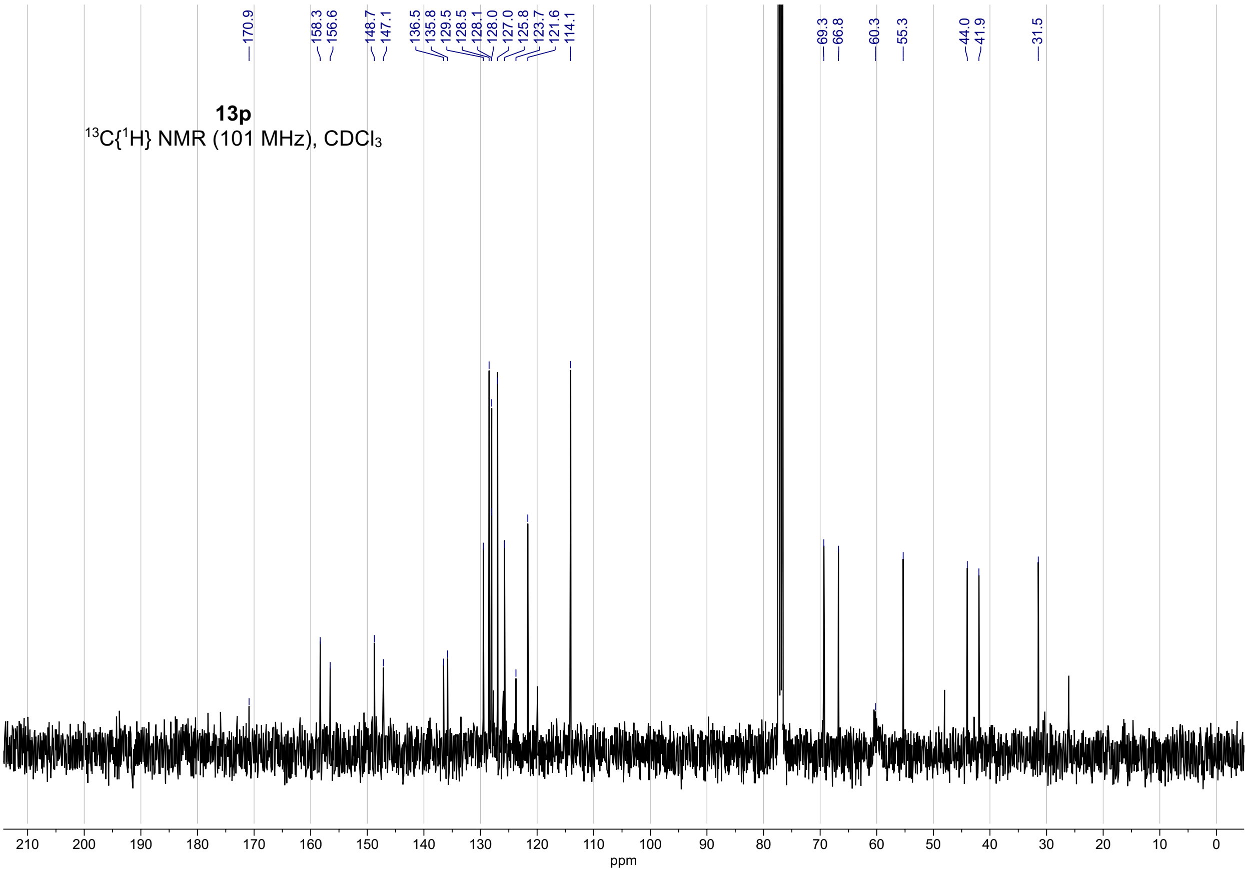
13p
[13]C{[1]H} NMR (101 MHz), CDCl3
ppm

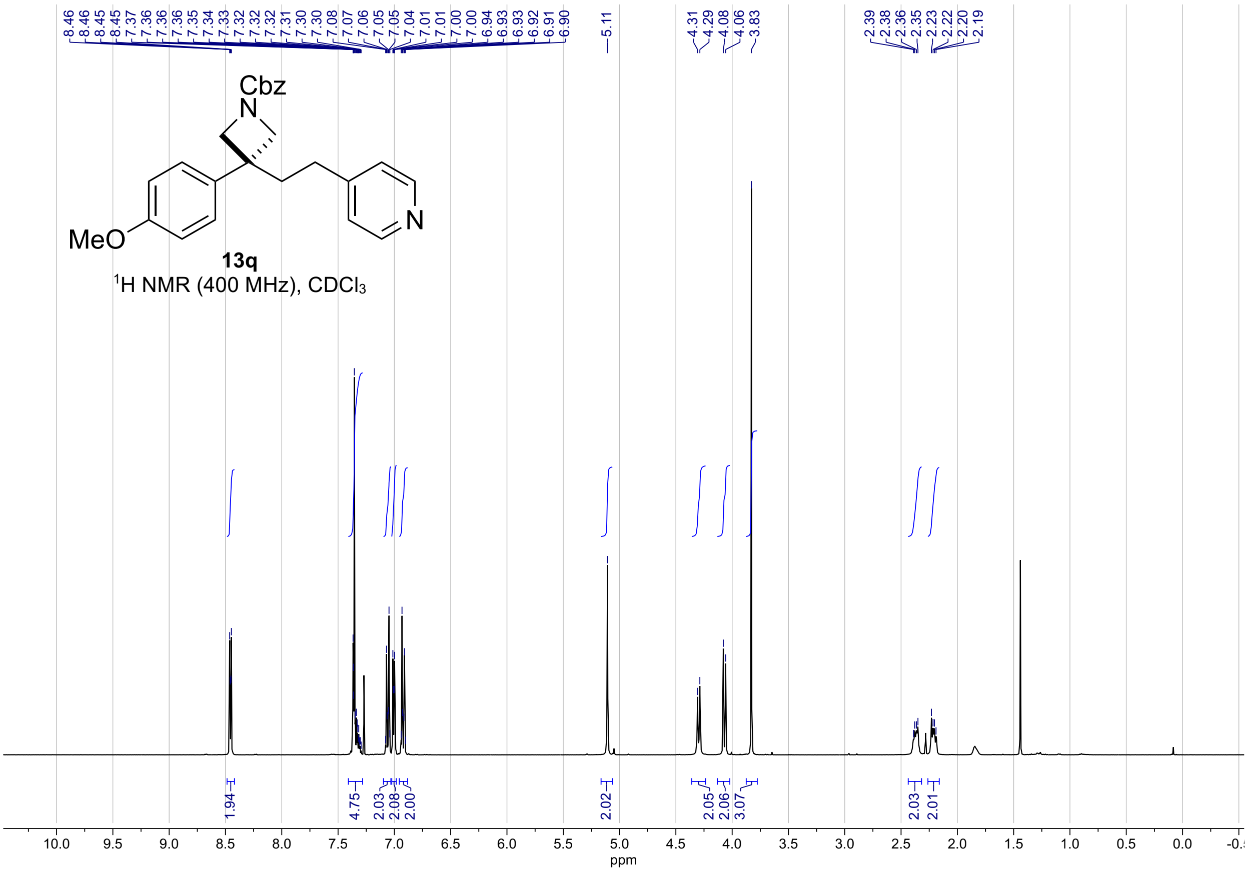


**13q**

$^1$H NMR (400 MHz), CDCl$_3$

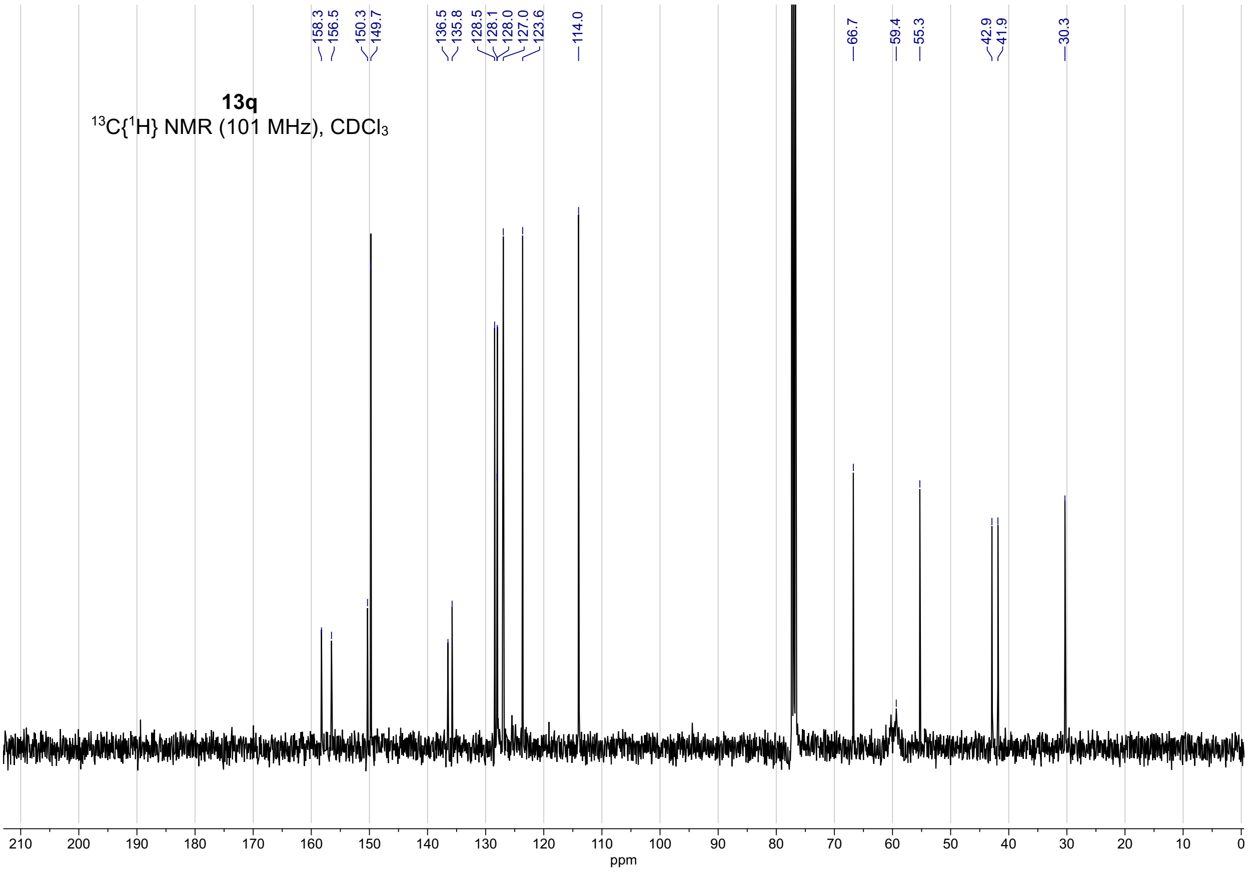


**13q**

$^{13}$C{$^1$H} NMR (101 MHz), CDCl$_3$

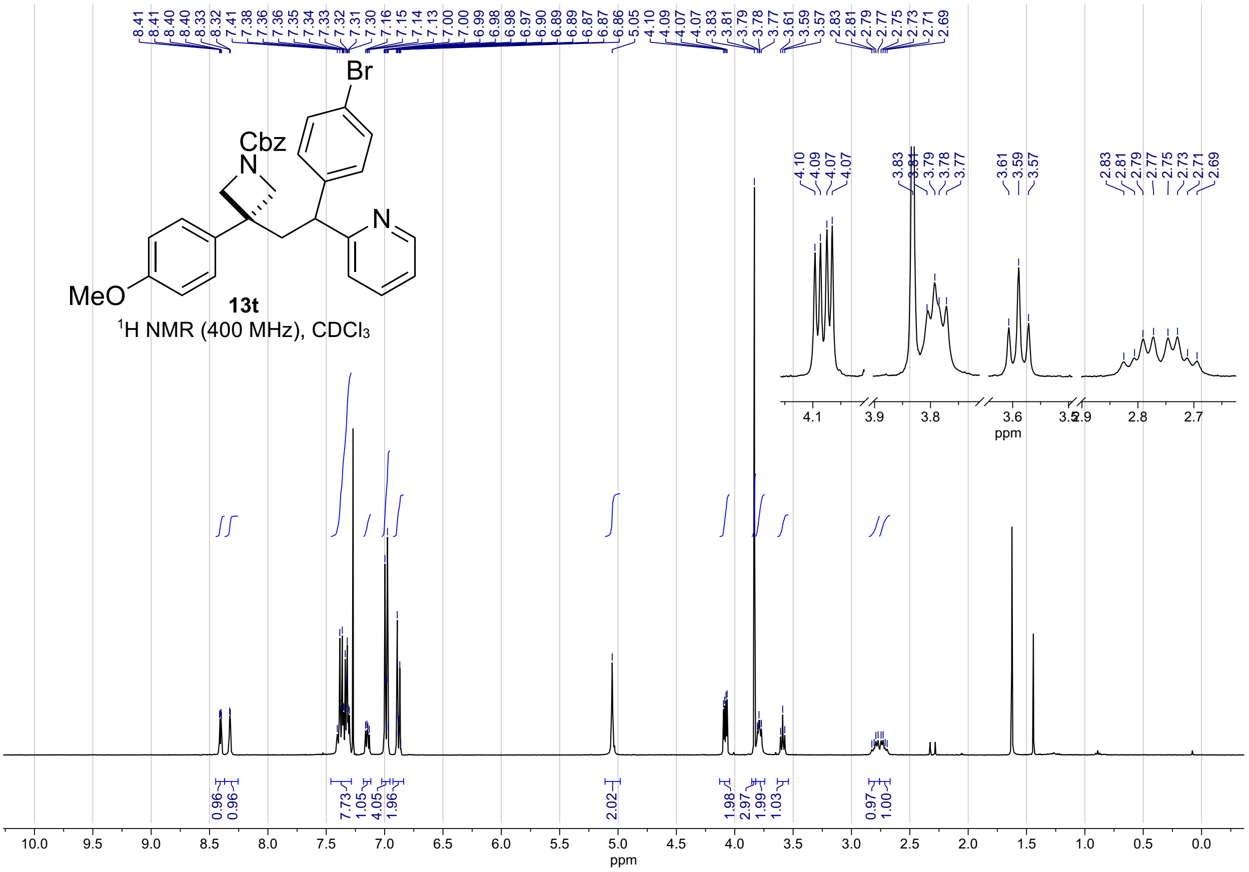

Br
Cbz
N
N
MeO
13t
1H NMR (400 MHz), CDCl3
ppm


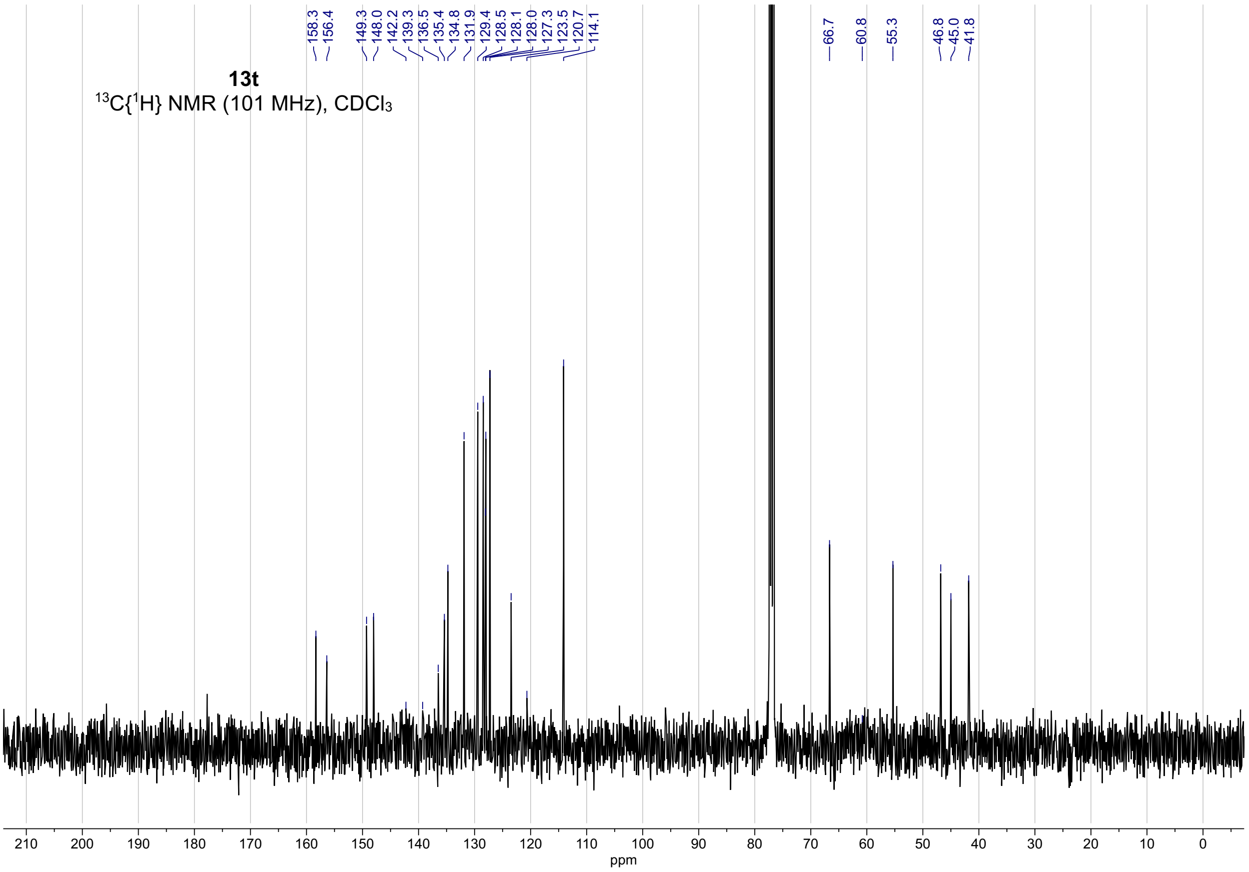

158.3
156.4
149.3
148.0
142.2
139.3
136.5
135.4
134.8
131.9
129.4
128.5
128.1
128.0
127.3
123.5
120.7
114.1
66.7
60.8
55.3
46.8
45.0
41.8
13t
13C{1H} NMR (101 MHz), CDCl3
ppm

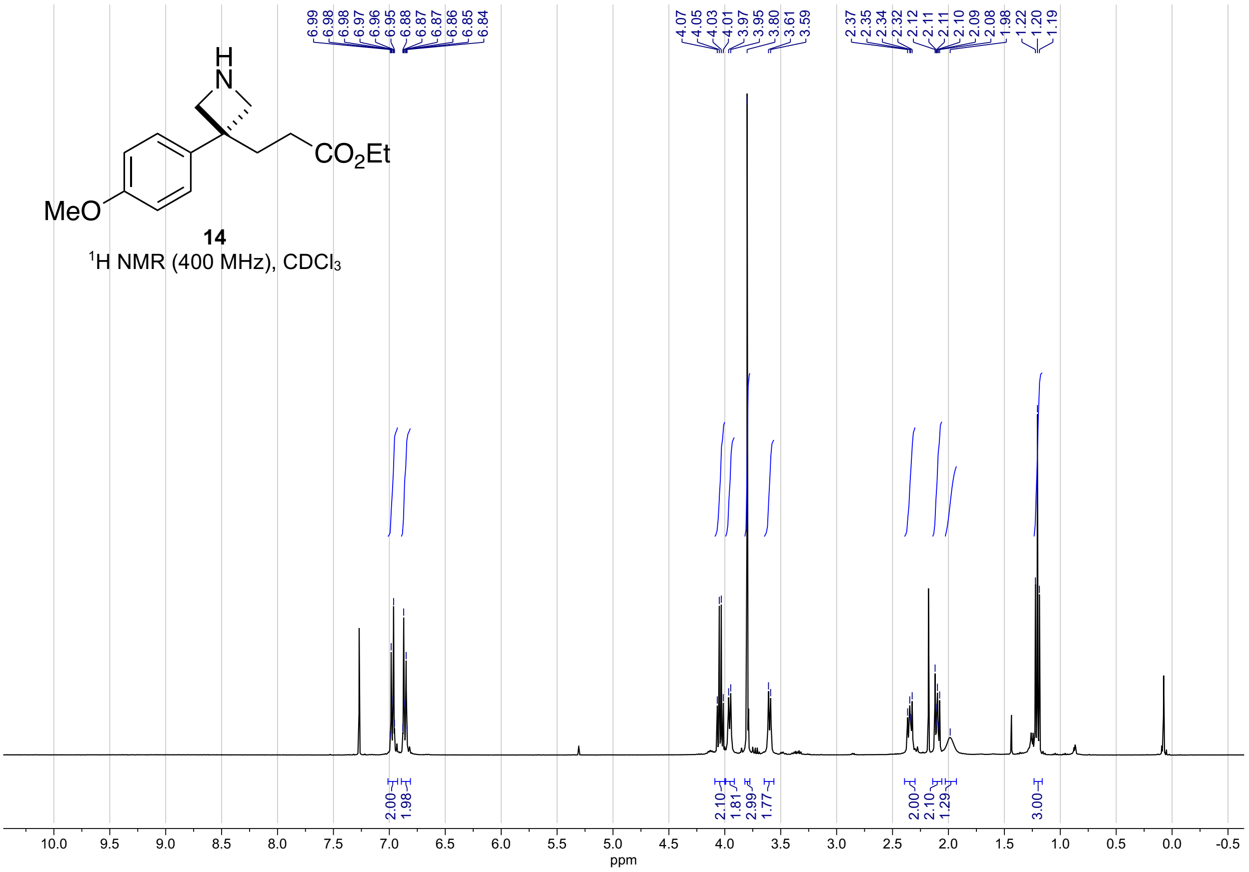

H
N
CO2Et
MeO
14
1H NMR (400 MHz), CDCl3
6.99 6.98 6.98 6.97 6.96 6.95 6.88 6.87 6.87 6.86 6.85 6.84
4.07 4.05 4.03 4.01 3.97 3.95 3.80 3.61 3.59
2.37 2.35 2.34 2.32 2.12 2.11 2.11 2.10 2.09 2.08 1.98 1.22 1.20 1.19
2.00 1.98 2.10 1.81 2.99 1.77 2.00 2.10 1.29 3.00
ppm


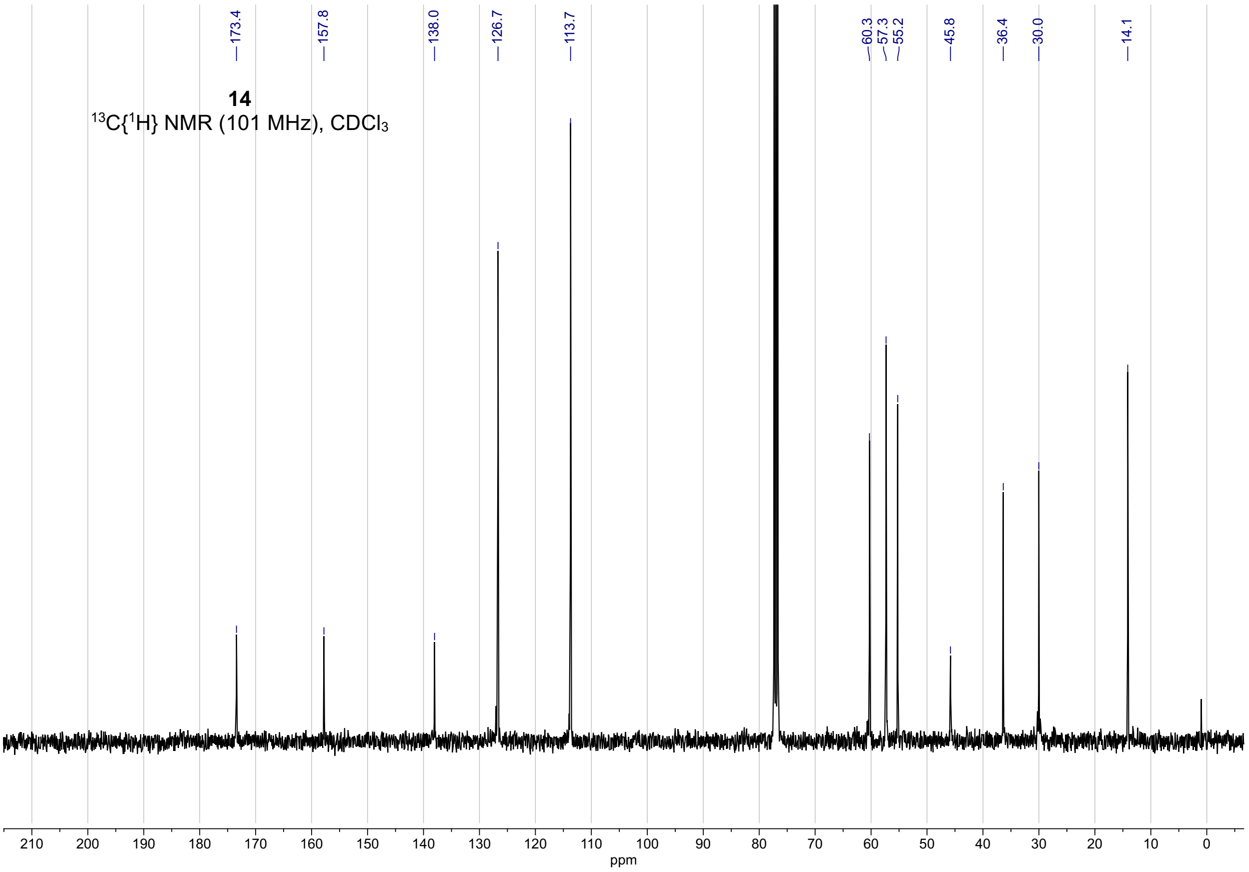

14
13C{1H} NMR (101 MHz), CDCl3
173.4
157.8
138.0
126.7
113.7
60.3 57.3 55.2
45.8
36.4
30.0
14.1
ppm

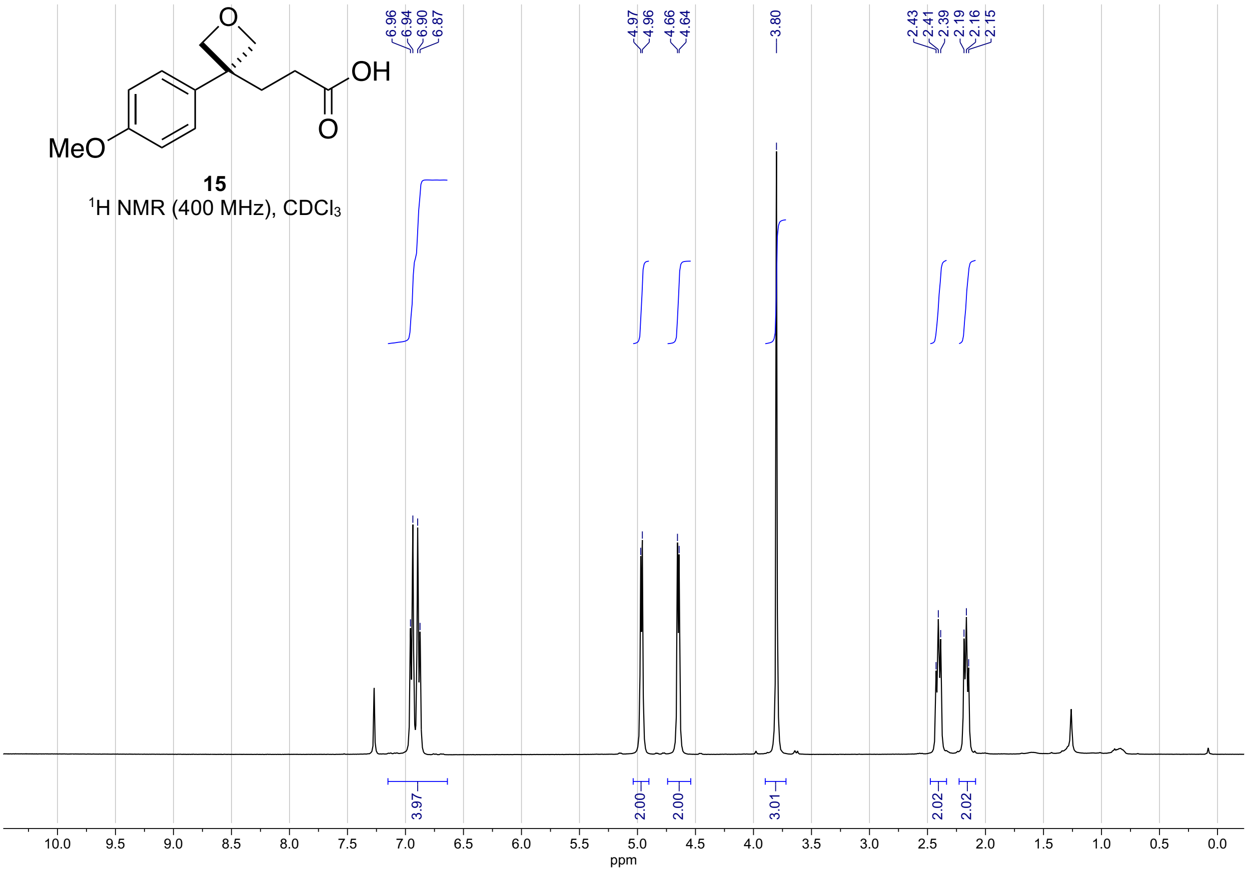


**15**
$^{1}H$ NMR (400 MHz), $CDCl_3$

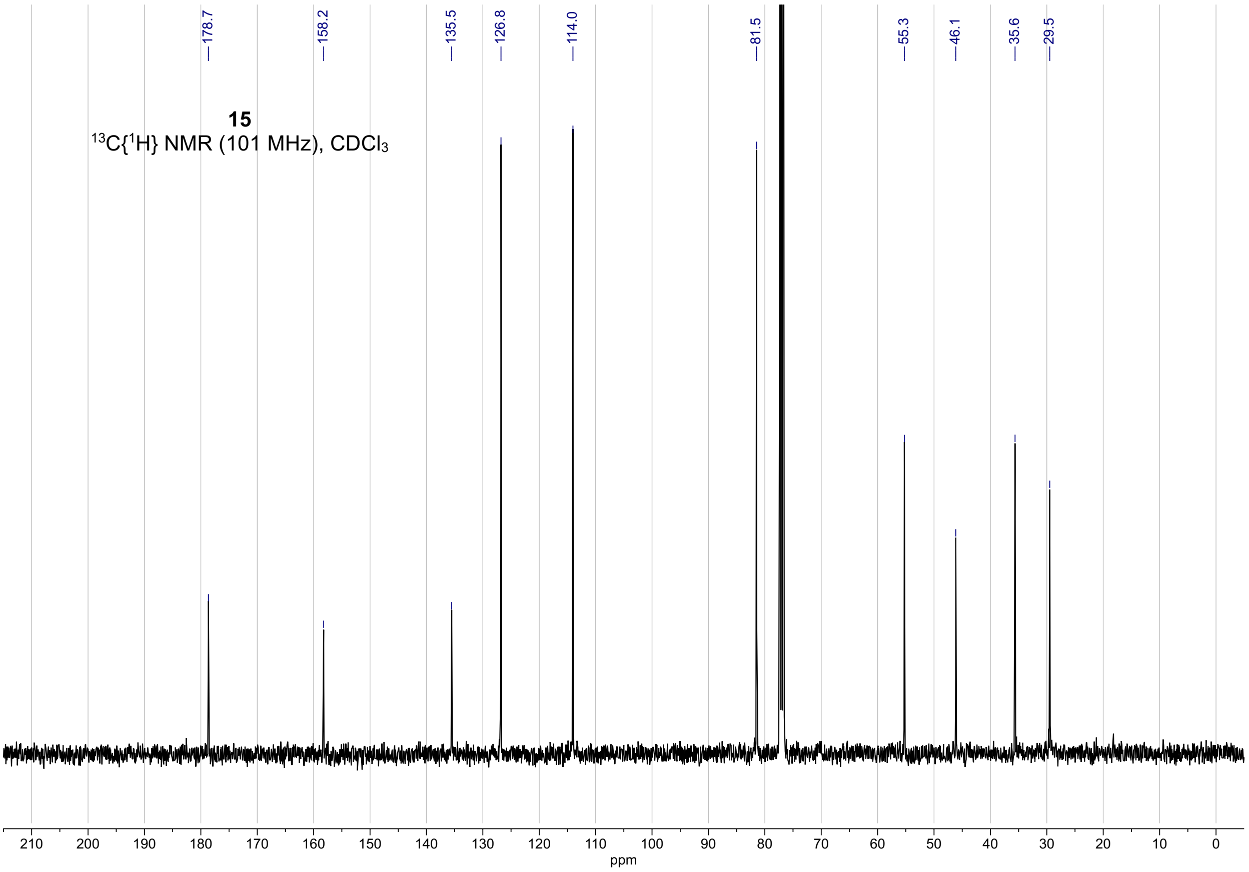


**15**
$^{13}C\{^{1}H\}$ NMR (101 MHz), $CDCl_3$

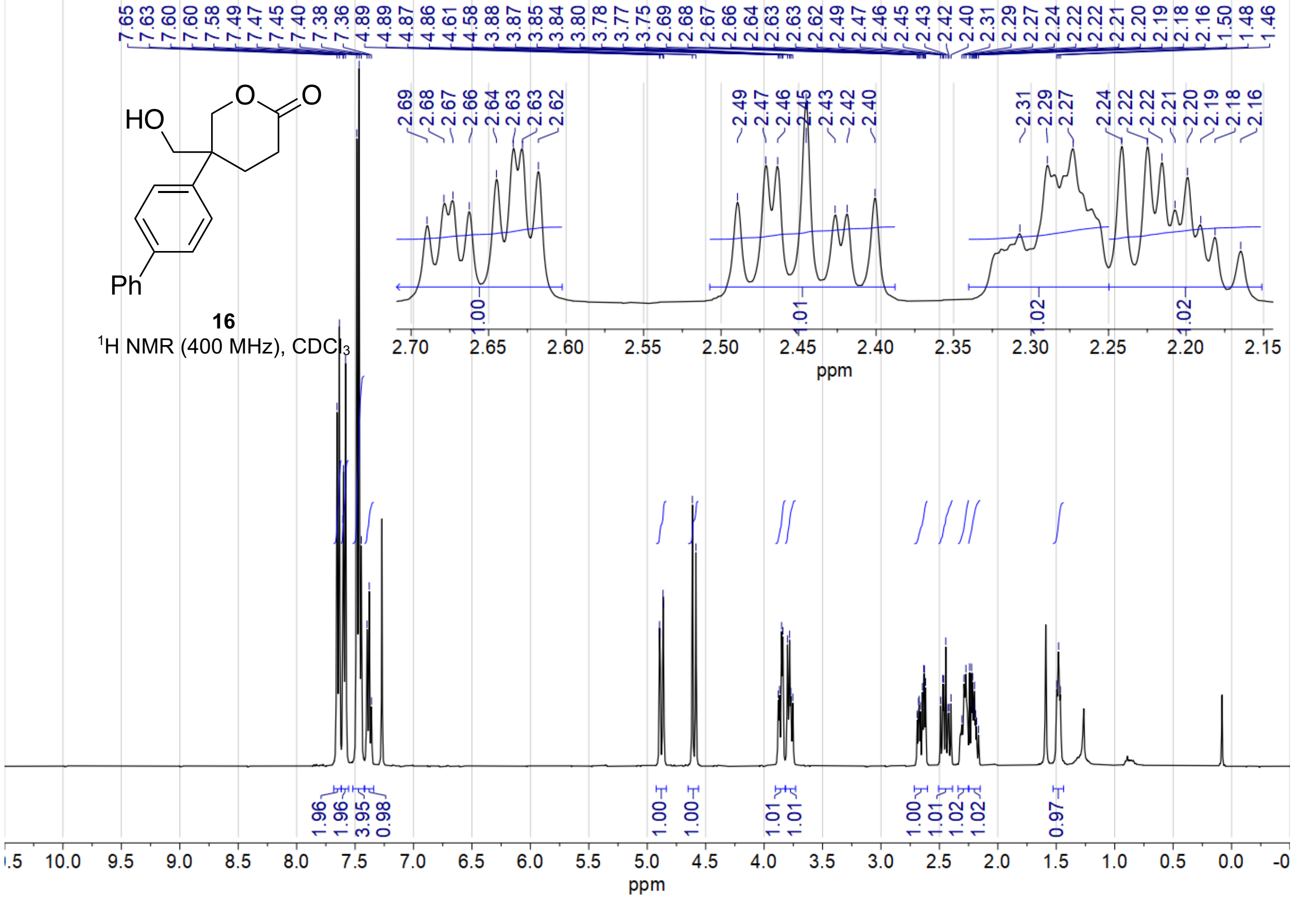
HO
O
O
Ph
16
$^1$H NMR (400 MHz), CDCl$_3$
ppm

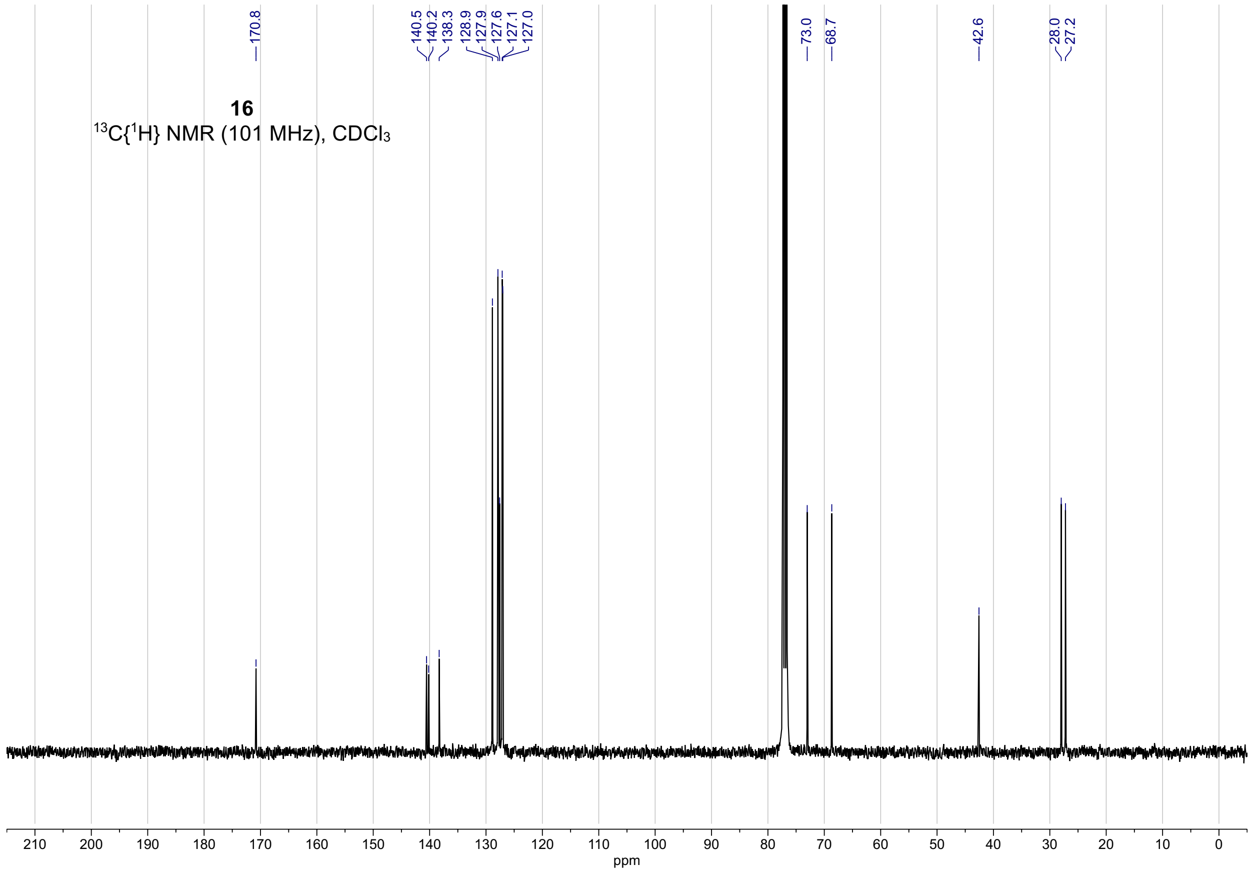
16
$^{13}$C{$^1$H} NMR (101 MHz), CDCl$_3$
170.8
140.5
140.2
138.3
128.9
127.9
127.6
127.1
127.0
73.0
68.7
42.6
28.0
27.2
ppm

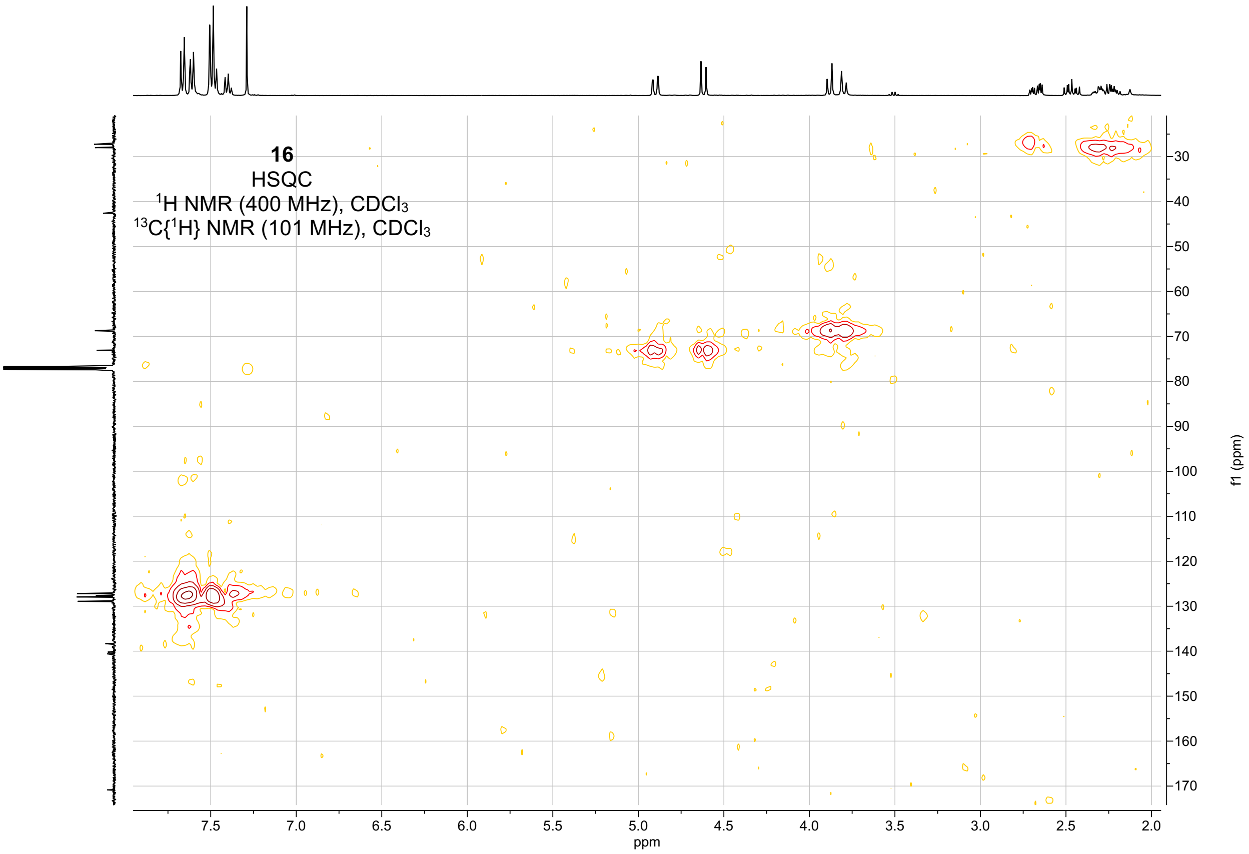
16
HSQC
1H NMR (400 MHz), CDCl3
13C{1H} NMR (101 MHz), CDCl3
ppm
f1 (ppm)

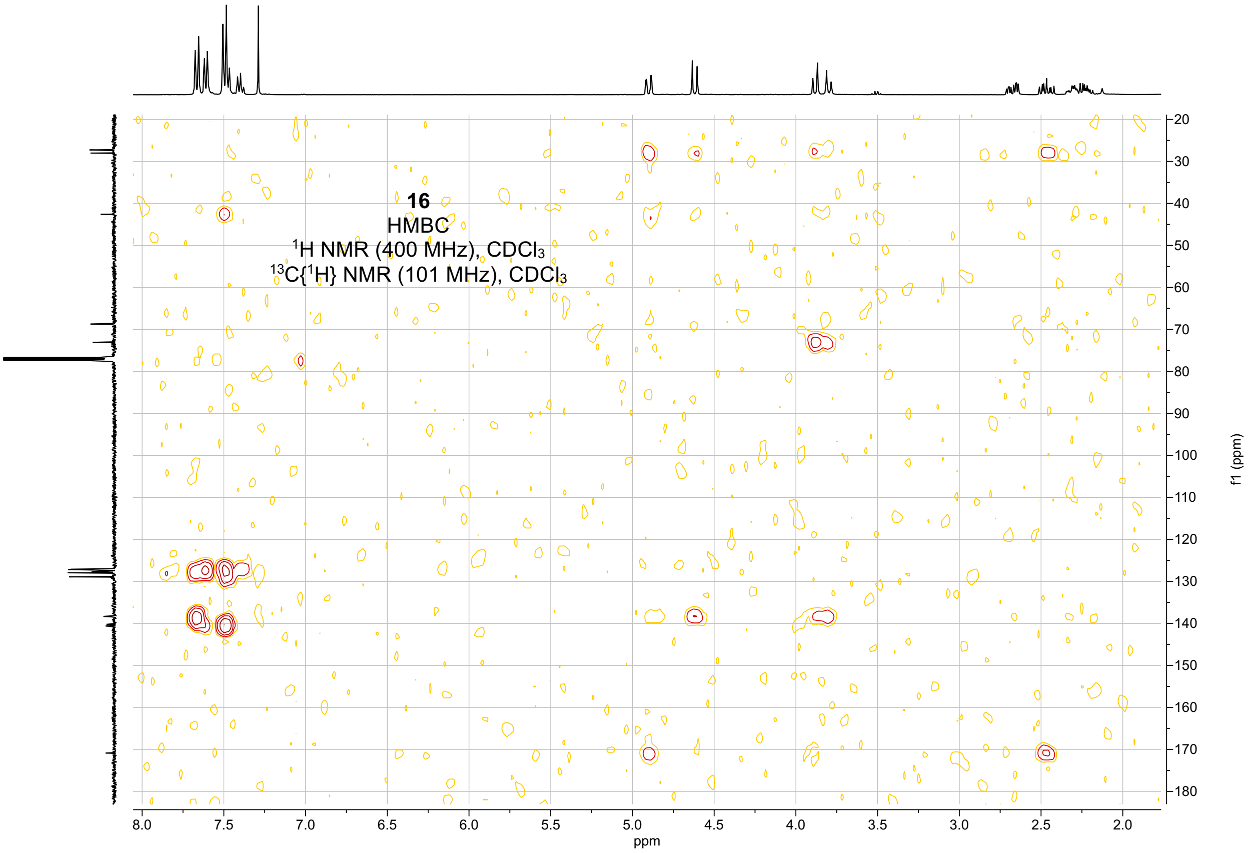
16
HMBC
1H NMR (400 MHz), CDCl3
13C{1H} NMR (101 MHz), CDCl3
ppm
f1 (ppm)

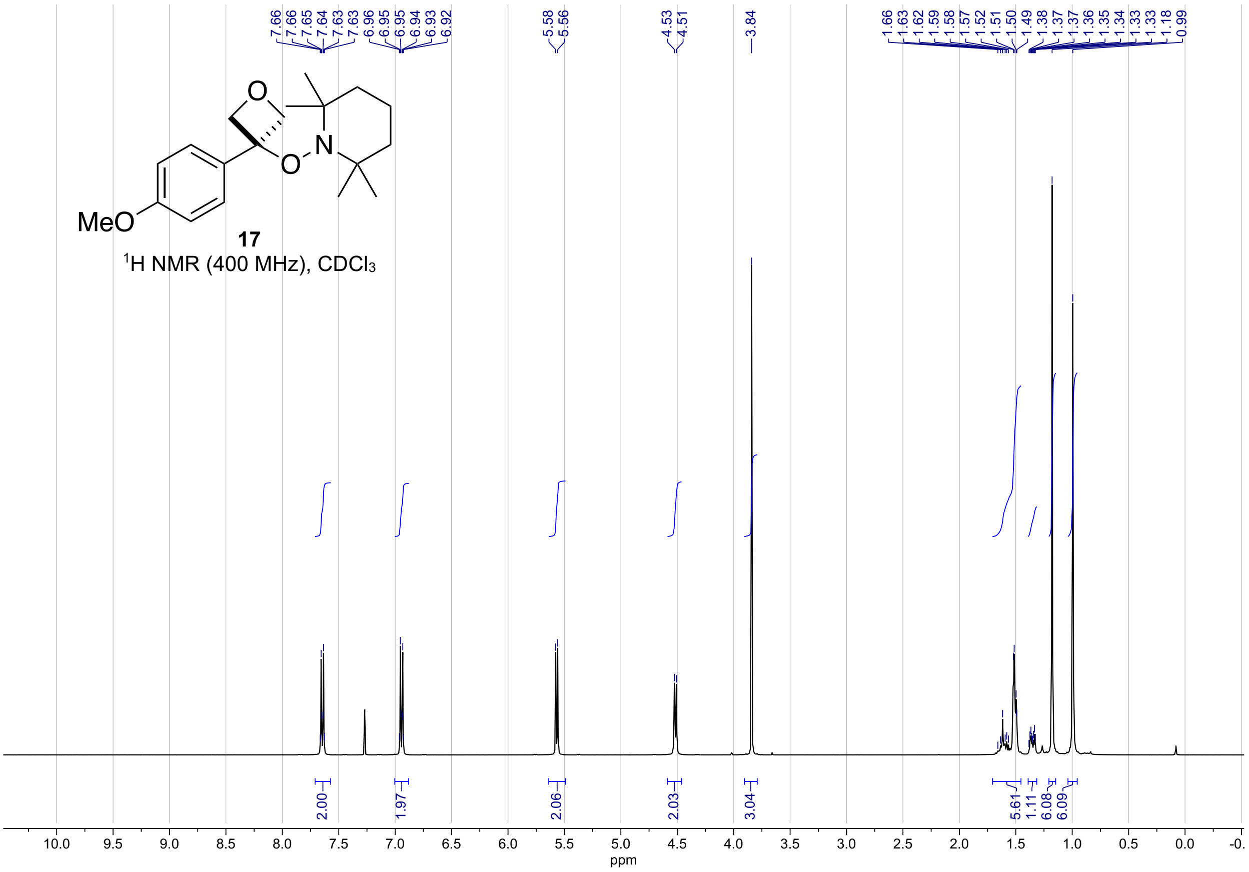
7.66 7.66 7.65 7.64 7.63 7.63 6.96 6.95 6.95 6.94 6.93 6.92
5.58 5.56
4.53 4.51
3.84
1.66 1.63 1.62 1.59 1.58 1.57 1.52 1.51 1.50 1.49 1.38 1.37 1.37 1.36 1.35 1.34 1.33 1.33 1.18 0.99
MeO
O
N
17
1H NMR (400 MHz), CDCl3
2.00 1.97 2.06 2.03 3.04 5.61 1.11 6.08 6.09
ppm

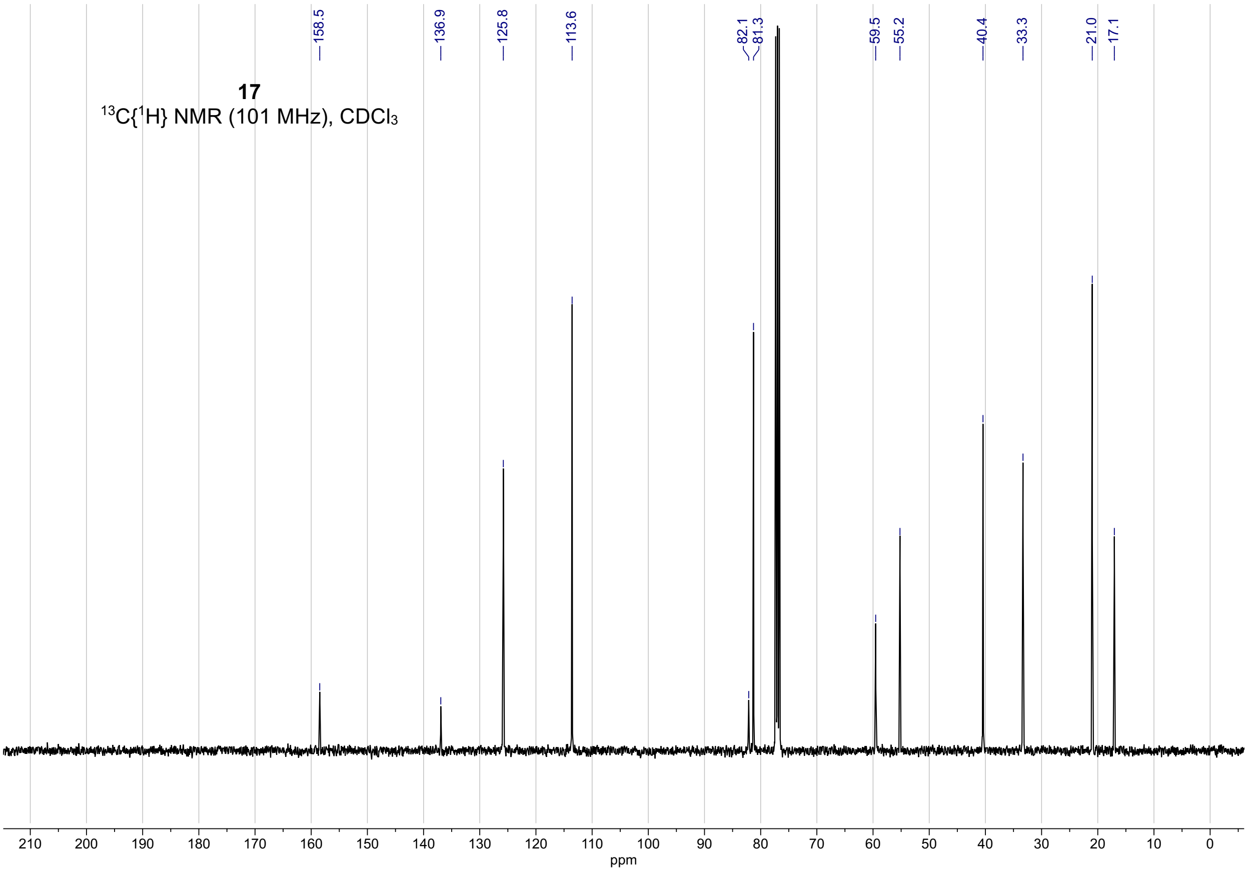
158.5
136.9
125.8
113.6
82.1 81.3
59.5 55.2
40.4 33.3
21.0 17.1
17
13C{1H} NMR (101 MHz), CDCl3
ppm

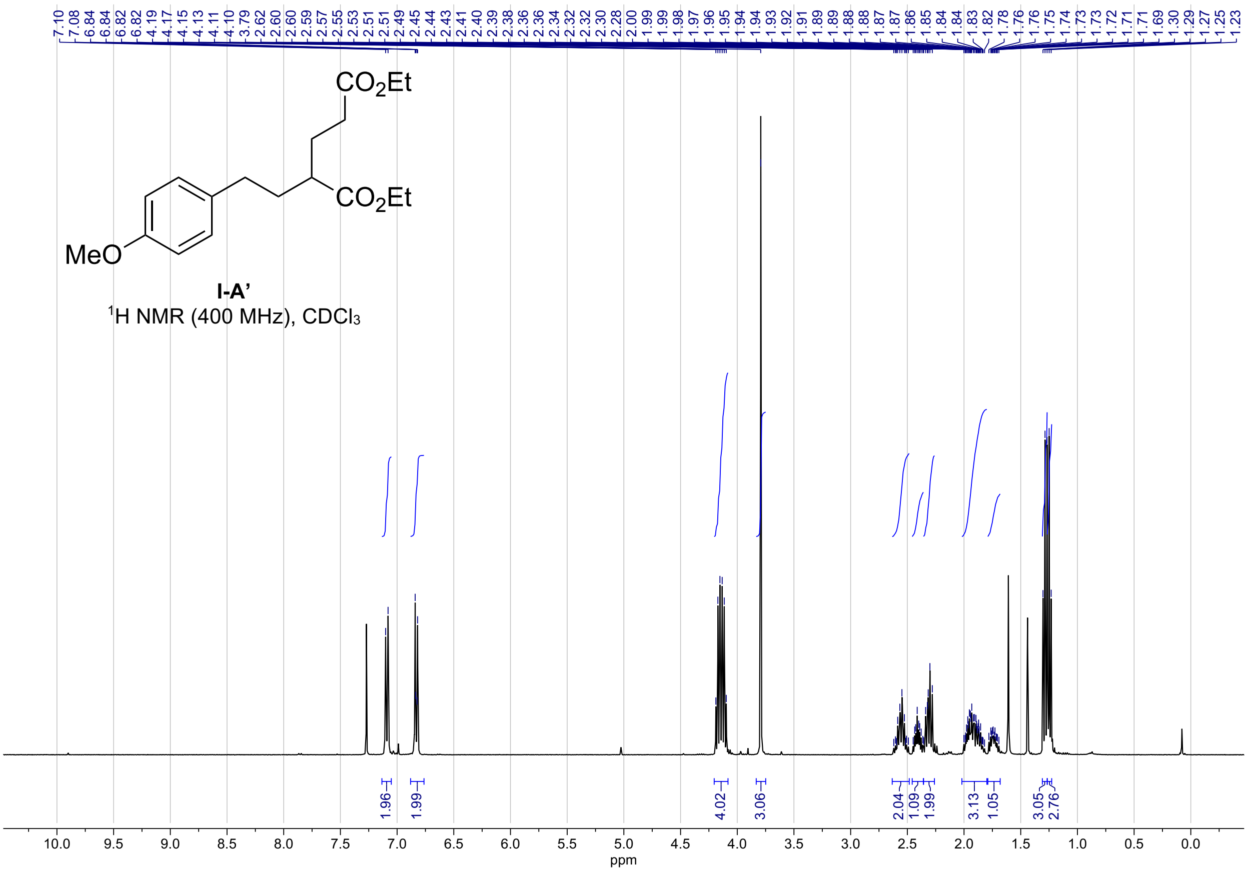
CO2Et
CO2Et
MeO
I-A'
1H NMR (400 MHz), CDCl3
1.96
1.99
4.02
3.06
2.04
1.09
1.99
3.13
1.05
3.05
2.76
ppm

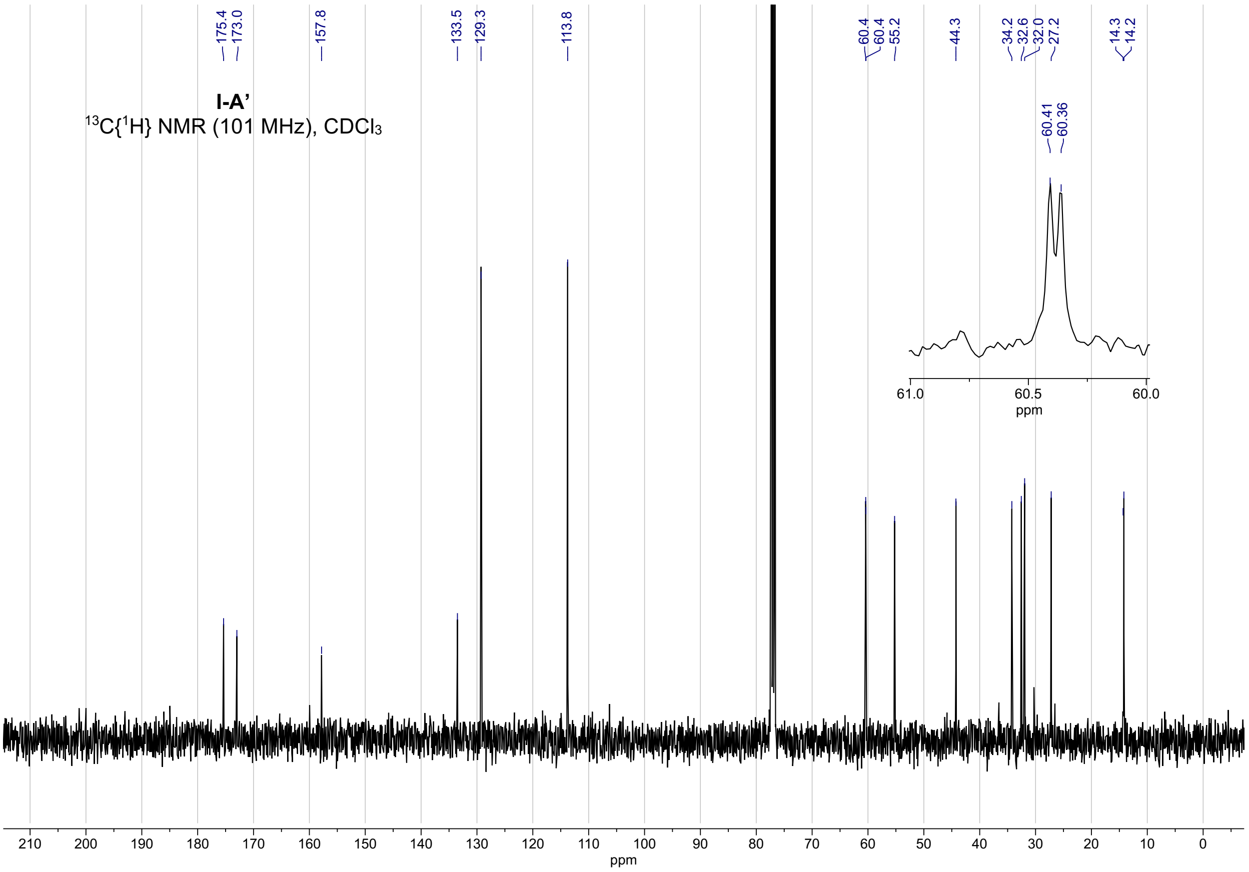
175.4
173.0
157.8
133.5
129.3
113.8
60.4
60.4
55.2
44.3
34.2
32.6
32.0
27.2
14.3
14.2
I-A'
13C{1H} NMR (101 MHz), CDCl3
60.41
60.36
ppm

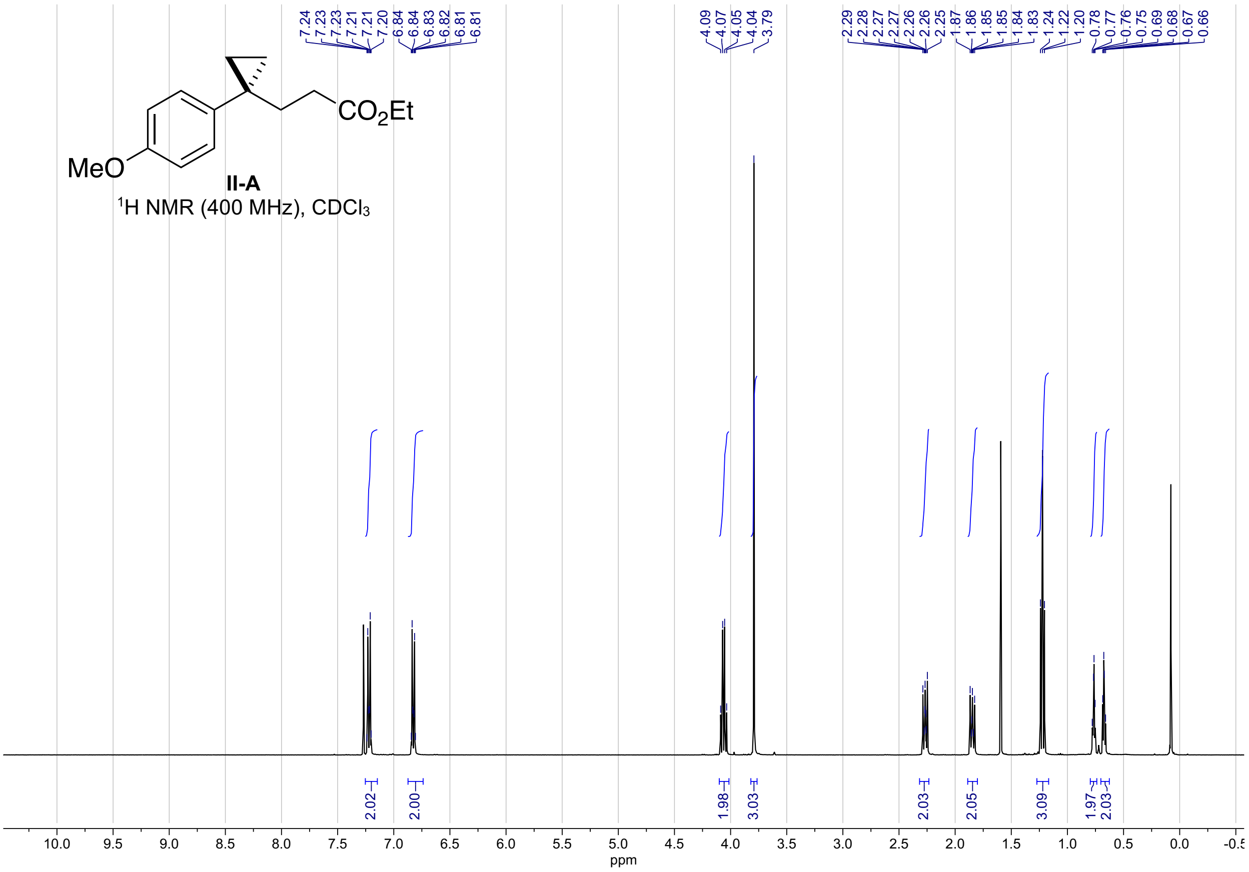
MeO
CO2Et
II-A
1H NMR (400 MHz), CDCl3
ppm

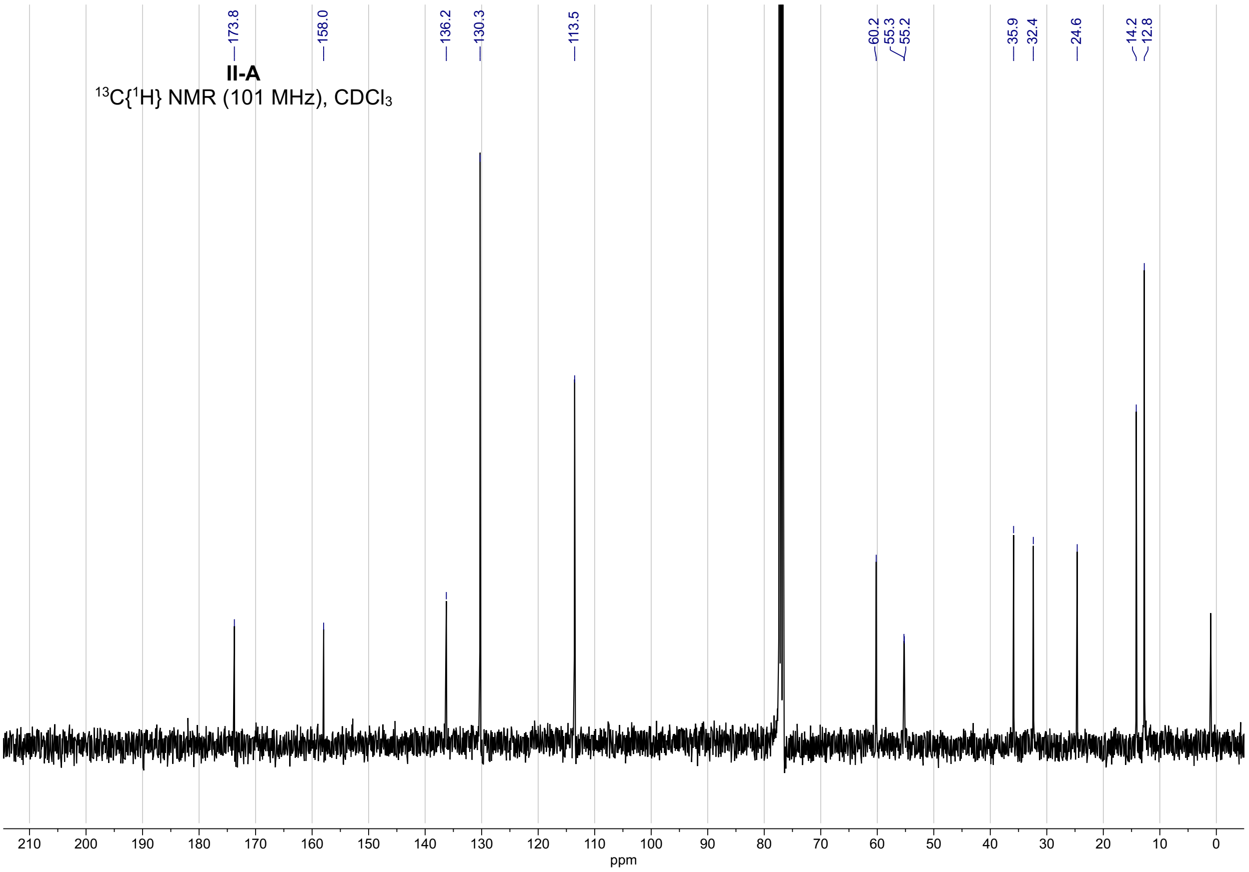
II-A
13C{1H} NMR (101 MHz), CDCl3
ppm

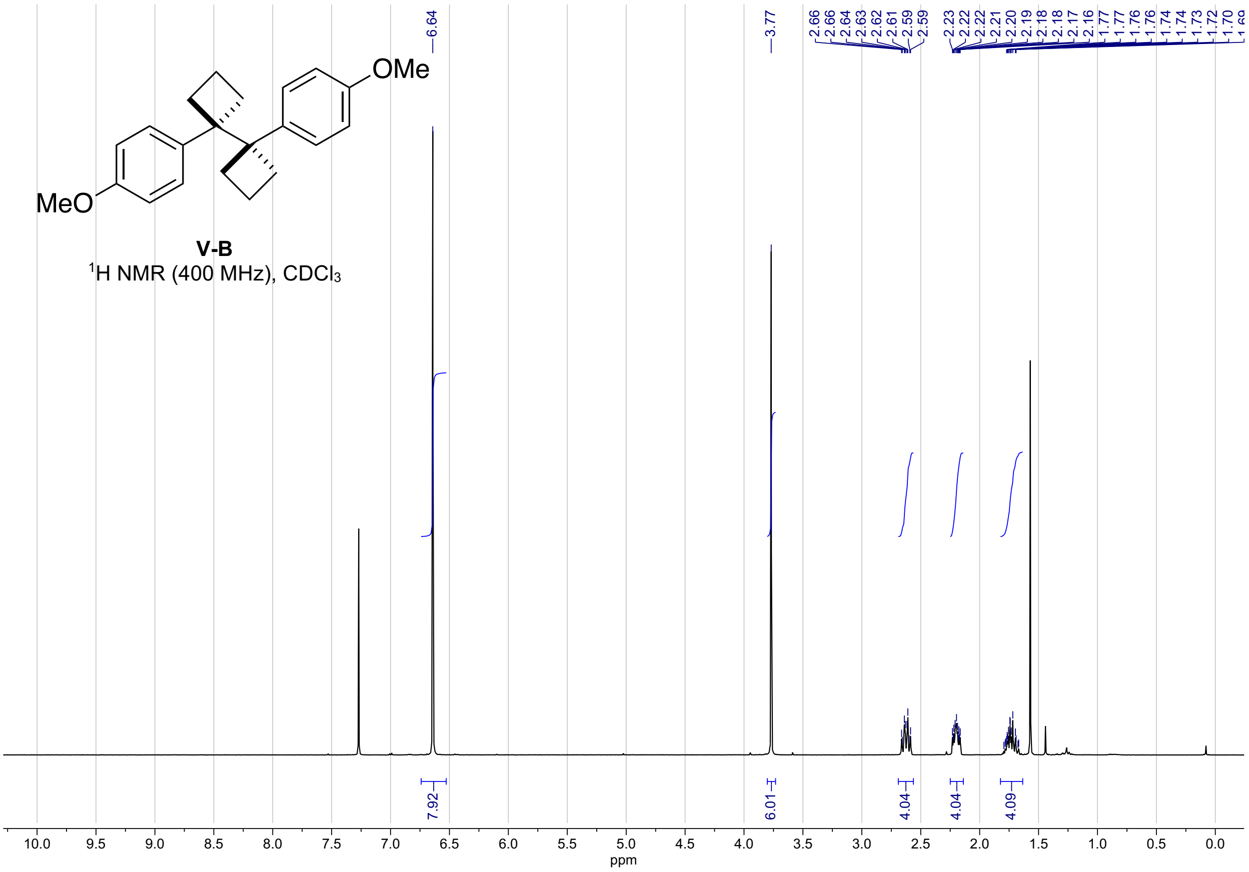
OMe
MeO
**V-B**
$^{1}H$ NMR (400 MHz), $CDCl_3$
6.64
3.77
2.66 2.66 2.64 2.63 2.62 2.61 2.59 2.59
2.23 2.22 2.22 2.21 2.20 2.19 2.18 2.17 2.16
1.77 1.77 1.76 1.76 1.74 1.73 1.72 1.70
7.92
6.01
4.04
4.04
4.09
10.0 9.5 9.0 8.5 8.0 7.5 7.0 6.5 6.0 5.5 5.0 4.5 4.0 3.5 3.0 2.5 2.0 1.5 1.0 0.5 0.0
ppm

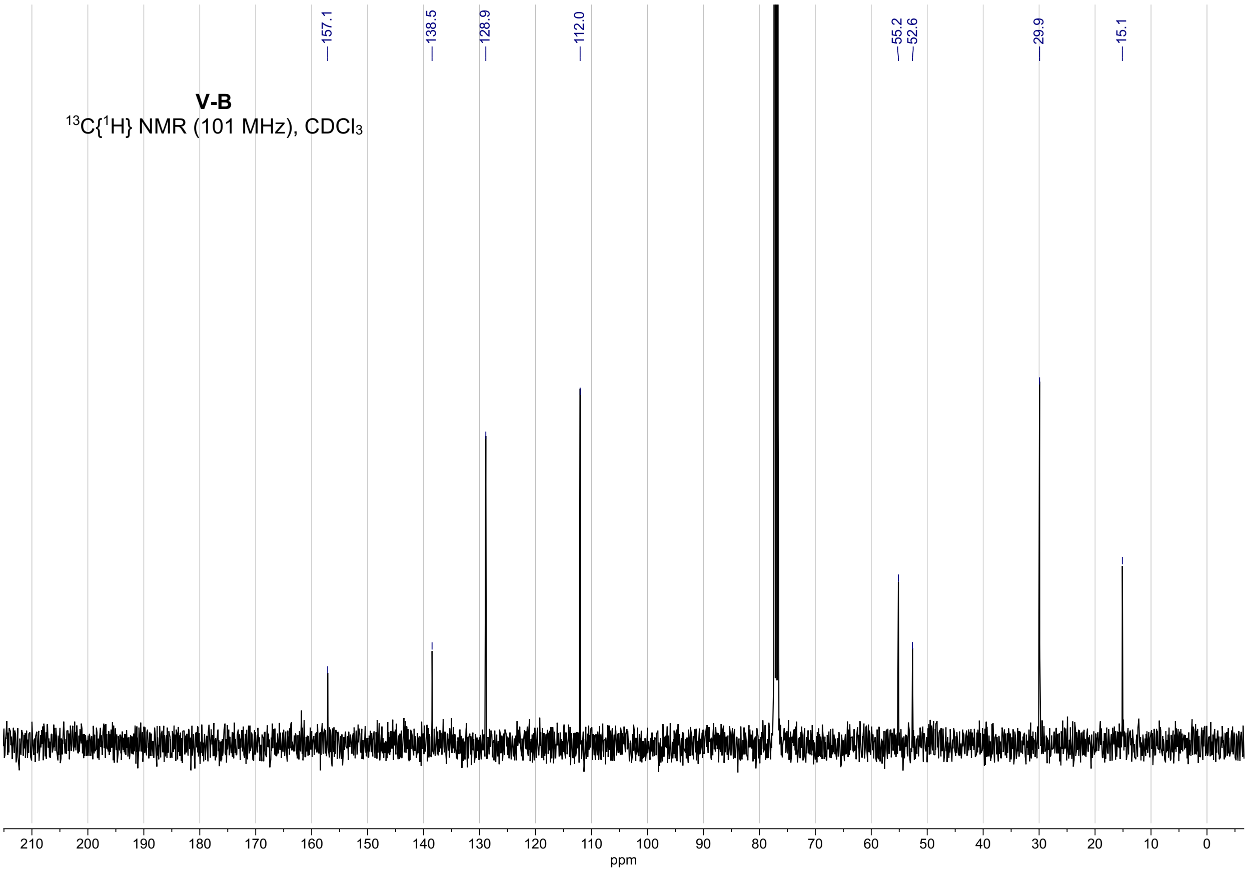
**V-B**
$^{13}C\{^{1}H\}$ NMR (101 MHz), $CDCl_3$
157.1
138.5
128.9
112.0
55.2
52.6
29.9
15.1
210 200 190 180 170 160 150 140 130 120 110 100 90 80 70 60 50 40 30 20 10 0
ppm

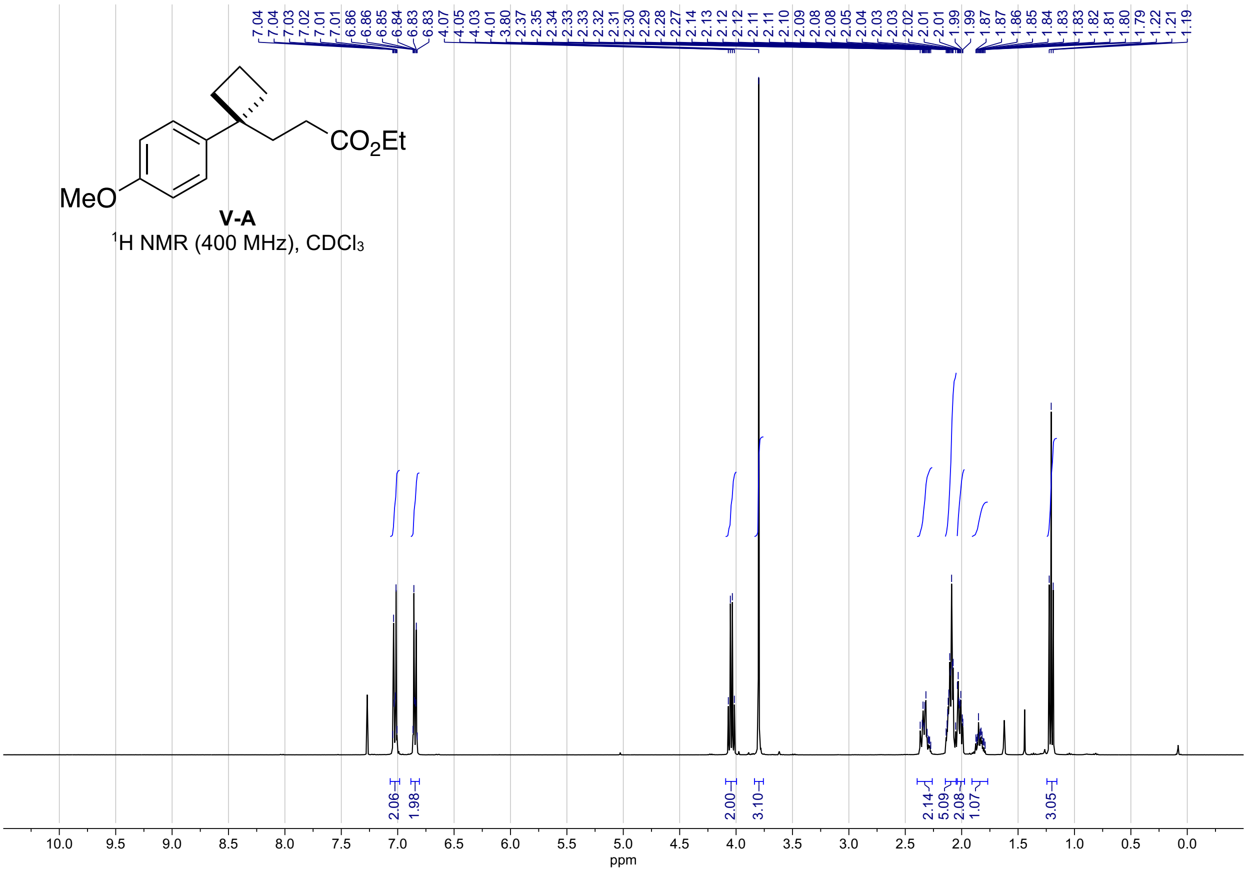
7.04
7.04
7.03
7.02
7.01
7.01
6.86
6.86
6.85
6.84
6.83
6.83
4.07
4.05
4.03
4.01
3.80
2.37
2.35
2.34
2.33
2.33
2.32
2.31
2.30
2.29
2.28
2.27
2.14
2.13
2.12
2.12
2.11
2.11
2.10
2.09
2.08
2.08
2.05
2.04
2.03
2.03
2.02
2.01
2.01
1.99
1.99
1.87
1.87
1.86
1.85
1.84
1.83
1.83
1.82
1.81
1.80
1.79
1.22
1.21
1.19
CO2Et
MeO
V-A
1H NMR (400 MHz), CDCl3
2.06
1.98
2.00
3.10
2.14
5.09
2.08
1.07
3.05
10.0
9.5
9.0
8.5
8.0
7.5
7.0
6.5
6.0
5.5
5.0
4.5
4.0
3.5
3.0
2.5
2.0
1.5
1.0
0.5
0.0
ppm

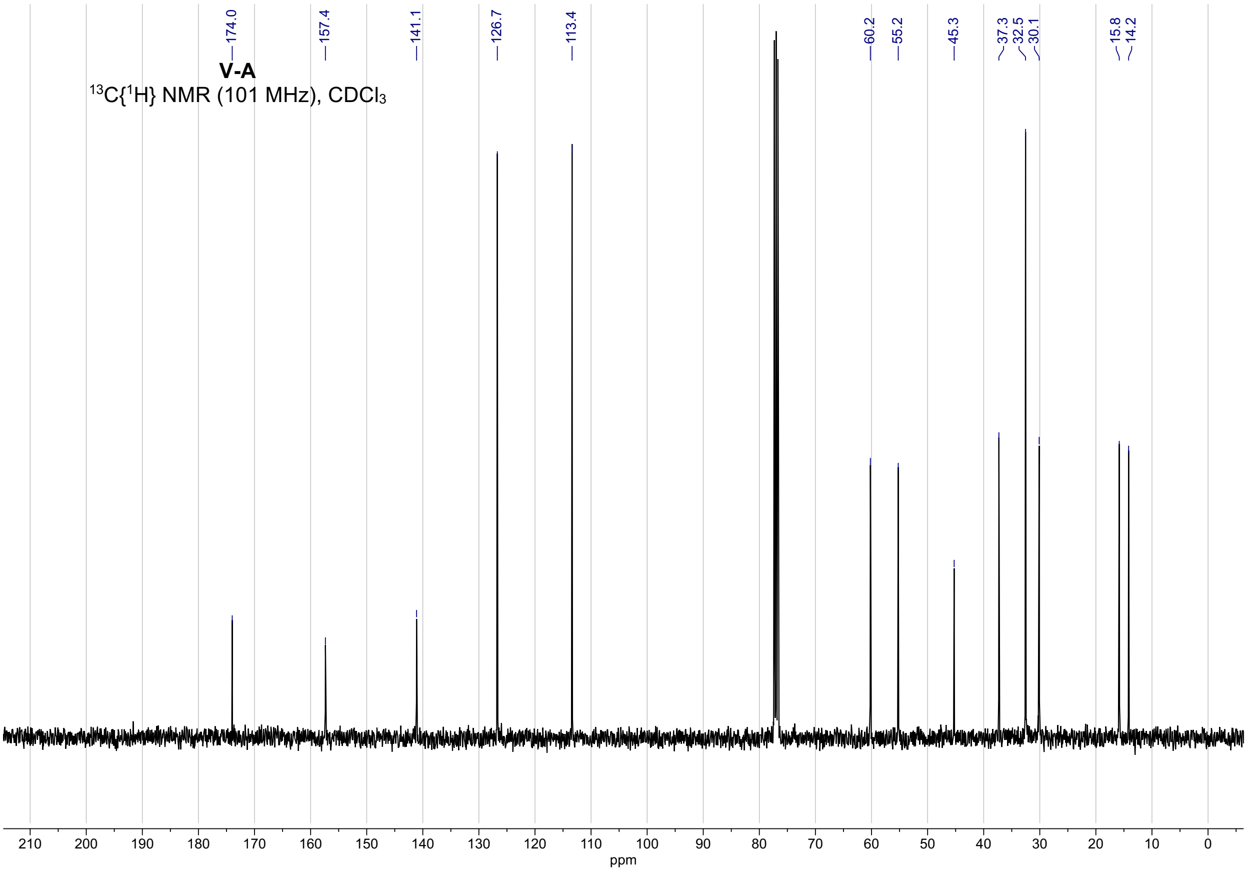
174.0
157.4
141.1
126.7
113.4
60.2
55.2
45.3
37.3
32.5
30.1
15.8
14.2
V-A
13C{1H} NMR (101 MHz), CDCl3
210
200
190
180
170
160
150
140
130
120
110
100
90
80
70
60
50
40
30
20
10
0
ppm

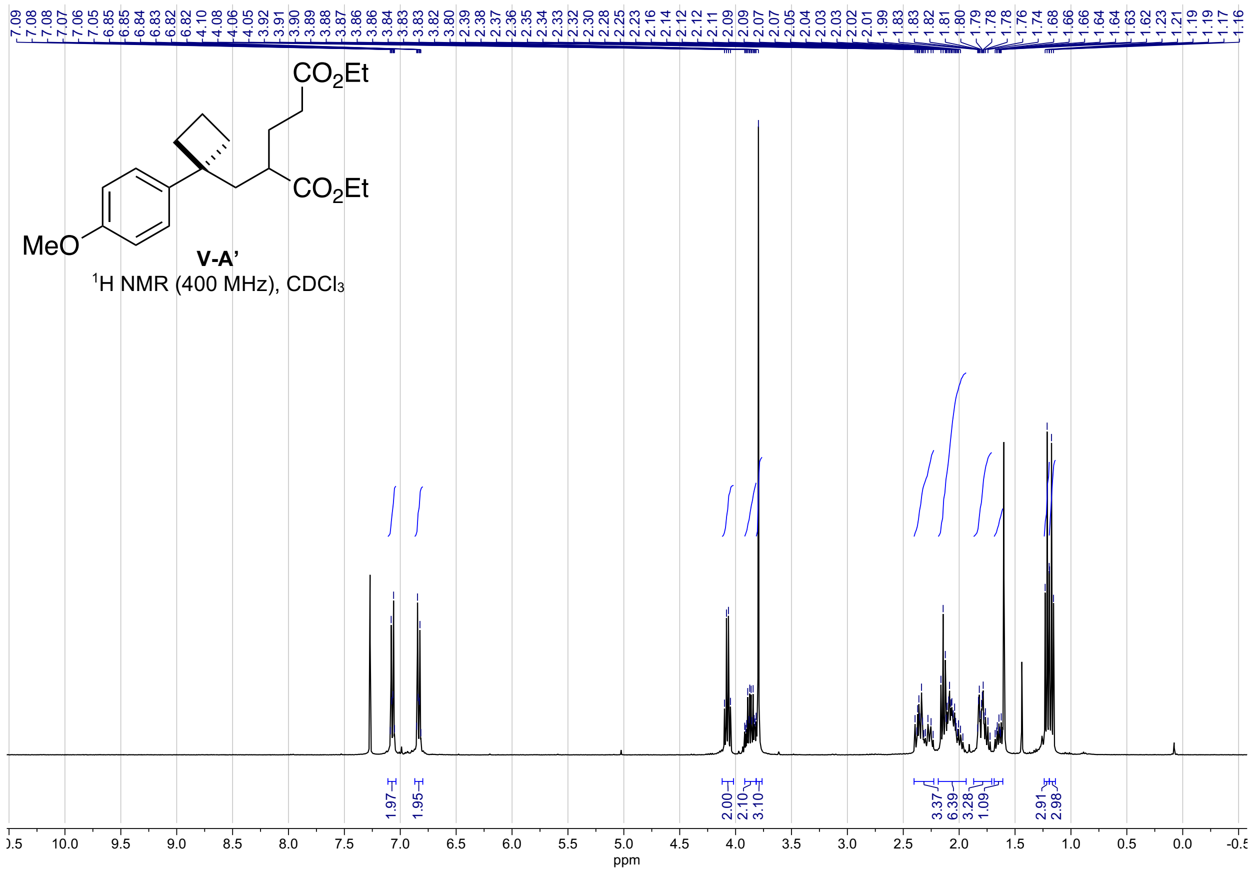
CO2Et
CO2Et
MeO
V-A'
1H NMR (400 MHz), CDCl3
ppm

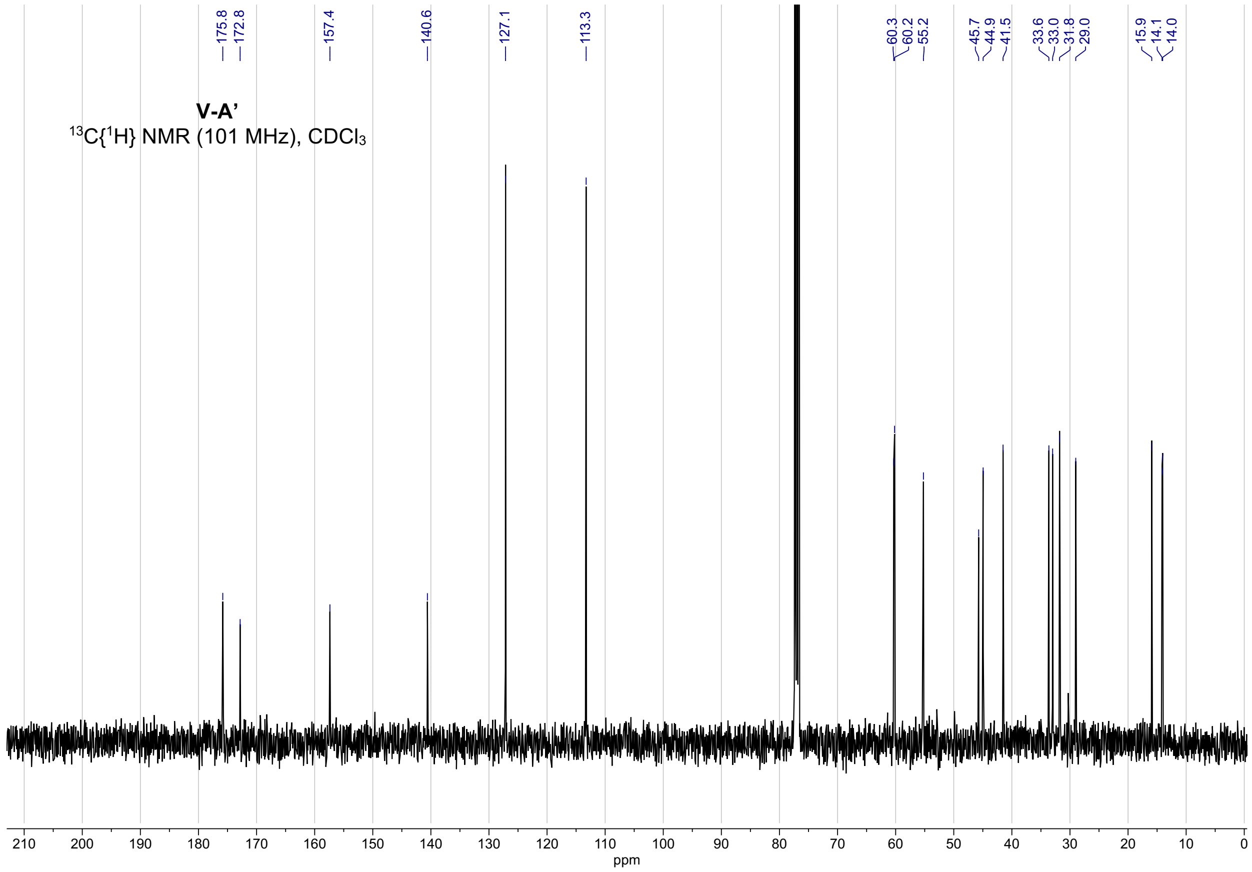
175.8
172.8
157.4
140.6
127.1
113.3
60.3
60.2
55.2
45.7
44.9
41.5
33.6
33.0
31.8
29.0
15.9
14.1
14.0
V-A'
13C{1H} NMR (101 MHz), CDCl3
ppm

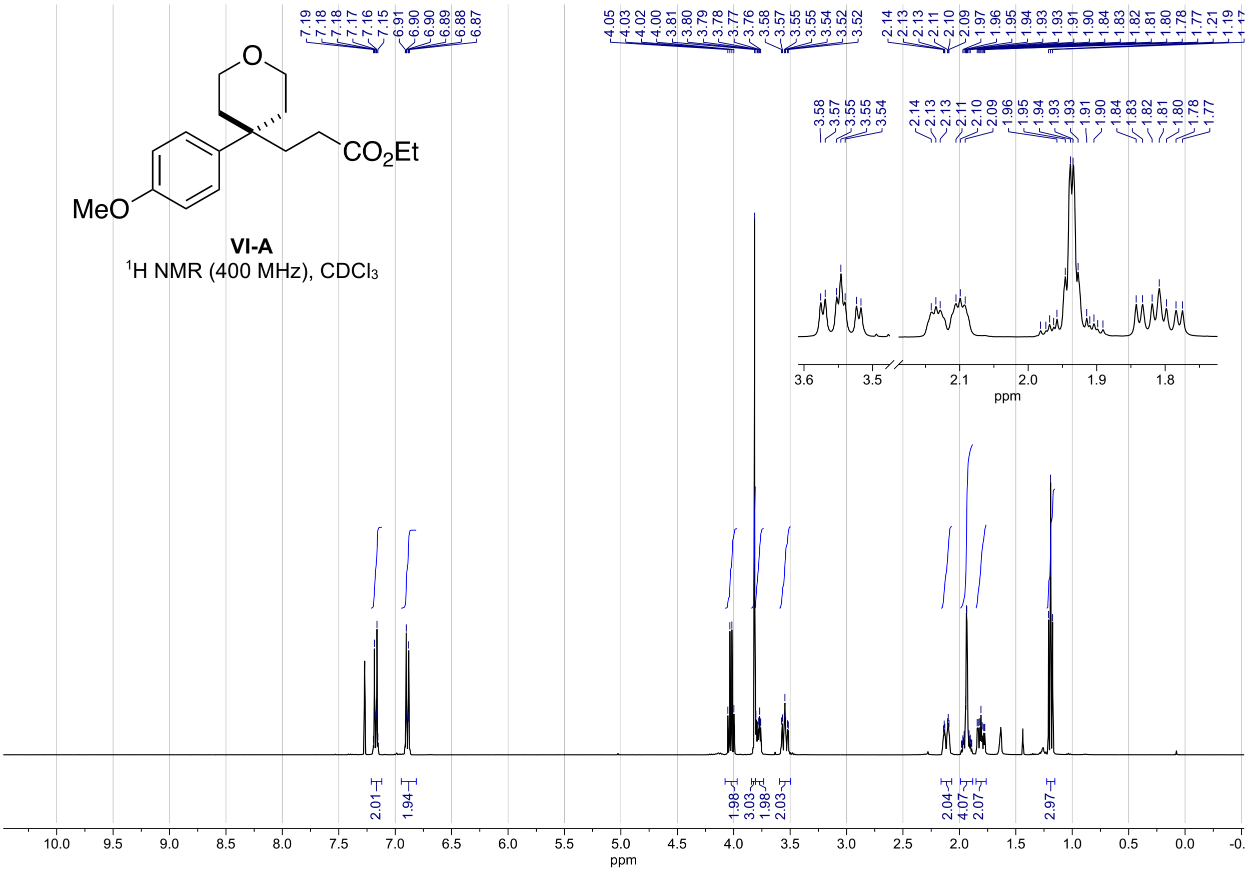
CO2Et
MeO
VI-A
1H NMR (400 MHz), CDCl3
ppm

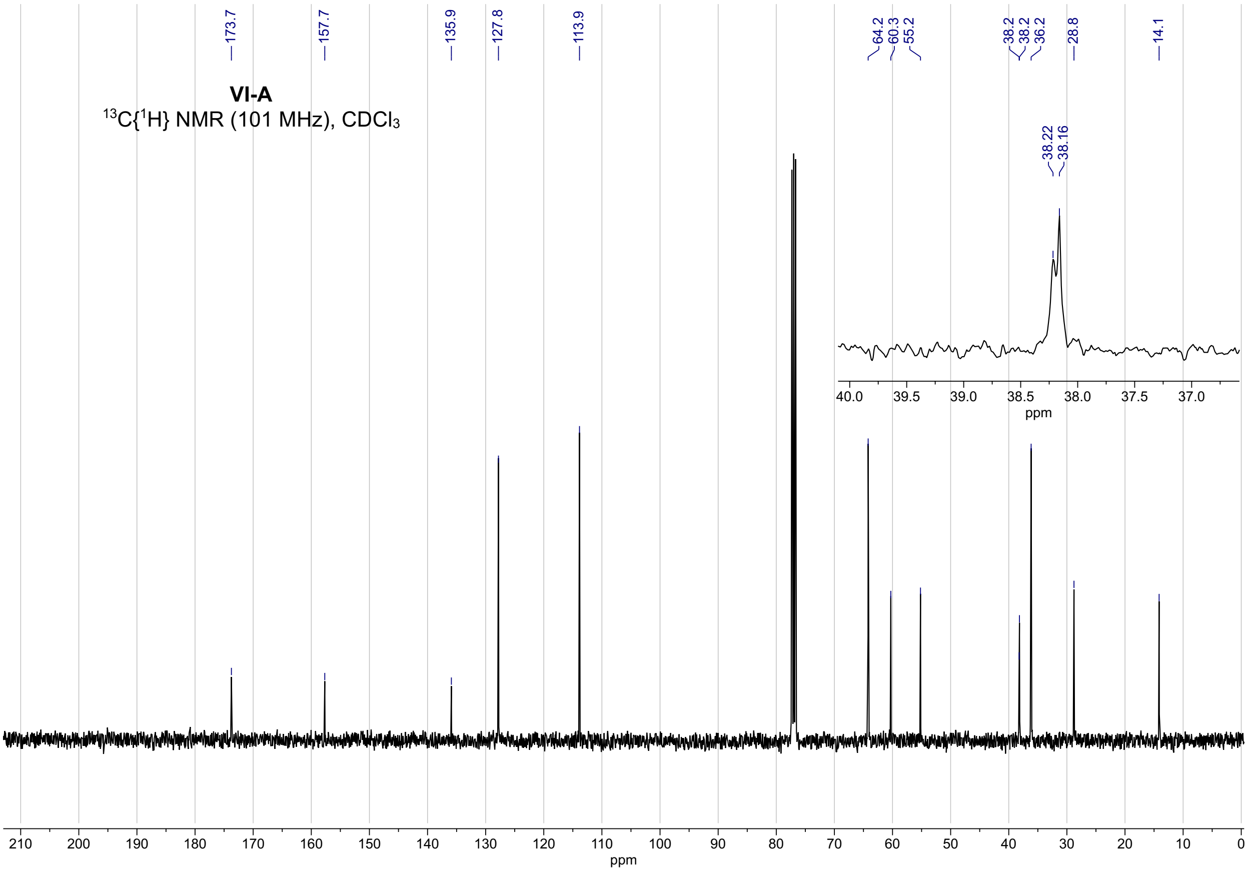
VI-A
13C{1H} NMR (101 MHz), CDCl3
ppm

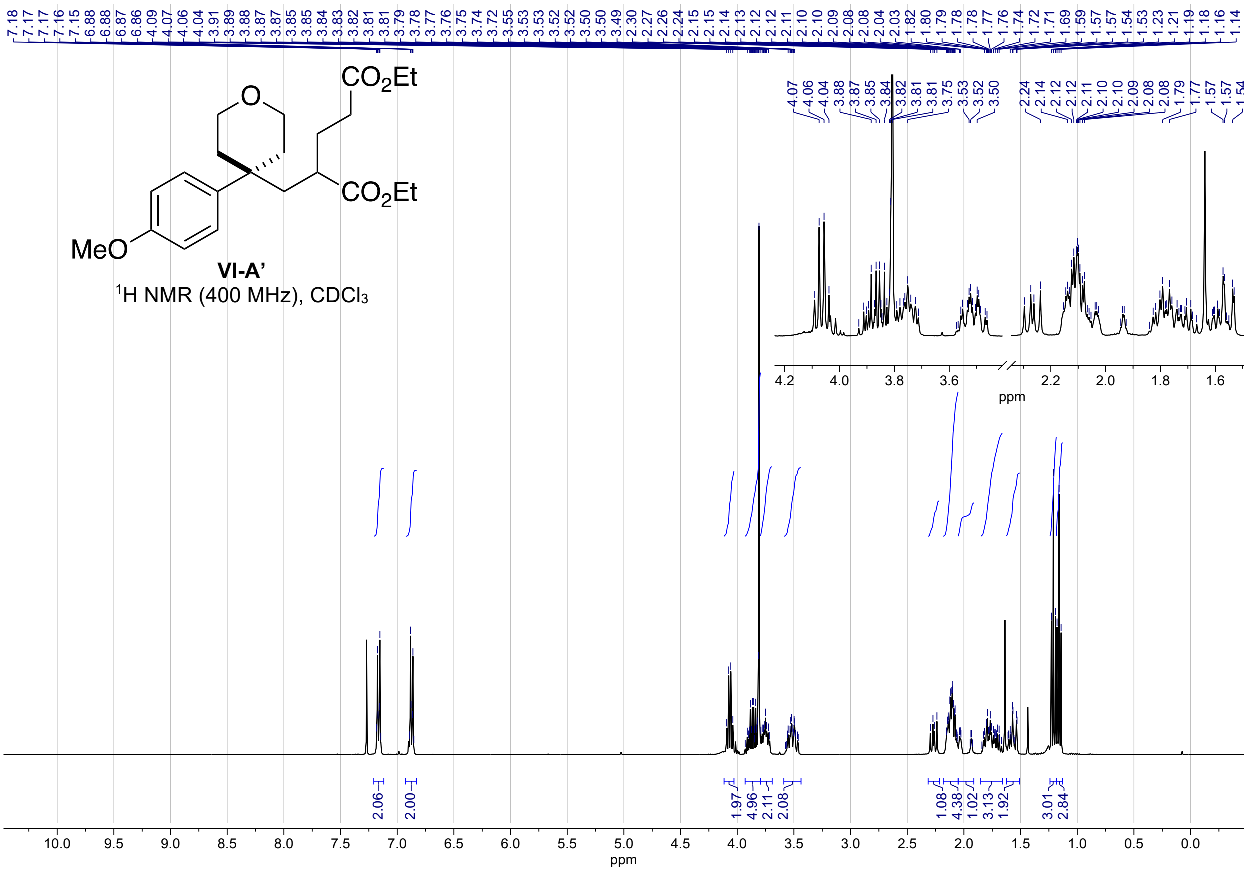
CO2Et
O
CO2Et
MeO
VI-A'
1H NMR (400 MHz), CDCl3
ppm

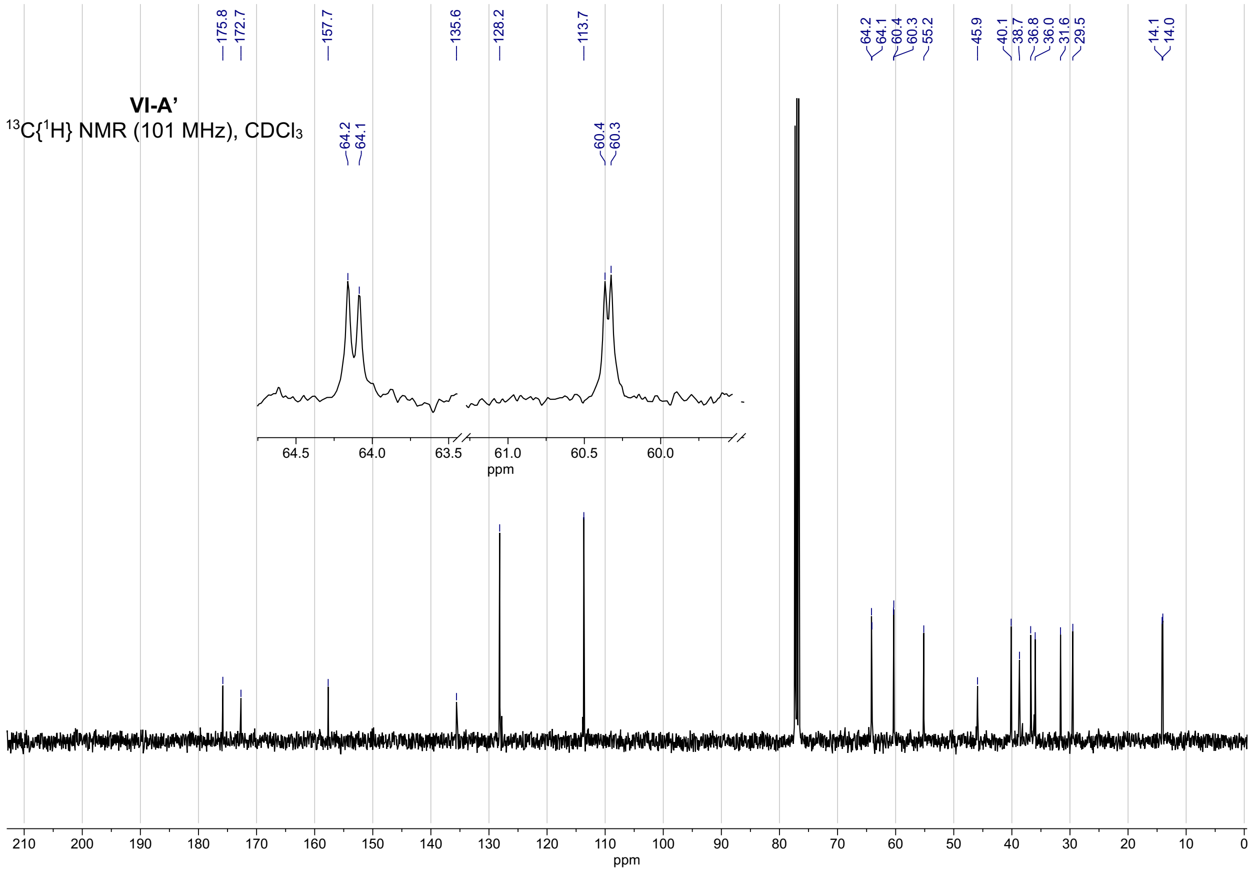
VI-A'
13C{1H} NMR (101 MHz), CDCl3
175.8
172.7
157.7
135.6
128.2
113.7
64.2
64.1
60.4
60.3
55.2
45.9
40.1
38.7
36.8
36.0
31.6
29.5
14.1
14.0
ppm

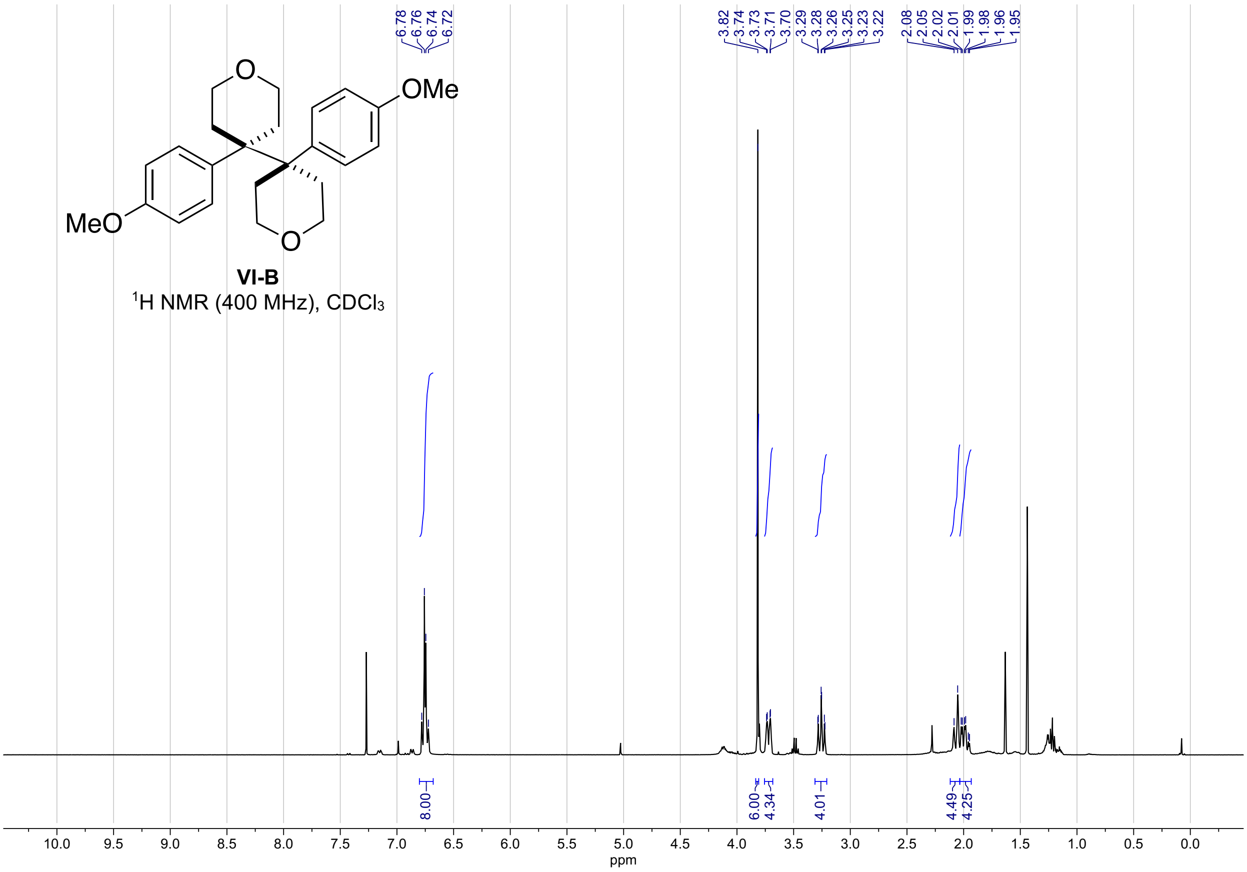
6.78
6.76
6.74
6.72
3.82
3.74
3.73
3.71
3.70
3.29
3.28
3.26
3.25
3.23
3.22
2.08
2.05
2.02
2.01
1.99
1.98
1.96
1.95
OMe
O
MeO
O
**VI-B**
$^{1}$H NMR (400 MHz), $CDCl_3$
8.00
6.00
4.34
4.01
4.49
4.25
ppm

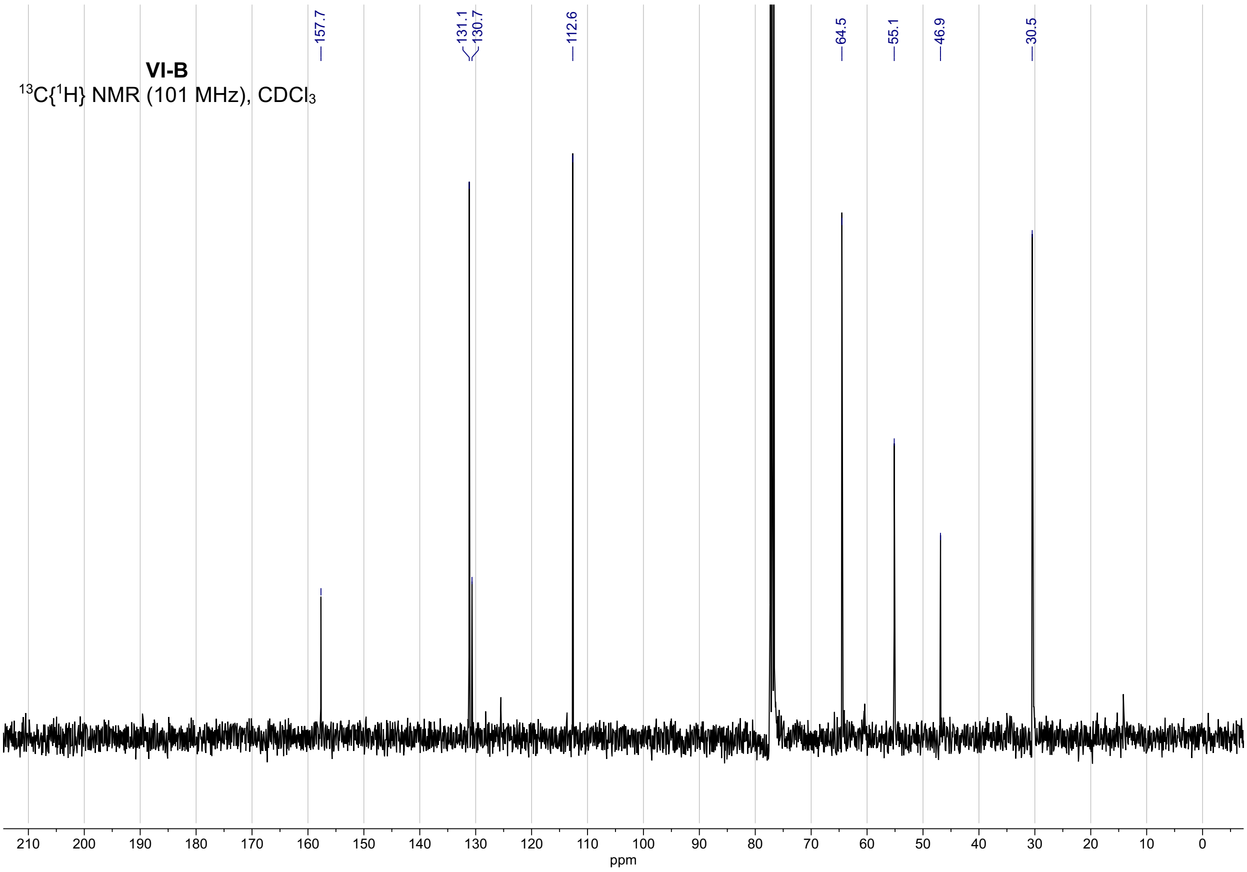
157.7
131.1
130.7
112.6
64.5
55.1
46.9
30.5
**VI-B**
$^{13}C\{^{1}H\}$ NMR (101 MHz), $CDCl_3$
ppm

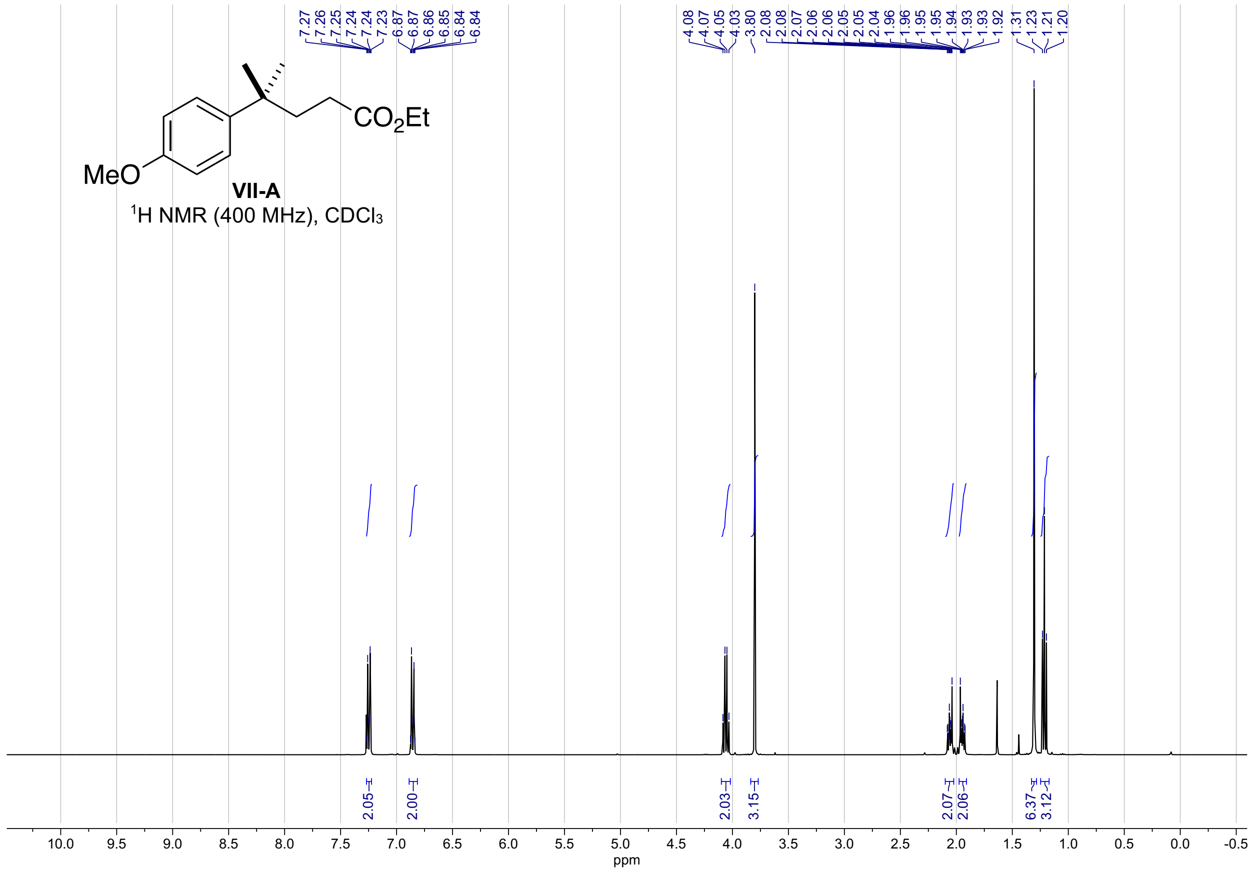
7.27
7.26
7.25
7.24
7.24
7.23
6.87
6.87
6.86
6.85
6.84
6.84
4.08
4.07
4.05
4.03
3.80
2.08
2.08
2.07
2.06
2.06
2.05
2.05
2.04
1.96
1.96
1.95
1.95
1.94
1.93
1.93
1.92
1.31
1.23
1.21
1.20
CO2Et
MeO
VII-A
1H NMR (400 MHz), CDCl3
2.05
2.00
2.03
3.15
2.07
2.06
6.37
3.12
ppm

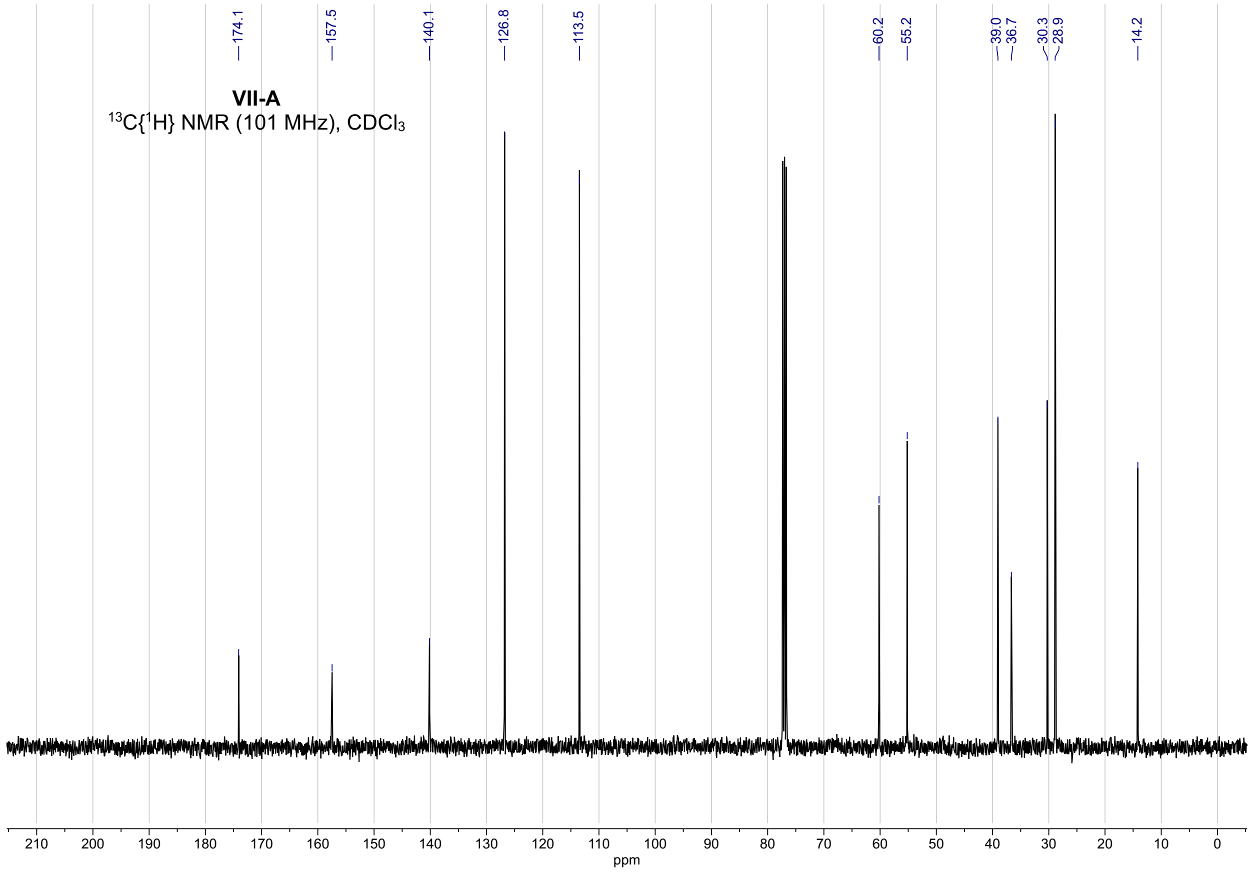
174.1
157.5
140.1
126.8
113.5
60.2
55.2
39.0
36.7
30.3
28.9
14.2
VII-A
13C{1H} NMR (101 MHz), CDCl3
ppm

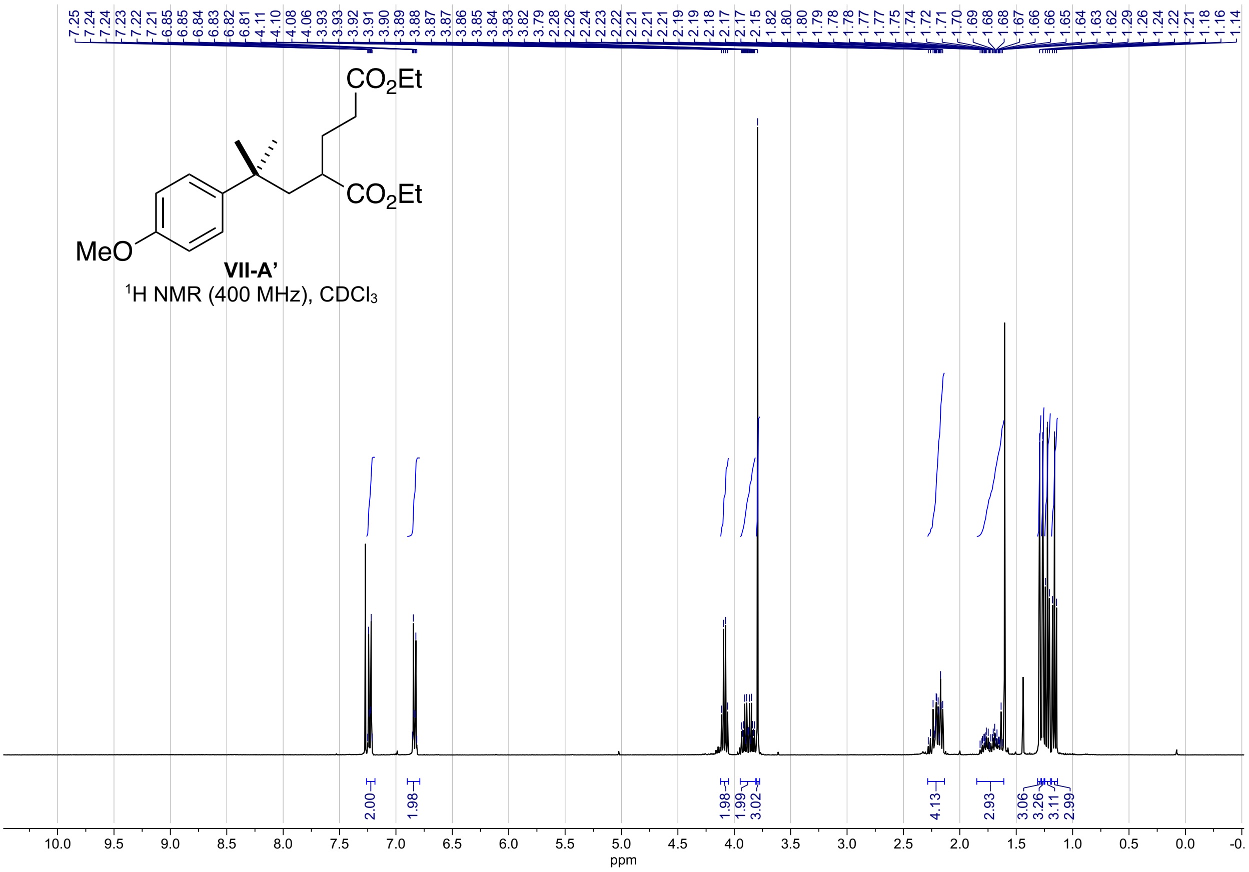
CO2Et
CO2Et
MeO
VII-A'
1H NMR (400 MHz), CDCl3
ppm

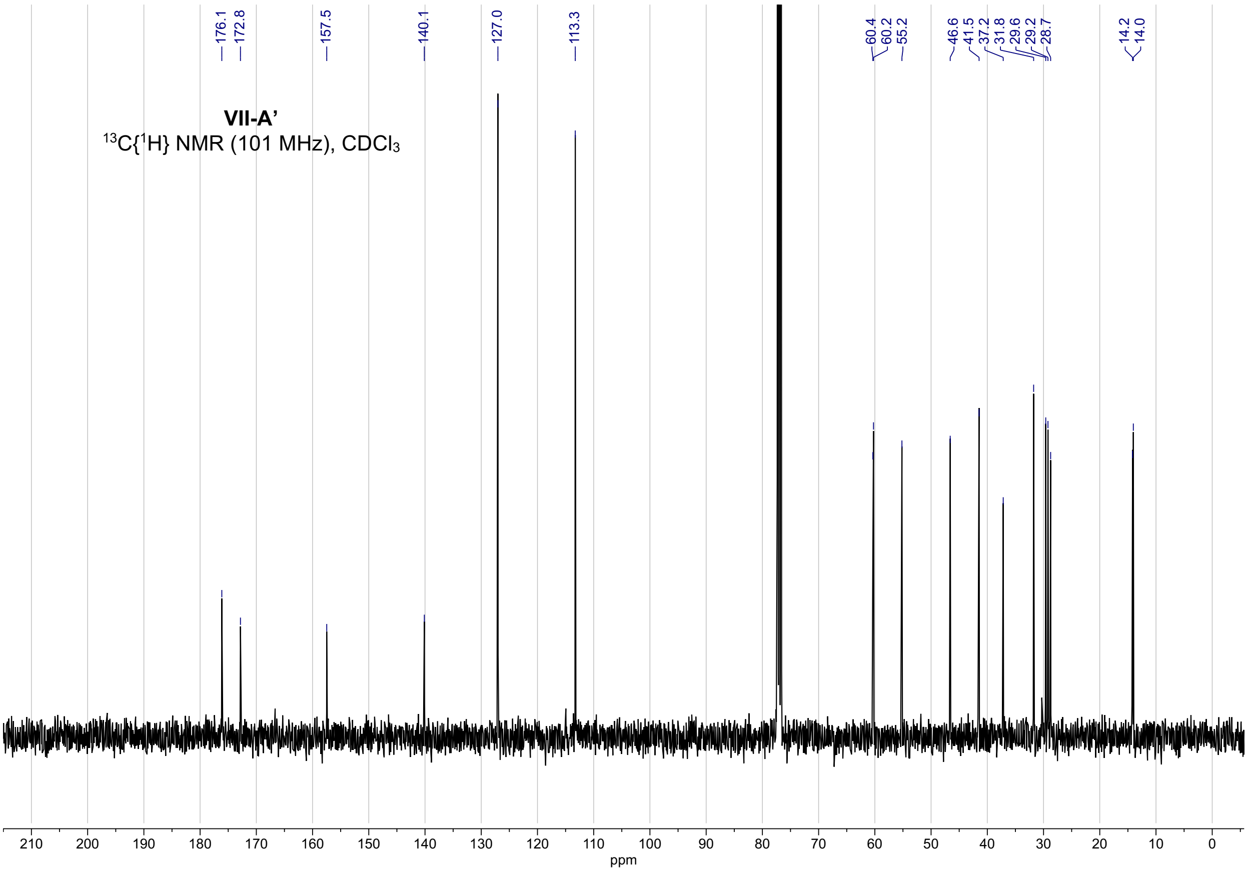
VII-A'
13C{1H} NMR (101 MHz), CDCl3
176.1
172.8
157.5
140.1
127.0
113.3
60.4
60.2
55.2
46.6
41.5
37.2
31.8
29.6
29.2
28.7
14.2
14.0
ppm

## References


1 (a) Ryder, A. S. H.; Cunningham, W. B.; Ballantyne, G.; Mules, T.; Kinsella, A. G.; Turner-Dore, J.; Alder, C. M.; Edwards, L. J.; McKay, B. S. J.; Grayson, M. N.; Cresswell, A. J. *Angew. Chem. Int. Ed.* **2020**, *59*, 14986–14991. (b) Askey, H. E.; Grayson, J. D.; Tibbetts, J. D.; Turner-Dore, J. C.; Holmes, J. M.; Kociok-Kohn, G.; Wrigley, G. L.; Cresswell, A. J. *J. Am. Chem. Soc.* **2021**, *143*, 15936–15945.

2 Nawrat, C. C.; Jamison, C. R.; Slutskyy, Y.; MacMillan, D. W. C.; Overman, L. E. *J. Am. Chem. Soc.* **2015**, *137*, 11270–11273.

3 The $T_1$ relaxation time of the oxetane methylene signals of standard product **2a**, which were used for the calculation of *in situ* yields, was determined to be 5 s. Thus, a 30 s delay between pulses were used to determine accurate *in situ* yields of **2a**.

4 Chu, L.; Ohta, C.; Zuo, Z.; MacMillan, D. W. C. *J. Am. Chem. Soc.* **2014**, *136*, 10886–10889.

5 Lowry, M. S.; Goldsmith, J. I.; Slinker, J. D.; Rohl, R.; Pascal, R. A.; Malliaras, G. G.; Bernhard, S. *Chem. Mater.* **2005**, *17*, 5712–5719.

6 Prier, C. K.; Rankic, D. A.; MacMillan, D. W. C. *Chem. Rev.* **2013**, *113*, 5322–5363.

7 Ryder, A. S. H.; Cunningham, W. B.; Ballantyne, G.; Mules, T.; Kinsella, A. G.; Turner-Dore, J.; Alder, C. M.; Edwards, L. J.; McKay, B. S. J.; Grayson, M. N.; Cresswell, A. J. *Angew. Chem. Int. Ed.* **2020**, *59*, 14986–14991.

8 Pitzer, L.; Schafers, F.; Glorius, F. *Angew. Chem. Int. Ed.* **2019**, *58*, 8572–8576.

9 Dubois, M. A. J.; Smith, M. A.; White, A. J. P.; Lee Wei Jie, A.; Mousseau, J. J.; Choi, C.; Bull, J. A. *Org. Lett.* **2020**, *22*, 5279–5283.

10 Neese, F.; Wennmohs, F.; Becker, U.; Riplinger, C. *J. Chem. Phys.* **2020**, *152*, 224108.

11 Young, T. A.; Silcock, J. J.; Sterling, A. J.; Duarte, F. *Angew. Chem. Int. Ed.* **2021**, *60*, 4266–4274.

12 Riniker, S.; Landrum, G. A. *J. Chem. Inf. Model.* **2015**, *55*, 2562–2574.

13 Bannwarth, C.; Caldeweyher, E.; Ehlert, S.; Hansen, A.; Pracht, P.; Seibert, J.; Spicher, S.; Grimme, S. *WIREs Mol. Sci.* **2021**, *11*, e1493.

14 (a) Adamo, C.; Barone, V. *J. Chem. Phys.* **1999**, *110*, 6158–6170. (b) Perdew, J. P.; Burke, K.; Ernzerhof, M. *Phys. Rev. Lett.* **1996**, *77*, 3865–3868. (c) Grimme, S.; Antony, J.; Ehrlich, S.; Krieg, H. *J. Chem. Phys.* **2010**, *132*, 154104. (d) Grimme, S.; Ehrlich, S.; Goerigk, L. *J. Comput. Chem.* **2011**, *32*, 1456–1465. (e) Weigend, F.; Ahlrichs, R. *Phys. Chem. Chem. Phys.* **2005**, *7*, 3297–3305.

15 (a) Lin, Y.-S.; Li, G.-D.; Mao, S.-P.; Chai, J.-D. *J. Chem. Theory Comput.* **2013**, *9*, 263–272. (b) Chai, J.-D.; Head-Gordon, M. *Phys. Chem. Chem. Phys.* **2008**, *10*, 6615–6620. (c) Neese, F.; Wennmohs, F.; Hansen, A.; Becker, U. *Chem. Phys.* **2009**, *356*, 98–109.

16 Isegawa, M.; Neese, F.; Pantazis, D. A. *J. Chem. Theory Comput.* **2016**, *12*, 2272–2284.

17 Tom Young **2020**. duartegroup/otherm: Major symmetry improvements (1.0.0-beta). Zenodo. https://doi.org/10.5281/zenodo.4005686.

18 Grimme, S. *Chem. Eur. J.* **2012**, *18*, 9955–9964.

19 Hirshfeld, F. L. *Theor. Chim. Acta* **1977**, *44*, 129–138.

20 Reed, A. E.; Weinstock, R. B.; Weinhold, F. *J. Chem. Phys.* **1985**, *83*, 735–746.

21 Hammerich, O.; Speiser, B. Techniques For Studies Of Electrochemical Reactions In Solution. In *Organic Electrochemistry*, 5th ed; Hammerich, O.; Speiser, B. Ed.; CRC Press, Boca Raton, 2015; pp. 98–168.

22 Gagne, R. R.; Koval, C. A.; Lisensky, G. C. *Inorganic Chemistry*. **1980**, *19*, 2854–2855.

23 Pavlishchuk, V. V.; Addison, A. W. *Inorganica Chim. Acta* **2000**, *298*, 97–102.

24 Leech, M. C.; Lam, K. *Nat. Rev. Chem.* **2022**, *6*, 275–286.

25 For a description on how to determine $E_{p/2}$: Roth, H.; Romero, N.; Nicewicz, D. *Synlett* **2015**, *27*, 714–723.

26 R Core Team. *R: A language and environment for statistical computing*, version 3.2.2; R Foundation for Statistical Computing: Vienna, Austria, 2015.

27 Capaldo, L.; Buzzetti, L.; Merli, D.; Fagnoni, M.; Ravelli, D. *J. Org. Chem.* **2016**, *81*, 7102–7109.

28 Hansch, C.; Leo, A.; Taft, R. W. *Chem. Rev.* **1991**, *91*, 165–195.

29 Tyssee, D. A.; Baizer, M. M. *J. Org. Chem.* **1974**, *39*, 2819–2823.

30 Arnold, D. R.; Borg, R. M.; Albini, A. *J. Chem. Soc. Chem. Commun.* **1981**, 138–139.
31 Schmittel, M.; Lal, M.; Lal, R.; Röck, M.; Langels, A.; Rappoport, Z.; Basheer, A.; Schlirf, J.; Deiseroth, H.-J.; Flörke, U.; Gescheidt, G. *Tetrahedron* **2009**, *65*, 10842–10855.
32 Allwood, D. M.; Blakemore, D. C.; Brown, A. D.; Ley, S. V. *J. Org. Chem.* **2014**, *79*, 328–338.
33 Pretsch, E.; Bühlmann, P.; Badertscher, M. in *Spektroskopische Daten zur Strukturaufklärung organischer Verbindungen*, 5th ed.; Springer, Berlin Heidelberg, **2010**.
34 Yang, Y.; Zhou, Q.; Cai, J.; Xue, T.; Liu, Y.; Jiang, Y.; Su, Y.; Chung, L.; Vicic, D. A. *Chem. Sci.* **2019**, *10*, 5275–5282.
35 Su, X.; Fox, D. J.; Blackwell, D. T.; Tanaka, K.; Spring, D. R. *Chem. Commun.* **2006**, 3883–3885.
36 Kurono, N.; Sugita, K.; Takasugi, S.; Tokuda, M. *Tetrahedron* **1999**, *55*, 6097–6108.
37 Hartmann, R. W.; Kranzfelder, G.; Von Angerer, E.; Schoenenberger, H. *J. Med. Chem.* **1980**, *23*, 841–848.
38 Resendiz, M. J. E.; Garcia-Garibay, M. A. *Org. Lett.* **2005**, *7*, 371–374.
39 (a) Garreau, M.; Le Vaillant, F.; Waser, J. *Angew. Chem. Int. Ed.* **2019**, *58*, 8182–8186. (b) Ryder, A. S. H.; Cunningham, W. B.; Ballantyne, G.; Mules, T.; Kinsella, A. G.; Turner-Dore, J.; Alder, C. M.; Edwards, L. J.; McKay, B. S. J.; Grayson, M. N.; Cresswell, A. J. *Angew. Chem. Int. Ed.* **2020**, *59*, 14986–14991.
40 Engle, S. M.; Kirkner, T. R.; Kelly, C. B. *Org. Synth.* **2019**, *96*, 455–473.
41 Oxetane acid **15** not taken into this analysis.
